# Suppression of SENP3 enhances macrophage alternative activation by mediating IRF4 de-SUMOylation in ESCC progression

**DOI:** 10.1186/s12964-024-01770-z

**Published:** 2024-08-09

**Authors:** Shaoyuan Zhang, Jianmin Gu, Wenhan Wang, Linyi Sun, Tian Jiang, Xinyu Yang, Jun Yin, Miao Lin, Dong Lin, Hao Wang, Lijie Tan

**Affiliations:** 1 Department of Thoracic Surgery, Zhongshan Hospital, Fudan University, Xuhui District, No. 180, Fenglin Road, Shanghai, 200032 People’s Republic of China; 2 Cancer Center, Zhongshan Hospital, Fudan University, Shanghai, 200032 People’s Republic of China; 3 Department of Biochemistry and Molecular Cell Biology, Shanghai Key Laboratory for Tumor Microenvironment and Inflammation, Shanghai Jiao Tong University School of Medicine, Shanghai, 200025 People’s Republic of China; 4 Department of Thoracic Surgery, Shanghai General Hospital, Shanghai Jiaotong University School of Medicine, Hongkou District, No. 100, Haining Road, Shanghai, 200080 People’s Republic of China

**Keywords:** Esophageal squamous cell carcinoma (ESCC), SENP3, Tumor-associated macrophage, IRF4, Alternative activation, SUMOylation, De-SUMOylation

## Abstract

Esophageal cancer is common worldwide, with ESCC being the most frequent tumor in East Asia. Tumor-associated macrophages are an important component of the ESCC microenvironment. SUMOylation is a post-translational modification of proteins, and SUMO-specific proteases (SENPs) play an important role in de-SUMOylation. In human patients, we discovered that the levels of SENP3 were upregulated in the tumor-associated macrophages. Furthermore, the loss of SENP3 enhanced the alternative activation of macrophages in the 4-NQO-induced ESCC mice model. This is the first study to identify SENP3-mediated macrophage polarization via the de-SUMOylation of interferon regulatory factor 4 (IRF4) at the K349 site. Alternative activation of macrophages increases the migration and invasion potential of ESCC cells and promotes their progression *in vivo*. Moreover, patients with relatively low SENP3 expression in macrophages exhibit higher primary PET SUVmax value and lymph node metastasis rates. In summary, this study revealed that SENP3-mediated IRF4 de-SUMOylation is crucial for the alternative activation of macrophages and influences the progression of ESCC.

**Graphical Abstract:**

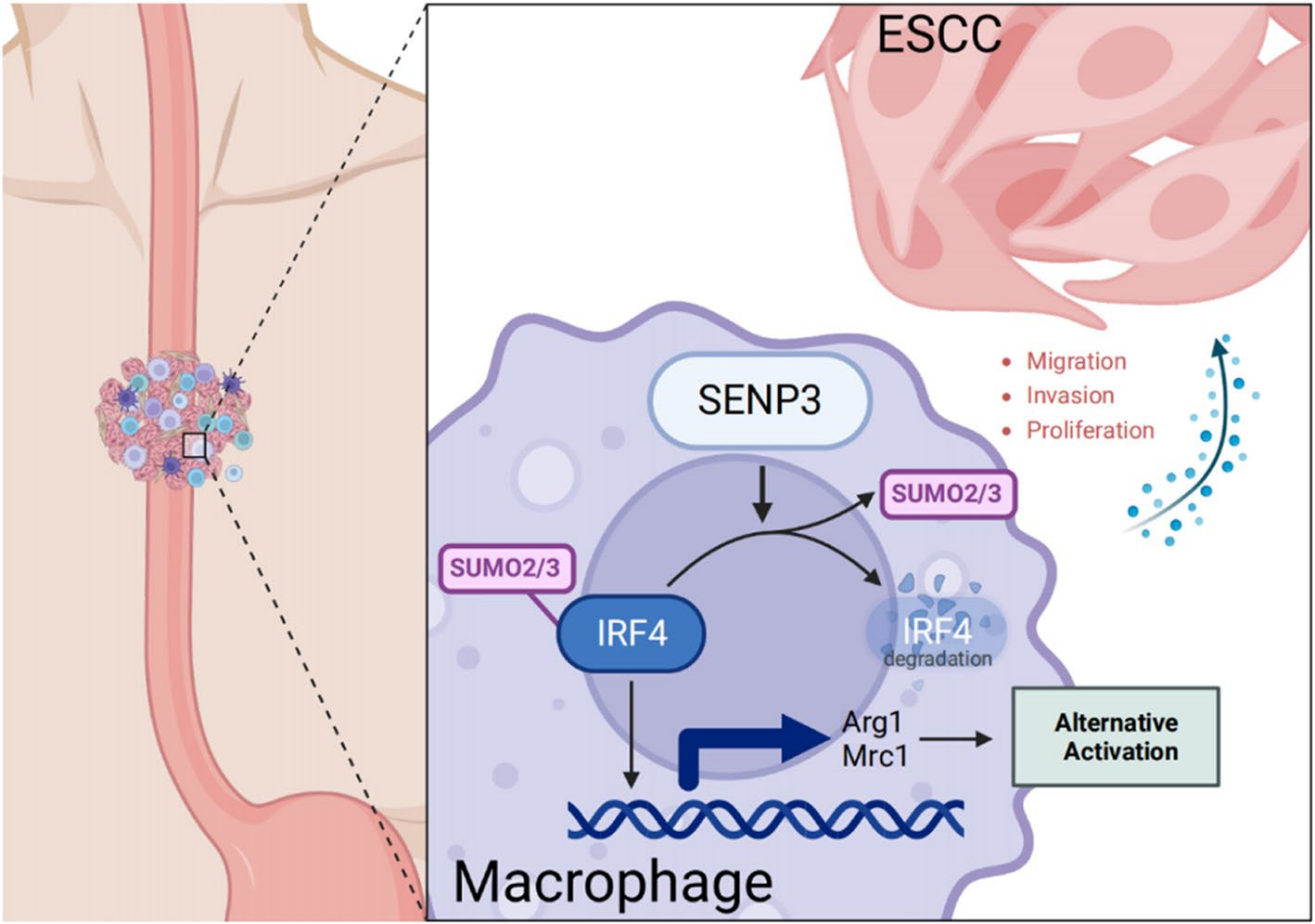

**Supplementary Information:**

The online version contains supplementary material available at 10.1186/s12964-024-01770-z.

## Highlights

• SENP3 expression is upregulated in macrophages in esophageal squamous cell carcinoma.

• The absence of SENP3 in macrophages promotes the progression of esophageal squamous cell carcinoma both in patients and in the 4-NQO-induced ESCC mice model.

• Loss of SENP3 promotes macrophages’ alternative activation by attenuating the de-SUMOylation of IRF4 at the K349 site.

• ESCC patients with relatively low SENP3 expression in macrophages had higher primary tumor SUVmax and lymph node metastasis rates.

**Supplementary Information:**

The online version contains supplementary material available at 10.1186/s12964-024-01770-z.

## Introduction

Esophageal cancer (EC) is one of the most common digestive tract tumors that threatens the health of Chinese people [1]. EC subtypes exhibit racial differences, for example, esophageal squamous cell carcinoma (ESCC) is the most common type in East Asia, including China, accounting for >90% of all EC [2]. Since ESCC has an insidious onset and lacks a specific biomarker, most patients are often presented with locally advanced tumors at diagnosis. Furthermore, ESCC has poor overall survival.

Tumor-associated macrophages are an important component of ESCC [3]. They are inflammatory cell subpopulations with the highest proportion in tumor microenvironment (TME), modulating tumor occurrence and development [4, 5]. Furthermore, macrophage subpopulations play a significant role in the occurrence and progression of ESCC. Macrophage-related markers, such as CD68, CD204, and CD206, have been used to identify tumor-associated macrophages in the ESCC microenvironment [6–8]. Cytokines secreted by macrophages are crucial for promoting the proliferation, metastasis, and ESCC immune evasion [3, 9]. Therefore, macrophages have become a particularly important target in tumor treatment.

Small ubiquitin-like Modifier (SUMO) proteins are a family of small proteins that can modify their functions by covalently connecting or dissociating with other proteins in cells, affecting the activity and stability of substrates (proteins) and playing an important role in inflammation or diseases such as tumors [10, 11]. SUMOylation modification is a dynamic and reversible modification; however, de-SUMOylation modification relies on the SUMO-specific protease family (SENPs). SENP3 is a critical SENP family component that can remove the SUMO2/3 modification of substrate proteins, thereby altering downstream gene expression or protein function in cells [12]. SUMOylation modification of substrate proteins in monocytes and macrophages strongly influences cell development and differentiation. However, the reports on SUMO substrate proteins in ESCC-associated macrophages and the underlying mechanism are limited.

This study demonstrates that SENP3 accumulation in ESCC-associated macrophages promotes SENP3-IRF4 interaction and de-SUMOylation of IRF4, whereas SENP3-specific deletion promotes IRF4 accumulation, increasing alternative activation of macrophages, which inhibits immune microenvironment. Patients with relatively low expression of SENP3 in macrophages have an increased risk of lymph node metastasis. All these data indicate that SENP3 is essential for ESCC-associated macrophages, and its increased activity could be a promising strategy for tumor immunotherapy.

## Method

### Patient cohort, tissue specimen, and tumor microarrays

This retrospective study enrolled EC patients who underwent surgery at Zhongshan Hospital Fudan University from January 2015 to January 2019. The inclusion criteria included patients who 1) were older than 18 years, 2) had ESCC confirmed by two pathologists, and 3) did not receive any preoperative neoadjuvant therapy. All patients had detailed preoperative PET-CT imaging (as reported in the literature) and underwent surgical resection with negative margins (pR0). Those with multifocal or diffuse tumors were excluded. This study was approved by the Ethics Committee of Zhongshan Hospital of Fudan University (B2022-271R), and informed consent was acquired for clinical and biological information. Tumor tissue microarrays were prepared using the paraffin blocks according to previous reports [13]. Patients’ ESCC and paired paracancerous tissues were obtained by surgery and were used fresh. Macrophages will be sorted by flow cytometry (BD FACS Aria II, BDBiosciences, USA) after the digestion of fresh tissue. None of the specimens received neoadjuvant therapy.

### Mice and esophageal squamous cell carcinoma model

To create macrophage-specific *Senp3* cKO mice (*Senp3* flox/flox × *Lyz2*-Cre), *Senp3* flox/flox mice [kindly donated by Professor J. Yi [13] were crossed with *Lyz2*-Cre mice [Jackson Laboratory; purchased from Cyagen Biosciences (Suzhou, China)] (Fig. S2B and S2C). All mice were bred and maintained under specific pathogen-free (SPF) conditions on a 12-h reverse light/dark cycle at 22 ± 2°C temperature and 40–70% humidity. Each independent experiment used age- and sex-matched male and female adult (6-8 weeks old) mice. Animal experiments strictly followed the "Guidelines for the Care and Use of Laboratory Animals" and were approved by the Experimental Animal Ethics Committee of Shanghai Jiao Tong University School of Medicine. *Senp3* cKO and flox/flox mice were treated with 100 mg/L of 4-NQO (N8141, Sigma) dissolved in drinking water for 16 weeks and then with normal drinking water for 12 weeks (Fig. 2G).

### Reverse transcription-quantitative real-time PCR (RT-qPCR)

Total RNA was extracted using TRIzol reagent (Invitrogen, Carlsbad, CA, USA). cDNA template was synthesized using the Hifair II 1st Strand cDNA Synthesis Kit (Yeasen Biotechnology, Shanghai, China), and qRT-PCR was performed on LightCycler 96 instrument (Roche, Basel, Switzerland) using SYBR Green Master Mix (Yeasen Biotechnology, Shanghai, China). For mRNA quantification, the 2^-ΔΔCT^ method was used. GAPDH served as an endogenous control. The primer sequences used for qRT-PCR are shown in the Supplementary Table.

### Western blotting

Cellular proteins were extracted using WB/IP Lysis buffer (Yeasen Biotechnology, Shanghai, China) with protease and phosphatase inhibitors and quantified using Pierce BCA Protein Assay Kit (Thermo Scientific, USA). The proteins were separated by SDS-PAGE, transferred onto PVDF membranes (0.2 µm, Merck Millipore, Burlington, MA, USA), blocked with 5% non-fat milk, and then incubated with primary antibodies and horseradish peroxidase-conjugated secondary antibody. ImageJ was used for the analysis of gray values.

### Flow cytometry

Adherent M2 macrophages were digested using ACCUTASE, washed, and then incubated with Brilliant Violet 421™ anti-human CD206 (MMR) antibody (BioLegend, San Diego, CA, USA, 321126) for 30 minutes at room temperature. Subsequently, 7-AAD (BD, 559925) was added, and analysis was performed by Beckman CytoFlex S (Brea, CA, USA). Secretome quantitatively analysis of is performed on ABplex Human 8-Plex Custom Panel (CCL5/RANTES, GM-CSF, IL-1 beta, IL-4, IL-6, IL-10, TNF-alpha, TGF-beta1) and tested by ABplex-100 (ABclonal Technology, WuHan, China).

### Lentiviral constructs and lentivirus packaging

*SENP3* shRNA lentivirus was constructed by VectorBuilder Inc. (Guangzhou, China), and lentiviral particles are packaged as previously described. HEK293FT cells were co-transfected with lentiviral and packaging vectors using Hieff Trans® liposome transfection reagent (Yeasen Biotechnology, Shanghai, China). Virus particles were harvested 48 – 72 h after transfection. The shNC group was transfected with a plasmid that did not contain our shRNA sequence and served as a Vector Control.

### Immunohistochemistry (IHC)

Immunohistochemistry was performed on paraformaldehyde-fixed and paraffin-embedded sections of ESCC tissues and normal esophageal tissues from patients. The primary antibodies are shown in the Supplementary Materials-Antibody List. Secondary antibodies (1:200) were incubated for 2 h. The Ki-67 positive control was a known positive tissue section (Late-Stage ESCC tissue slides), and PBS was used instead of the primary antibody as a negative control. The sections were detected using the DAB detection system (Vector Laboratories, Burlingame, CA, USA) and counterstained with hematoxylin. Images were captured using slide-scanners (KFBio, Ningbo, China), and the average optical density of at least three fields of view for each image was quantified and statistically analyzed using ImageJ software (NIH, Bethesda, MD, USA).

### Co-immunoprecipitation (Co-IP)

The Co-IP was performed using methods described previously (Huang et al., 2009). Briefly, for 30 min, the cells were lysed in IP lysis buffer (Yeasen Biotechnology, Shanghai, China) at 4°C and then centrifuged for 15 min at 4°C and 13000 g. Anti-Flag or Protein A/G magnetic beads (MedChem Express, USA) were applied for immunoprecipitation. For Protein A/G magnetic beads, specific antibodies were incubated overnight at 4 ℃ before incubation with whole cell lysates at 4 ℃ for 4 hours. Anti-Flag magnetic beads were incubated with whole cell lysate overnight at 4℃. The beads were then washed 5 times, mixed with 1x loading buffer, and examined by immunoblot.

### ECAR and OCR analysis

The extracellular acidification rate (ECAR) and cellular oxygen consumption rate (OCR) were determined using the Seahorse XFe96 analyzer (Seahorse Bioscience, Billerica, MA, USA). Briefly, the cells were seeded in 96-well plates (4×10^5^/well). For ECAR detection, glucose (10 mM), oligomycin (1 μM), and 2-deoxyglucose (50 mM) were added to test plates of Seahorse XFe96. For OCR assay, oligomycin (5 μM), Carbonyl cyanide-p-trifluoromethoxy phenylhydrazone (FCCP) (optimal concentration: 0 to 2.0 μM), and Antimycin A/Rotenone (Rote +AA; 0.5 μM) were added to test plates.

### Statistics analysis

Statistical analysis was performed using GraphPad Prism 9.4 (GraphPad Software, La Jolla, CA, USA) and R (V4.2.1, R Foundation, Vienna, Austria). All data are expressed as mean ± standard deviation (SD). Student's t-test and ANOVA were used to analyze differences between two or more groups. Logistic regression was used to analyze the relationship between gene expression levels and clinicopathological characteristics. Kaplan-Meier analysis was used to evaluate patient survival differences. The data were statistically processed: *p value <0.05, **p value <0.01, ***p value <0.001, and two-sided p value <0.05 were considered statistically significant.

## Result

### SENP3 is upregulated in macrophages in esophageal squamous cell carcinoma

**Fig. 1:**
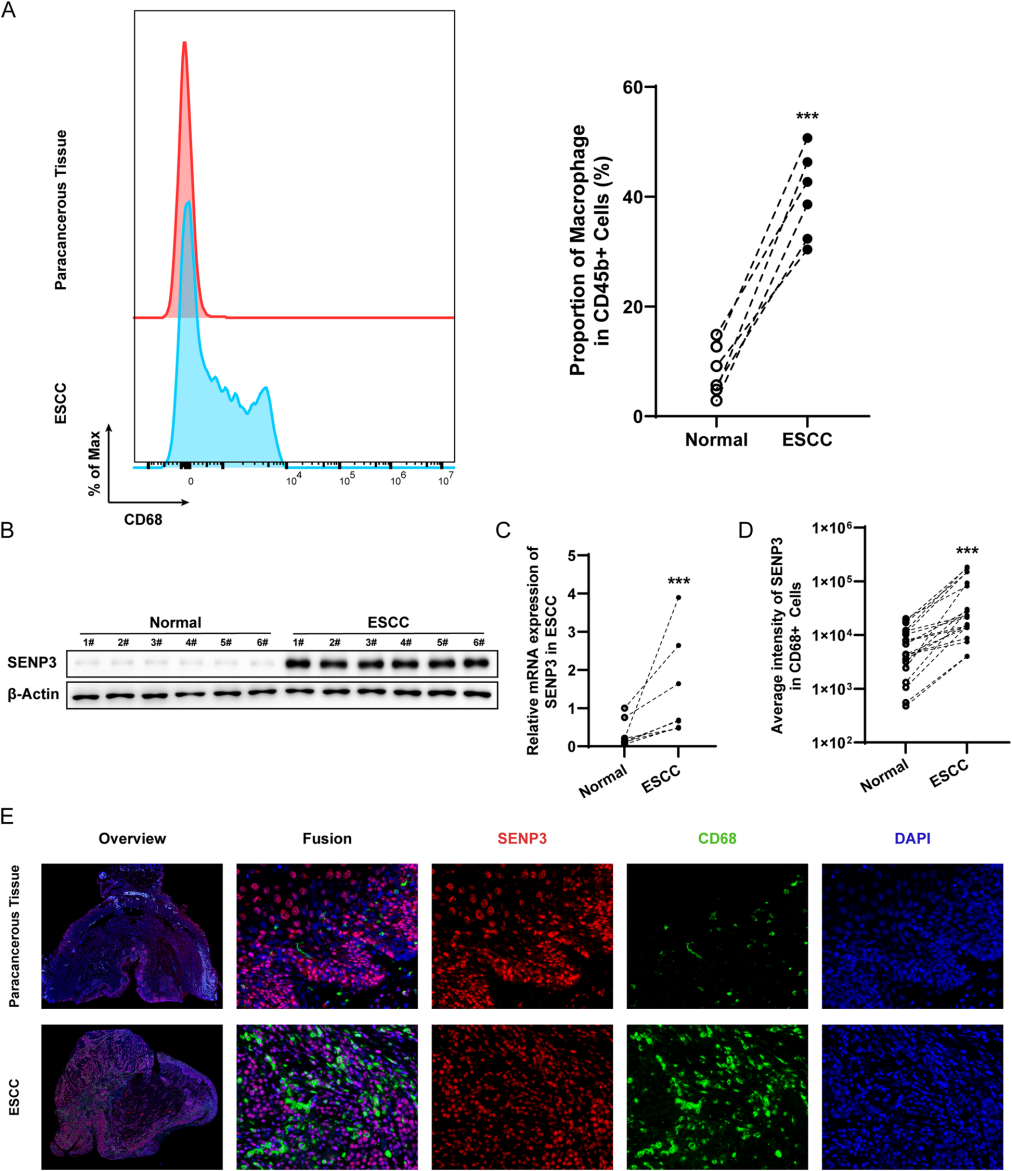
SENP3 is upregulated in macrophages in esophageal squamous cell carcinoma. **A** The proportion of macrophages increase in ESCC. **B** Western blot analysis was performed on 1 × 10^6^ macrophage whole-cell lysates freshly isolated from 6 pairs of paracancer and ESCC tissues from patients. **C** Quantification of SENP3 expression in patient-derived macrophages from paracancer and ESCC tissue from patients (*n*=8). **D** Compared with normal tissue, the average SENP3 fluorescence intensity in CD68+ cells in ESCC increased significantly (*n*=20). **E** Multiple immunofluorescences show that SENP3 is up-regulated in ESCC-associated macrophages than the control paracancerous tissues

To study the ESCC-related microenvironment, we isolated macrophages from adjacent tumor and ESCC tissues and then performed Western blot analysis. Interestingly, the proportion of macrophages in ESCC was significantly increased compared with that in paracancerous tissues (Fig. 1A and S1A), while at the same time, SENP3 was significantly accumulated in macrophages in ESCC tissues (Fig. 1B and C). To further verify the localization of SENP3, we performed multiple immunofluorescence staining, and the results showed that SENP3 was significantly upregulated in CD68-positive cells in tumor tissues compared with normal tissues (Fig. 1D and E), which is a common marker of macrophages. In addition, SENP3 in EC macrophages was gradually upregulated compared with normal squamous epithelium. We further found that SENP3 in esophageal squamous cell carcinoma was mainly expressed in CD206+ macrophages (Fig. S1B).

#### Loss of SENP3 in macrophages promotes ESCC progression *in vivo* and mouse models

**Fig. 2:**
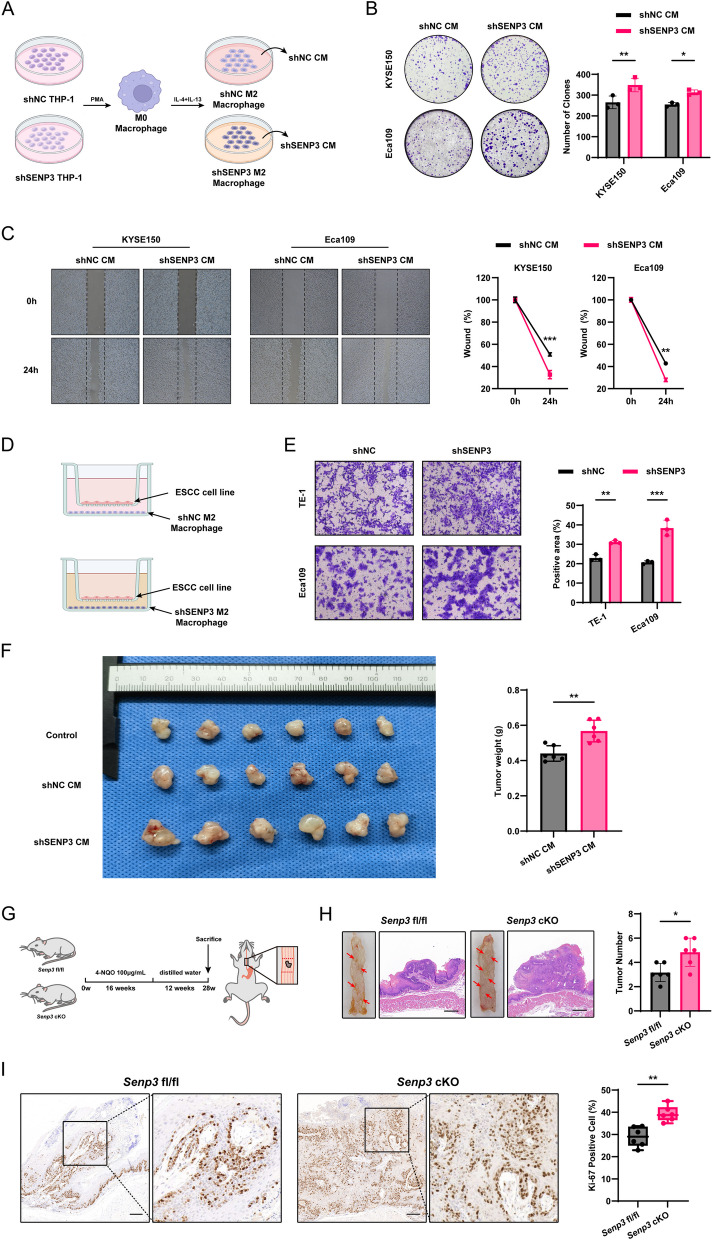
Deletion of SENP3 in macrophages promotes ESCC progression *in vivo* and mouse model. **A** Schematic diagram of shNC and sh*SENP3* M2 macrophage cell culture and acquisition of conditioned medium. **B** The proliferation ability of KYSE150 and Eca109 cell lines was enhanced after treatment with sh*SENP3* M2 conditioned medium. **C** sh*SENP3* M2 CM can improve the migration ability of KYSE150 and Eca109 cell lines compared with shNC M2 CM. **D** Schematic diagram of M2 macrophages and ESCC cell co-culture. **E**. M2 macrophages and ESCC cells were co-cultured in Transwell chambers, which revealed that sh*SENP3* treatment groups could increase the migration ability of ESCC cells. **F** Tumor cells treated with shNC and sh*SENP3* CM were subjected to tumor formation in nude mice; sh*SENP3* CM indicated to increase in the proliferation of ESCC cells. **G** *Senp3* fl/fl and *Senp3* cKO mouse ESCC animal models were constructed with the 4-NQO drinking water method. **H** All mice were sacrificed at 28 weeks, and the mouse esophagus was carefully dissected. *Senp3* cKO mice showed more tumors than *Senp3* fl/fl mice (*n*=6). **I** Immunohistochemical staining was performed on *Senp3* cKO and *Senp3* fl/fl mice, indicating that the Ki-67 positive rate was higher in the *Senp3* cKO group

SENP3 in macrophages is crucial for their anti-tumor function, and this study indicated its up-regulation during ESCC. To assess the impact of SENP3 in macrophage on ESCC, SENP3 was knocked down in THP-1 through lentivirus. Conditioned medium was then collected upon macrophage alternative activation induced by PMA, IL-4 and IL-13 *in vitro* (Fig. 2A). Compared with the NC group, the clonogenic ability of Eca109 and KYSE150 after treated with sh*SENP3* conditioned medium was significantly enhanced (Fig. 2B). Moreover, the conditioned media were used for wound healing, and it was found that the sh*SENP3*-treated cells exhibited enhanced migration ability (Fig. 2C). Subsequently, a Transwell chamber was used to observe the effect of macrophages specifically lacking SENP3 on ESCC cells in co-culture (Fig. 2D). The results showed that after treatment, ESCC-sh*SENP3* had a significantly increased migration ability (Fig.2E). Additionally, the conditioned medium-treated cell lines were subjected to nude mouse tumorigenesis experiments, and the *in vivo* proliferation potential was compared. ESCC cell lines tamed by shSENP3 macrophages could significantly promote tumor formation (Fig. 2F). To further study the role of SENP3 in macrophages *in vivo*, *Senp3* fl/fl and *Senp3* fl/fl *Lyz2*-Cre (*Senp3* cKO) mice were treated with 4-NQO (100 mg/L) (Fig. 2G). Interestingly, specific SENP3 inhibition in macrophages promoted tumor growth (Fig. 2H). The immunohistochemical staining of Ki-67 on the mouse esophagus revealed that compared with *Senp3* fl/fl mice, the proportion of Ki-67 positive cells in the embassy tissue of *Senp3* cKO mice was higher. These data indicate that specific deletion of SENP3 in macrophages promotes ESCC both *in vivo* and *in vitro*, indicating its anti-tumor effect.

#### The absence of SENP3 in macrophages promotes the alternative activation of macrophages in ESCC

**Fig. 3:**
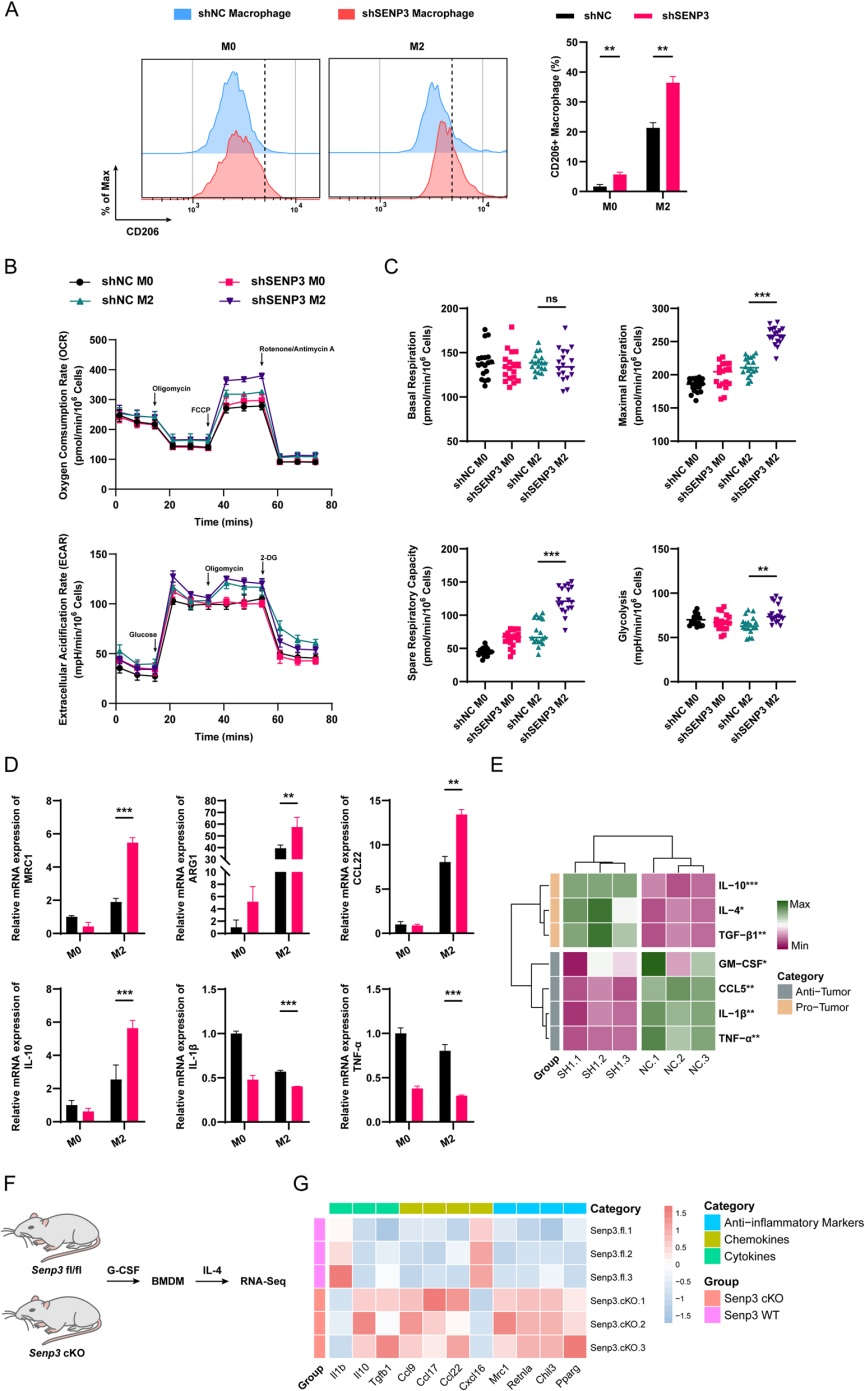
The absence of SENP3 in macrophages promotes the alternative activation of macrophages in ESCC. **A** Flow cytometry shows that CD206+ cells in M2 macrophages stimulated by IL-4 and IL13 are increased in the sh*SENP3* group compared with the shNC group. **B** The Seahorse Extracellular Flux Analyzer XFe96 assay determined the ECAR and OCR, which reflect differences in the intracellular metabolism of shNC and sh*SENP3* in M2 macrophages. **C** Comparison of basal respiration, maximal respiration, spare respiratory capacity, and glycolytic capacity in shNC M0, shNC M2, sh*SENP3* M0, sh*SENP3* M2 macrophages. **D** The relative mRNA amounts of *MRC1*, *ARG1*, *CCL22*, *IL-10*, *IL-1β*, and *TNF-α* were detected for M0 and M2 macrophages in the shNC group and sh*SENP3* group, respectively. **E** Heatmap of secretome analysis (ABplex Human 8-Plex Custom Panel) of shNC and sh*SENP3* M2 macrophage media. **F** Schematic diagram indicating the acquisition of M2 macrophage from the *Senp3* fl/fl and *Senp3* cKO mice. **G** RNA sequencing analysis of BMDM induced into M2 macrophages from *Senp3* fl/fl and *Senp3* cKO mice showing a heatmap of alternative activation-related genes

The M2 type (alternative activation) of macrophages plays a significant role in promoting tumors. We used CD206 as a marker to elucidate alternative activation. Flow cytometry revealed that in M0-type macrophages, the loss of SENP3 can increase CD206 positivity. Cell ratio and CD206-positive cells increased significantly after IL-4/IL-13 treatment compared with the control group, indicating that SENP3 is crucial for the alternative activation of macrophages (Fig. 3A). We also tested the amount of M1 macrophage and found that the M1 polarization level in sh*SENP3* group was reduced compared with shNC group (Fig. S1E). Compared with M1, M2 macrophages heavily rely on glycolysis and mitochondrial respiration, and their levels reflect the degree of alternative activation. To determine if SENP3 deficiency alters energy homeostasis and thereby affects alternative activation, M2 macrophages were obtained by IL-4/IL-13 stimulation, and M0 macrophages were obtained as control, and then their ECAR and OCR were evaluated (Fig. 3B). ECAR and maximal respiratory capacity (Fig. 3C) were increased in KD cells, indicating upregulation of both glycolysis and mitochondrial respiration levels, which are critical for alternative activation. Interestingly, OCR and spare respiratory volume were also upregulated, suggesting that lipolysis is also affected. Altogether, these data indicate that SENP3 deficiency promotes macrophage glycolysis and mitochondrial respiration, thereby enhancing alternative activation processes. Additionally, qRT-PCR revealed that alternative activation-related genes, including *MRC1*, *ARG1*, *CCL22*, *IL-10*, etc., were up-regulated in SENP3-deficient macrophages(Fig. 3D).

The secretome testing on the cell supernatant of the SENP3 deletion group indicated down-regulation of the anti-tumor factors (CCL5 and IL-β) and up-regulation of tumor-promoting factors (IL-10 and TGF-β) (Fig. 3E). These suggest that specific deletion of SENP3 in macrophages promotes alternative activation and creates a suppressive immune environment. Subsequently, BMDM was extracted from *Senp3* flox/flox and *Senp3* cKO mice, and M2 macrophages were acquired through G-CSF and Il-4 stimulation *in vitro* (Fig. 3F). RNA sequencing data showed that Senp3 deletion promoted several cytokines. Furthermore, the expression of chemokines, including *Ccl17*, *Ccl22*, etc. (Fig. 3G), has also been reported to promote the progression of ESCC. In summary, SENP3 in macrophages is crucial for their alternative activation, and the SENP3 reduction creates a suppressive immune microenvironment.

#### SENP3 mediates IRF4 de-SUMOylation at K349 site

**Fig. 4:**
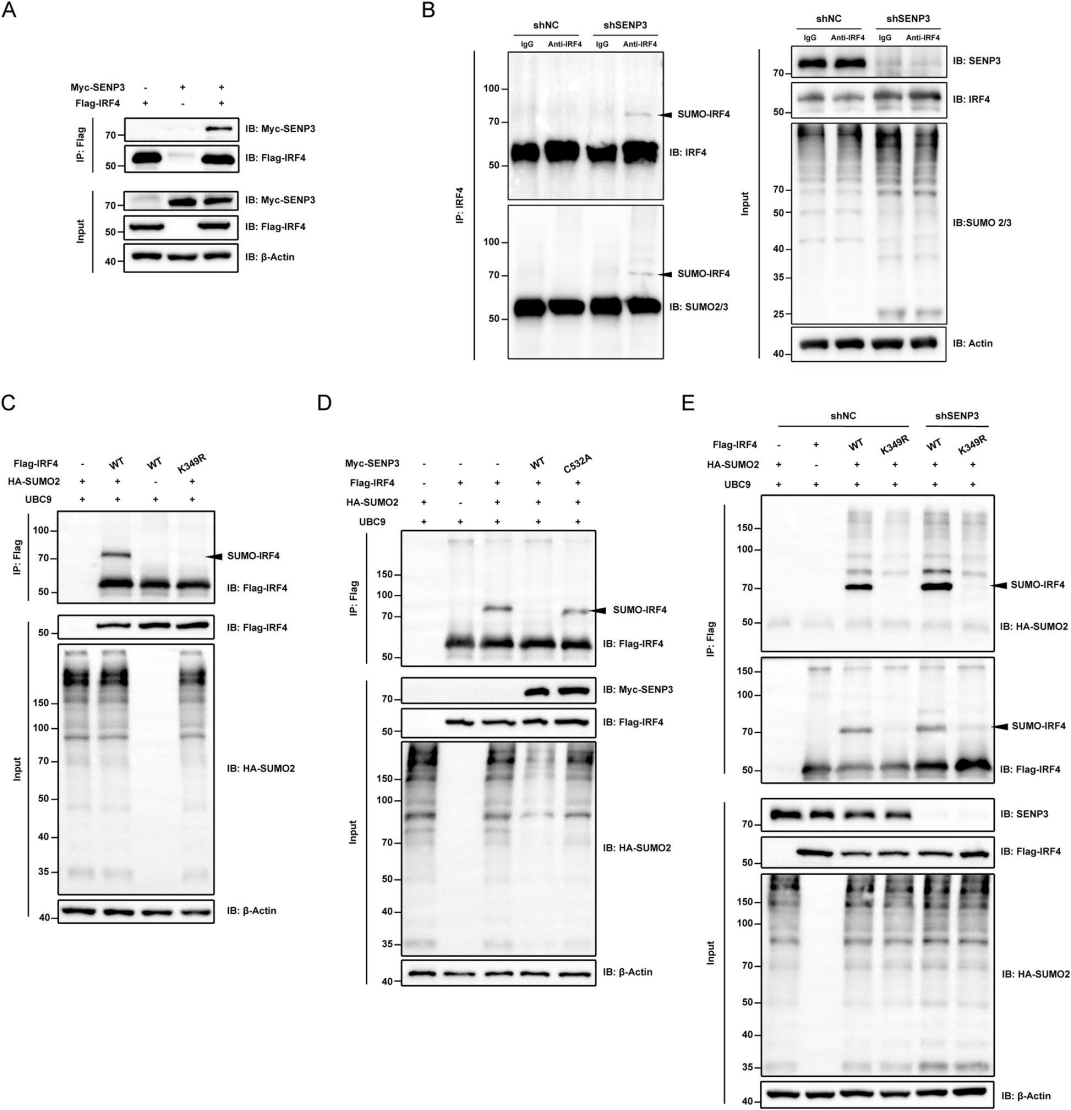
SENP3 mediates IRF4 de-SUMOylation at K349 site. **A** Exogenous interaction of SENP3 with IRF4 determined by Co-IP. HEK293T cells were transfected with Myc-SENP3 and FLAG-IRF4 plasmids for 48 hours. For Co-IP, anti-Flag magnetic beads were used whereas for immunoblotting (IB), FLAG and Myc antibodies were used. **B** Endogenous SUMOylation of IRF4 in M2 macrophages. After PMA stimulation for 48 h, THP-1 cells were stimulated with IL-4 and IL-13 for a further 48 h to acquire M2 macrophages. Whole-cell lysates were used for pull-down assays. Co-IP was performed with IRF4 antibody, and IB was performed with SUMO2/3, SENP3, and IRF4 antibodies. **C** IRF4 can be SUMOylated at K349. Pull-down assays were performed using Flag antibodies by exogenous addition of HA-SUMO2, UBC9, and Flag-IRF4. For IB, anti-HA and anti-Flag antibodies were utilized. **D** IRF4 can be SUMOylated at K349 by SENP3. Anti-Flag Magnetic Beads determined conjugates of SUMO2 and IRF4. FLAG-IRF4, HA-SUMO2, and UBC9 were co-transfected with Myc-SENP3 or Myc-SENP3 C532A mutant into HEK293T cells for 48 h. Flag-IRF4 was pulled down using Flag Magnetic Beads and then analyzed by IB using anti-Myc, anti-HA, and anti-Flag Antibodies. **E** Endogenous SENP3 was knocked down using the lentiviral in HEK293T cells co-transfected with FLAG-IRF4 WT or mutated FLAG-IRF4 K349R, HA-SUMO2, and UBC9 for 48 h. Cells were lysed, Flag-IRF4 was pulled down using anti-Flag Magnetic Beads, and analyzed by IB using anti-HA and anti-Flag Antibodies. Arrows indicate SUMO2-conjugated IRF4 (B, C, D, and E)

To study the molecular mechanism of *Senp3* cKO mice promoting ESCC, the SENP3 substrates in macrophages were identified. Our previous studies found that SUMO2/3 modification of specific transcription factors increases their activity [14], and this activity is reversed by SENP3-mediated de-SUMOylation. Based on the protein SUMOylation prediction system [15], the potential modification of alternative activation-related transcription factors by SUMO2/3 was assessed *in vitro*. SUMO prediction software (http://www.jassa.fr/) was employed to screen the factors related to alternative activation and found that interferon regulatory factor 4 (IRF4) had a high SUMO modification score. To verify if the modification of IRF4 is by SUMO2/3 during macrophage activation and formation. First, the binding of SENP3 and IRF4 with each other was assessed *in vitro* through Co-IP experiments using tag proteins (Fig. 4A). Then Co-IP was performed for M0 and alternatively activated macrophages, respectively. The results showed that after alternative activation, IRF4 was significantly modified by SUMO2/3 (Fig. 4B). Furthermore, on gels with a molecular weight of 70 kDa, SUMO2/3-conjugated IRF4 showed a prominent band, indicating single-SUMO protein conjugation.

To further study the de-SUMOylation effect of SENP3 on IRF4, the HEK293T overexpression system was employed. Based on previous literature, the K349 site of IRF4 was found to have a significant impact on macrophage polarization [16]. Therefore, a lysine-349-arginine mutated IRF4 plasmid was constructed to determine the specific amino acids on IRF4 modified by SUMO2. SUMOylation experiments showed that SUMO2 modification on IRF4 was abolished when K349 was mutated to R (Fig. 4C). Subsequently, SENP3 WT and the SENP3 C532A mutant, which lacks SENP3 de-SUMOylation activity, were overexpressed. In cells, SENP3 WT overexpression was sufficient to dissociate SUMO2 from IRF4, whereas the SENP3 mutant was not (Fig. 4D). To further investigate the impact of SENP3, *SENP3* was knocked down in HEK293T, IRF4 K349R mutant cannot bind to SUMO2 and *SENP3* knockdown increased SUMO2 modification on IRF4 (Fig. 4E). These data confirm that IRF4 is a substrate of SENP3 and K349 is a SUMO2 modified site. SUMOylation of IRF4 is increased during alternative activation of macrophages due to weakened interaction with SENP3. Lysine 349 of IRF4 is the binding site of SUMO2, and SUMOylation of IRF4 helps improve macrophages' alternative activation ability.

#### IRF4 SUMOylation enhances IRF4 stability and promotes alternative activation in macrophage

**Fig. 5:**
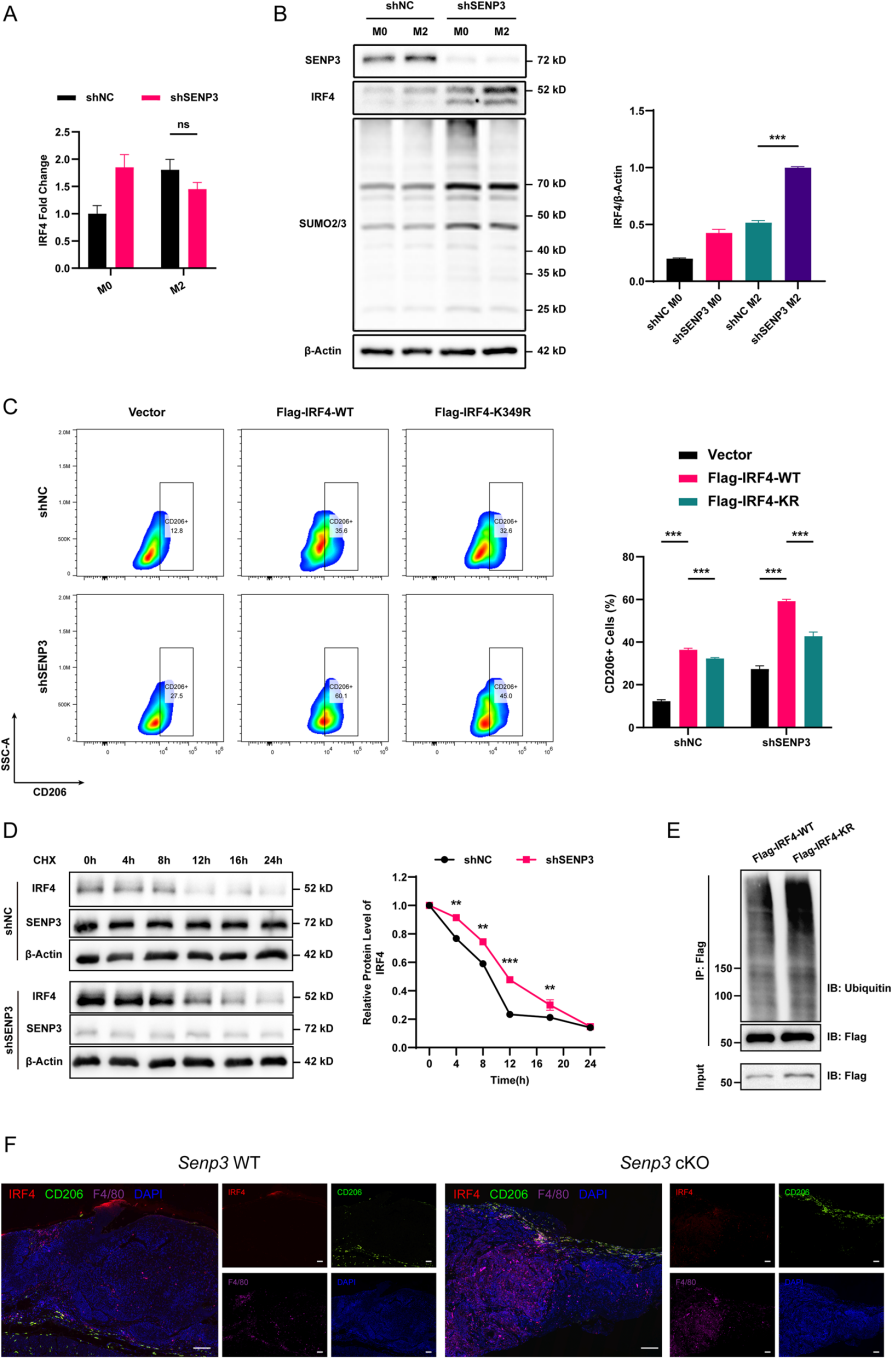
IRF4 SUMOylation enhances IRF4 stability and promotes alternative activation in macrophage. **A** The relative expression of IRF4 in M0 and M2 macrophages in shNC and sh*SENP3* groups. **B** Western blotting detected the levels of IRF4 and SUMO2/3 in shNC and shSENP3 macrophages before and after alternative activation. **C** Flow cytometry analysis of the proportion of CD206+ cells in shNC and sh*SENP3* groups after transfection with Vector, Flag-IRF4-WT, and Flag-IRF4-KR. **D** Cycloheximide was used to hinder protein synthesis. In the shNC and sh*SENP3* groups, the intracellular protein levels of IRF4 were measured at 0, 4, 8, 12, and 16 24 h, respectively. **E** Flag-IRF4-WT and Flag-IRF4-KR proteins were purified using anti-Flag magnetic beads, IRF4 immunoprecipitates were detected using ubiquitin antibodies, and IRF4 ubiquitination was analyzed. **F** Multiple immunofluorescence staining of Irf4, Cd206 and F4/80 was performed after 4-NQO-induced ESCC in *Senp3* WT and *Senp3* cKO mice

To further analyze the mechanism by which SENP3 deletion promotes the alternate activation of macrophages, the effect of SENP3-mediated SUMOylation on IRF4 function was studied. IRF4 can regulate a key transcription factor in M2 macrophage polarization through metabolic reprogramming. Therefore, first, IRF4 expression at the transcriptional level was detected. Interestingly, there is no difference in the transcriptional level of IRF4 in the absence or presence of SENP3 (Fig. 5A). IRF4 is almost undetectable at the protein level in the normal non-activated state, while in the activated state, IRF4 is detectable. The expression of IRF4 protein was markedly increased after IL-4/IL-13 induction, and this upregulation was more obvious in the absence of SENP3 (Fig. 5B). These further illustrate that SENP3 regulates IRF4 at the protein modification level rather than the transcription level. Furthermore, the relationship between SUMOylation modification and alternative activation of IRF4 was explored. Flag-tagged IRF4-WT and IRF4-K349R were transfected into THP-1 cells and then induced by PMA+IL-4/IL-13. Flow cytometry showed that IRF4 can significantly affect the alternative activation of macrophages and the SUMOylation level of IRF4 can significantly increase the proportion of CD206-positive cells. Moreover, the difference in CD206-positive cells proportion between Flag-IRF4-WT and Flag-IRF4-K349R transduced macrophages was reduced (Fig. 5C), confirming that SENP3-mediated SUMOylation can regulate IRF4 activity. Additionally, the effect of SUMOylation on IRF4 stability was observed. Cycloheximide (CHX) was added to inhibit IRF4 translation 24 hours after IL-4/IL-13 stimulation, and then cells were collected at designated time points for Western blot analysis of IRF4 protein levels. Interestingly, after 4 - 12 h exposure to CHX, IRF4-K349R was more easily degraded, and its protein level was much lower compared to the IRF4-WT group (Fig. 5D). To further explain this issue, IRF4 protein was purified by immunoprecipitation, and Flag-IRF4-K349R showed higher levels of the polyubiquitin-bound form of IRF4 compared with WT (Fig. 5E). Subsequently, we performed immunofluorescence staining in *Senp3* cKO and *Senp3* WT mice, and the results suggested that the level of Irf4 in macrophages increased significantly in *Senp3* cKO mice (Fig. 5F). These data indicate that SENP3 mediates the SUMOylation of IRF4 in macrophages, thereby enhancing the stability of IRF4 protein under IL-4 induction.

#### Relatively low SENP3 expression in macrophages is associated with poor prognosis in ESCC patients

**Fig. 6:**
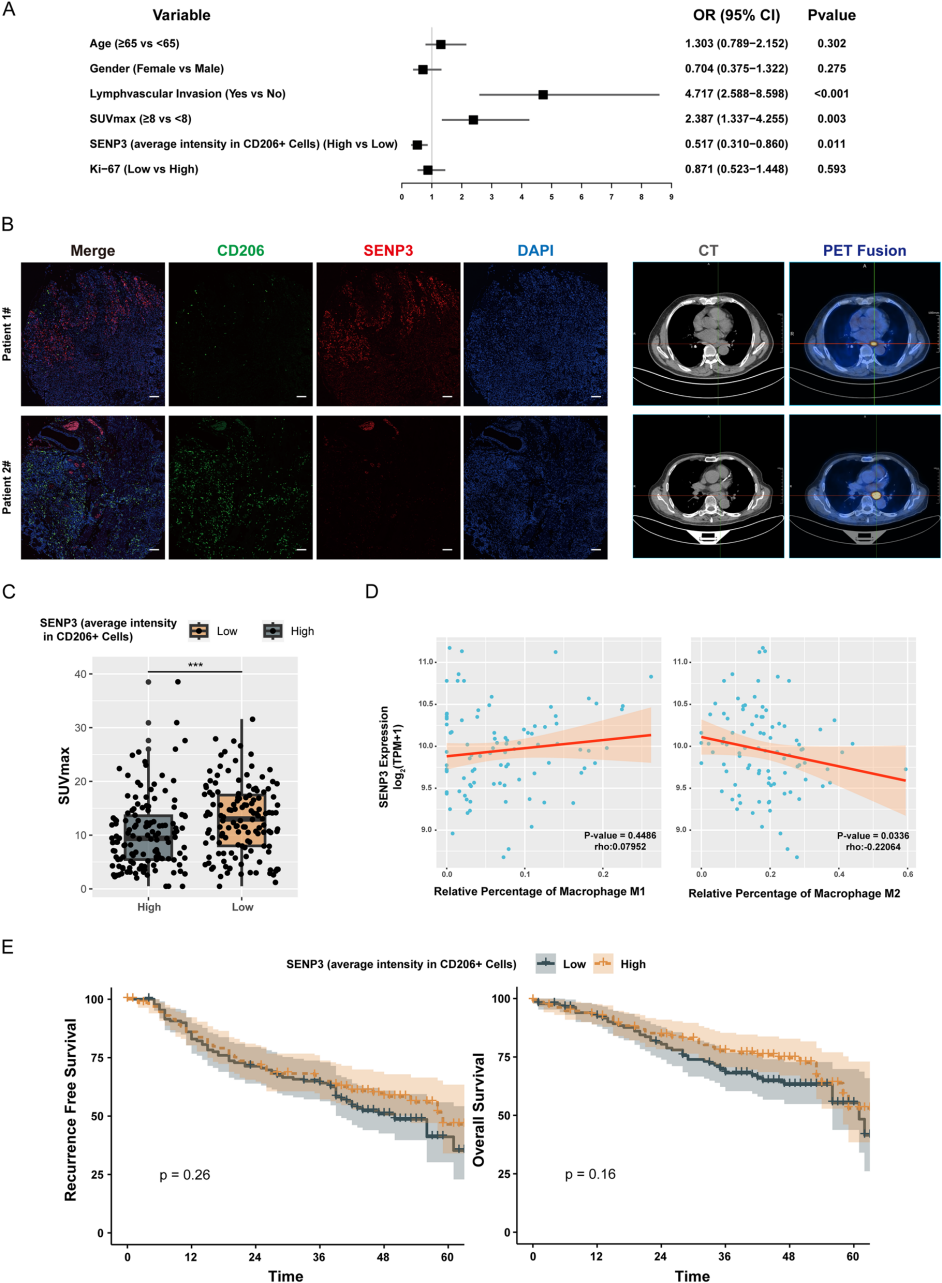
Relatively low SENP3 expression in macrophages is associated with poor prognosis in ESCC patients**. A** Univariate regression analysis assessed the risk factors related to ESCC lymph node metastasis, including age, gender, lymphatic vessel invasion, primary lesion SUVmax, macrophage SENP3 relative expression, and primary lesion Ki-67 level. **B** Multiplex immunofluorescence staining was used to co-stain SENP3 and CD206 and revealed the relative expression of SENP3 in ESCC-related macrophages, which was then compared with the patient's preoperative PET-CT. **C** Box plot of relative expression of SENP3 in macrophages and SUVmax of primary lesions. **D** Patients with squamous cell carcinoma in the TCGA-ESCA database were identified. The ratio of M1 and M2 macrophages in the patients was calculated using CibersortX, and the correlation between the expression of SENP3 and the ratio of M1/M2 cells in the patients was analyzed. **E** Kaplan-Meier curve analysis of patients with high and low relative SENP3 expression levels in macrophages and recurrence-free and overall survival

Finally, SENP3 levels in macrophages and clinicopathological data of ESCC patients were analyzed. The intensities of SENP3, CD206, and SENP3 in CD206-positive cells in patient tissue sections were assessed through multiple immunofluorescence staining. Univariate regression analysis revealed that lymphovascular invasion (*p < 0.001*), primary lesion SUVmax ≥ 8 (*p = 0.003*), and relatively low expression of SENP3 in CD206-positive cells (*p = 0.011*) were significantly associated with lymph node metastasis in ESCC patients (Fig. 6A). The patients' pathological sections were compared with preoperative PET-CT and one patient with high and one with low relative expression of SENP3 in CD206-positive cells were selected, and their preoperative PET-CT was compared. It was observed that patients with relatively low SENP3 intensity in CD206 positive cell seem to have higher primary tumor SUVmax (Fig. 6B). Furthermore, the SUVmax of all patients were recorded, and then these patients were categorized into high and low groups based on the intensity of SENP3 in CD206-positive cells. Patients with low SENP3 expression in CD206-positive cells had higher SUVmax in their primary lesions (*p <0.001*) (Fig. 6C). Furthermore, ESCC patients in the TCGA-ESCA database were selected and calculated through CibersortX (https://cibersortx.stanford.edu/) [17]. The results showed that patients with low SENP3 expression had a higher proportion of M2 macrophages (r = -0.22, *p = 0.0336*) (Fig. 6D). Lastly, survival analysis was performed for the two groups of patients, which indicated no significant statistical difference in recurrence-free and overall survival (Fig. 6E).

## Discussion

ESCC is one of the most common tumors in China and ranks 6^th^ in morbidity and mortality among all tumors. It is one of the malignant tumors that threatens the health of the Chinese people and causes a huge medical and economic burden. This study revealed that SENP3 in macrophages is significantly up-regulated in ESCC tissue, and the relatively low SENP3 expression is related to a poor ESCC prognosis, such as lymph node metastasis or increased SUVmax of the primary lesion. The *in vivo* and *in vitro* experiments revealed that the replacement of shSENP3 activated macrophage conditioned medium can promote ESCC proliferation and migration and exhibit faster tumor proliferation and progression at the animal level. Comprehensive analysis revealed that SENP3 interacts with IRF4 and affects the SUMO2/3 level of IRF4. Loss of SENP3 increases IRF4 SUMOylation, thereby increasing the level of alternative activation of macrophages and secreting more immunosuppressive cytokines.

Macrophage polarization refers to the differentiation of macrophages upon stimulation into distinct types of cells [18], including M1 (classic activation) and M2 (alternative activation) [19]. M1 macrophages are pro-inflammatory macrophages that clear pathogens and produce inflammatory mediators such as IL-1β, IL-6, and TNF-α during infection and inflammatory responses. Polarization of M1 macrophages is usually induced by LPS and cytokines (such as IFN-γ) [20]. M2 macrophages are anti-inflammatory cells with the key functions of wound healing and tissue repair [21]. Polarization of M2 macrophages is stimulated by cytokines (such as IL-4, IL-10, and IL-13) and parasite infection [22]. The type and degree of macrophage polarization are affected by multiple environmental factors, including cytokines released within the microenvironment and metabolic homeostasis [23].

This study found that IRF4 levels gradually increased with the M0-M2 process during macrophage polarization, and the IRF4 protein level after sh*SENP3* was also significantly higher than that in the shNC group. This study is the first to discover and report the interaction between SENP3 and IRF4 and its regulation of the SUMOylation modification of IRF4. Furthermore, it validated that SENP3 can regulate the SUMOylation of its K349 site and promote the M2 polarization process. We fully validated this process in the 293T cell line, including KR mutation and shRNA knockdown of endogenous *SENP3*. Moreover, THP-1 was used to transduce Flag-IRF4-WT and Flag-IRF4-KR and found through flow cytometry that these were indispensable for macrophage polarization. In shSENP3, since Flag-IRF4-KR cannot undergo SUMO modification, the de-SUMO modification is inhibited, therefore, Flag-IRF4-WT can undergo SUMO modification and promote macrophage M2 polarization.

Interferon regulatory factors (IRFs) play different functions in the transcriptional regulation of the immune system. IRF4 can regulate the differentiation of Th2, Th9, Th17, Tfh, Treg, and cytotoxic effector CD8+ T cells [24, 25]. It can promote dendritic cells to secrete IL-23 and increase Th17 cell response [26]. Studies have shown that IRF4 regulates the function of TAM and is involved in tumor progression and metastasis [16, 27]. SUMOylation modification is also important for the homeostasis of IRF4 protein. Tatham *et al.* found that IRF4 is modified by SUMO-2, which prevents IRF4 degradation through the ubiquitin/proteasome pathway, indicating that SUMO modification can also target proteins for degradation, rendering them unable to bind IRF4 [28]. This study has certain limitations. 1) This study determined that SENP3 mediates the SUMOylation of IRF4 to exert its function but lacks an in-depth and detailed analysis of downstream IRF4. 2) SENP3 may also promote M2 polarization of macrophages by affecting other alternative activation substrates which need further assessments. 

## Conclusion

In summary, this study provided evidence that SENP3 binds IRF4 and affects the alternative activation of ESCC-related macrophages by affecting the SUMOylation modification of IRF4.

### Supplementary Information

Supplementary Material 1.

Supplementary Material 2.

Supplementary Material 3.

Supplementary Material 4.

Supplementary Material 5.

## Data Availability

No datasets were generated or analysed during the current study.

## Abbreviations

4-NQO: 4-nitroquinoline-1-oxide
7-AAD: 7-Amino-Actinomycin D
AMP: Ampicillin
BMDM: bone marrow-derived macrophage
CCK-8: Cell Counting Kit-8
Co-IP: Co-immunoprecipitation
DEPC: Diethylpyrocarbonate
DMEM: Dulbecco's Modified Eagle Medium
DMSO: Dimethyl sulfoxide
ELISA: Enzyme-linked immunosorbent assay
ESCC: Esophageal squamous cell carcinoma
FBS: Fetal Bovine Serum
H&E: Hematoxylin-eosin
IF: Immunofluorescence
IFN-γ: Interferon-γ
IHC: Immunohistochemistry
IL-13: Interleukin 13
IL-4: Interleukin 4
IP: Immunoprecipitation
IRF4: Interferon Regulatory Factor 4
LNM: lymph node metastasis
LPS: Lipopolysaccharide
NEM: N-Ethylmaleimide
PBS: Phosphate buffer saline
PMA: Phorbol 12-myristate 13-acetate
qRT-PCR: Quantitative real-time polymerase chain reaction
rpm: Revolutions Per Minute
SENP3: Sentrin/SUMO specific protease 3
shRNA: short hairpin RNA
SUMO: Small ubiquitin-like modifier
TAE Buffer: Tris Acetate-EDTA buffer
TAM: Tumor-associated macrophage
TCGA: The Cancer Genome Atlas
TGF-β: Transforming growth factor beta
TMA: Tissue microarray
WB: Western blotting

## References

[1] Chen W, Zheng R, Baade PD, Zhang S, Zeng H, Bray F, et al. Cancer statistics in China, 2015. CA Cancer J Clin. 2016;66(2):115–32. 26808342 10.3322/caac.21338

[2] Zheng R, Zhang S, Zeng H, Wang S, Sun K, Chen R, et al. Cancer incidence and mortality in China, 2016. J Nat Cancer Center. 2022;2:1–9. 10.1016/j.jncc.2022.02.002 PMC11256658 39035212

[3] Chen J, Zhao D, Zhang L, Zhang J, Xiao Y, Wu Q, et al. Tumor-associated macrophage (TAM)-derived CCL22 induces FAK addiction in esophageal squamous cell carcinoma (ESCC). Cell Mol Immunol. 2022;19(9):1054–66. 35962191 10.1038/s41423-022-00903-z PMC9424285

[4] Yang H, Zhang Q, Xu M, Wang L, Chen X, Feng Y, et al. CCL2-CCR2 axis recruits tumor associated macrophages to induce immune evasion through PD-1 signaling in esophageal carcinogenesis. Mol Cancer. 2020;19(1):41. 32103760 10.1186/s12943-020-01165-x PMC7045401

[5] Liu J, Li C, Zhang L, Liu K, Jiang X, Wang X, et al. Association of tumour-associated macrophages with cancer cell EMT, invasion, and metastasis of Kazakh oesophageal squamous cell cancer. Diagn Pathol. 2019;14(1):55. 31186031 10.1186/s13000-019-0834-0 PMC6560903

[6] Shigeoka M, Urakawa N, Nakamura T, Nishio M, Watajima T, Kuroda D, et al. Tumor associated macrophage expressing CD204 is associated with tumor aggressiveness of esophageal squamous cell carcinoma. Cancer Sci. 2013;104(8):1112–9. 23648122 10.1111/cas.12188 PMC7657117

[7] Chen Y, Feng R, He B, Wang J, Xian N, Huang G, et al. PD-1H Expression Associated With CD68 Macrophage Marker Confers an Immune-Activated Microenvironment and Favorable Overall Survival in Human Esophageal Squamous Cell Carcinoma. Front Mol Biosci. 2021;8:777370. 34950702 10.3389/fmolb.2021.777370 PMC8688962

[8] Lu Y, Guo L, Ding G. PD1+ tumor associated macrophages predict poor prognosis of locally advanced esophageal squamous cell carcinoma. Future Oncol. 2019;15(35):4019–30. 31612729 10.2217/fon-2019-0519

[9] Mai S, Liu L, Jiang J, Ren P, Diao D, Wang H, et al. Oesophageal squamous cell carcinoma-associated IL-33 rewires macrophage polarization towards M2 via activating ornithine decarboxylase. Cell Prolif. 2021;54(2):e12960. 33305406 10.1111/cpr.12960 PMC7848962

[10] Vertegaal ACO. Signaling mechanisms and cellular functions of SUMO. Nat Rev Mol Cell Biol. 2022;23(11):715–31. 35750927 10.1038/s41580-022-00500-y

[11] Yeh ETH. SUMOylation and De-SUMOylation: wrestling with life’s processes. J Biol Chem. 2009;284(13):8223–7. 19008217 10.1074/jbc.R800050200 PMC2659178

[12] Wang M, Sang J, Ren Y, Liu K, Liu X, Zhang J, et al. SENP3 regulates the global protein turnover and the Sp1 level via antagonizing SUMO2/3-targeted ubiquitination and degradation. Protein Cell. 2016;7(1):63–77. 26511642 10.1007/s13238-015-0216-7 PMC4707158

[13] Huang J, Jiang D, Zhu T, Wang Y, Wang H, Wang Q, et al. Prognostic Significance of c-MYC Amplification in Esophageal Squamous Cell Carcinoma. Ann Thorac Surg. 2019;107(2):436–43. 30273571 10.1016/j.athoracsur.2018.07.077

[14] Zhang Y, Yang K, Yang J, Lao Y, Deng L, Deng G, et al. SENP3 Suppresses Osteoclastogenesis by De-conjugating SUMO2/3 from IRF8 in Bone Marrow-Derived Monocytes. Cell Rep. 2020;30(6):1951-1963.e4. 32049023 10.1016/j.celrep.2020.01.036

[15] Hendriks IA, Lyon D, Su D, Skotte NH, Daniel JA, Jensen LJ, et al. Site-specific characterization of endogenous SUMOylation across species and organs. Nat Commun. 2018;9(1):2456. 29942033 10.1038/s41467-018-04957-4 PMC6018634

[16] Wang F, Sun F, Luo J, Yue T, Chen L, Zhou H, et al. Loss of ubiquitin-conjugating enzyme E2 (Ubc9) in macrophages exacerbates multiple low-dose streptozotocin-induced diabetes by attenuating M2 macrophage polarization. Cell Death & Disease. 2019;10(12):892. 31767832 10.1038/s41419-019-2130-z PMC6877645

[17] Newman AM, Steen CB, Liu CL, Gentles AJ, Chaudhuri AA, Scherer F, et al. Determining cell type abundance and expression from bulk tissues with digital cytometry. Nat Biotechnol. 2019;37(7):773–82. 31061481 10.1038/s41587-019-0114-2 PMC6610714

[18] Immunometabolism Minton K. Stress-induced macrophage polarization. Nat Rev Immunol. 2017;17(5):277. 28393924 10.1038/nri.2017.41

[19] Xue J, Schmidt SV, Sander J, Draffehn A, Krebs W, Quester I, et al. Transcriptome-based network analysis reveals a spectrum model of human macrophage activation. Immunity. 2014;40(2):274–88. 24530056 10.1016/j.immuni.2014.01.006 PMC3991396

[20] Orecchioni M, Ghosheh Y, Pramod AB, Ley K. Macrophage Polarization: Different Gene Signatures in M1(LPS+) vs. Classically and M2(LPS-) vs. Alternatively Activated Macrophages. Front Immunol. 2019;10:1084. 31178859 10.3389/fimmu.2019.01084 PMC6543837

[21] Mantovani A, Sozzani S, Locati M, Allavena P, Sica A. Macrophage polarization: tumor-associated macrophages as a paradigm for polarized M2 mononuclear phagocytes. Trends Immunol. 2002;23(11):549–55. 12401408 10.1016/S1471-4906(02)02302-5

[22] Mantovani A, Sica A, Locati M. Macrophage polarization comes of age. Immunity. 2005;23(4):344–6. 16226499 10.1016/j.immuni.2005.10.001

[23] Liu Y-C, Zou X-B, Chai Y-F, Yao Y-M. Macrophage polarization in inflammatory diseases. Int J Biol Sci. 2014;10(5):520–9. 24910531 10.7150/ijbs.8879 PMC4046879

[24] Brüstle A, Heink S, Huber M, Rosenplänter C, Stadelmann C, Yu P, et al. The development of inflammatory T(H)-17 cells requires interferon-regulatory factor 4. Nat Immunol. 2007;8(9):958–66. 17676043 10.1038/ni1500

[25] Bollig N, Brüstle A, Kellner K, Ackermann W, Abass E, Raifer H, et al. Transcription factor IRF4 determines germinal center formation through follicular T-helper cell differentiation. Proc Natl Acad Sci U S A. 2012;109(22):8664–9. 22552227 10.1073/pnas.1205834109 PMC3365194

[26] Schlitzer A, McGovern N, Teo P, Zelante T, Atarashi K, Low D, et al. IRF4 transcription factor-dependent CD11b+ dendritic cells in human and mouse control mucosal IL-17 cytokine responses. Immunity. 2013;38(5):970–83. 23706669 10.1016/j.immuni.2013.04.011 PMC3666057

[27] Huang SCC, Smith AM, Everts B, Colonna M, Pearce EL, Schilling JD, et al. Metabolic Reprogramming Mediated by the mTORC2-IRF4 Signaling Axis Is Essential for Macrophage Alternative Activation. Immunity. 2016;45(4):817–30. 27760338 10.1016/j.immuni.2016.09.016 PMC5535820

[28] Tatham MH, Geoffroy M-C, Shen L, Plechanovova A, Hattersley N, Jaffray EG, et al. RNF4 is a poly-SUMO-specific E3 ubiquitin ligase required for arsenic-induced PML degradation. Nat Cell Biol. 2008;10(5):538–46. 18408734 10.1038/ncb1716

