# Supplementary figures and images for: Suppression of SENP3 enhances macrophage alternative activation by mediating IRF4 de-SUMOylation in ESCC progression (part 1 of 2)
Source: Cell Commun Signal. 2024 Aug 9;22:395. doi: 10.1186/s12964-024-01770-z (PMC11312810; doi:10.1186/s12964-024-01770-z)

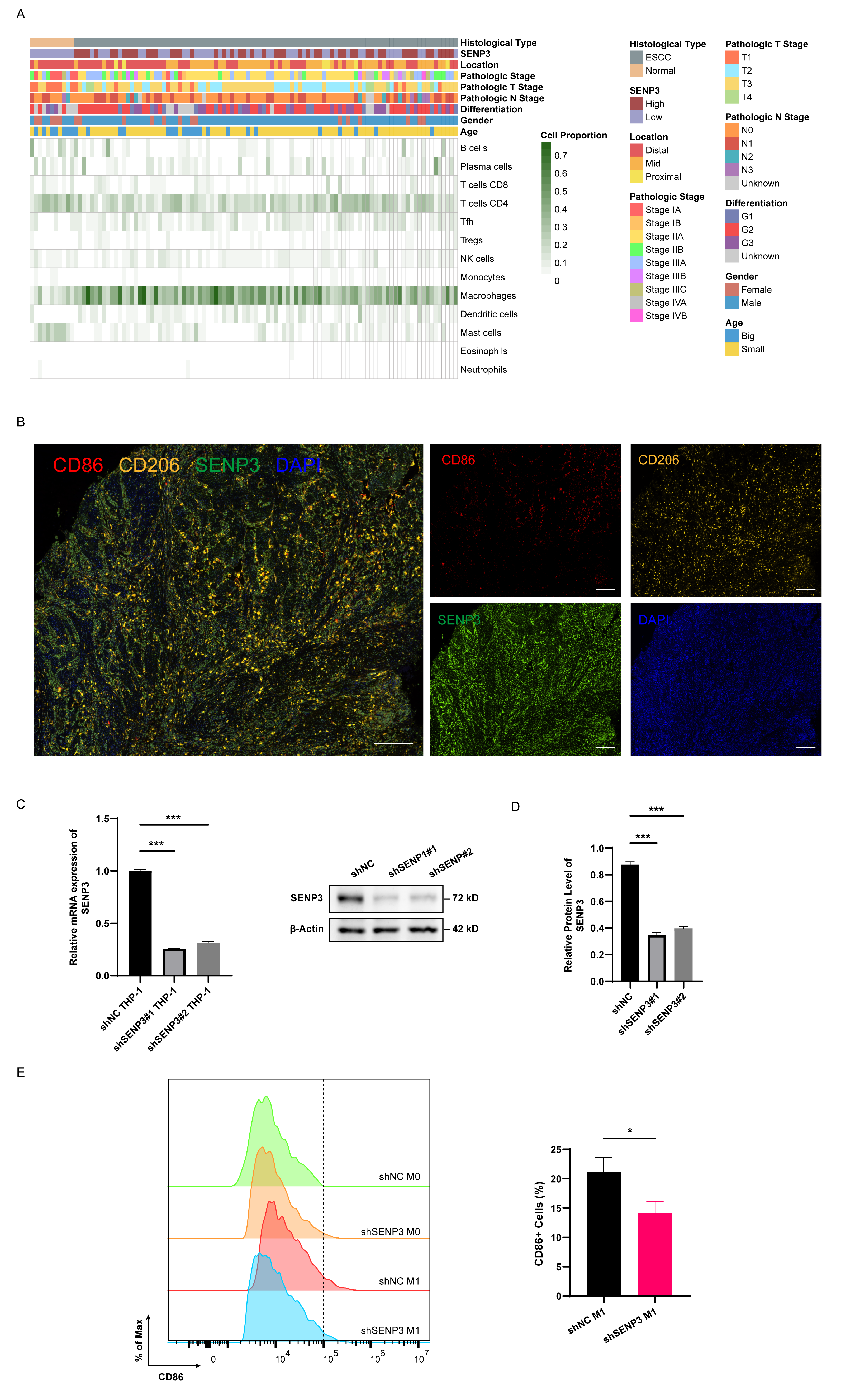

Supplement: Supplementary file 1 — Supplementary Material 1. [file 12964_2024_1770_MOESM1_ESM.tif]

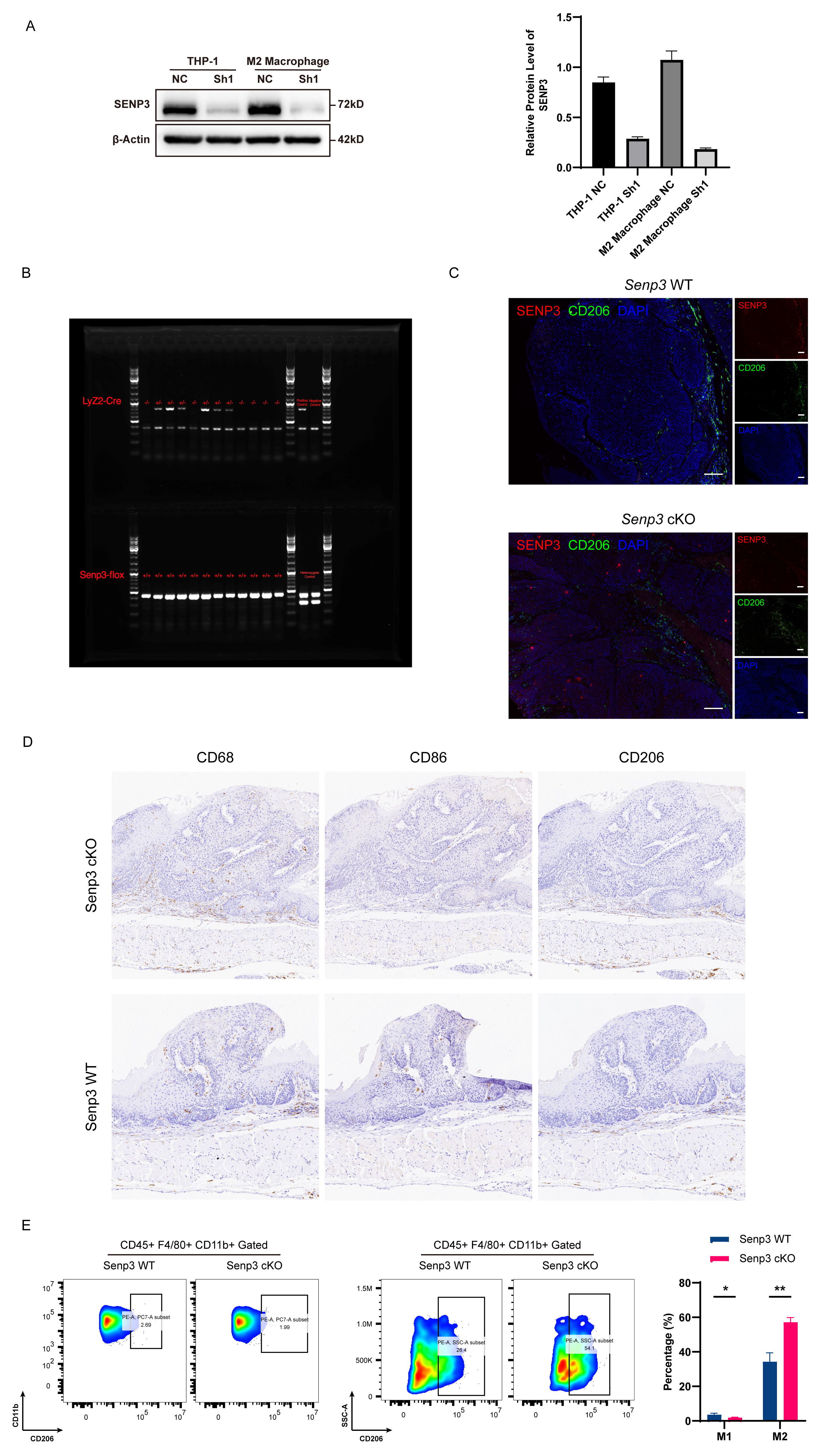

Supplement: Supplementary file 2 — Supplementary Material 2. [file 12964_2024_1770_MOESM2_ESM.tif]

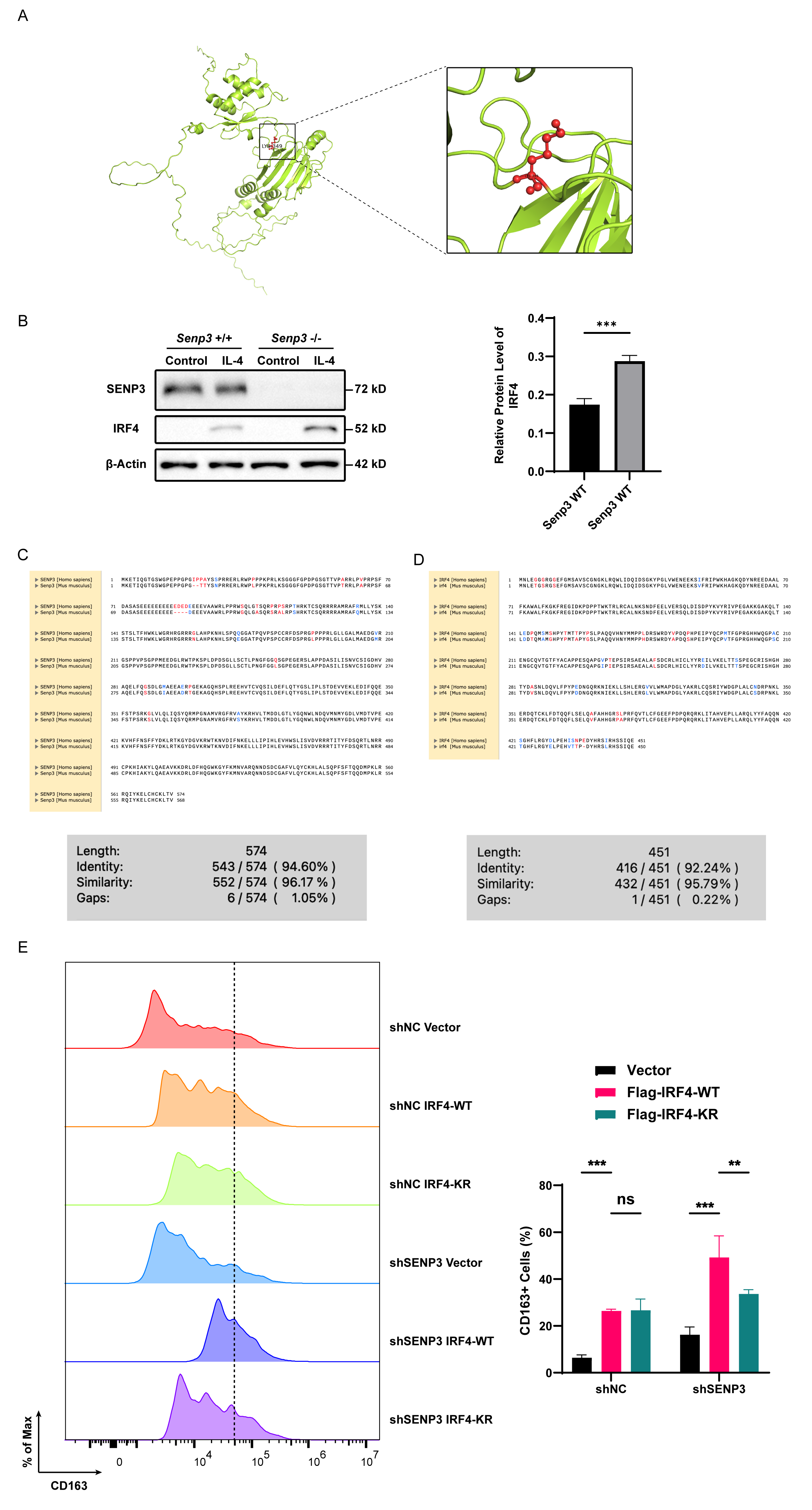

Supplement: Supplementary file 3 — Supplementary Material 3. [file 12964_2024_1770_MOESM3_ESM.tif]

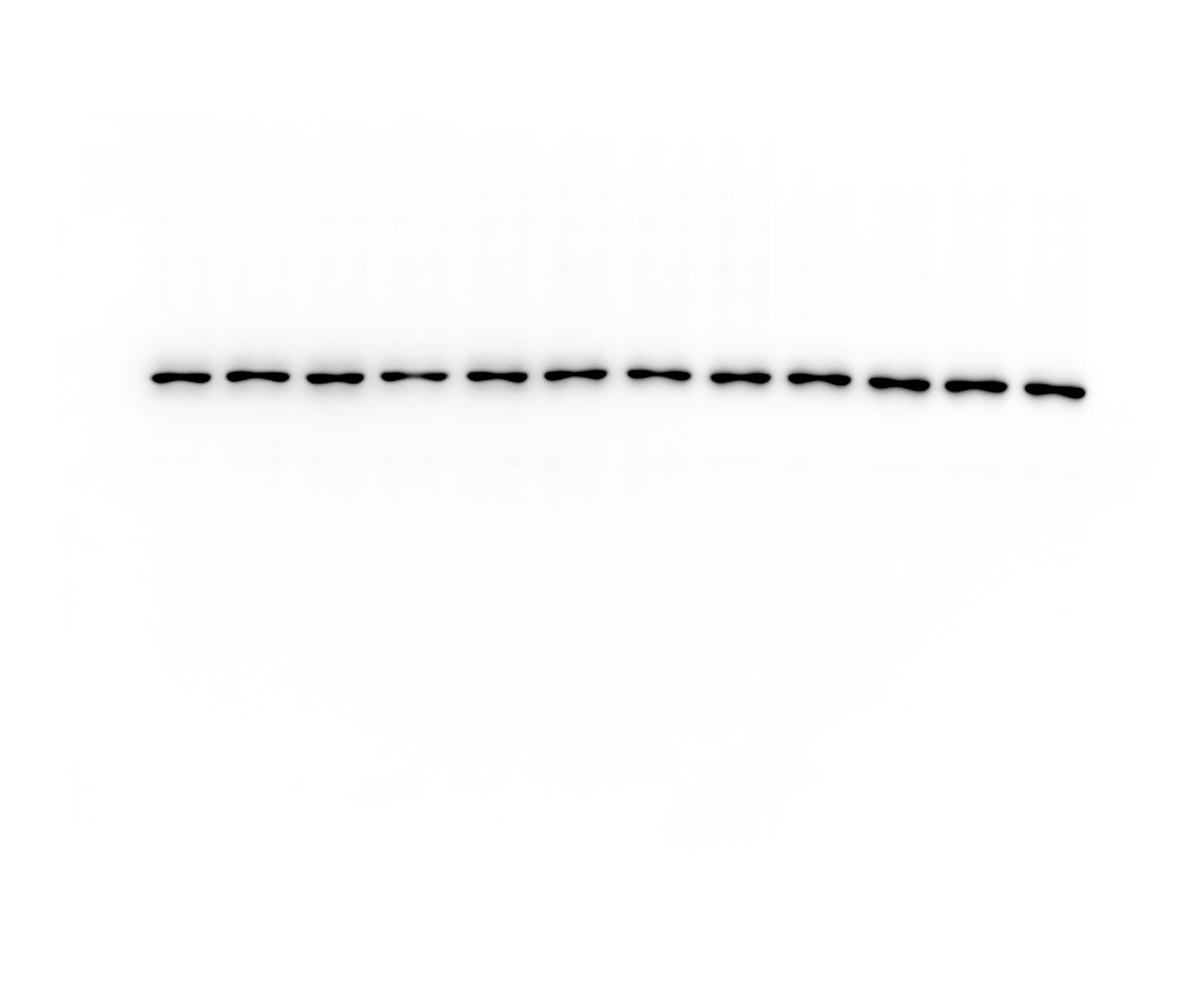

Supplement: Supplementary file 4 — Supplementary Material 4. [file 12964_2024_1770_MOESM4_ESM.zip › SENP3 TAM WB/WB-Figure1/Fig1B/ACTB/5.Tif]

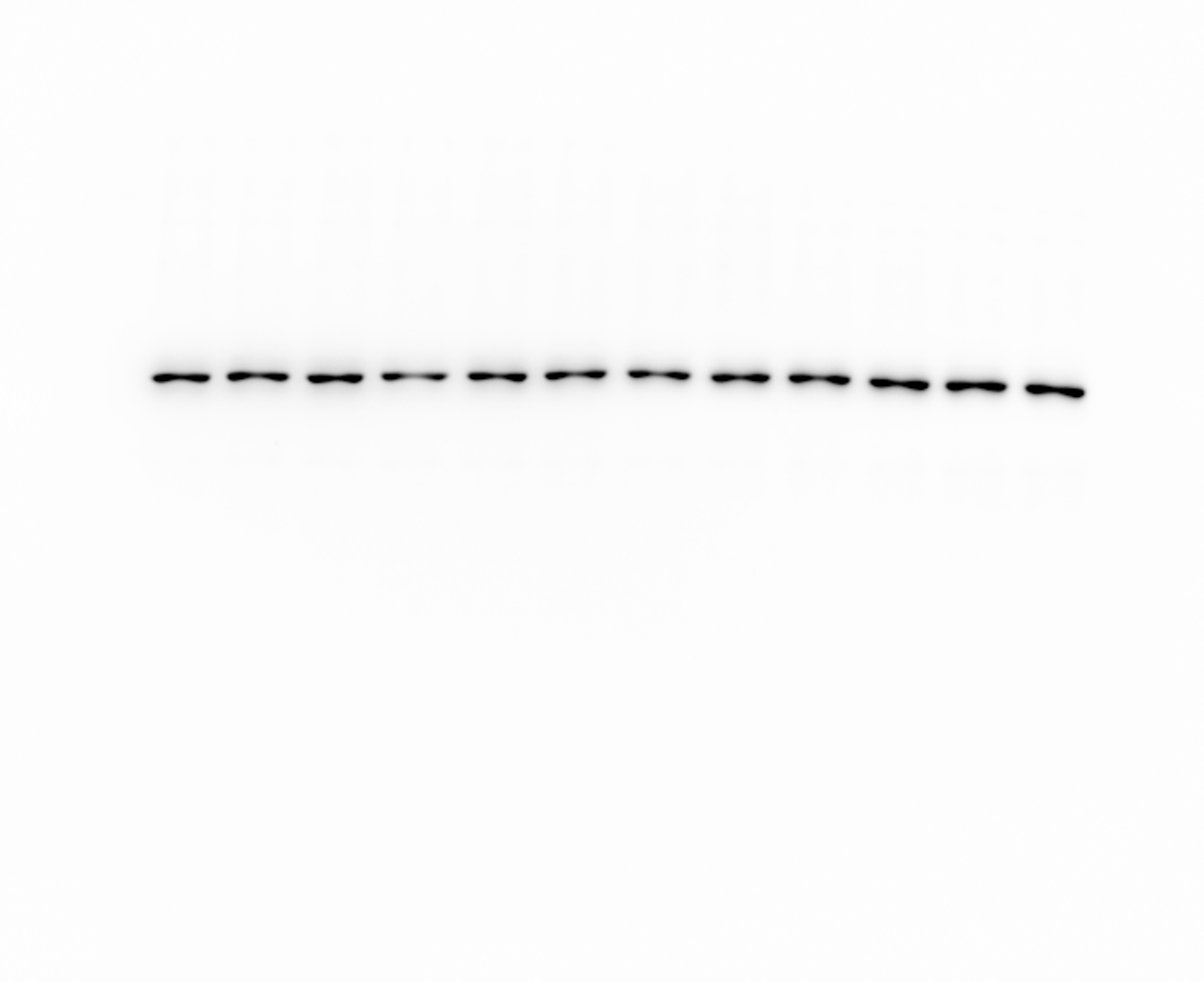

Supplement: Supplementary file 4 — Supplementary Material 4. [file 12964_2024_1770_MOESM4_ESM.zip › SENP3 TAM WB/WB-Figure1/Fig1B/ACTB/5-16.Tif]

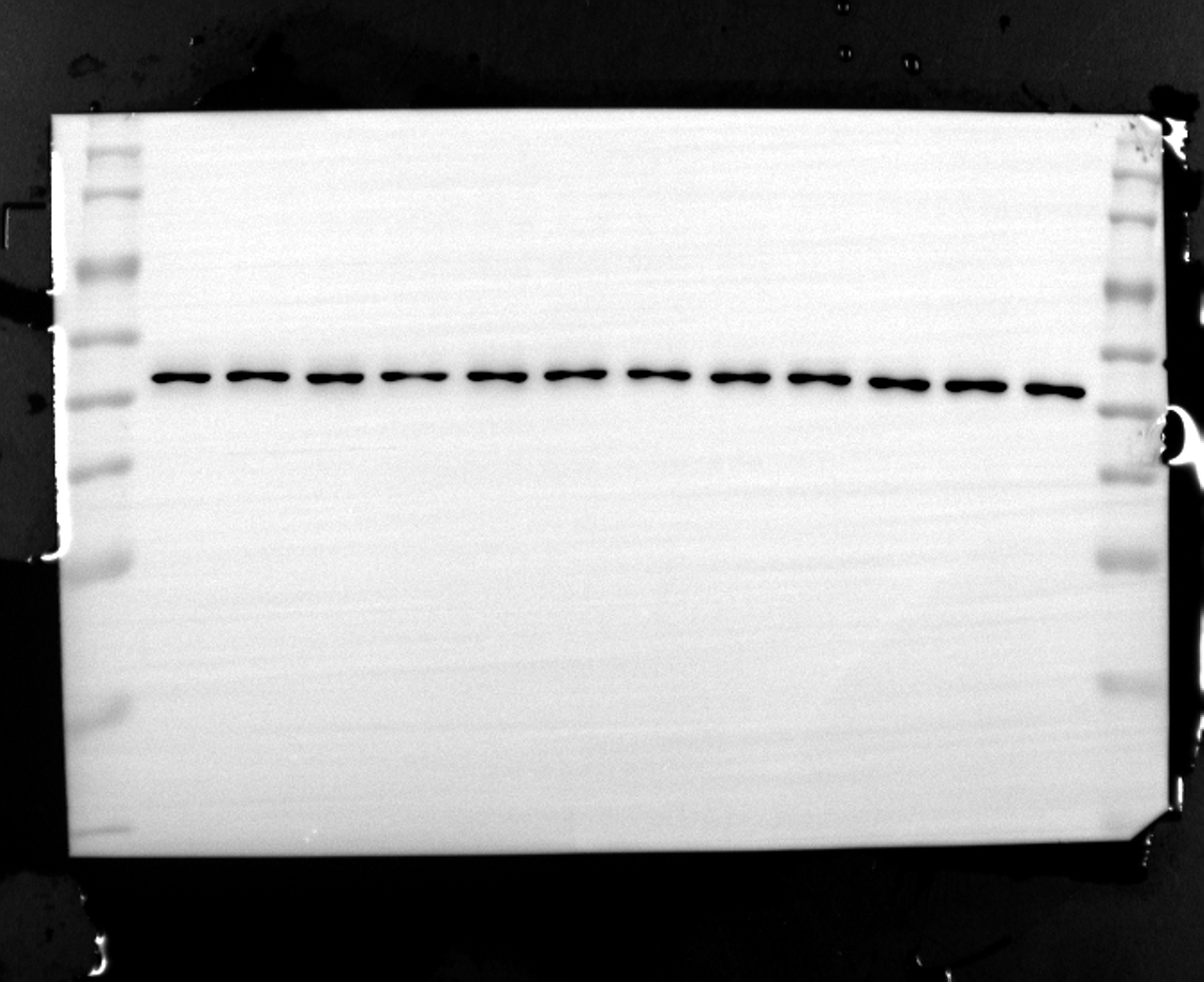

Supplement: Supplementary file 4 — Supplementary Material 4. [file 12964_2024_1770_MOESM4_ESM.zip › SENP3 TAM WB/WB-Figure1/Fig1B/ACTB/M.Tif]

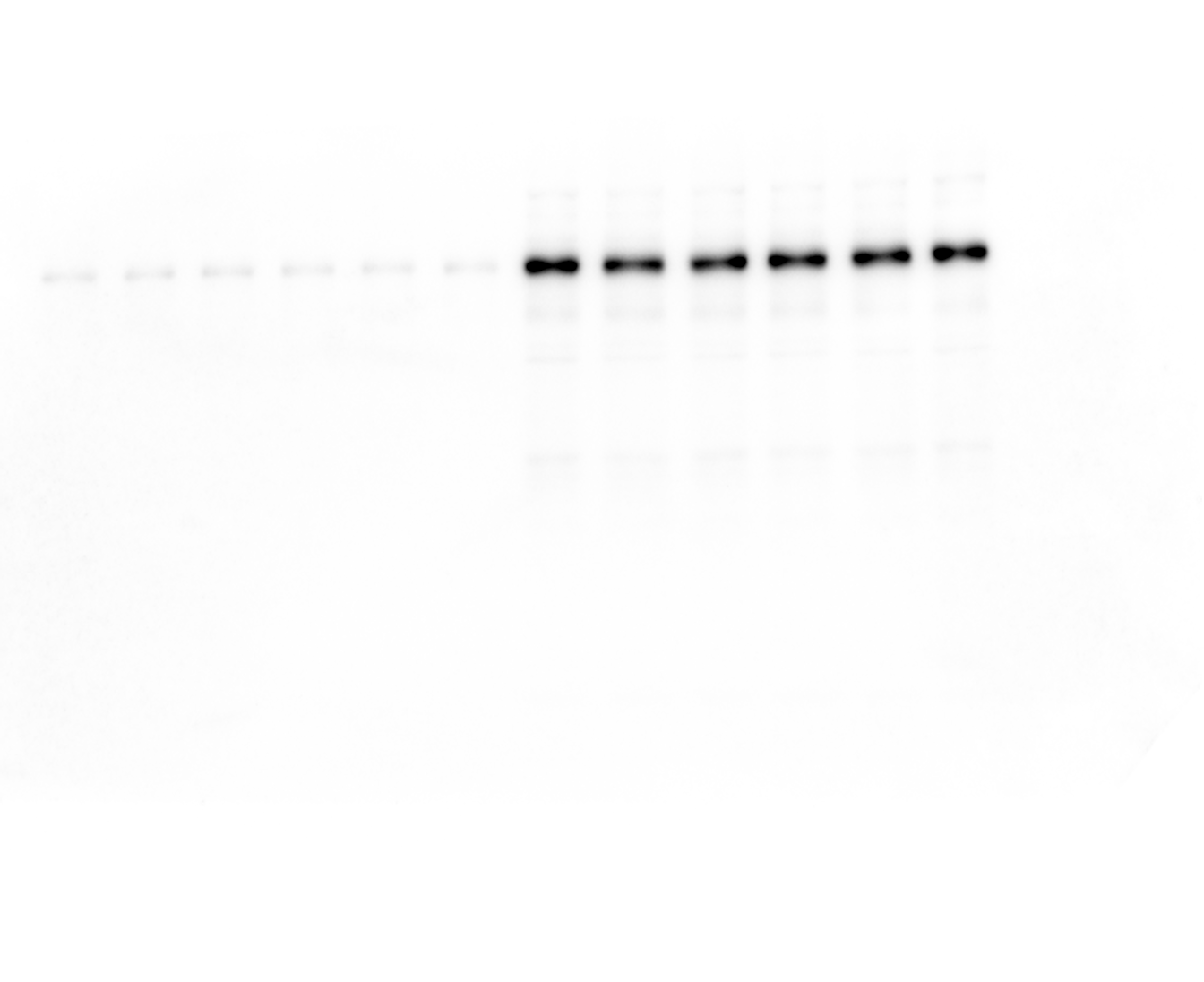

Supplement: Supplementary file 4 — Supplementary Material 4. [file 12964_2024_1770_MOESM4_ESM.zip › SENP3 TAM WB/WB-Figure1/Fig1B/SENP3/4.Tif]

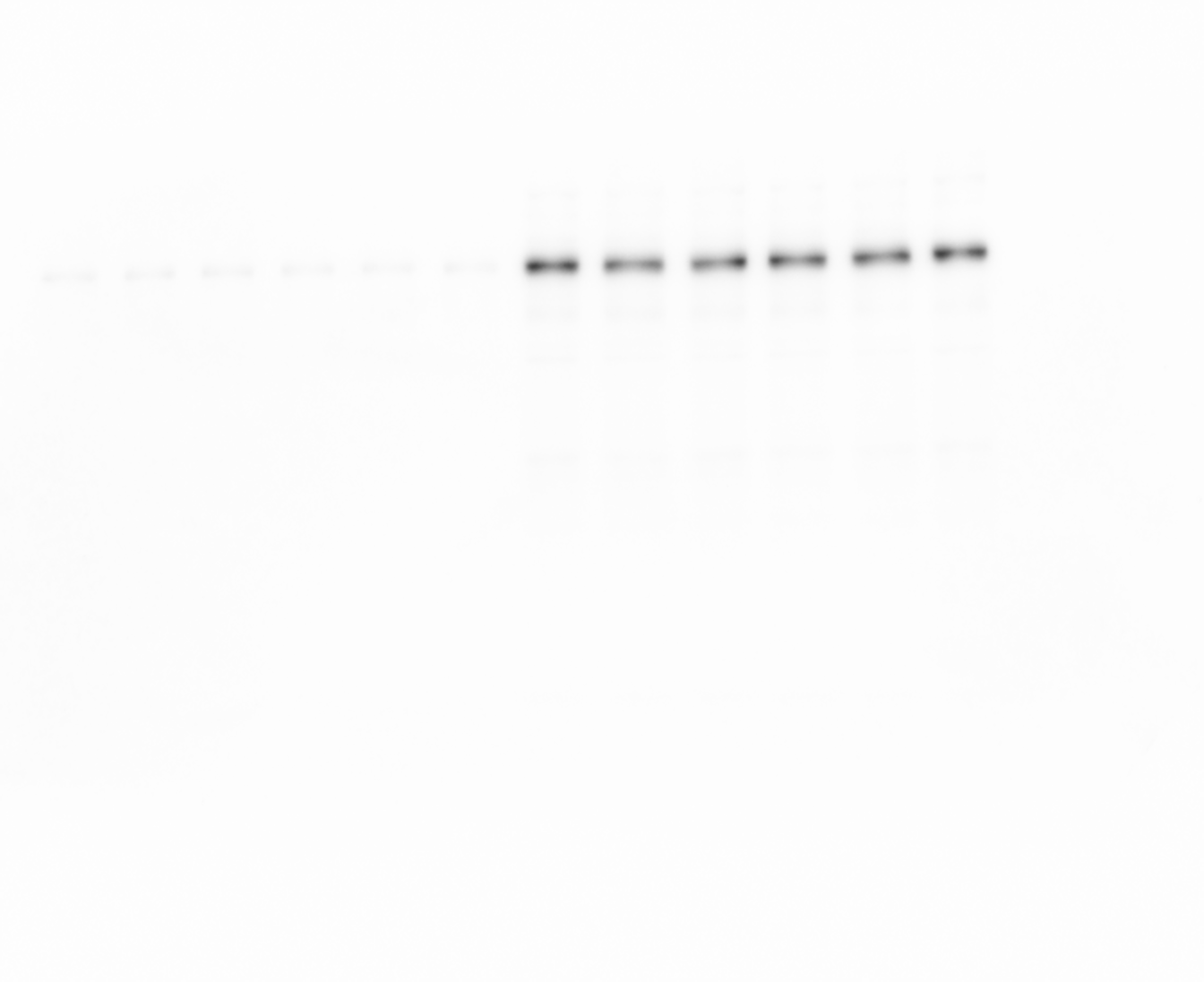

Supplement: Supplementary file 4 — Supplementary Material 4. [file 12964_2024_1770_MOESM4_ESM.zip › SENP3 TAM WB/WB-Figure1/Fig1B/SENP3/4-16.Tif]

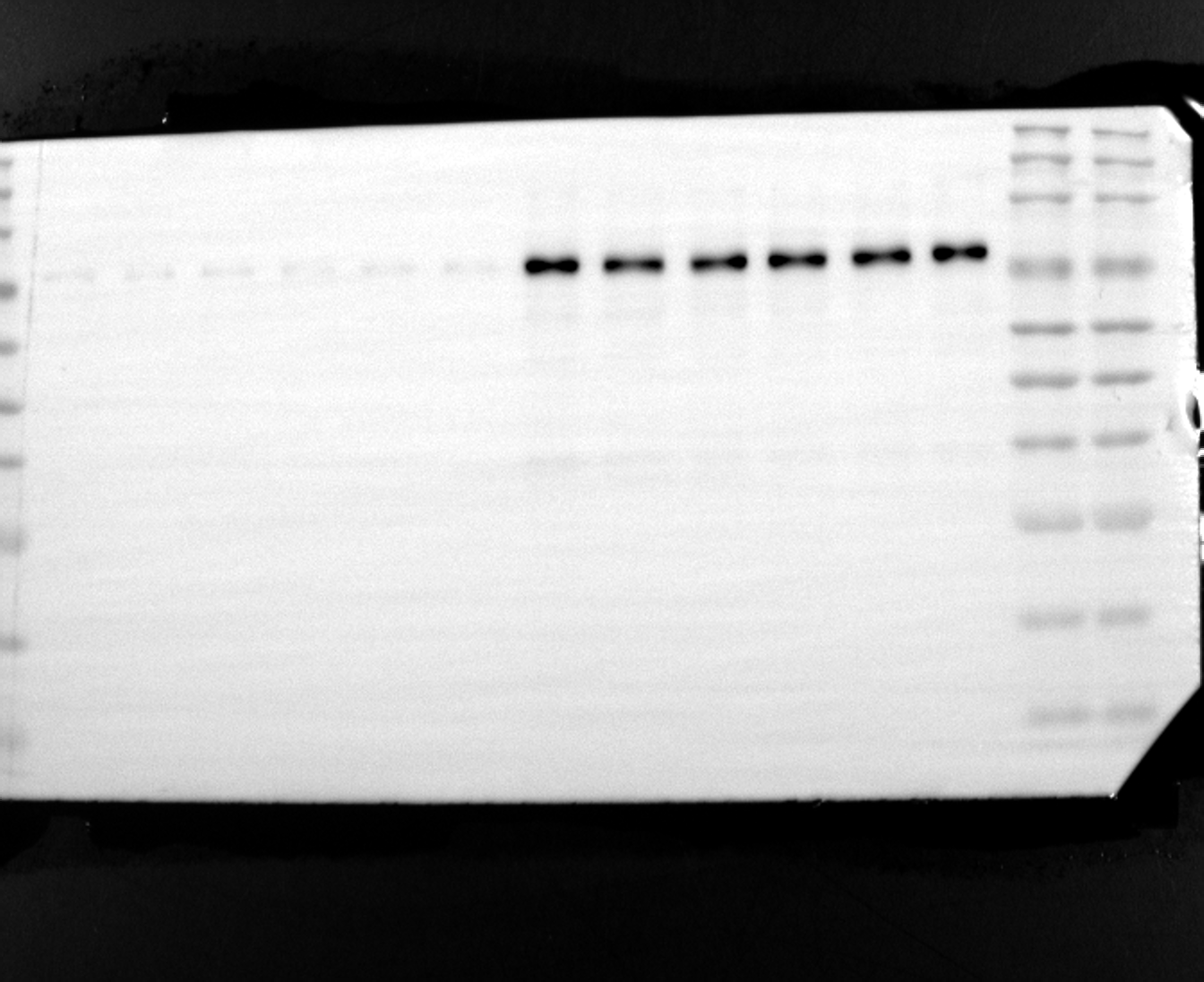

Supplement: Supplementary file 4 — Supplementary Material 4. [file 12964_2024_1770_MOESM4_ESM.zip › SENP3 TAM WB/WB-Figure1/Fig1B/SENP3/M.Tif]

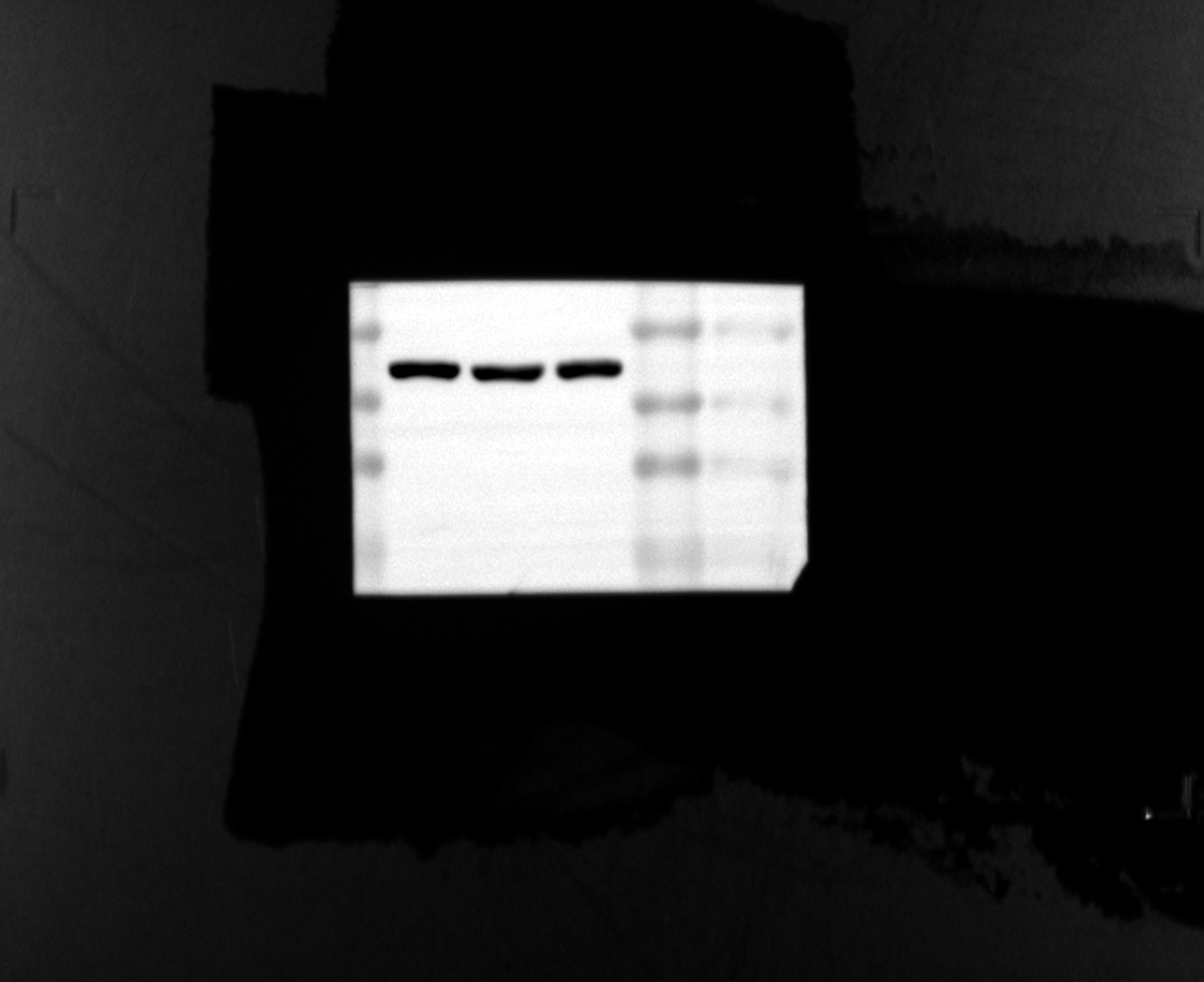

Supplement: Supplementary file 4 — Supplementary Material 4. [file 12964_2024_1770_MOESM4_ESM.zip › SENP3 TAM WB/WB-Figure4/A IRF4 SENP3 co-IP/2023-01-18 IRF4 SENP3 CO-IP/INPUT ACT M.Tif]

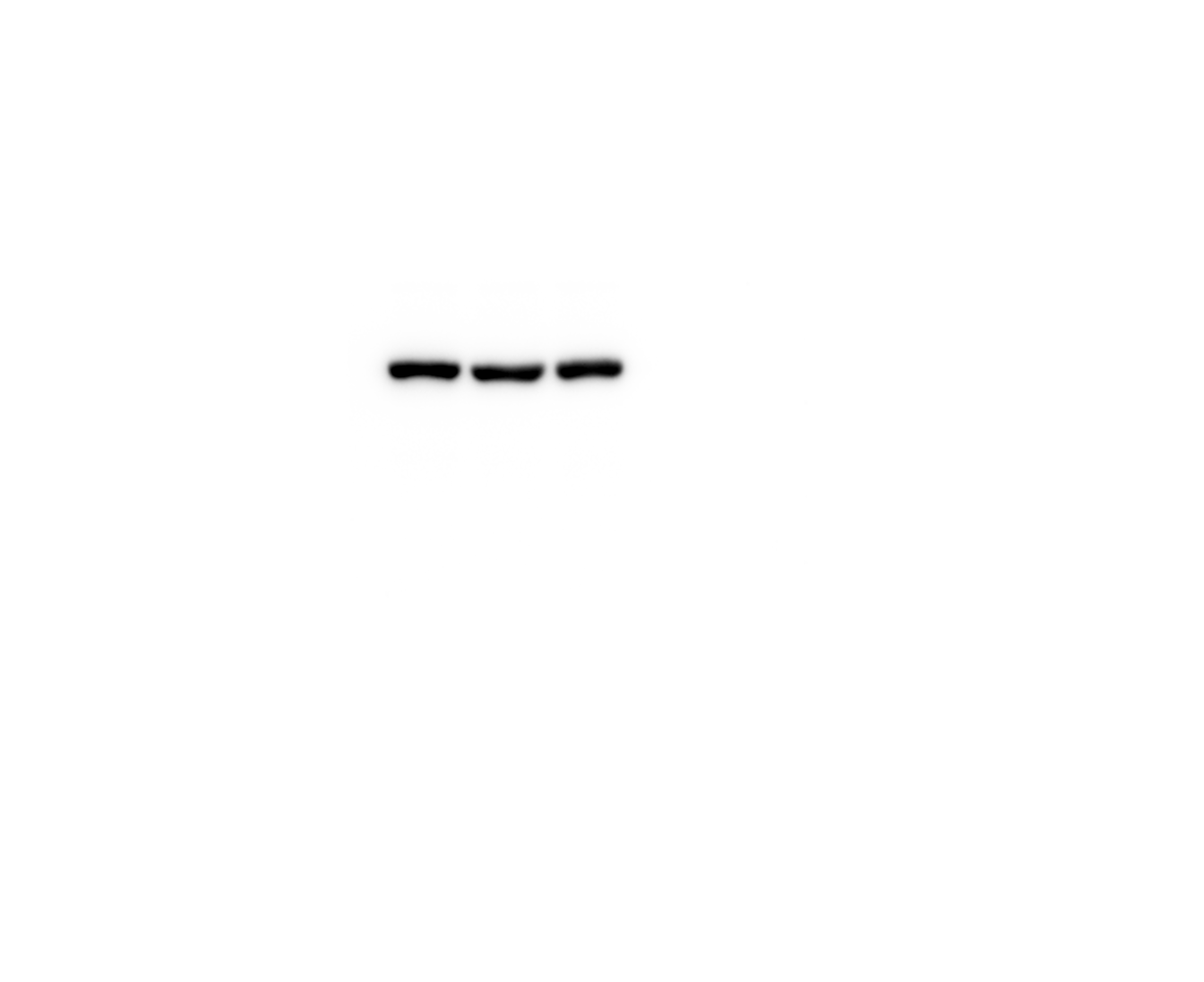

Supplement: Supplementary file 4 — Supplementary Material 4. [file 12964_2024_1770_MOESM4_ESM.zip › SENP3 TAM WB/WB-Figure4/A IRF4 SENP3 co-IP/2023-01-18 IRF4 SENP3 CO-IP/INPUT ACT.Tif]

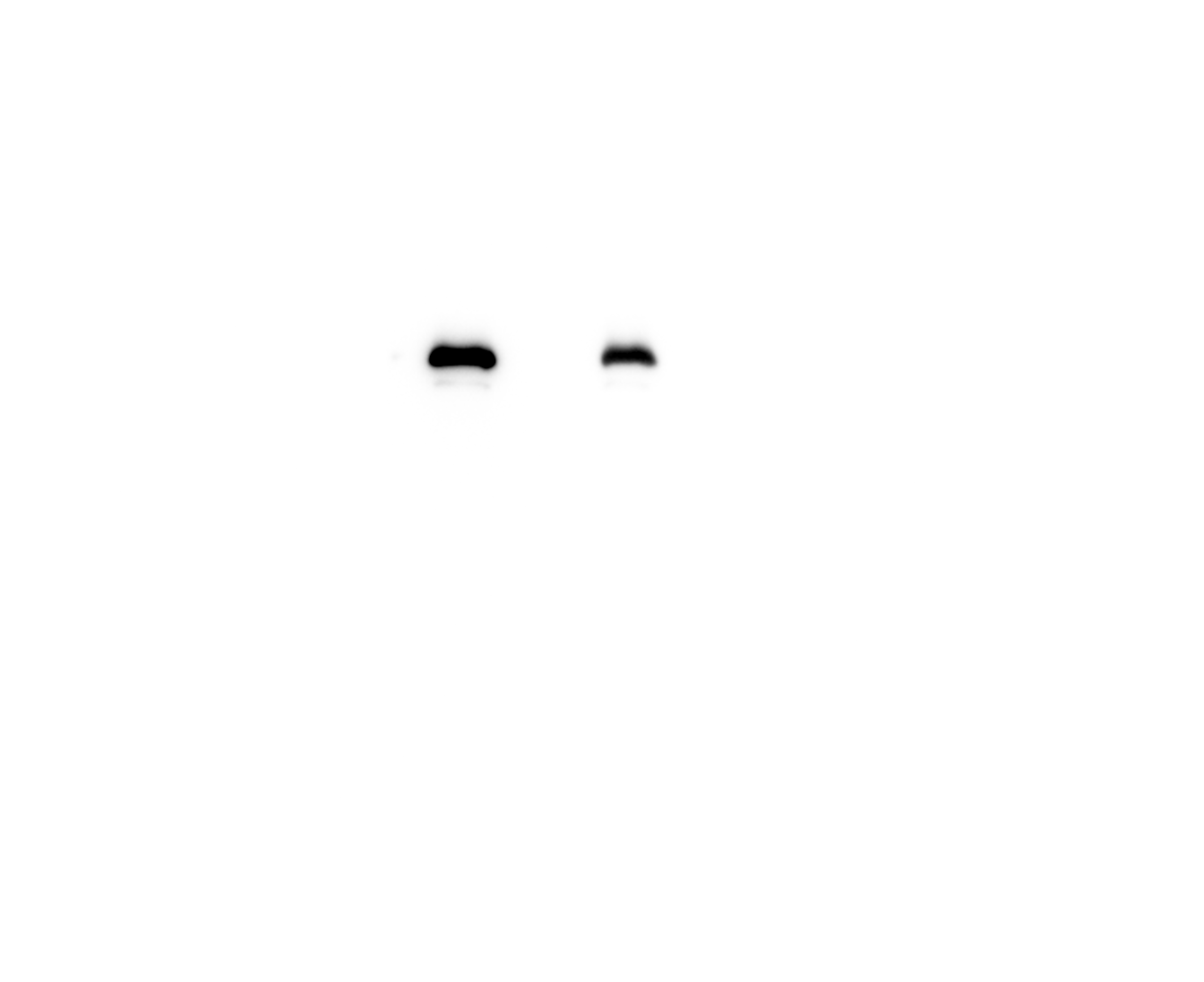

Supplement: Supplementary file 4 — Supplementary Material 4. [file 12964_2024_1770_MOESM4_ESM.zip › SENP3 TAM WB/WB-Figure4/A IRF4 SENP3 co-IP/2023-01-18 IRF4 SENP3 CO-IP/INPUT FLAG IRF4/INPUT FLAG IRF4 2.8S 0126.Tif]

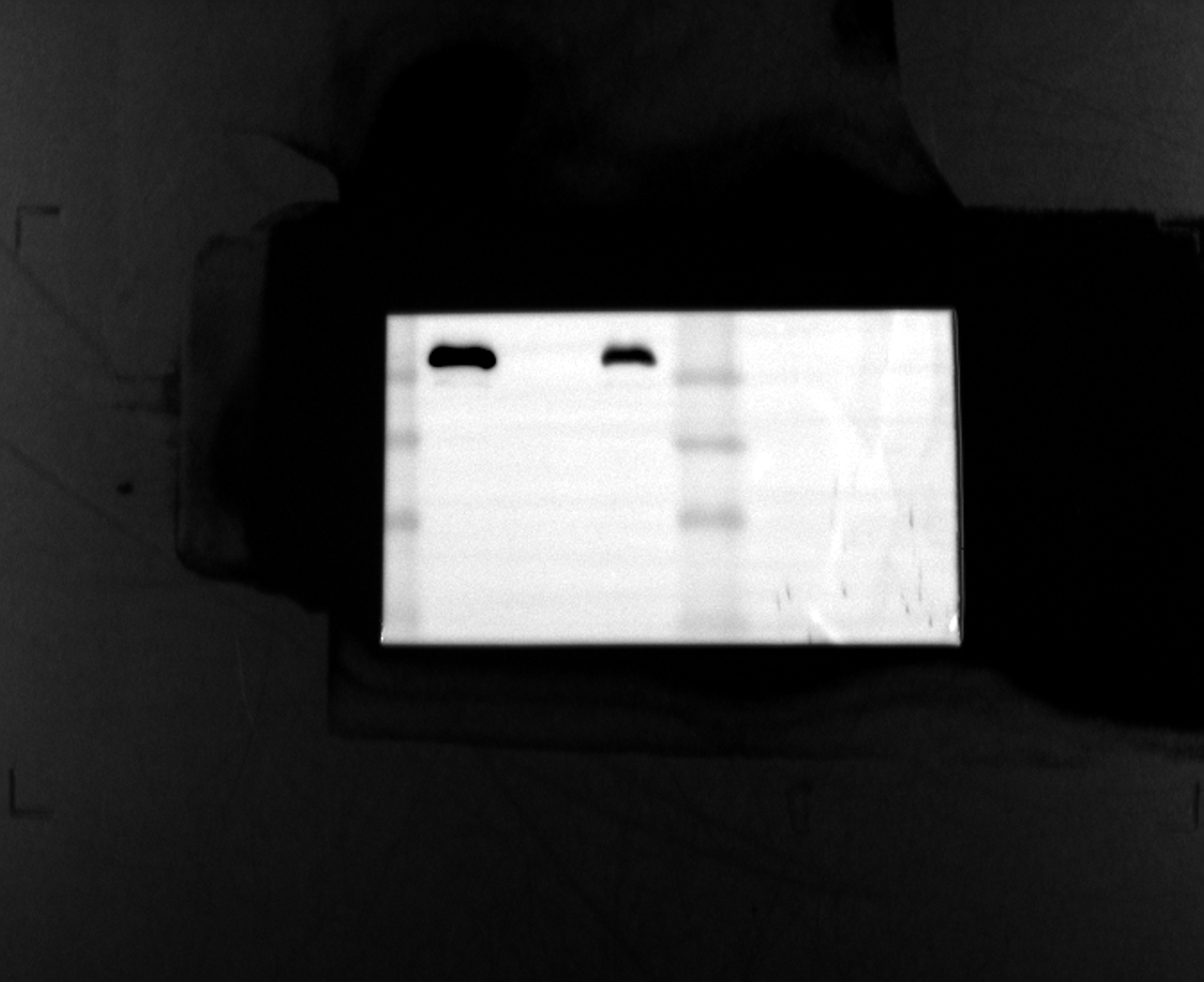

Supplement: Supplementary file 4 — Supplementary Material 4. [file 12964_2024_1770_MOESM4_ESM.zip › SENP3 TAM WB/WB-Figure4/A IRF4 SENP3 co-IP/2023-01-18 IRF4 SENP3 CO-IP/INPUT FLAG IRF4/INPUT FLAG IRF4 2.8S M 0126.Tif]

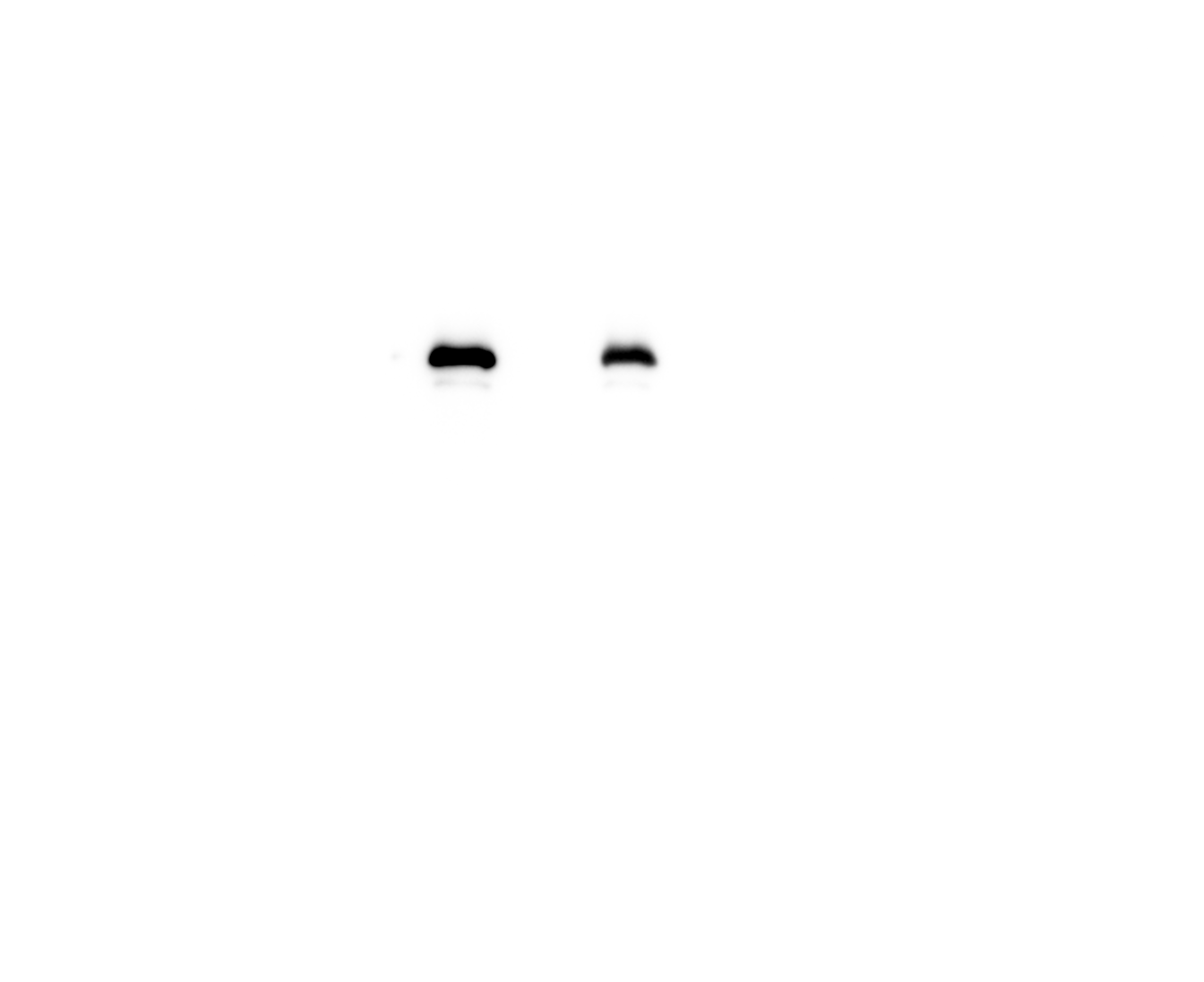

Supplement: Supplementary file 4 — Supplementary Material 4. [file 12964_2024_1770_MOESM4_ESM.zip › SENP3 TAM WB/WB-Figure4/A IRF4 SENP3 co-IP/2023-01-18 IRF4 SENP3 CO-IP/INPUT FLAG IRF4/INPUT FLAG IRF4 3.1S 0126.Tif]

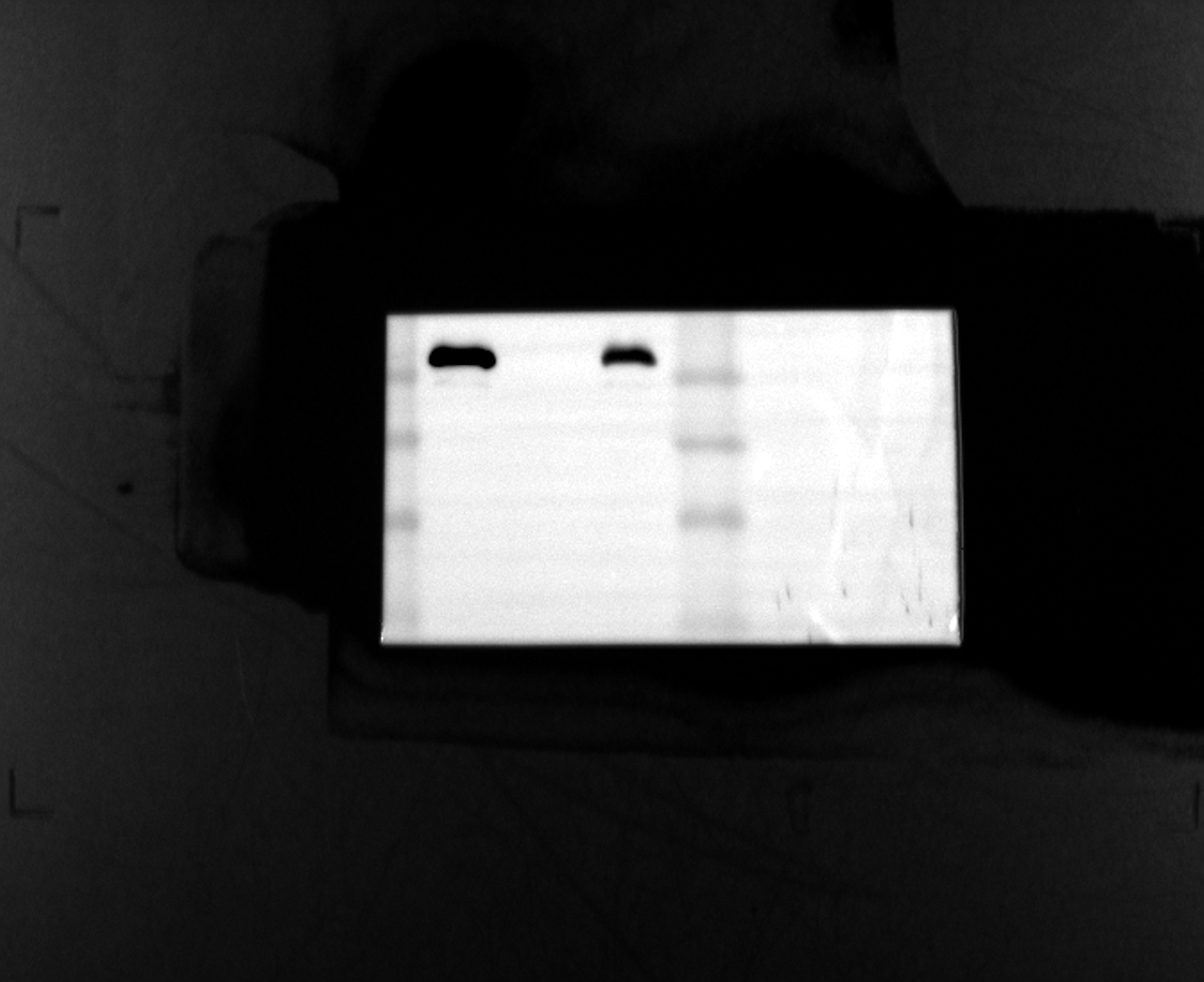

Supplement: Supplementary file 4 — Supplementary Material 4. [file 12964_2024_1770_MOESM4_ESM.zip › SENP3 TAM WB/WB-Figure4/A IRF4 SENP3 co-IP/2023-01-18 IRF4 SENP3 CO-IP/INPUT FLAG IRF4/INPUT FLAG IRF4 3.1S M 0126.Tif]

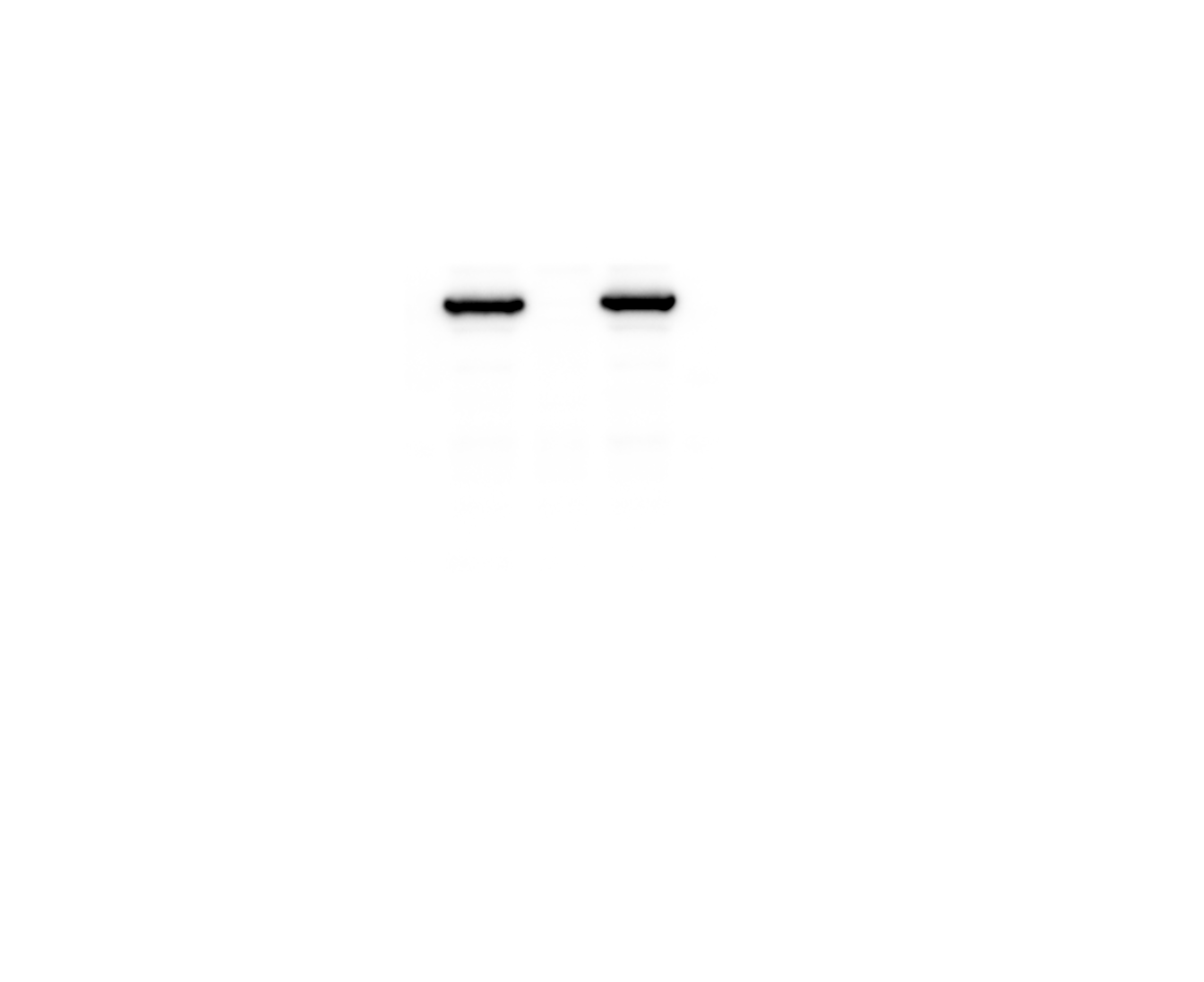

Supplement: Supplementary file 4 — Supplementary Material 4. [file 12964_2024_1770_MOESM4_ESM.zip › SENP3 TAM WB/WB-Figure4/A IRF4 SENP3 co-IP/2023-01-18 IRF4 SENP3 CO-IP/INPUT IRF4 0.4S.Tif]

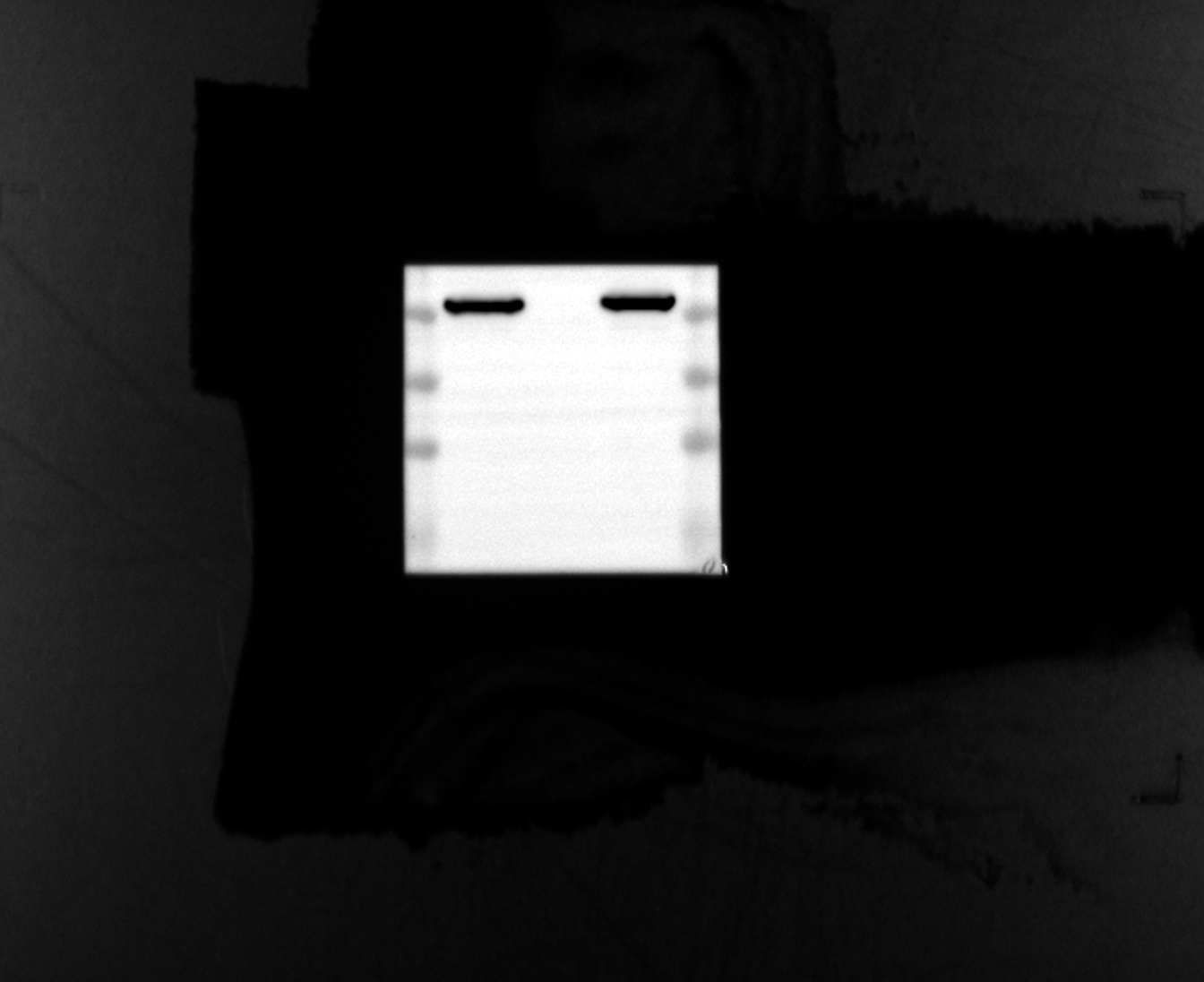

Supplement: Supplementary file 4 — Supplementary Material 4. [file 12964_2024_1770_MOESM4_ESM.zip › SENP3 TAM WB/WB-Figure4/A IRF4 SENP3 co-IP/2023-01-18 IRF4 SENP3 CO-IP/INPUT IRF4 m 0.4S.Tif]

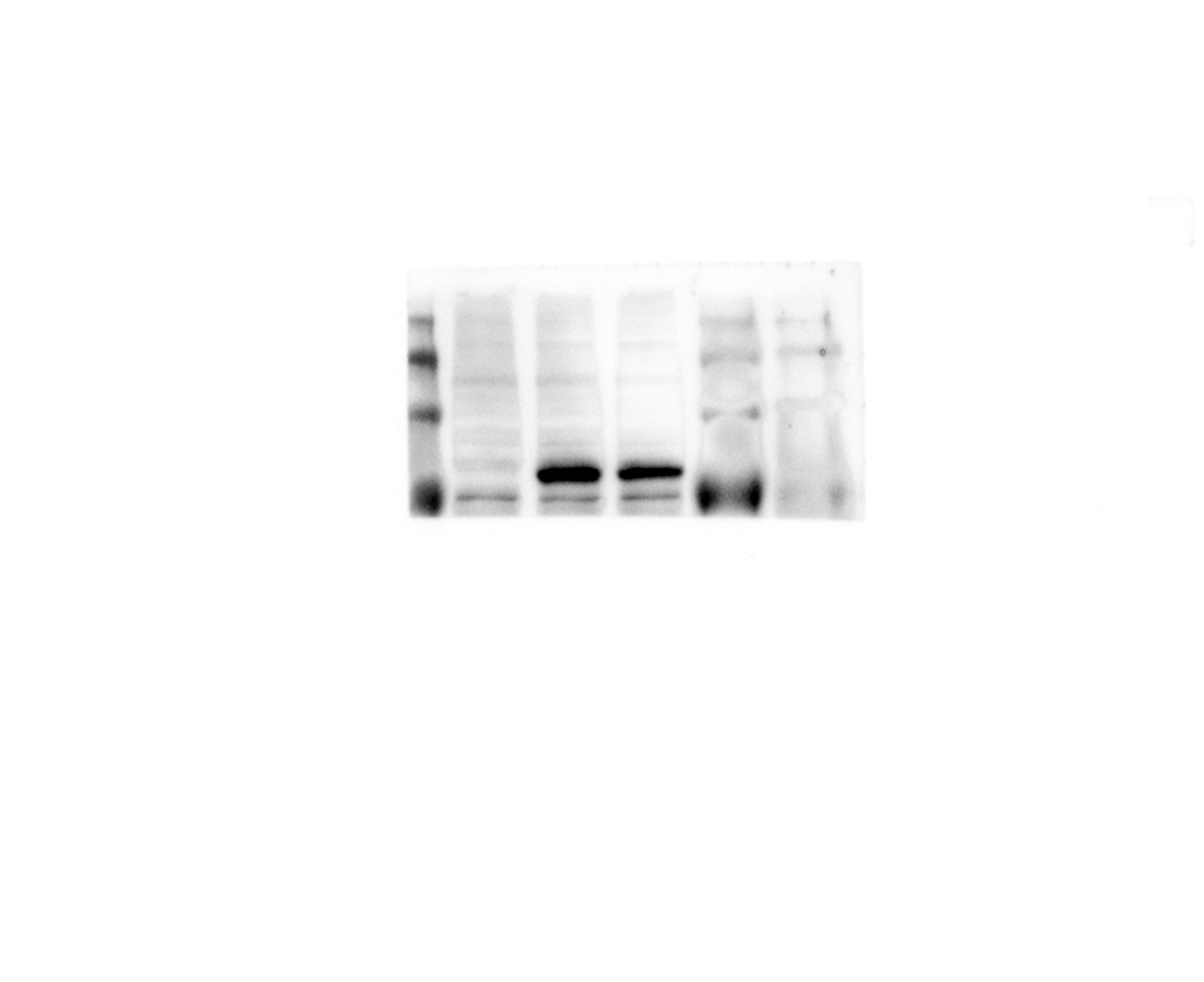

Supplement: Supplementary file 4 — Supplementary Material 4. [file 12964_2024_1770_MOESM4_ESM.zip › SENP3 TAM WB/WB-Figure4/A IRF4 SENP3 co-IP/2023-01-18 IRF4 SENP3 CO-IP/INPUT MYC SENP3/2 INPUT MYC SENP3 2.0S 0120.Tif]

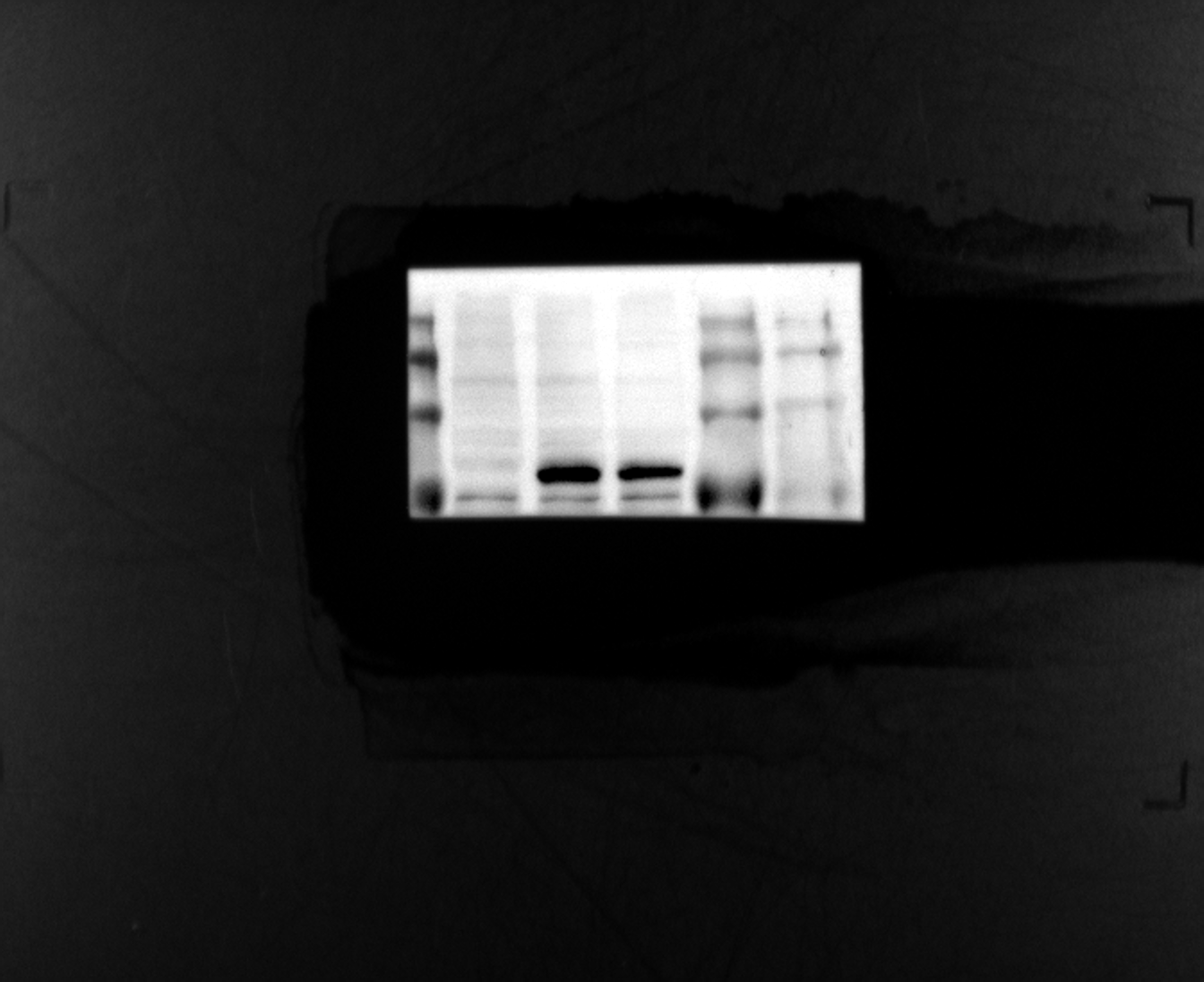

Supplement: Supplementary file 4 — Supplementary Material 4. [file 12964_2024_1770_MOESM4_ESM.zip › SENP3 TAM WB/WB-Figure4/A IRF4 SENP3 co-IP/2023-01-18 IRF4 SENP3 CO-IP/INPUT MYC SENP3/2 INPUT MYC SENP3 M 2.0S 0120.Tif]

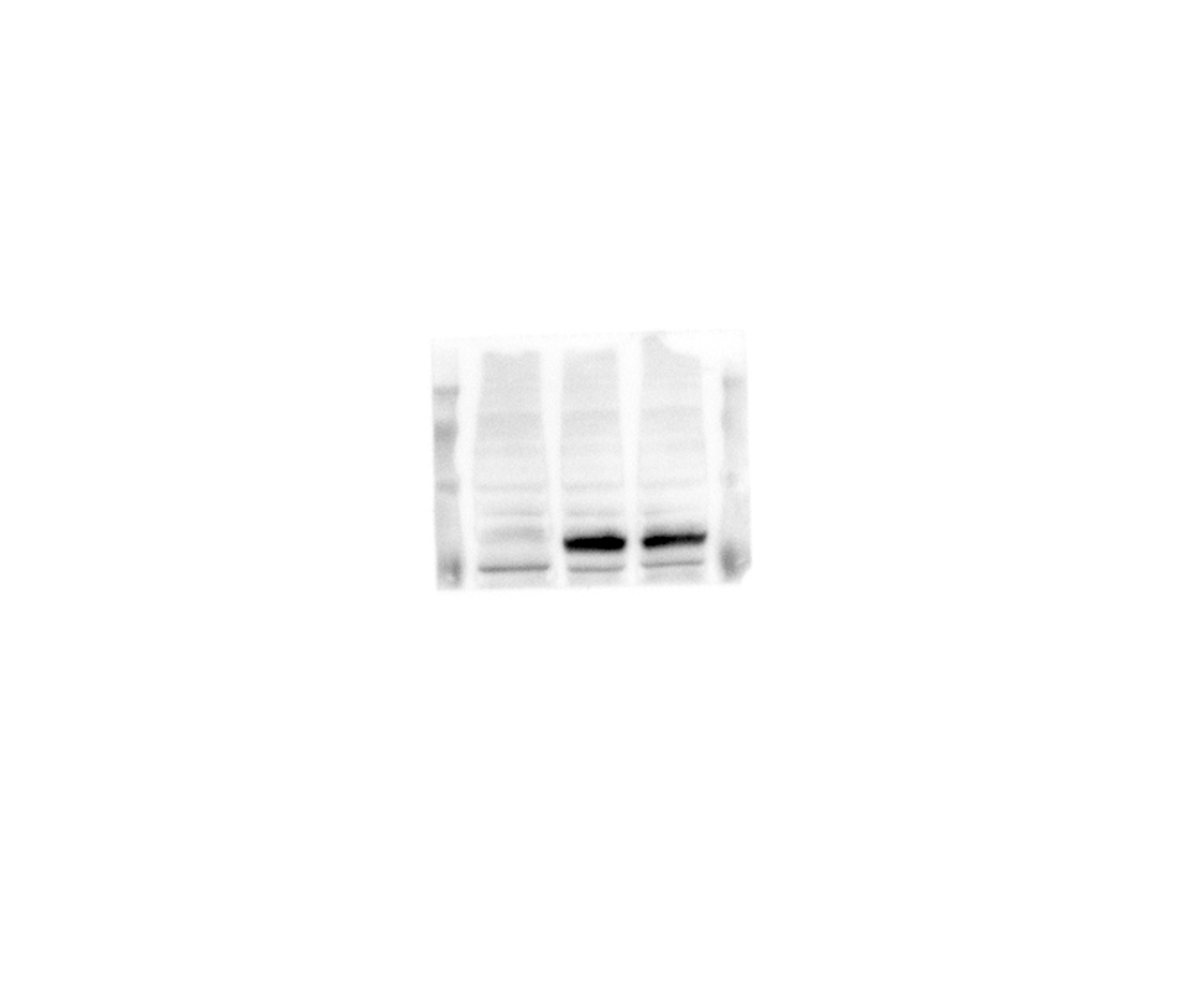

Supplement: Supplementary file 4 — Supplementary Material 4. [file 12964_2024_1770_MOESM4_ESM.zip › SENP3 TAM WB/WB-Figure4/A IRF4 SENP3 co-IP/2023-01-18 IRF4 SENP3 CO-IP/INPUT MYC SENP3/INPUT MYC SENP3 1.0S 0120.Tif]

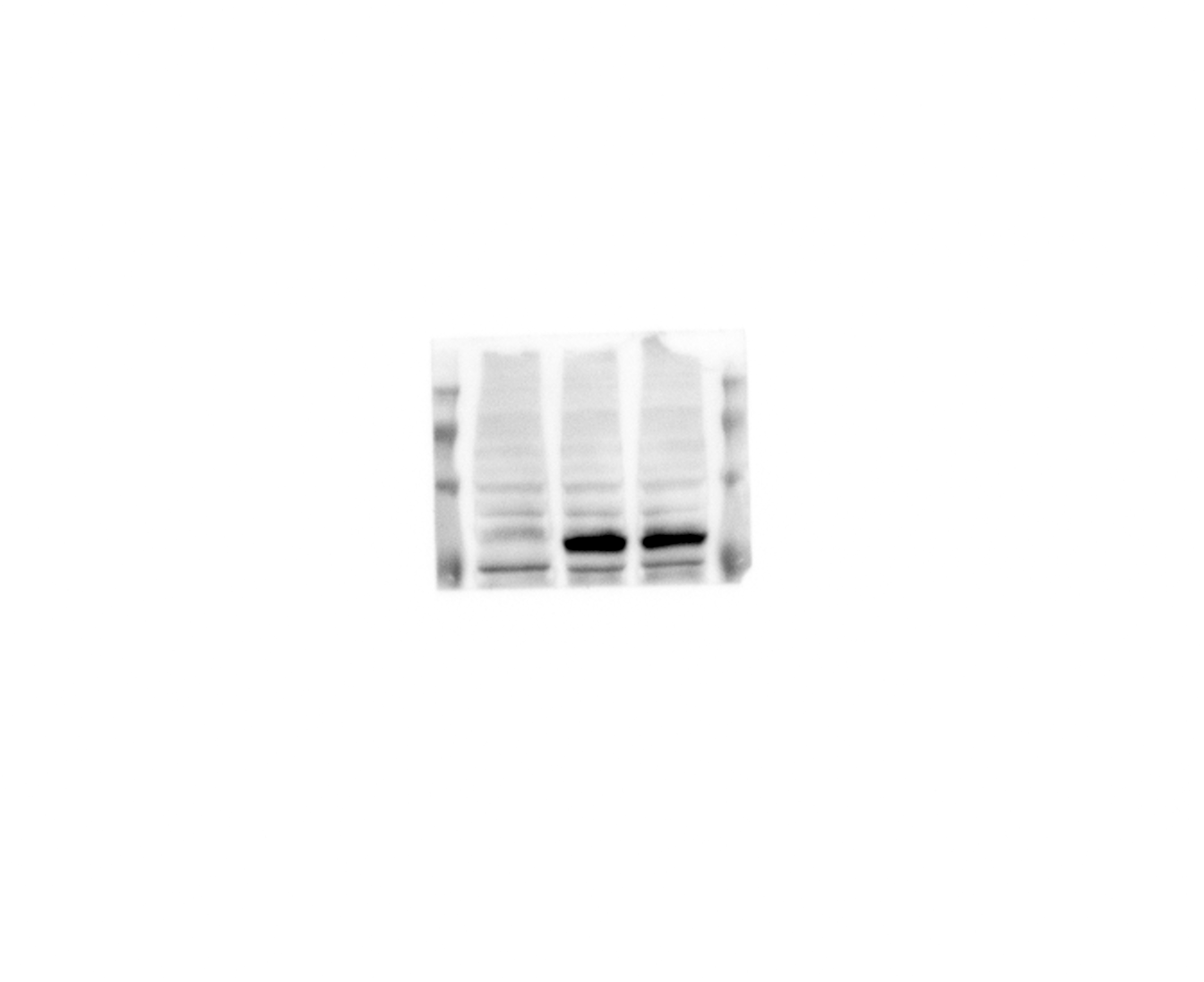

Supplement: Supplementary file 4 — Supplementary Material 4. [file 12964_2024_1770_MOESM4_ESM.zip › SENP3 TAM WB/WB-Figure4/A IRF4 SENP3 co-IP/2023-01-18 IRF4 SENP3 CO-IP/INPUT MYC SENP3/INPUT MYC SENP3 1.8S 0120.Tif]

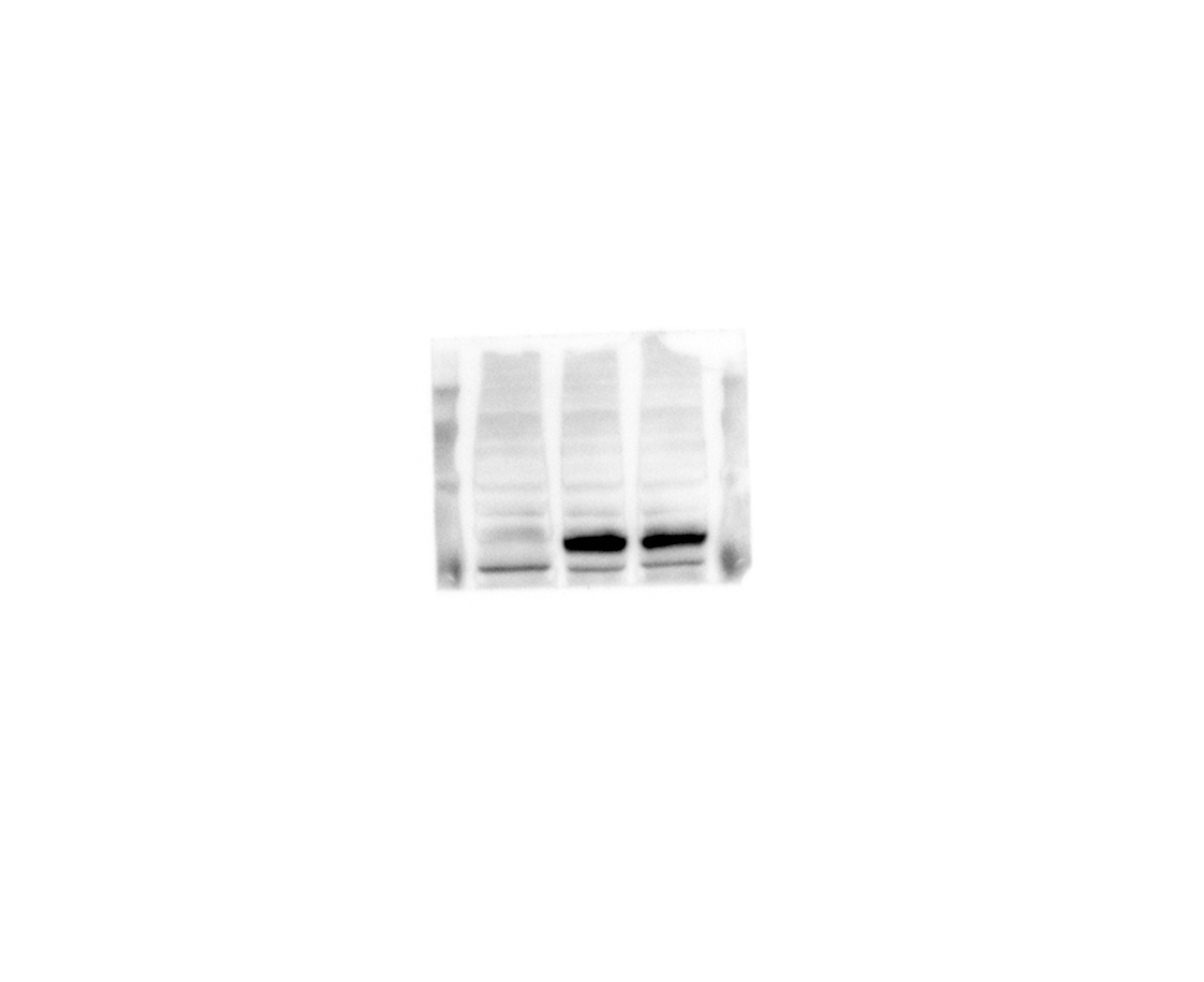

Supplement: Supplementary file 4 — Supplementary Material 4. [file 12964_2024_1770_MOESM4_ESM.zip › SENP3 TAM WB/WB-Figure4/A IRF4 SENP3 co-IP/2023-01-18 IRF4 SENP3 CO-IP/INPUT MYC SENP3/INPUT MYC SENP3 2.0S 0120.Tif]

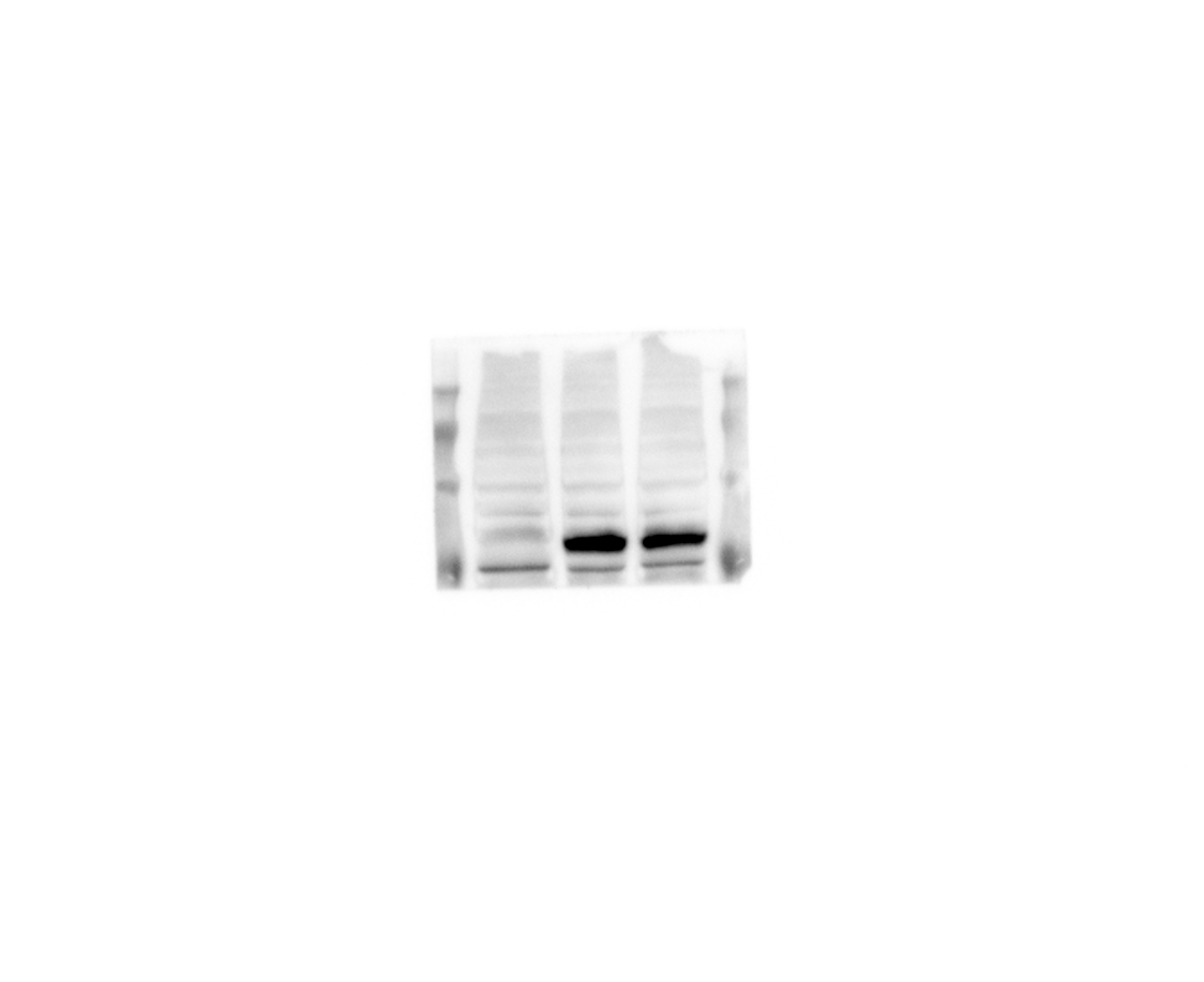

Supplement: Supplementary file 4 — Supplementary Material 4. [file 12964_2024_1770_MOESM4_ESM.zip › SENP3 TAM WB/WB-Figure4/A IRF4 SENP3 co-IP/2023-01-18 IRF4 SENP3 CO-IP/INPUT MYC SENP3/INPUT MYC SENP3 3.0S 0120.Tif]

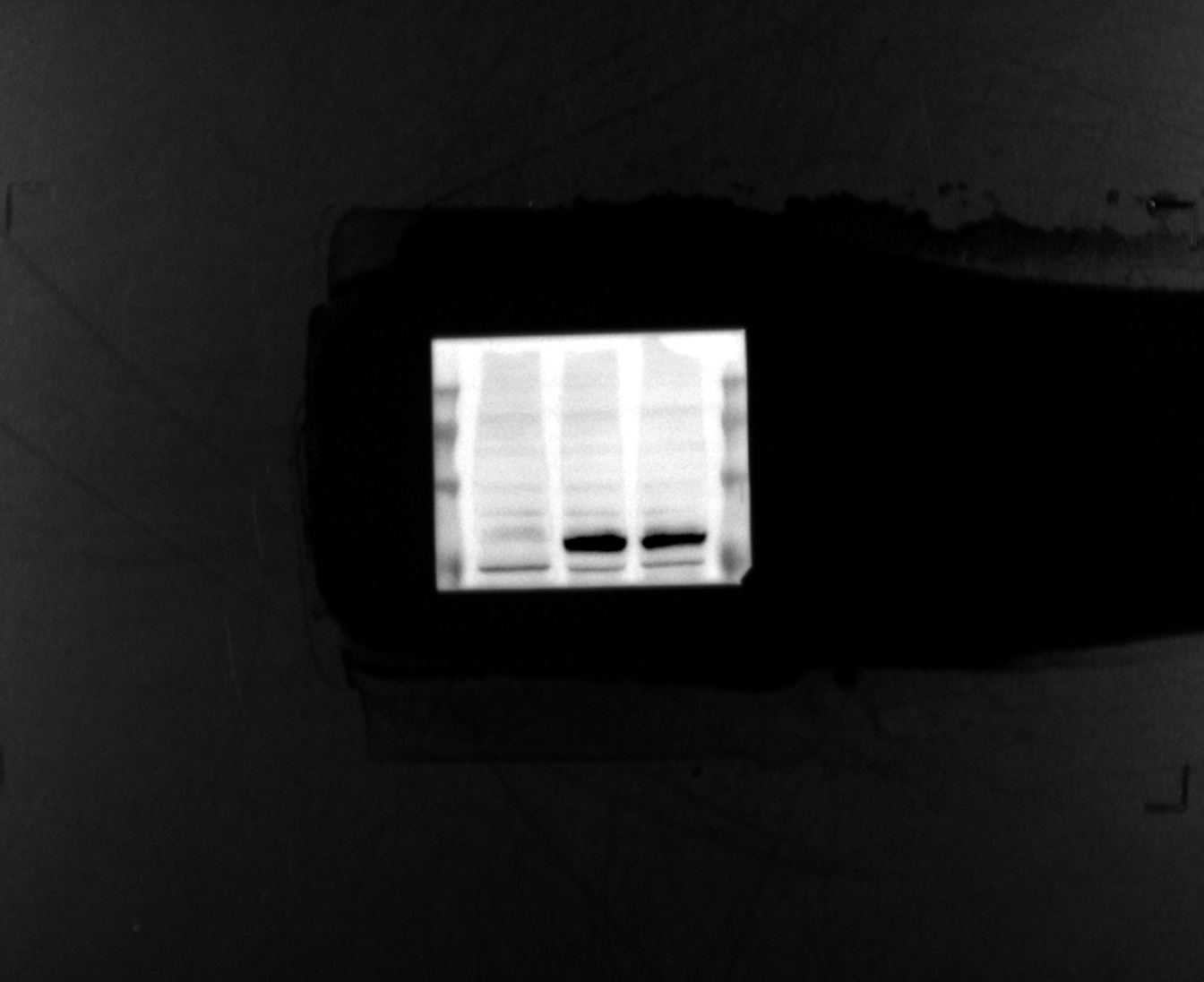

Supplement: Supplementary file 4 — Supplementary Material 4. [file 12964_2024_1770_MOESM4_ESM.zip › SENP3 TAM WB/WB-Figure4/A IRF4 SENP3 co-IP/2023-01-18 IRF4 SENP3 CO-IP/INPUT MYC SENP3/INPUT MYC SENP3 M 1.0S 0120.Tif]

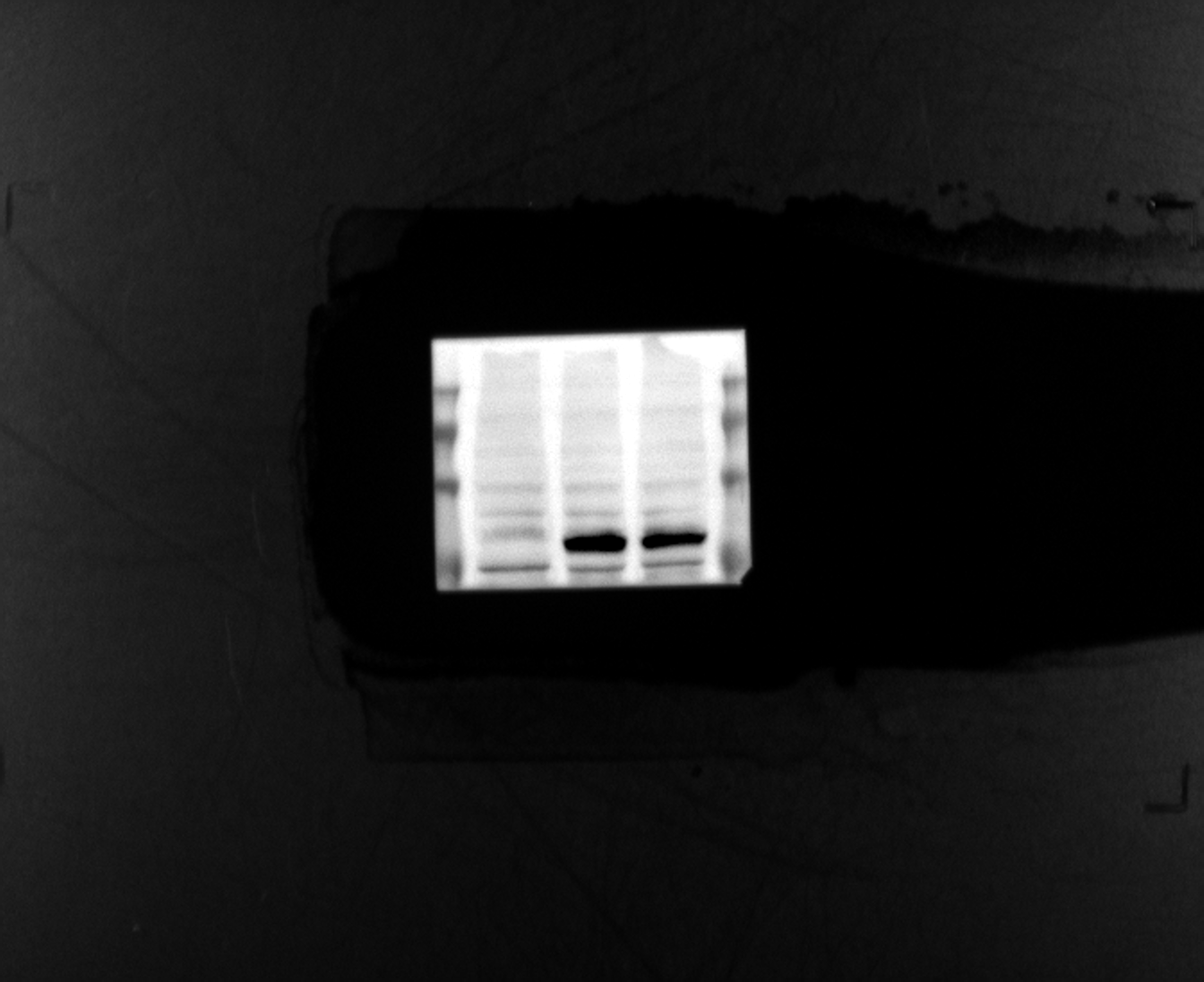

Supplement: Supplementary file 4 — Supplementary Material 4. [file 12964_2024_1770_MOESM4_ESM.zip › SENP3 TAM WB/WB-Figure4/A IRF4 SENP3 co-IP/2023-01-18 IRF4 SENP3 CO-IP/INPUT MYC SENP3/INPUT MYC SENP3 M 1.8S 0120.Tif]

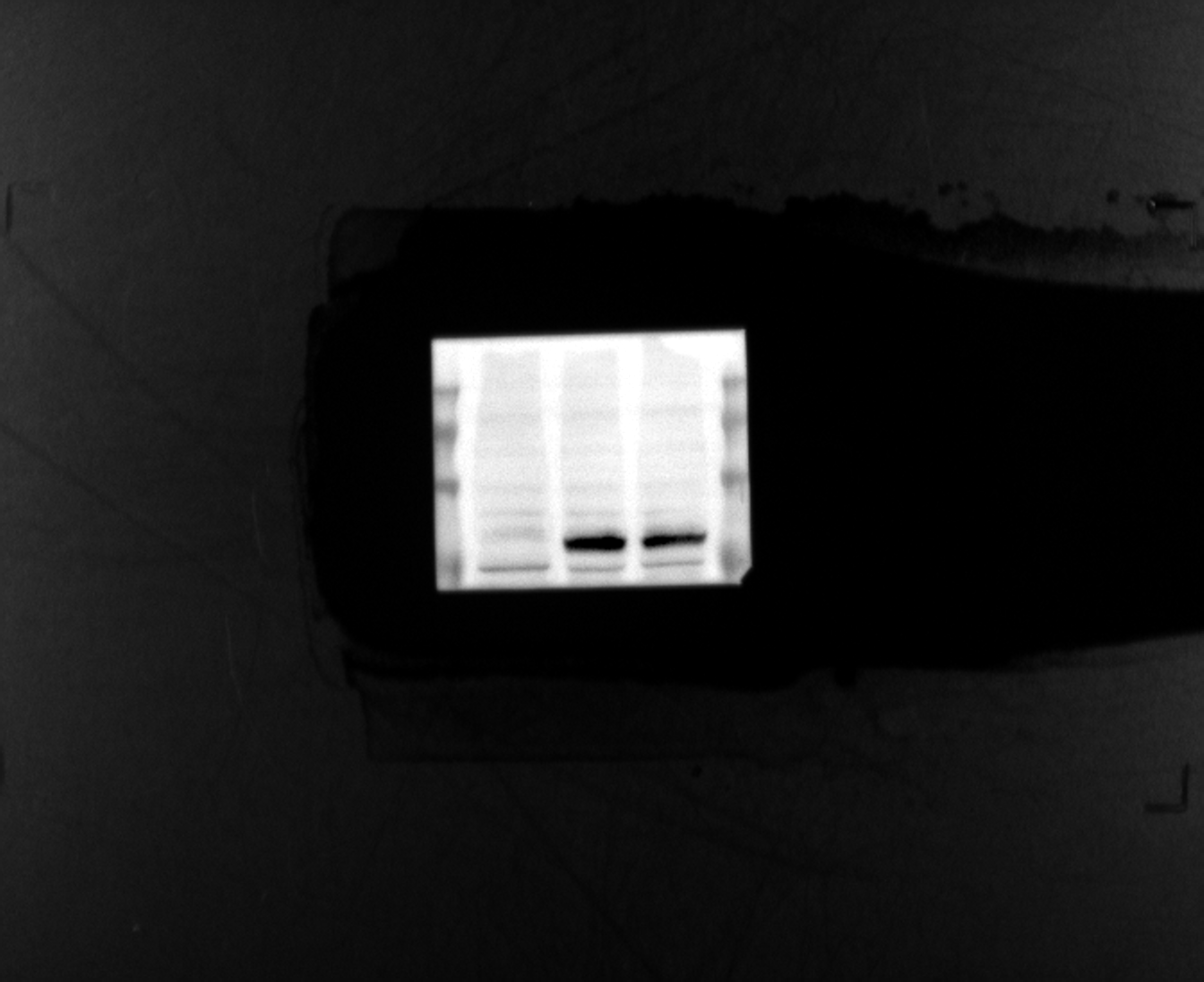

Supplement: Supplementary file 4 — Supplementary Material 4. [file 12964_2024_1770_MOESM4_ESM.zip › SENP3 TAM WB/WB-Figure4/A IRF4 SENP3 co-IP/2023-01-18 IRF4 SENP3 CO-IP/INPUT MYC SENP3/INPUT MYC SENP3 M 2.0S 0120.Tif]

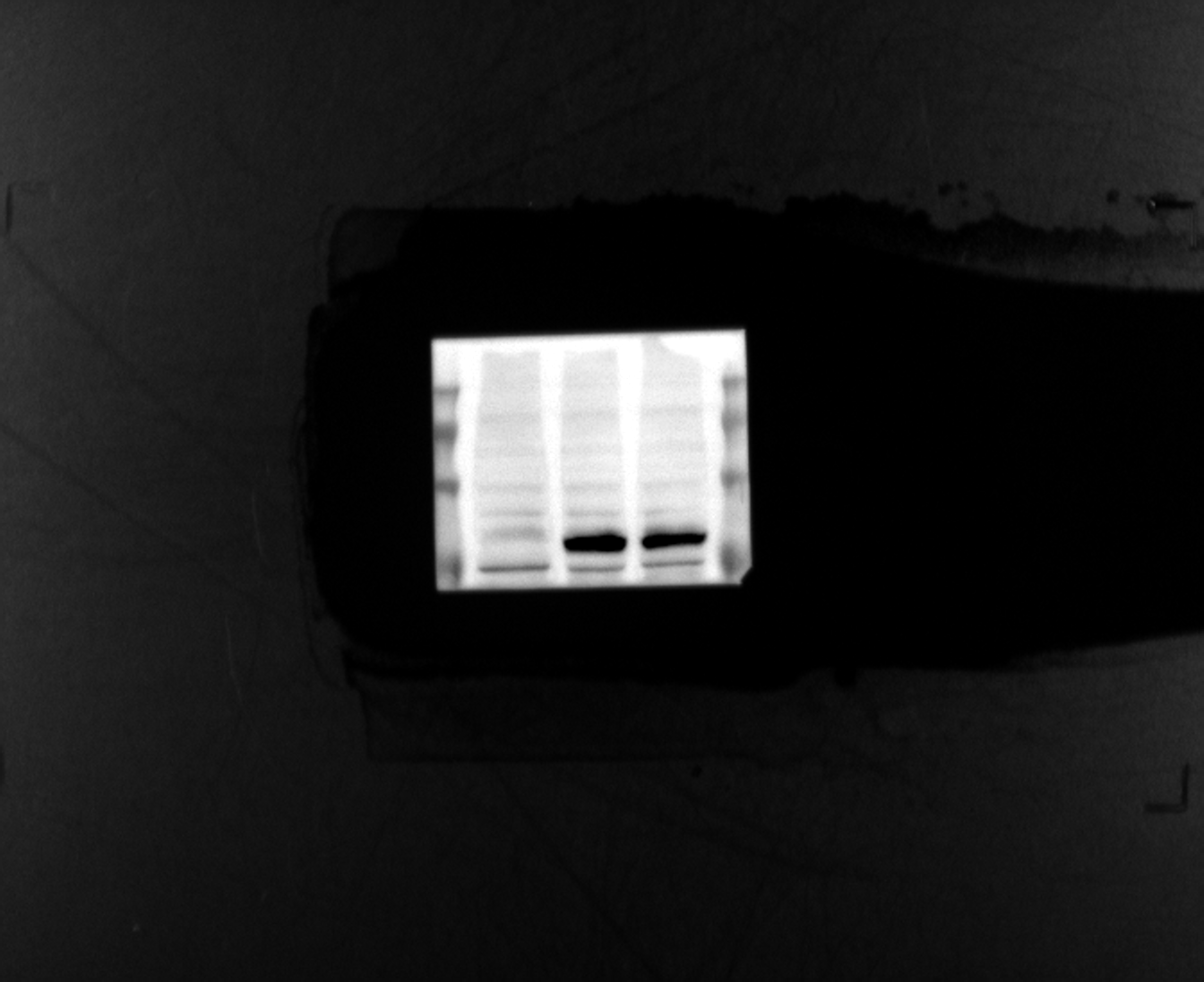

Supplement: Supplementary file 4 — Supplementary Material 4. [file 12964_2024_1770_MOESM4_ESM.zip › SENP3 TAM WB/WB-Figure4/A IRF4 SENP3 co-IP/2023-01-18 IRF4 SENP3 CO-IP/INPUT MYC SENP3/INPUT MYC SENP3 M 3.0S 0120.Tif]

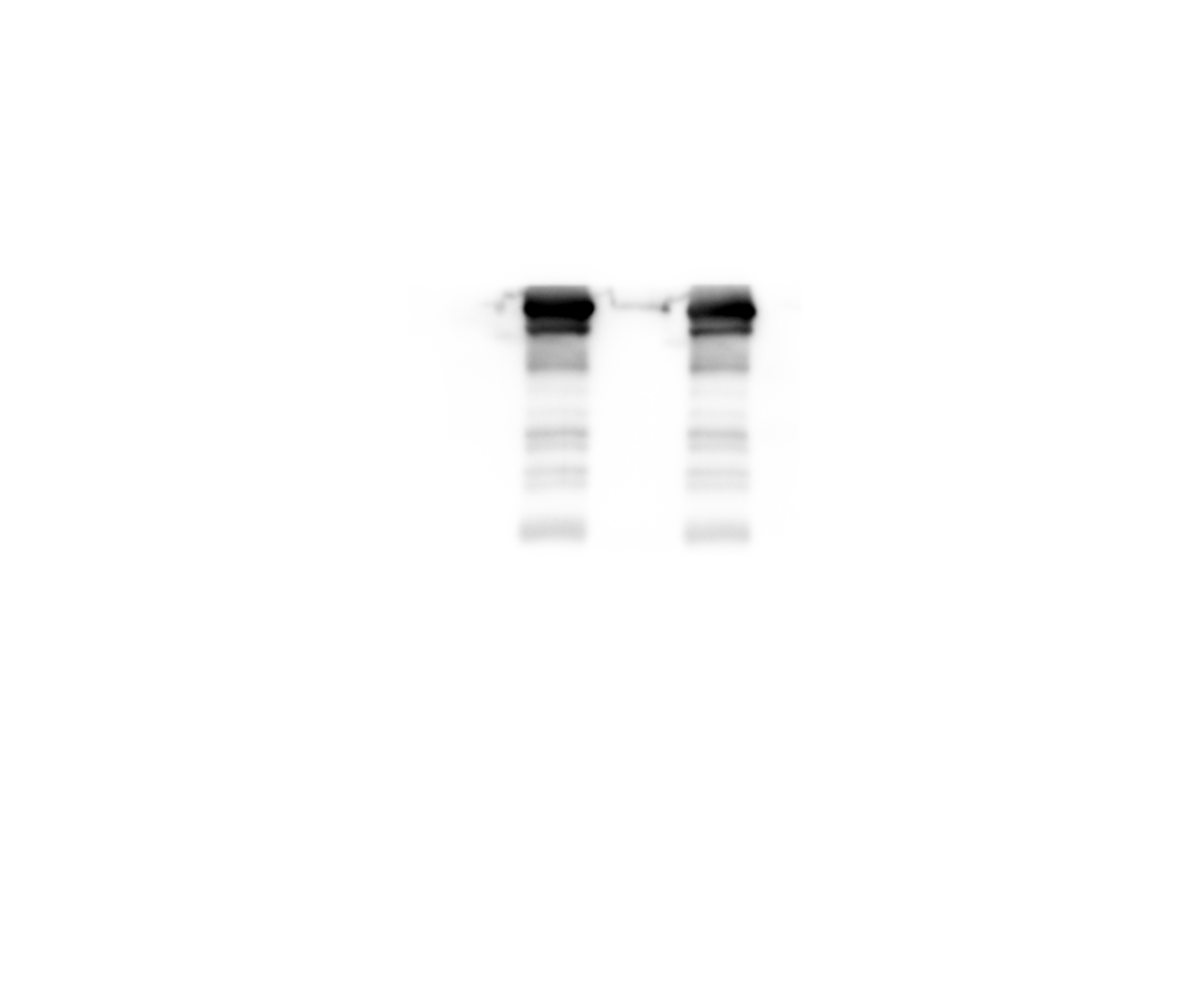

Supplement: Supplementary file 4 — Supplementary Material 4. [file 12964_2024_1770_MOESM4_ESM.zip › SENP3 TAM WB/WB-Figure4/A IRF4 SENP3 co-IP/2023-01-18 IRF4 SENP3 CO-IP/IP FLAG IRF4/IP IRF4 0.3S 0121.Tif]

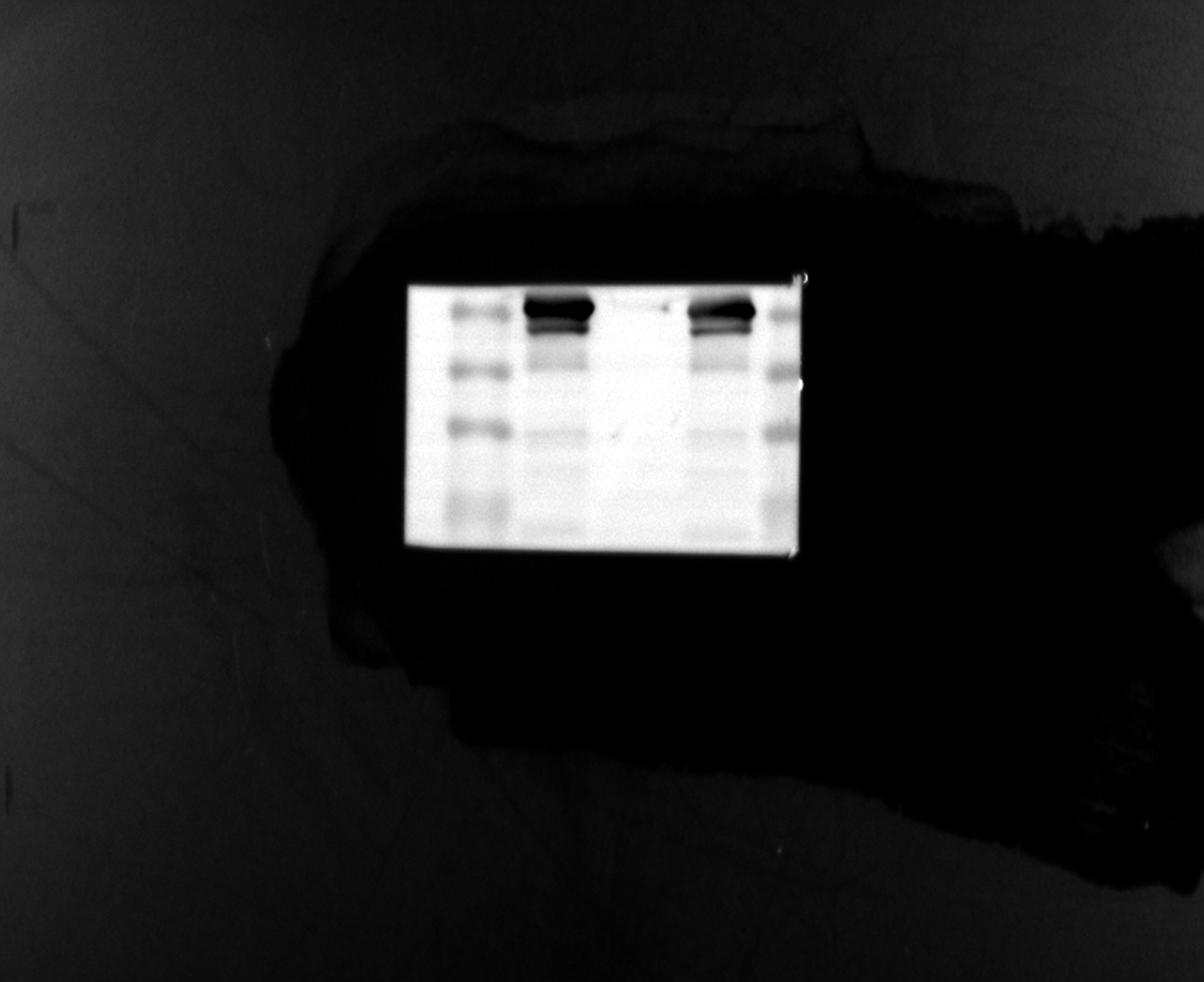

Supplement: Supplementary file 4 — Supplementary Material 4. [file 12964_2024_1770_MOESM4_ESM.zip › SENP3 TAM WB/WB-Figure4/A IRF4 SENP3 co-IP/2023-01-18 IRF4 SENP3 CO-IP/IP FLAG IRF4/IP IRF4 m 0.3S 0121.Tif]

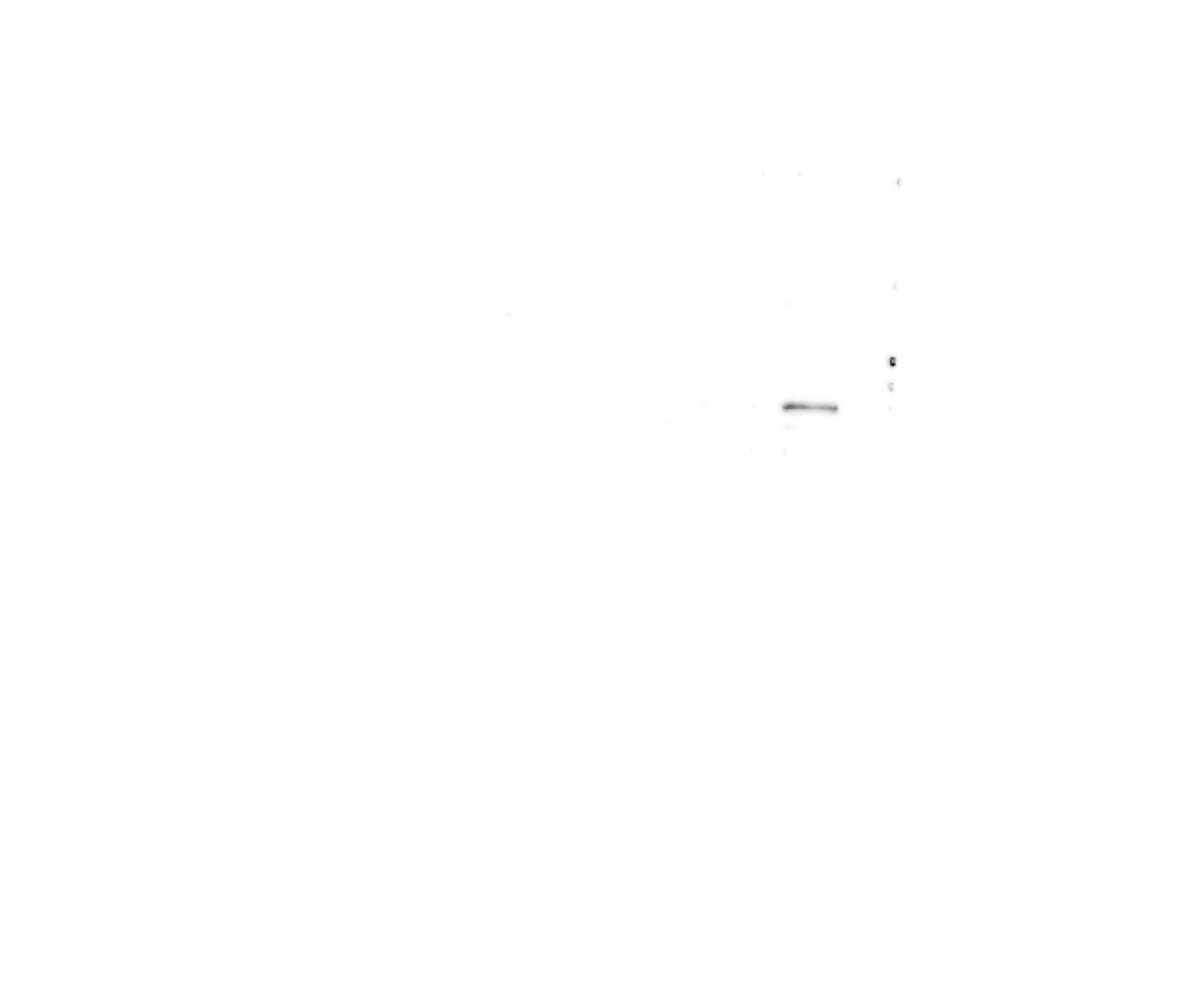

Supplement: Supplementary file 4 — Supplementary Material 4. [file 12964_2024_1770_MOESM4_ESM.zip › SENP3 TAM WB/WB-Figure4/A IRF4 SENP3 co-IP/2023-01-18 IRF4 SENP3 CO-IP/IP FLAG MYC/IP MYC SENP3 10S 0121.Tif]

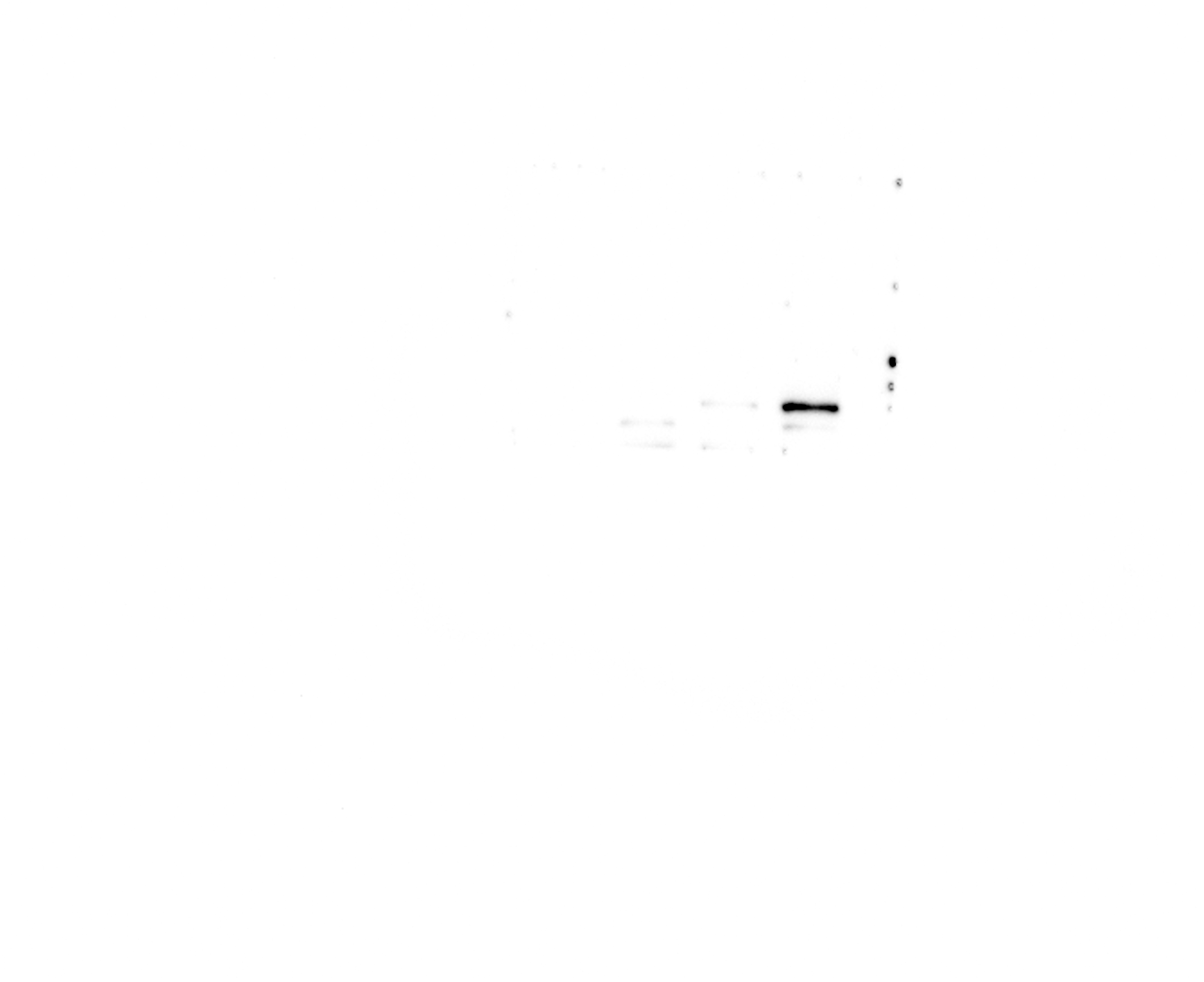

Supplement: Supplementary file 4 — Supplementary Material 4. [file 12964_2024_1770_MOESM4_ESM.zip › SENP3 TAM WB/WB-Figure4/A IRF4 SENP3 co-IP/2023-01-18 IRF4 SENP3 CO-IP/IP FLAG MYC/IP MYC SENP3 20S 0121.Tif]

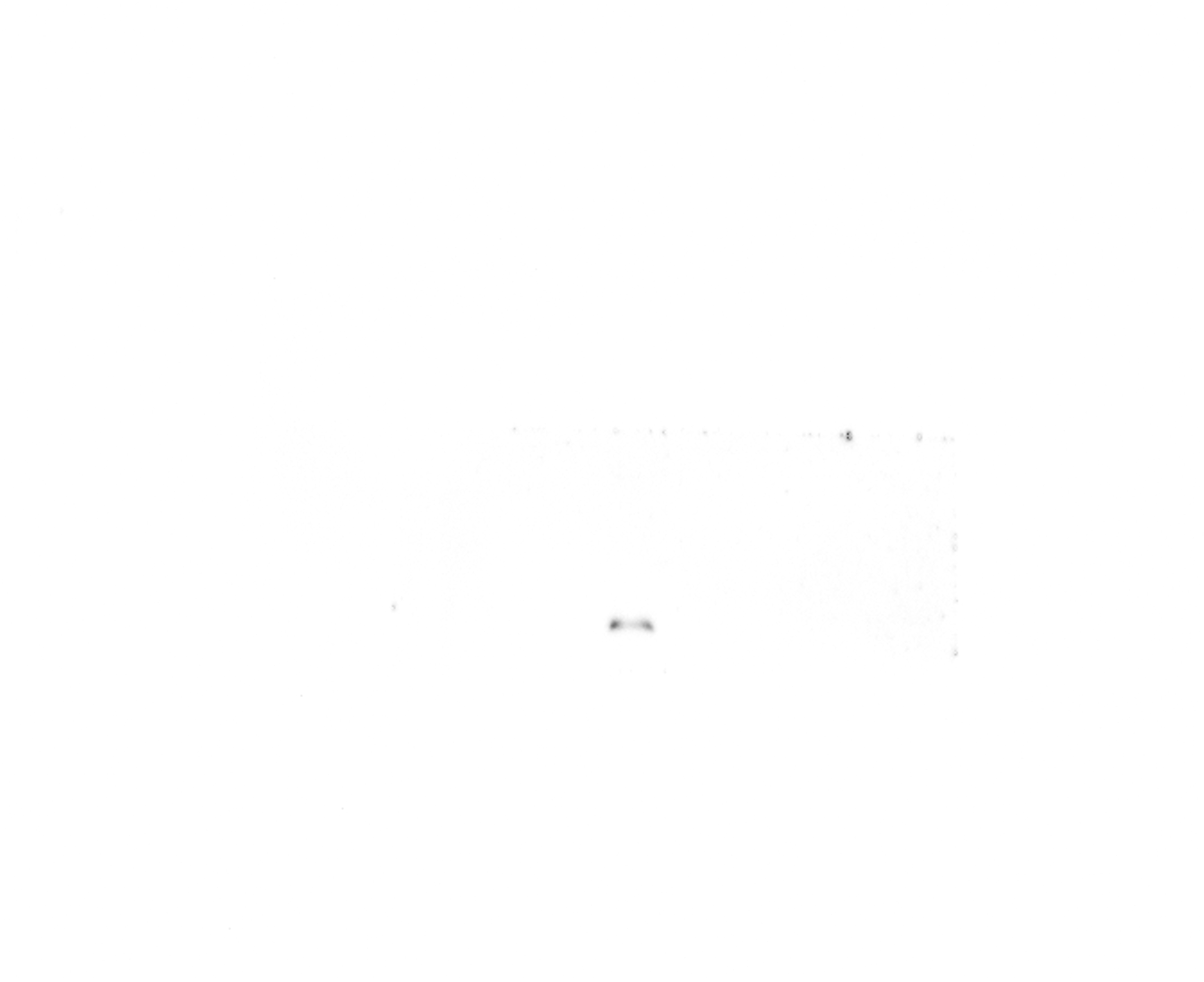

Supplement: Supplementary file 4 — Supplementary Material 4. [file 12964_2024_1770_MOESM4_ESM.zip › SENP3 TAM WB/WB-Figure4/A IRF4 SENP3 co-IP/2023-01-18 IRF4 SENP3 CO-IP/IP FLAG MYC/IP MYC SENP3 20S 0126.Tif]

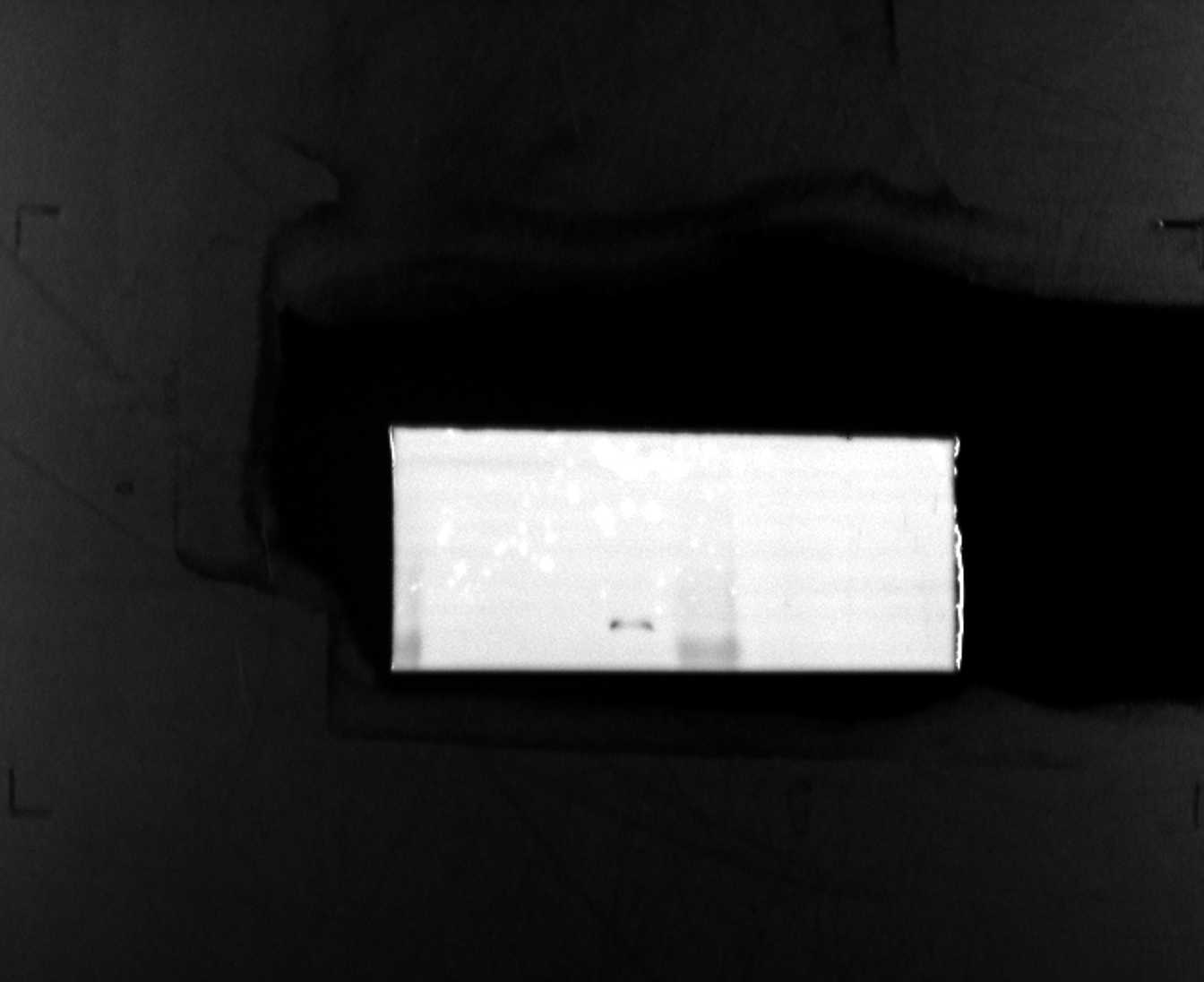

Supplement: Supplementary file 4 — Supplementary Material 4. [file 12964_2024_1770_MOESM4_ESM.zip › SENP3 TAM WB/WB-Figure4/A IRF4 SENP3 co-IP/2023-01-18 IRF4 SENP3 CO-IP/IP FLAG MYC/IP MYC SENP3 20S M 0126.Tif]

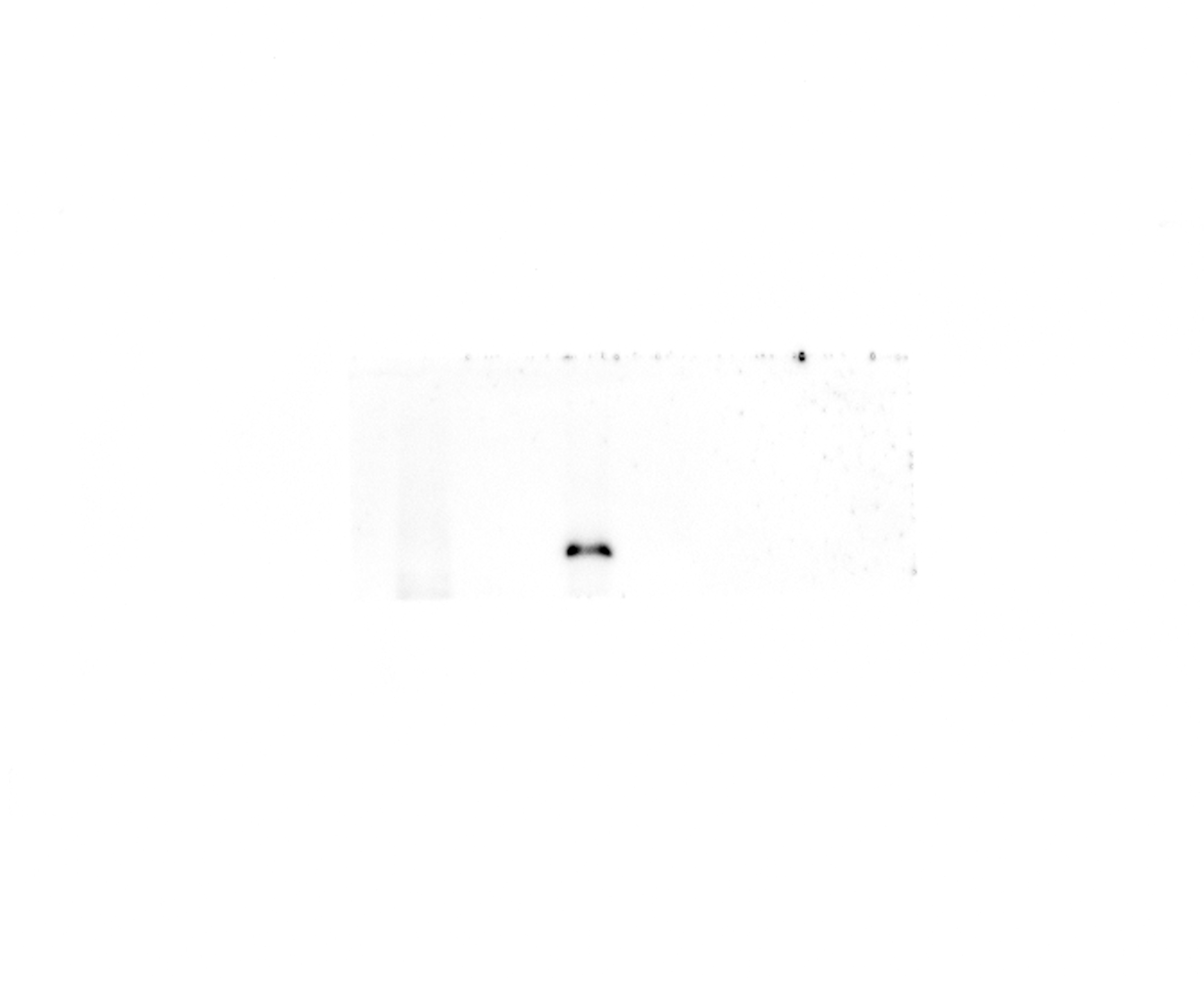

Supplement: Supplementary file 4 — Supplementary Material 4. [file 12964_2024_1770_MOESM4_ESM.zip › SENP3 TAM WB/WB-Figure4/A IRF4 SENP3 co-IP/2023-01-18 IRF4 SENP3 CO-IP/IP FLAG MYC/IP MYC SENP3 30S 0126.Tif]

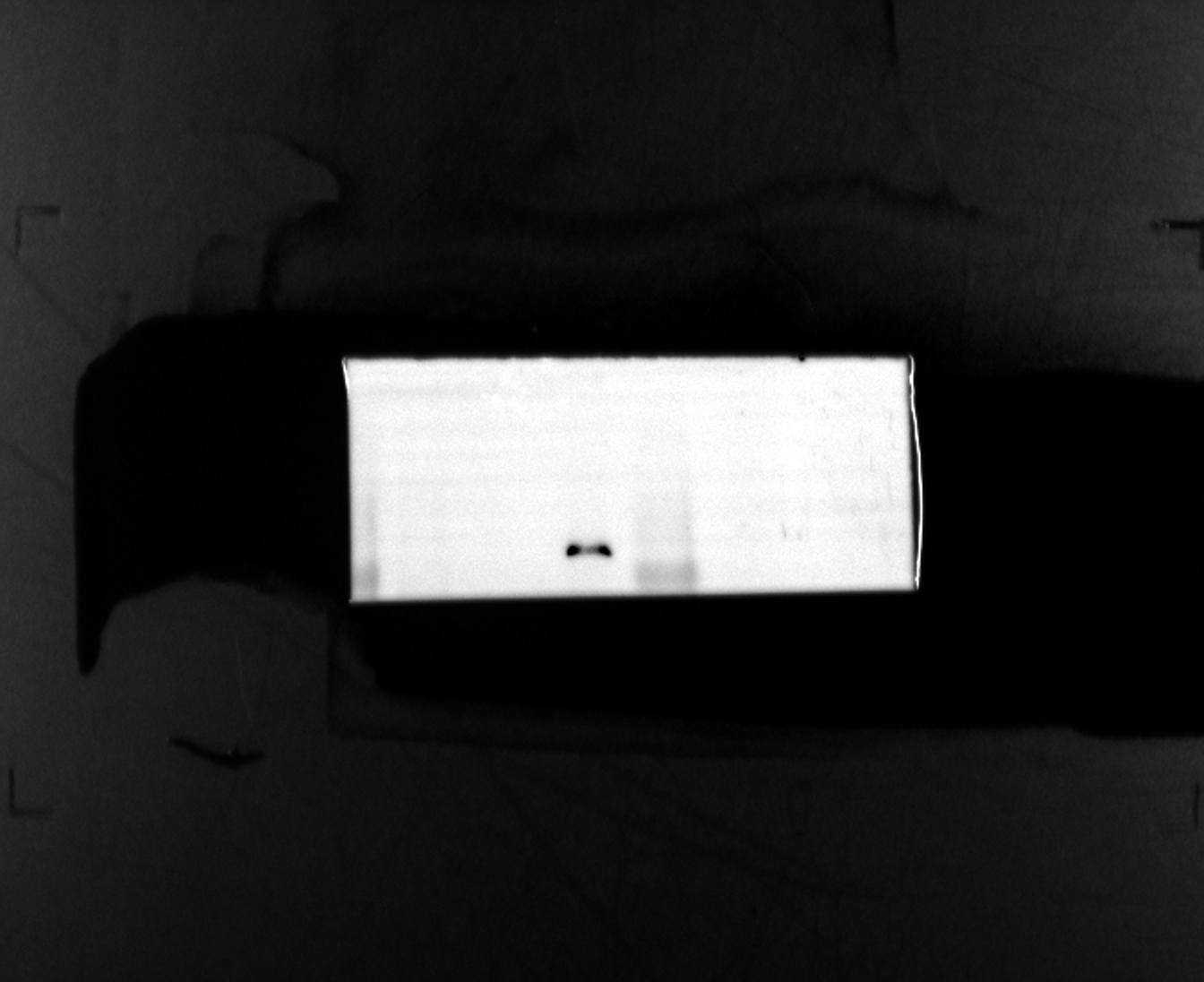

Supplement: Supplementary file 4 — Supplementary Material 4. [file 12964_2024_1770_MOESM4_ESM.zip › SENP3 TAM WB/WB-Figure4/A IRF4 SENP3 co-IP/2023-01-18 IRF4 SENP3 CO-IP/IP FLAG MYC/IP MYC SENP3 30S M 0126.Tif]

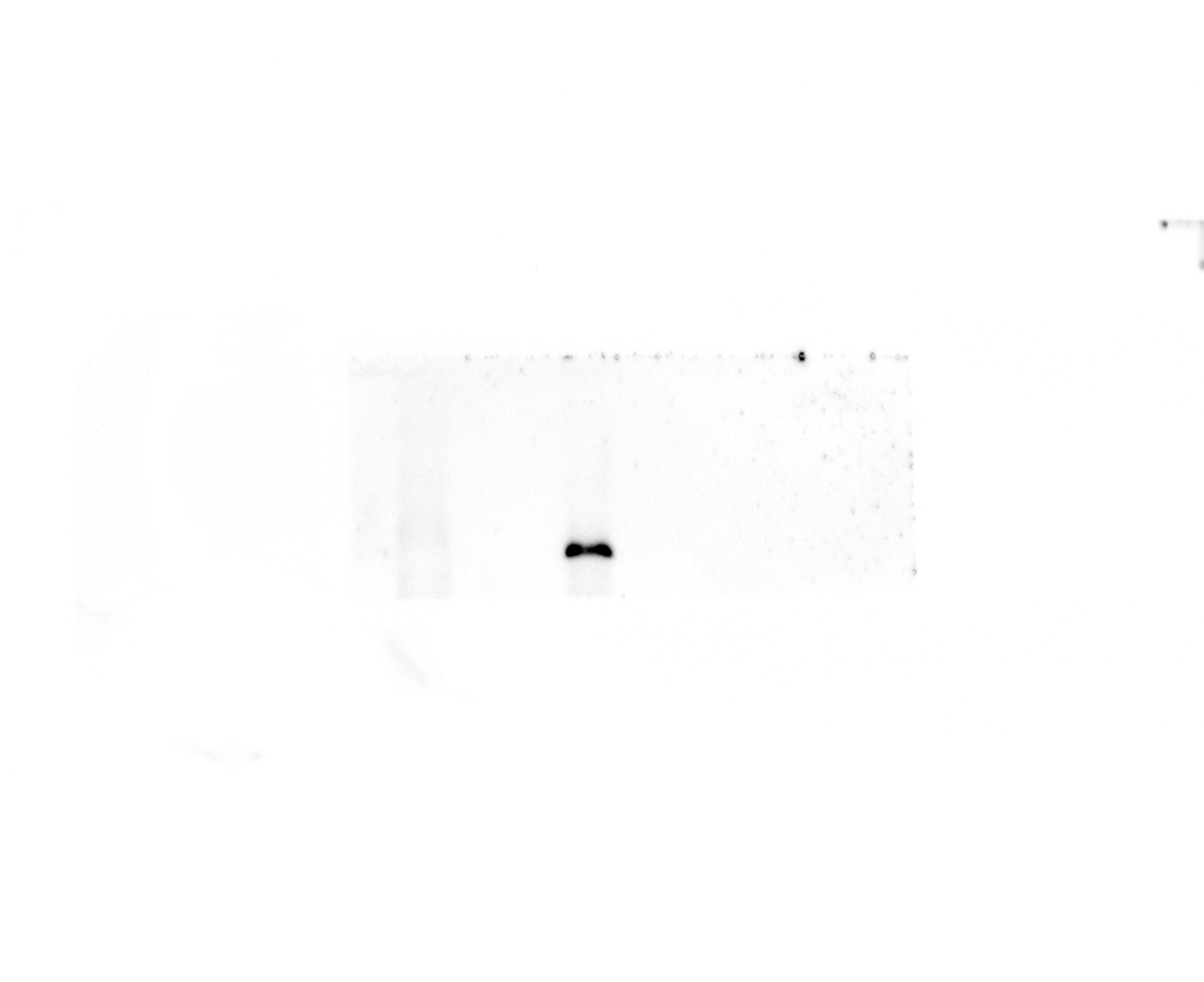

Supplement: Supplementary file 4 — Supplementary Material 4. [file 12964_2024_1770_MOESM4_ESM.zip › SENP3 TAM WB/WB-Figure4/A IRF4 SENP3 co-IP/2023-01-18 IRF4 SENP3 CO-IP/IP FLAG MYC/IP MYC SENP3 40S 0126.Tif]

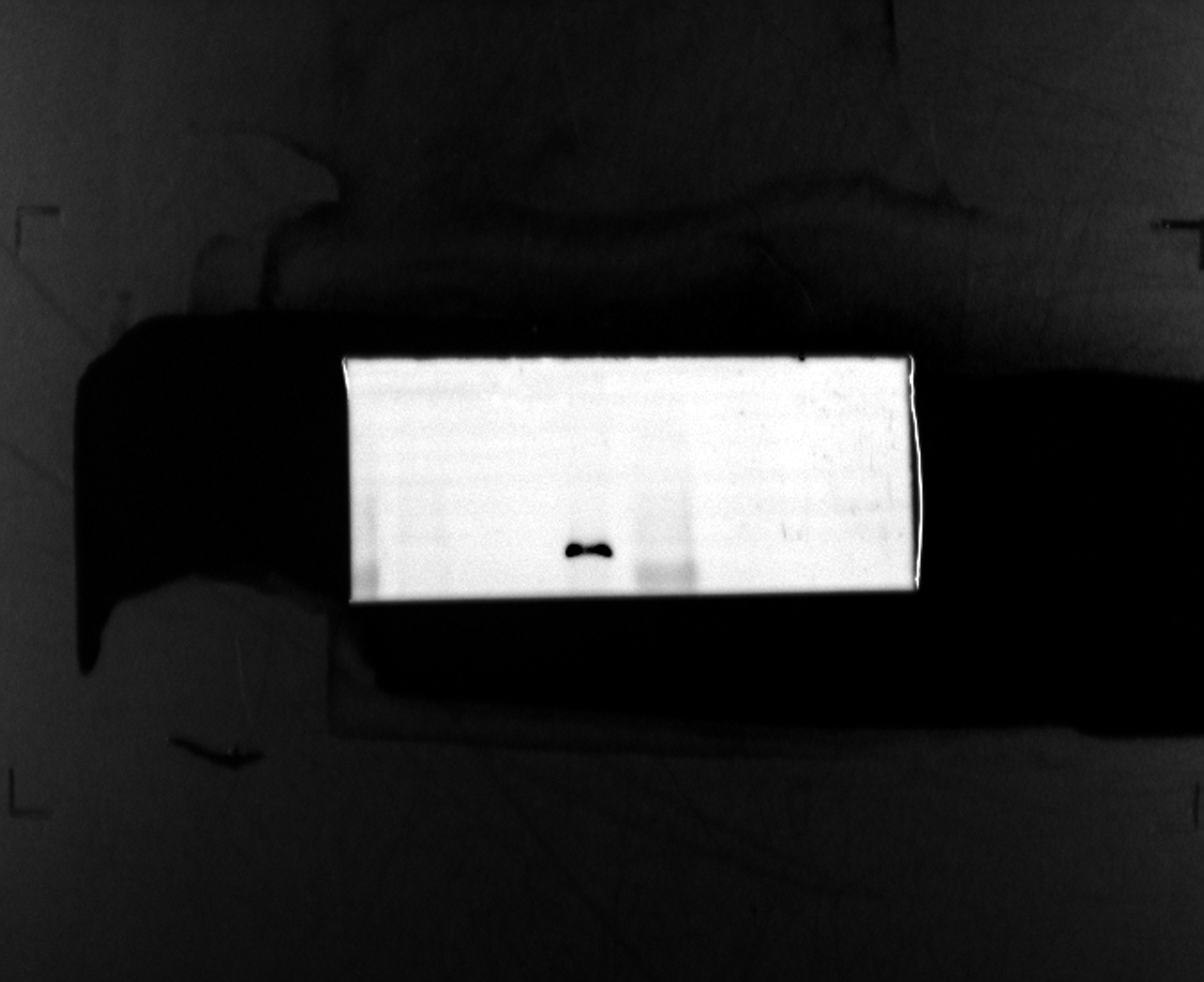

Supplement: Supplementary file 4 — Supplementary Material 4. [file 12964_2024_1770_MOESM4_ESM.zip › SENP3 TAM WB/WB-Figure4/A IRF4 SENP3 co-IP/2023-01-18 IRF4 SENP3 CO-IP/IP FLAG MYC/IP MYC SENP3 40S M 0126.Tif]

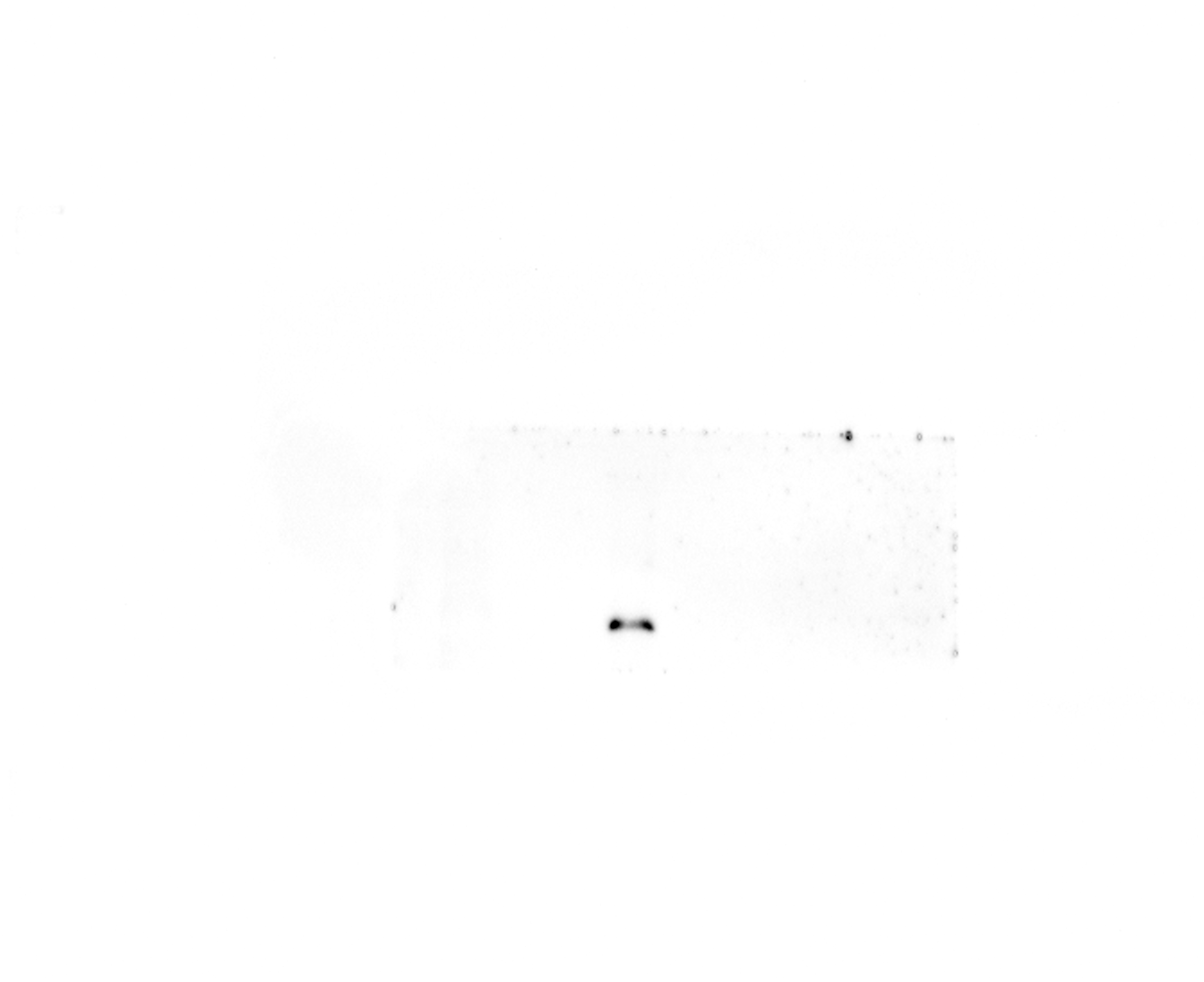

Supplement: Supplementary file 4 — Supplementary Material 4. [file 12964_2024_1770_MOESM4_ESM.zip › SENP3 TAM WB/WB-Figure4/A IRF4 SENP3 co-IP/2023-01-18 IRF4 SENP3 CO-IP/IP FLAG MYC/IP MYC SENP3 50S 0126.Tif]

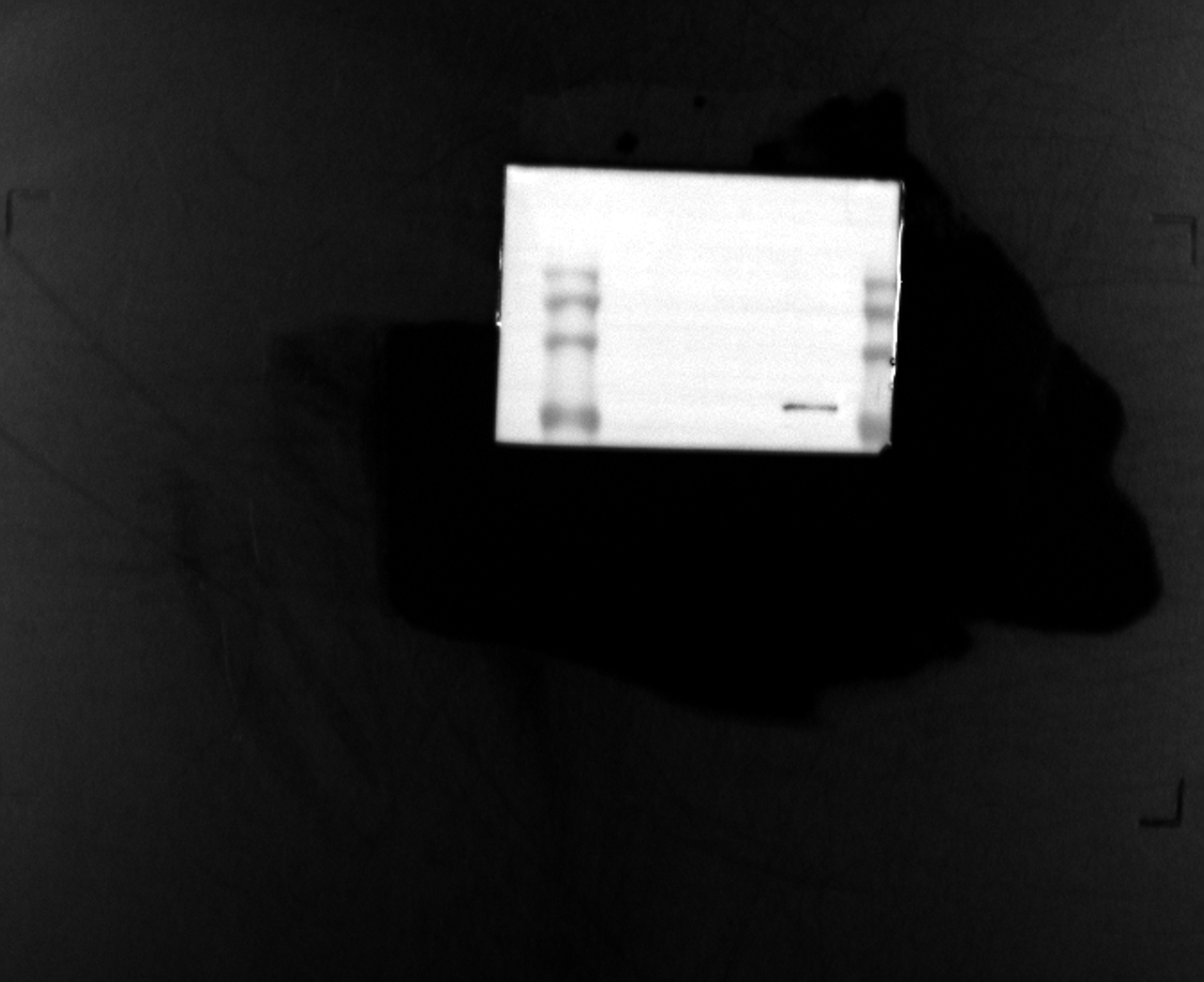

Supplement: Supplementary file 4 — Supplementary Material 4. [file 12964_2024_1770_MOESM4_ESM.zip › SENP3 TAM WB/WB-Figure4/A IRF4 SENP3 co-IP/2023-01-18 IRF4 SENP3 CO-IP/IP FLAG MYC/IP MYC SENP3 M 10S 0121.Tif]

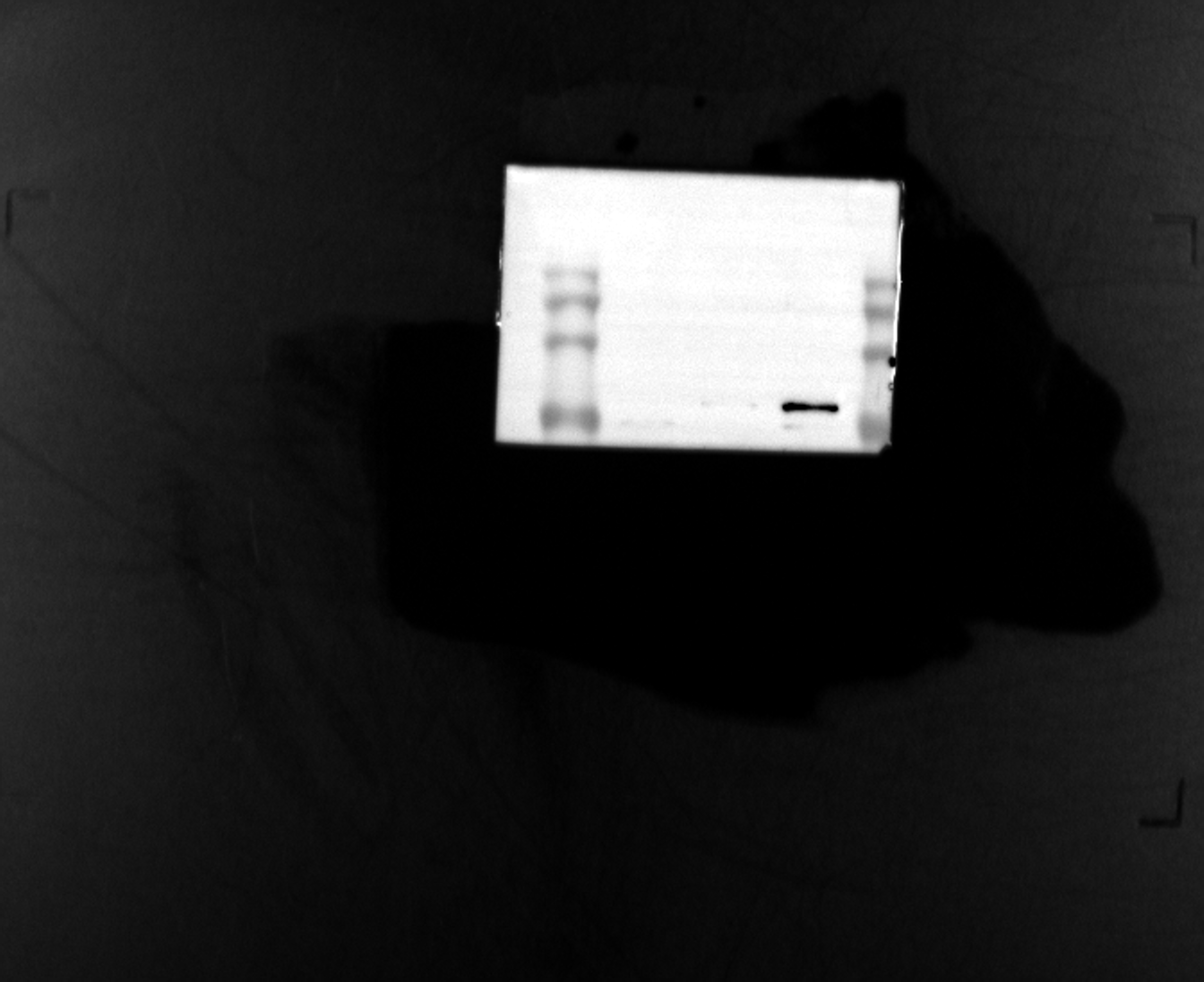

Supplement: Supplementary file 4 — Supplementary Material 4. [file 12964_2024_1770_MOESM4_ESM.zip › SENP3 TAM WB/WB-Figure4/A IRF4 SENP3 co-IP/2023-01-18 IRF4 SENP3 CO-IP/IP FLAG MYC/IP MYC SENP3 M 20S 0121.Tif]

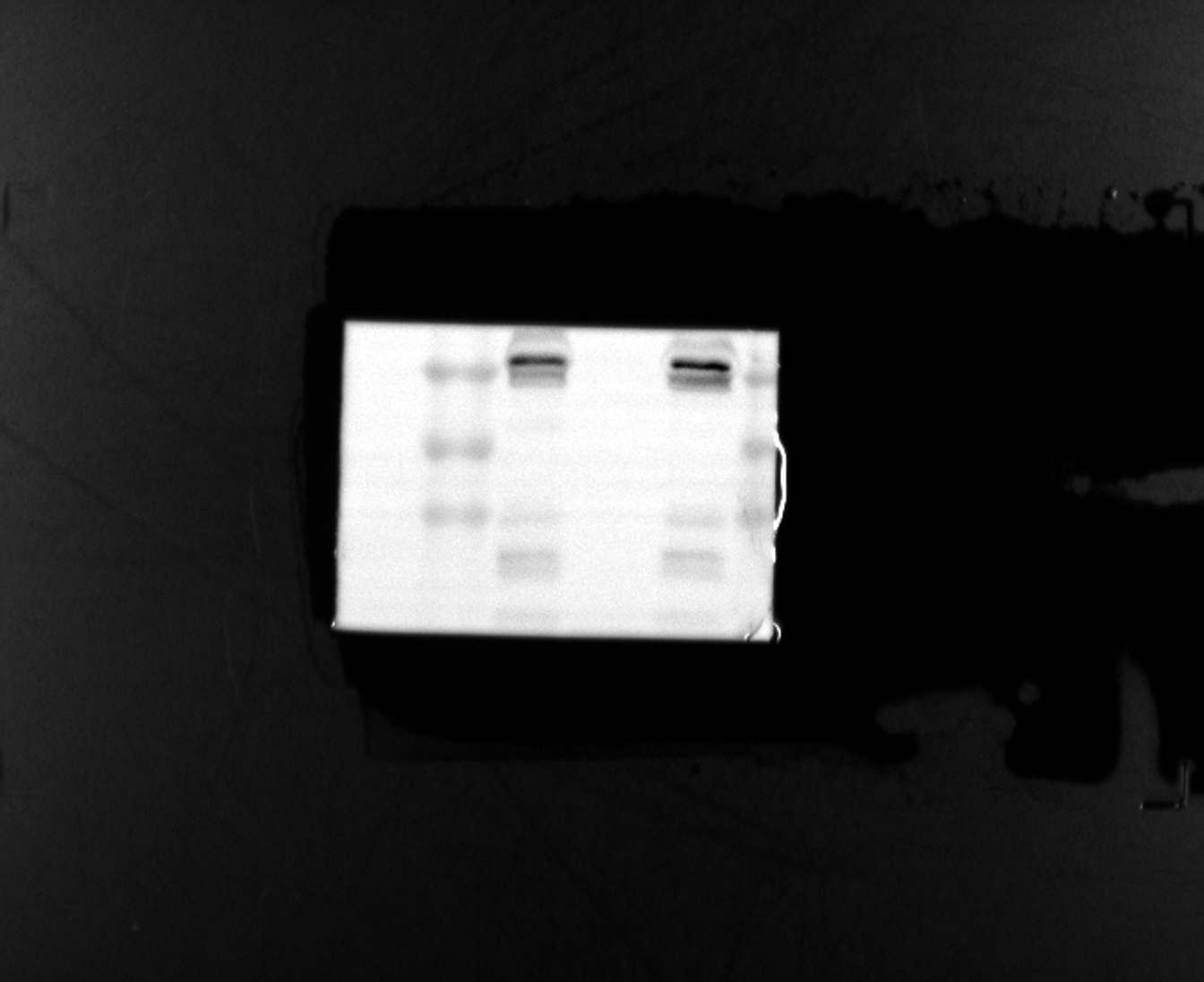

Supplement: Supplementary file 4 — Supplementary Material 4. [file 12964_2024_1770_MOESM4_ESM.zip › SENP3 TAM WB/WB-Figure4/A IRF4 SENP3 co-IP/2023-01-18 IRF4 SENP3 CO-IP/IP-FLAG IB IRF4 M 0.1S 0120.Tif]

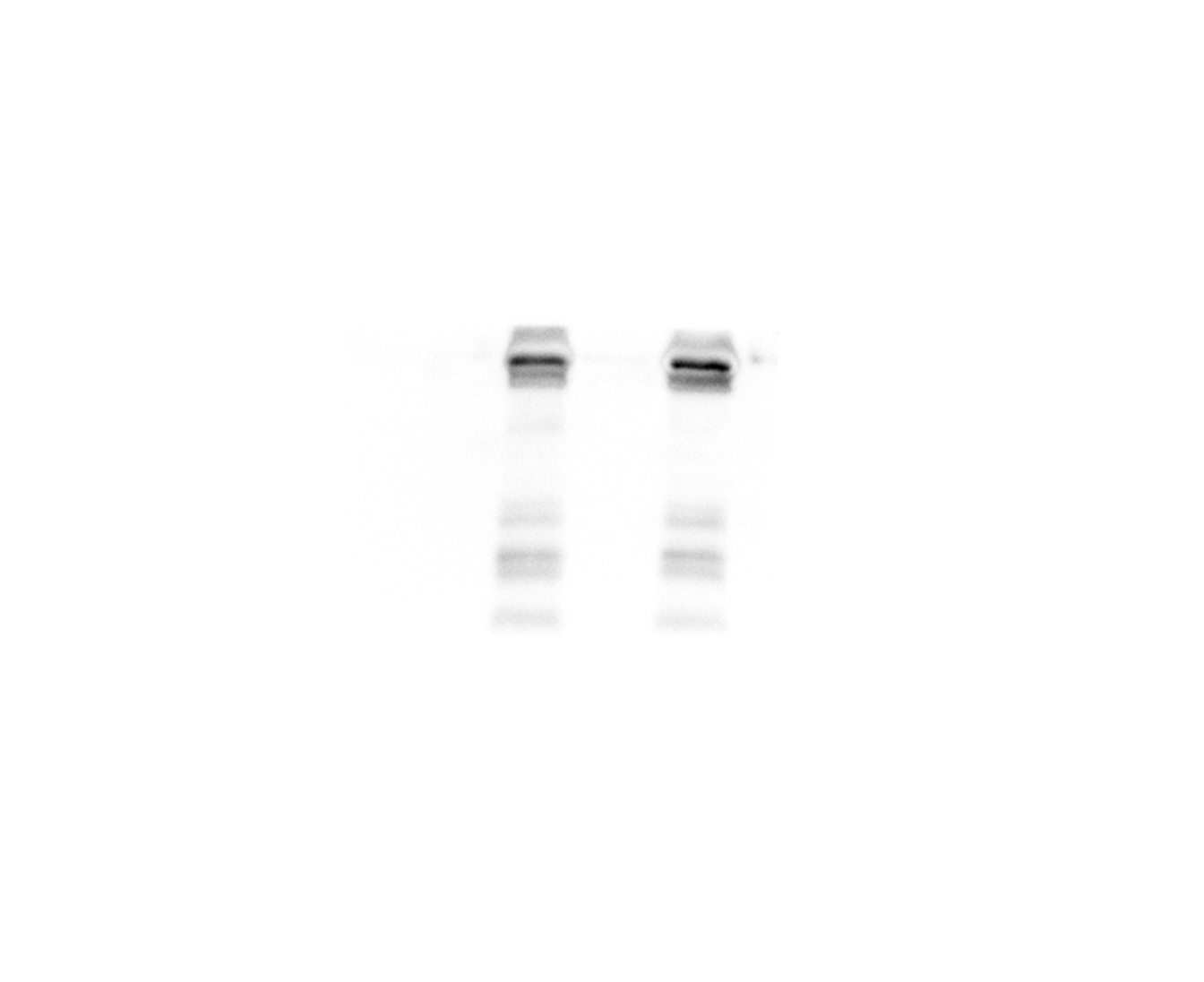

Supplement: Supplementary file 4 — Supplementary Material 4. [file 12964_2024_1770_MOESM4_ESM.zip › SENP3 TAM WB/WB-Figure4/A IRF4 SENP3 co-IP/2023-01-18 IRF4 SENP3 CO-IP/IP-FLAG IB IRF4 0.1S 0120.Tif]

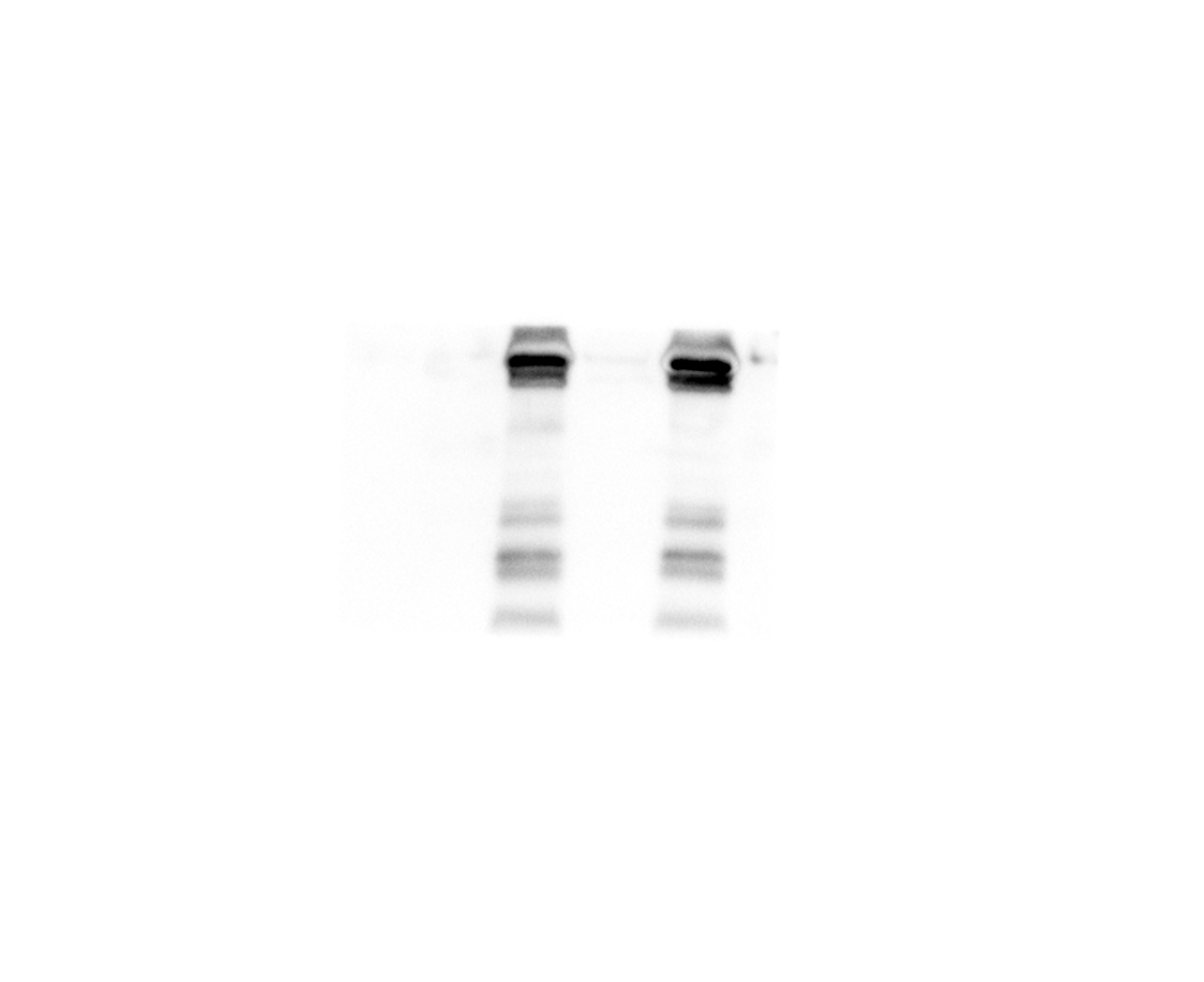

Supplement: Supplementary file 4 — Supplementary Material 4. [file 12964_2024_1770_MOESM4_ESM.zip › SENP3 TAM WB/WB-Figure4/A IRF4 SENP3 co-IP/2023-01-18 IRF4 SENP3 CO-IP/IP-FLAG IB IRF4 0.2S 0120.Tif]

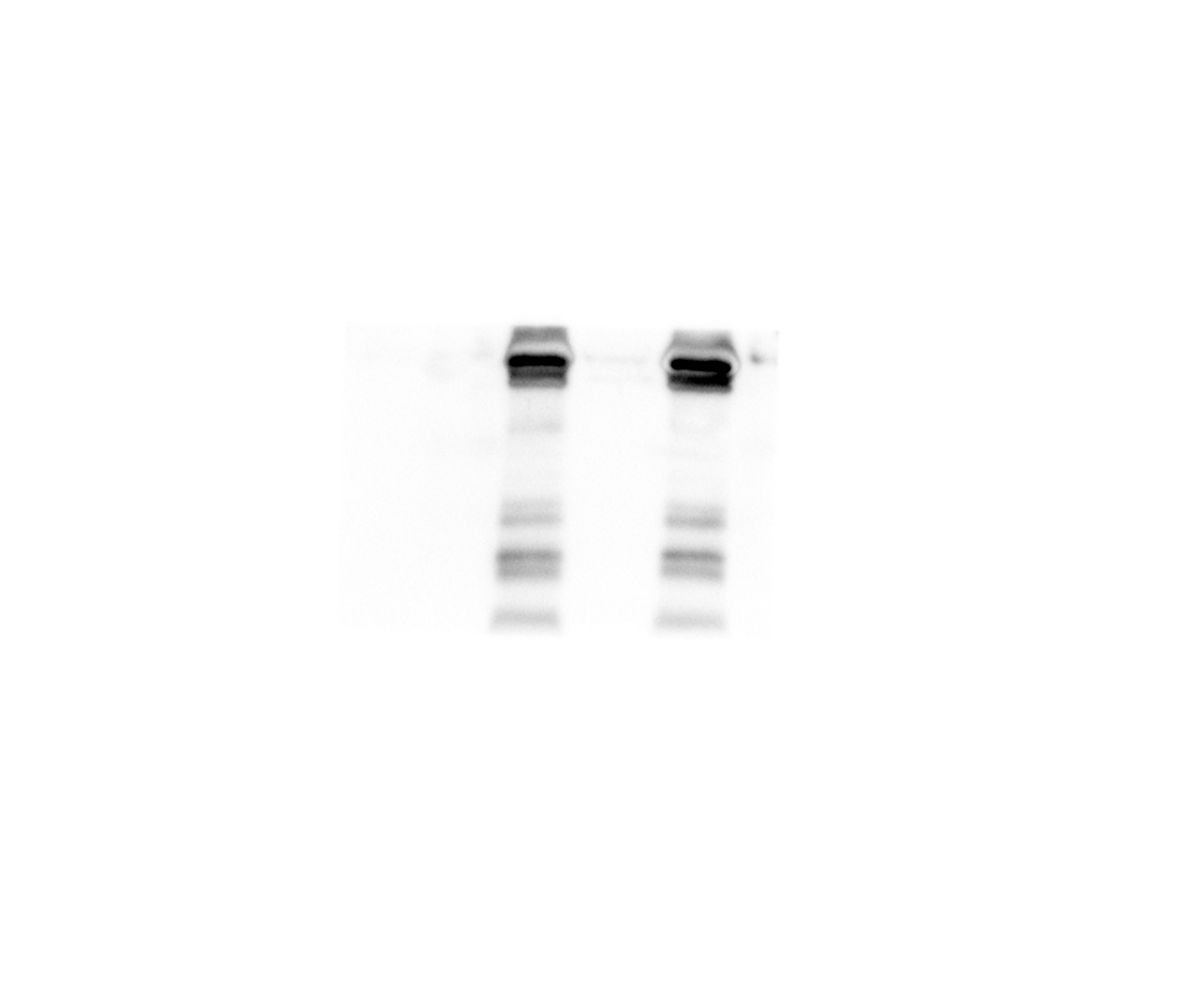

Supplement: Supplementary file 4 — Supplementary Material 4. [file 12964_2024_1770_MOESM4_ESM.zip › SENP3 TAM WB/WB-Figure4/A IRF4 SENP3 co-IP/2023-01-18 IRF4 SENP3 CO-IP/IP-FLAG IB IRF4 0.3S 0120.Tif]

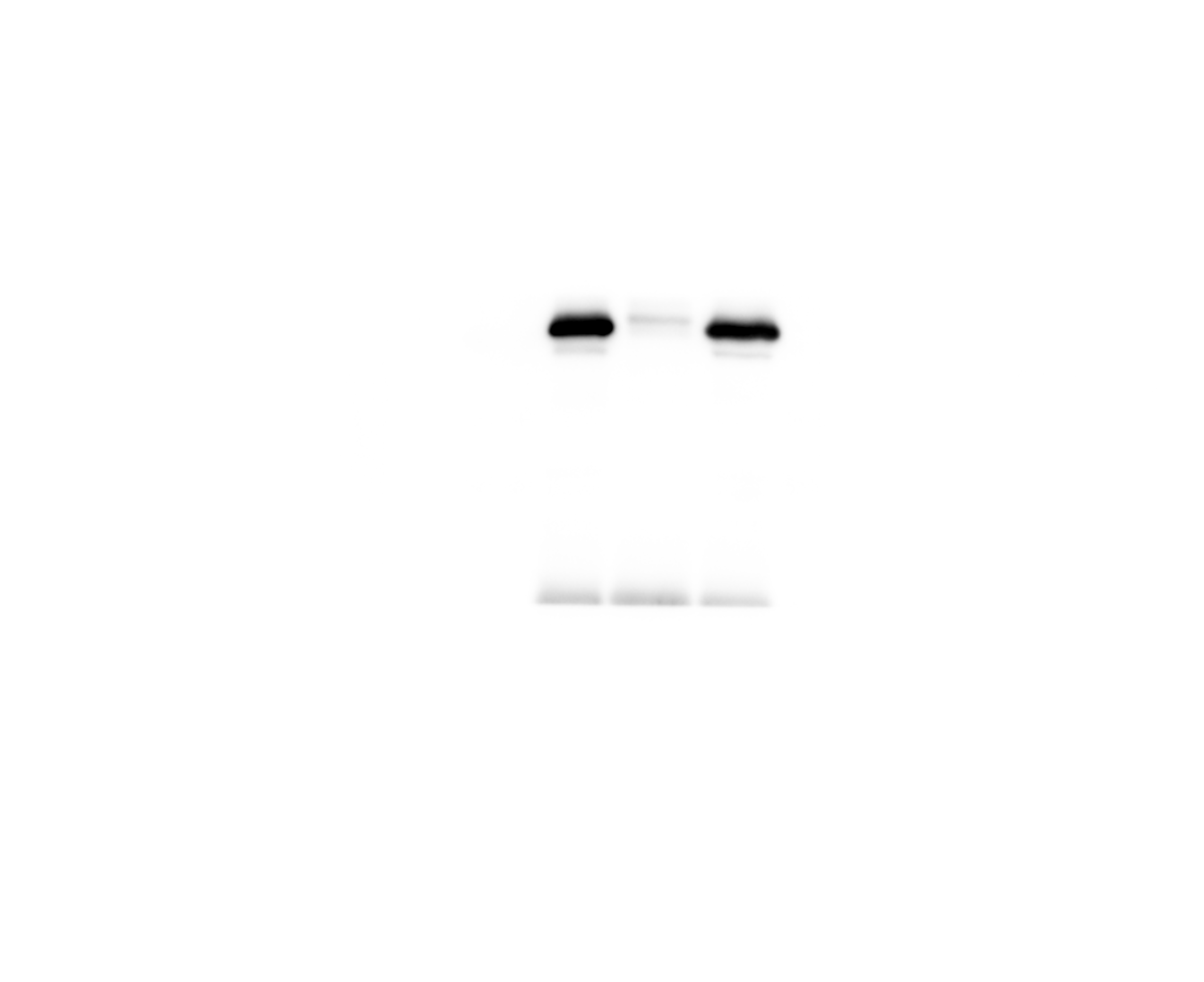

Supplement: Supplementary file 4 — Supplementary Material 4. [file 12964_2024_1770_MOESM4_ESM.zip › SENP3 TAM WB/WB-Figure4/A IRF4 SENP3 co-IP/2023-01-18 IRF4 SENP3 CO-IP/IP-FLAG IB IRF4 0.6S.Tif]

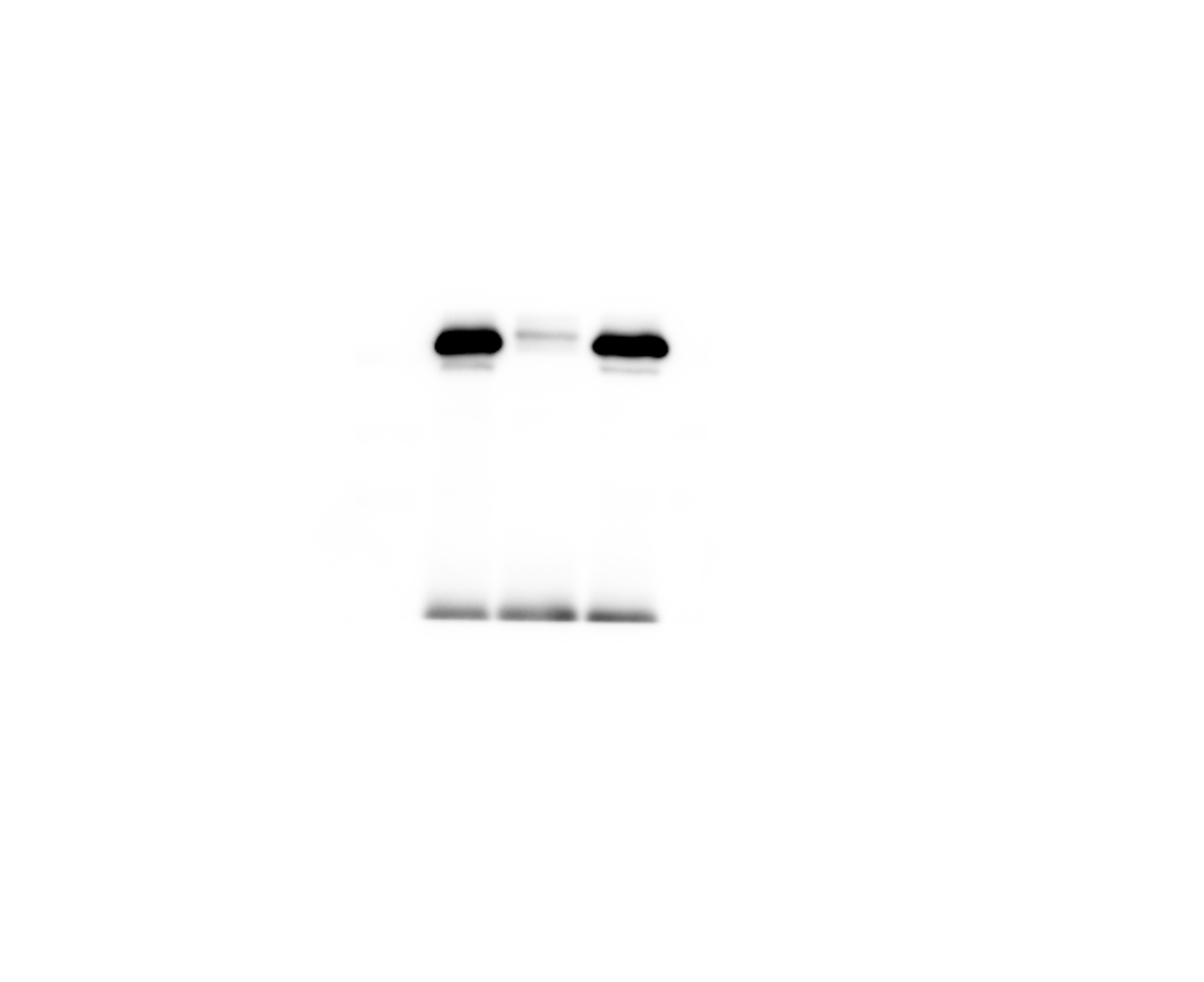

Supplement: Supplementary file 4 — Supplementary Material 4. [file 12964_2024_1770_MOESM4_ESM.zip › SENP3 TAM WB/WB-Figure4/A IRF4 SENP3 co-IP/2023-01-18 IRF4 SENP3 CO-IP/IP-FLAG IB IRF4 0.9A.Tif]

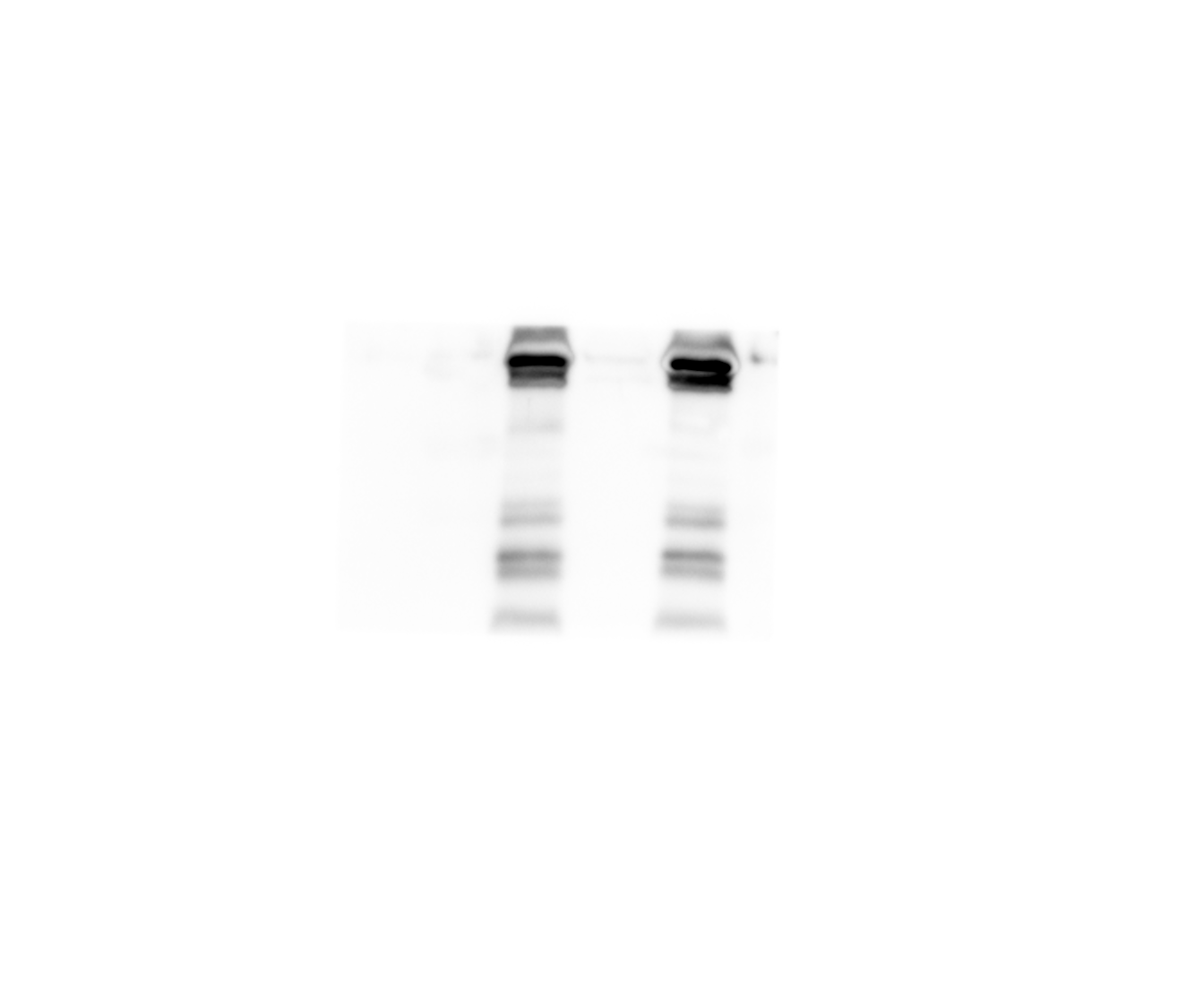

Supplement: Supplementary file 4 — Supplementary Material 4. [file 12964_2024_1770_MOESM4_ESM.zip › SENP3 TAM WB/WB-Figure4/A IRF4 SENP3 co-IP/2023-01-18 IRF4 SENP3 CO-IP/IP-FLAG IB IRF4 1.3S 0120.Tif]

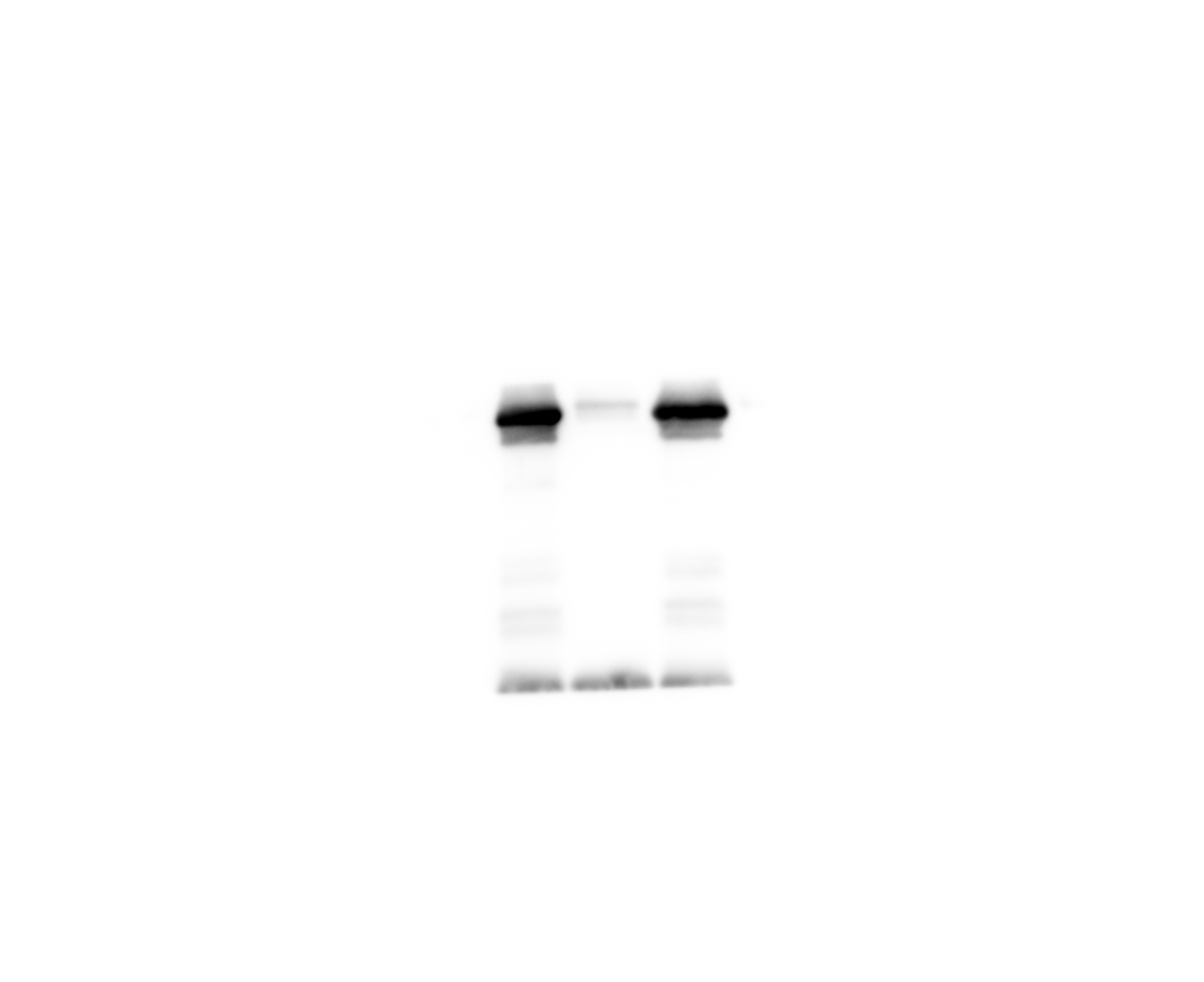

Supplement: Supplementary file 4 — Supplementary Material 4. [file 12964_2024_1770_MOESM4_ESM.zip › SENP3 TAM WB/WB-Figure4/A IRF4 SENP3 co-IP/2023-01-18 IRF4 SENP3 CO-IP/IP-FLAG IB IRF4 2 2S.Tif]

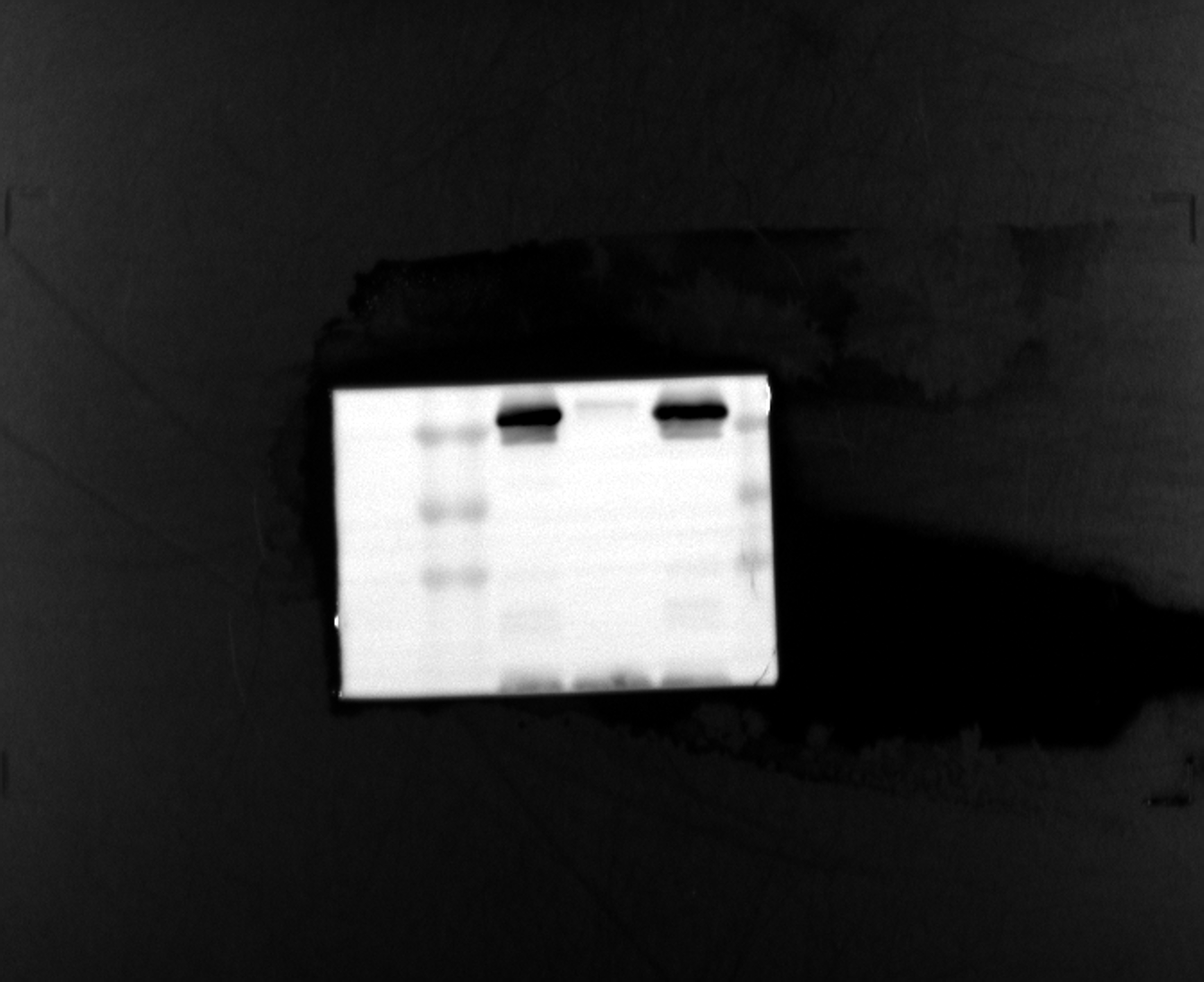

Supplement: Supplementary file 4 — Supplementary Material 4. [file 12964_2024_1770_MOESM4_ESM.zip › SENP3 TAM WB/WB-Figure4/A IRF4 SENP3 co-IP/2023-01-18 IRF4 SENP3 CO-IP/IP-FLAG IB IRF4 -2 2S.Tif]

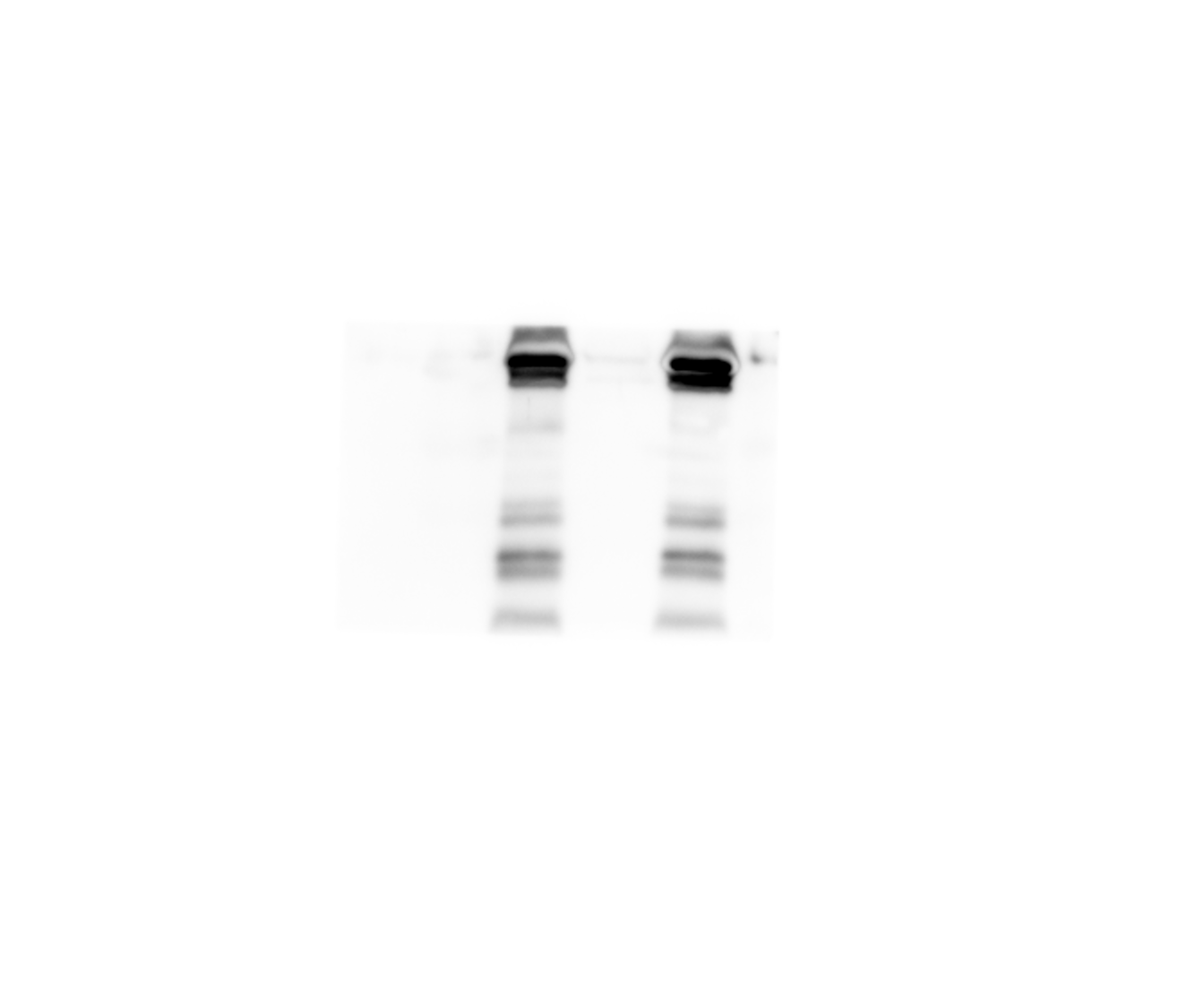

Supplement: Supplementary file 4 — Supplementary Material 4. [file 12964_2024_1770_MOESM4_ESM.zip › SENP3 TAM WB/WB-Figure4/A IRF4 SENP3 co-IP/2023-01-18 IRF4 SENP3 CO-IP/IP-FLAG IB IRF4 2.3S 0120.Tif]

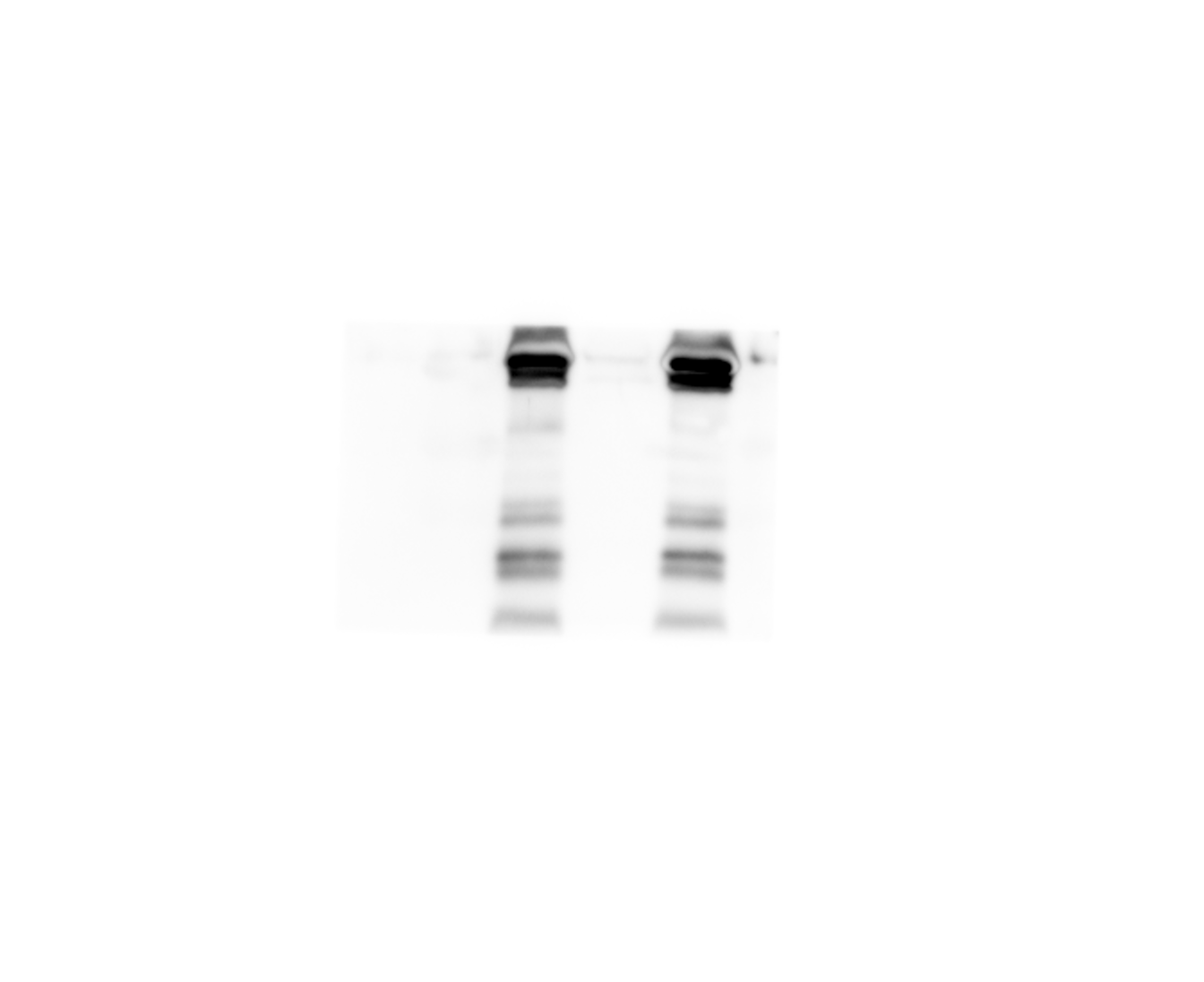

Supplement: Supplementary file 4 — Supplementary Material 4. [file 12964_2024_1770_MOESM4_ESM.zip › SENP3 TAM WB/WB-Figure4/A IRF4 SENP3 co-IP/2023-01-18 IRF4 SENP3 CO-IP/IP-FLAG IB IRF4 2.5S 0120.Tif]

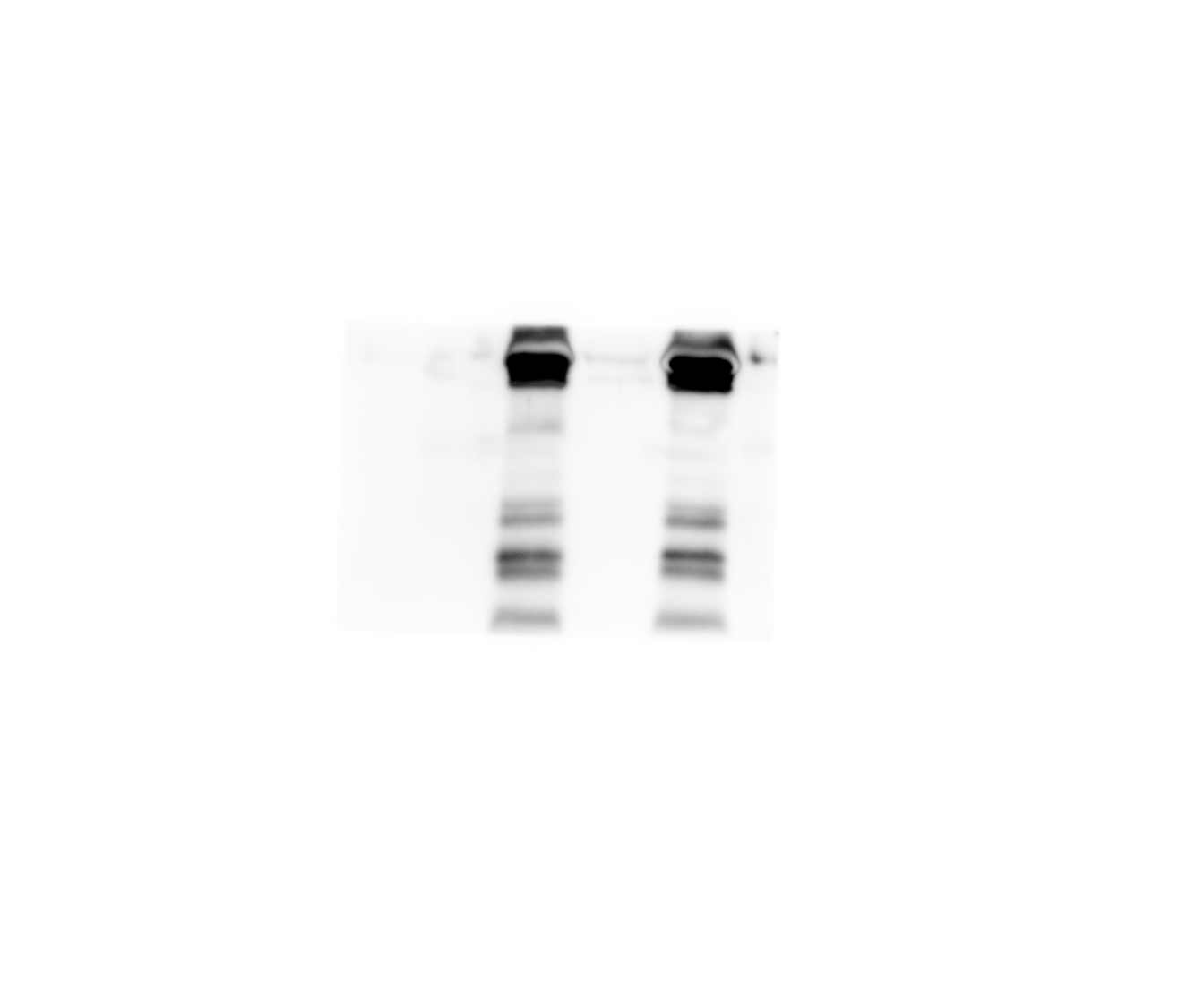

Supplement: Supplementary file 4 — Supplementary Material 4. [file 12964_2024_1770_MOESM4_ESM.zip › SENP3 TAM WB/WB-Figure4/A IRF4 SENP3 co-IP/2023-01-18 IRF4 SENP3 CO-IP/IP-FLAG IB IRF4 3.3S 0120.Tif]

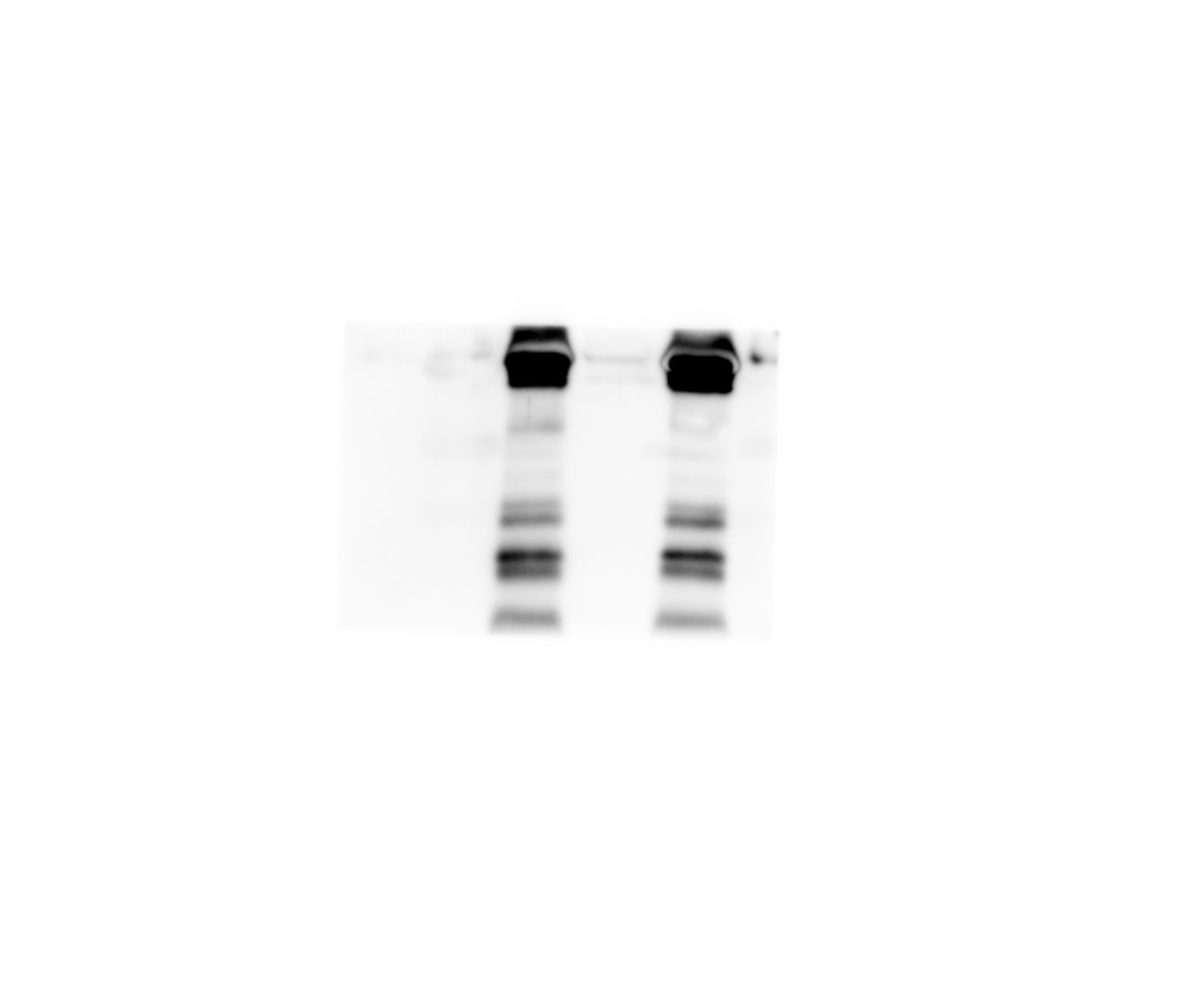

Supplement: Supplementary file 4 — Supplementary Material 4. [file 12964_2024_1770_MOESM4_ESM.zip › SENP3 TAM WB/WB-Figure4/A IRF4 SENP3 co-IP/2023-01-18 IRF4 SENP3 CO-IP/IP-FLAG IB IRF4 4.3S 0120.Tif]

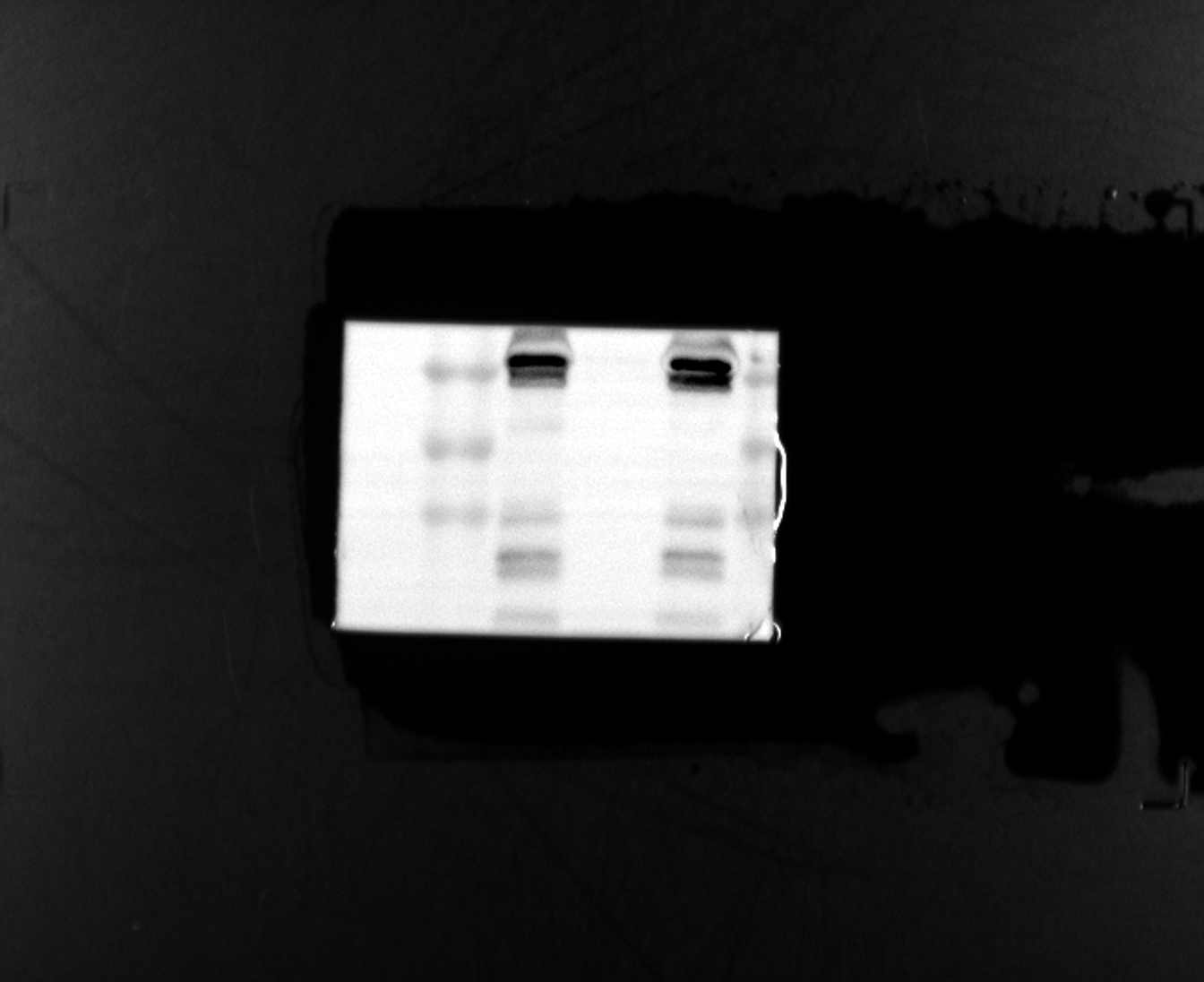

Supplement: Supplementary file 4 — Supplementary Material 4. [file 12964_2024_1770_MOESM4_ESM.zip › SENP3 TAM WB/WB-Figure4/A IRF4 SENP3 co-IP/2023-01-18 IRF4 SENP3 CO-IP/IP-FLAG IB IRF4 M 0.2S 0120.Tif]

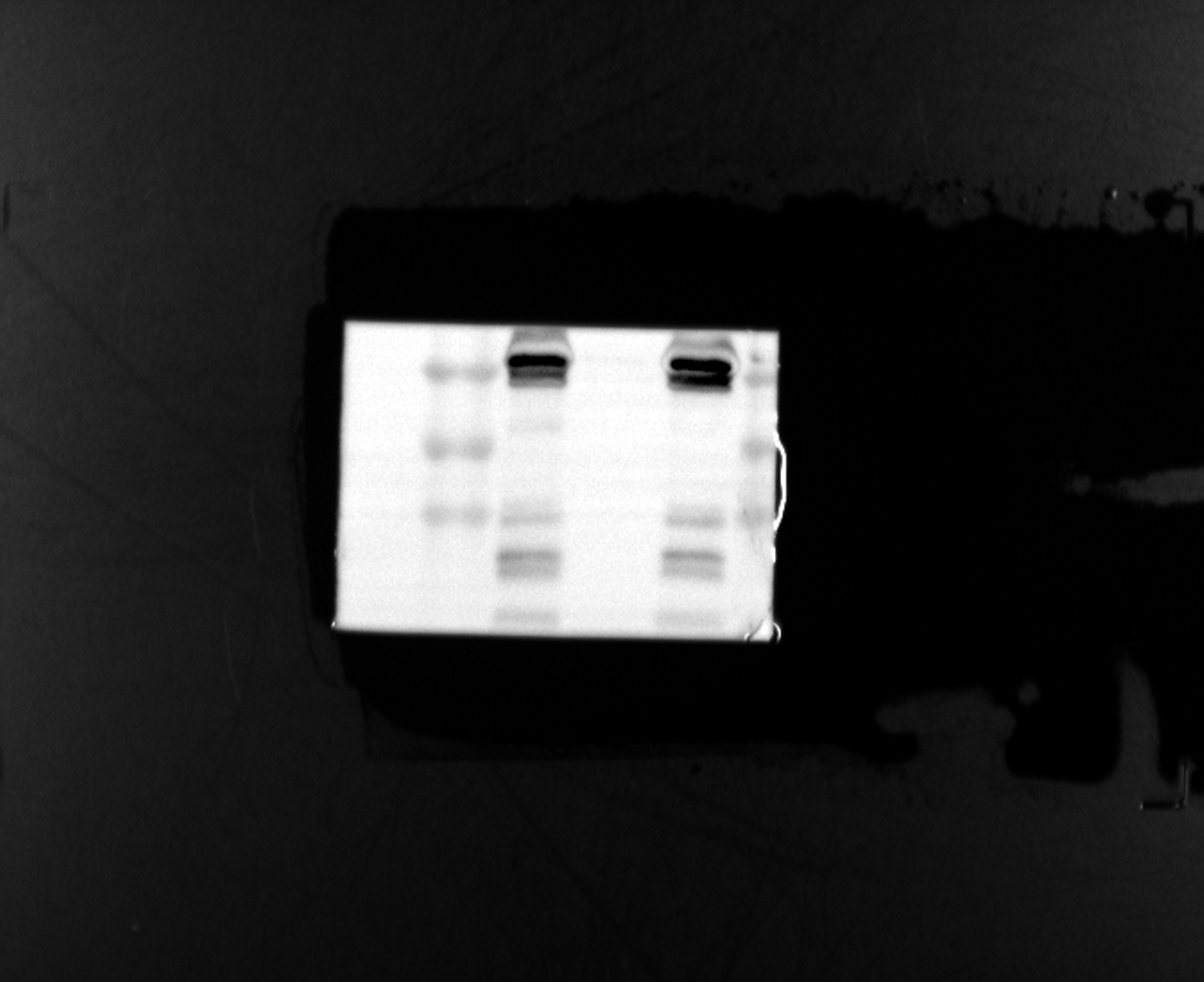

Supplement: Supplementary file 4 — Supplementary Material 4. [file 12964_2024_1770_MOESM4_ESM.zip › SENP3 TAM WB/WB-Figure4/A IRF4 SENP3 co-IP/2023-01-18 IRF4 SENP3 CO-IP/IP-FLAG IB IRF4 M 0.3S 0120.Tif]

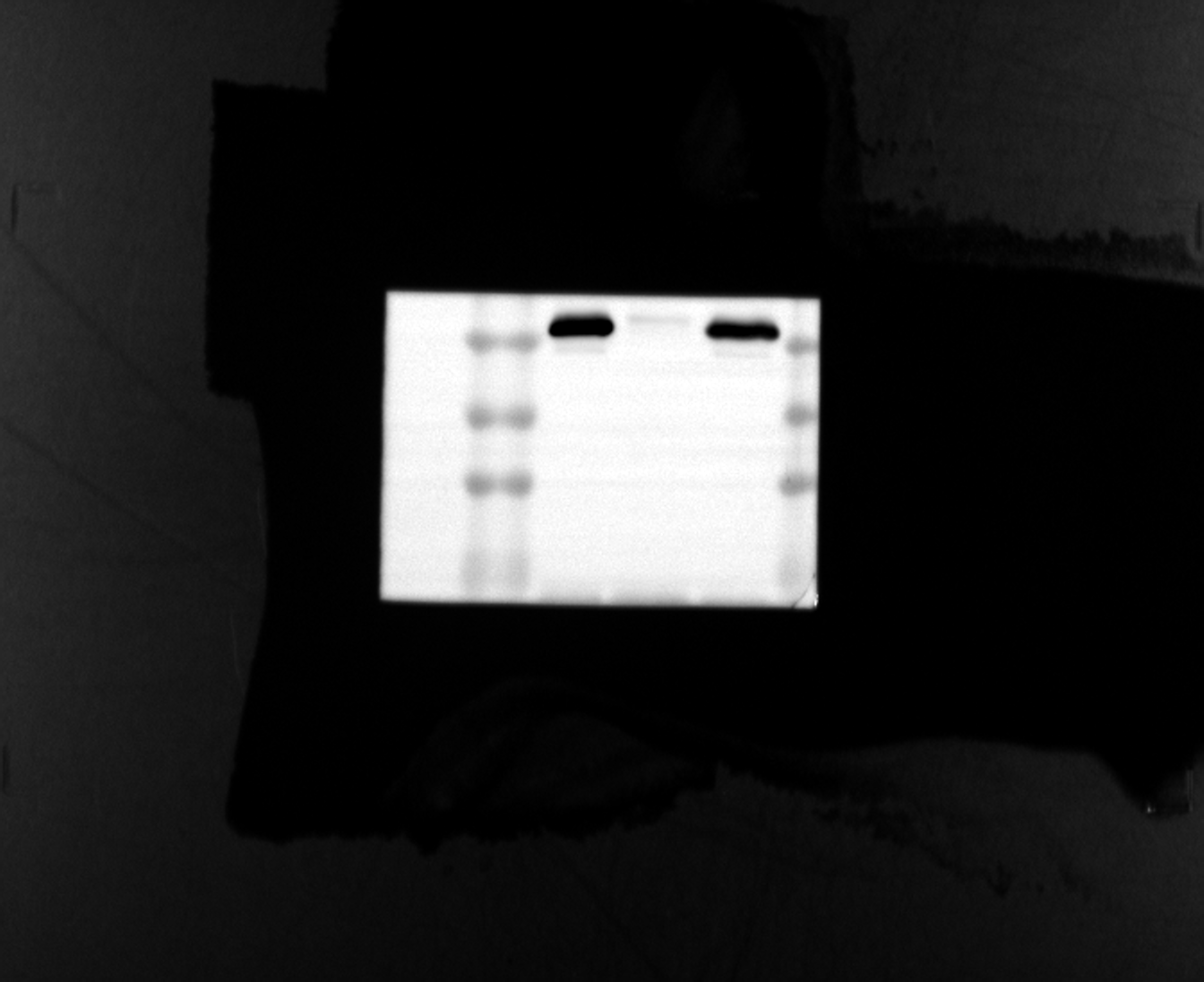

Supplement: Supplementary file 4 — Supplementary Material 4. [file 12964_2024_1770_MOESM4_ESM.zip › SENP3 TAM WB/WB-Figure4/A IRF4 SENP3 co-IP/2023-01-18 IRF4 SENP3 CO-IP/IP-FLAG IB IRF4 m 0.6S.Tif]

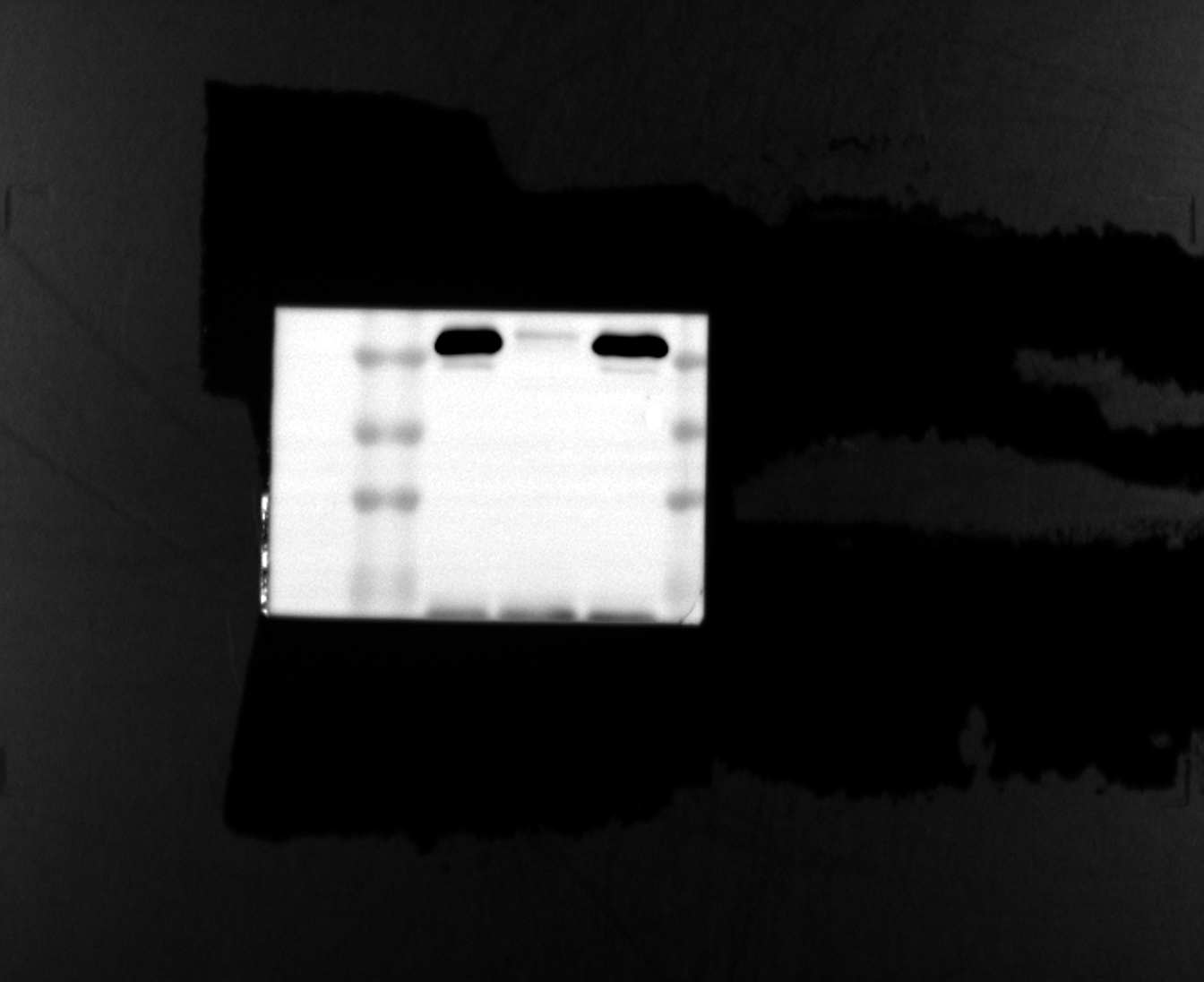

Supplement: Supplementary file 4 — Supplementary Material 4. [file 12964_2024_1770_MOESM4_ESM.zip › SENP3 TAM WB/WB-Figure4/A IRF4 SENP3 co-IP/2023-01-18 IRF4 SENP3 CO-IP/IP-FLAG IB IRF4 m 0.9S.Tif]

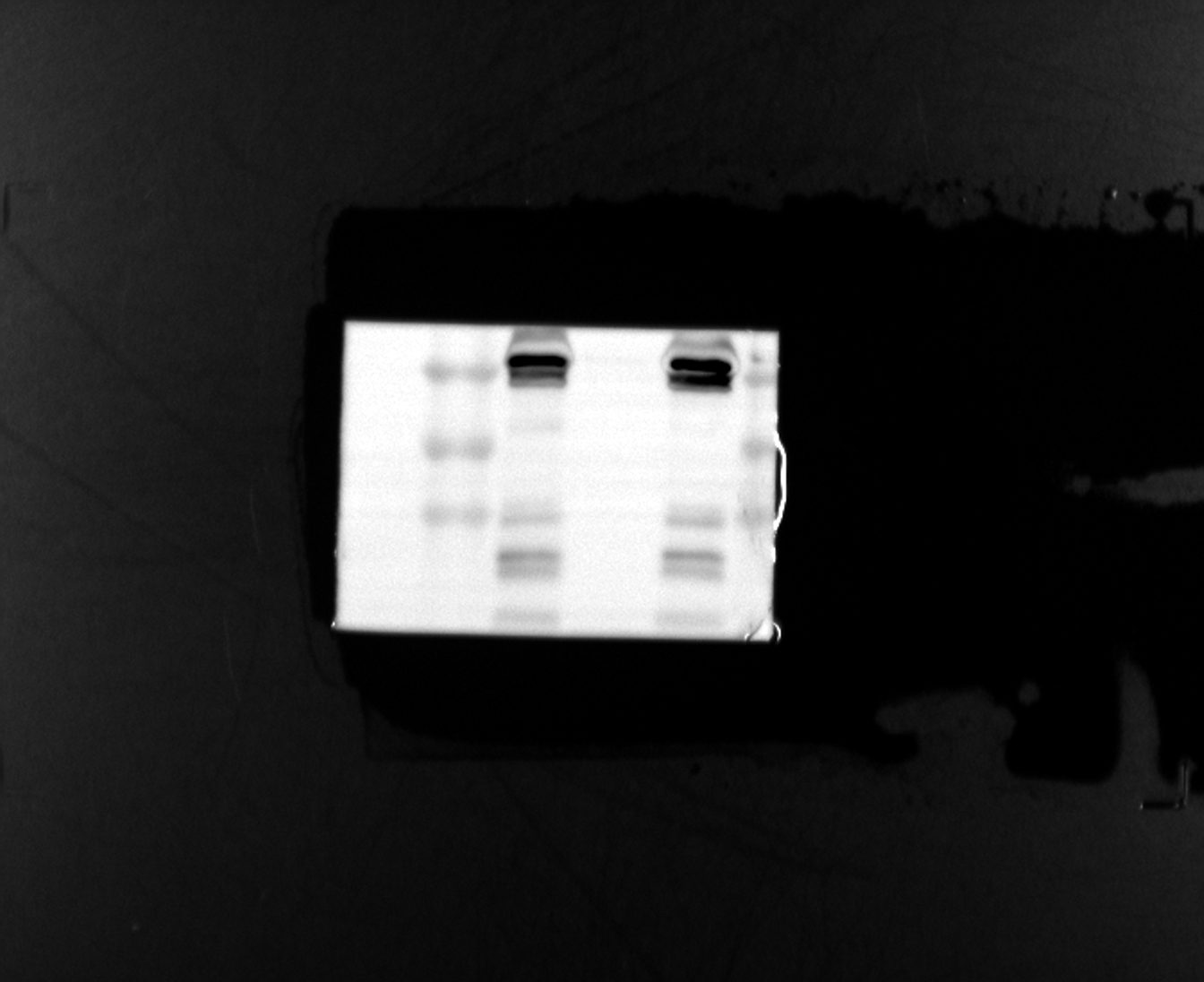

Supplement: Supplementary file 4 — Supplementary Material 4. [file 12964_2024_1770_MOESM4_ESM.zip › SENP3 TAM WB/WB-Figure4/A IRF4 SENP3 co-IP/2023-01-18 IRF4 SENP3 CO-IP/IP-FLAG IB IRF4 M 1.3S 0120.Tif]

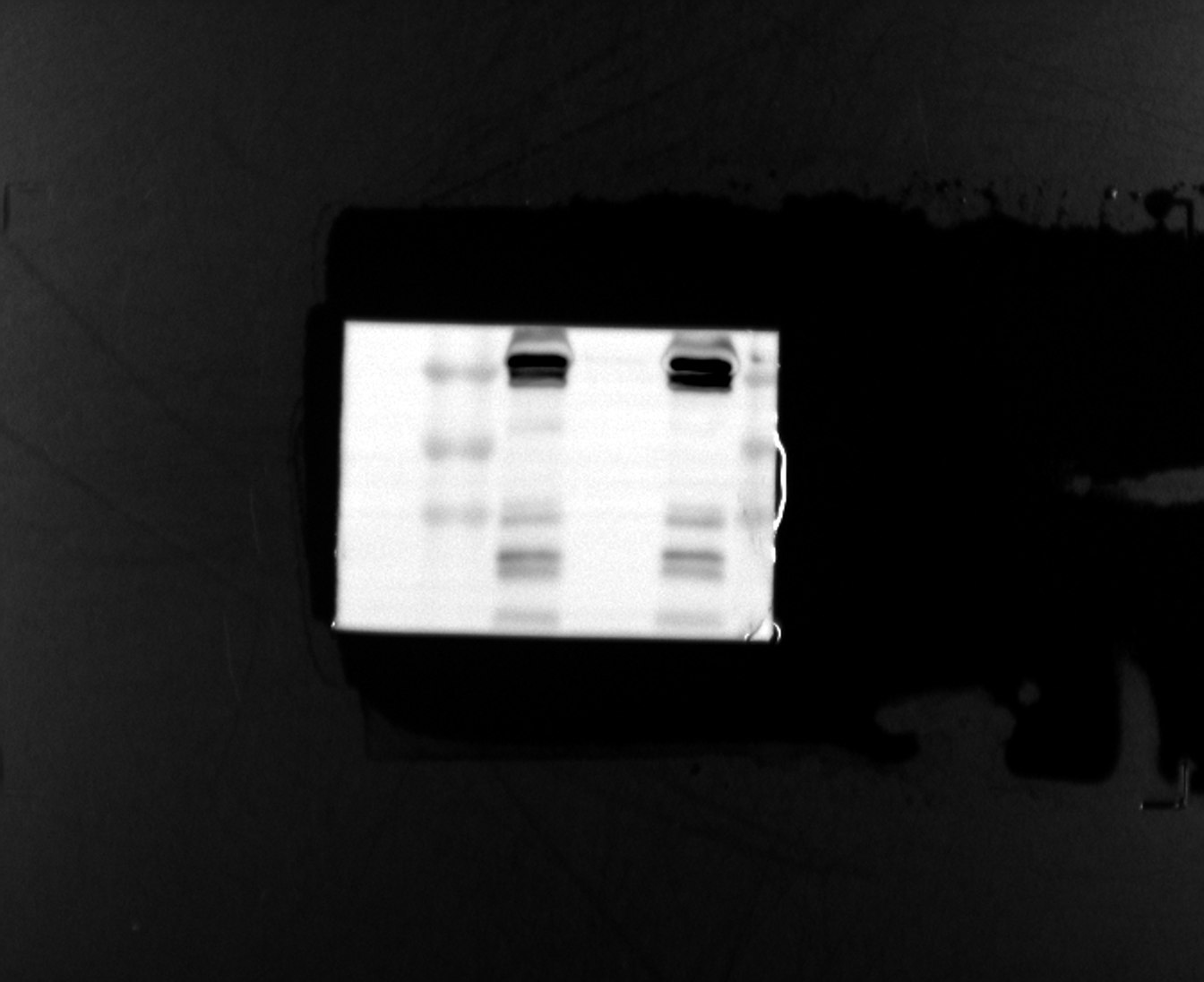

Supplement: Supplementary file 4 — Supplementary Material 4. [file 12964_2024_1770_MOESM4_ESM.zip › SENP3 TAM WB/WB-Figure4/A IRF4 SENP3 co-IP/2023-01-18 IRF4 SENP3 CO-IP/IP-FLAG IB IRF4 M 2.3S 0120.Tif]

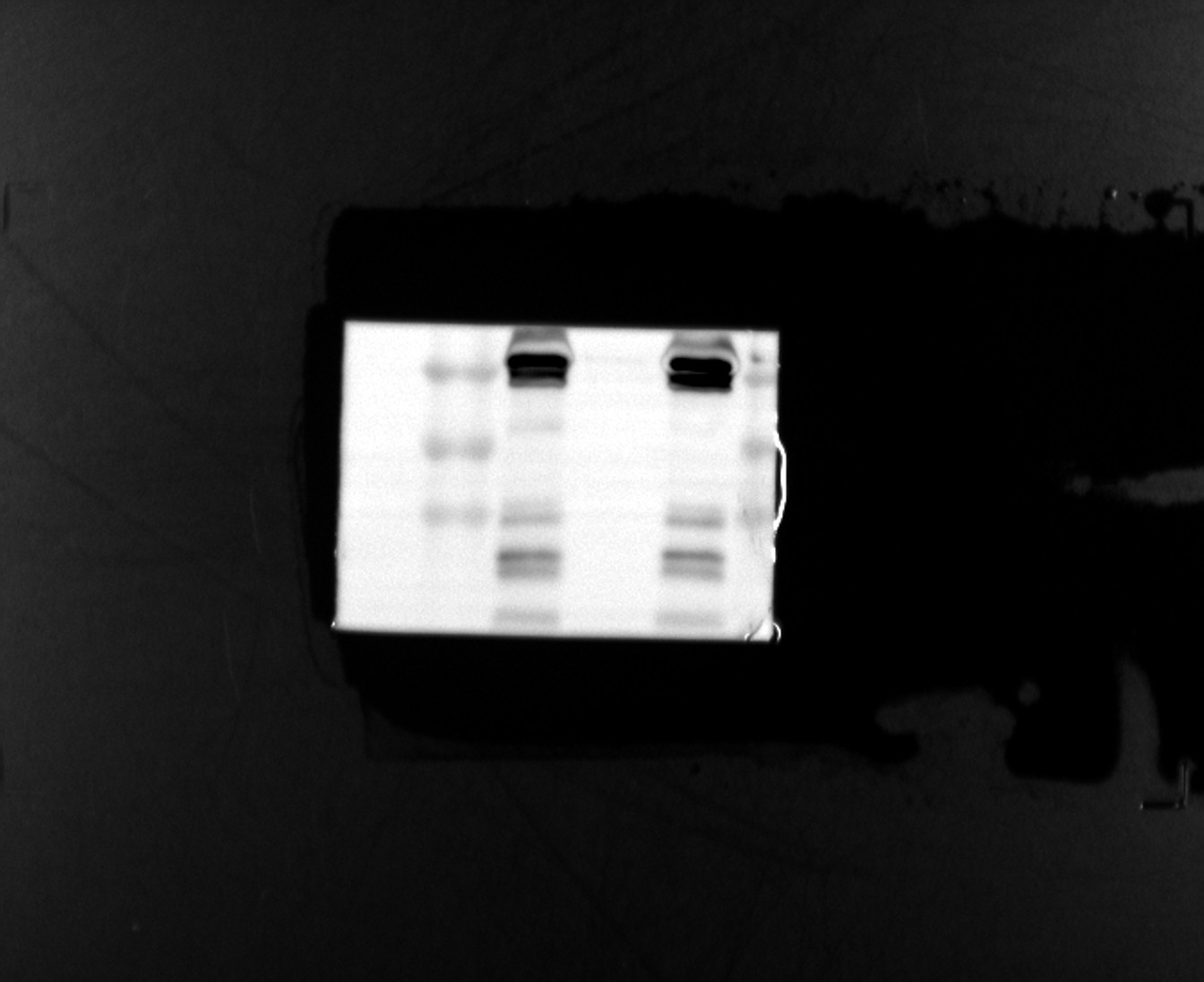

Supplement: Supplementary file 4 — Supplementary Material 4. [file 12964_2024_1770_MOESM4_ESM.zip › SENP3 TAM WB/WB-Figure4/A IRF4 SENP3 co-IP/2023-01-18 IRF4 SENP3 CO-IP/IP-FLAG IB IRF4 M 2.5S 0120.Tif]

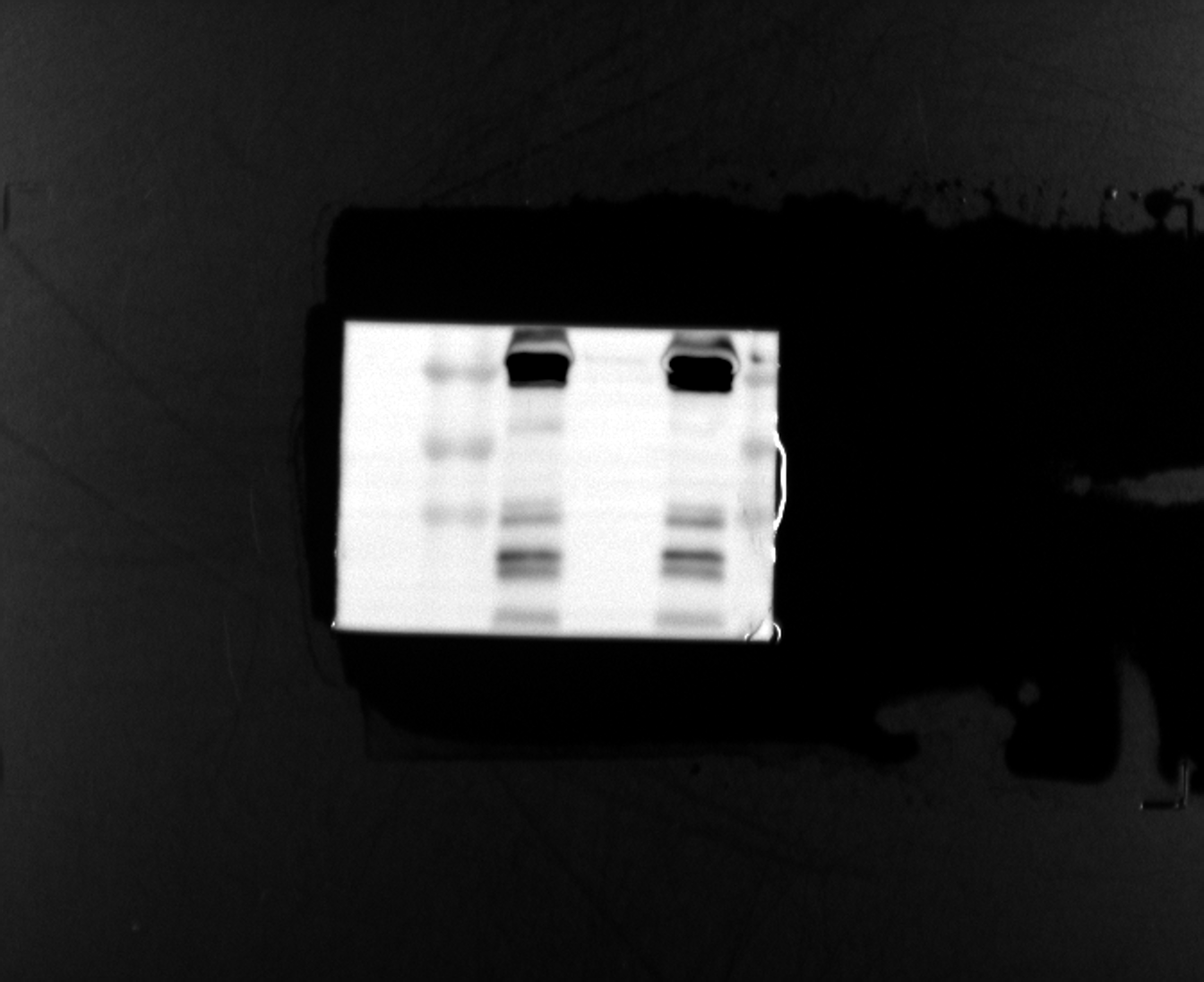

Supplement: Supplementary file 4 — Supplementary Material 4. [file 12964_2024_1770_MOESM4_ESM.zip › SENP3 TAM WB/WB-Figure4/A IRF4 SENP3 co-IP/2023-01-18 IRF4 SENP3 CO-IP/IP-FLAG IB IRF4 M 3.3S 0120.Tif]

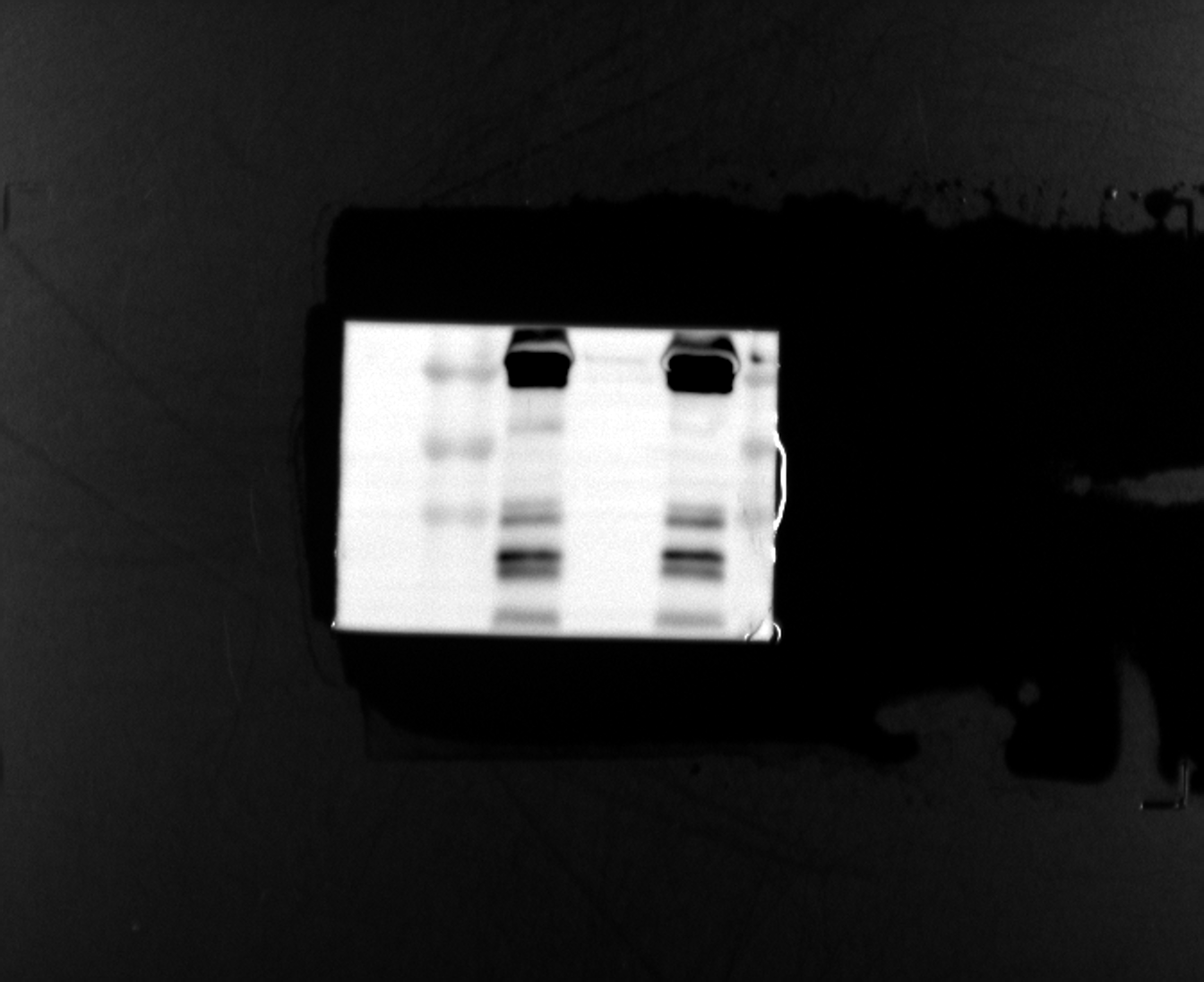

Supplement: Supplementary file 4 — Supplementary Material 4. [file 12964_2024_1770_MOESM4_ESM.zip › SENP3 TAM WB/WB-Figure4/A IRF4 SENP3 co-IP/2023-01-18 IRF4 SENP3 CO-IP/IP-FLAG IB IRF4 M 4.3S 0120.Tif]

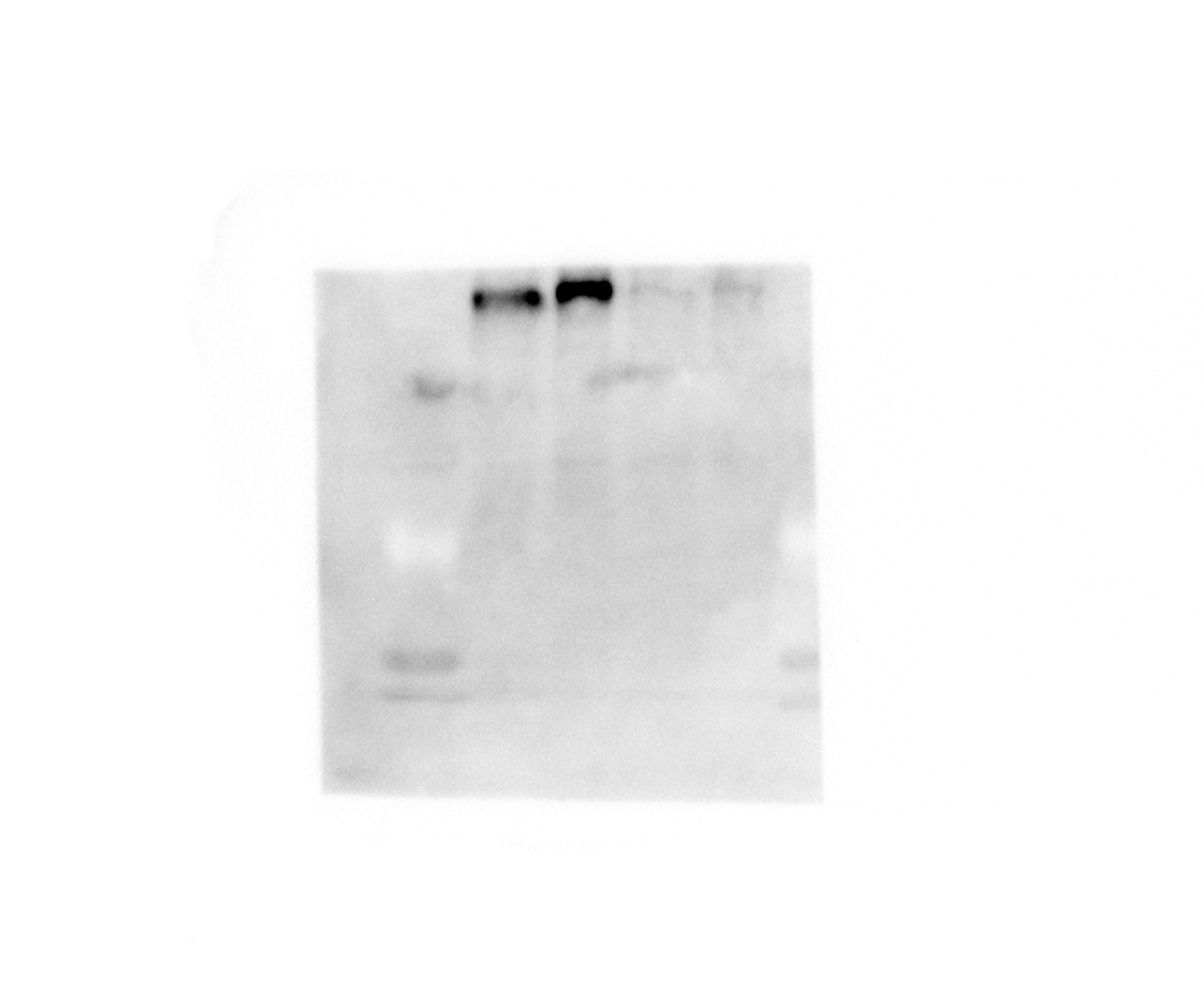

Supplement: Supplementary file 4 — Supplementary Material 4. [file 12964_2024_1770_MOESM4_ESM.zip › SENP3 TAM WB/WB-Figure4/B M0 M2 EndoIP/2023-02-14 ─┌╘┤IP shNC shSENP3 IRF4/INPUT IRF4/INPUT IRF4 10S 0124.Tif]

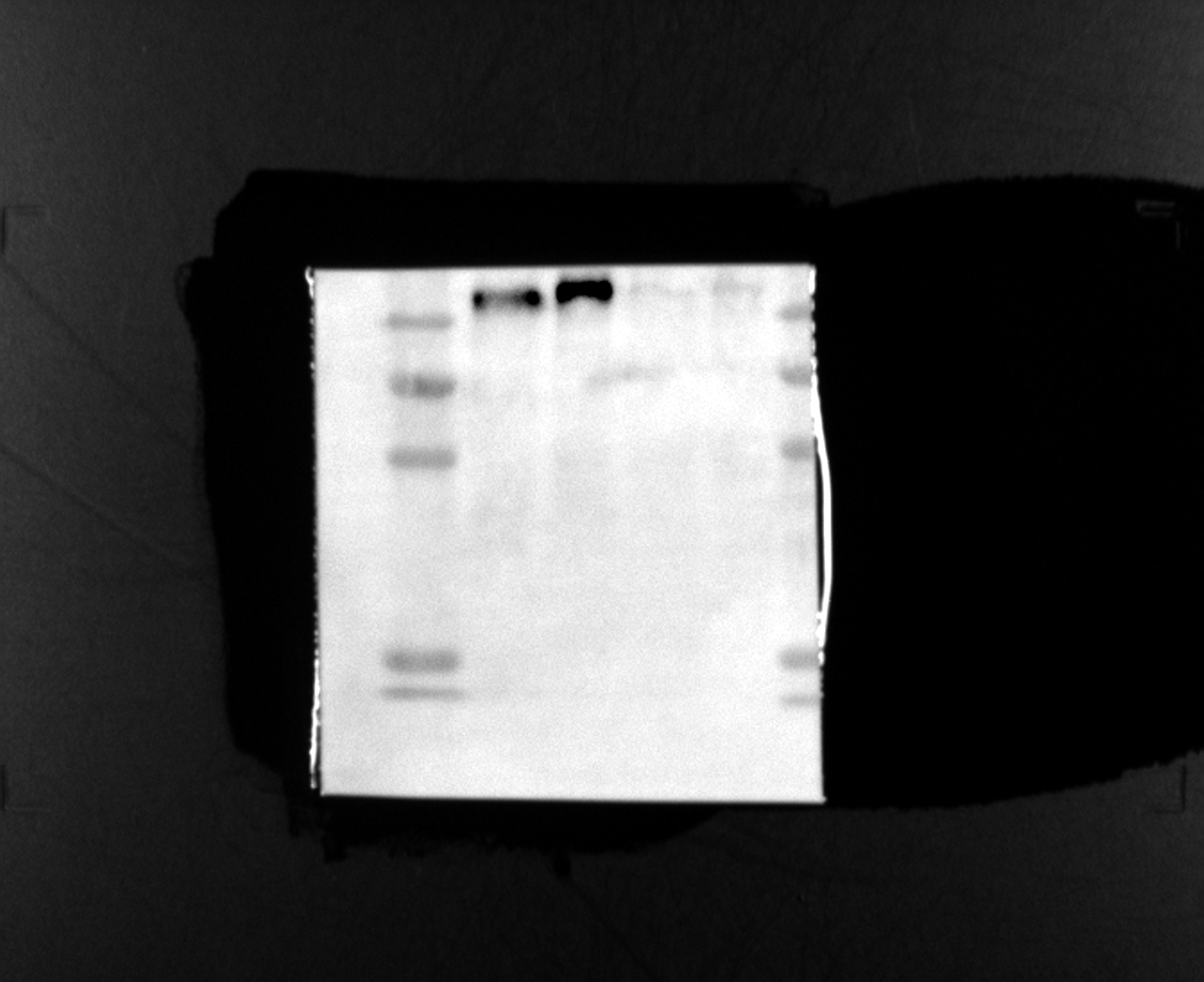

Supplement: Supplementary file 4 — Supplementary Material 4. [file 12964_2024_1770_MOESM4_ESM.zip › SENP3 TAM WB/WB-Figure4/B M0 M2 EndoIP/2023-02-14 ─┌╘┤IP shNC shSENP3 IRF4/INPUT IRF4/INPUT IRF4 10S M 0124.Tif]

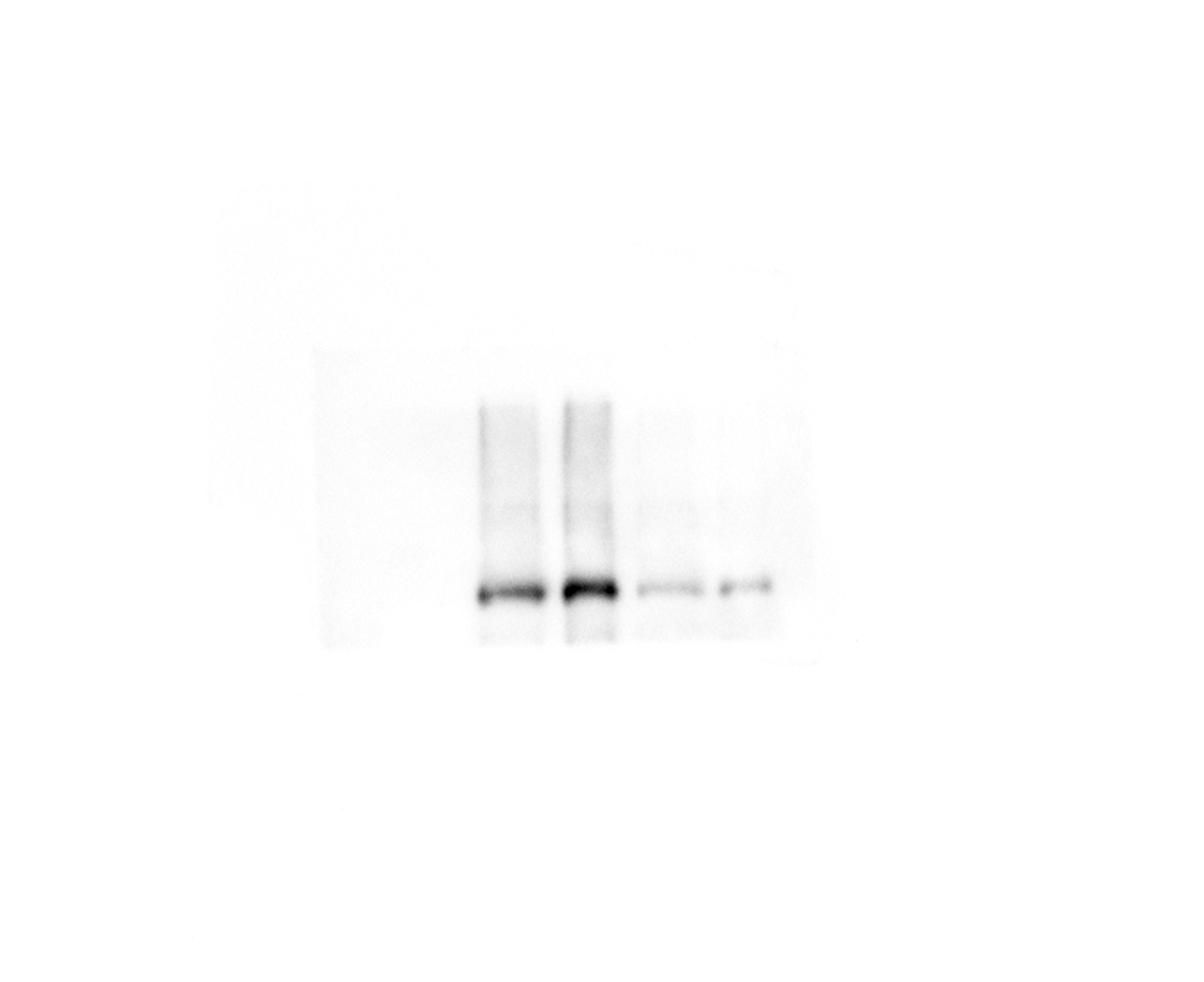

Supplement: Supplementary file 4 — Supplementary Material 4. [file 12964_2024_1770_MOESM4_ESM.zip › SENP3 TAM WB/WB-Figure4/B M0 M2 EndoIP/2023-02-14 ─┌╘┤IP shNC shSENP3 IRF4/INPUT SENP3/INPUT SENP3 10S 0214.Tif]

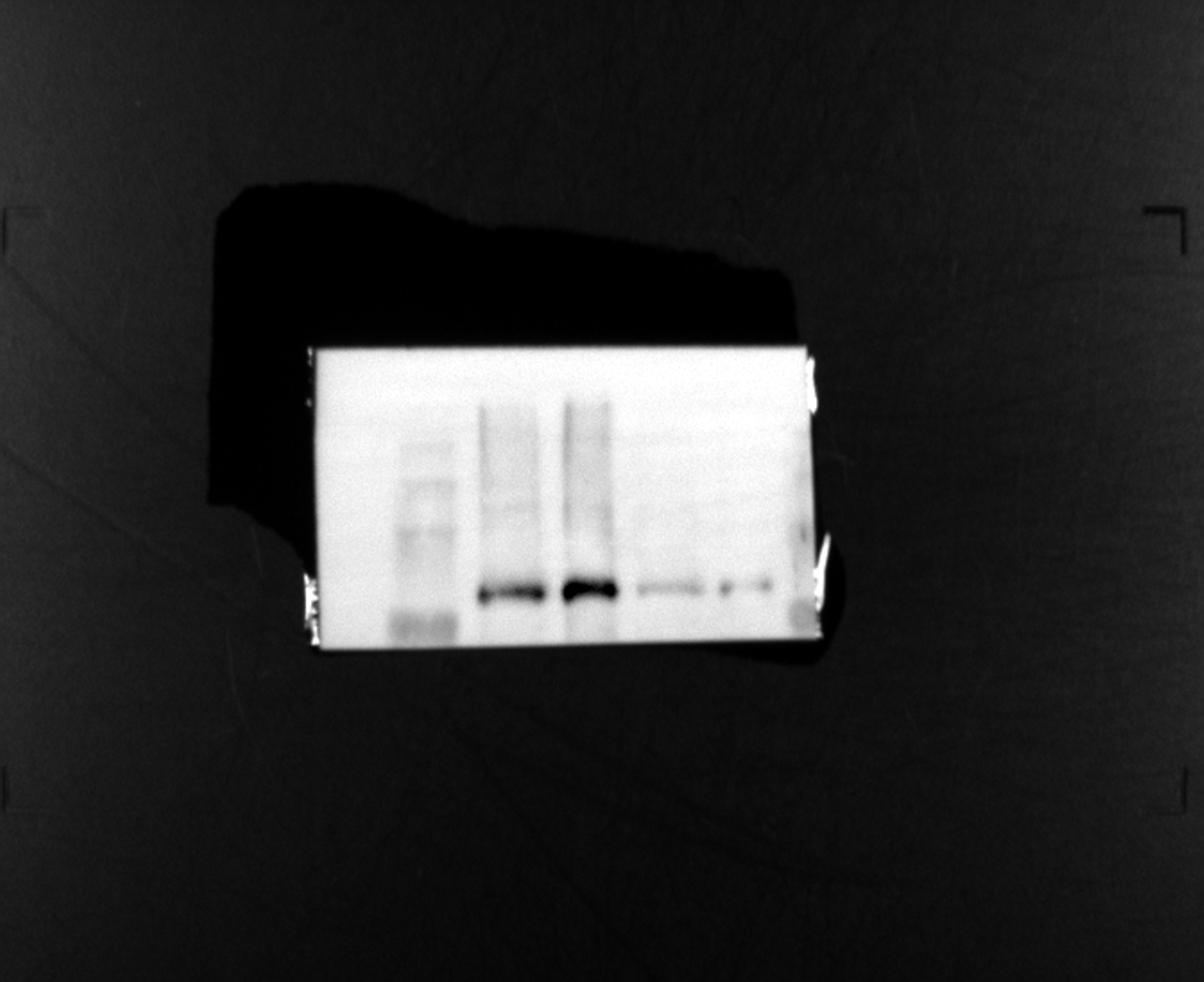

Supplement: Supplementary file 4 — Supplementary Material 4. [file 12964_2024_1770_MOESM4_ESM.zip › SENP3 TAM WB/WB-Figure4/B M0 M2 EndoIP/2023-02-14 ─┌╘┤IP shNC shSENP3 IRF4/INPUT SENP3/INPUT SENP3 10S M 0214.Tif]

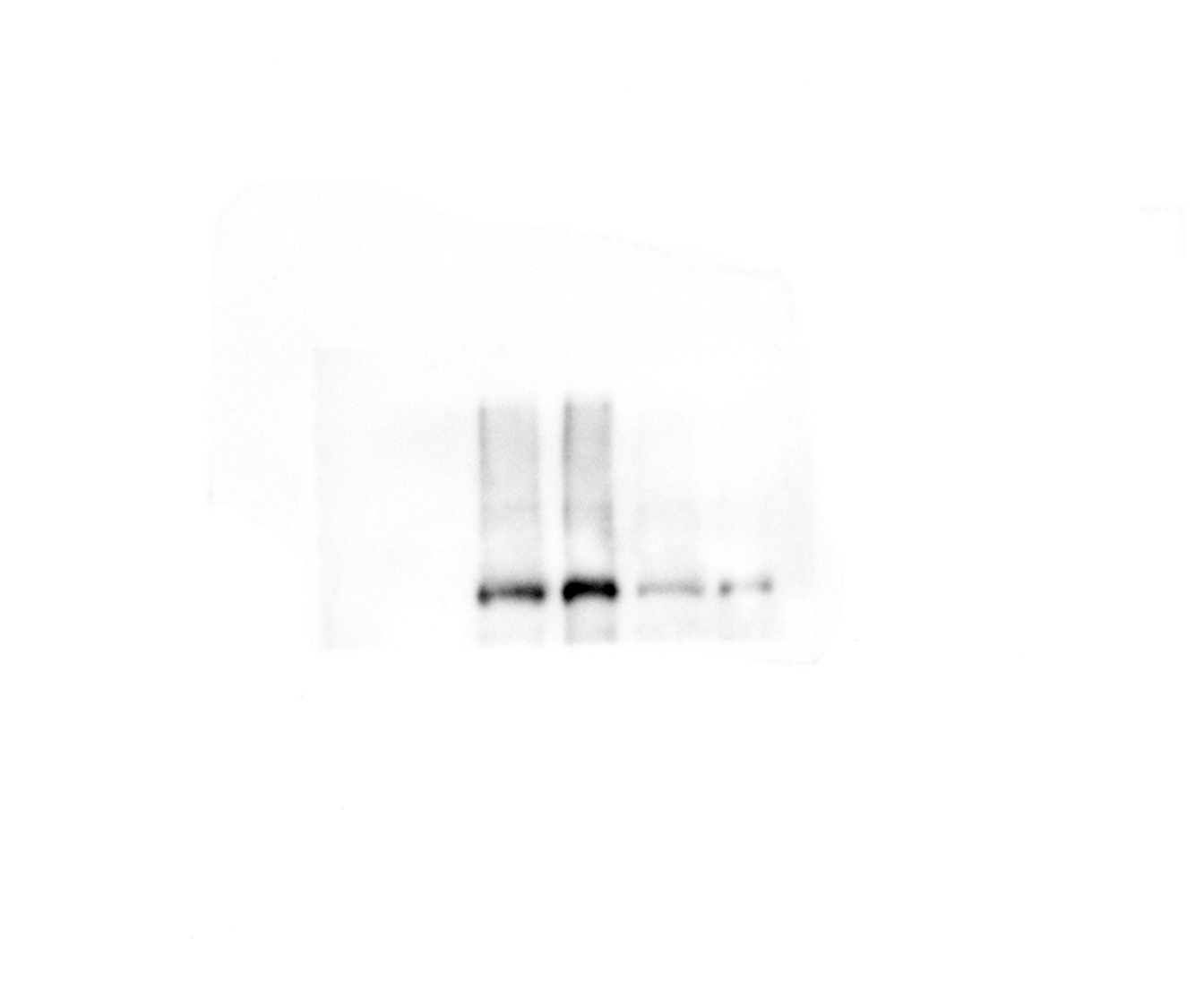

Supplement: Supplementary file 4 — Supplementary Material 4. [file 12964_2024_1770_MOESM4_ESM.zip › SENP3 TAM WB/WB-Figure4/B M0 M2 EndoIP/2023-02-14 ─┌╘┤IP shNC shSENP3 IRF4/INPUT SENP3/INPUT SENP3 15S 0214.Tif]

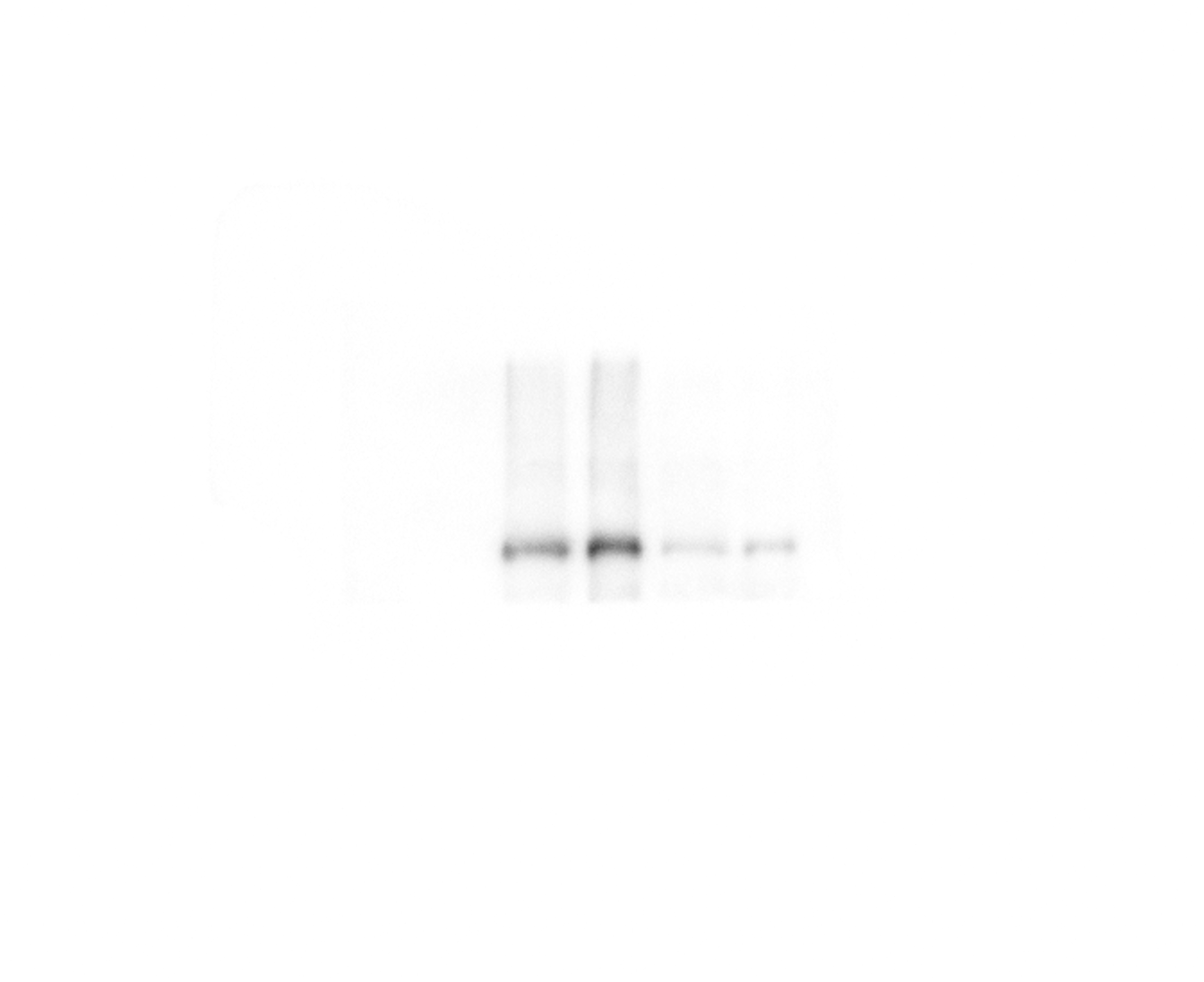

Supplement: Supplementary file 4 — Supplementary Material 4. [file 12964_2024_1770_MOESM4_ESM.zip › SENP3 TAM WB/WB-Figure4/B M0 M2 EndoIP/2023-02-14 ─┌╘┤IP shNC shSENP3 IRF4/INPUT SENP3/INPUT SENP3 5S 0214.Tif]

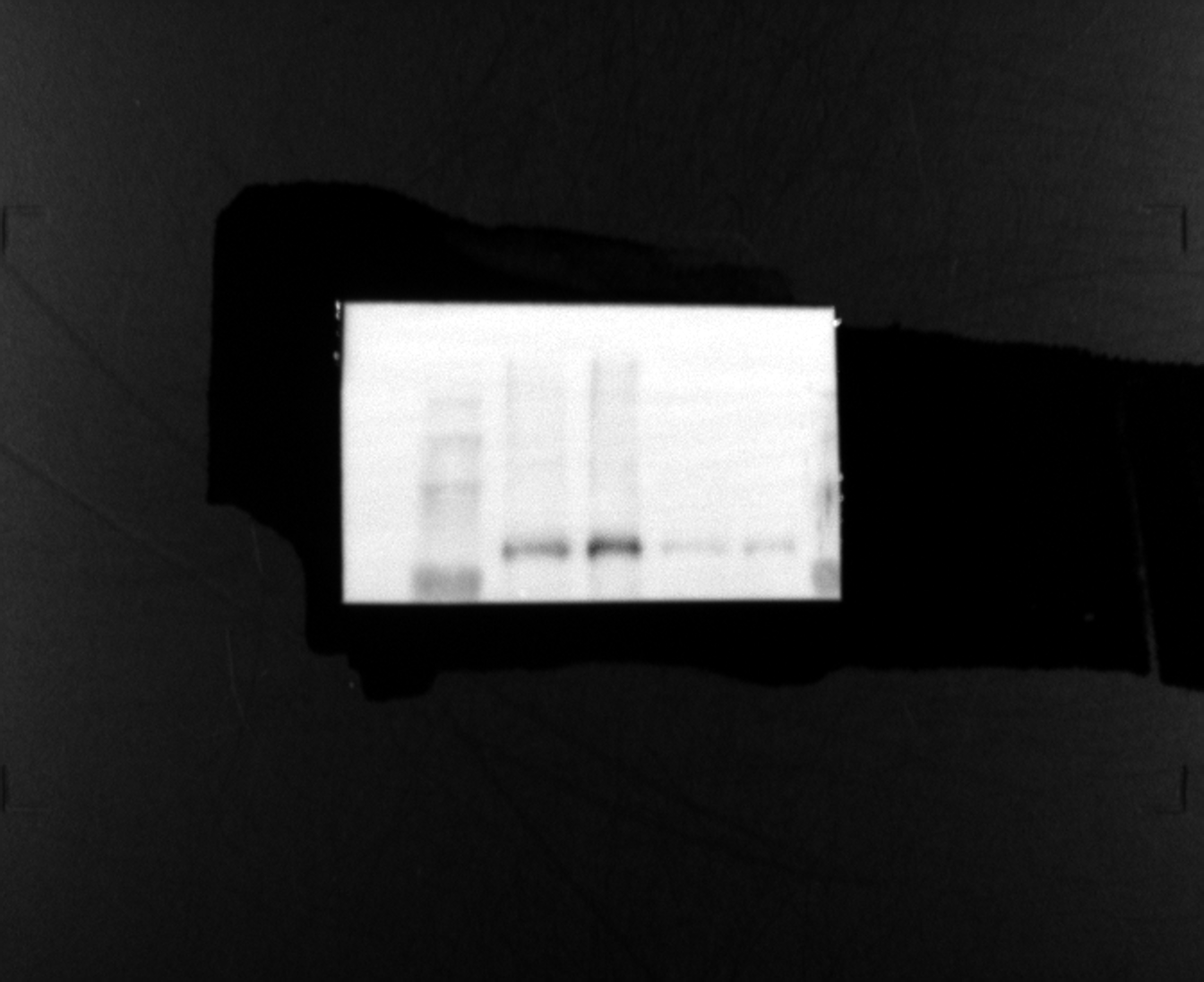

Supplement: Supplementary file 4 — Supplementary Material 4. [file 12964_2024_1770_MOESM4_ESM.zip › SENP3 TAM WB/WB-Figure4/B M0 M2 EndoIP/2023-02-14 ─┌╘┤IP shNC shSENP3 IRF4/INPUT SENP3/INPUT SENP3 5S M 0214.Tif]

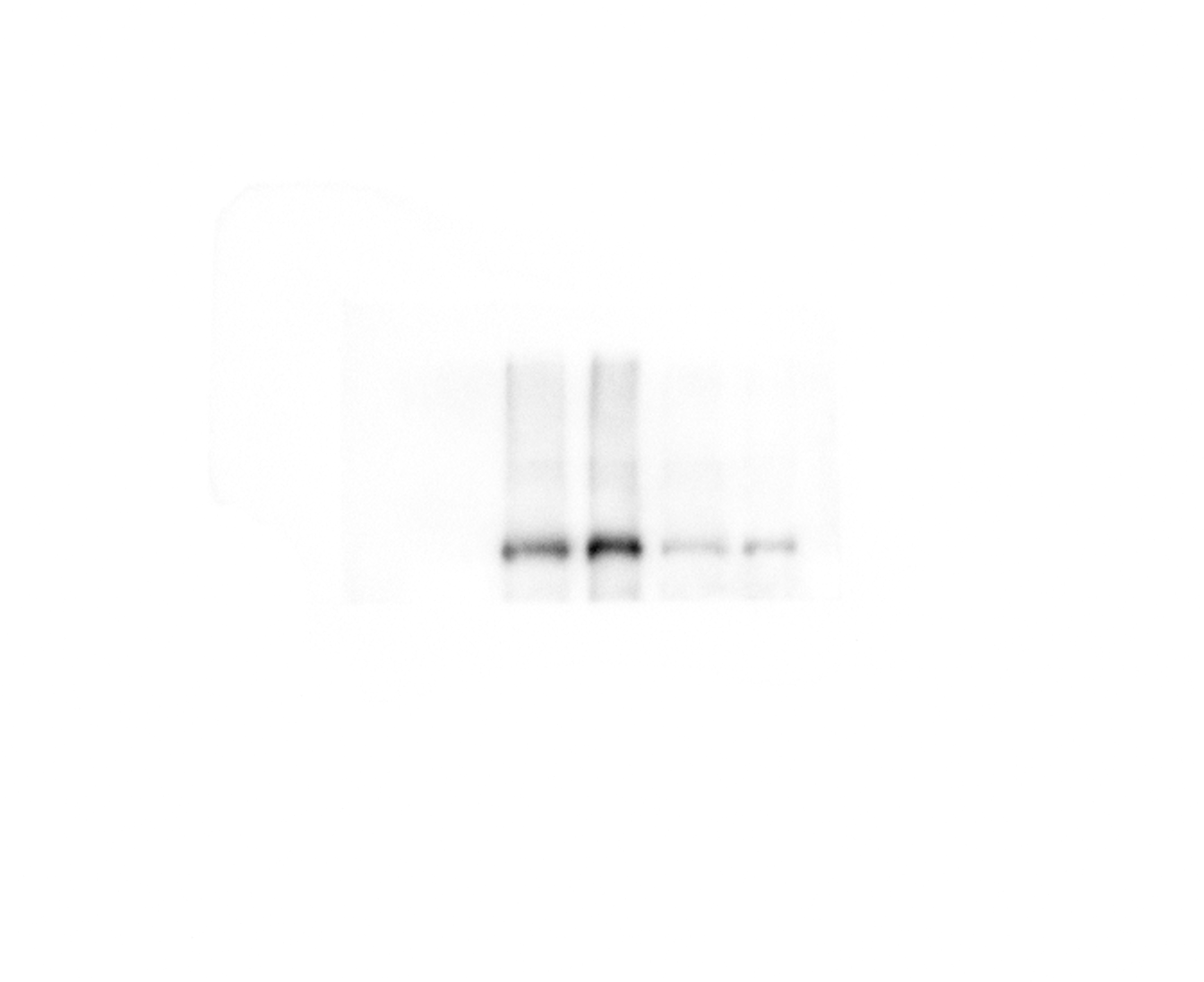

Supplement: Supplementary file 4 — Supplementary Material 4. [file 12964_2024_1770_MOESM4_ESM.zip › SENP3 TAM WB/WB-Figure4/B M0 M2 EndoIP/2023-02-14 ─┌╘┤IP shNC shSENP3 IRF4/INPUT SENP3/INPUT SENP3 7S 0214.Tif]

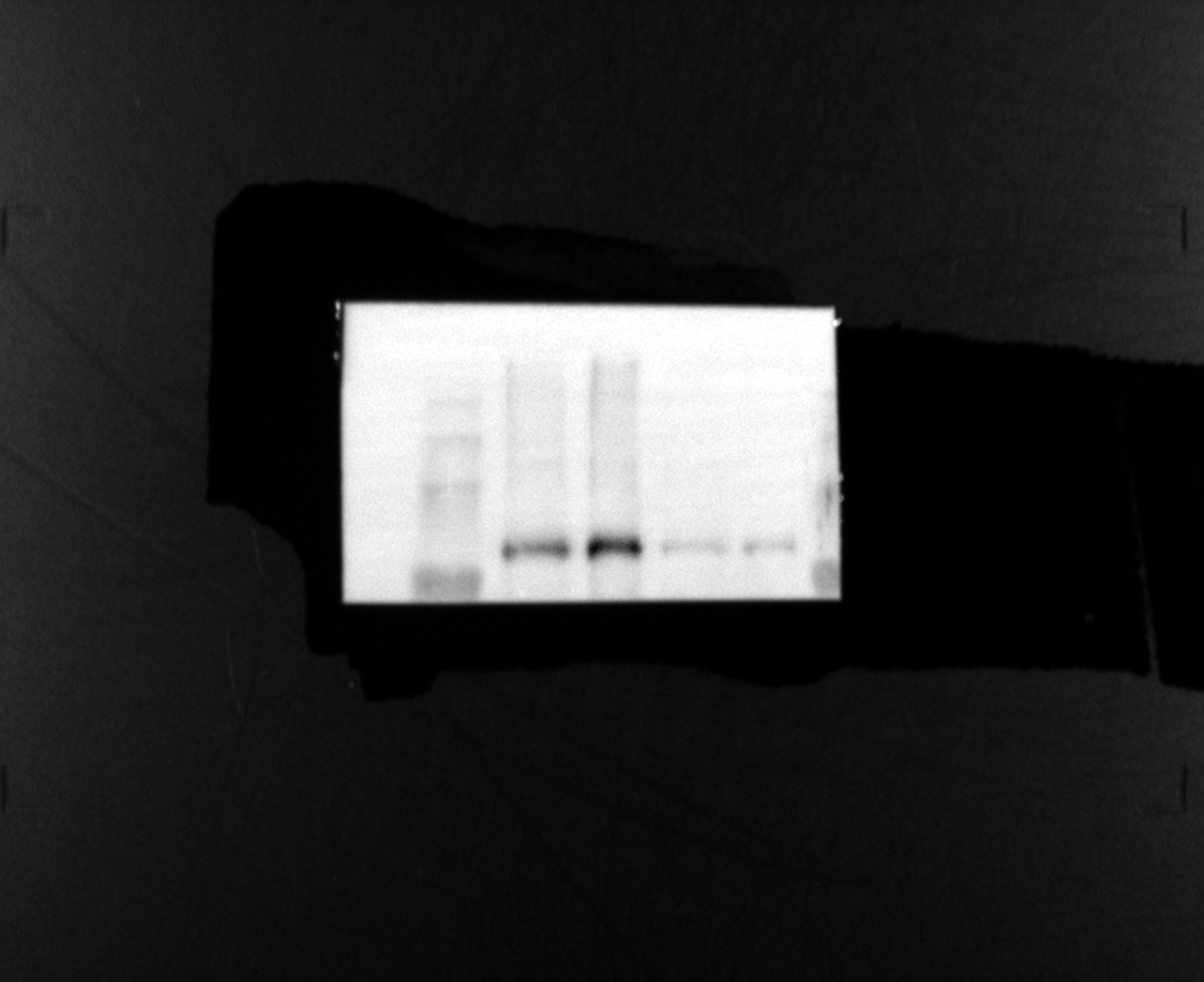

Supplement: Supplementary file 4 — Supplementary Material 4. [file 12964_2024_1770_MOESM4_ESM.zip › SENP3 TAM WB/WB-Figure4/B M0 M2 EndoIP/2023-02-14 ─┌╘┤IP shNC shSENP3 IRF4/INPUT SENP3/INPUT SENP3 7S M 0214.Tif]

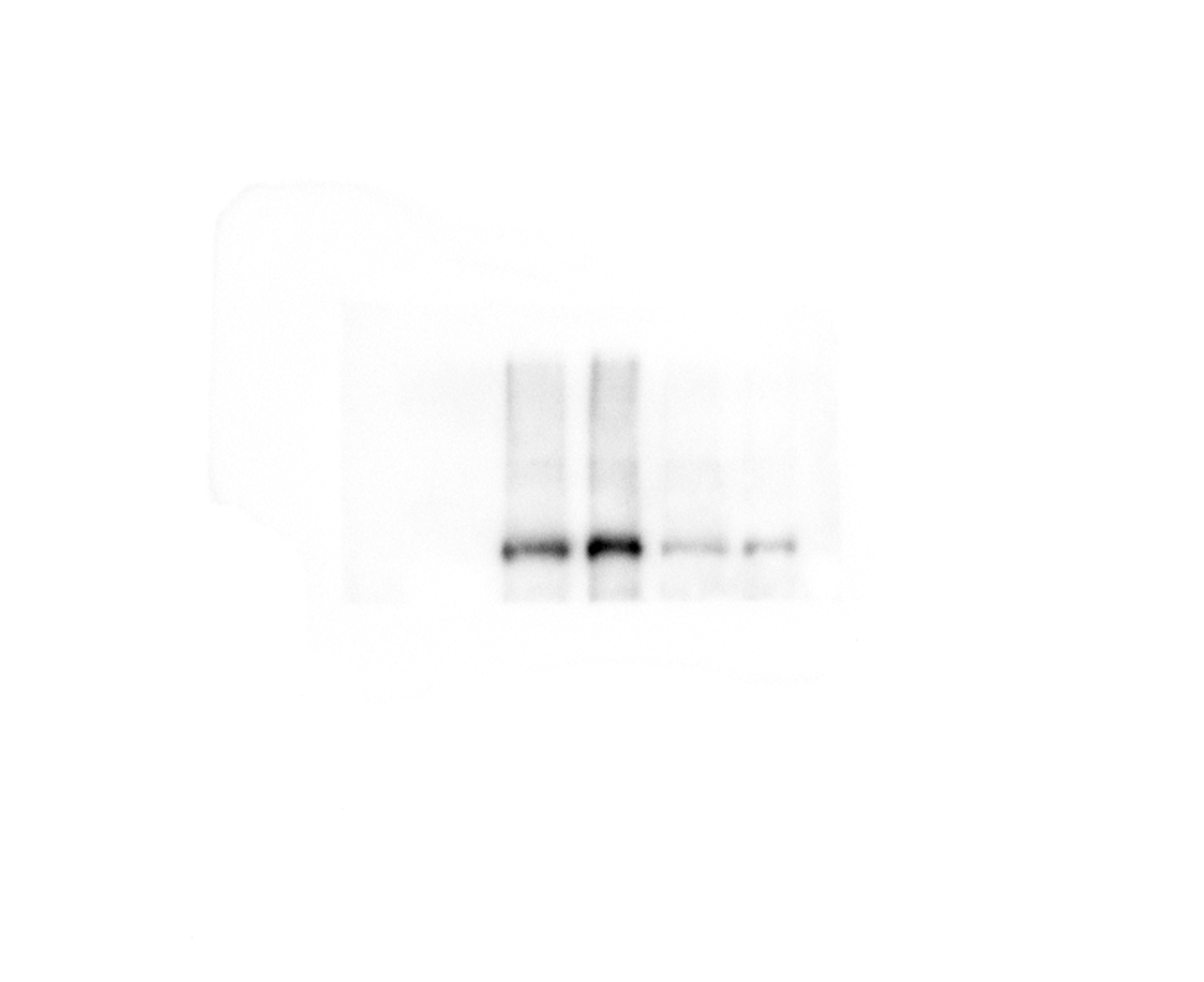

Supplement: Supplementary file 4 — Supplementary Material 4. [file 12964_2024_1770_MOESM4_ESM.zip › SENP3 TAM WB/WB-Figure4/B M0 M2 EndoIP/2023-02-14 ─┌╘┤IP shNC shSENP3 IRF4/INPUT SENP3/INPUT SENP3 9S 0214.Tif]

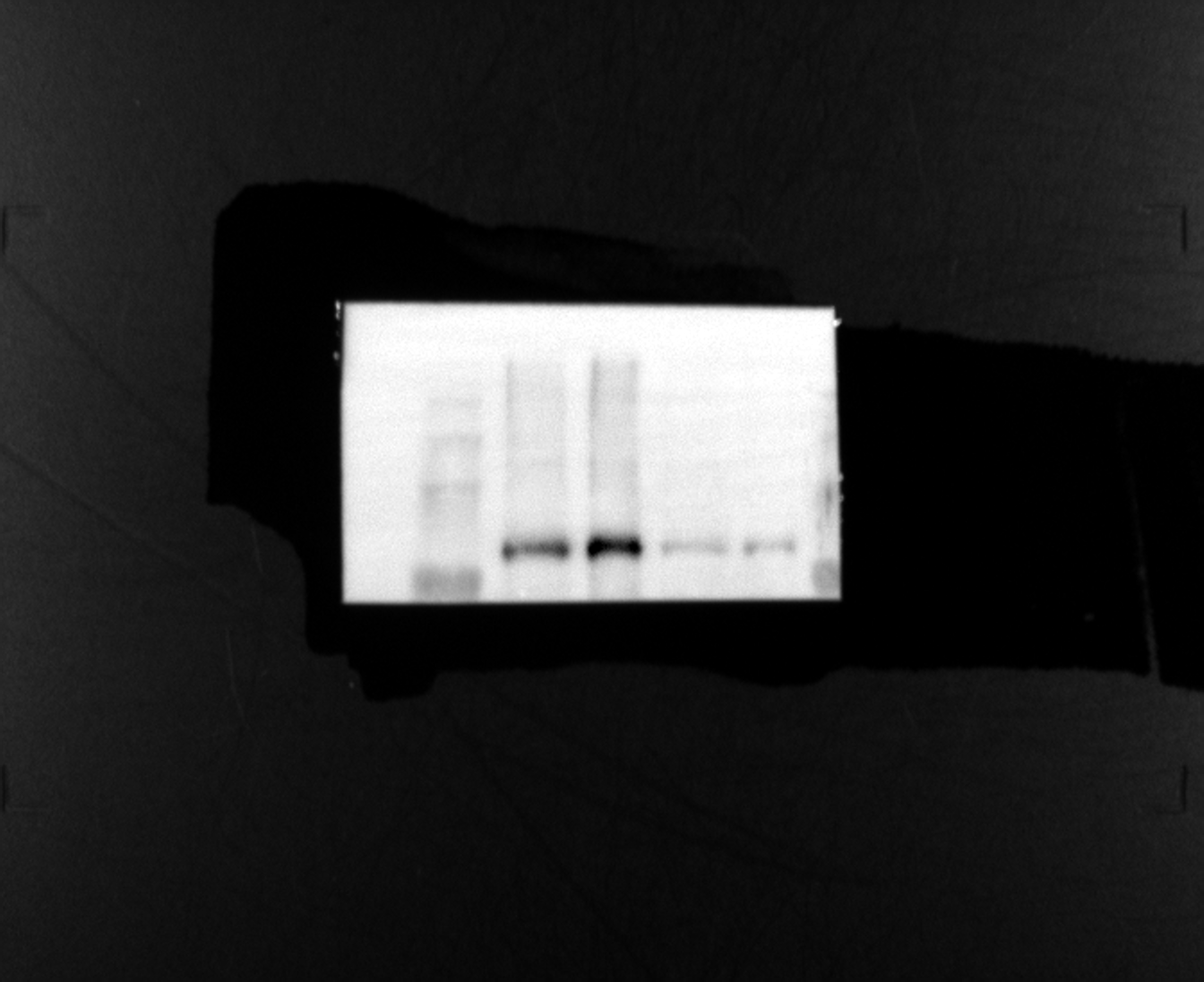

Supplement: Supplementary file 4 — Supplementary Material 4. [file 12964_2024_1770_MOESM4_ESM.zip › SENP3 TAM WB/WB-Figure4/B M0 M2 EndoIP/2023-02-14 ─┌╘┤IP shNC shSENP3 IRF4/INPUT SENP3/INPUT SENP3 9S M 0214.Tif]

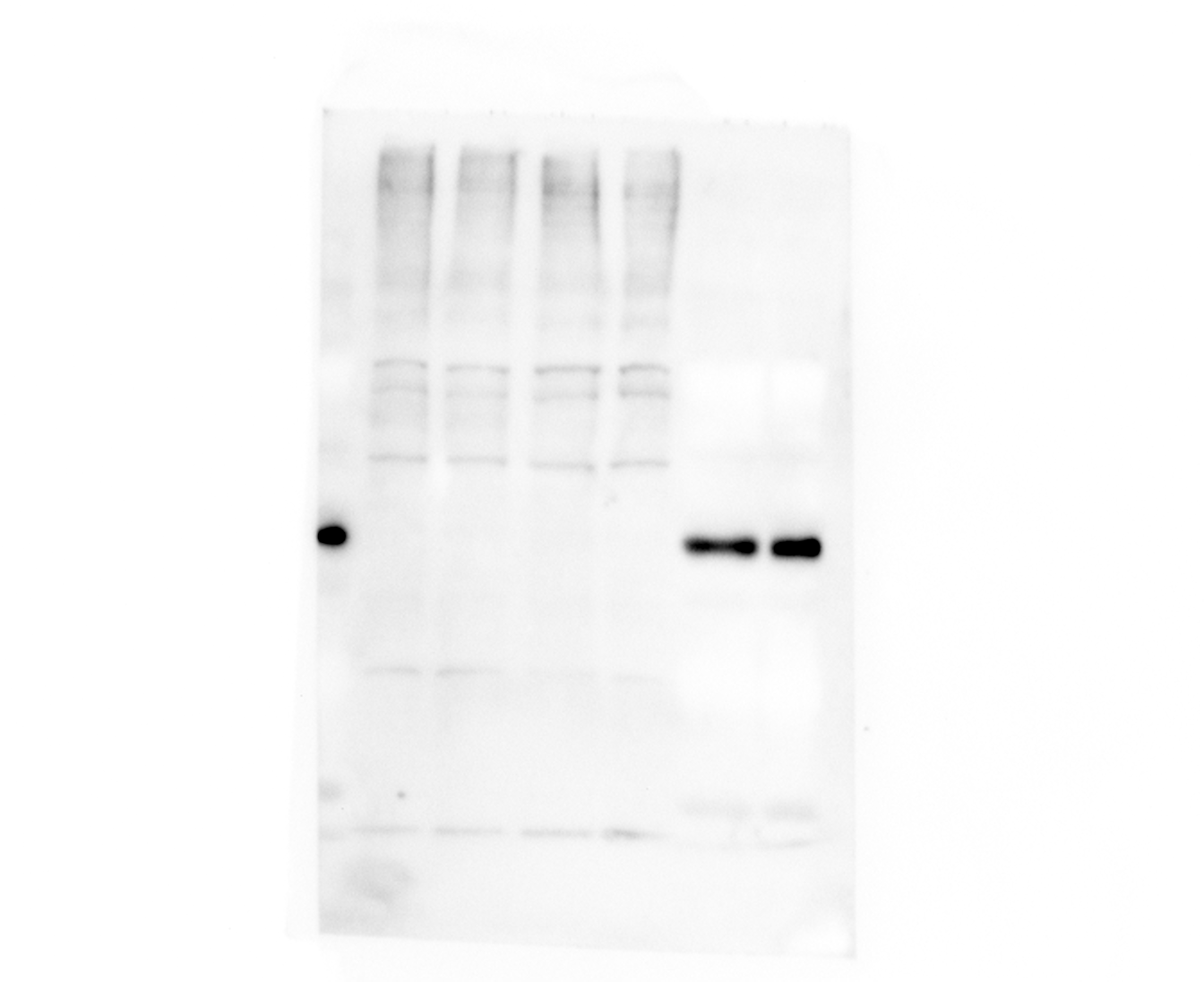

Supplement: Supplementary file 4 — Supplementary Material 4. [file 12964_2024_1770_MOESM4_ESM.zip › SENP3 TAM WB/WB-Figure4/B M0 M2 EndoIP/2023-02-14 ─┌╘┤IP shNC shSENP3 IRF4/INPUT SUMO23/INPUT SUMO 23 40S 0214.Tif]

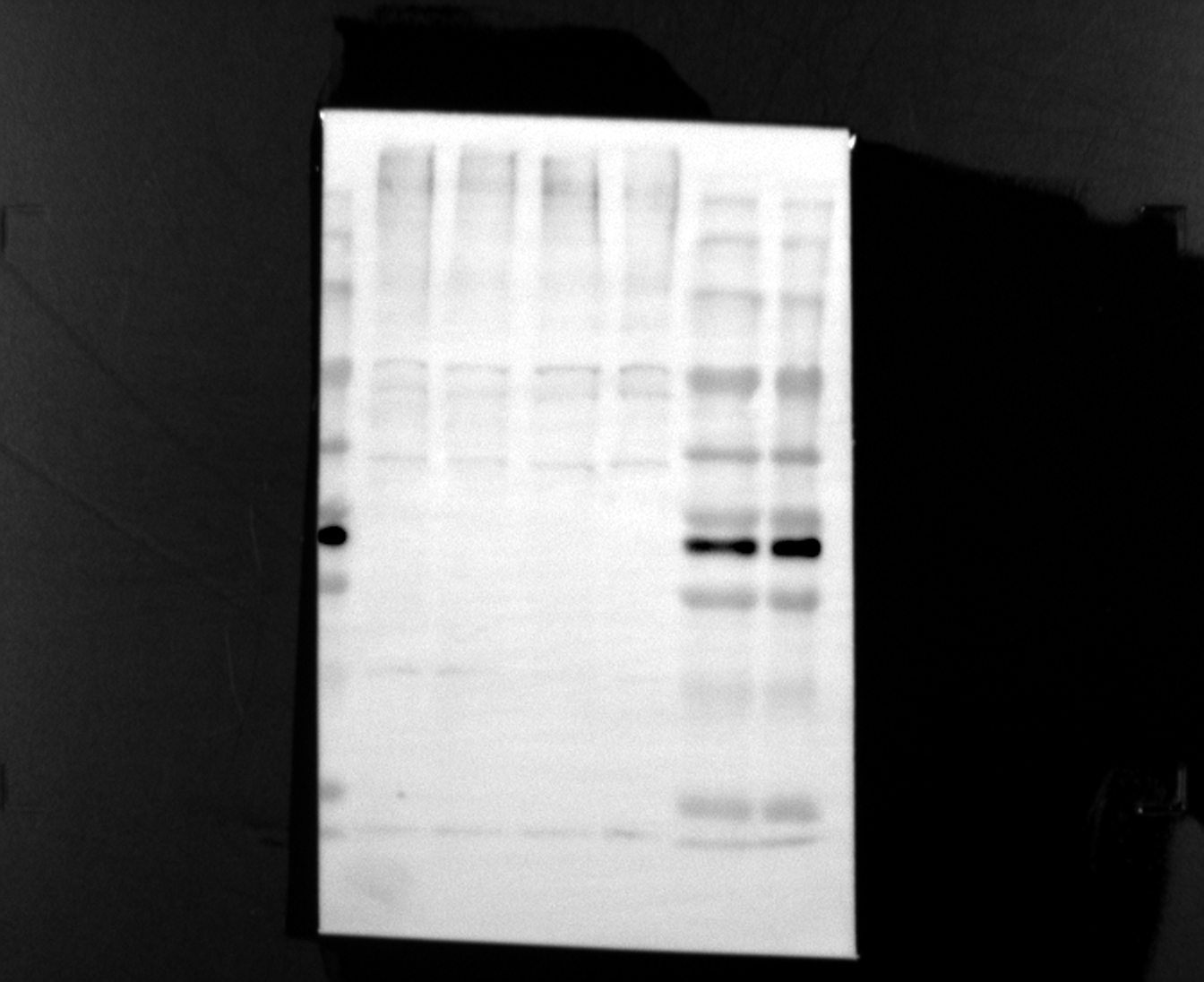

Supplement: Supplementary file 4 — Supplementary Material 4. [file 12964_2024_1770_MOESM4_ESM.zip › SENP3 TAM WB/WB-Figure4/B M0 M2 EndoIP/2023-02-14 ─┌╘┤IP shNC shSENP3 IRF4/INPUT SUMO23/INPUT SUMO 23 40S M 0214.Tif]

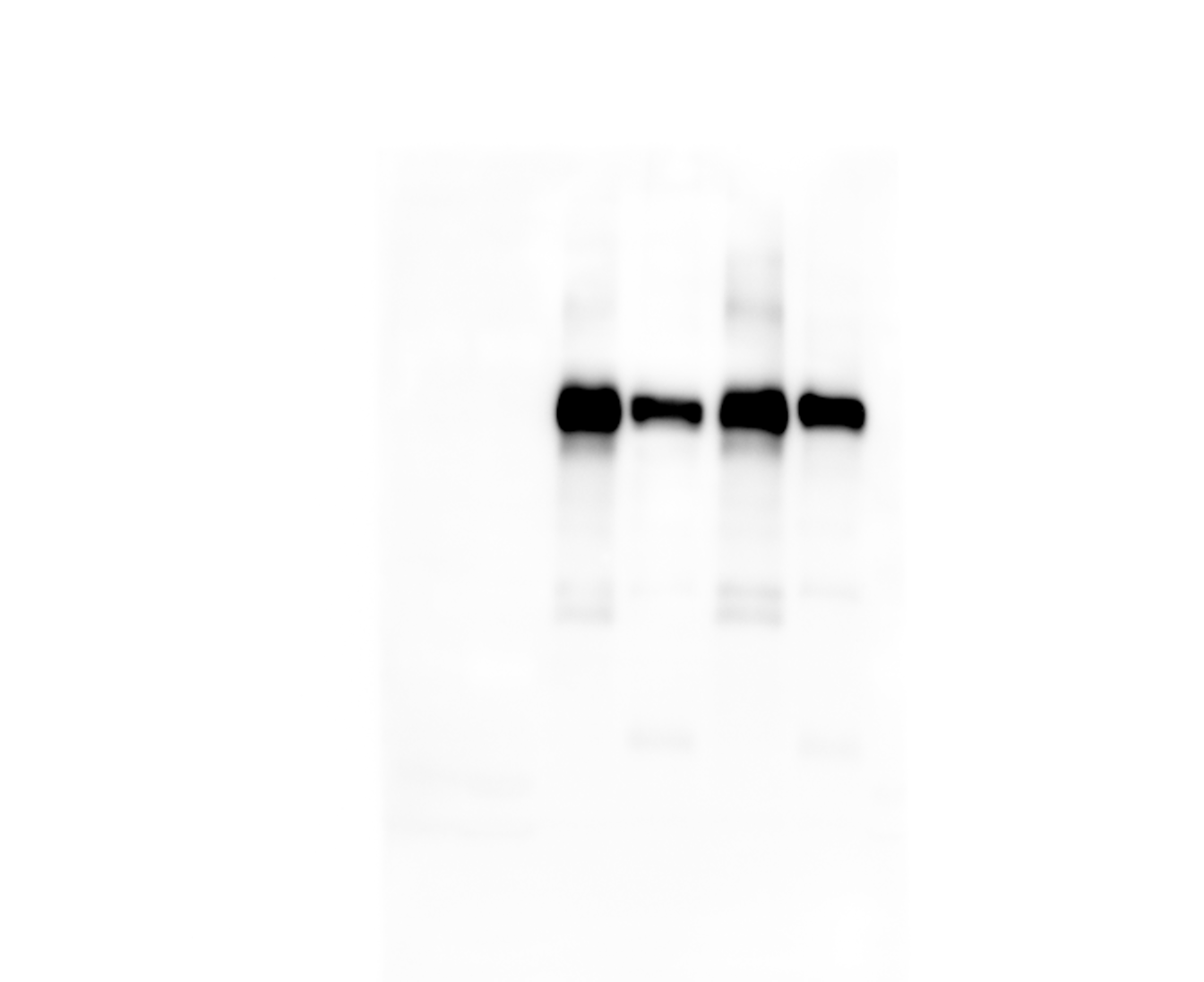

Supplement: Supplementary file 4 — Supplementary Material 4. [file 12964_2024_1770_MOESM4_ESM.zip › SENP3 TAM WB/WB-Figure4/B M0 M2 EndoIP/2023-02-14 ─┌╘┤IP shNC shSENP3 IRF4/IP IRF4/IP IRF4 0214 15S.Tif]

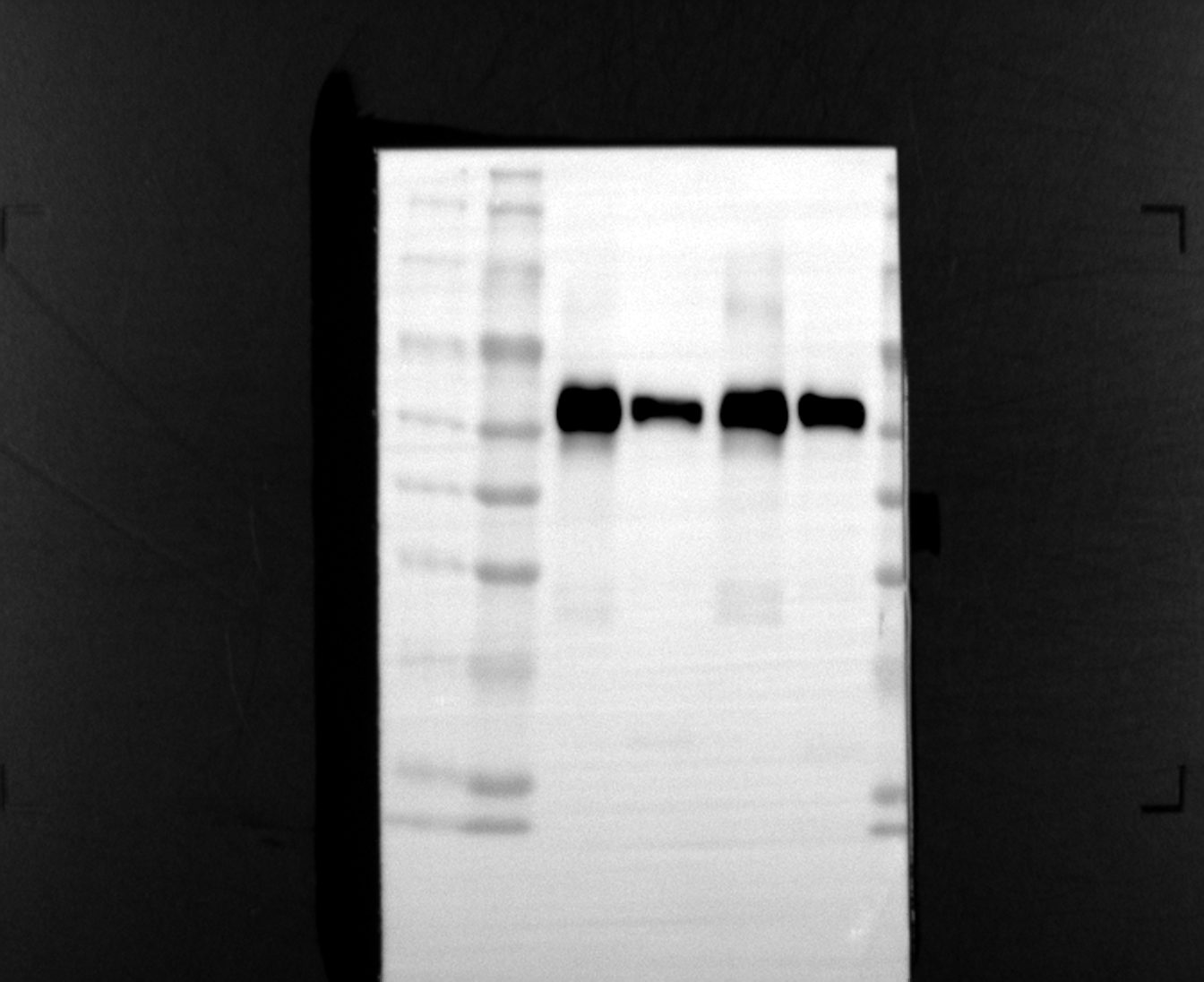

Supplement: Supplementary file 4 — Supplementary Material 4. [file 12964_2024_1770_MOESM4_ESM.zip › SENP3 TAM WB/WB-Figure4/B M0 M2 EndoIP/2023-02-14 ─┌╘┤IP shNC shSENP3 IRF4/IP IRF4/IP IRF4 0214 M 15S.Tif]

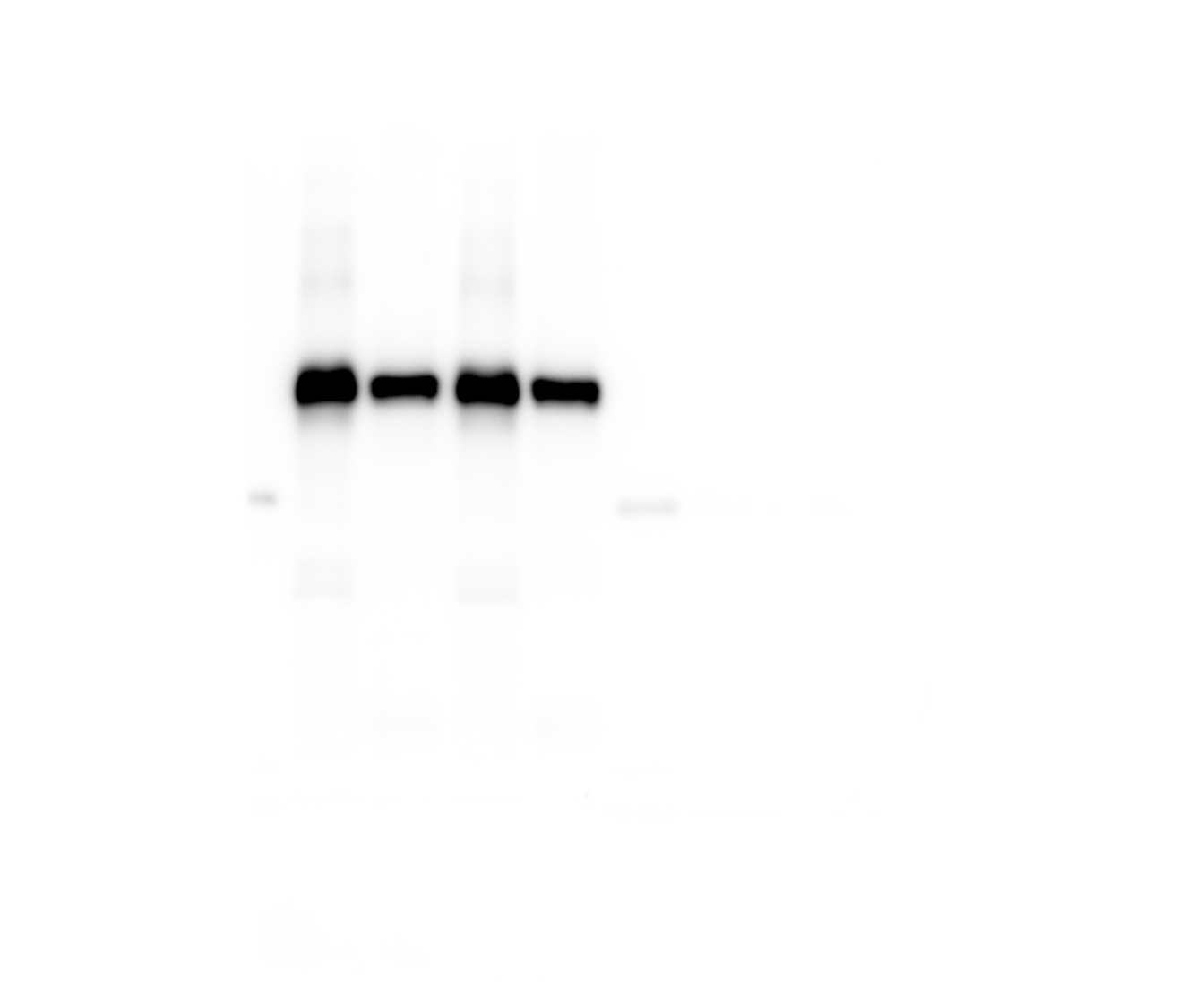

Supplement: Supplementary file 4 — Supplementary Material 4. [file 12964_2024_1770_MOESM4_ESM.zip › SENP3 TAM WB/WB-Figure4/B M0 M2 EndoIP/2023-02-14 ─┌╘┤IP shNC shSENP3 IRF4/IP SUMO23/IP SUMO23 10S 0214.Tif]

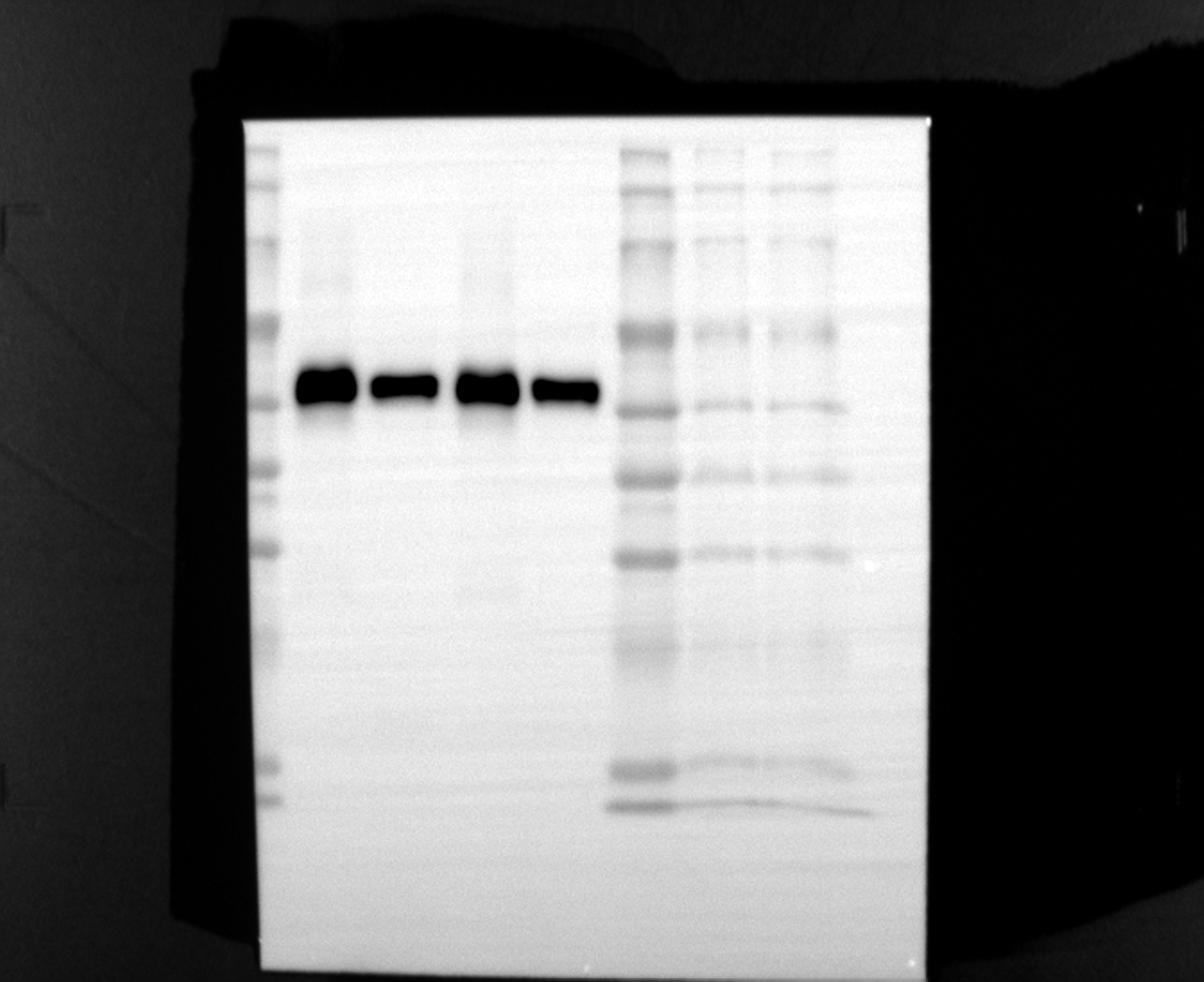

Supplement: Supplementary file 4 — Supplementary Material 4. [file 12964_2024_1770_MOESM4_ESM.zip › SENP3 TAM WB/WB-Figure4/B M0 M2 EndoIP/2023-02-14 ─┌╘┤IP shNC shSENP3 IRF4/IP SUMO23/IP SUMO23 10S M 0214.Tif]

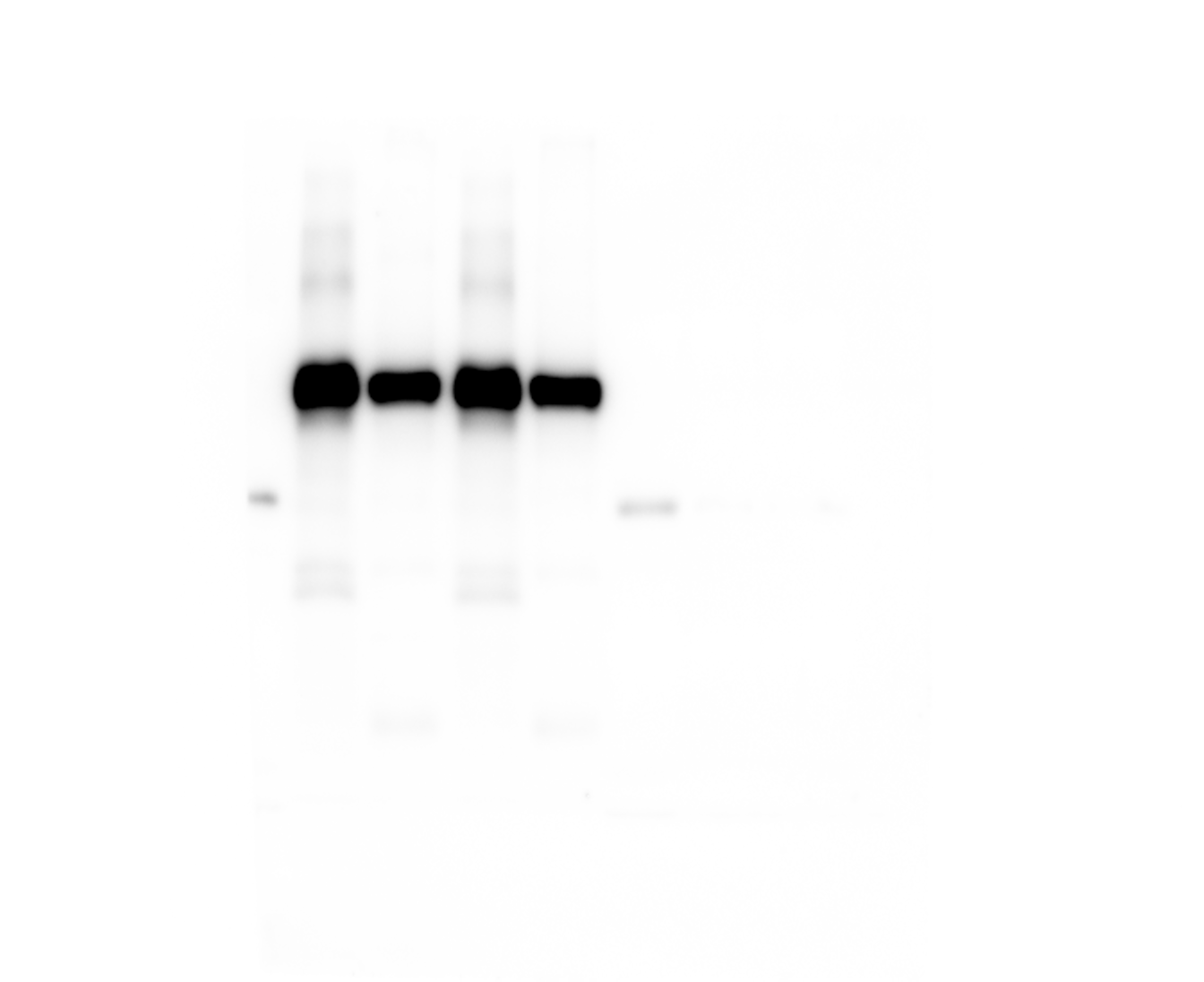

Supplement: Supplementary file 4 — Supplementary Material 4. [file 12964_2024_1770_MOESM4_ESM.zip › SENP3 TAM WB/WB-Figure4/B M0 M2 EndoIP/2023-02-14 ─┌╘┤IP shNC shSENP3 IRF4/IP SUMO23/IP SUMO23 20S 0214.Tif]

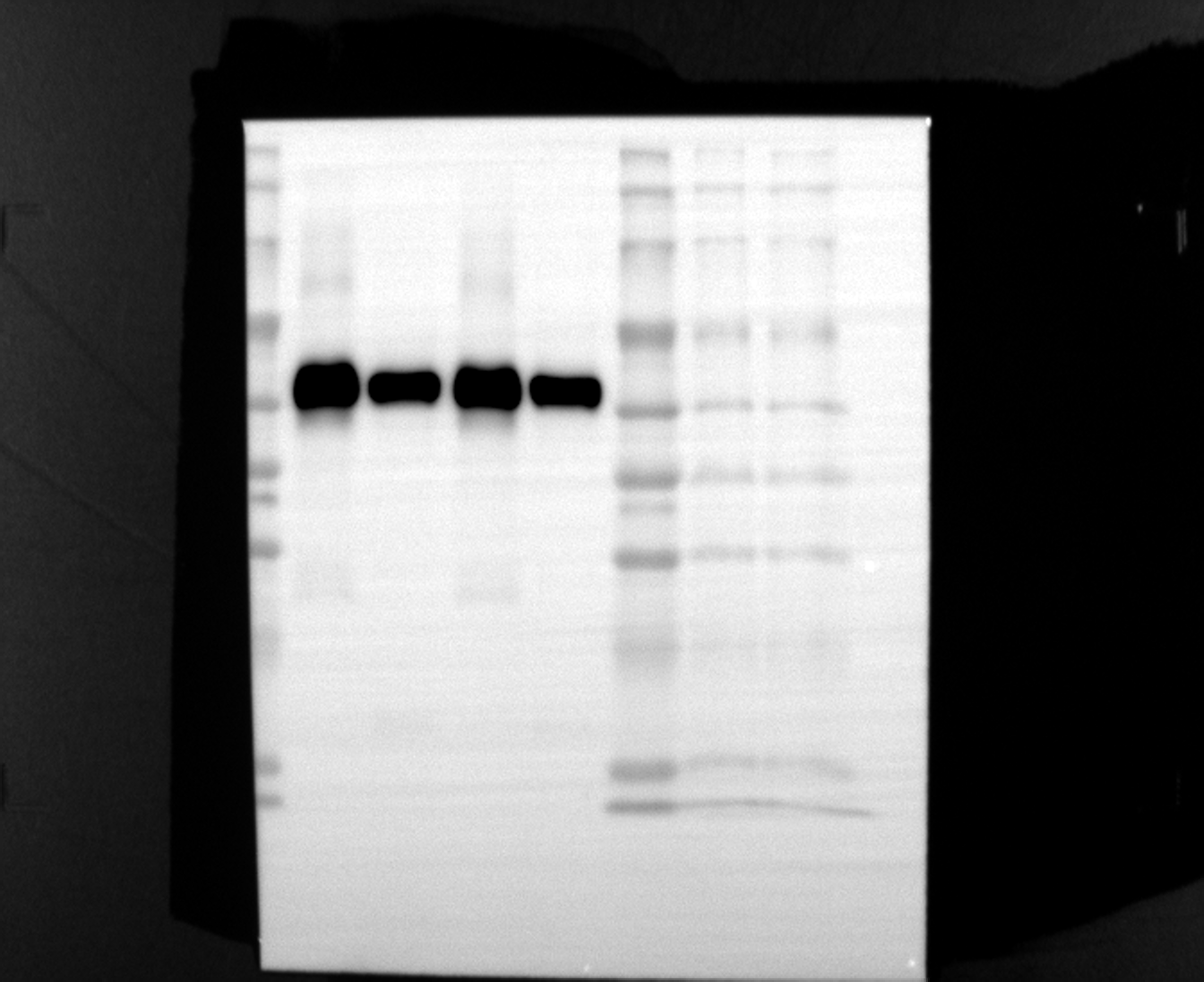

Supplement: Supplementary file 4 — Supplementary Material 4. [file 12964_2024_1770_MOESM4_ESM.zip › SENP3 TAM WB/WB-Figure4/B M0 M2 EndoIP/2023-02-14 ─┌╘┤IP shNC shSENP3 IRF4/IP SUMO23/IP SUMO23 20S M 0214.Tif]

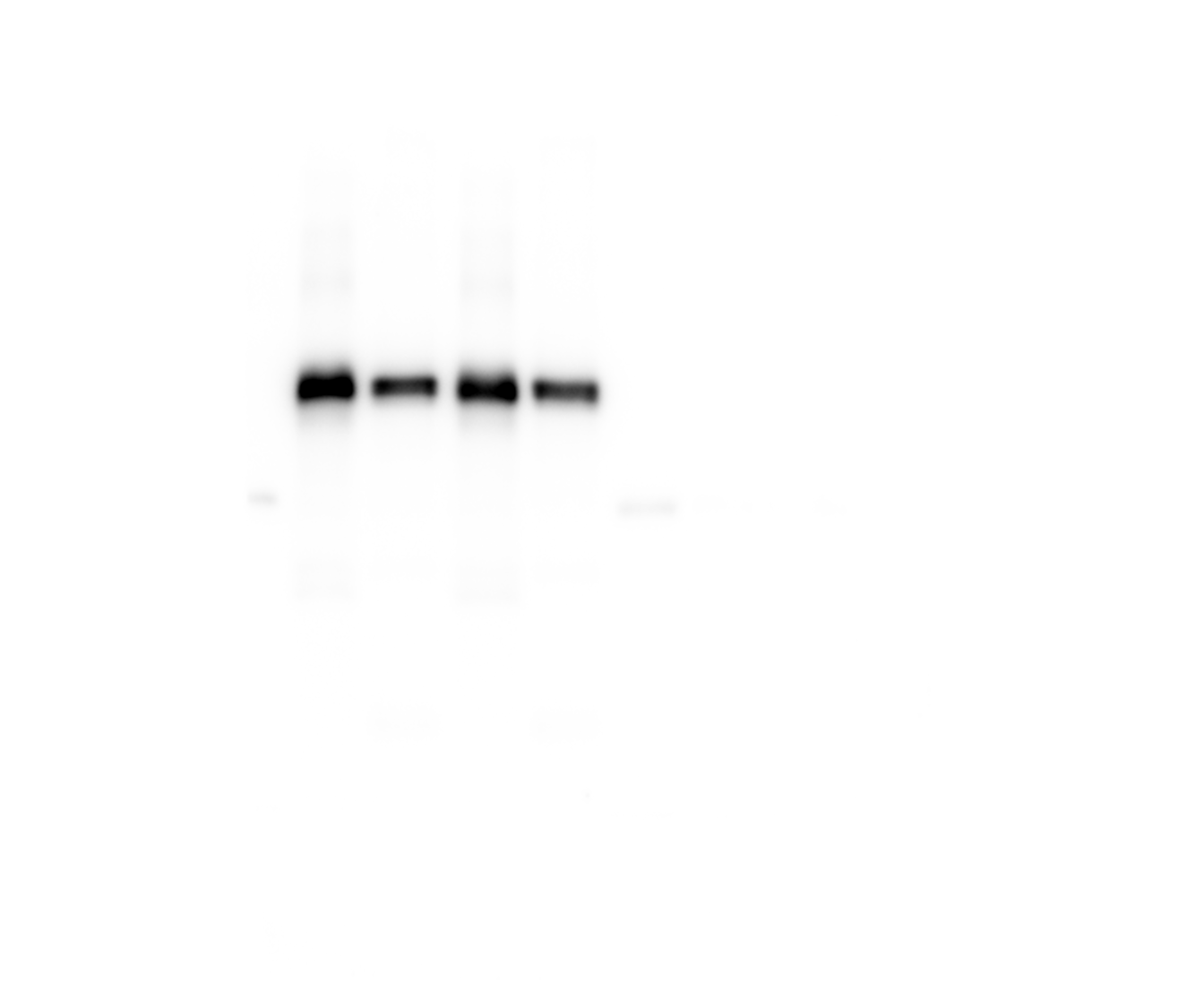

Supplement: Supplementary file 4 — Supplementary Material 4. [file 12964_2024_1770_MOESM4_ESM.zip › SENP3 TAM WB/WB-Figure4/B M0 M2 EndoIP/2023-02-14 ─┌╘┤IP shNC shSENP3 IRF4/IP SUMO23/IP SUMO23 5S 0214.Tif]

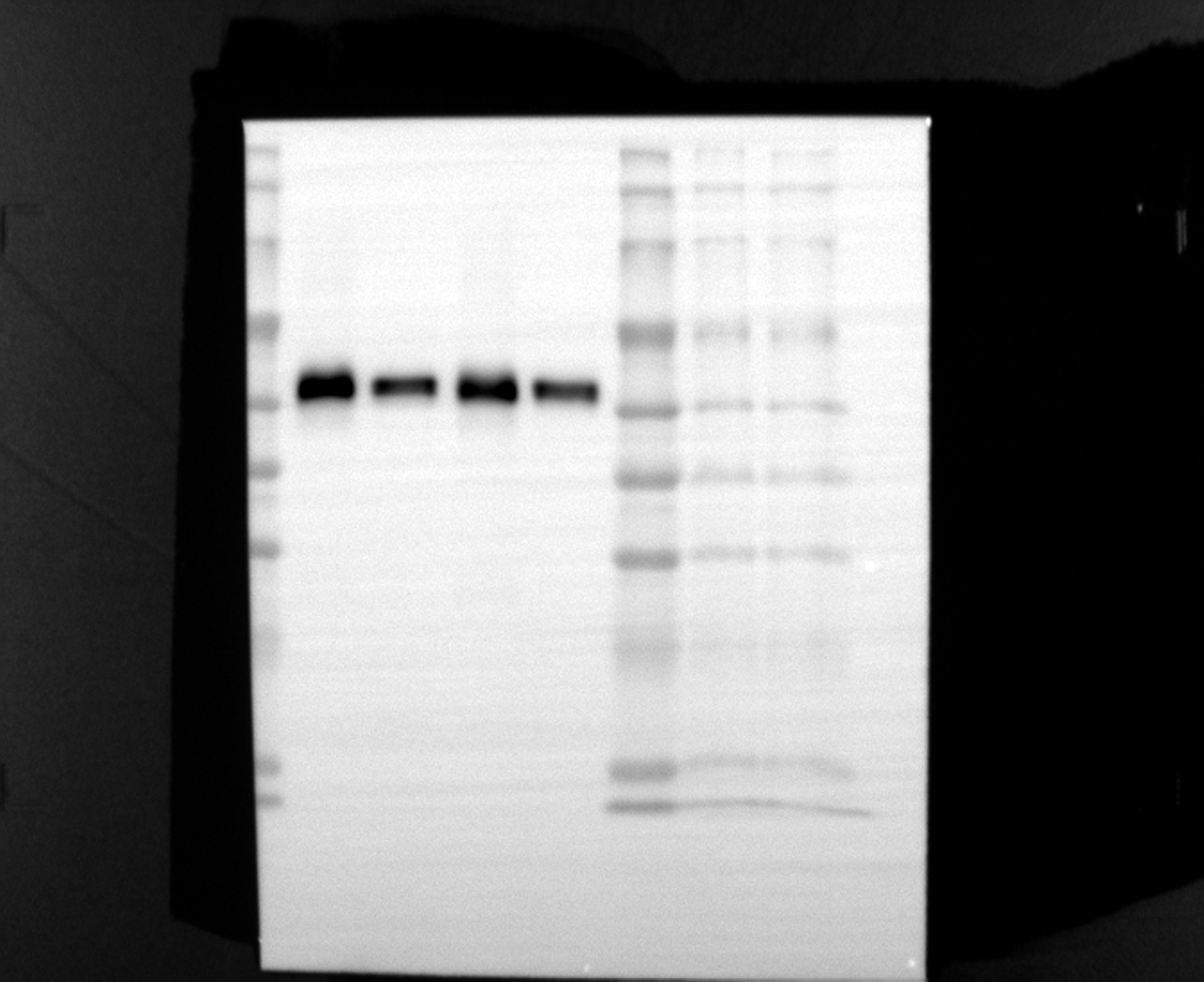

Supplement: Supplementary file 4 — Supplementary Material 4. [file 12964_2024_1770_MOESM4_ESM.zip › SENP3 TAM WB/WB-Figure4/B M0 M2 EndoIP/2023-02-14 ─┌╘┤IP shNC shSENP3 IRF4/IP SUMO23/IP SUMO23 5S M 0214.Tif]

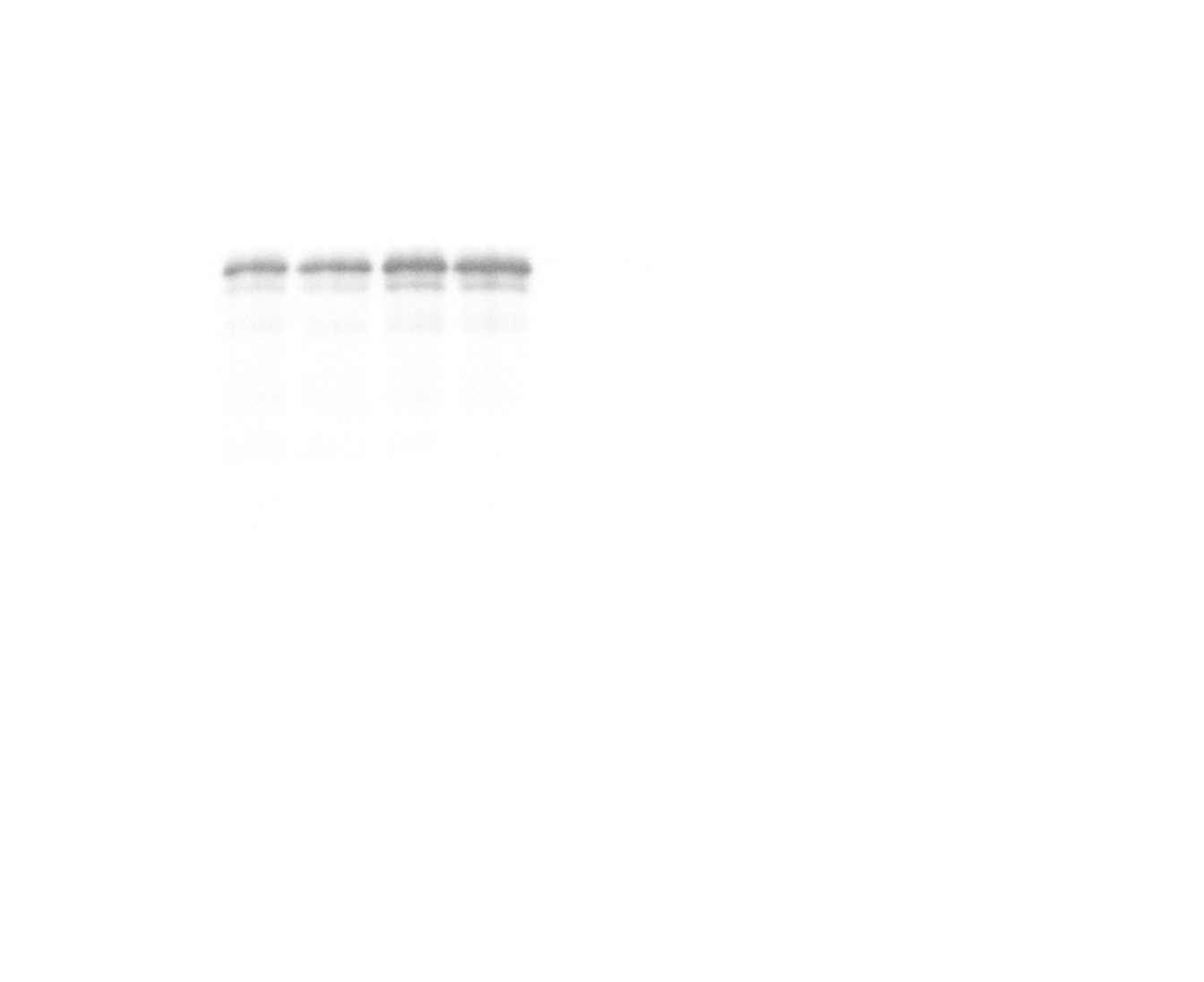

Supplement: Supplementary file 4 — Supplementary Material 4. [file 12964_2024_1770_MOESM4_ESM.zip › SENP3 TAM WB/WB-Figure4/B M0 M2 EndoIP/2023-02-16 ─┌╘┤IP shNC shSENP3 IRF4/INPUT IRF4/NPUT IRF4 0.5S 0216.Tif]

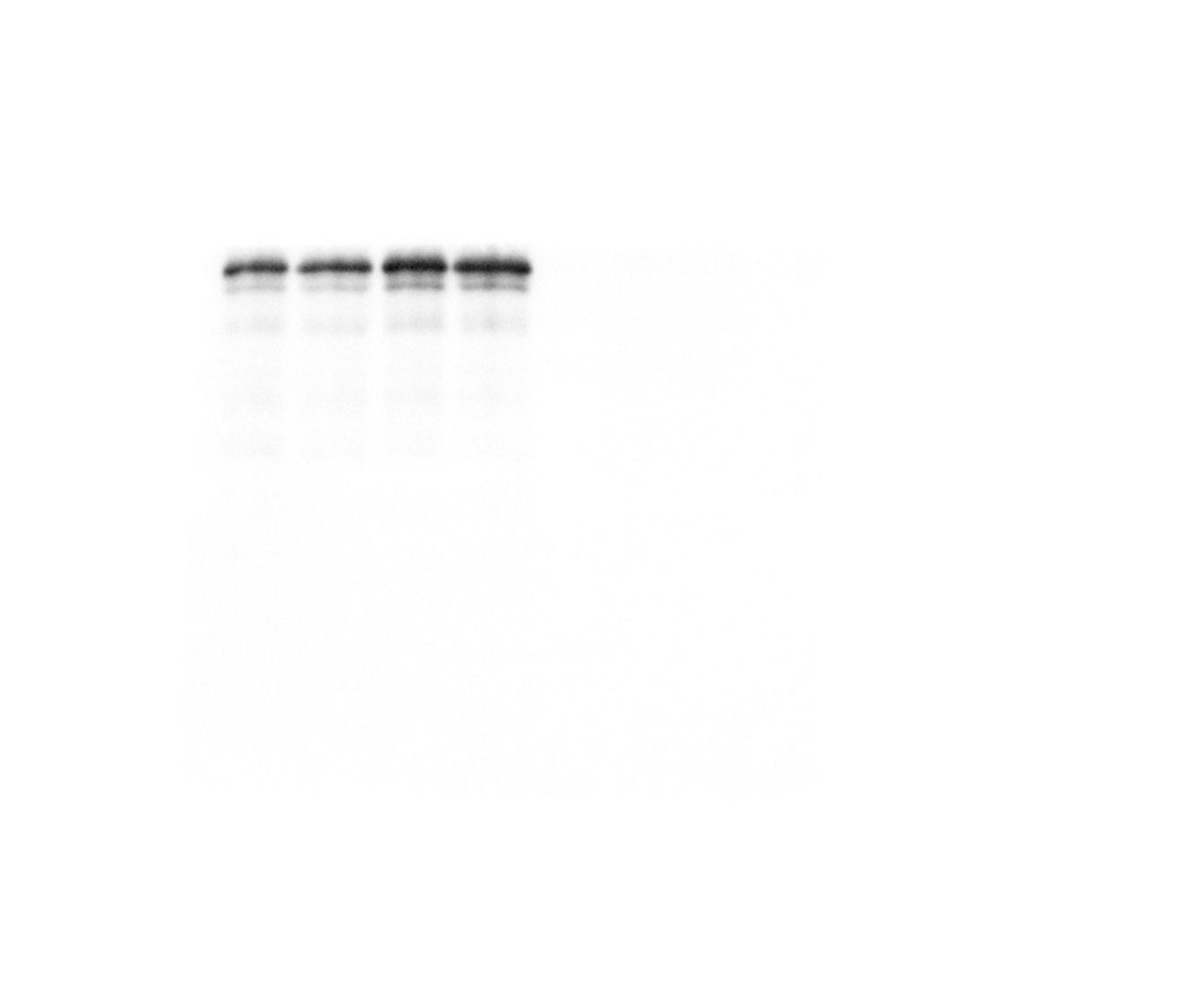

Supplement: Supplementary file 4 — Supplementary Material 4. [file 12964_2024_1770_MOESM4_ESM.zip › SENP3 TAM WB/WB-Figure4/B M0 M2 EndoIP/2023-02-16 ─┌╘┤IP shNC shSENP3 IRF4/INPUT IRF4/NPUT IRF4 1S 0216.Tif]

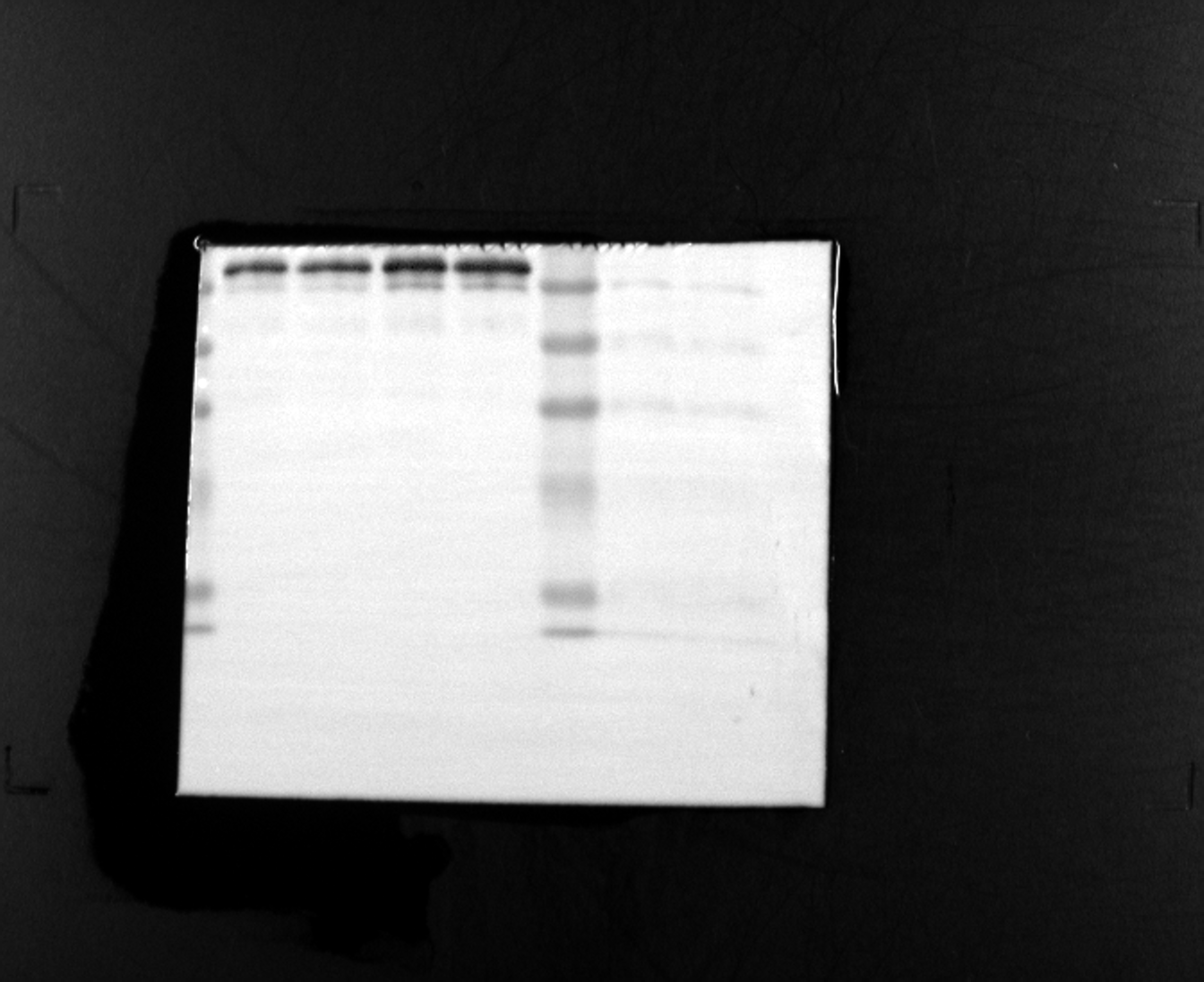

Supplement: Supplementary file 4 — Supplementary Material 4. [file 12964_2024_1770_MOESM4_ESM.zip › SENP3 TAM WB/WB-Figure4/B M0 M2 EndoIP/2023-02-16 ─┌╘┤IP shNC shSENP3 IRF4/INPUT IRF4/NPUT IRF4 1S M 0216.Tif]

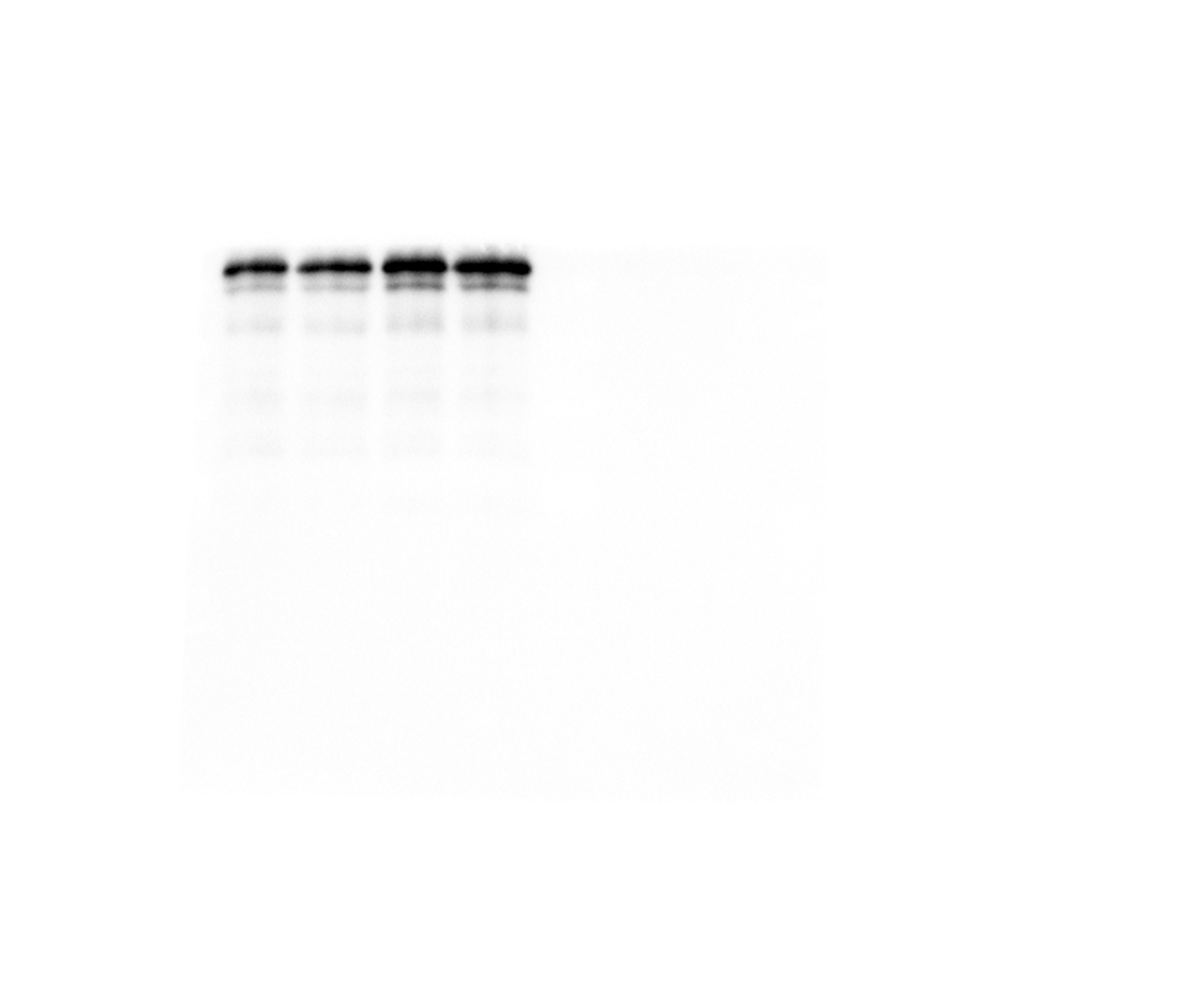

Supplement: Supplementary file 4 — Supplementary Material 4. [file 12964_2024_1770_MOESM4_ESM.zip › SENP3 TAM WB/WB-Figure4/B M0 M2 EndoIP/2023-02-16 ─┌╘┤IP shNC shSENP3 IRF4/INPUT IRF4/NPUT IRF4 2S 0216.Tif]

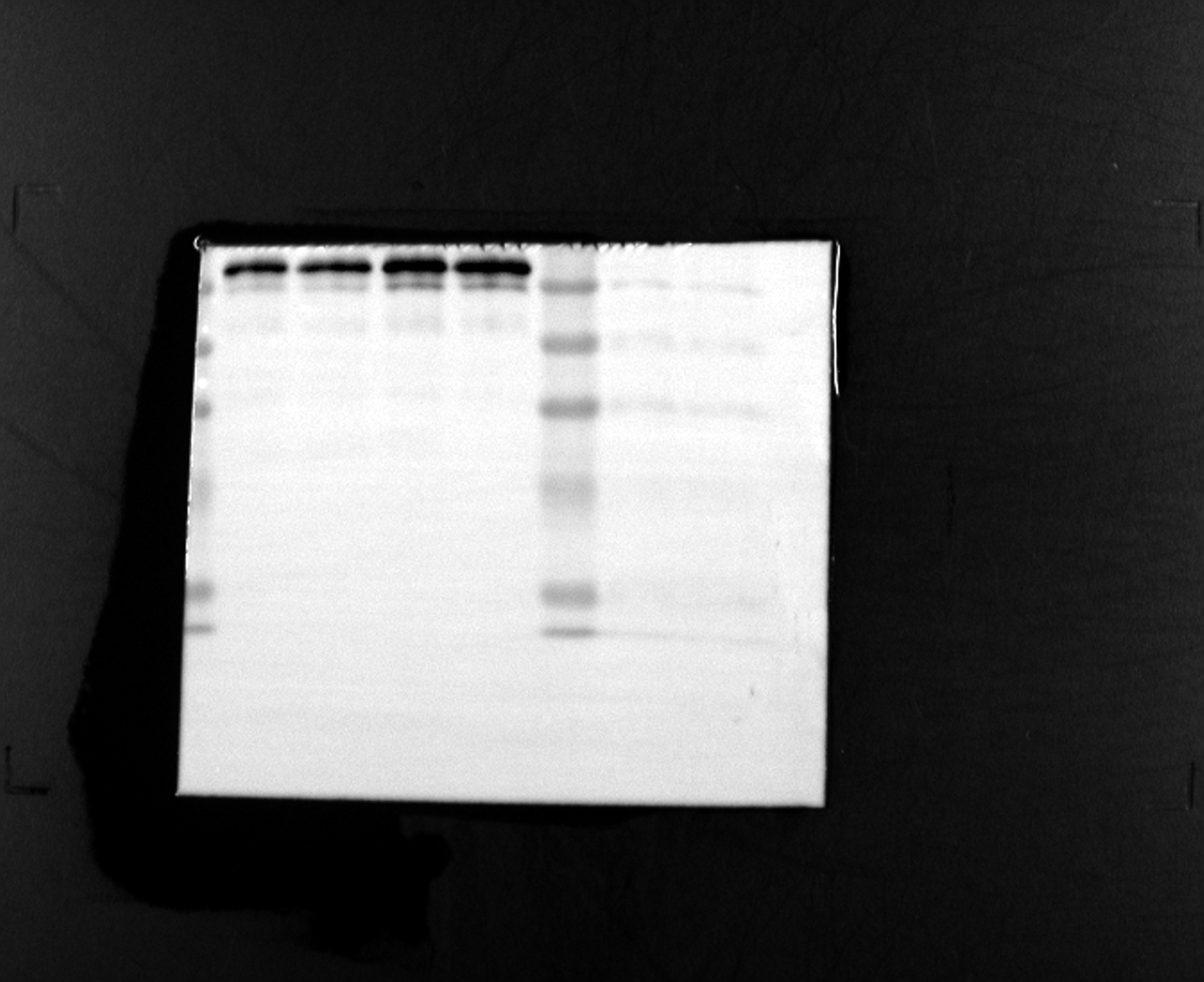

Supplement: Supplementary file 4 — Supplementary Material 4. [file 12964_2024_1770_MOESM4_ESM.zip › SENP3 TAM WB/WB-Figure4/B M0 M2 EndoIP/2023-02-16 ─┌╘┤IP shNC shSENP3 IRF4/INPUT IRF4/NPUT IRF4 2S M 0216.Tif]

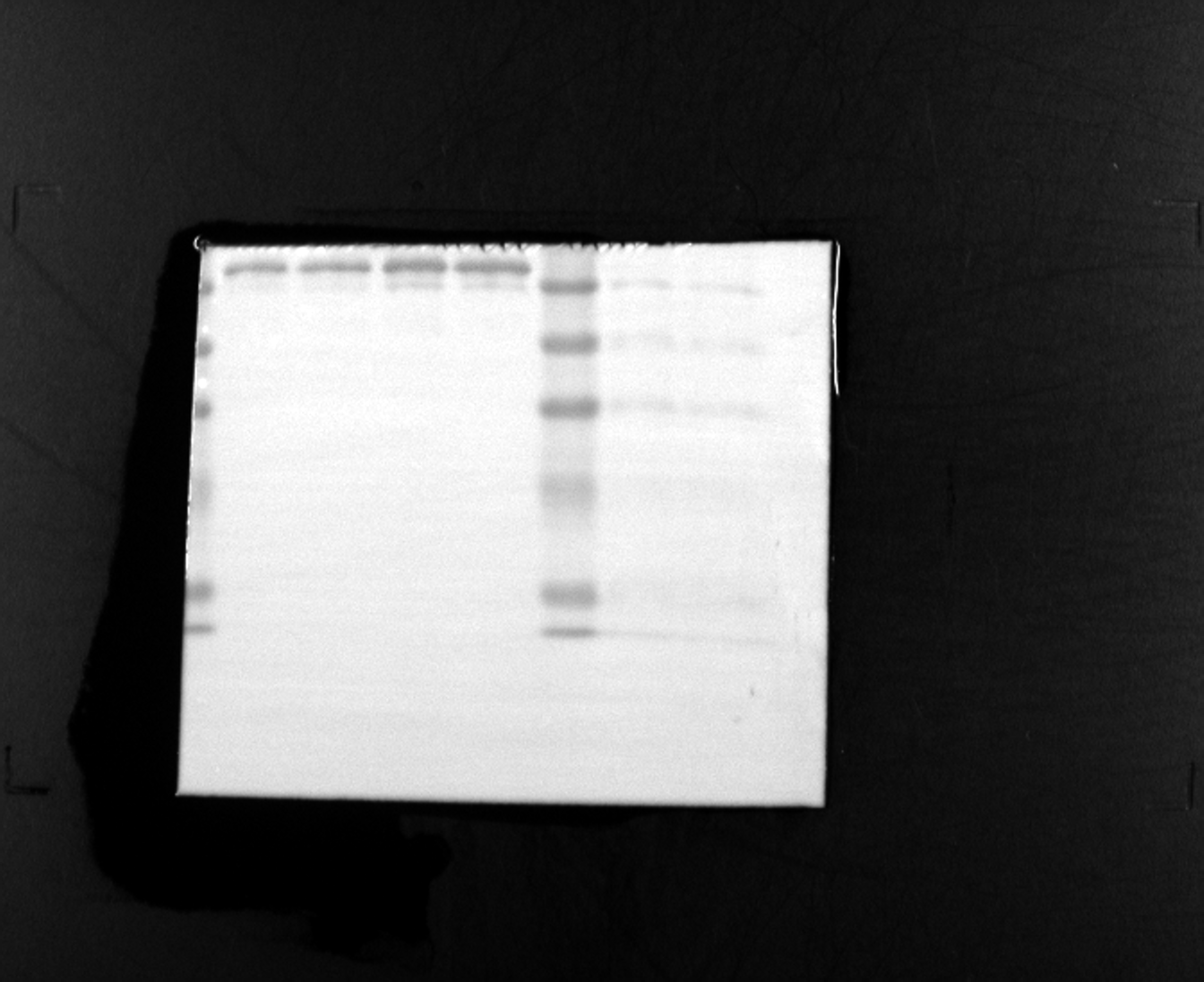

Supplement: Supplementary file 4 — Supplementary Material 4. [file 12964_2024_1770_MOESM4_ESM.zip › SENP3 TAM WB/WB-Figure4/B M0 M2 EndoIP/2023-02-16 ─┌╘┤IP shNC shSENP3 IRF4/INPUT IRF4/NPUT IRF4 0.5S M 0216.Tif]

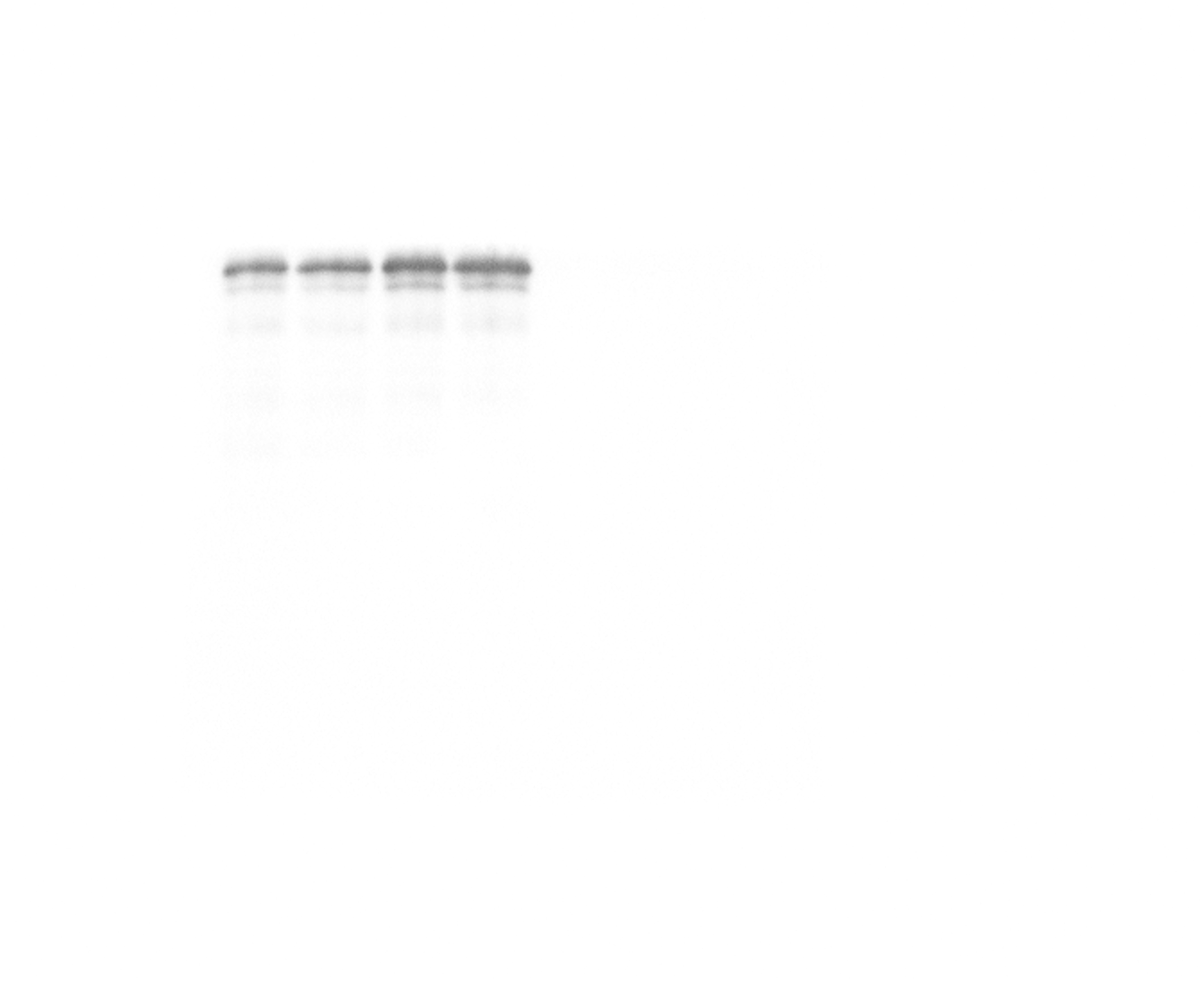

Supplement: Supplementary file 4 — Supplementary Material 4. [file 12964_2024_1770_MOESM4_ESM.zip › SENP3 TAM WB/WB-Figure4/B M0 M2 EndoIP/2023-02-16 ─┌╘┤IP shNC shSENP3 IRF4/INPUT IRF4/NPUT IRF4 0.7S 0216.Tif]

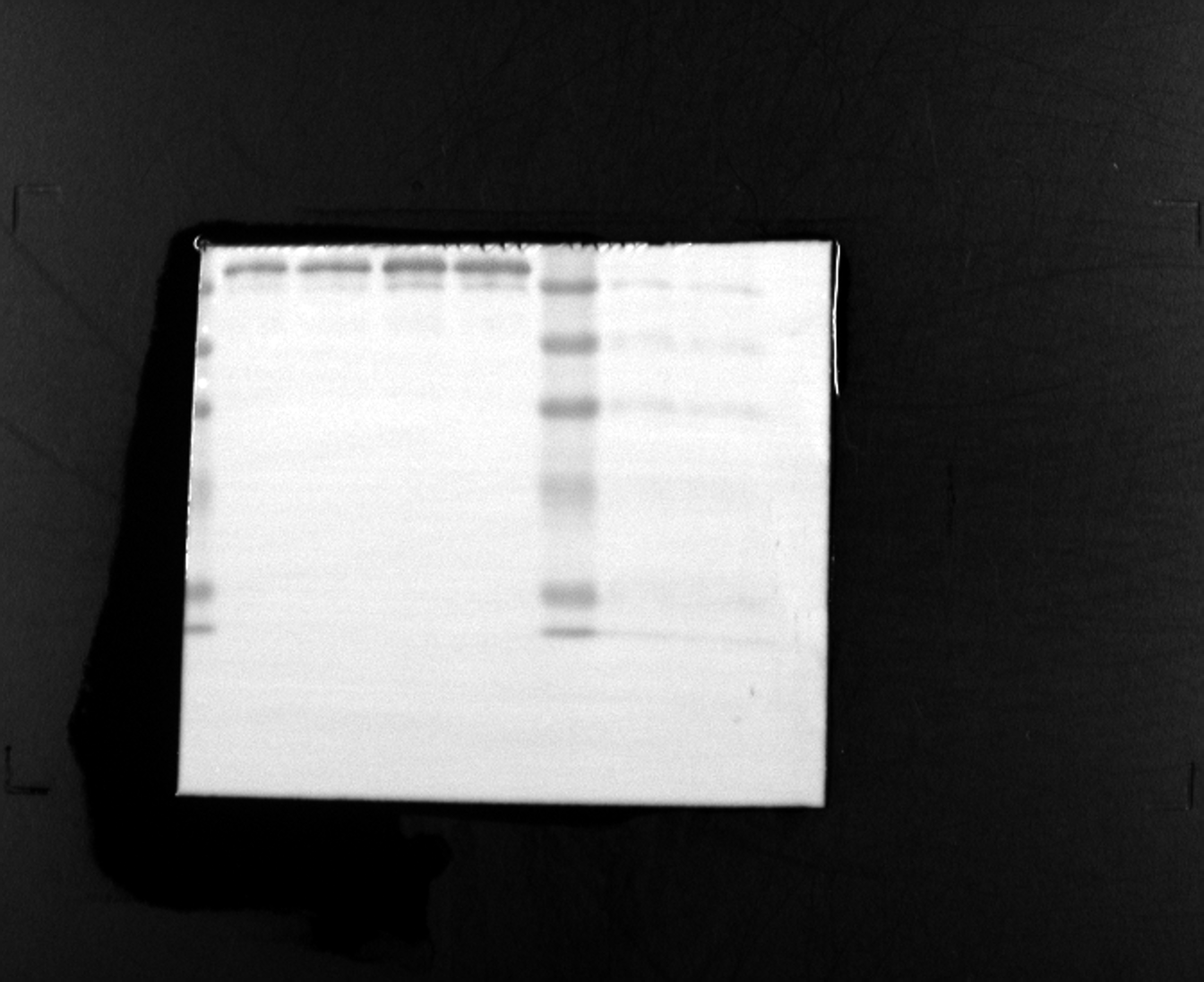

Supplement: Supplementary file 4 — Supplementary Material 4. [file 12964_2024_1770_MOESM4_ESM.zip › SENP3 TAM WB/WB-Figure4/B M0 M2 EndoIP/2023-02-16 ─┌╘┤IP shNC shSENP3 IRF4/INPUT IRF4/NPUT IRF4 0.7S M 0216.Tif]

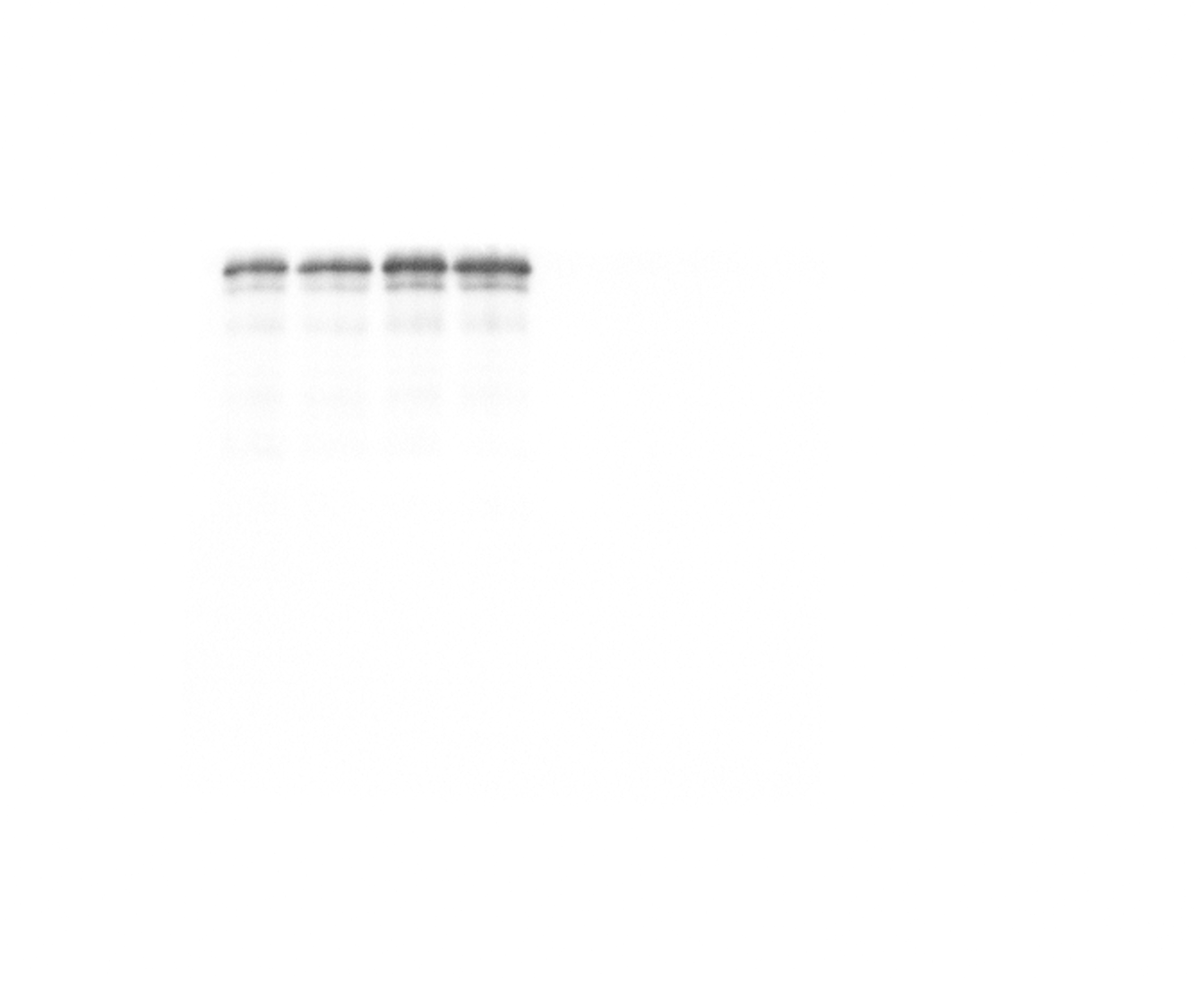

Supplement: Supplementary file 4 — Supplementary Material 4. [file 12964_2024_1770_MOESM4_ESM.zip › SENP3 TAM WB/WB-Figure4/B M0 M2 EndoIP/2023-02-16 ─┌╘┤IP shNC shSENP3 IRF4/INPUT IRF4/NPUT IRF4 0.8S 0216.Tif]

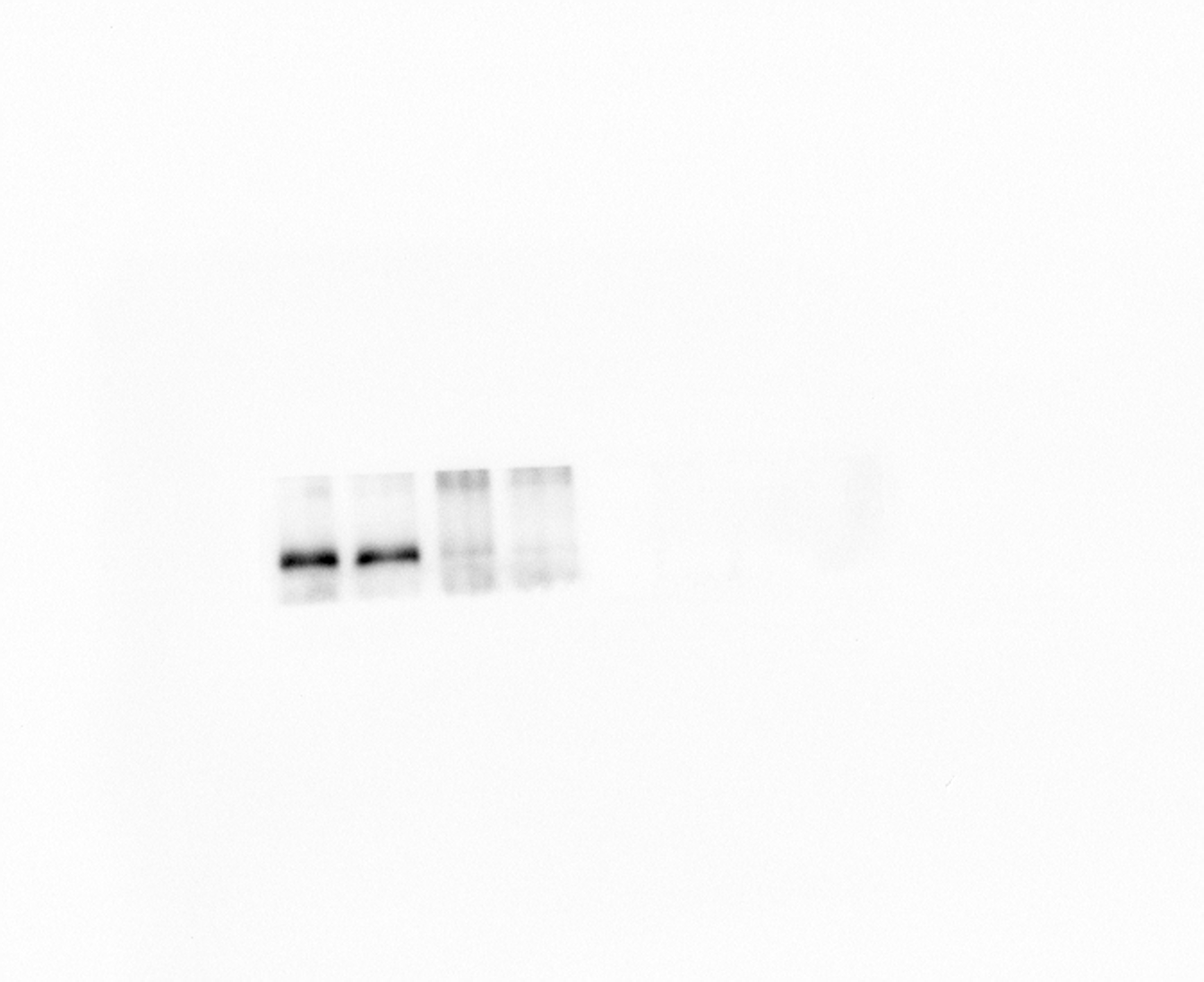

Supplement: Supplementary file 4 — Supplementary Material 4. [file 12964_2024_1770_MOESM4_ESM.zip › SENP3 TAM WB/WB-Figure4/B M0 M2 EndoIP/2023-02-16 ─┌╘┤IP shNC shSENP3 IRF4/INPUT SENP3/INPUT SENP3 10S 0216.Tif]

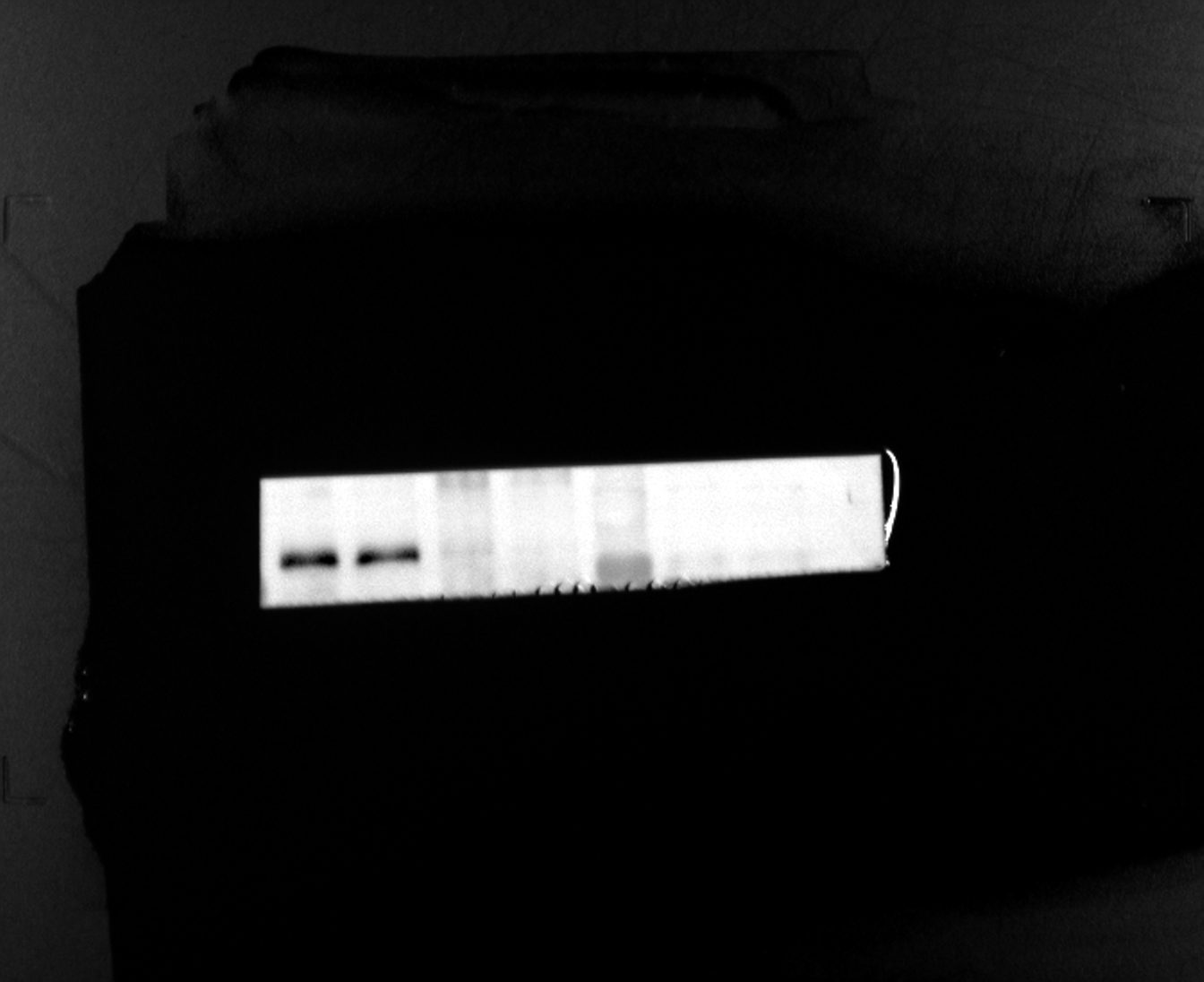

Supplement: Supplementary file 4 — Supplementary Material 4. [file 12964_2024_1770_MOESM4_ESM.zip › SENP3 TAM WB/WB-Figure4/B M0 M2 EndoIP/2023-02-16 ─┌╘┤IP shNC shSENP3 IRF4/INPUT SENP3/INPUT SENP3 10S M 0216.Tif]

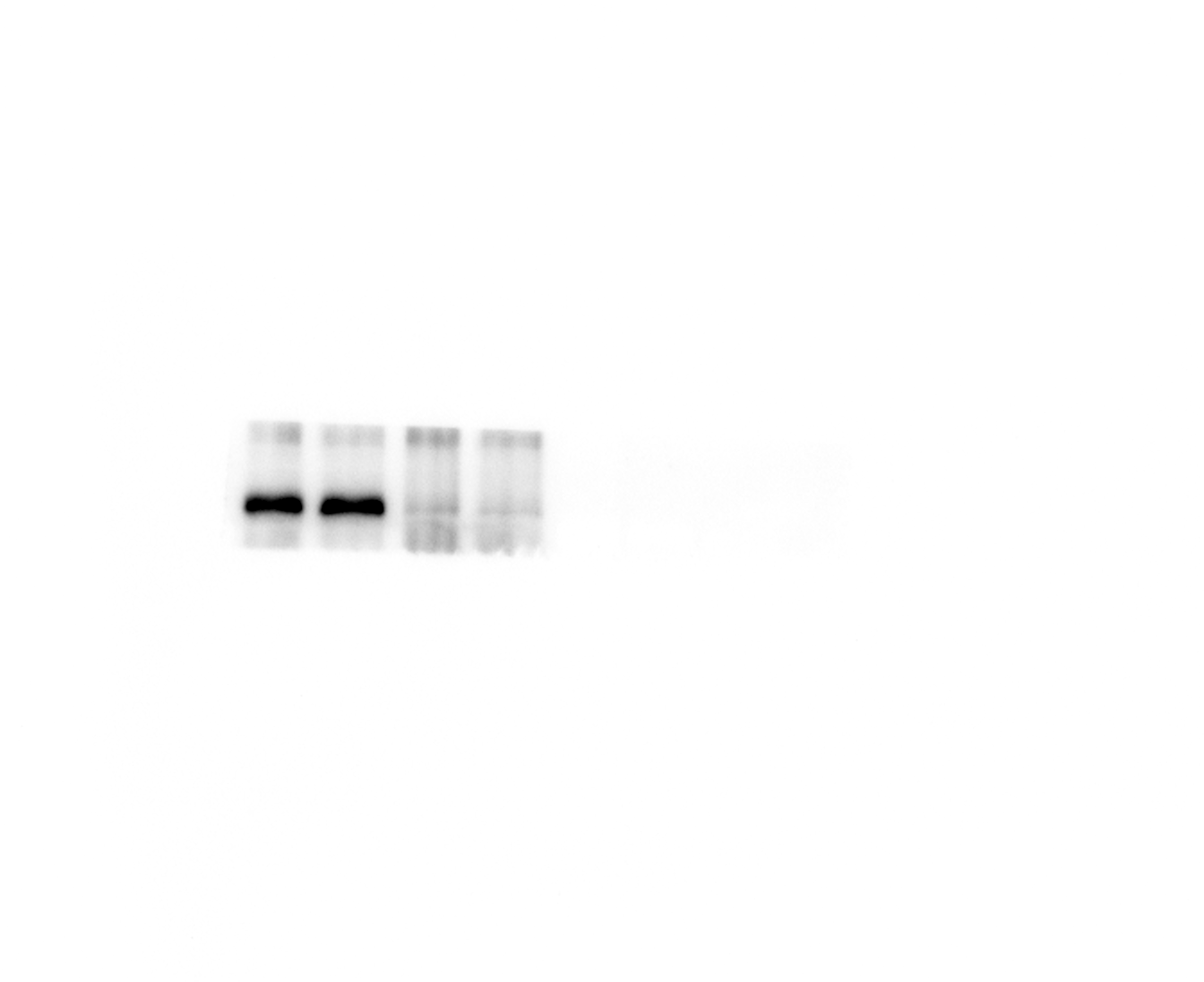

Supplement: Supplementary file 4 — Supplementary Material 4. [file 12964_2024_1770_MOESM4_ESM.zip › SENP3 TAM WB/WB-Figure4/B M0 M2 EndoIP/2023-02-16 ─┌╘┤IP shNC shSENP3 IRF4/INPUT SENP3/INPUT SENP3 11S 0216.Tif]

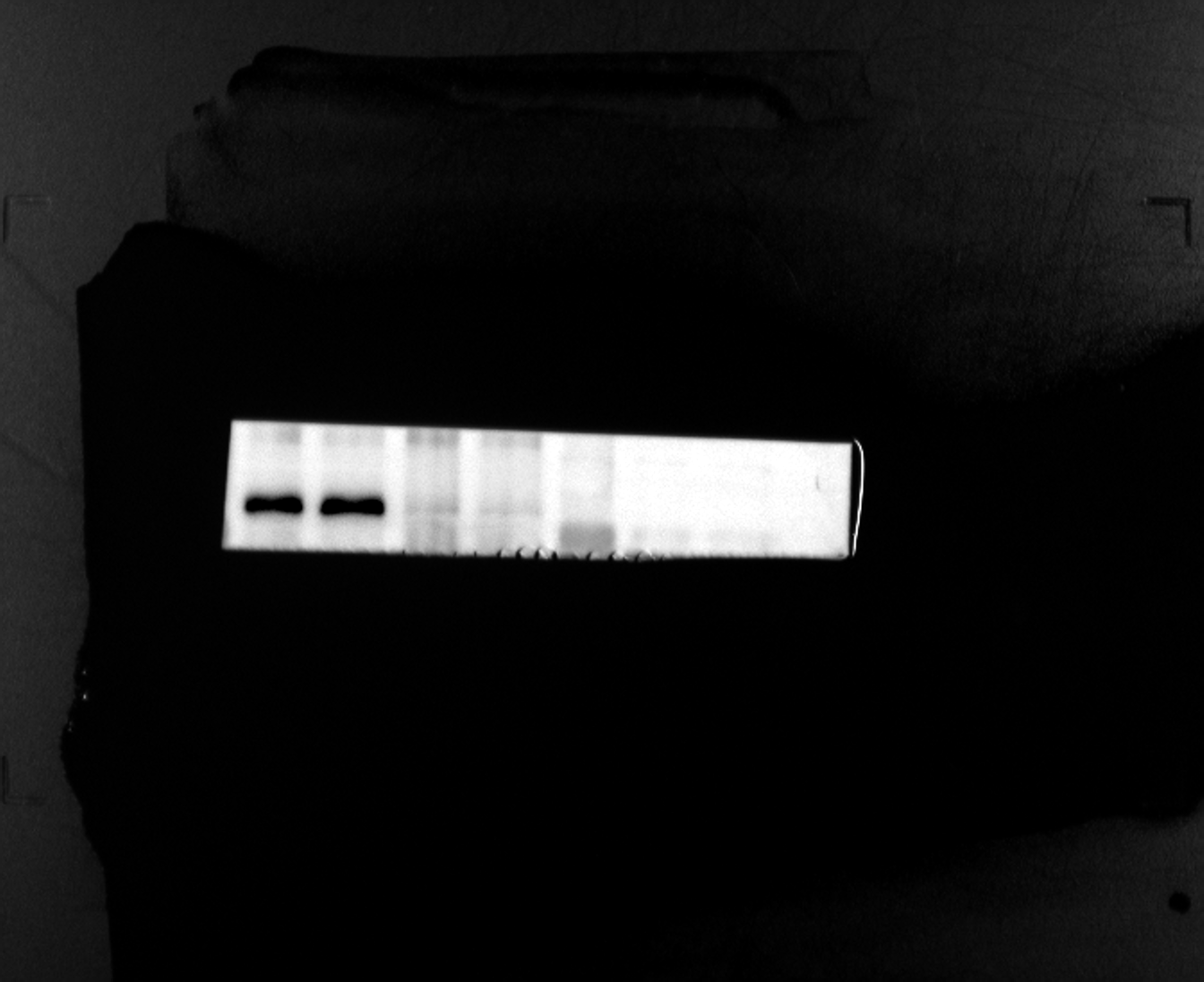

Supplement: Supplementary file 4 — Supplementary Material 4. [file 12964_2024_1770_MOESM4_ESM.zip › SENP3 TAM WB/WB-Figure4/B M0 M2 EndoIP/2023-02-16 ─┌╘┤IP shNC shSENP3 IRF4/INPUT SENP3/INPUT SENP3 11S M 0216.Tif]

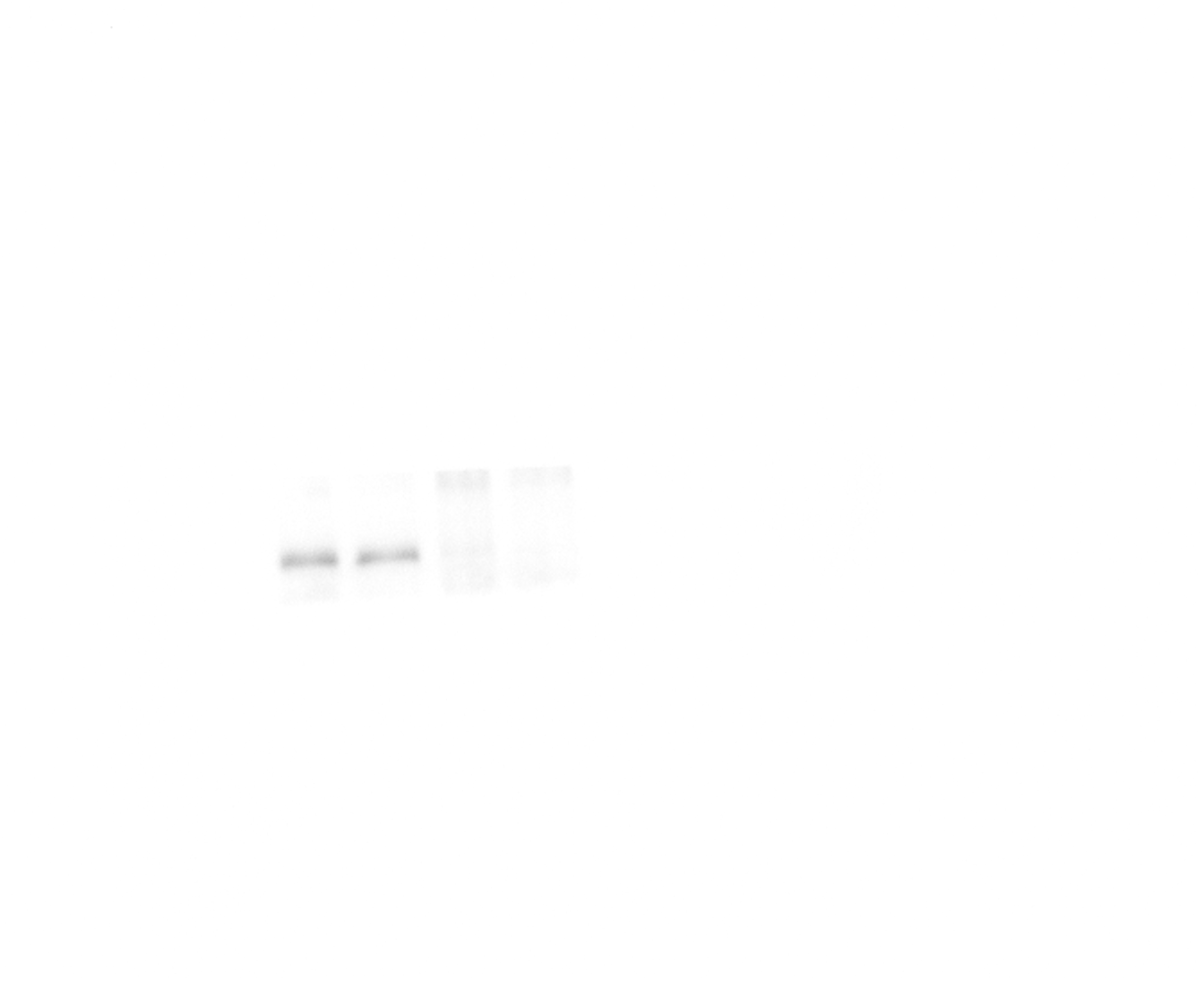

Supplement: Supplementary file 4 — Supplementary Material 4. [file 12964_2024_1770_MOESM4_ESM.zip › SENP3 TAM WB/WB-Figure4/B M0 M2 EndoIP/2023-02-16 ─┌╘┤IP shNC shSENP3 IRF4/INPUT SENP3/INPUT SENP3 3S 0216.Tif]

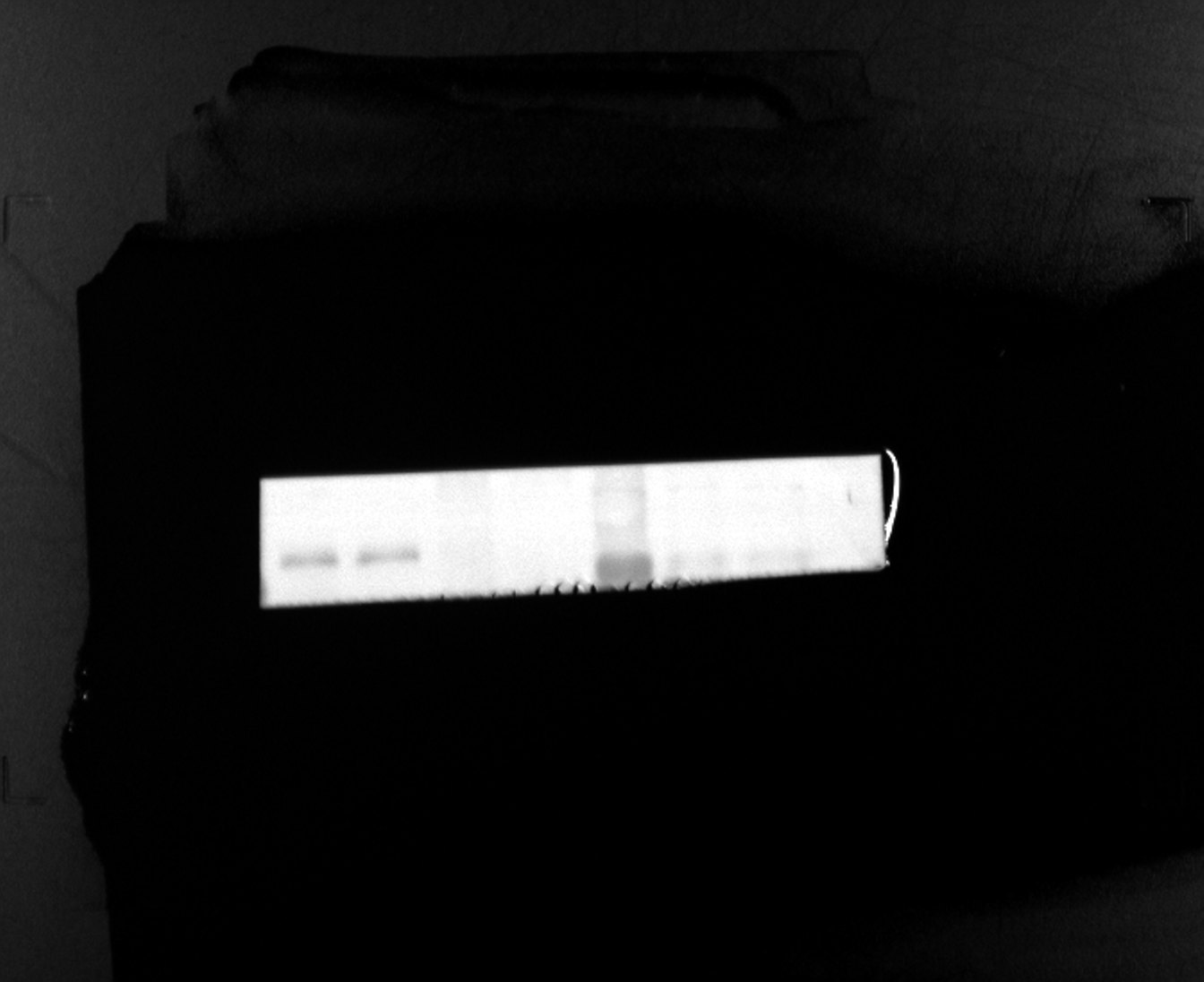

Supplement: Supplementary file 4 — Supplementary Material 4. [file 12964_2024_1770_MOESM4_ESM.zip › SENP3 TAM WB/WB-Figure4/B M0 M2 EndoIP/2023-02-16 ─┌╘┤IP shNC shSENP3 IRF4/INPUT SENP3/INPUT SENP3 3S M 0216.Tif]

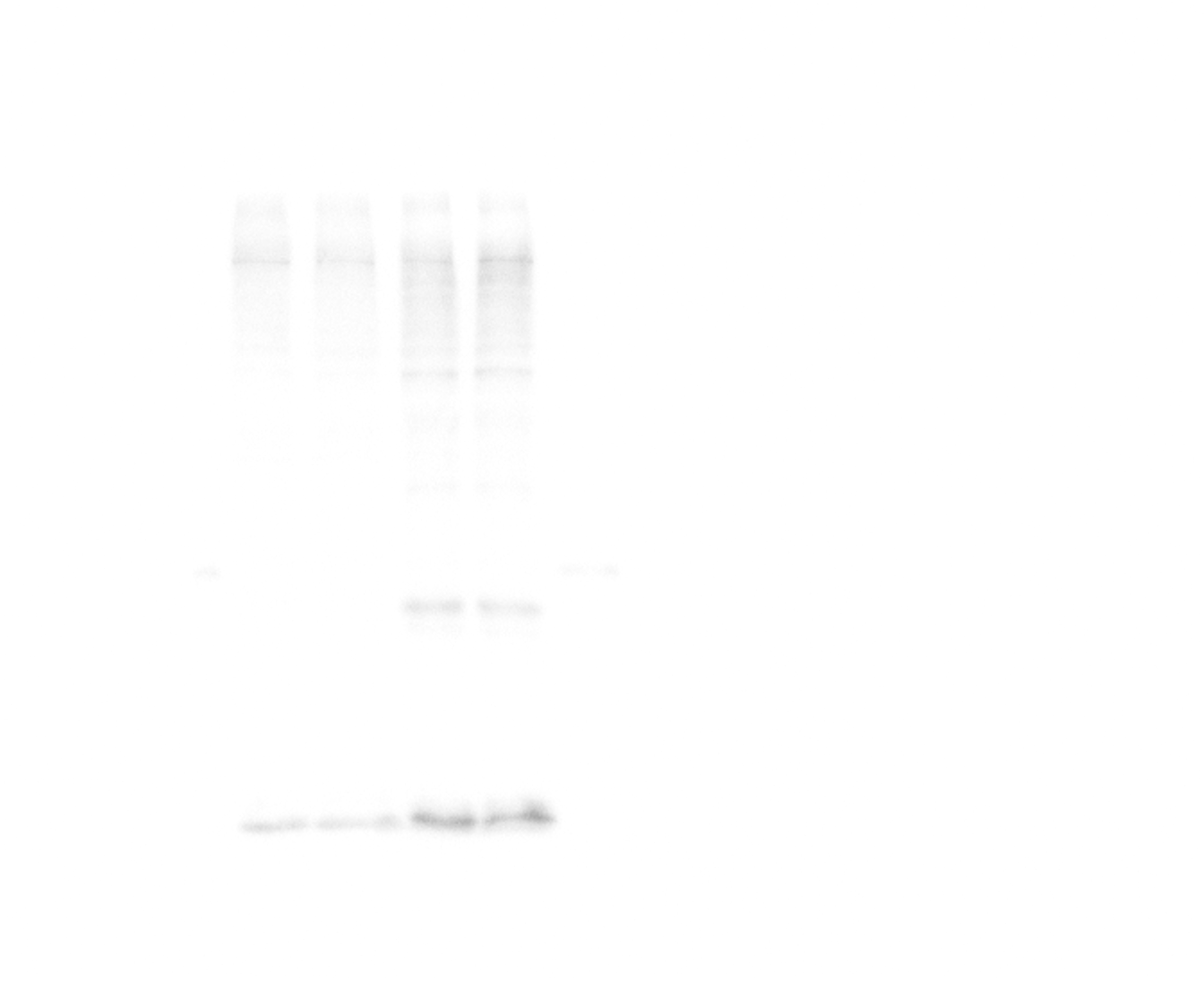

Supplement: Supplementary file 4 — Supplementary Material 4. [file 12964_2024_1770_MOESM4_ESM.zip › SENP3 TAM WB/WB-Figure4/B M0 M2 EndoIP/2023-02-16 ─┌╘┤IP shNC shSENP3 IRF4/INPUT SUMO23/INPUT SUMO23 0.3S 0216.Tif]

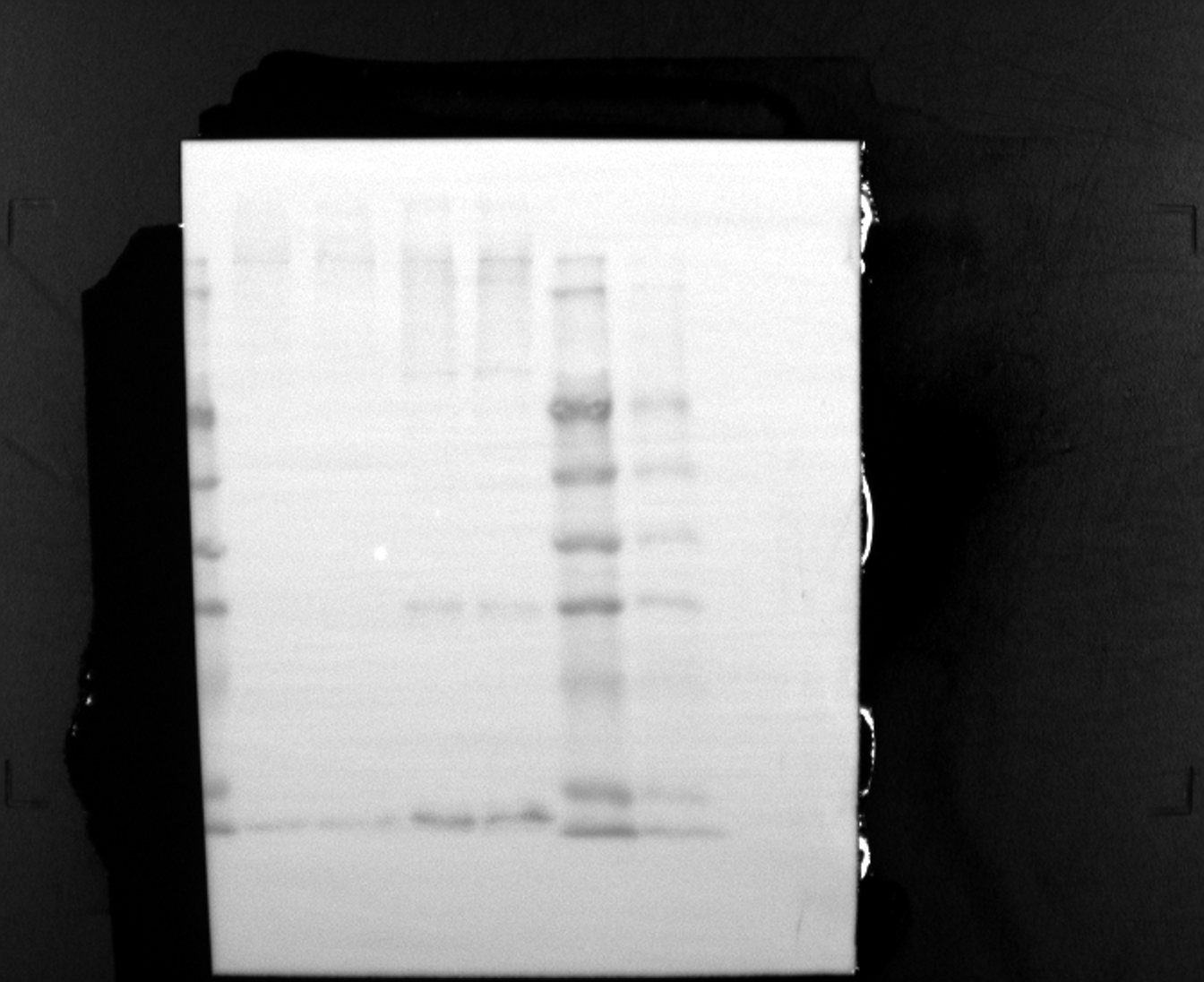

Supplement: Supplementary file 4 — Supplementary Material 4. [file 12964_2024_1770_MOESM4_ESM.zip › SENP3 TAM WB/WB-Figure4/B M0 M2 EndoIP/2023-02-16 ─┌╘┤IP shNC shSENP3 IRF4/INPUT SUMO23/INPUT SUMO23 0.3S M 0216.Tif]
