# Supplementary figures and images for: Suppression of SENP3 enhances macrophage alternative activation by mediating IRF4 de-SUMOylation in ESCC progression (part 2 of 2)
Source: Cell Commun Signal. 2024 Aug 9;22:395. doi: 10.1186/s12964-024-01770-z (PMC11312810; doi:10.1186/s12964-024-01770-z)

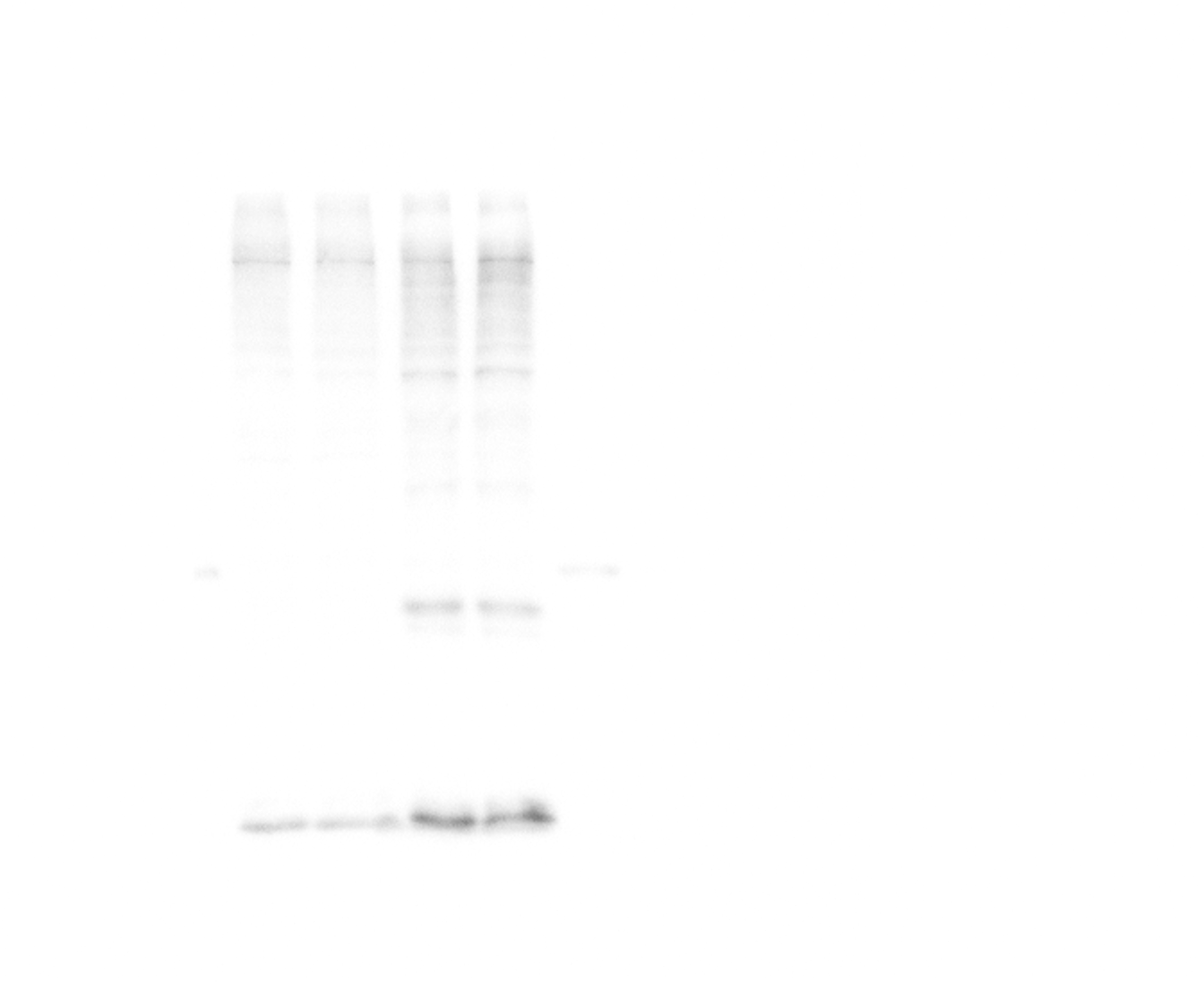

Supplement: Supplementary file 4 — Supplementary Material 4. [file 12964_2024_1770_MOESM4_ESM.zip › SENP3 TAM WB/WB-Figure4/B M0 M2 EndoIP/2023-02-16 ─┌╘┤IP shNC shSENP3 IRF4/INPUT SUMO23/INPUT SUMO23 0.5S 0216.Tif]

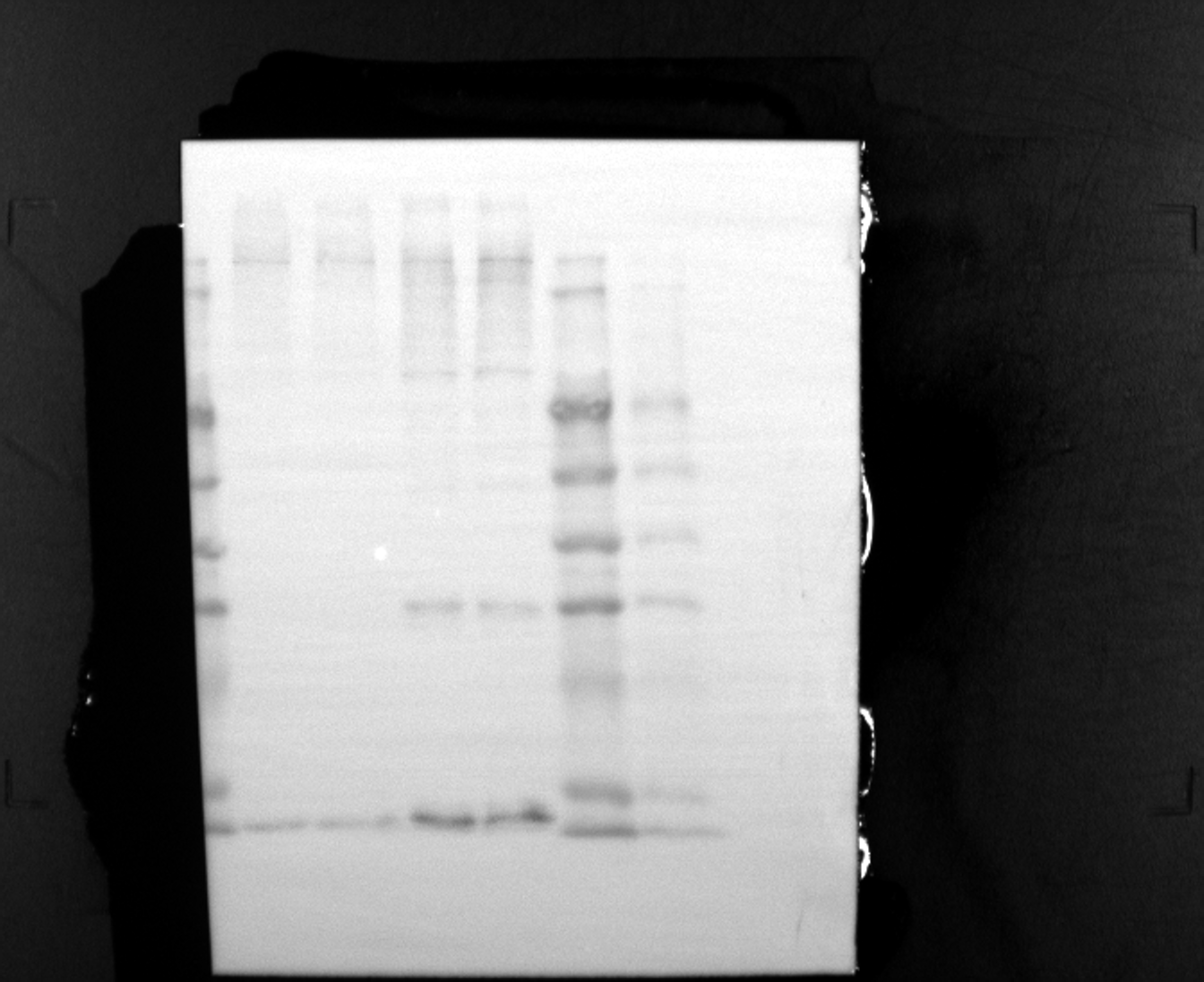

Supplement: Supplementary file 4 — Supplementary Material 4. [file 12964_2024_1770_MOESM4_ESM.zip › SENP3 TAM WB/WB-Figure4/B M0 M2 EndoIP/2023-02-16 ─┌╘┤IP shNC shSENP3 IRF4/INPUT SUMO23/INPUT SUMO23 0.5S M 0216.Tif]

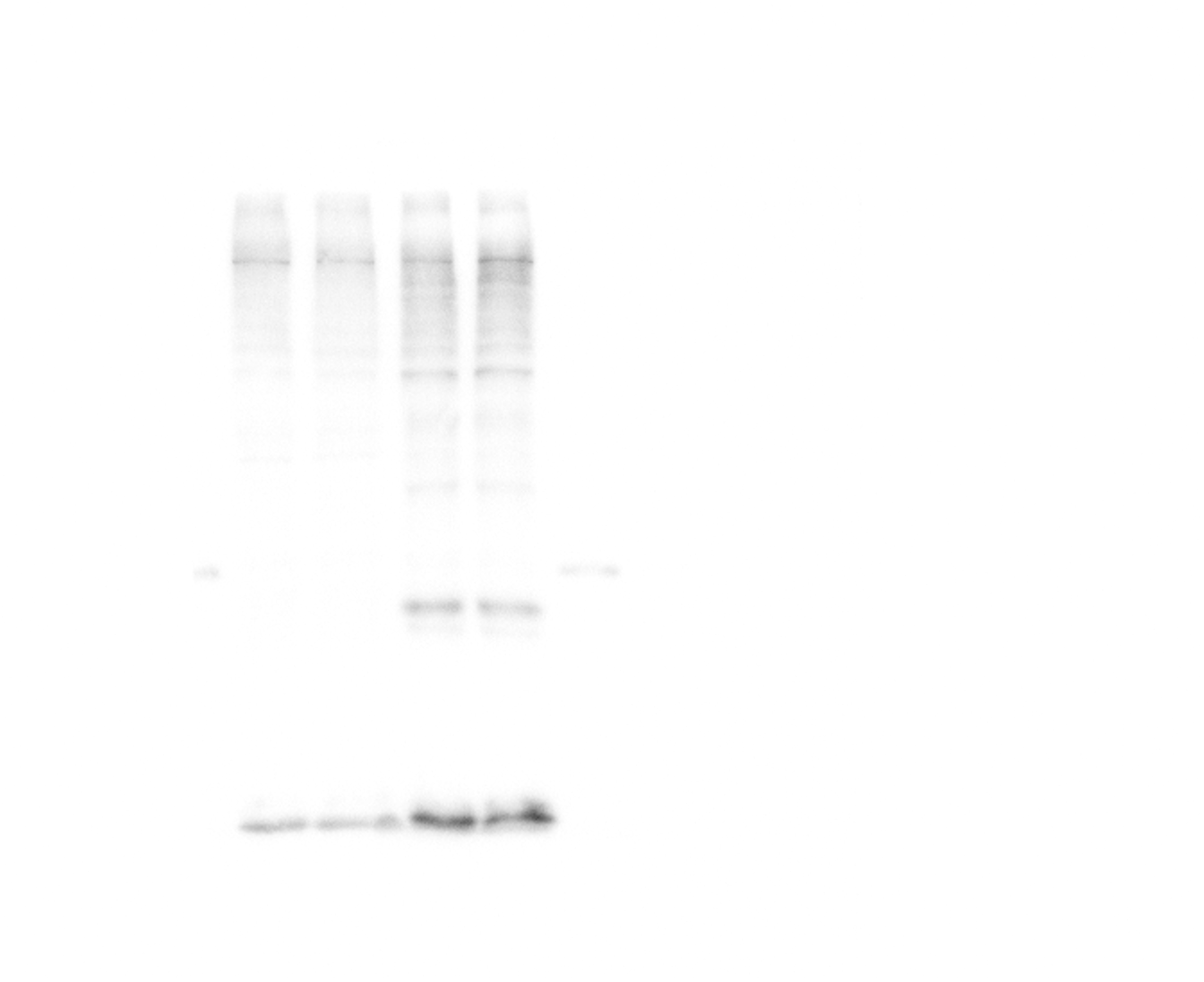

Supplement: Supplementary file 4 — Supplementary Material 4. [file 12964_2024_1770_MOESM4_ESM.zip › SENP3 TAM WB/WB-Figure4/B M0 M2 EndoIP/2023-02-16 ─┌╘┤IP shNC shSENP3 IRF4/INPUT SUMO23/INPUT SUMO23 0.7S 0216.Tif]

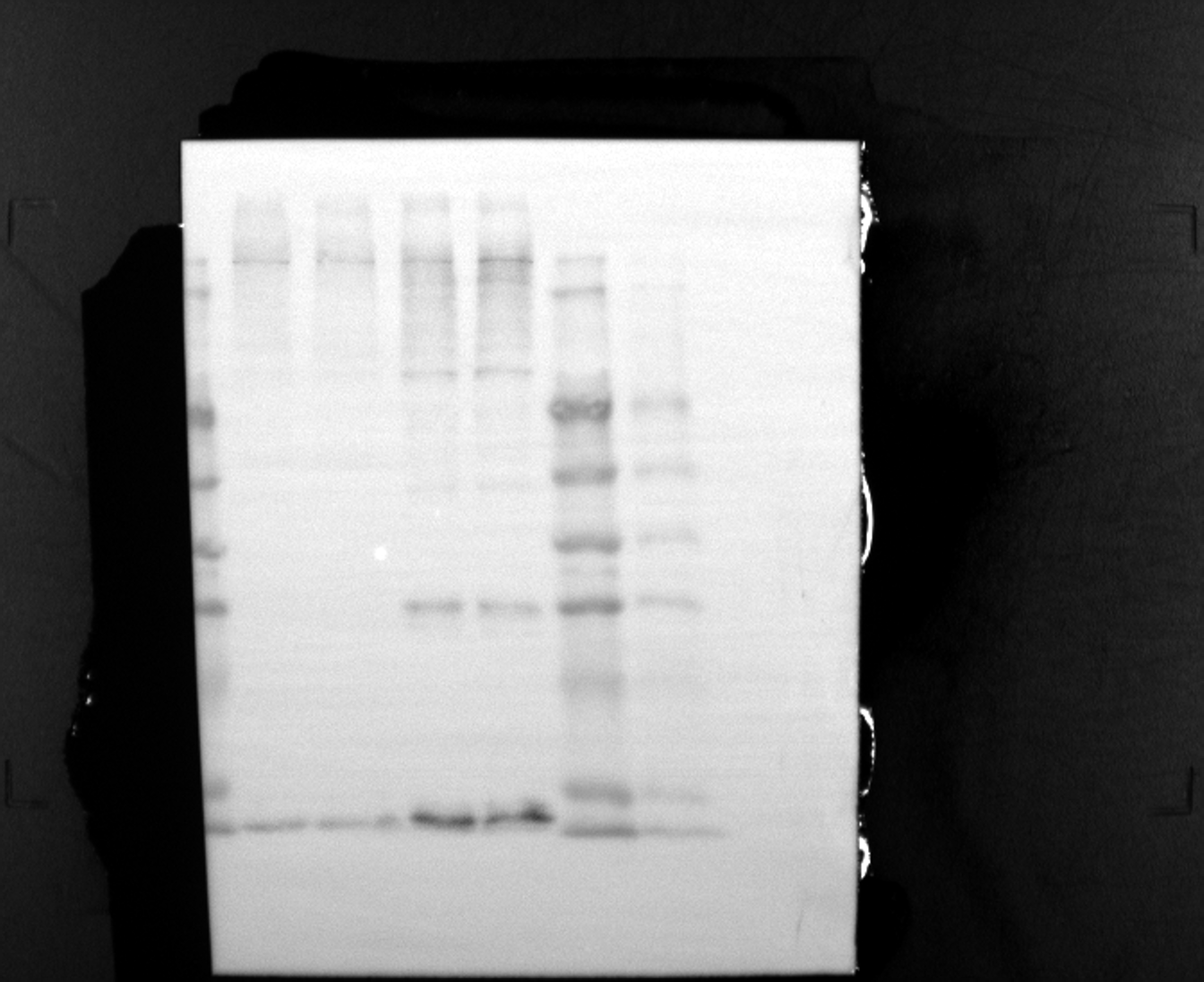

Supplement: Supplementary file 4 — Supplementary Material 4. [file 12964_2024_1770_MOESM4_ESM.zip › SENP3 TAM WB/WB-Figure4/B M0 M2 EndoIP/2023-02-16 ─┌╘┤IP shNC shSENP3 IRF4/INPUT SUMO23/INPUT SUMO23 0.7S M 0216.Tif]

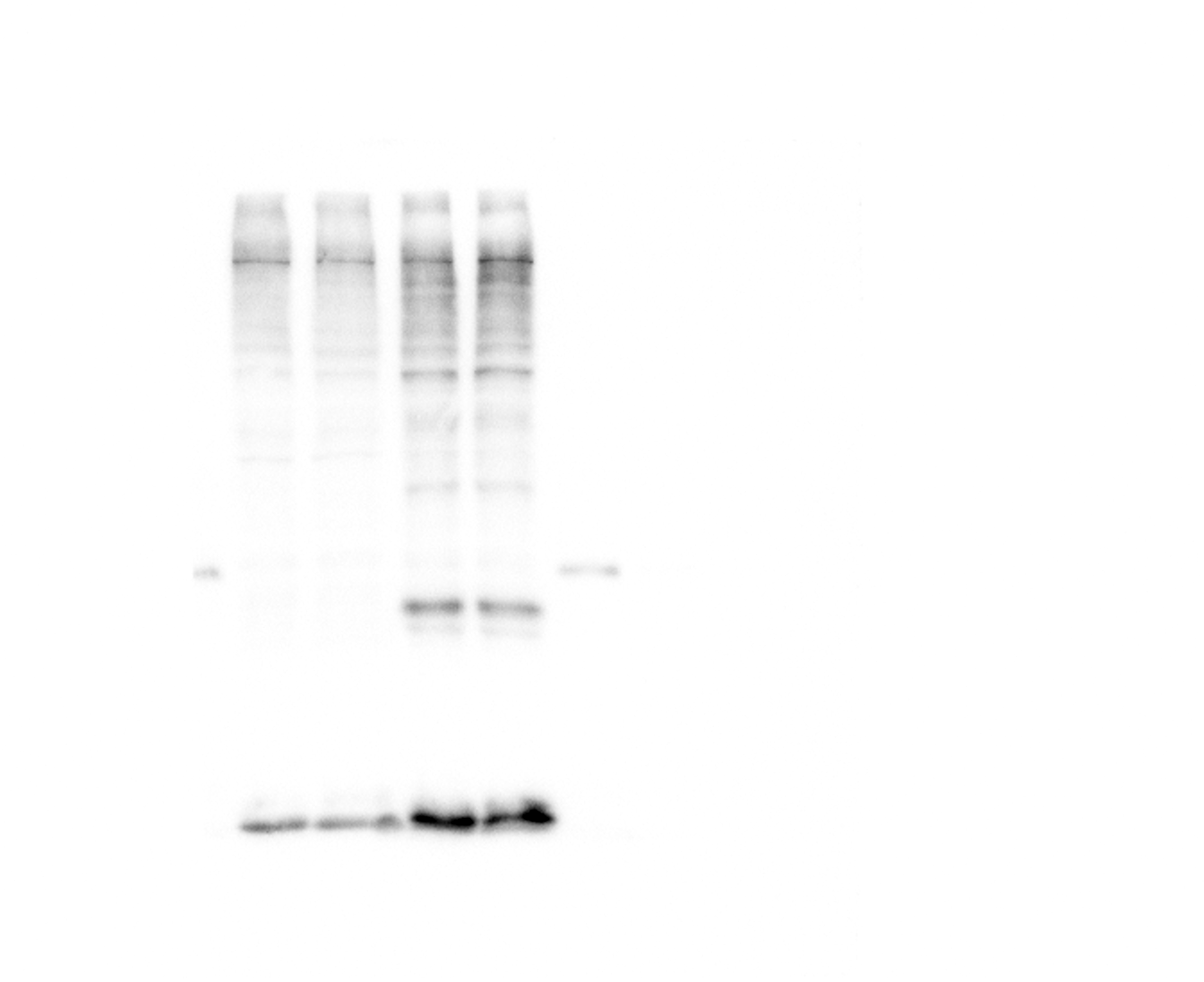

Supplement: Supplementary file 4 — Supplementary Material 4. [file 12964_2024_1770_MOESM4_ESM.zip › SENP3 TAM WB/WB-Figure4/B M0 M2 EndoIP/2023-02-16 ─┌╘┤IP shNC shSENP3 IRF4/INPUT SUMO23/INPUT SUMO23 1.5S 0216.Tif]

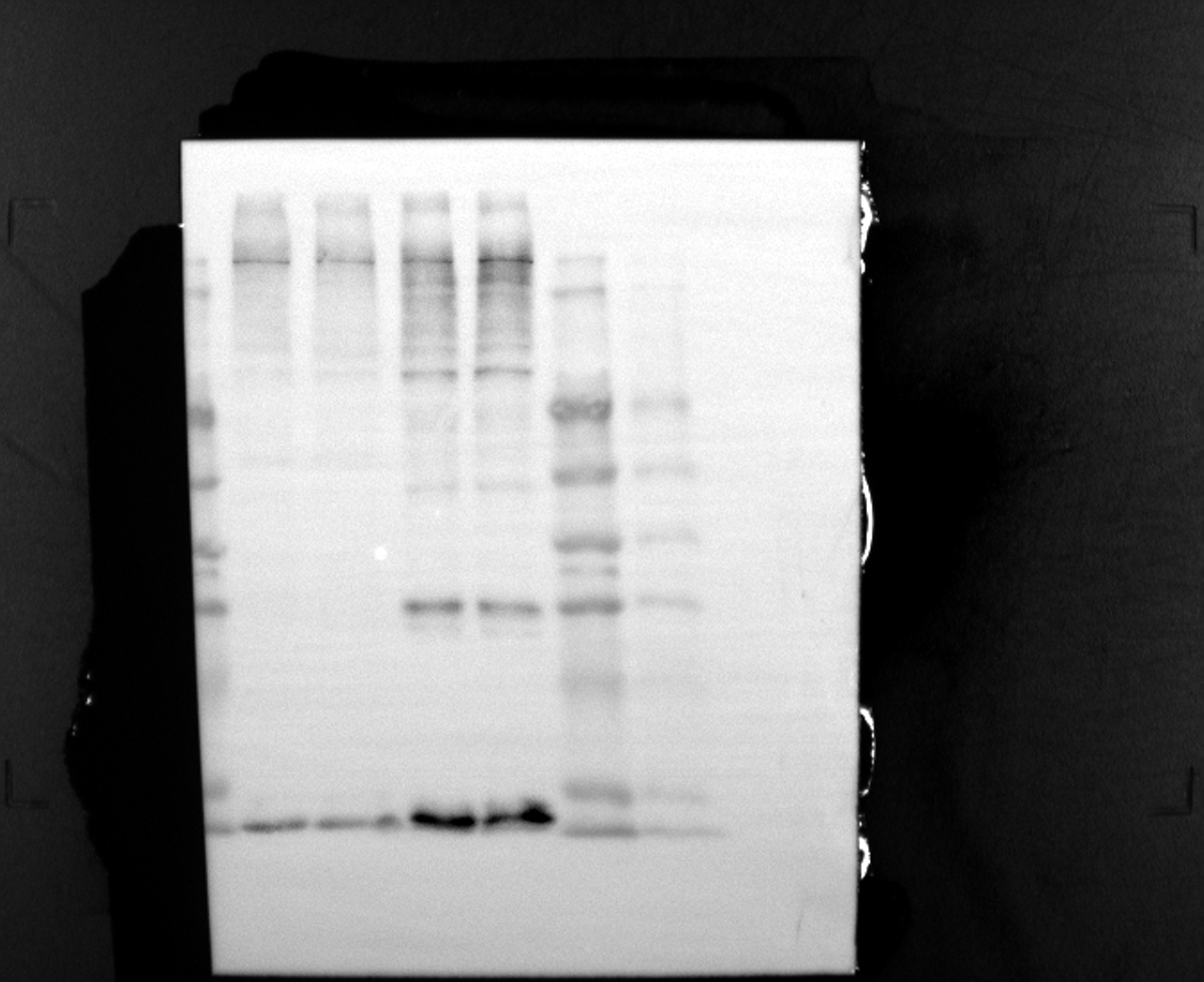

Supplement: Supplementary file 4 — Supplementary Material 4. [file 12964_2024_1770_MOESM4_ESM.zip › SENP3 TAM WB/WB-Figure4/B M0 M2 EndoIP/2023-02-16 ─┌╘┤IP shNC shSENP3 IRF4/INPUT SUMO23/INPUT SUMO23 1.5S M 0216.Tif]

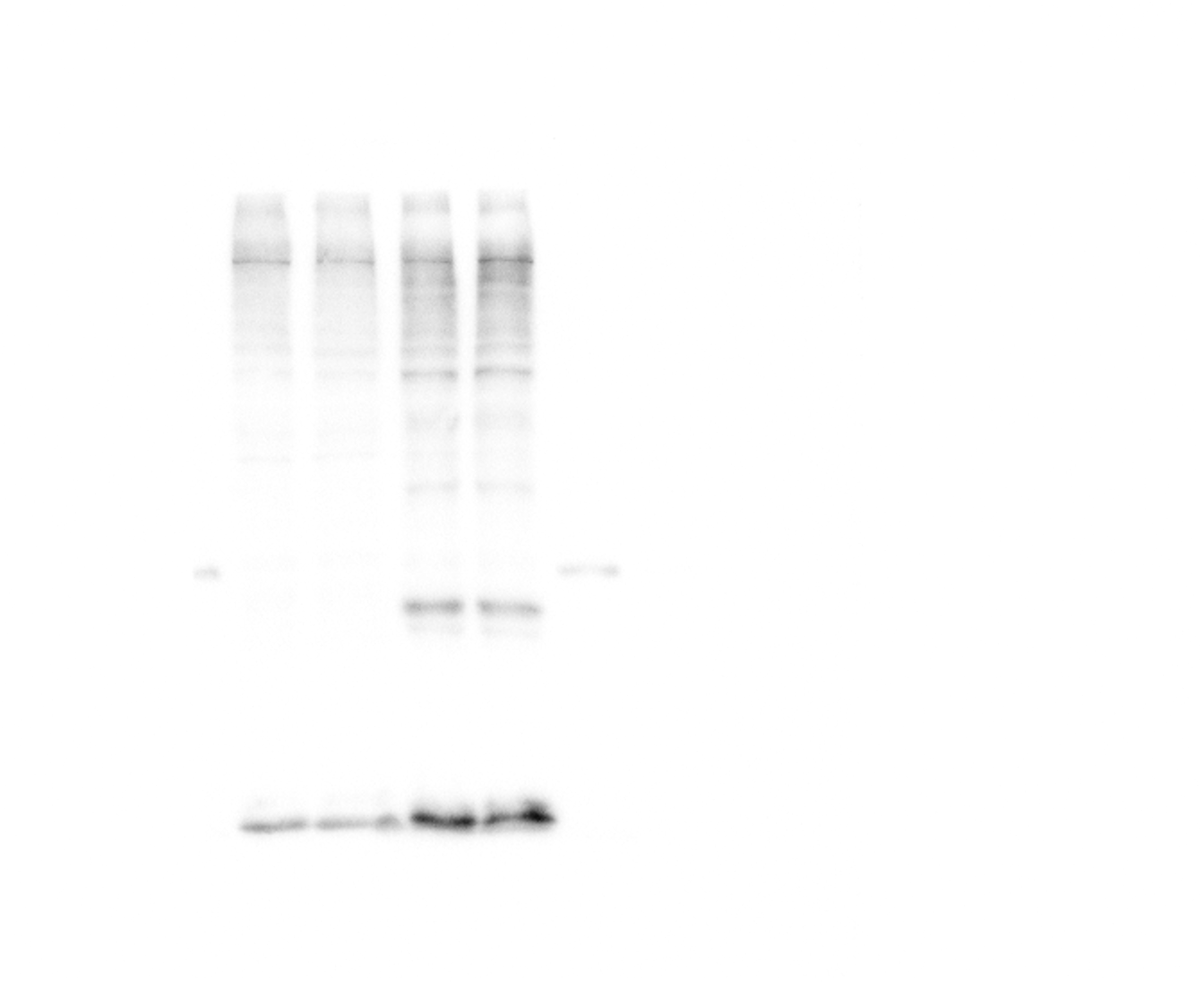

Supplement: Supplementary file 4 — Supplementary Material 4. [file 12964_2024_1770_MOESM4_ESM.zip › SENP3 TAM WB/WB-Figure4/B M0 M2 EndoIP/2023-02-16 ─┌╘┤IP shNC shSENP3 IRF4/INPUT SUMO23/INPUT SUMO23 1S 0216.Tif]

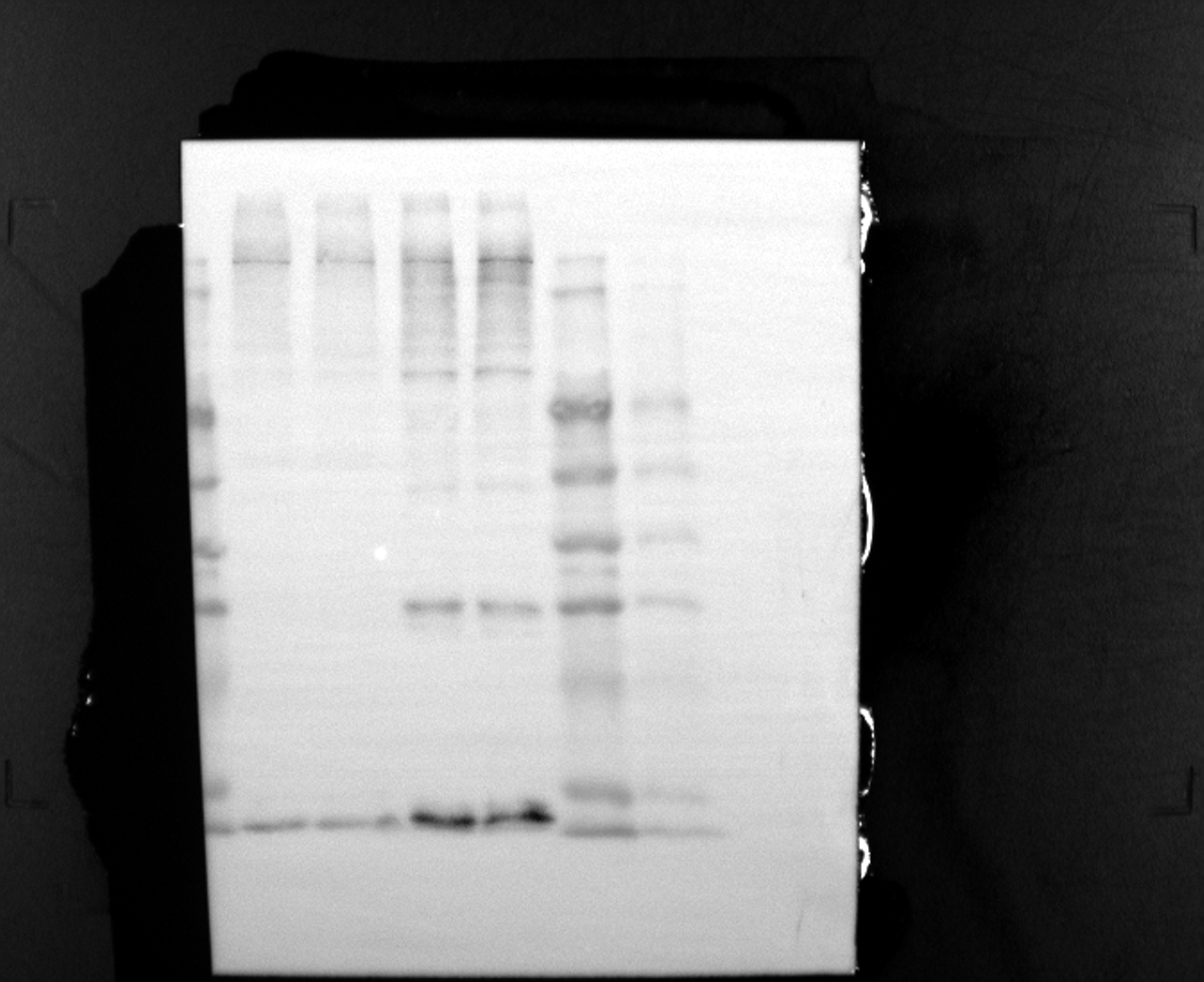

Supplement: Supplementary file 4 — Supplementary Material 4. [file 12964_2024_1770_MOESM4_ESM.zip › SENP3 TAM WB/WB-Figure4/B M0 M2 EndoIP/2023-02-16 ─┌╘┤IP shNC shSENP3 IRF4/INPUT SUMO23/INPUT SUMO23 1S M 0216.Tif]

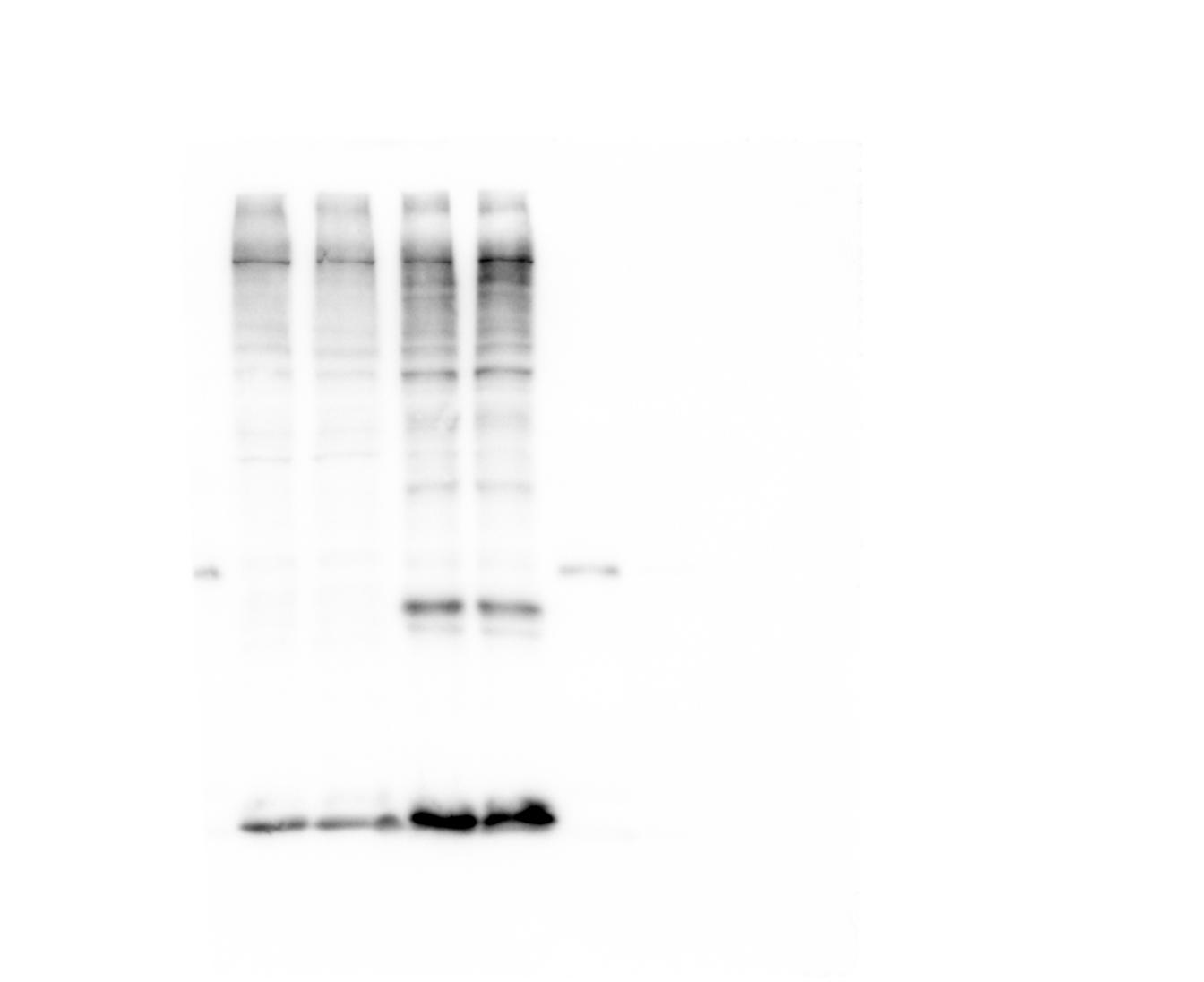

Supplement: Supplementary file 4 — Supplementary Material 4. [file 12964_2024_1770_MOESM4_ESM.zip › SENP3 TAM WB/WB-Figure4/B M0 M2 EndoIP/2023-02-16 ─┌╘┤IP shNC shSENP3 IRF4/INPUT SUMO23/INPUT SUMO23 7.3S 0216.Tif]

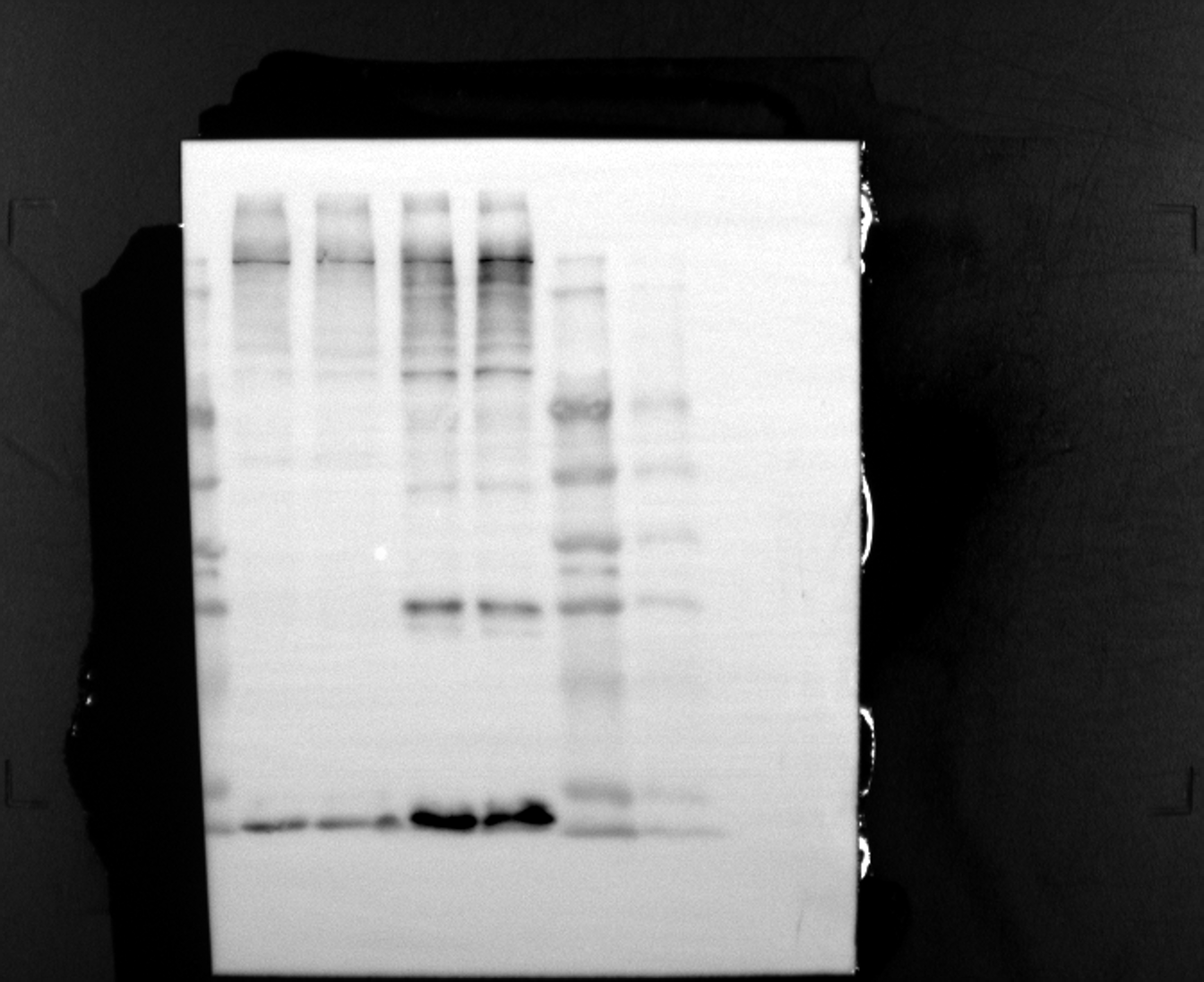

Supplement: Supplementary file 4 — Supplementary Material 4. [file 12964_2024_1770_MOESM4_ESM.zip › SENP3 TAM WB/WB-Figure4/B M0 M2 EndoIP/2023-02-16 ─┌╘┤IP shNC shSENP3 IRF4/INPUT SUMO23/INPUT SUMO23 7.3S M 0216.Tif]

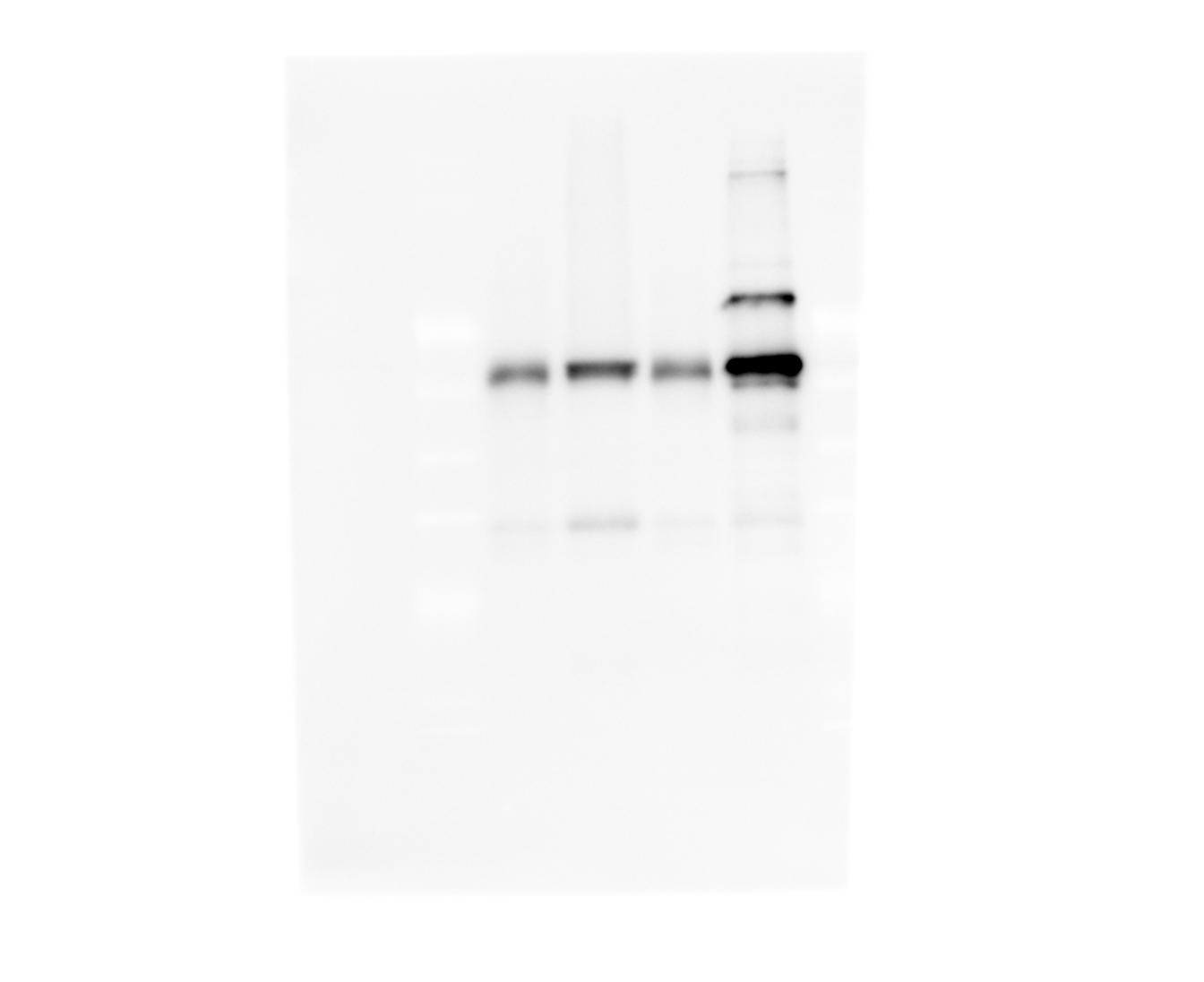

Supplement: Supplementary file 4 — Supplementary Material 4. [file 12964_2024_1770_MOESM4_ESM.zip › SENP3 TAM WB/WB-Figure4/B M0 M2 EndoIP/2023-02-16 ─┌╘┤IP shNC shSENP3 IRF4/IP IRF4/IP IRF4 1.9S 0216.Tif]

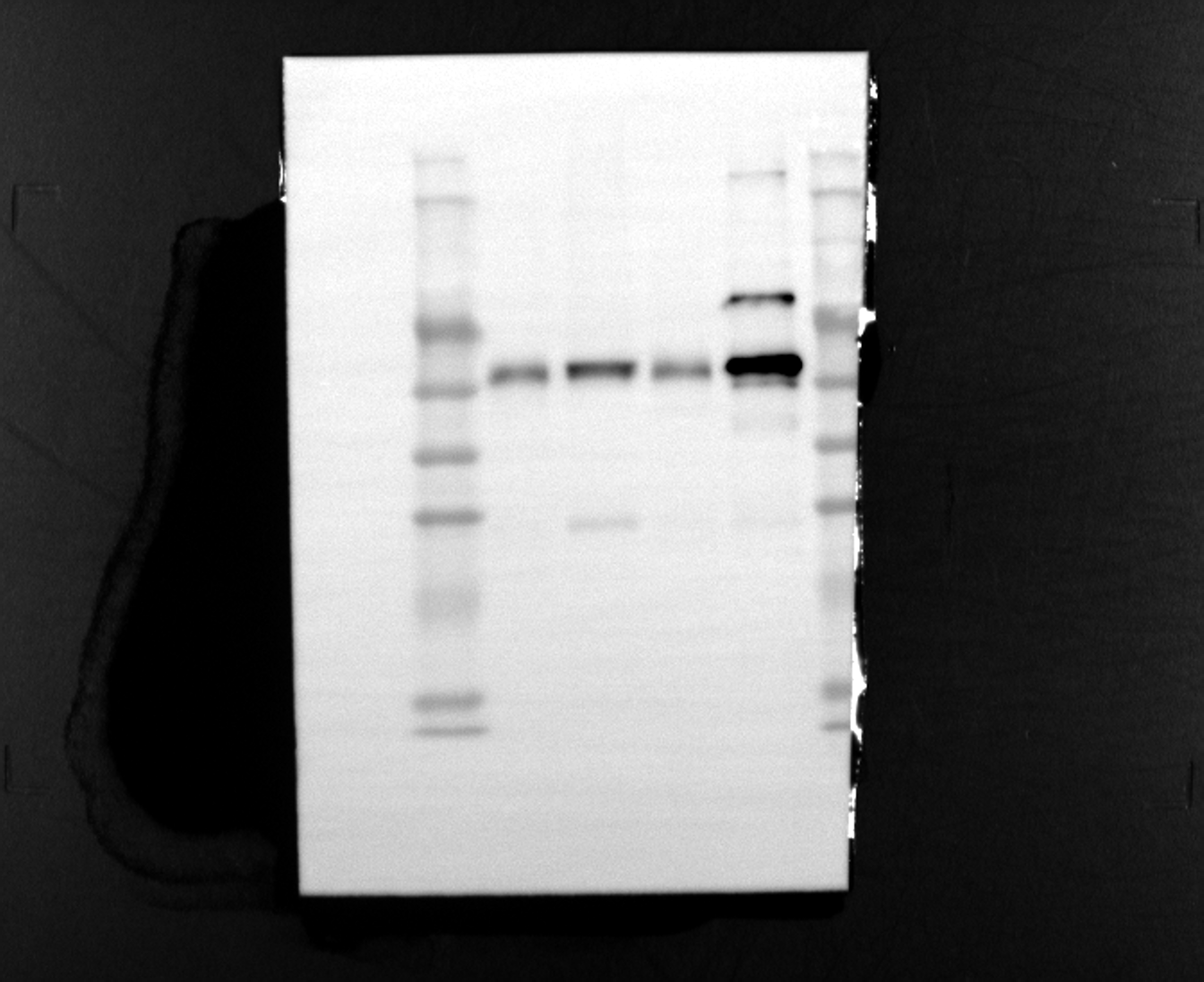

Supplement: Supplementary file 4 — Supplementary Material 4. [file 12964_2024_1770_MOESM4_ESM.zip › SENP3 TAM WB/WB-Figure4/B M0 M2 EndoIP/2023-02-16 ─┌╘┤IP shNC shSENP3 IRF4/IP IRF4/IP IRF4 1.9S M 0216.Tif]

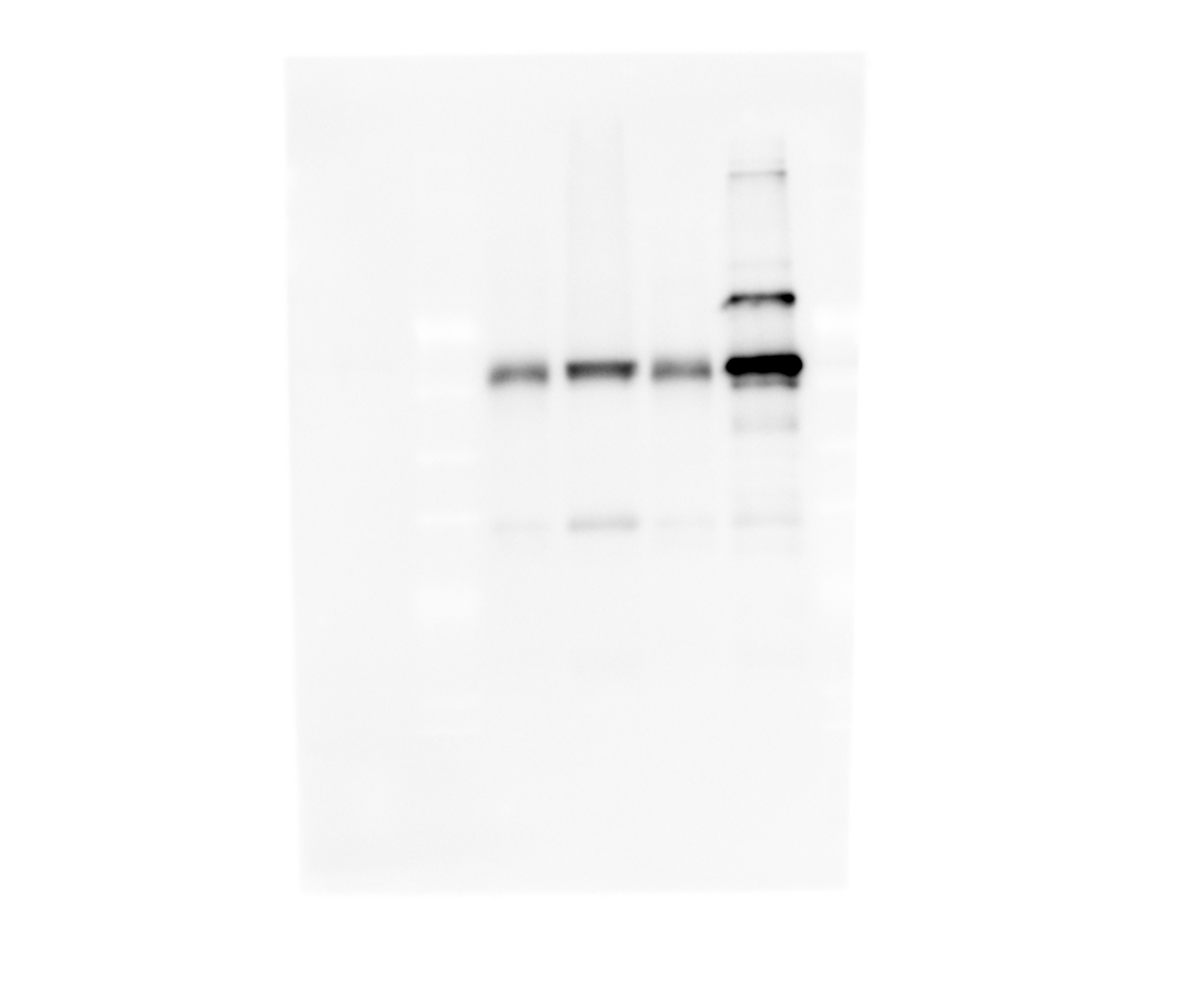

Supplement: Supplementary file 4 — Supplementary Material 4. [file 12964_2024_1770_MOESM4_ESM.zip › SENP3 TAM WB/WB-Figure4/B M0 M2 EndoIP/2023-02-16 ─┌╘┤IP shNC shSENP3 IRF4/IP IRF4/IP IRF4 2.1S 0216.Tif]

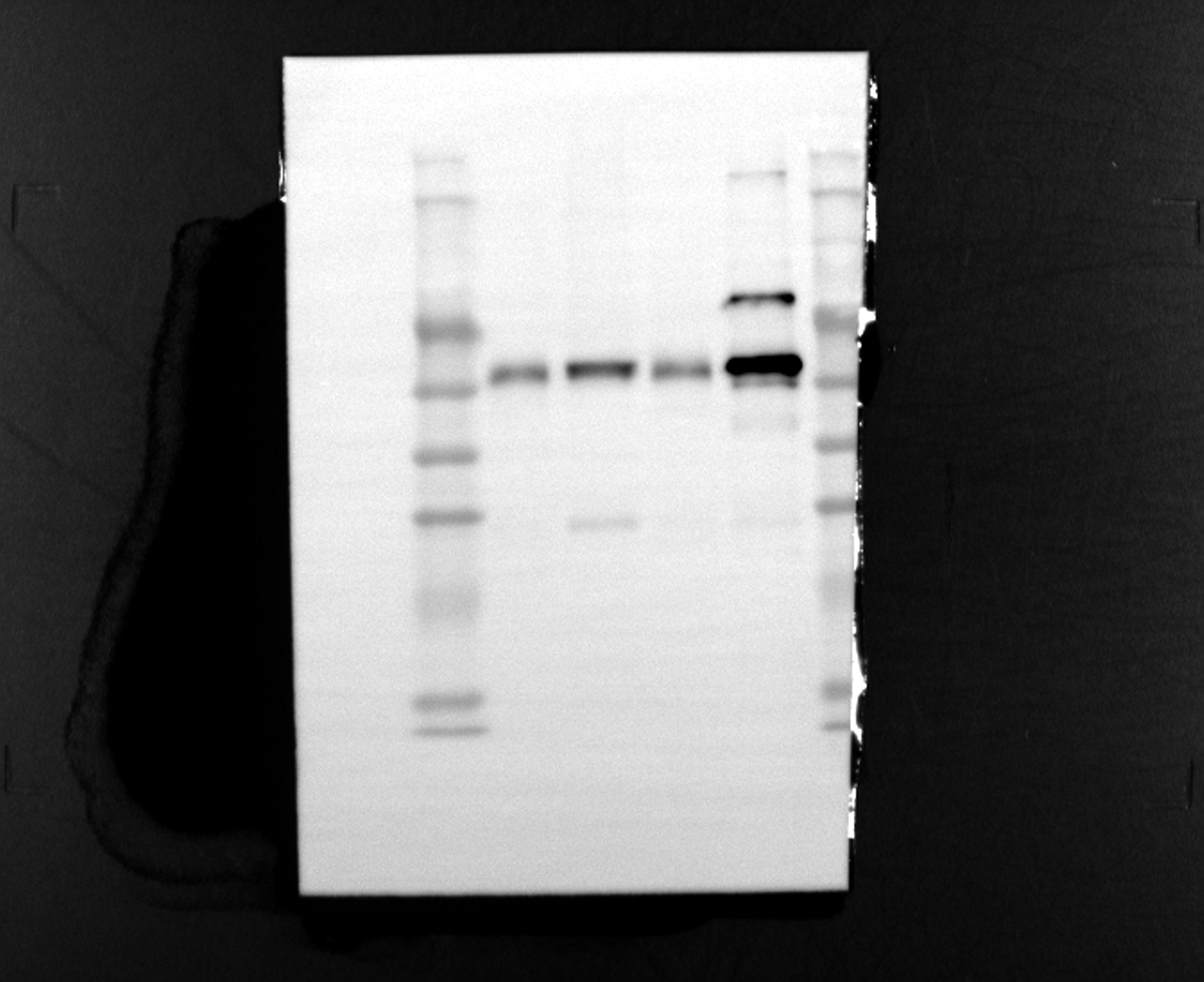

Supplement: Supplementary file 4 — Supplementary Material 4. [file 12964_2024_1770_MOESM4_ESM.zip › SENP3 TAM WB/WB-Figure4/B M0 M2 EndoIP/2023-02-16 ─┌╘┤IP shNC shSENP3 IRF4/IP IRF4/IP IRF4 2.1S M 0216.Tif]

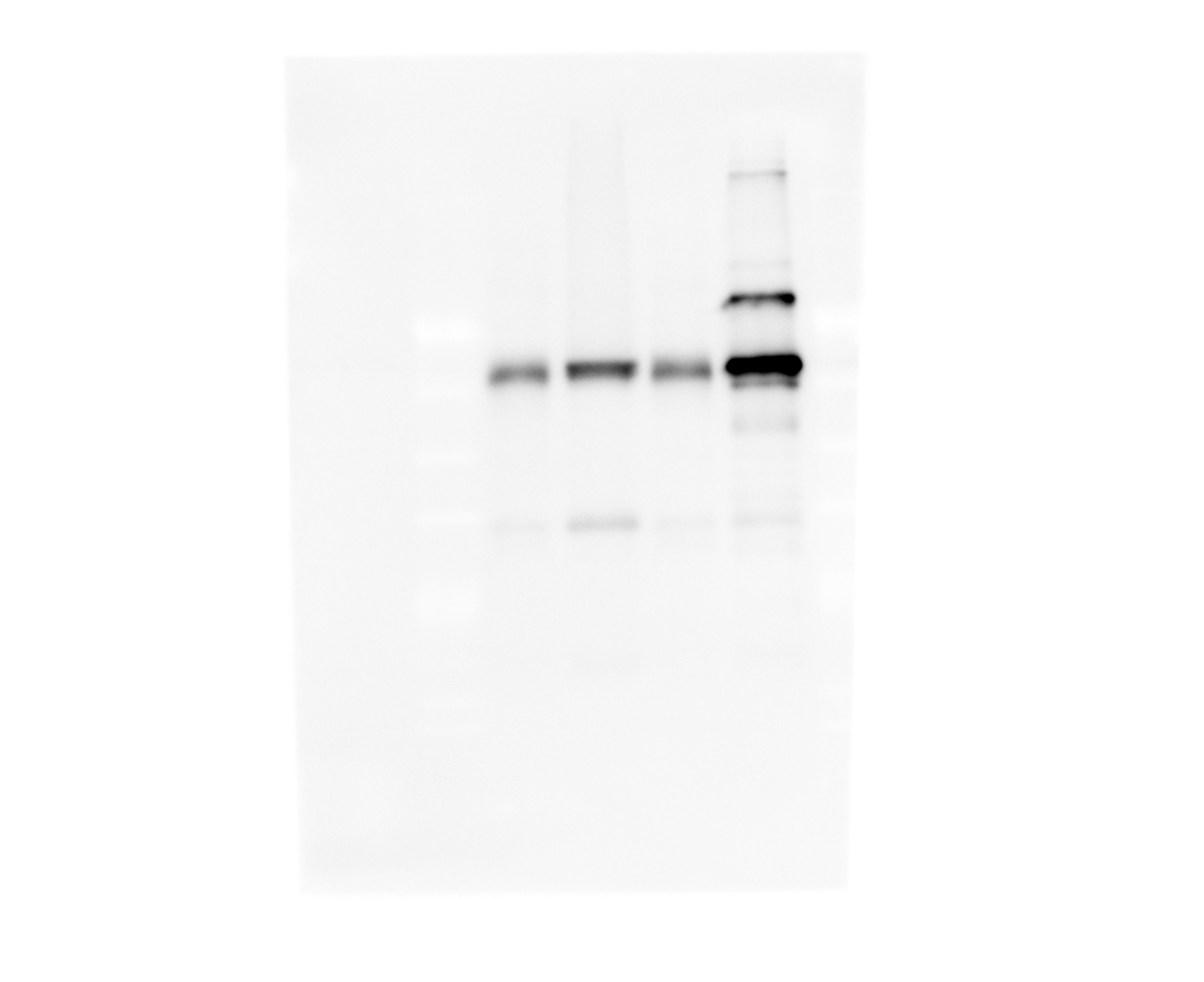

Supplement: Supplementary file 4 — Supplementary Material 4. [file 12964_2024_1770_MOESM4_ESM.zip › SENP3 TAM WB/WB-Figure4/B M0 M2 EndoIP/2023-02-16 ─┌╘┤IP shNC shSENP3 IRF4/IP IRF4/IP IRF4 2.2S 0216.Tif]

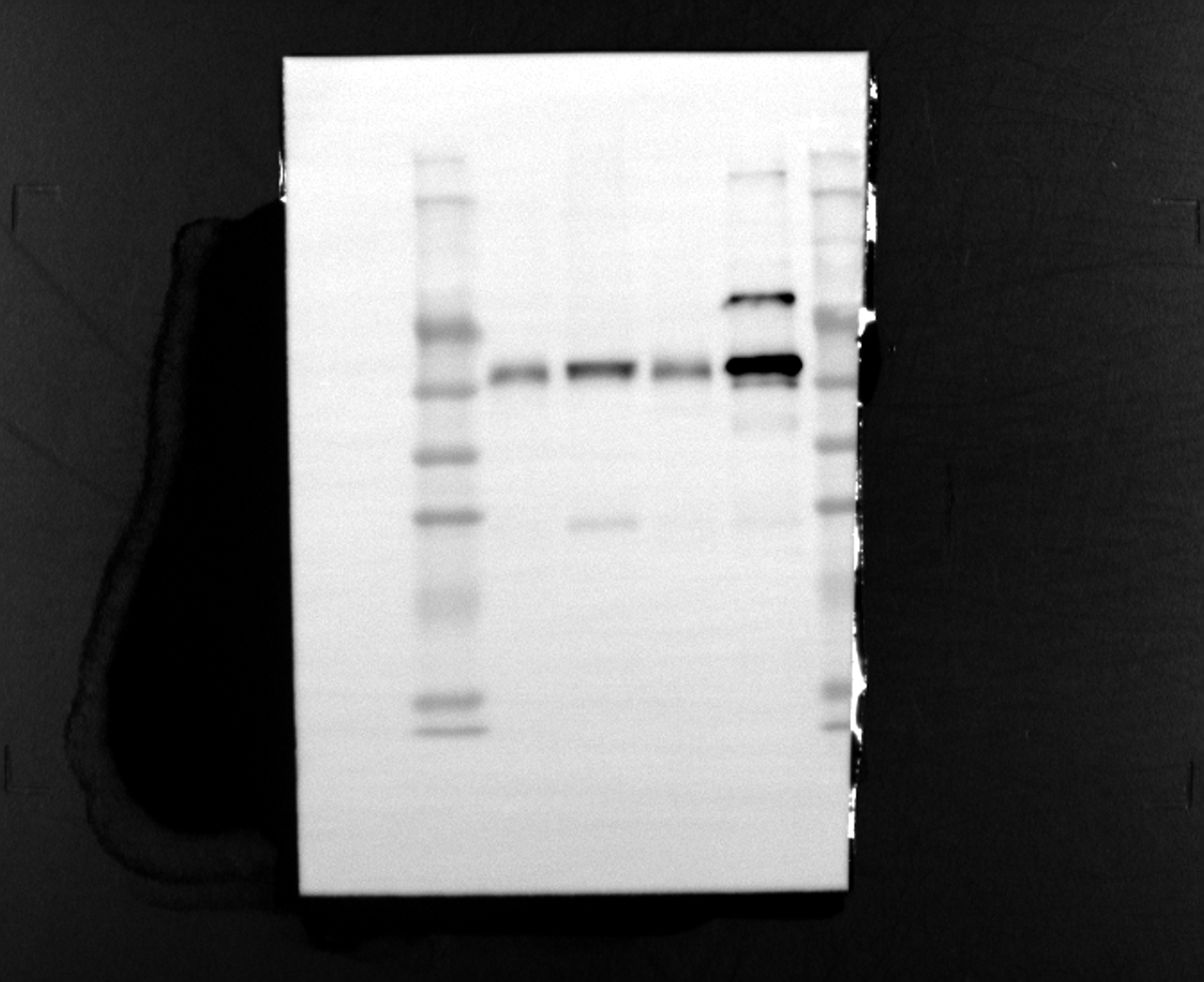

Supplement: Supplementary file 4 — Supplementary Material 4. [file 12964_2024_1770_MOESM4_ESM.zip › SENP3 TAM WB/WB-Figure4/B M0 M2 EndoIP/2023-02-16 ─┌╘┤IP shNC shSENP3 IRF4/IP IRF4/IP IRF4 2.2S M 0216.Tif]

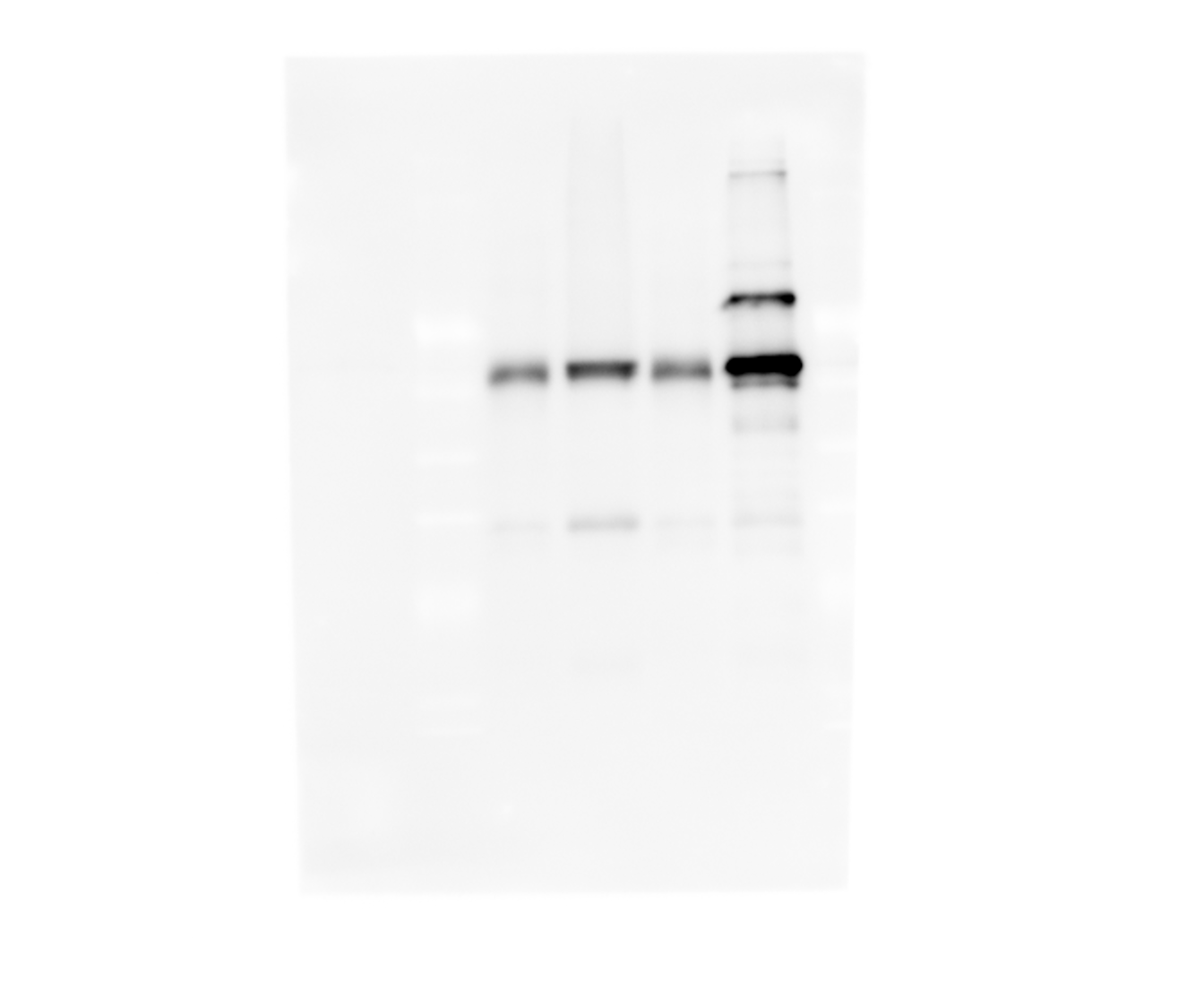

Supplement: Supplementary file 4 — Supplementary Material 4. [file 12964_2024_1770_MOESM4_ESM.zip › SENP3 TAM WB/WB-Figure4/B M0 M2 EndoIP/2023-02-16 ─┌╘┤IP shNC shSENP3 IRF4/IP IRF4/IP IRF4 5.8S 0216.Tif]

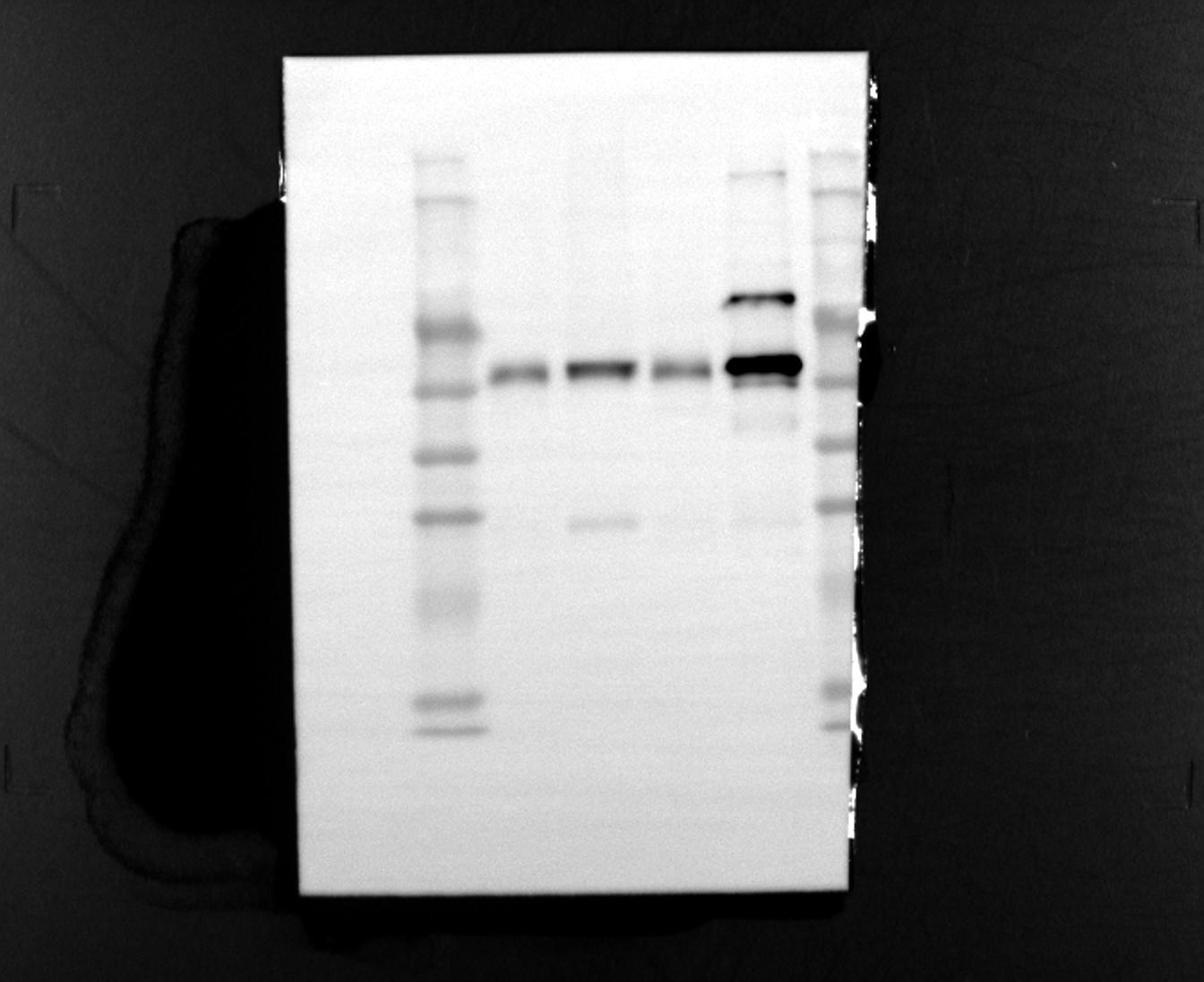

Supplement: Supplementary file 4 — Supplementary Material 4. [file 12964_2024_1770_MOESM4_ESM.zip › SENP3 TAM WB/WB-Figure4/B M0 M2 EndoIP/2023-02-16 ─┌╘┤IP shNC shSENP3 IRF4/IP IRF4/IP IRF4 5.8S M 0216.Tif]

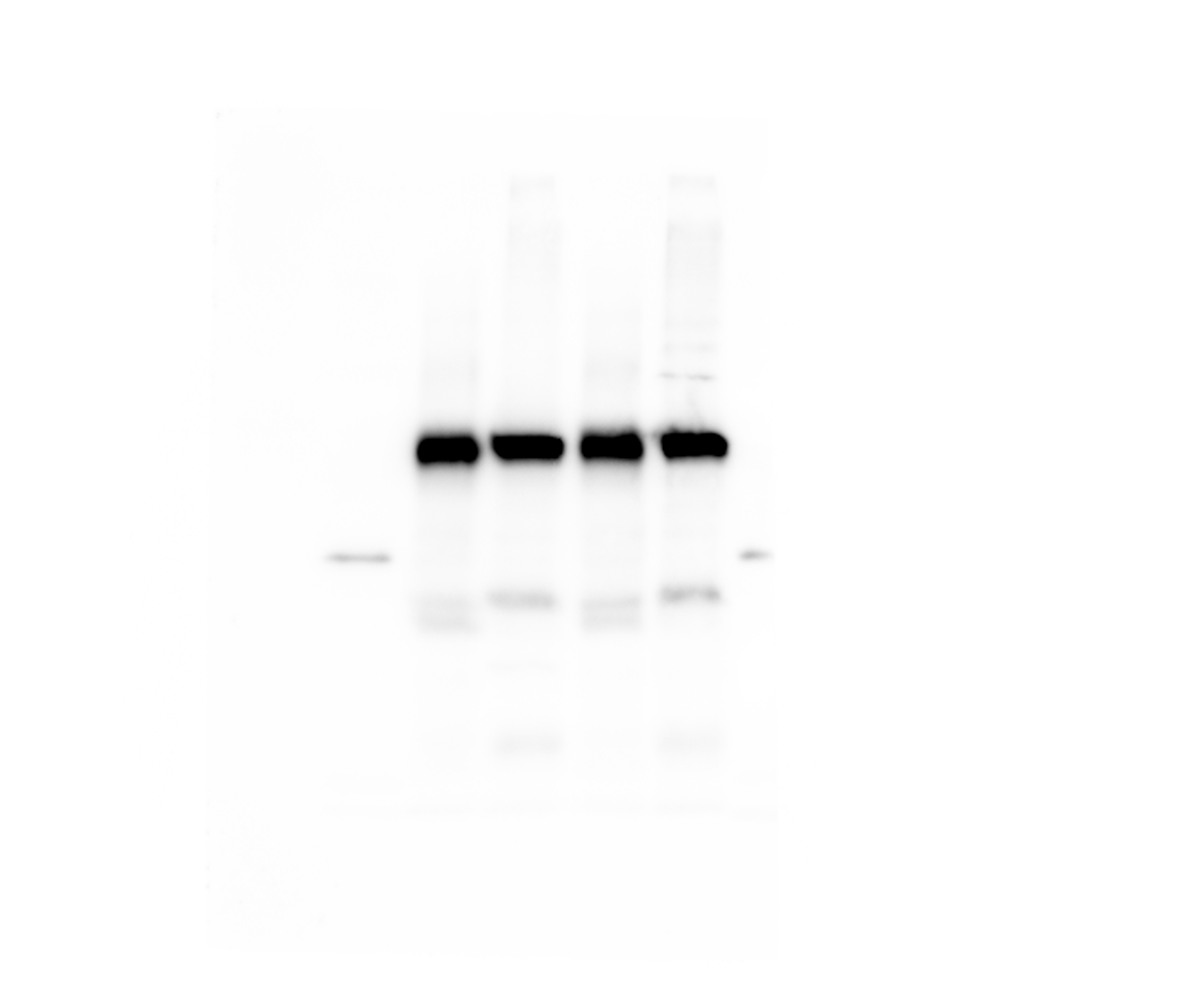

Supplement: Supplementary file 4 — Supplementary Material 4. [file 12964_2024_1770_MOESM4_ESM.zip › SENP3 TAM WB/WB-Figure4/B M0 M2 EndoIP/2023-02-16 ─┌╘┤IP shNC shSENP3 IRF4/IP SUMO23/IP SUMO23 20S 0216.Tif]

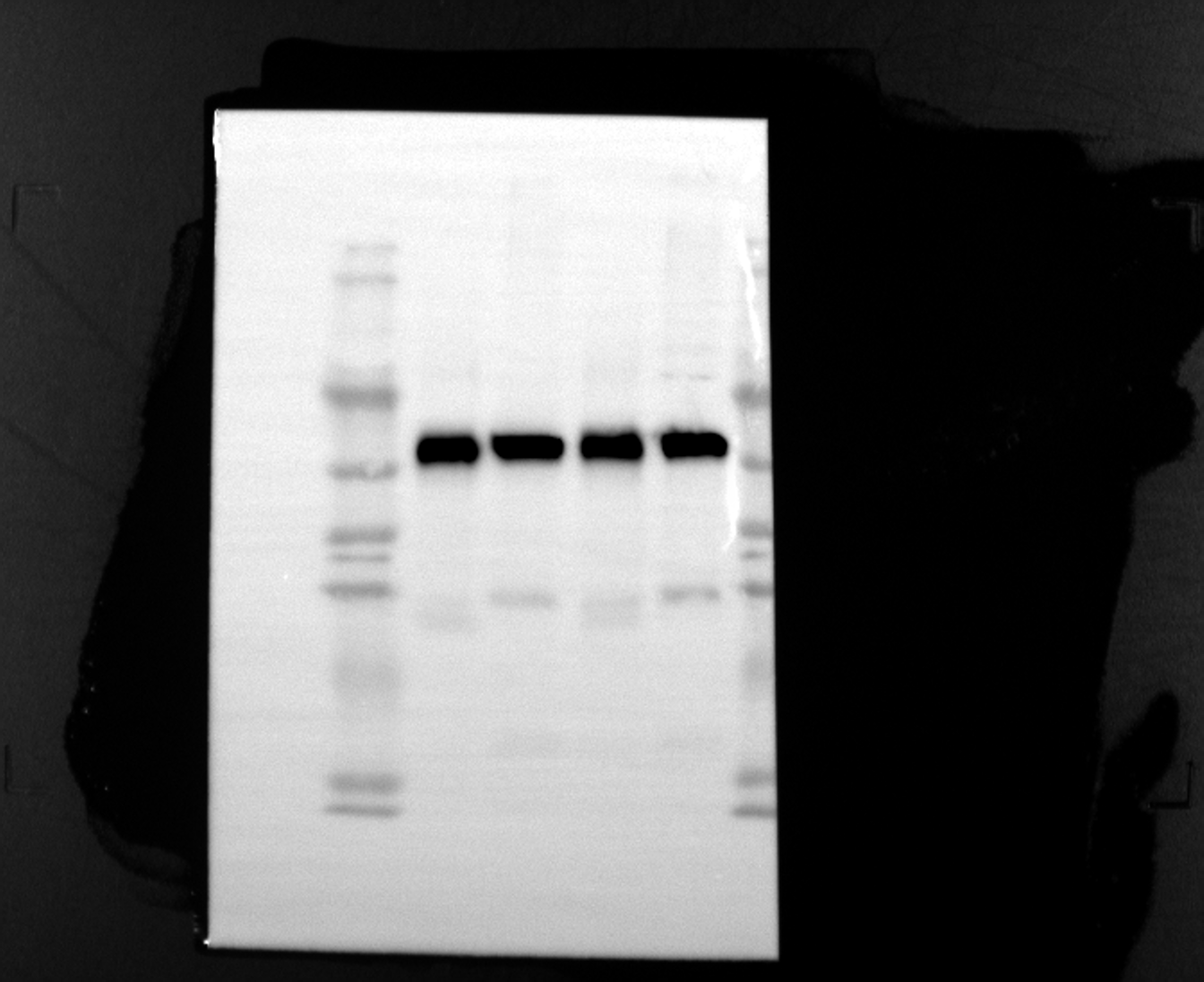

Supplement: Supplementary file 4 — Supplementary Material 4. [file 12964_2024_1770_MOESM4_ESM.zip › SENP3 TAM WB/WB-Figure4/B M0 M2 EndoIP/2023-02-16 ─┌╘┤IP shNC shSENP3 IRF4/IP SUMO23/IP SUMO23 20S M 0216.Tif]

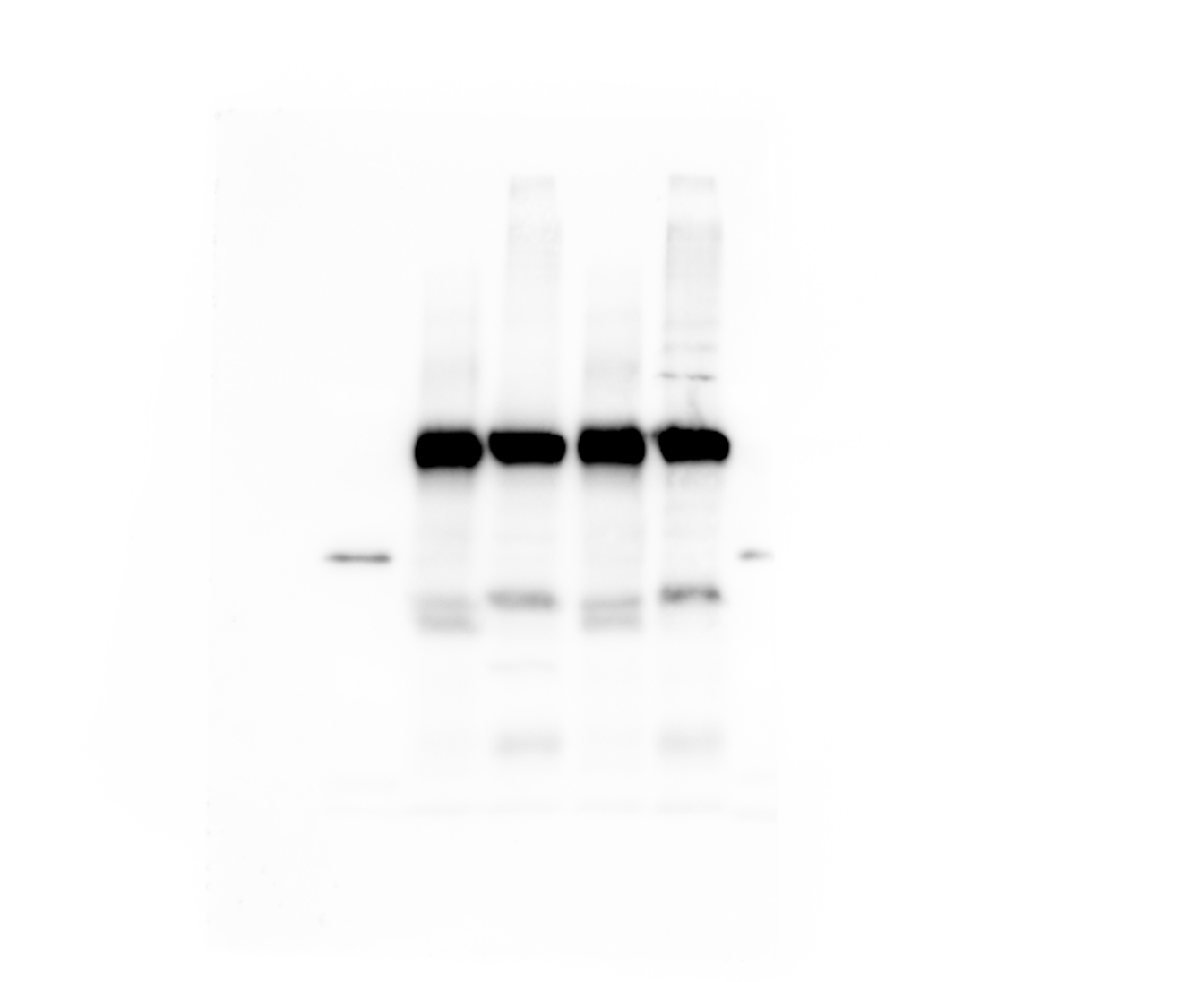

Supplement: Supplementary file 4 — Supplementary Material 4. [file 12964_2024_1770_MOESM4_ESM.zip › SENP3 TAM WB/WB-Figure4/B M0 M2 EndoIP/2023-02-16 ─┌╘┤IP shNC shSENP3 IRF4/IP SUMO23/IP SUMO23 40S 0216.Tif]

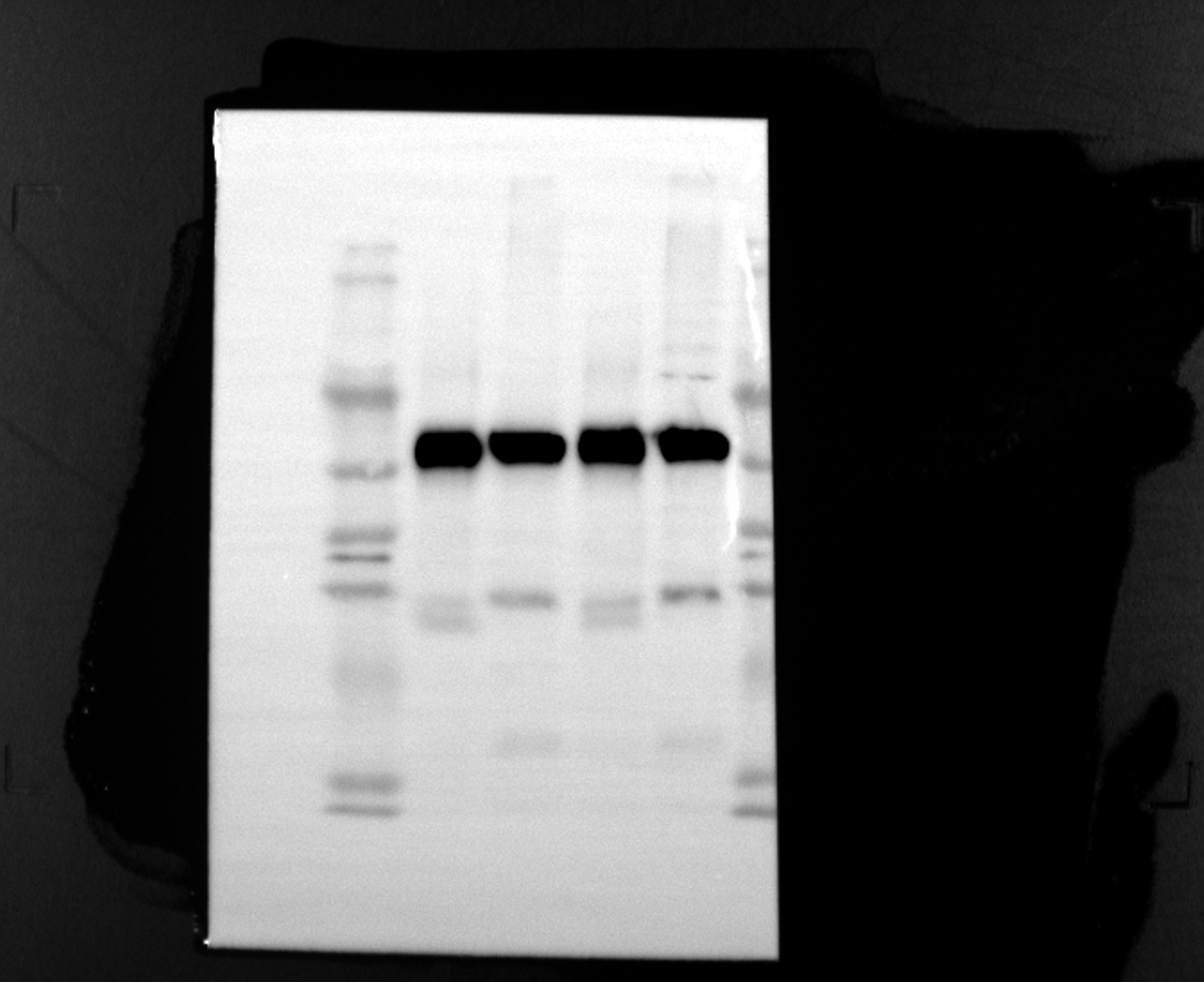

Supplement: Supplementary file 4 — Supplementary Material 4. [file 12964_2024_1770_MOESM4_ESM.zip › SENP3 TAM WB/WB-Figure4/B M0 M2 EndoIP/2023-02-16 ─┌╘┤IP shNC shSENP3 IRF4/IP SUMO23/IP SUMO23 40S M 0216.Tif]

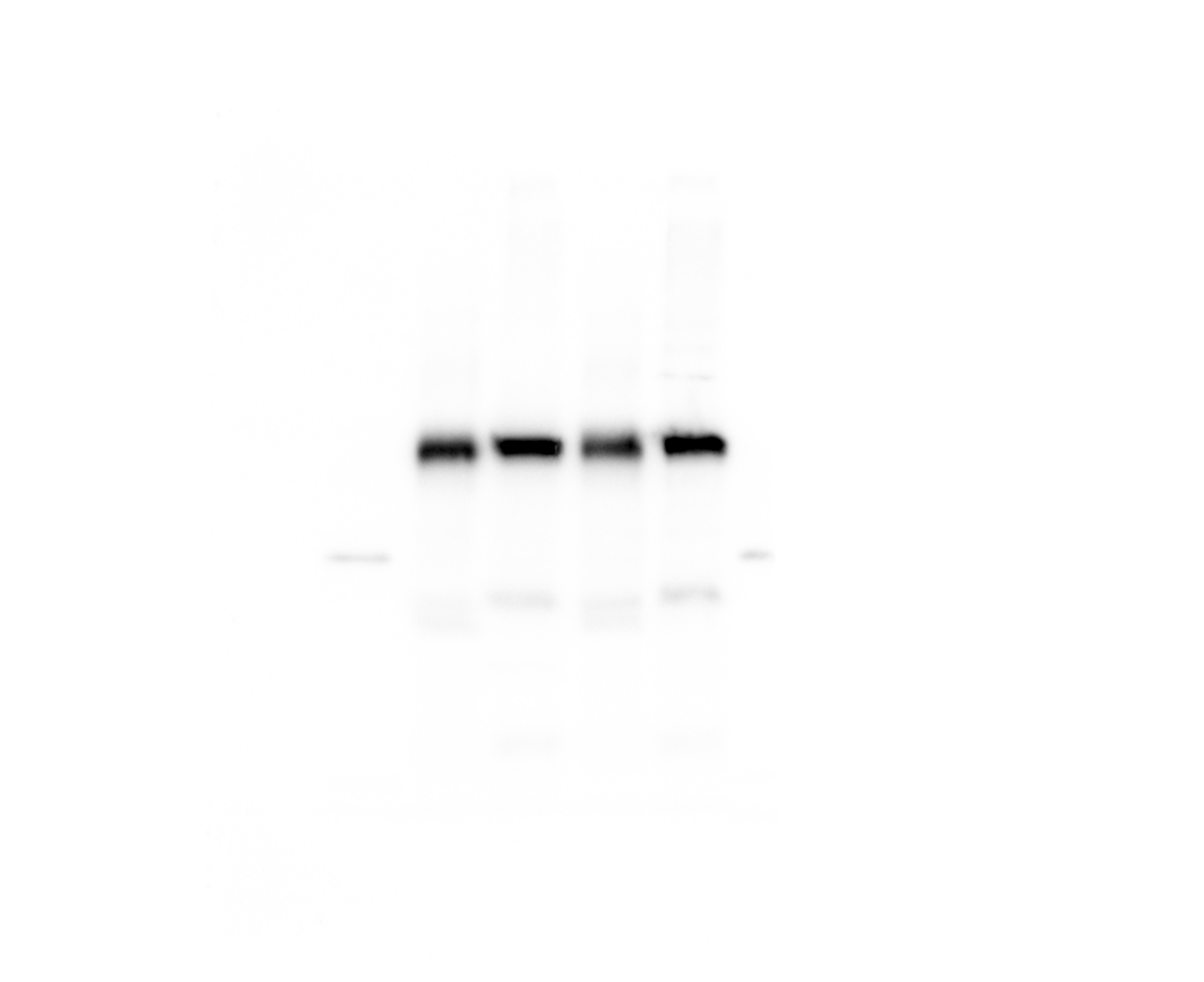

Supplement: Supplementary file 4 — Supplementary Material 4. [file 12964_2024_1770_MOESM4_ESM.zip › SENP3 TAM WB/WB-Figure4/B M0 M2 EndoIP/2023-02-16 ─┌╘┤IP shNC shSENP3 IRF4/IP SUMO23/IP SUMO23 4S 0216.Tif]

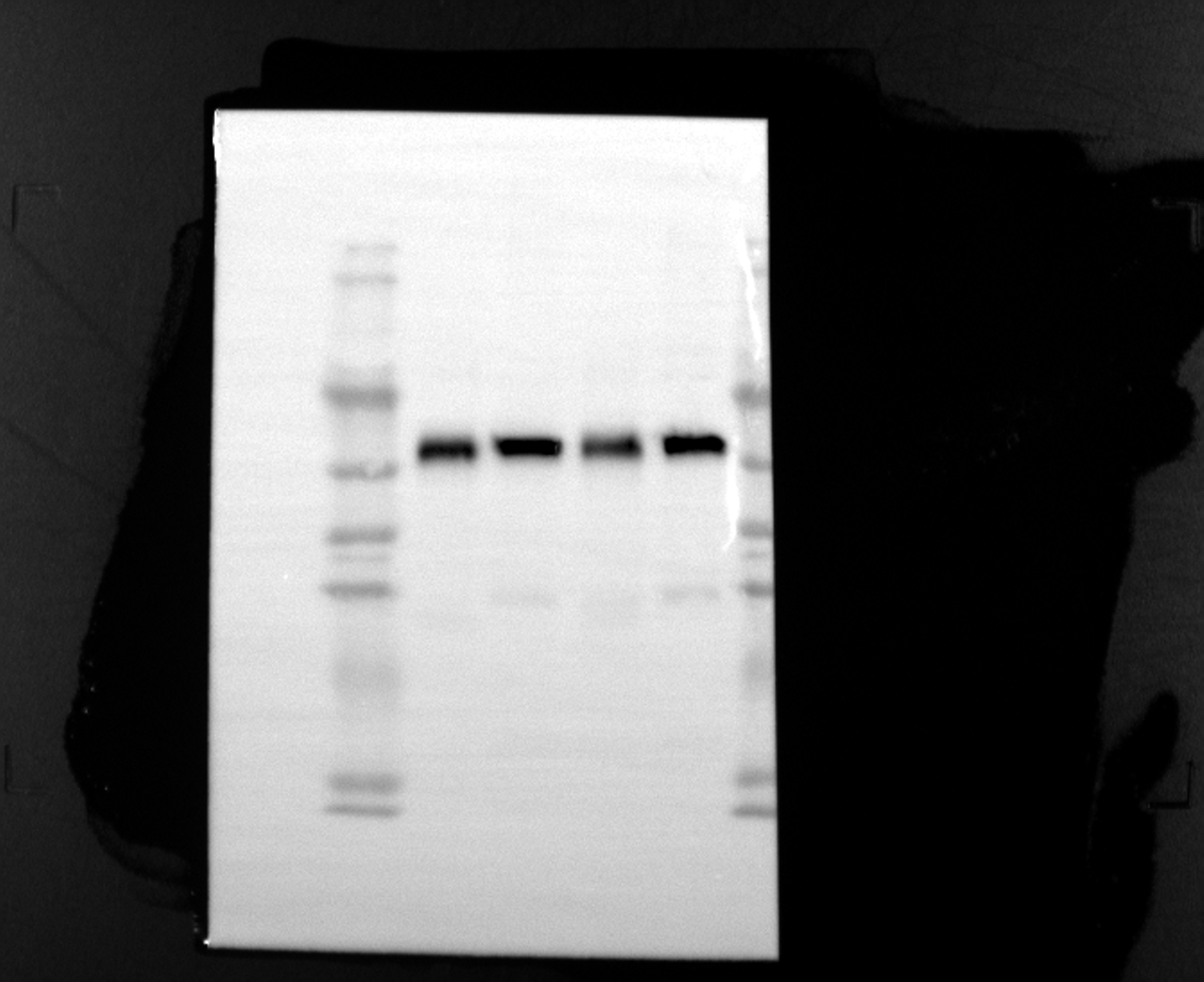

Supplement: Supplementary file 4 — Supplementary Material 4. [file 12964_2024_1770_MOESM4_ESM.zip › SENP3 TAM WB/WB-Figure4/B M0 M2 EndoIP/2023-02-16 ─┌╘┤IP shNC shSENP3 IRF4/IP SUMO23/IP SUMO23 4S M 0216.Tif]

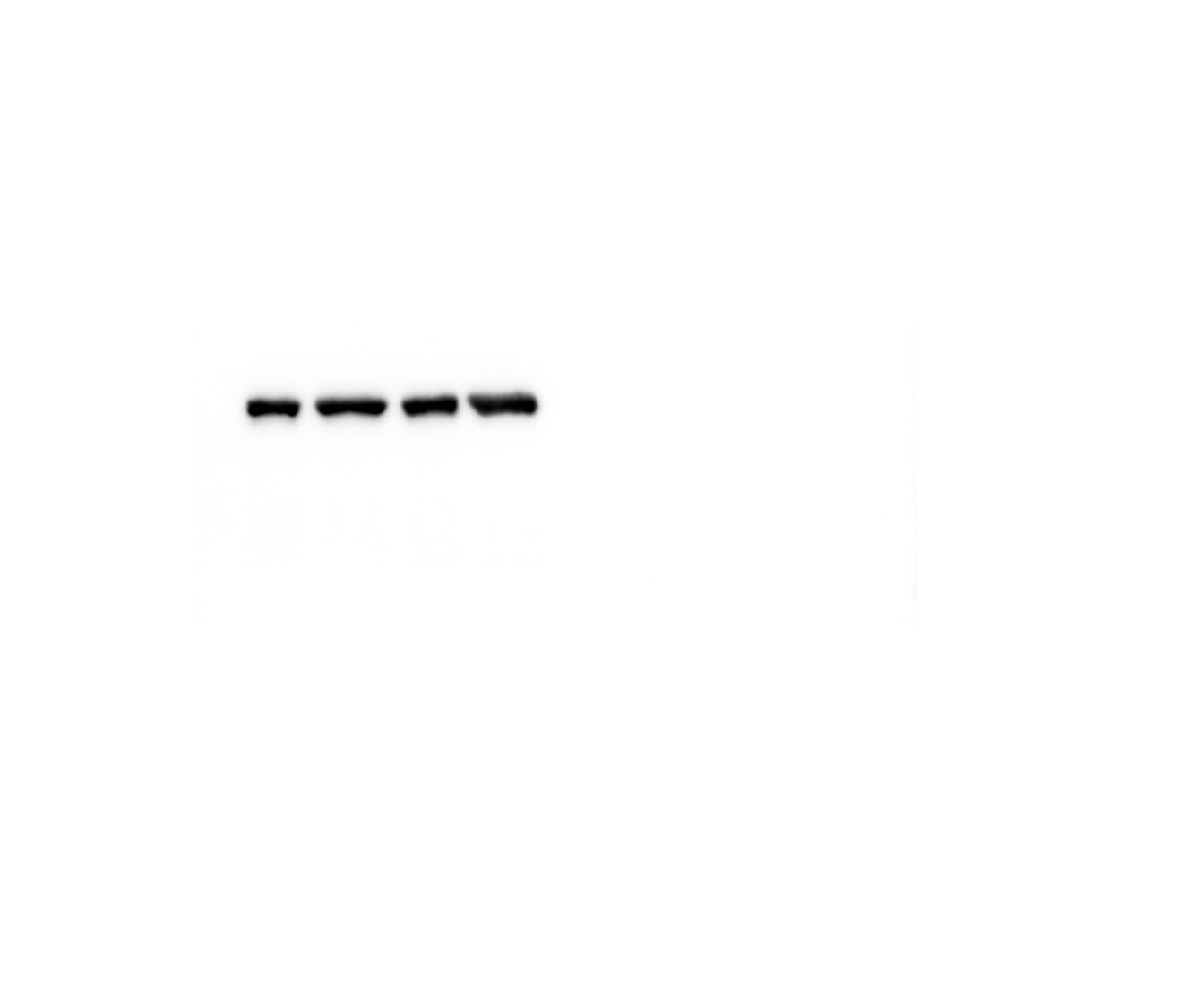

Supplement: Supplementary file 4 — Supplementary Material 4. [file 12964_2024_1770_MOESM4_ESM.zip › SENP3 TAM WB/WB-Figure4/B M0 M2 EndoIP/2023-02-18 ─┌╘┤IP shNC shSENP3 IRF4/INPUT ACT 0222/INPUT ACT 0.8S 0222.Tif]

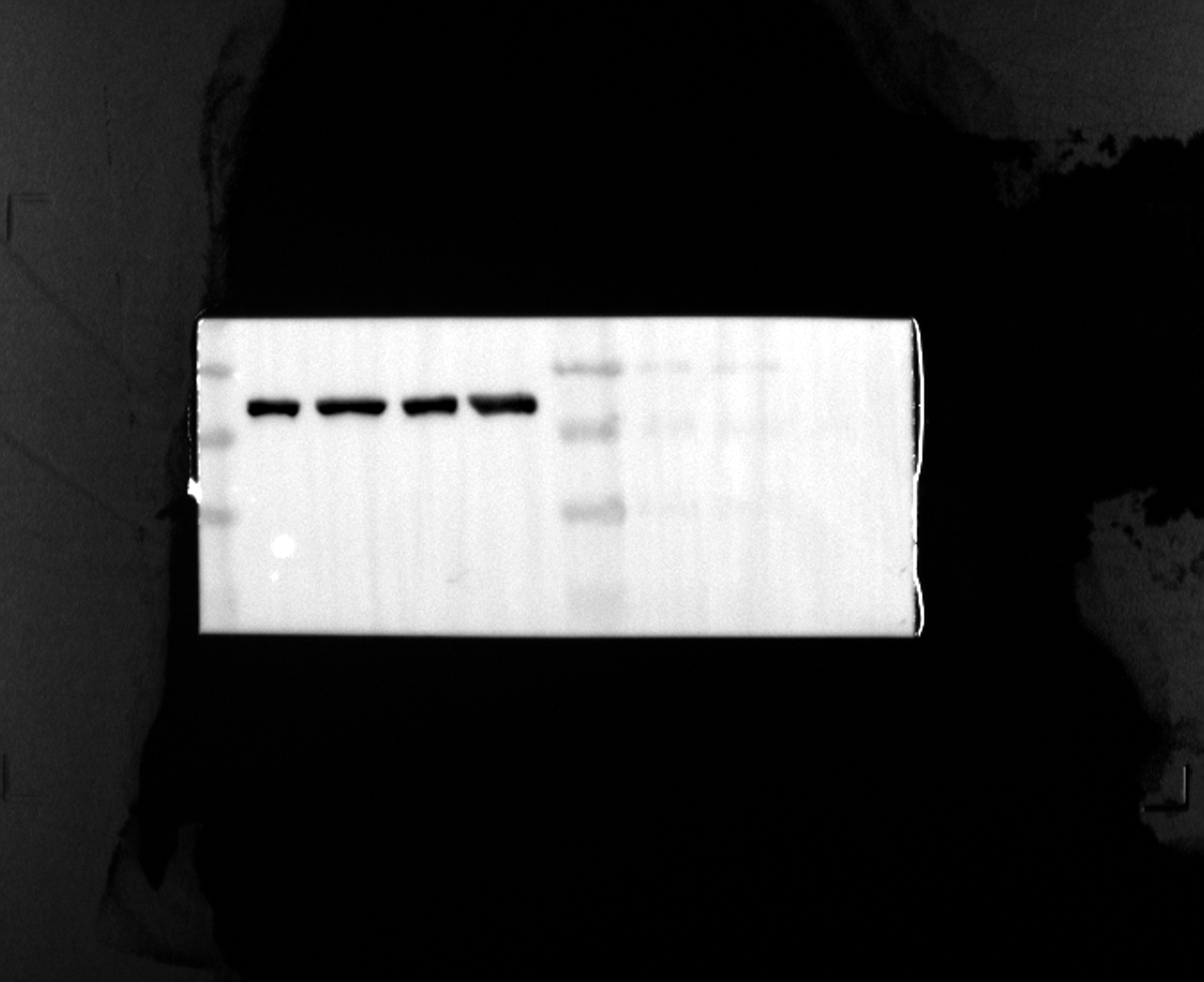

Supplement: Supplementary file 4 — Supplementary Material 4. [file 12964_2024_1770_MOESM4_ESM.zip › SENP3 TAM WB/WB-Figure4/B M0 M2 EndoIP/2023-02-18 ─┌╘┤IP shNC shSENP3 IRF4/INPUT ACT 0222/INPUT ACT 0.8S M 0222.Tif]

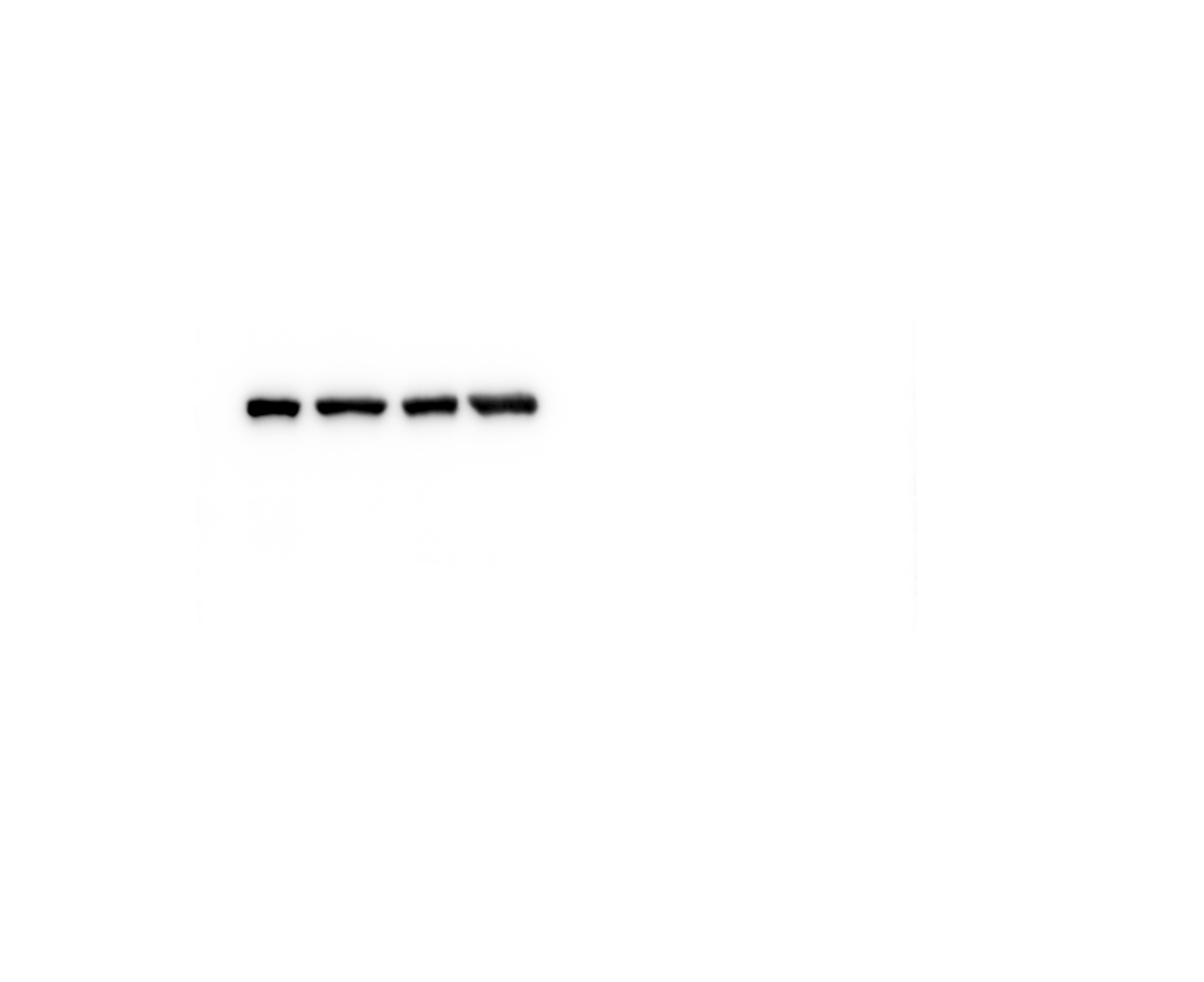

Supplement: Supplementary file 4 — Supplementary Material 4. [file 12964_2024_1770_MOESM4_ESM.zip › SENP3 TAM WB/WB-Figure4/B M0 M2 EndoIP/2023-02-18 ─┌╘┤IP shNC shSENP3 IRF4/INPUT ACT 0222/INPUT ACT 1.1S 0222.Tif]

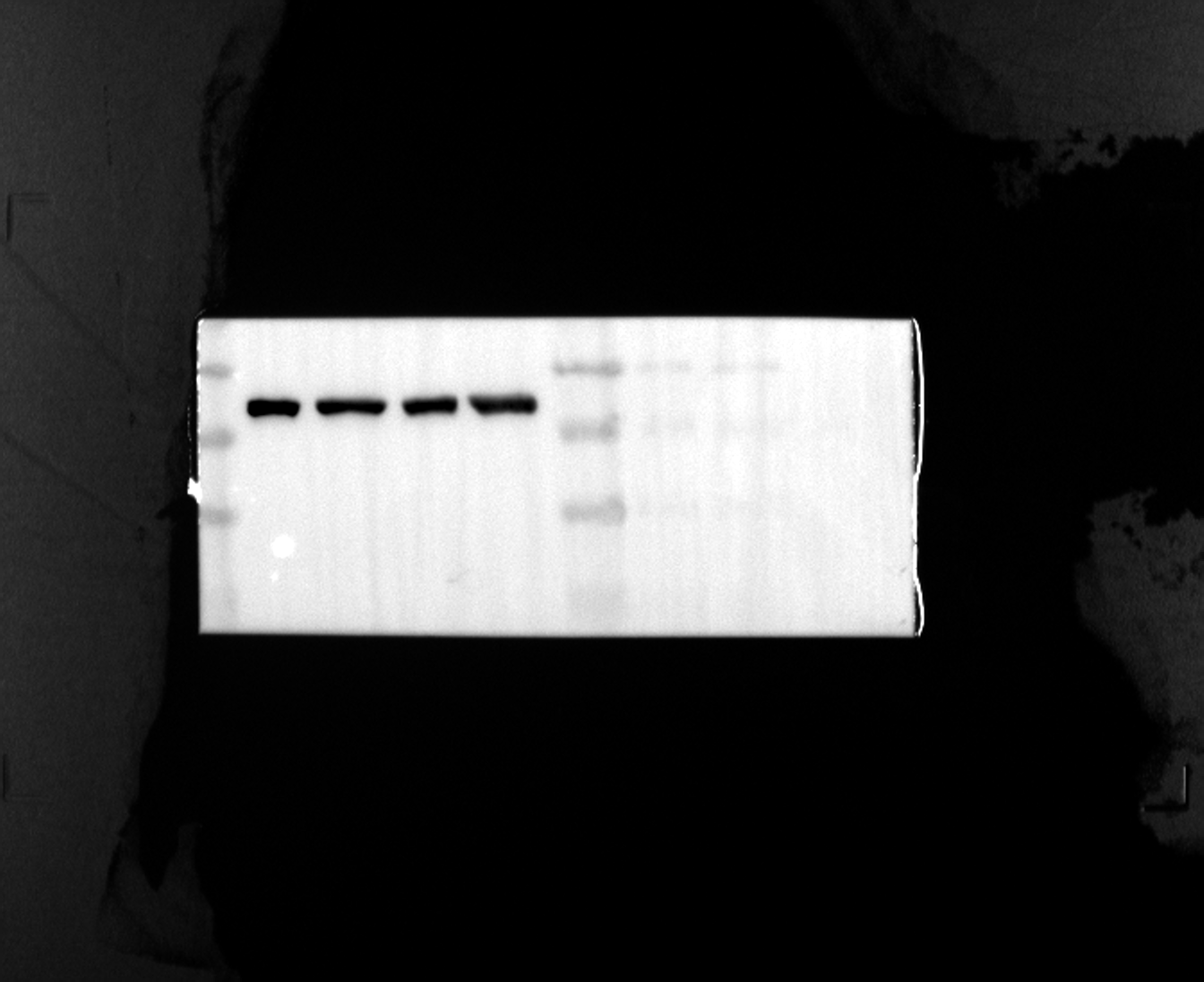

Supplement: Supplementary file 4 — Supplementary Material 4. [file 12964_2024_1770_MOESM4_ESM.zip › SENP3 TAM WB/WB-Figure4/B M0 M2 EndoIP/2023-02-18 ─┌╘┤IP shNC shSENP3 IRF4/INPUT ACT 0222/INPUT ACT 1.1S M 0222.Tif]

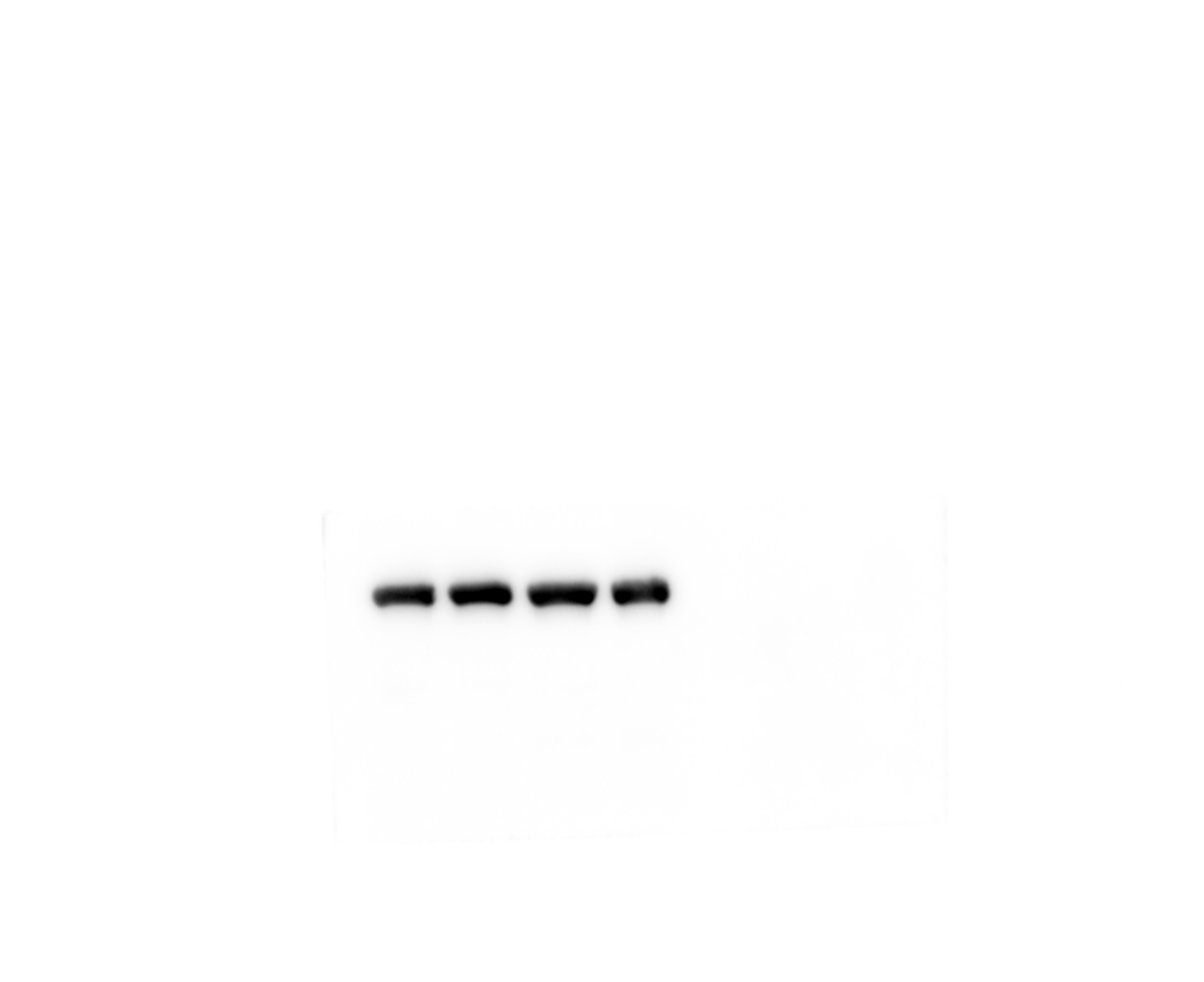

Supplement: Supplementary file 4 — Supplementary Material 4. [file 12964_2024_1770_MOESM4_ESM.zip › SENP3 TAM WB/WB-Figure4/B M0 M2 EndoIP/2023-02-18 ─┌╘┤IP shNC shSENP3 IRF4/INPUT ACT 0225/INPUT ACT 0225 2S.Tif]

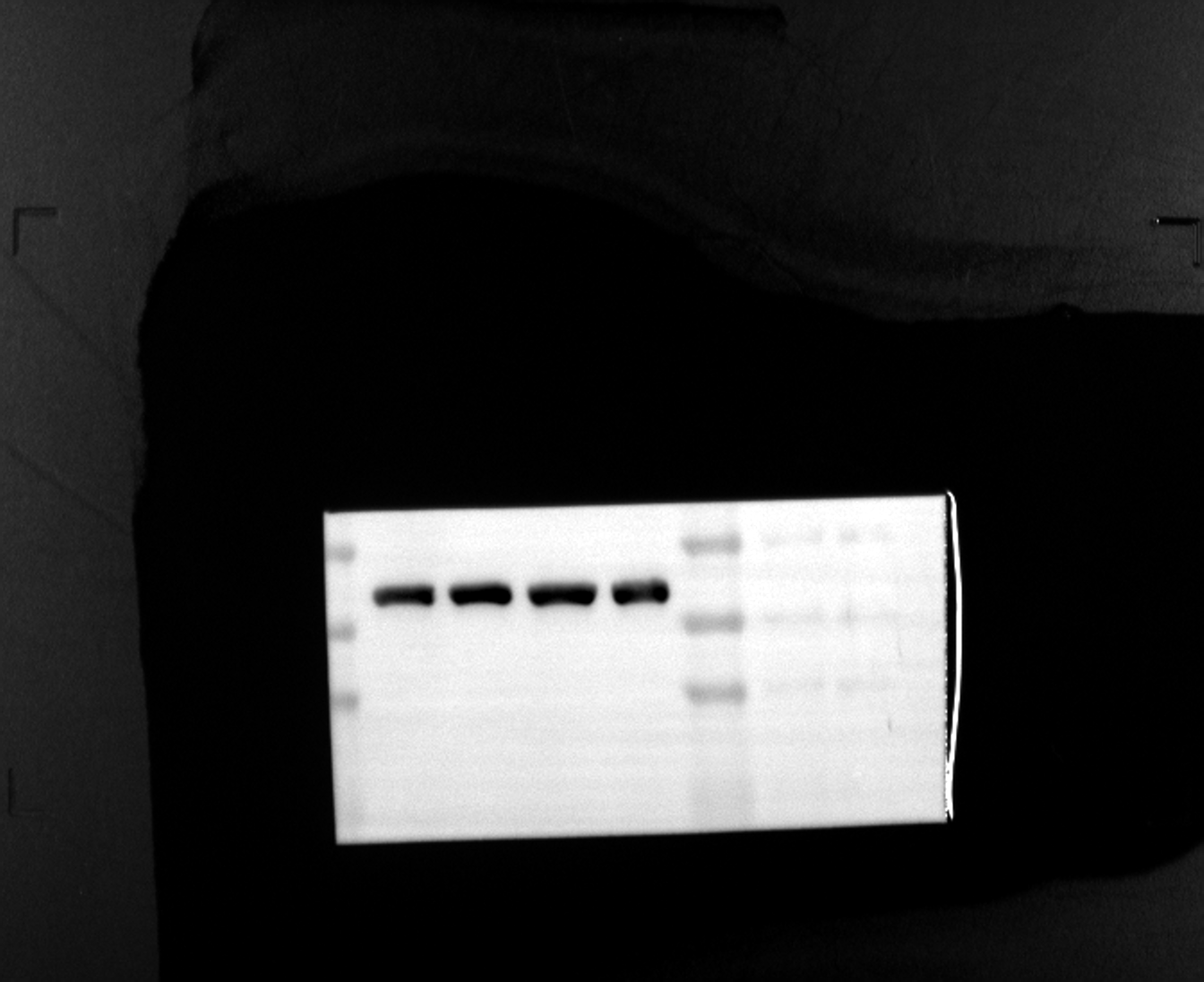

Supplement: Supplementary file 4 — Supplementary Material 4. [file 12964_2024_1770_MOESM4_ESM.zip › SENP3 TAM WB/WB-Figure4/B M0 M2 EndoIP/2023-02-18 ─┌╘┤IP shNC shSENP3 IRF4/INPUT ACT 0225/INPUT ACT 0225 M 2S.Tif]

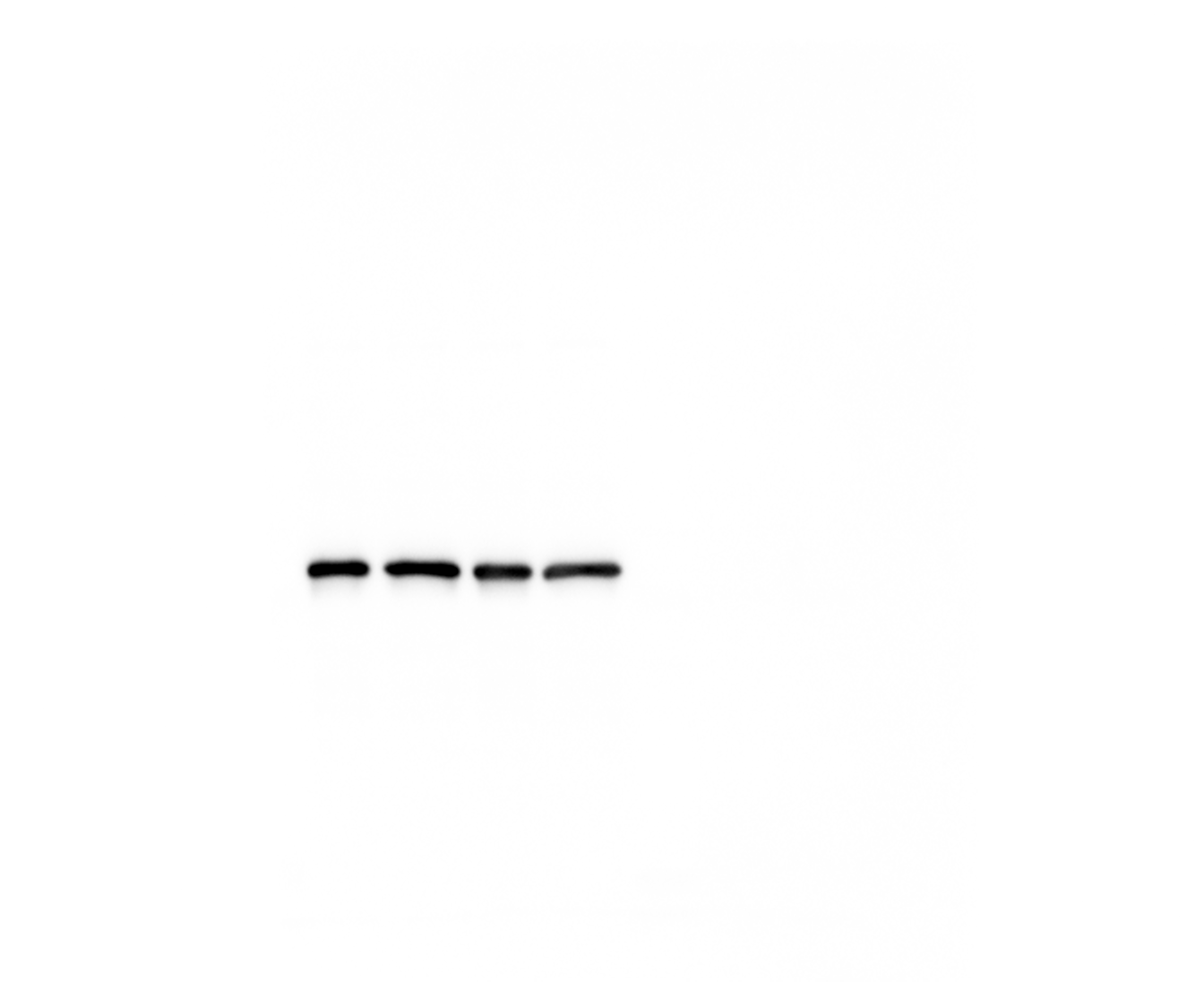

Supplement: Supplementary file 4 — Supplementary Material 4. [file 12964_2024_1770_MOESM4_ESM.zip › SENP3 TAM WB/WB-Figure4/B M0 M2 EndoIP/2023-02-18 ─┌╘┤IP shNC shSENP3 IRF4/INPUT ACT/INPUT ACT 0.3S 0221.Tif]

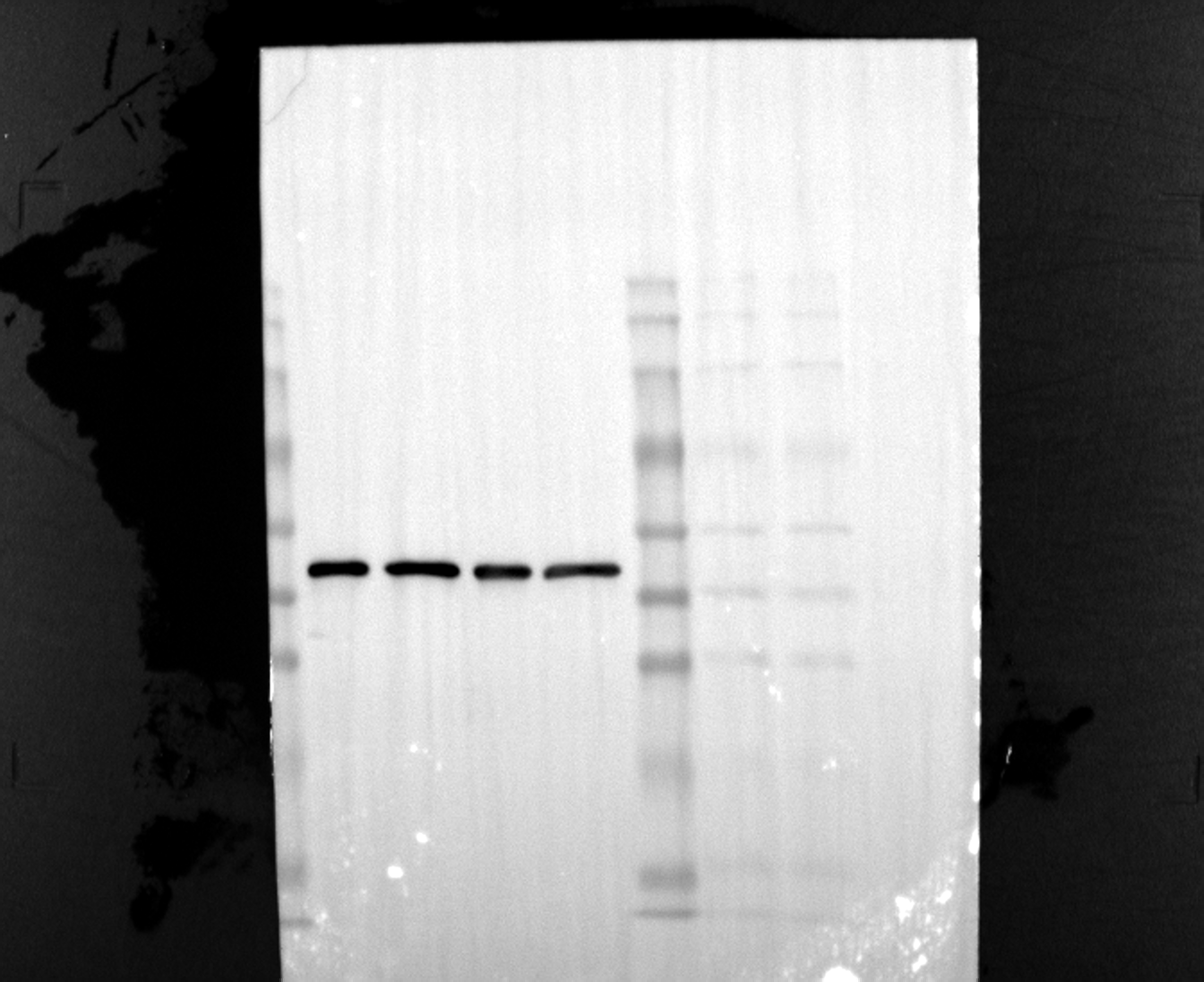

Supplement: Supplementary file 4 — Supplementary Material 4. [file 12964_2024_1770_MOESM4_ESM.zip › SENP3 TAM WB/WB-Figure4/B M0 M2 EndoIP/2023-02-18 ─┌╘┤IP shNC shSENP3 IRF4/INPUT ACT/INPUT ACT 0.3S M 0221.Tif]

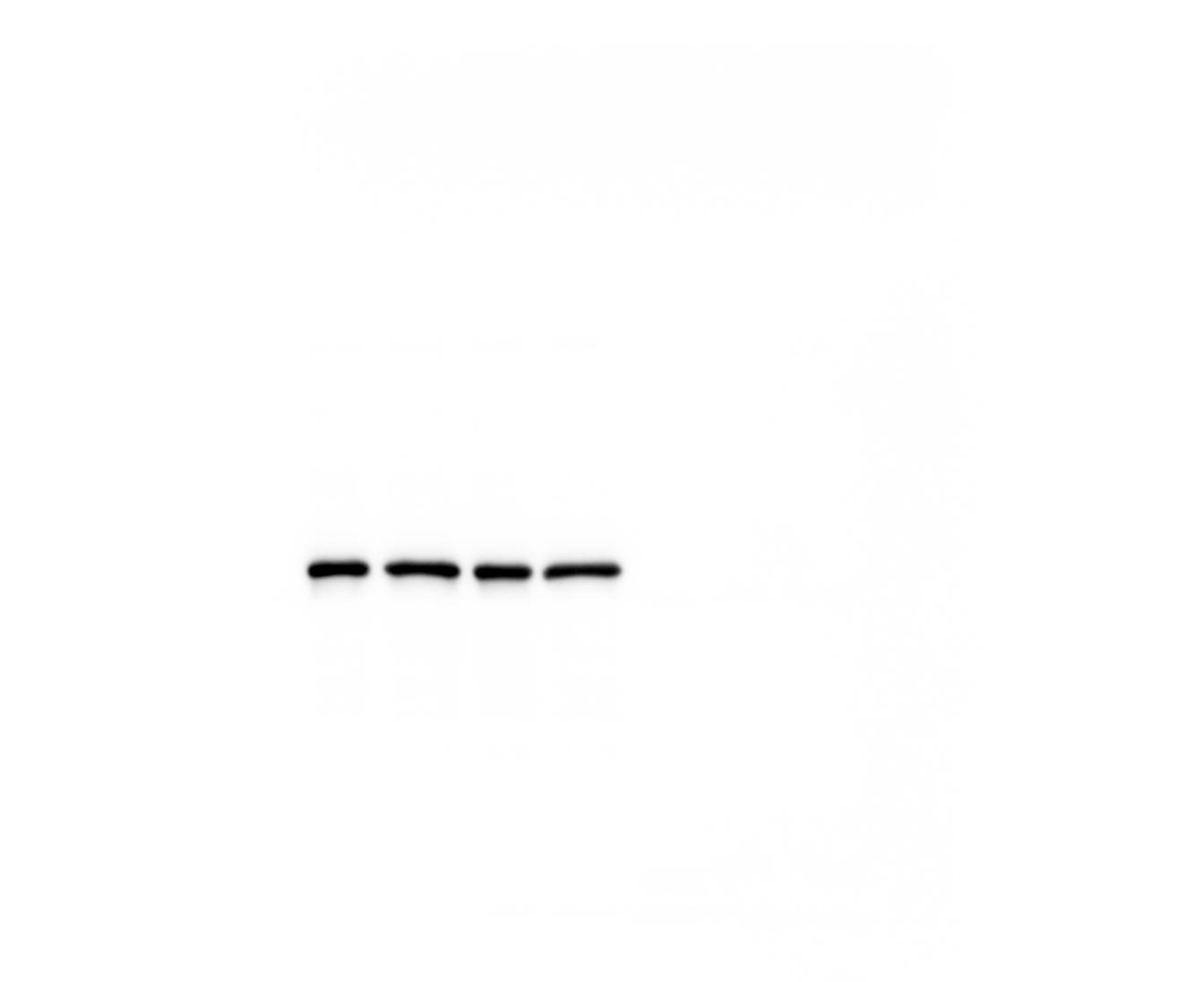

Supplement: Supplementary file 4 — Supplementary Material 4. [file 12964_2024_1770_MOESM4_ESM.zip › SENP3 TAM WB/WB-Figure4/B M0 M2 EndoIP/2023-02-18 ─┌╘┤IP shNC shSENP3 IRF4/INPUT ACT/INPUT ACT 0.6S 0221.Tif]

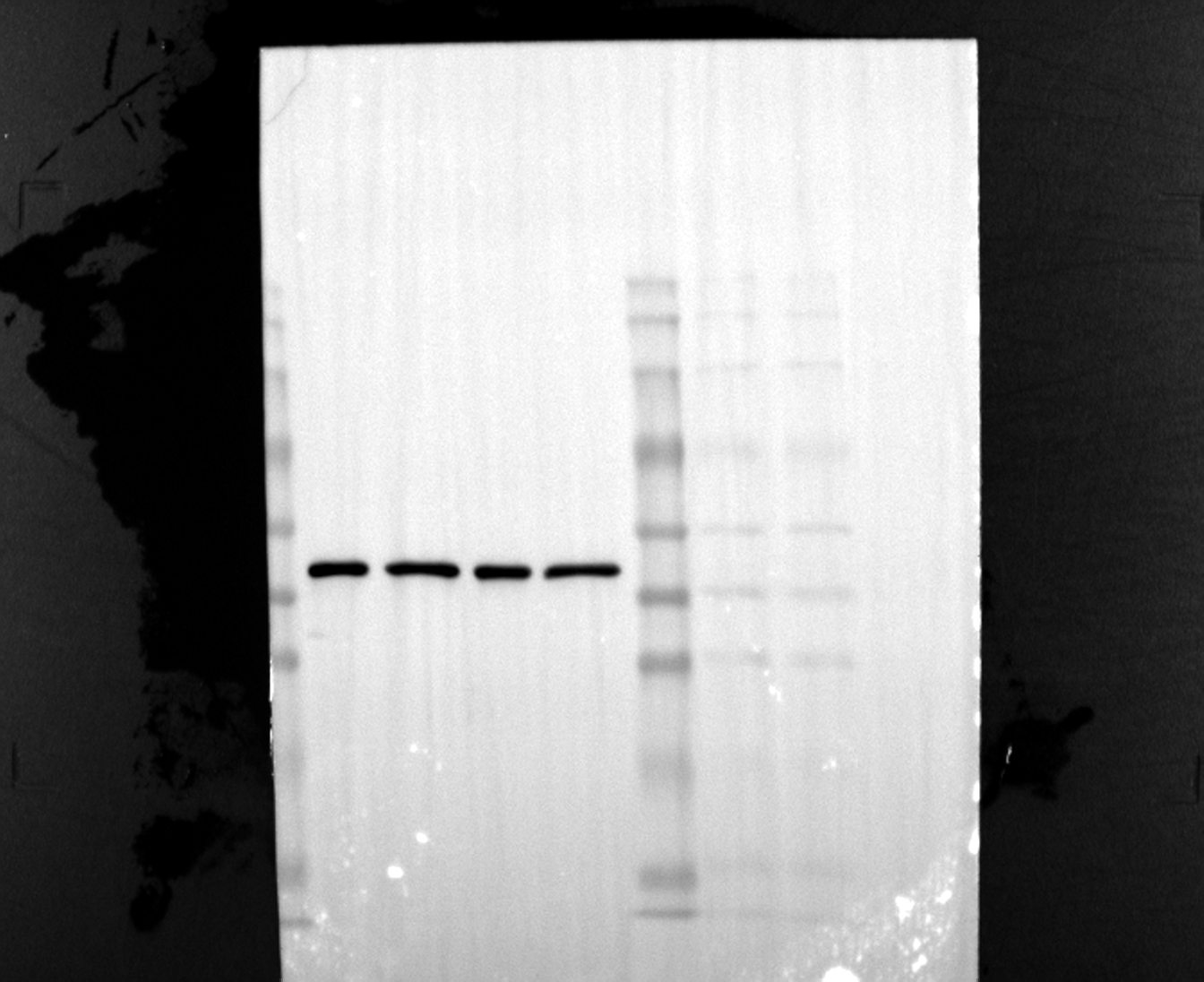

Supplement: Supplementary file 4 — Supplementary Material 4. [file 12964_2024_1770_MOESM4_ESM.zip › SENP3 TAM WB/WB-Figure4/B M0 M2 EndoIP/2023-02-18 ─┌╘┤IP shNC shSENP3 IRF4/INPUT ACT/INPUT ACT 0.6S M 0221.Tif]

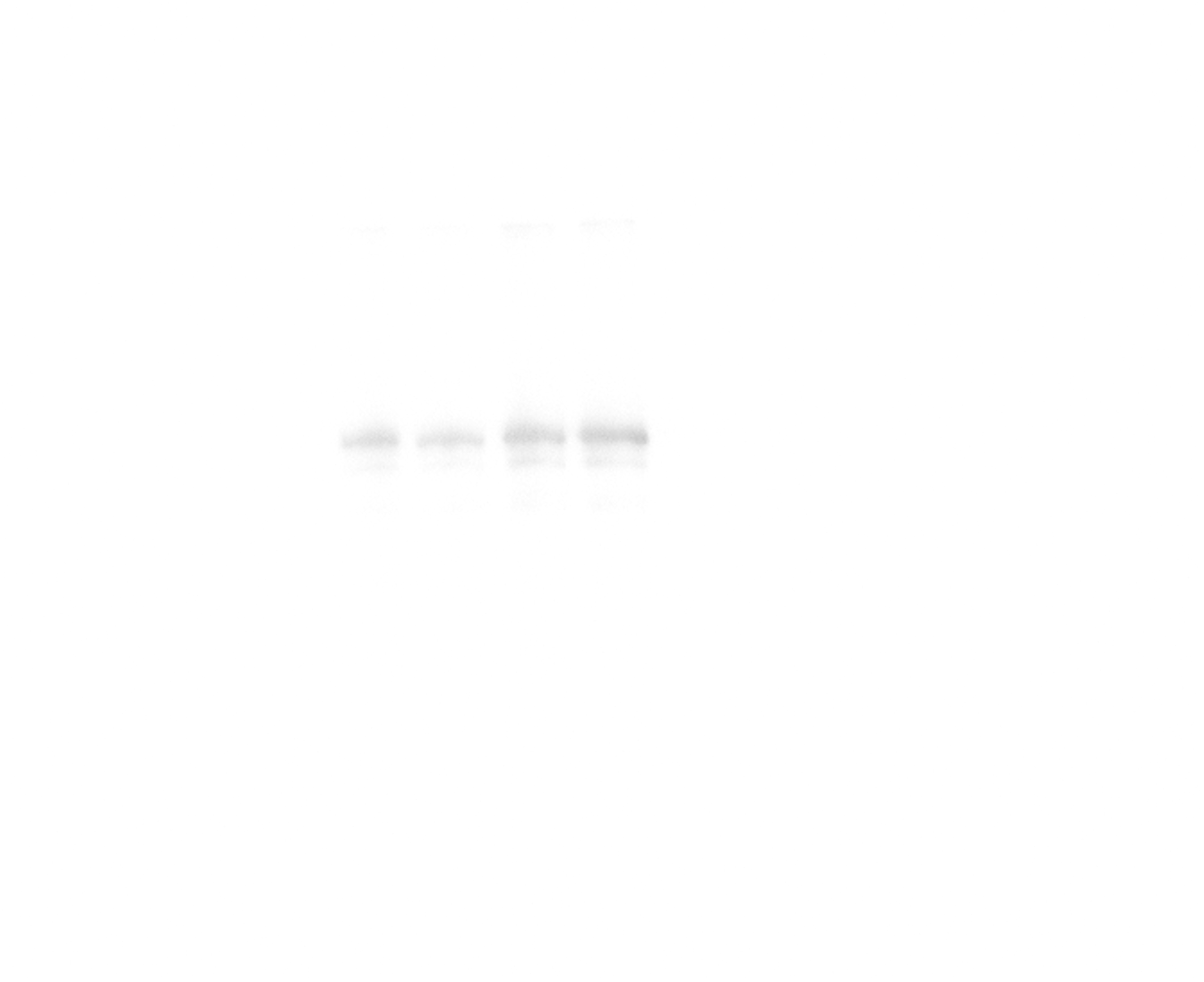

Supplement: Supplementary file 4 — Supplementary Material 4. [file 12964_2024_1770_MOESM4_ESM.zip › SENP3 TAM WB/WB-Figure4/B M0 M2 EndoIP/2023-02-18 ─┌╘┤IP shNC shSENP3 IRF4/INPUT IRF4/INPUT IRF4 0.2S 0218.Tif]

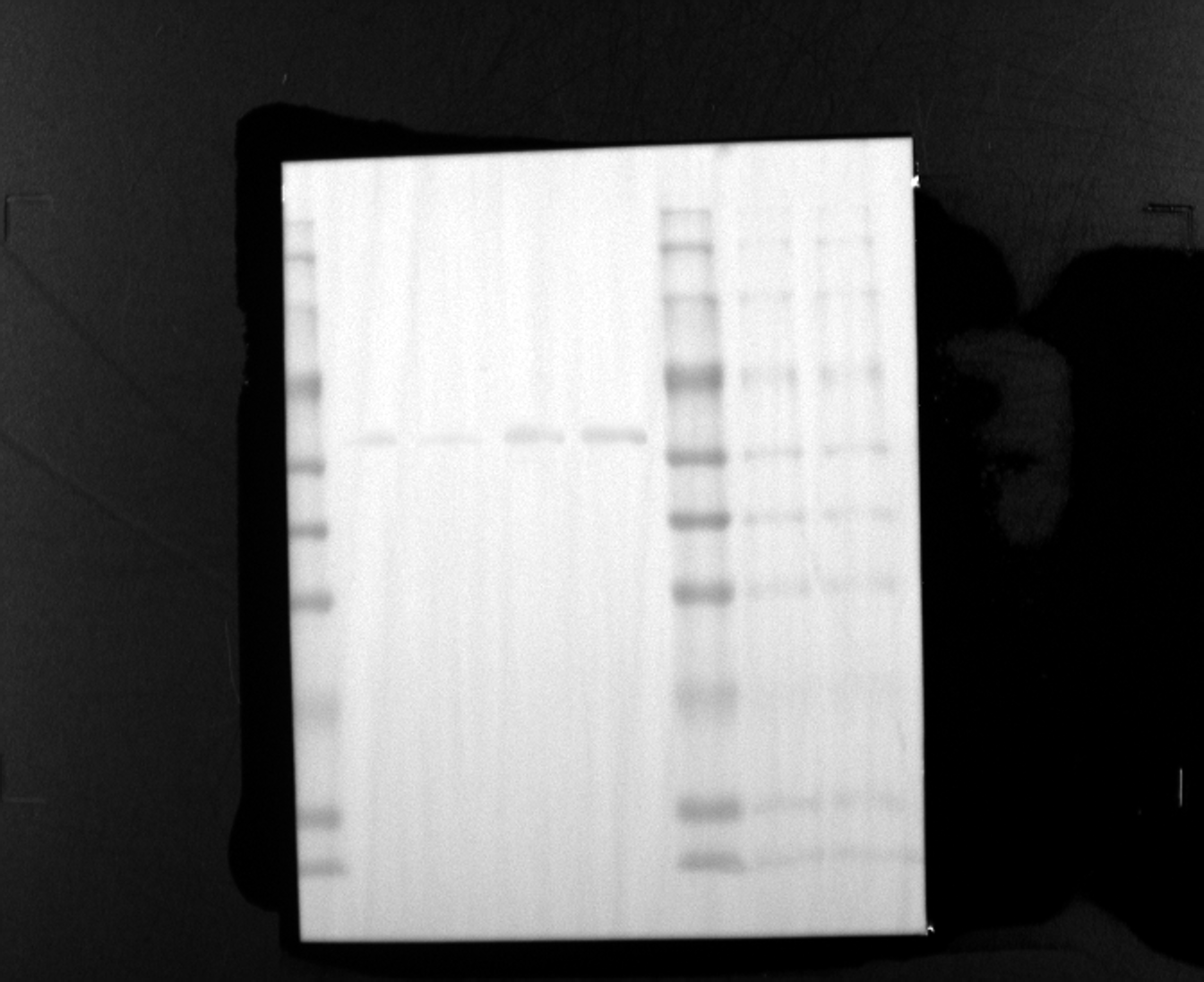

Supplement: Supplementary file 4 — Supplementary Material 4. [file 12964_2024_1770_MOESM4_ESM.zip › SENP3 TAM WB/WB-Figure4/B M0 M2 EndoIP/2023-02-18 ─┌╘┤IP shNC shSENP3 IRF4/INPUT IRF4/INPUT IRF4 0.2S M 0218.Tif]

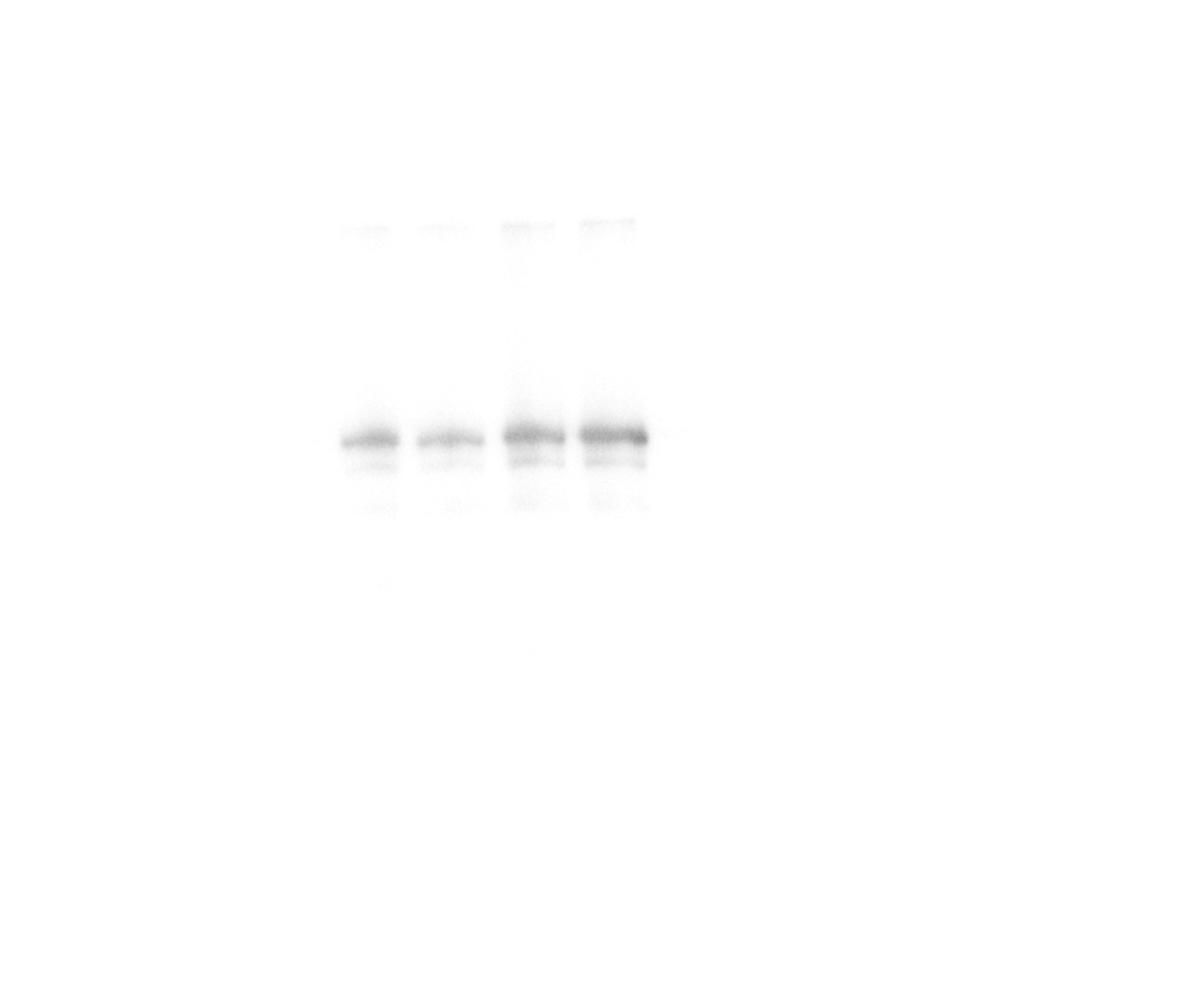

Supplement: Supplementary file 4 — Supplementary Material 4. [file 12964_2024_1770_MOESM4_ESM.zip › SENP3 TAM WB/WB-Figure4/B M0 M2 EndoIP/2023-02-18 ─┌╘┤IP shNC shSENP3 IRF4/INPUT IRF4/INPUT IRF4 0.4S 0218.Tif]

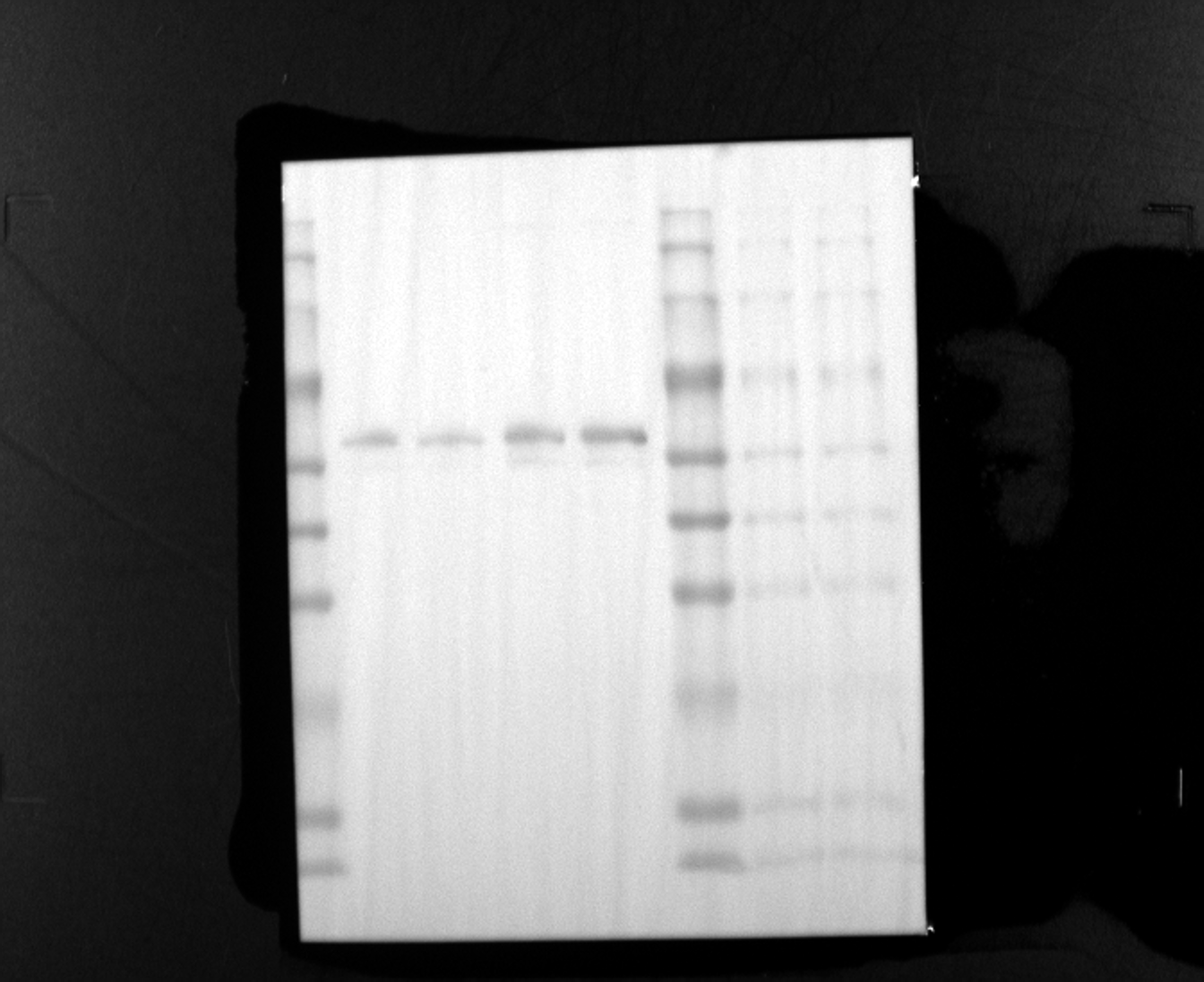

Supplement: Supplementary file 4 — Supplementary Material 4. [file 12964_2024_1770_MOESM4_ESM.zip › SENP3 TAM WB/WB-Figure4/B M0 M2 EndoIP/2023-02-18 ─┌╘┤IP shNC shSENP3 IRF4/INPUT IRF4/INPUT IRF4 0.4S M 0218.Tif]

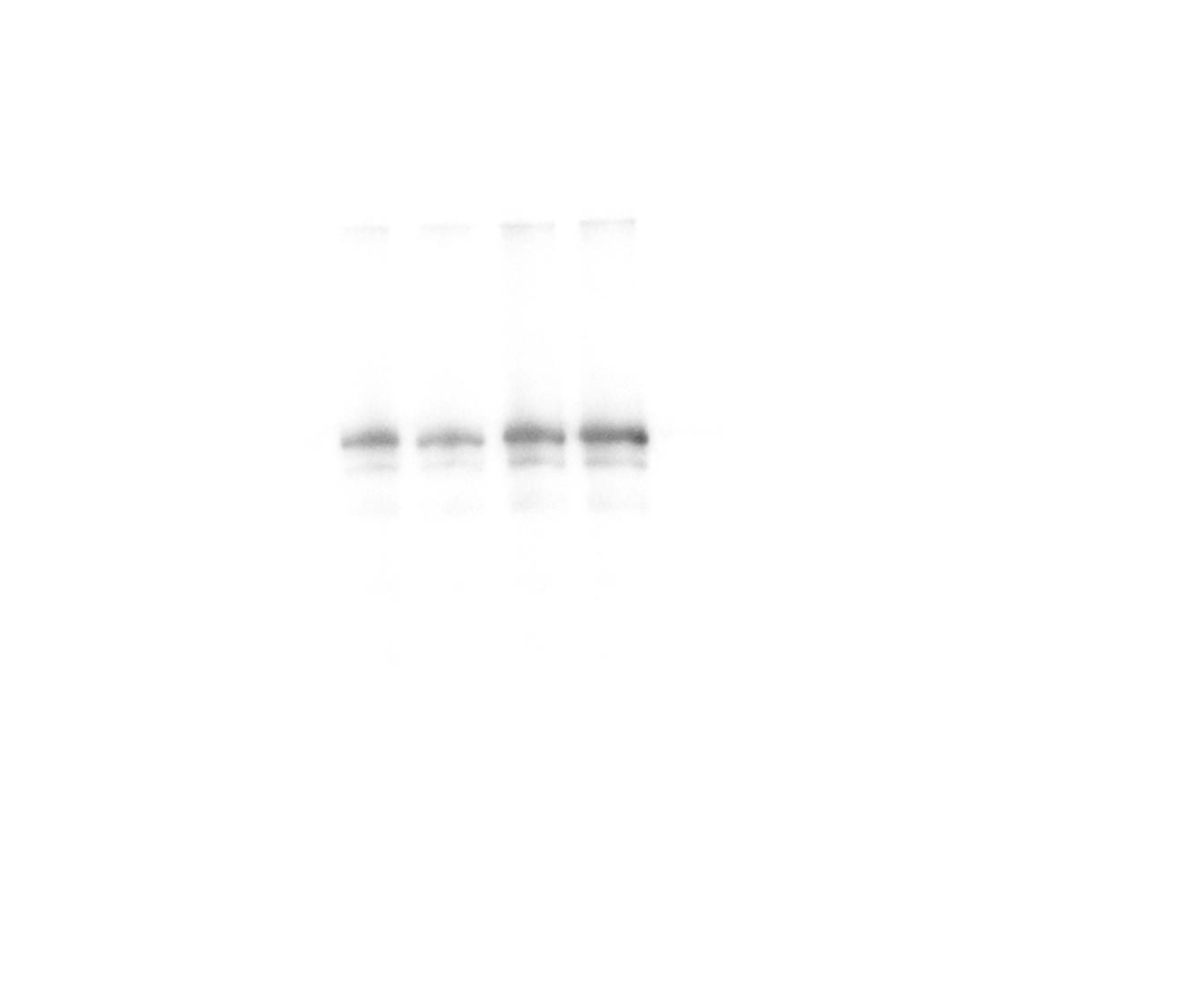

Supplement: Supplementary file 4 — Supplementary Material 4. [file 12964_2024_1770_MOESM4_ESM.zip › SENP3 TAM WB/WB-Figure4/B M0 M2 EndoIP/2023-02-18 ─┌╘┤IP shNC shSENP3 IRF4/INPUT IRF4/INPUT IRF4 0.6S 0218.Tif]

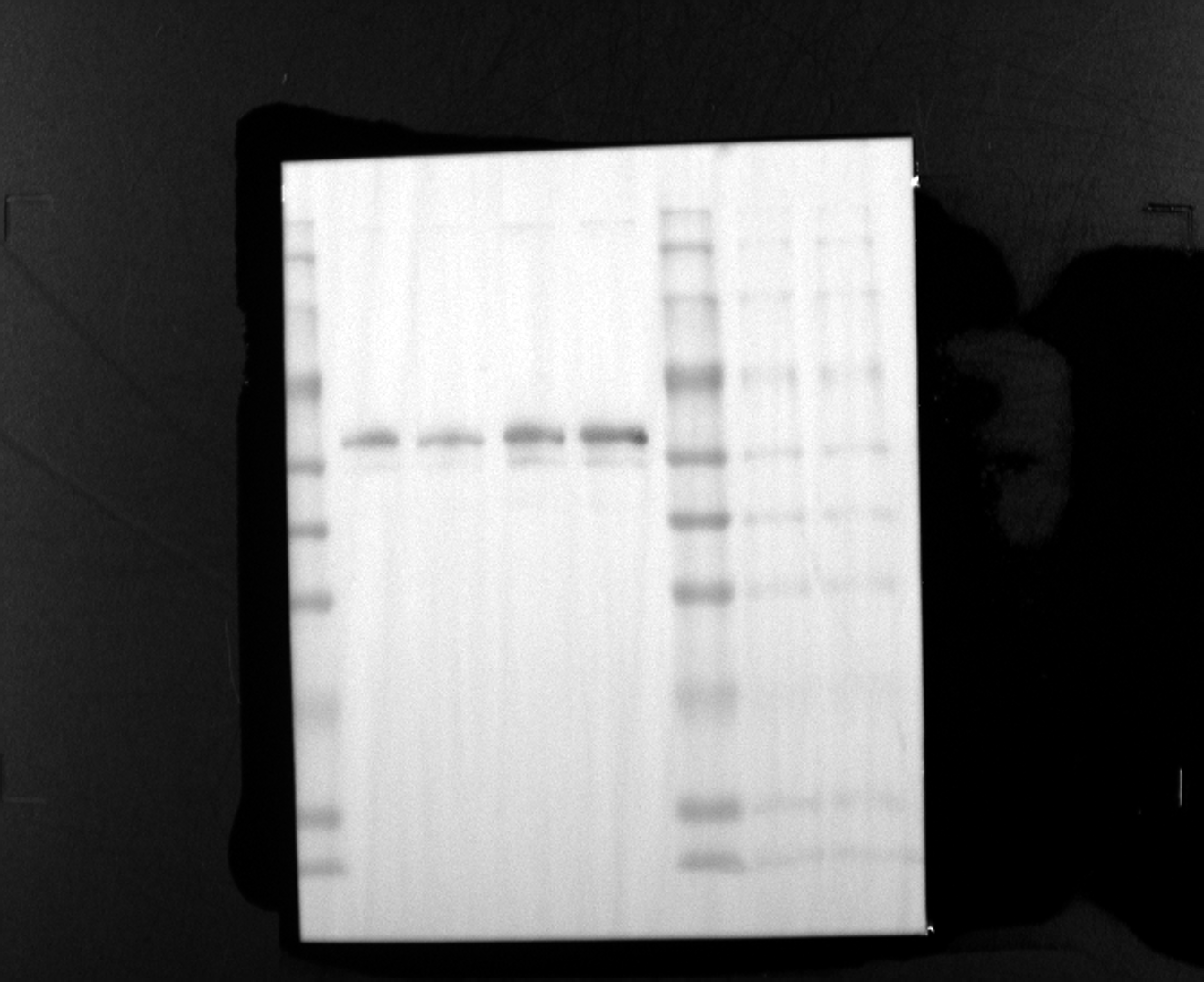

Supplement: Supplementary file 4 — Supplementary Material 4. [file 12964_2024_1770_MOESM4_ESM.zip › SENP3 TAM WB/WB-Figure4/B M0 M2 EndoIP/2023-02-18 ─┌╘┤IP shNC shSENP3 IRF4/INPUT IRF4/INPUT IRF4 0.6S M 0218.Tif]

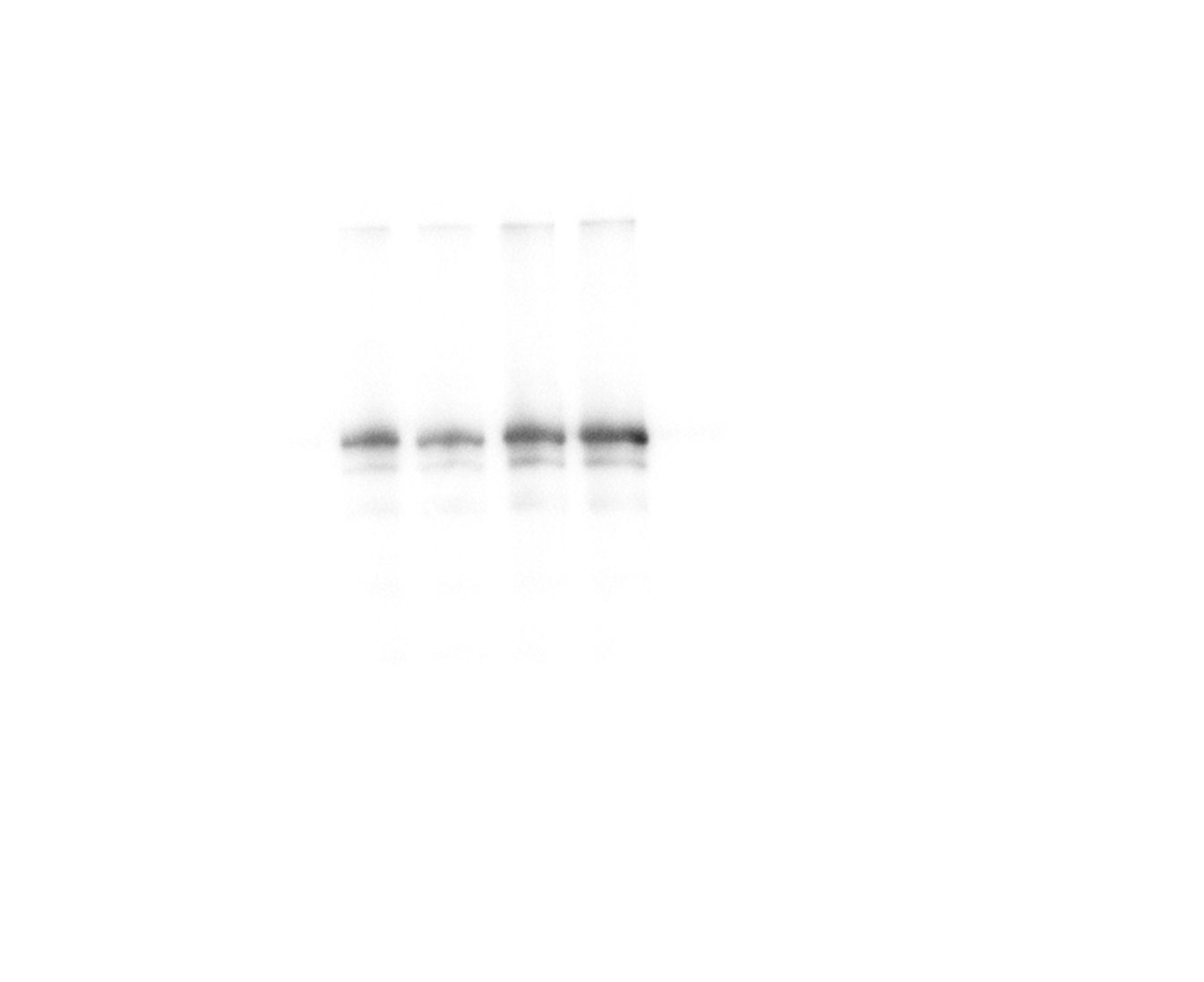

Supplement: Supplementary file 4 — Supplementary Material 4. [file 12964_2024_1770_MOESM4_ESM.zip › SENP3 TAM WB/WB-Figure4/B M0 M2 EndoIP/2023-02-18 ─┌╘┤IP shNC shSENP3 IRF4/INPUT IRF4/INPUT IRF4 0.8S 0218.Tif]

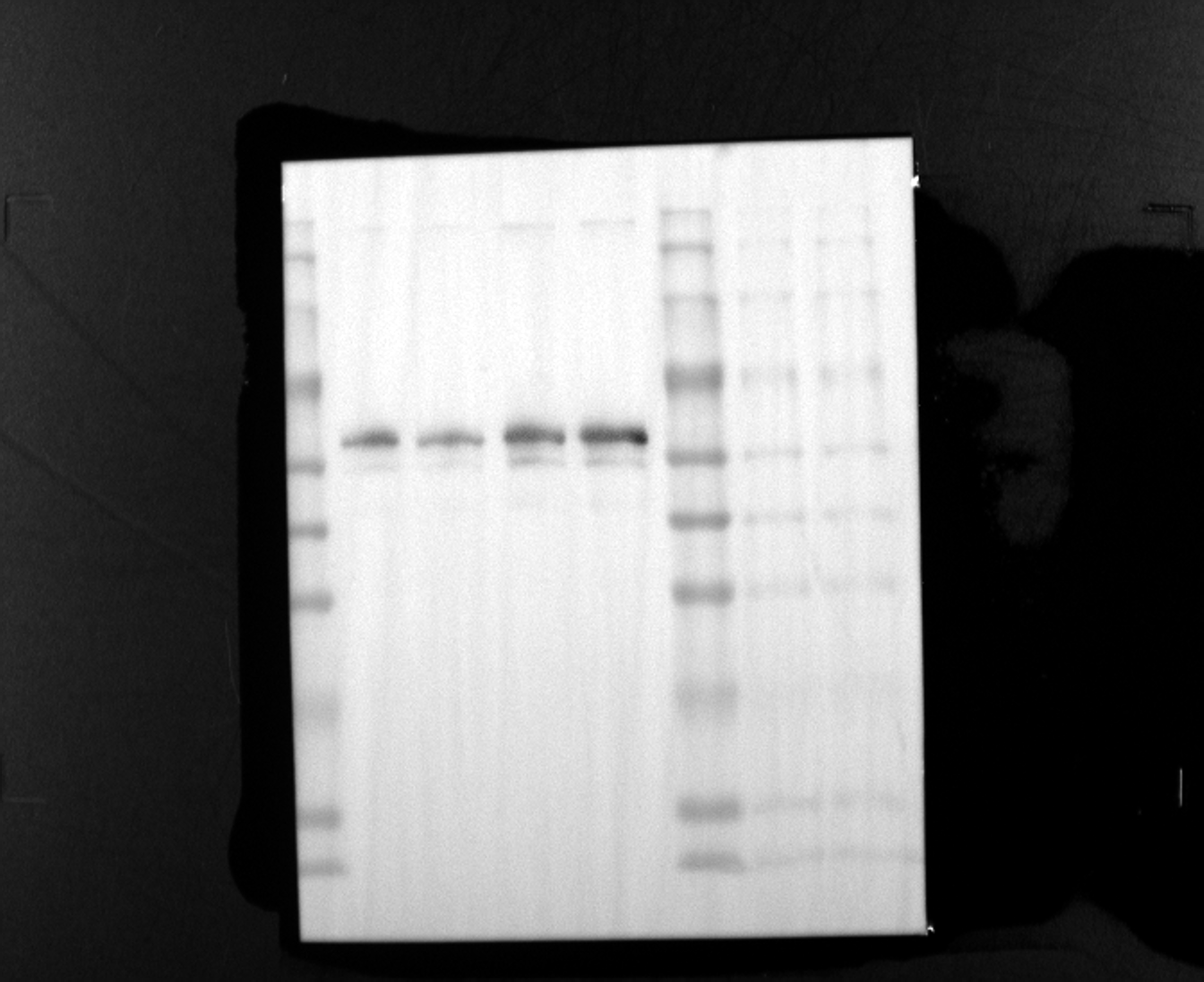

Supplement: Supplementary file 4 — Supplementary Material 4. [file 12964_2024_1770_MOESM4_ESM.zip › SENP3 TAM WB/WB-Figure4/B M0 M2 EndoIP/2023-02-18 ─┌╘┤IP shNC shSENP3 IRF4/INPUT IRF4/INPUT IRF4 0.8S M 0218.Tif]

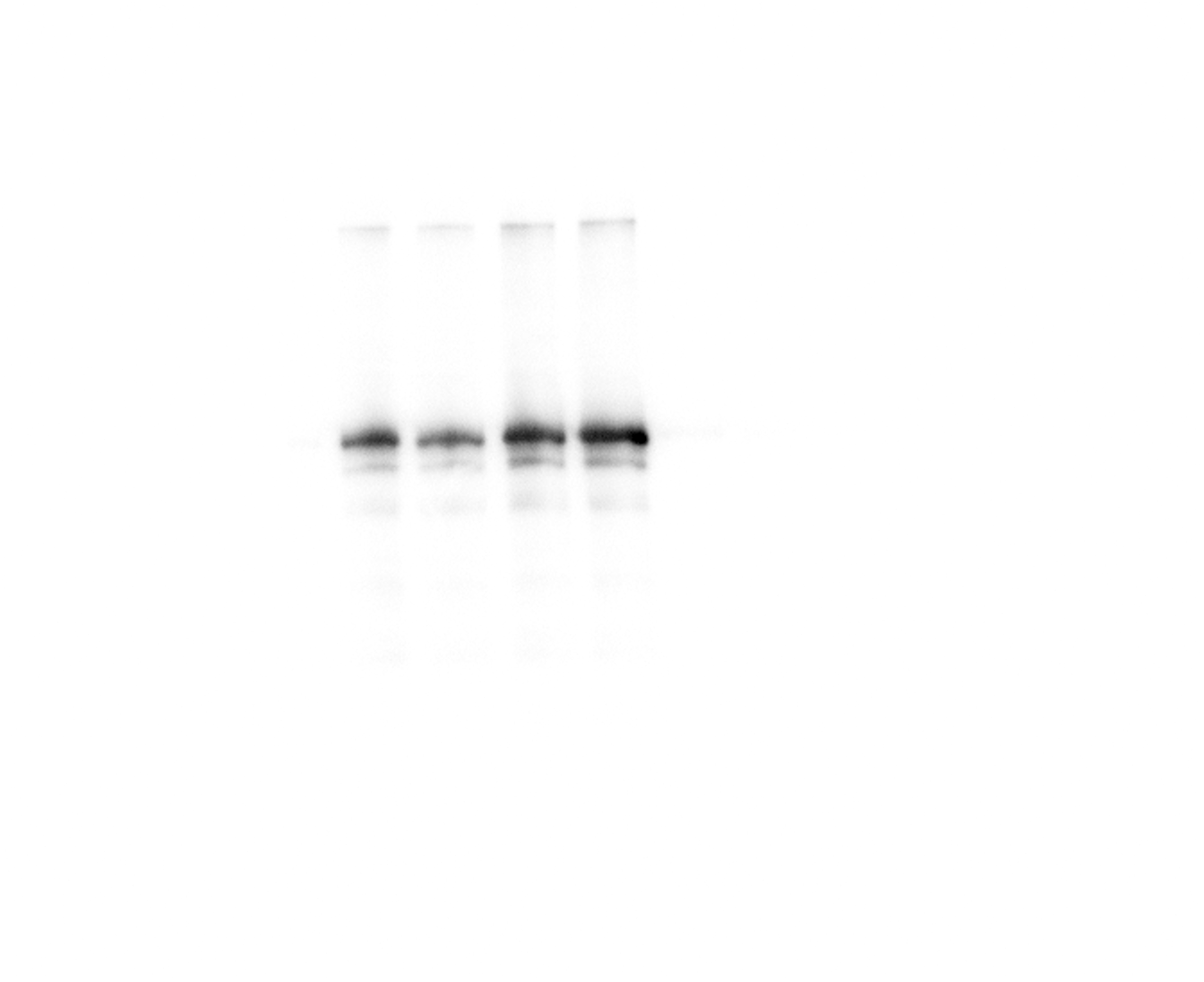

Supplement: Supplementary file 4 — Supplementary Material 4. [file 12964_2024_1770_MOESM4_ESM.zip › SENP3 TAM WB/WB-Figure4/B M0 M2 EndoIP/2023-02-18 ─┌╘┤IP shNC shSENP3 IRF4/INPUT IRF4/INPUT IRF4 1.2S 0218.Tif]

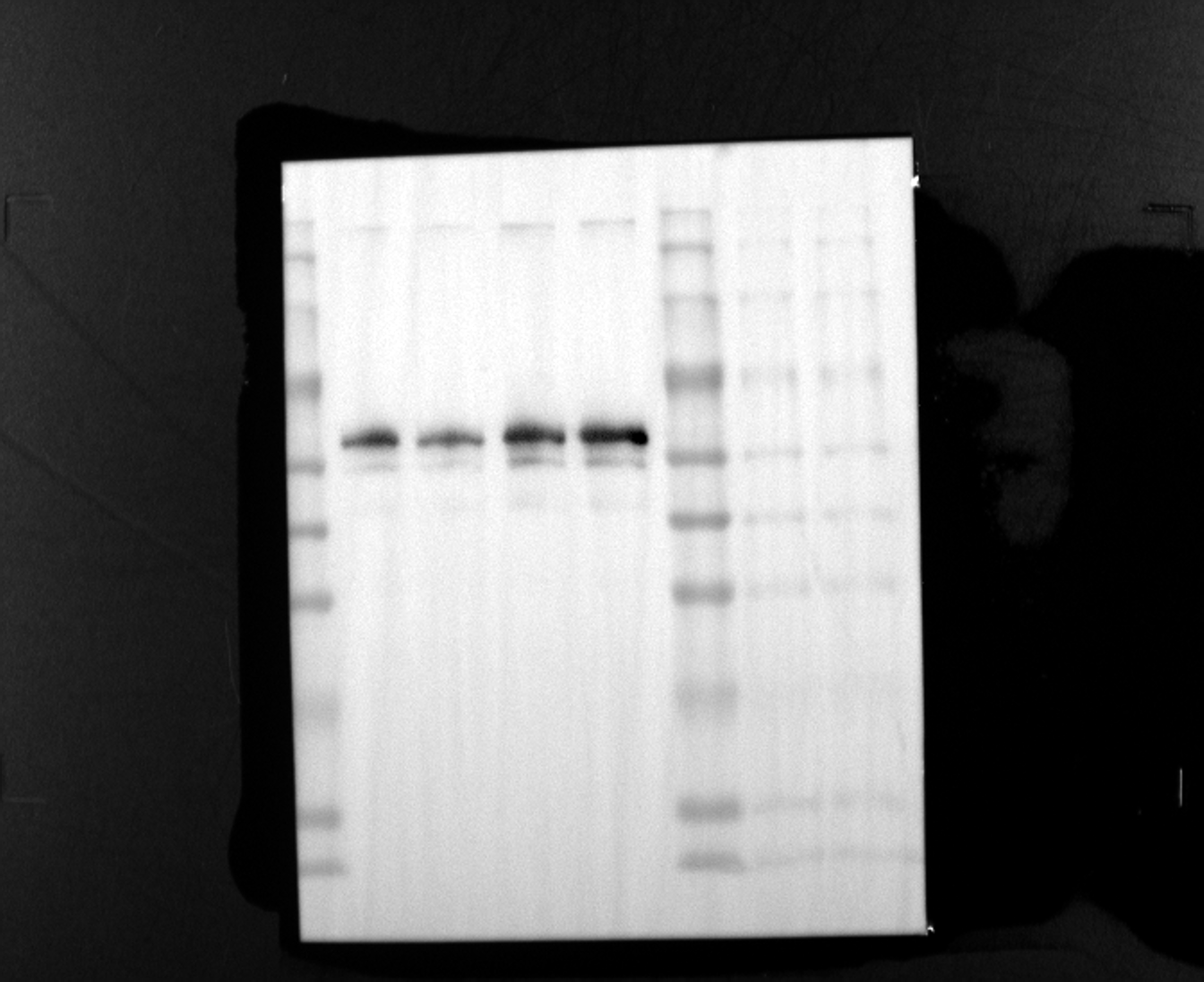

Supplement: Supplementary file 4 — Supplementary Material 4. [file 12964_2024_1770_MOESM4_ESM.zip › SENP3 TAM WB/WB-Figure4/B M0 M2 EndoIP/2023-02-18 ─┌╘┤IP shNC shSENP3 IRF4/INPUT IRF4/INPUT IRF4 1.2S M 0218.Tif]

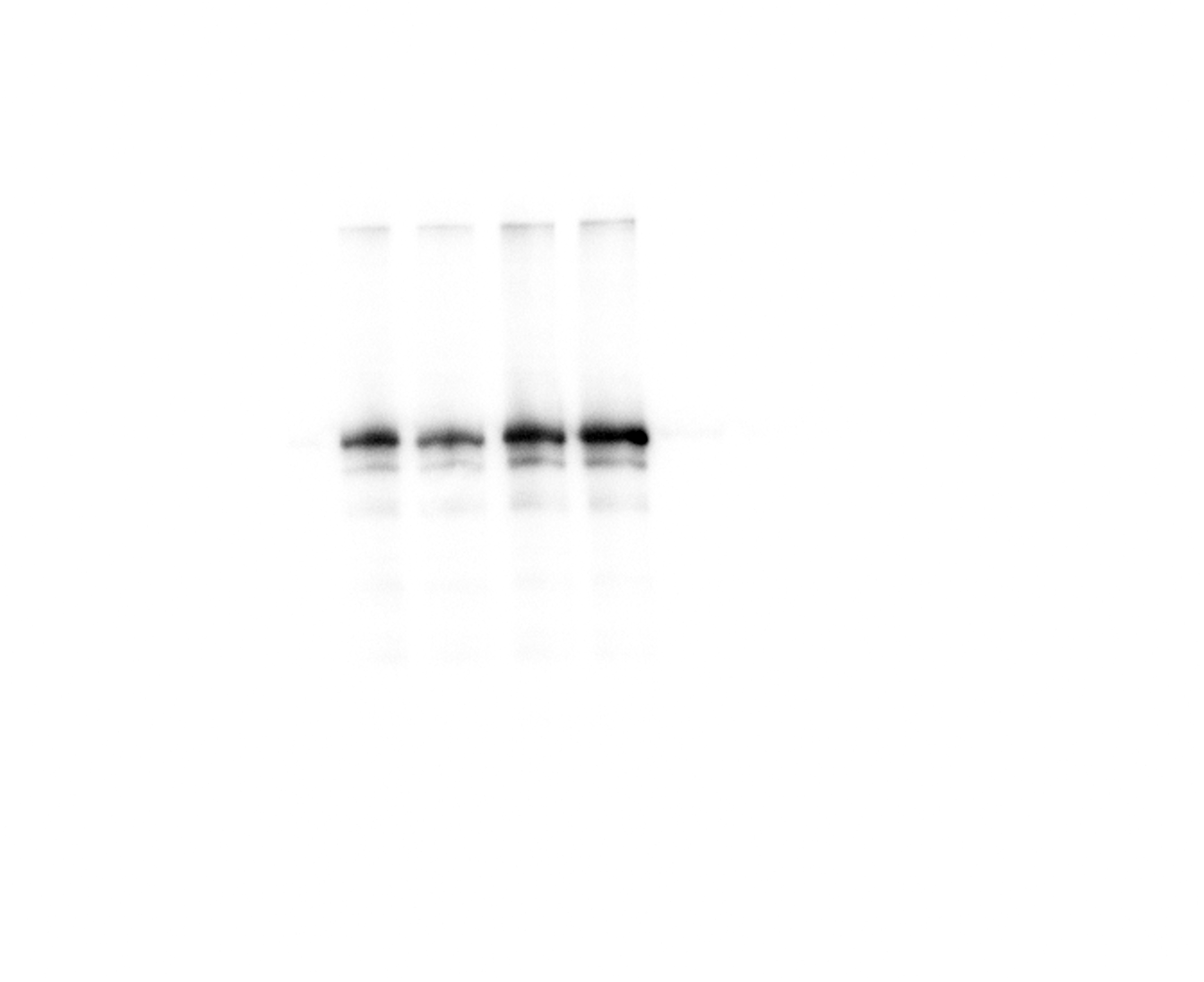

Supplement: Supplementary file 4 — Supplementary Material 4. [file 12964_2024_1770_MOESM4_ESM.zip › SENP3 TAM WB/WB-Figure4/B M0 M2 EndoIP/2023-02-18 ─┌╘┤IP shNC shSENP3 IRF4/INPUT IRF4/INPUT IRF4 1.4S 0218.Tif]

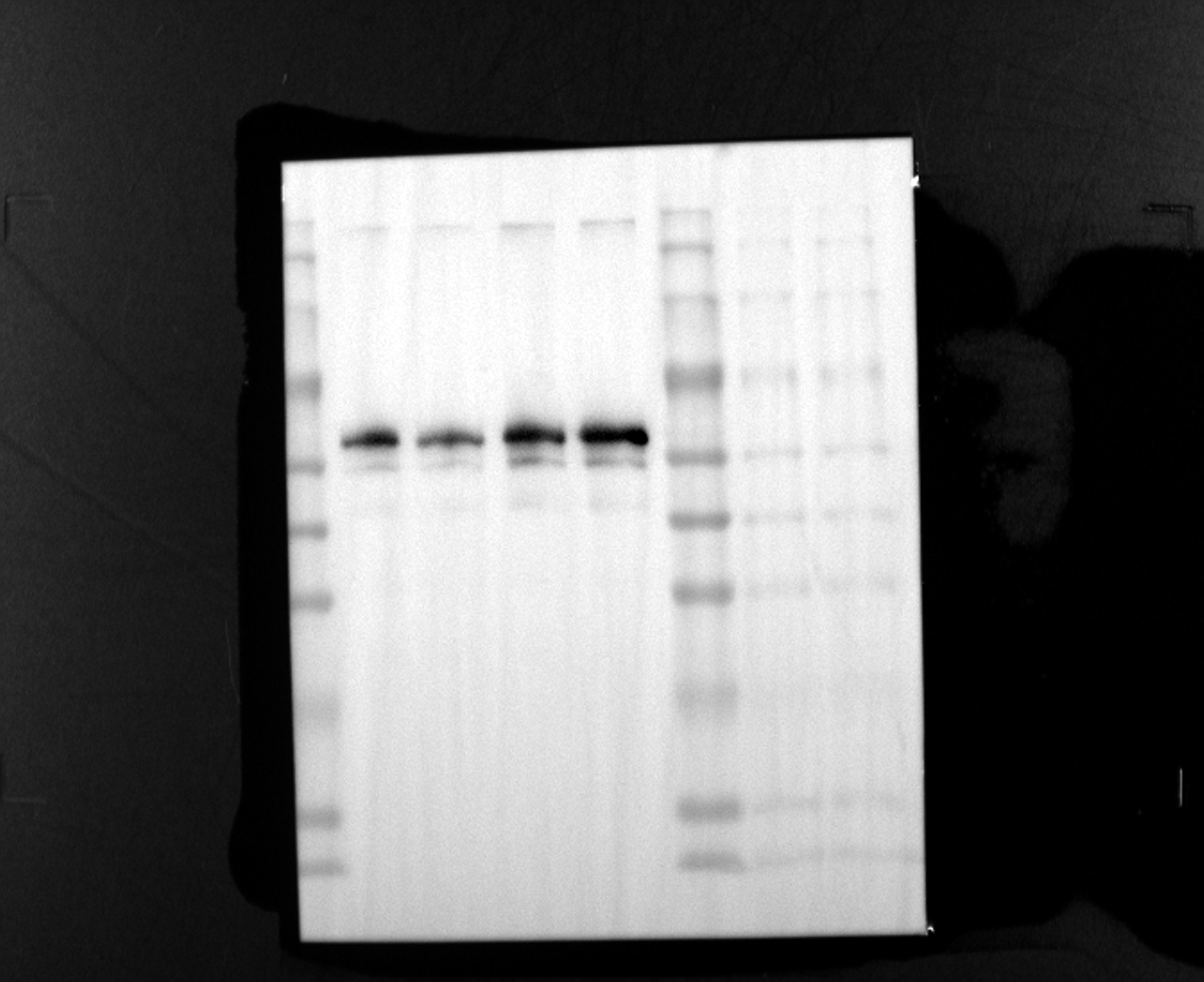

Supplement: Supplementary file 4 — Supplementary Material 4. [file 12964_2024_1770_MOESM4_ESM.zip › SENP3 TAM WB/WB-Figure4/B M0 M2 EndoIP/2023-02-18 ─┌╘┤IP shNC shSENP3 IRF4/INPUT IRF4/INPUT IRF4 1.4S M 0218.Tif]

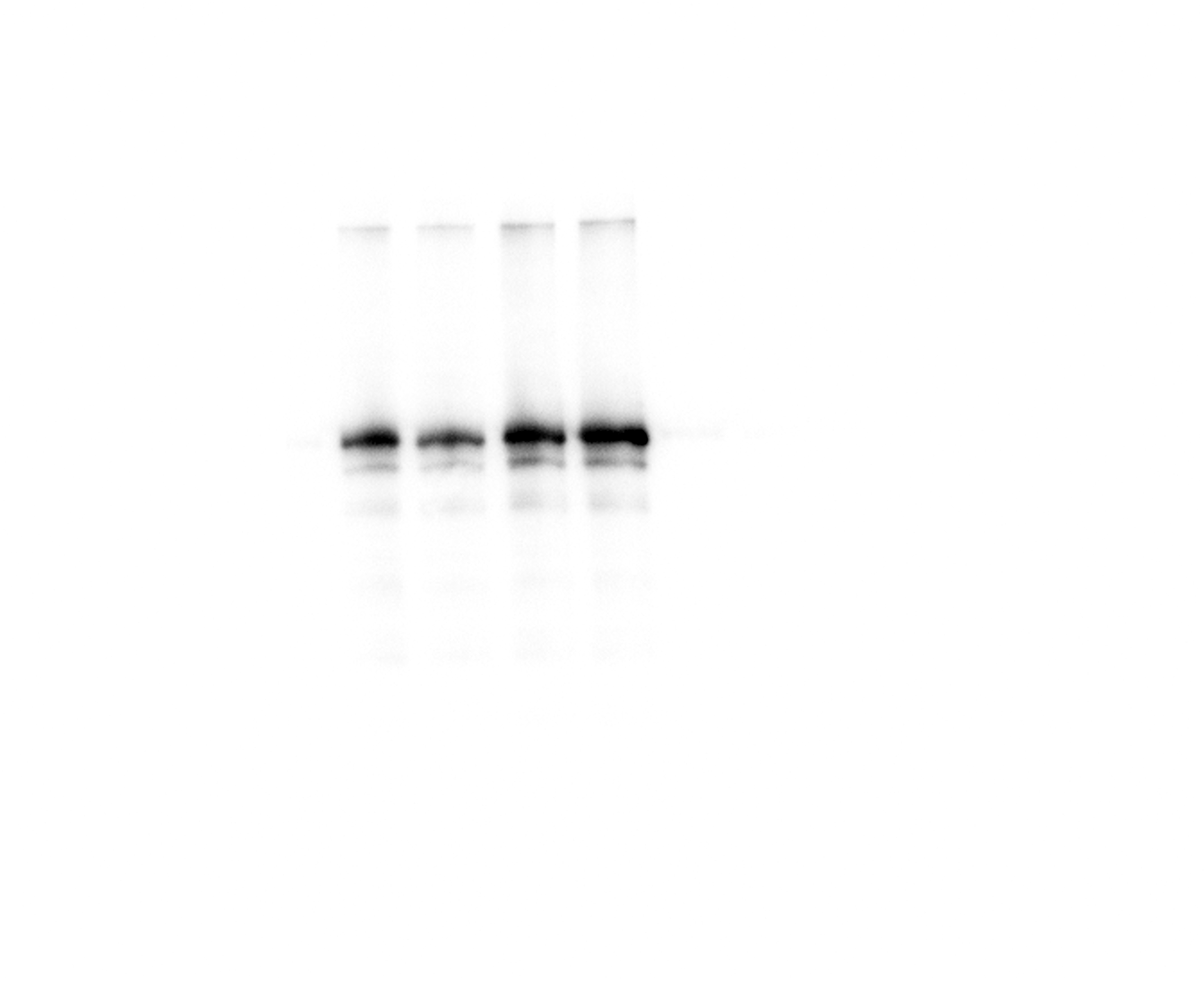

Supplement: Supplementary file 4 — Supplementary Material 4. [file 12964_2024_1770_MOESM4_ESM.zip › SENP3 TAM WB/WB-Figure4/B M0 M2 EndoIP/2023-02-18 ─┌╘┤IP shNC shSENP3 IRF4/INPUT IRF4/INPUT IRF4 1.6S 0218.Tif]

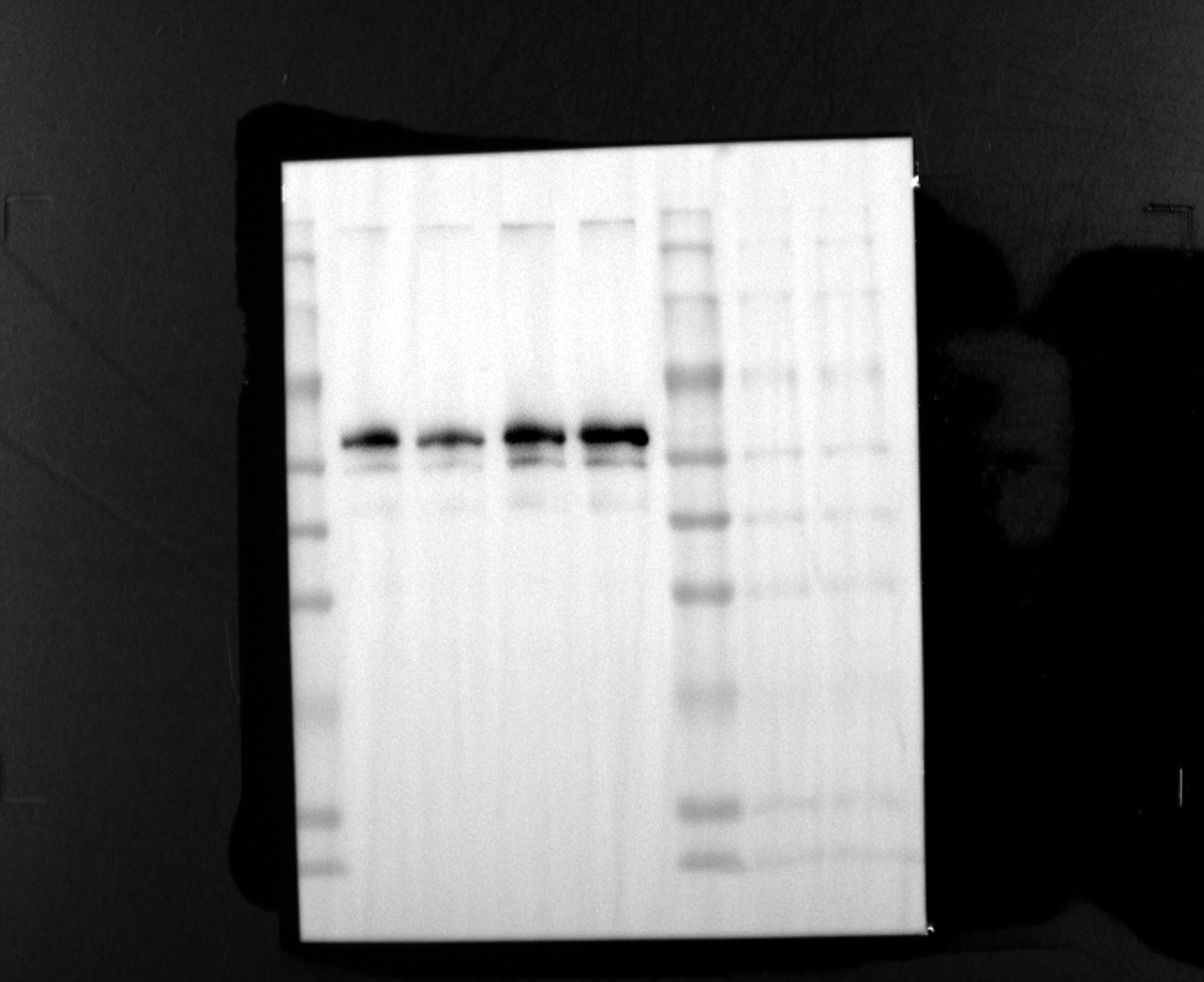

Supplement: Supplementary file 4 — Supplementary Material 4. [file 12964_2024_1770_MOESM4_ESM.zip › SENP3 TAM WB/WB-Figure4/B M0 M2 EndoIP/2023-02-18 ─┌╘┤IP shNC shSENP3 IRF4/INPUT IRF4/INPUT IRF4 1.6S M 0218.Tif]

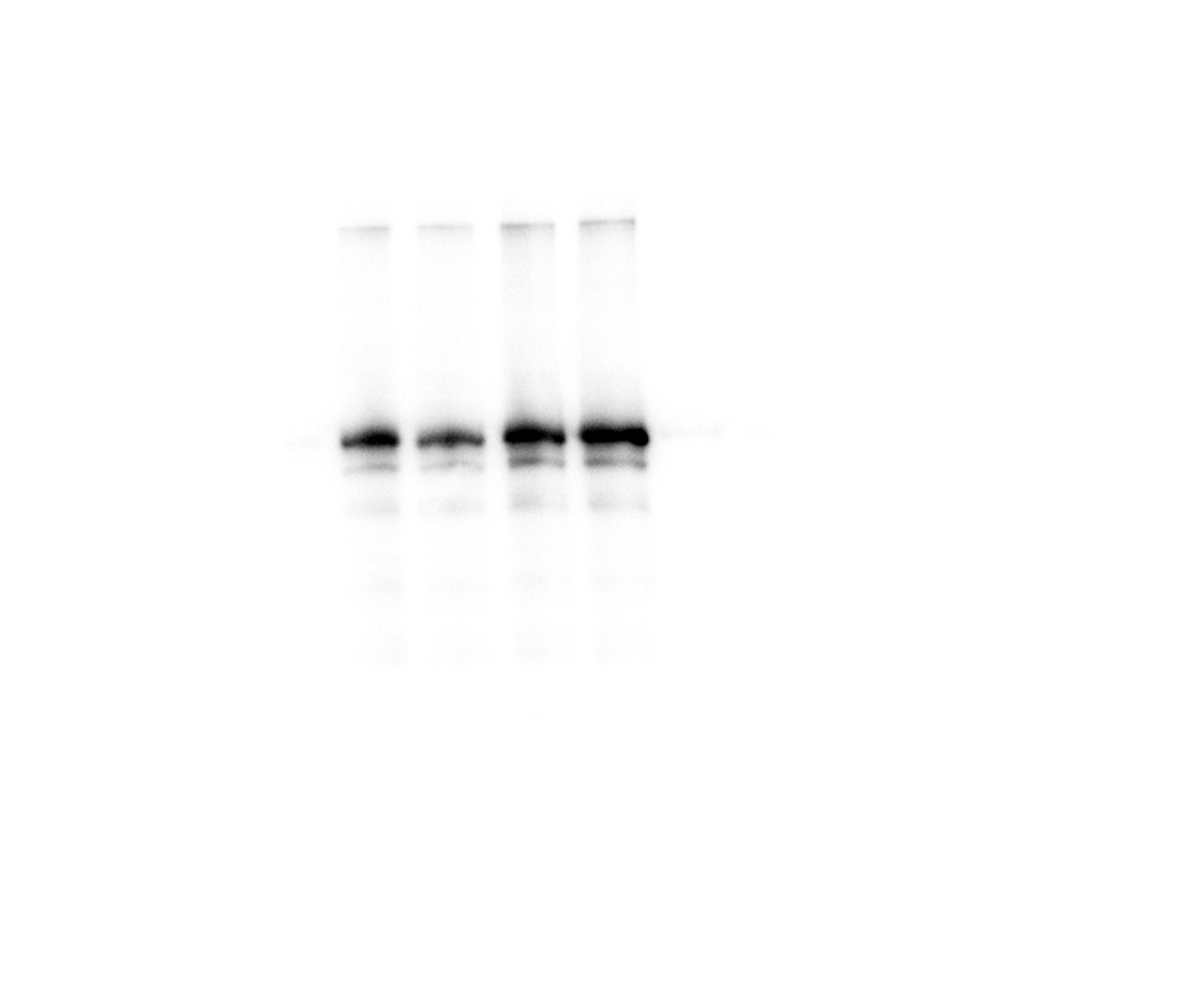

Supplement: Supplementary file 4 — Supplementary Material 4. [file 12964_2024_1770_MOESM4_ESM.zip › SENP3 TAM WB/WB-Figure4/B M0 M2 EndoIP/2023-02-18 ─┌╘┤IP shNC shSENP3 IRF4/INPUT IRF4/INPUT IRF4 1.8S 0218.Tif]

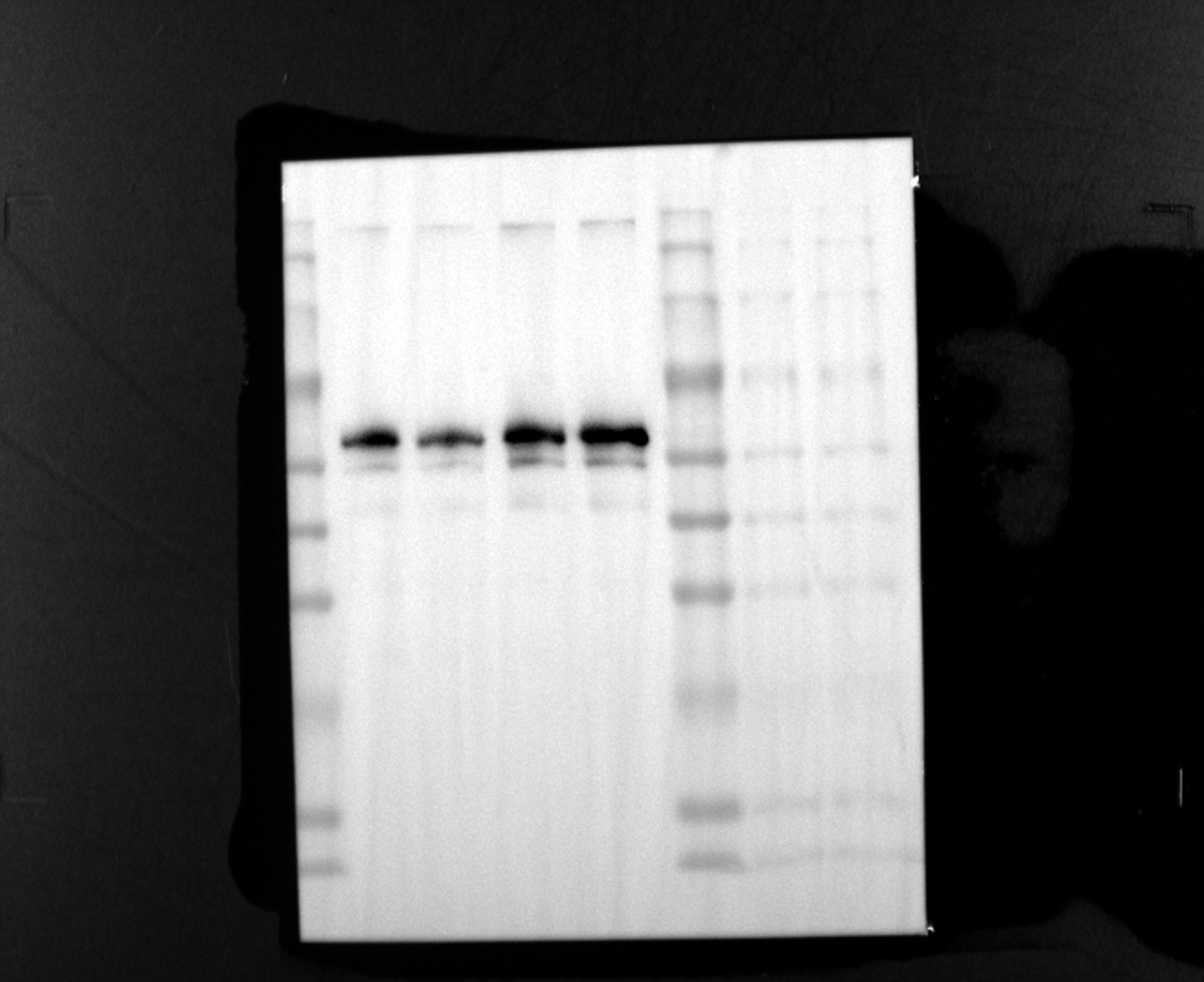

Supplement: Supplementary file 4 — Supplementary Material 4. [file 12964_2024_1770_MOESM4_ESM.zip › SENP3 TAM WB/WB-Figure4/B M0 M2 EndoIP/2023-02-18 ─┌╘┤IP shNC shSENP3 IRF4/INPUT IRF4/INPUT IRF4 1.8S M 0218.Tif]

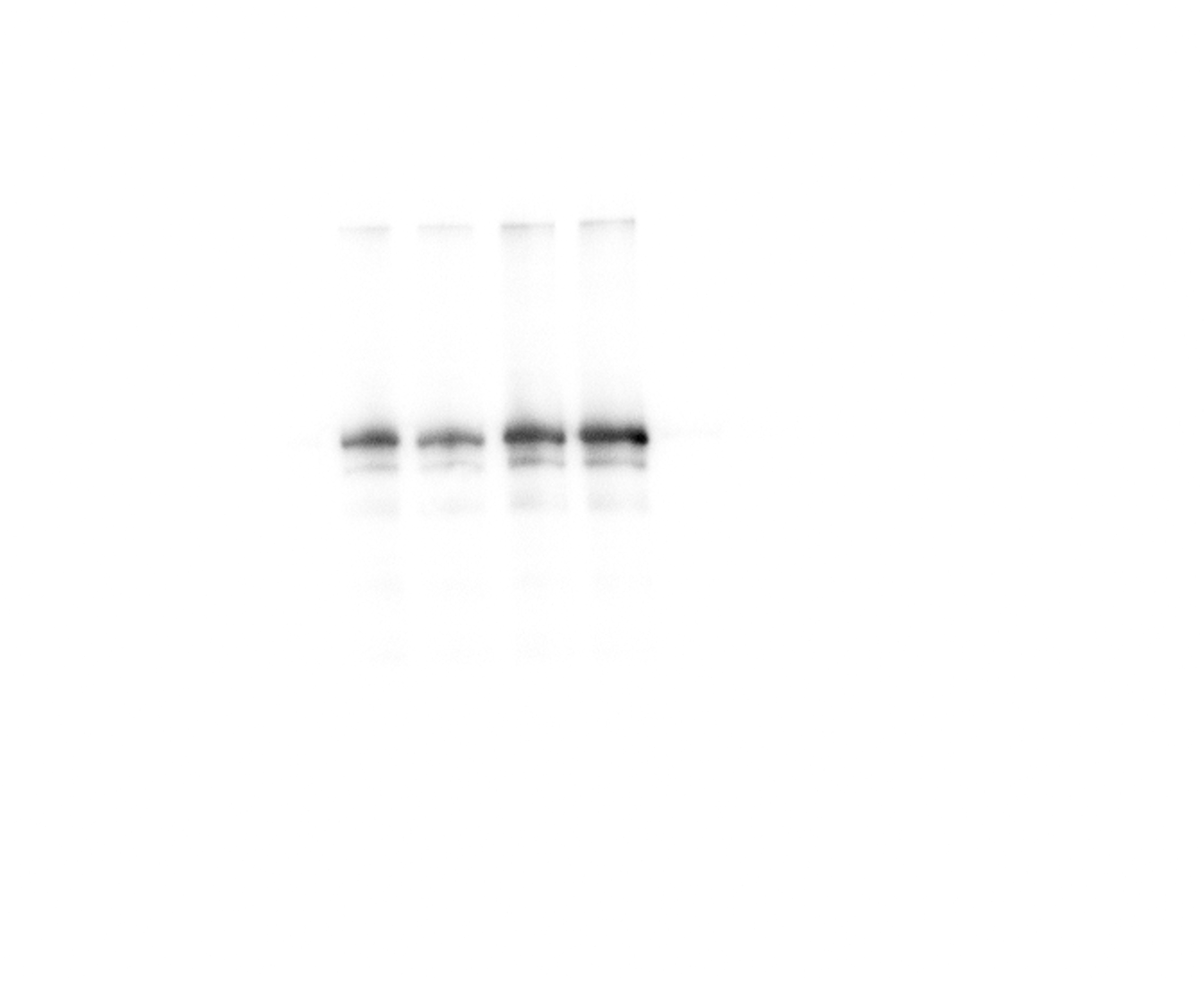

Supplement: Supplementary file 4 — Supplementary Material 4. [file 12964_2024_1770_MOESM4_ESM.zip › SENP3 TAM WB/WB-Figure4/B M0 M2 EndoIP/2023-02-18 ─┌╘┤IP shNC shSENP3 IRF4/INPUT IRF4/INPUT IRF4 1S 0218.Tif]

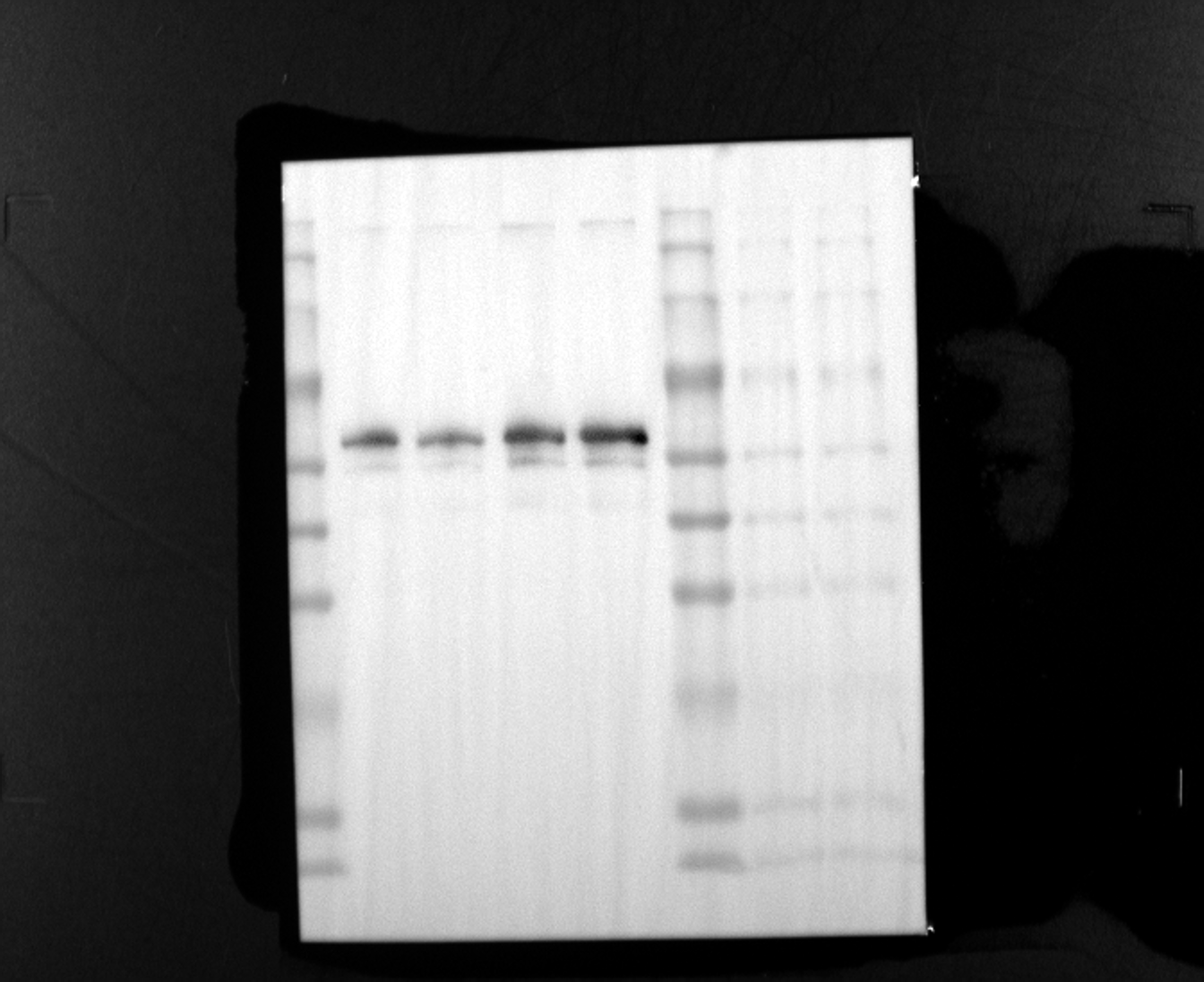

Supplement: Supplementary file 4 — Supplementary Material 4. [file 12964_2024_1770_MOESM4_ESM.zip › SENP3 TAM WB/WB-Figure4/B M0 M2 EndoIP/2023-02-18 ─┌╘┤IP shNC shSENP3 IRF4/INPUT IRF4/INPUT IRF4 1S M 0218.Tif]

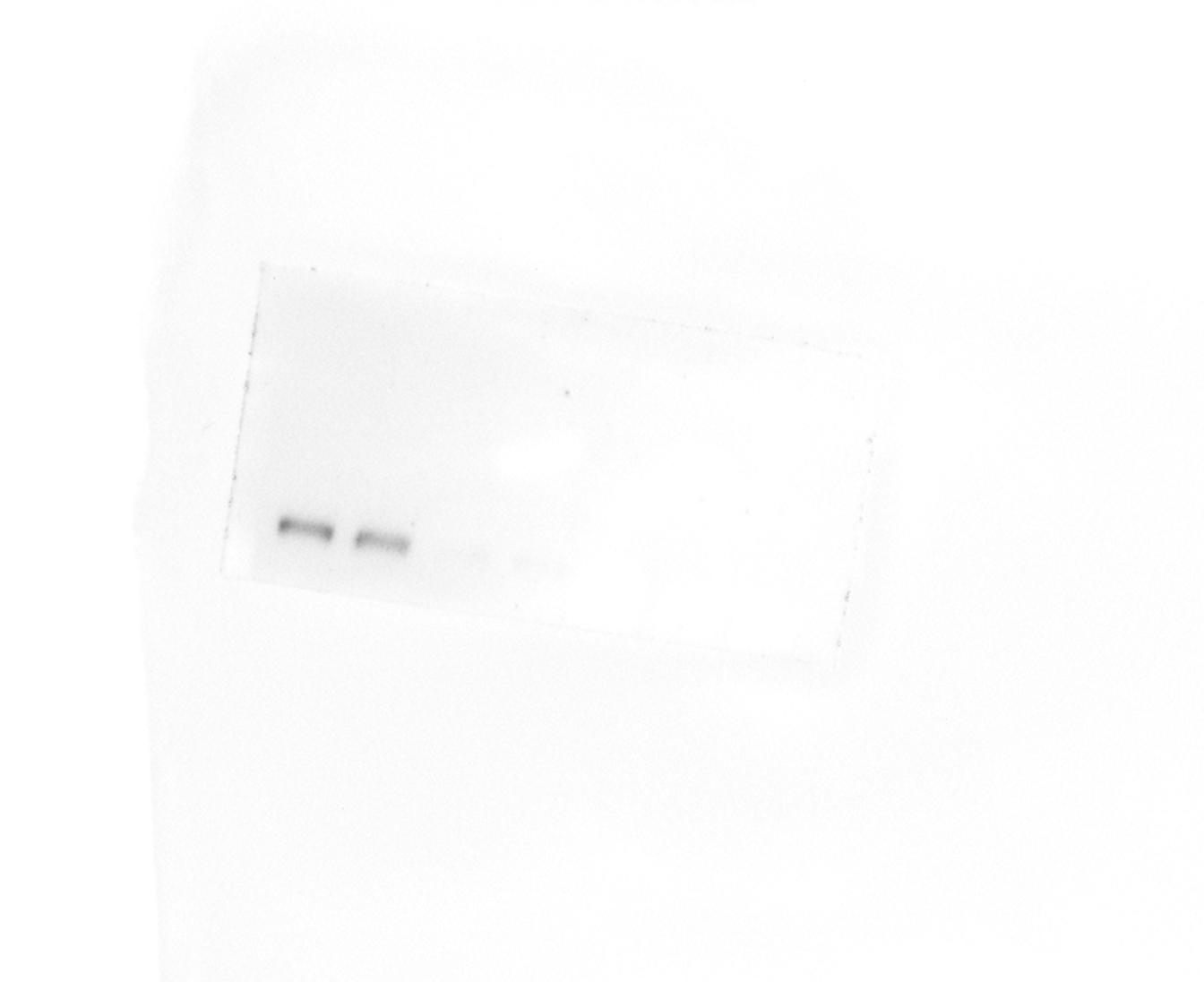

Supplement: Supplementary file 4 — Supplementary Material 4. [file 12964_2024_1770_MOESM4_ESM.zip › SENP3 TAM WB/WB-Figure4/B M0 M2 EndoIP/2023-02-18 ─┌╘┤IP shNC shSENP3 IRF4/INPUT SENP3 0225/INPUT SENP3 40S 0225.Tif]

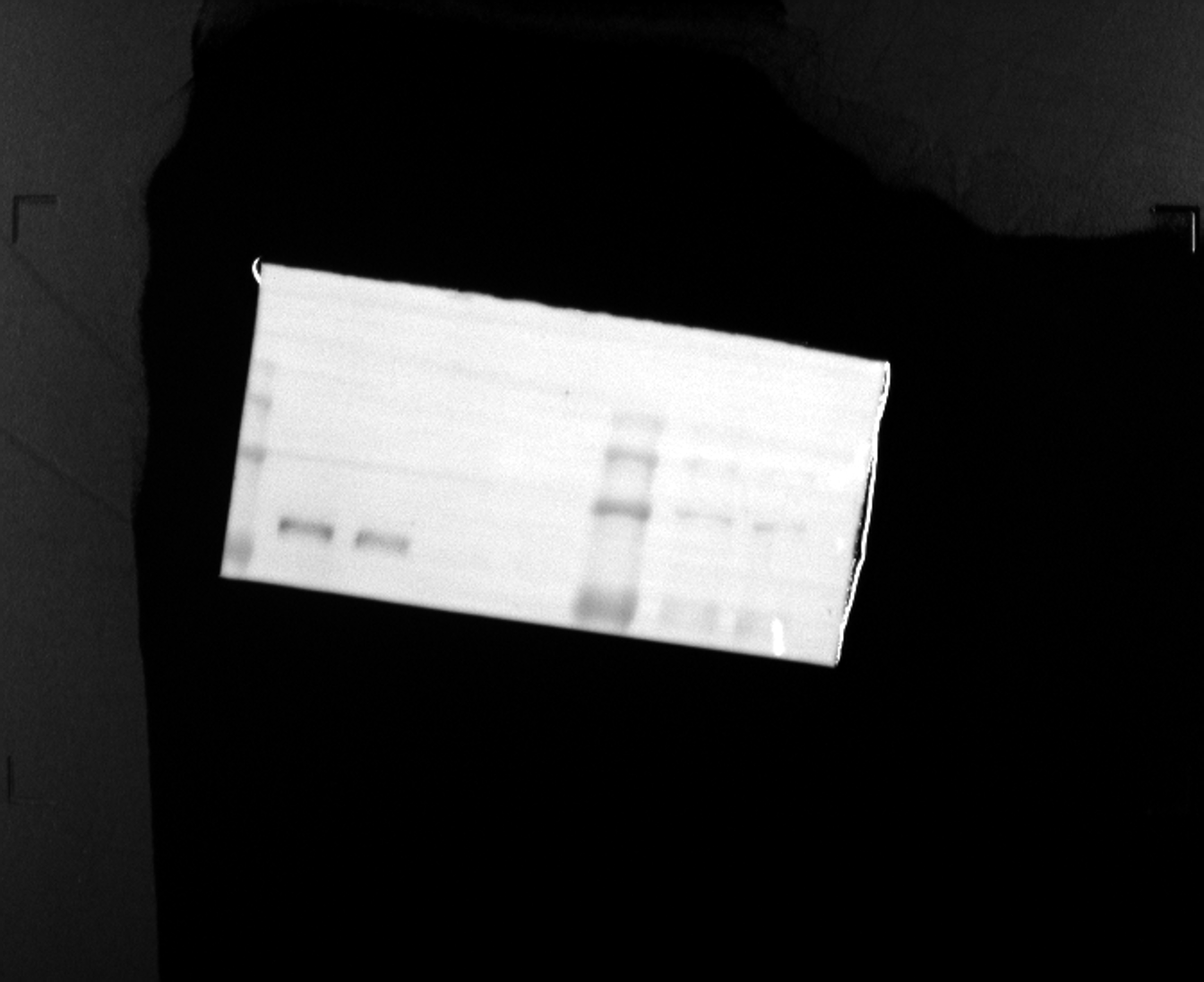

Supplement: Supplementary file 4 — Supplementary Material 4. [file 12964_2024_1770_MOESM4_ESM.zip › SENP3 TAM WB/WB-Figure4/B M0 M2 EndoIP/2023-02-18 ─┌╘┤IP shNC shSENP3 IRF4/INPUT SENP3 0225/INPUT SENP3 40S M 0225.Tif]

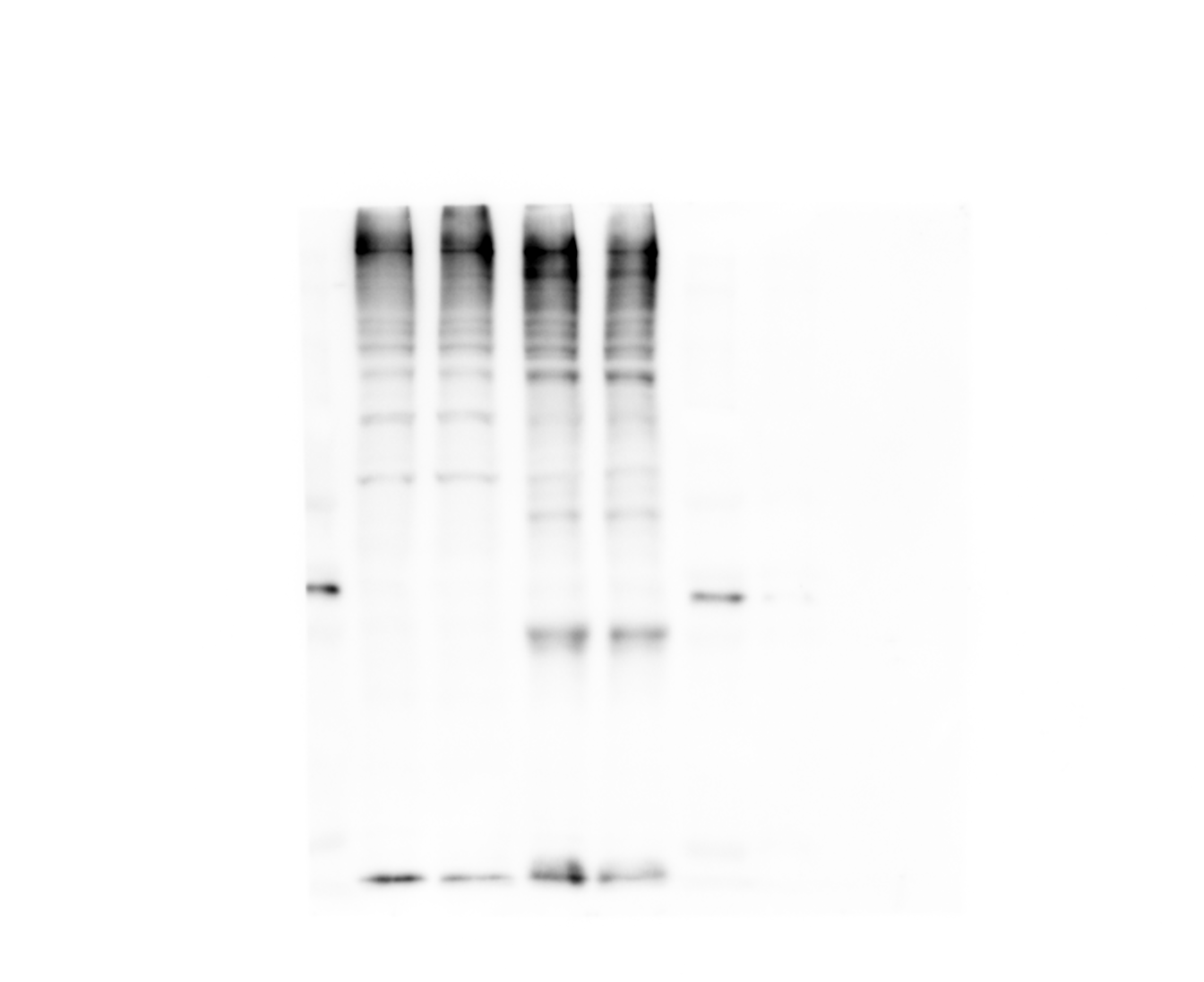

Supplement: Supplementary file 4 — Supplementary Material 4. [file 12964_2024_1770_MOESM4_ESM.zip › SENP3 TAM WB/WB-Figure4/B M0 M2 EndoIP/2023-02-18 ─┌╘┤IP shNC shSENP3 IRF4/INPUT SUMO23 0221/INPUT SUMO23 10S 0221.Tif]

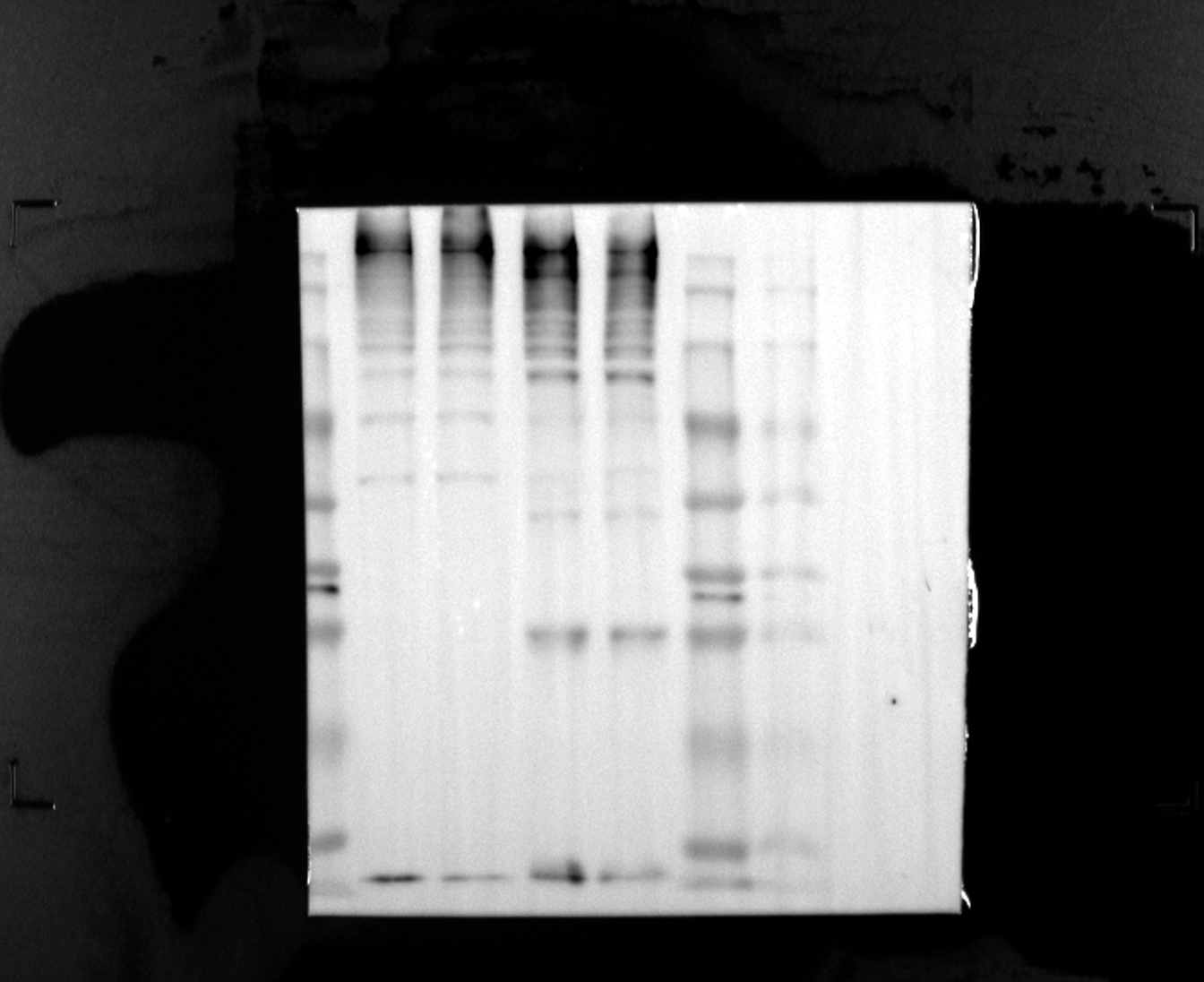

Supplement: Supplementary file 4 — Supplementary Material 4. [file 12964_2024_1770_MOESM4_ESM.zip › SENP3 TAM WB/WB-Figure4/B M0 M2 EndoIP/2023-02-18 ─┌╘┤IP shNC shSENP3 IRF4/INPUT SUMO23 0221/INPUT SUMO23 10S M 0221.Tif]

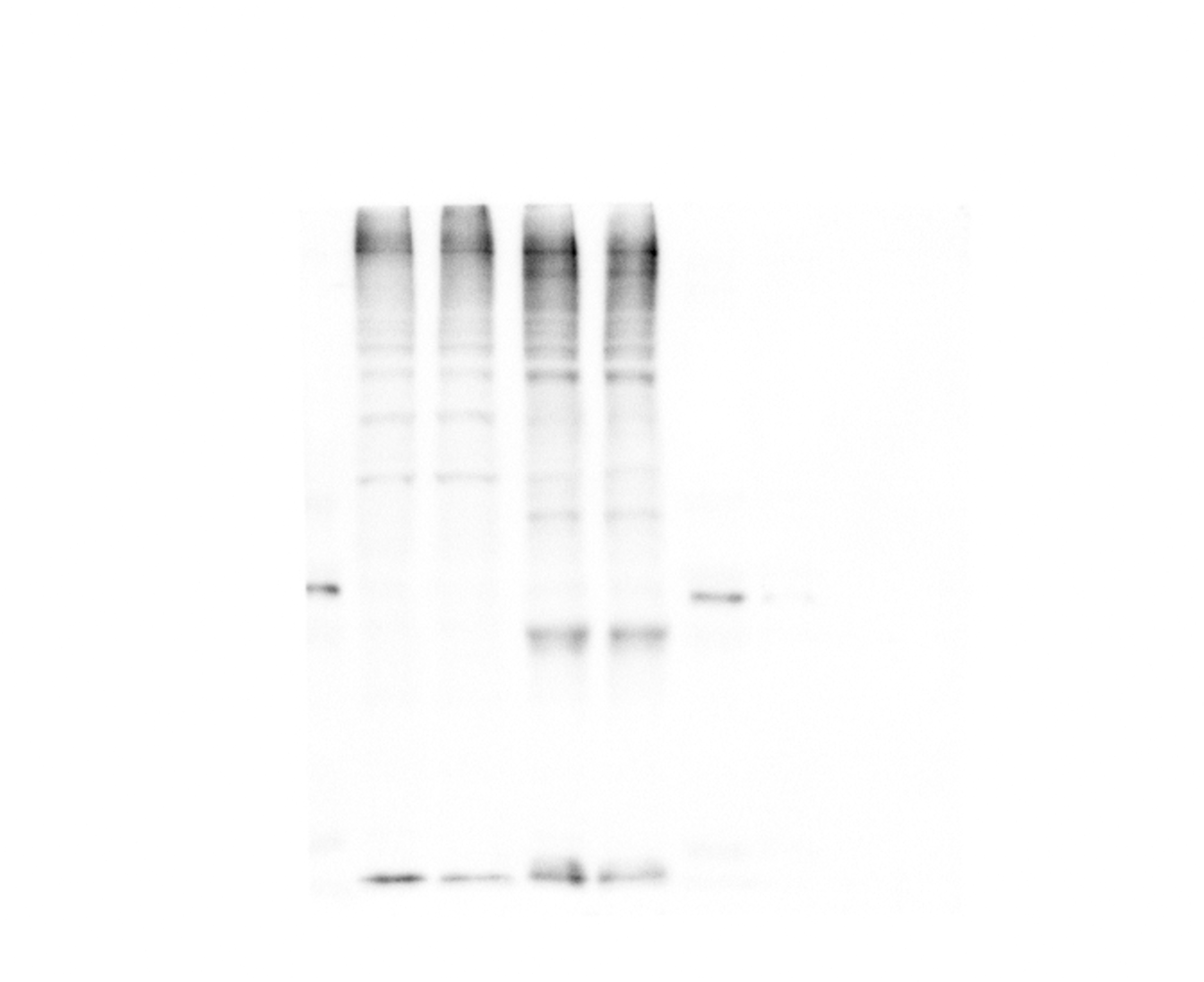

Supplement: Supplementary file 4 — Supplementary Material 4. [file 12964_2024_1770_MOESM4_ESM.zip › SENP3 TAM WB/WB-Figure4/B M0 M2 EndoIP/2023-02-18 ─┌╘┤IP shNC shSENP3 IRF4/INPUT SUMO23 0221/INPUT SUMO23 1S 0221.Tif]

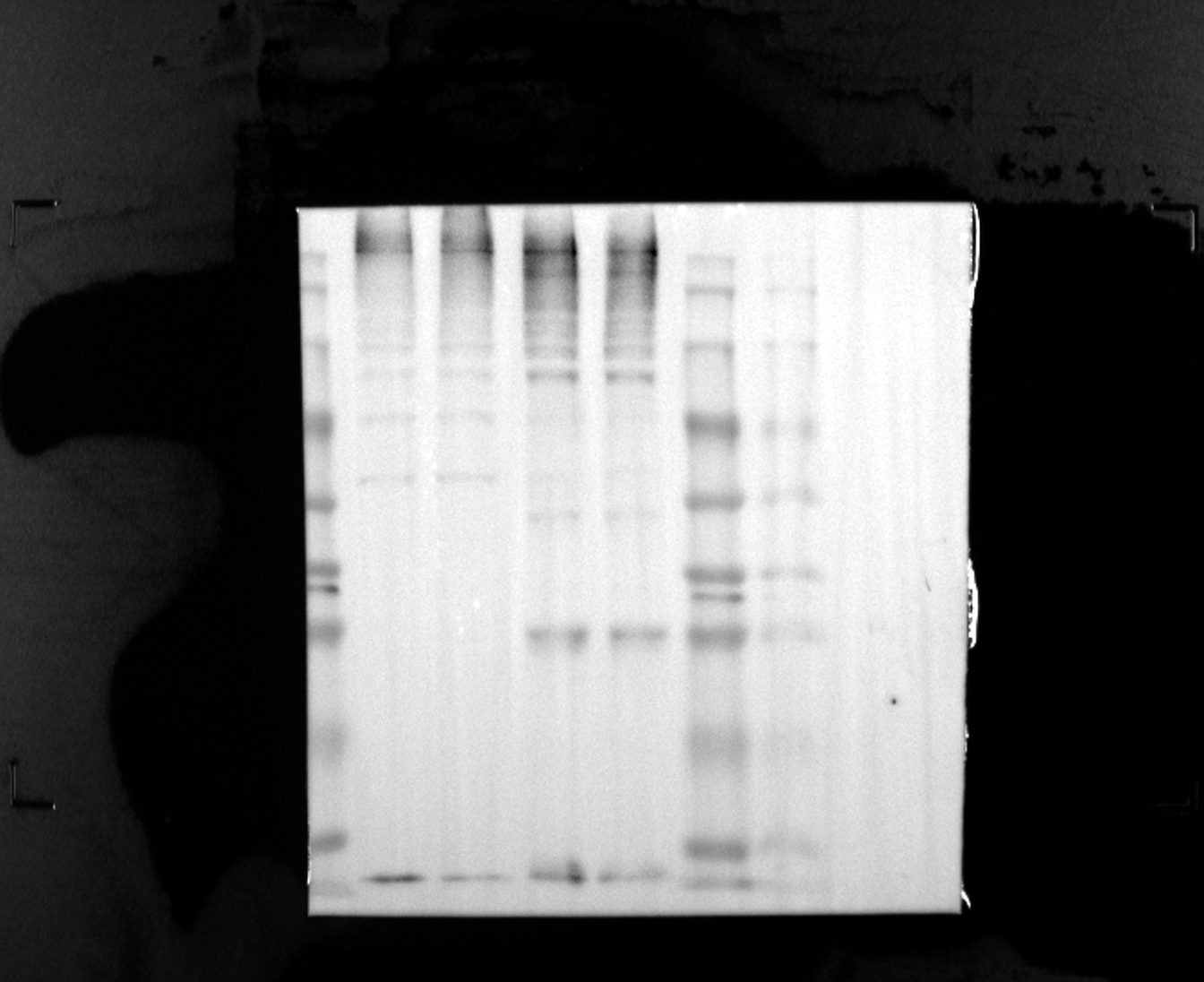

Supplement: Supplementary file 4 — Supplementary Material 4. [file 12964_2024_1770_MOESM4_ESM.zip › SENP3 TAM WB/WB-Figure4/B M0 M2 EndoIP/2023-02-18 ─┌╘┤IP shNC shSENP3 IRF4/INPUT SUMO23 0221/INPUT SUMO23 1S M 0221.Tif]

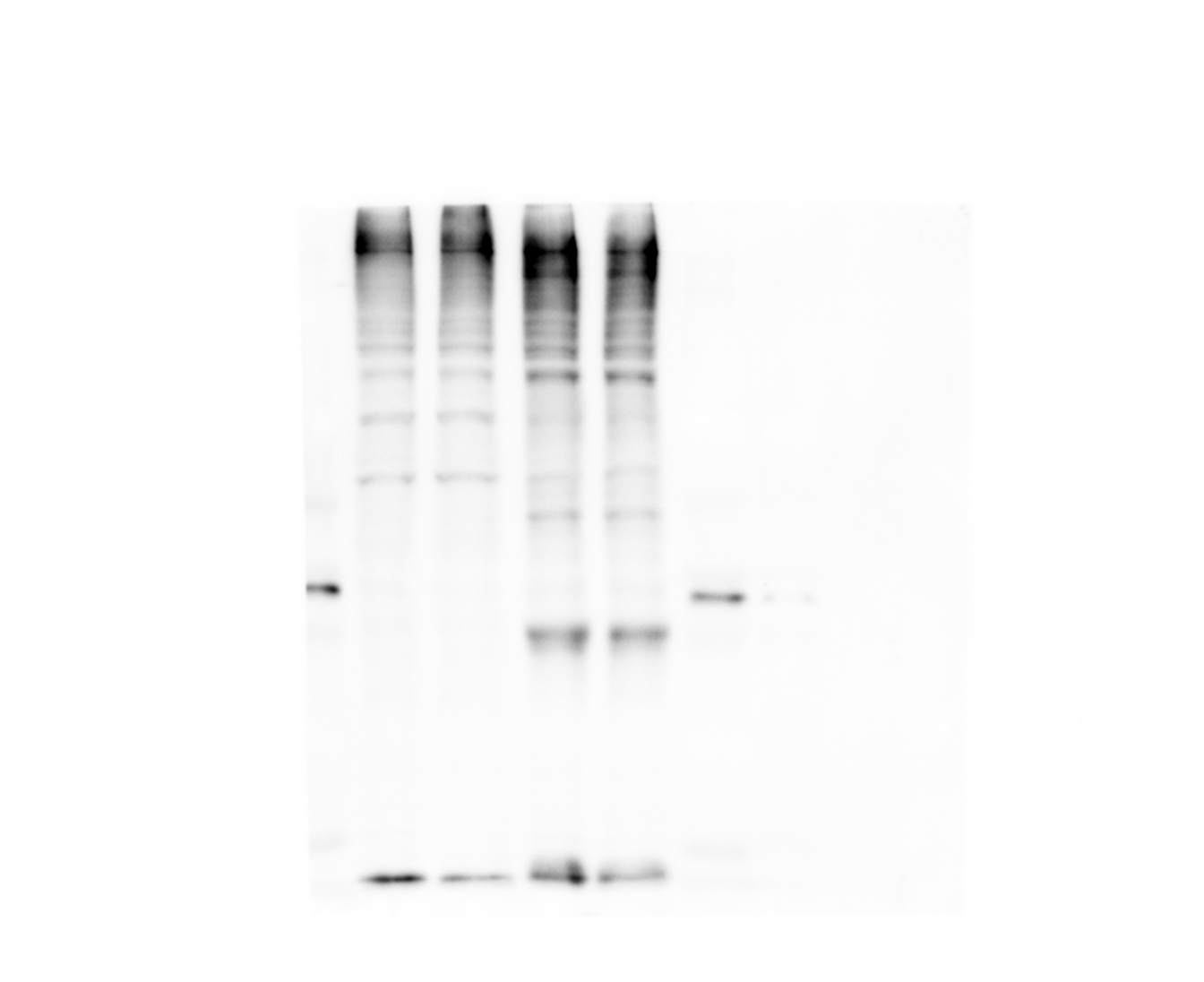

Supplement: Supplementary file 4 — Supplementary Material 4. [file 12964_2024_1770_MOESM4_ESM.zip › SENP3 TAM WB/WB-Figure4/B M0 M2 EndoIP/2023-02-18 ─┌╘┤IP shNC shSENP3 IRF4/INPUT SUMO23 0221/INPUT SUMO23 7S 0221.Tif]

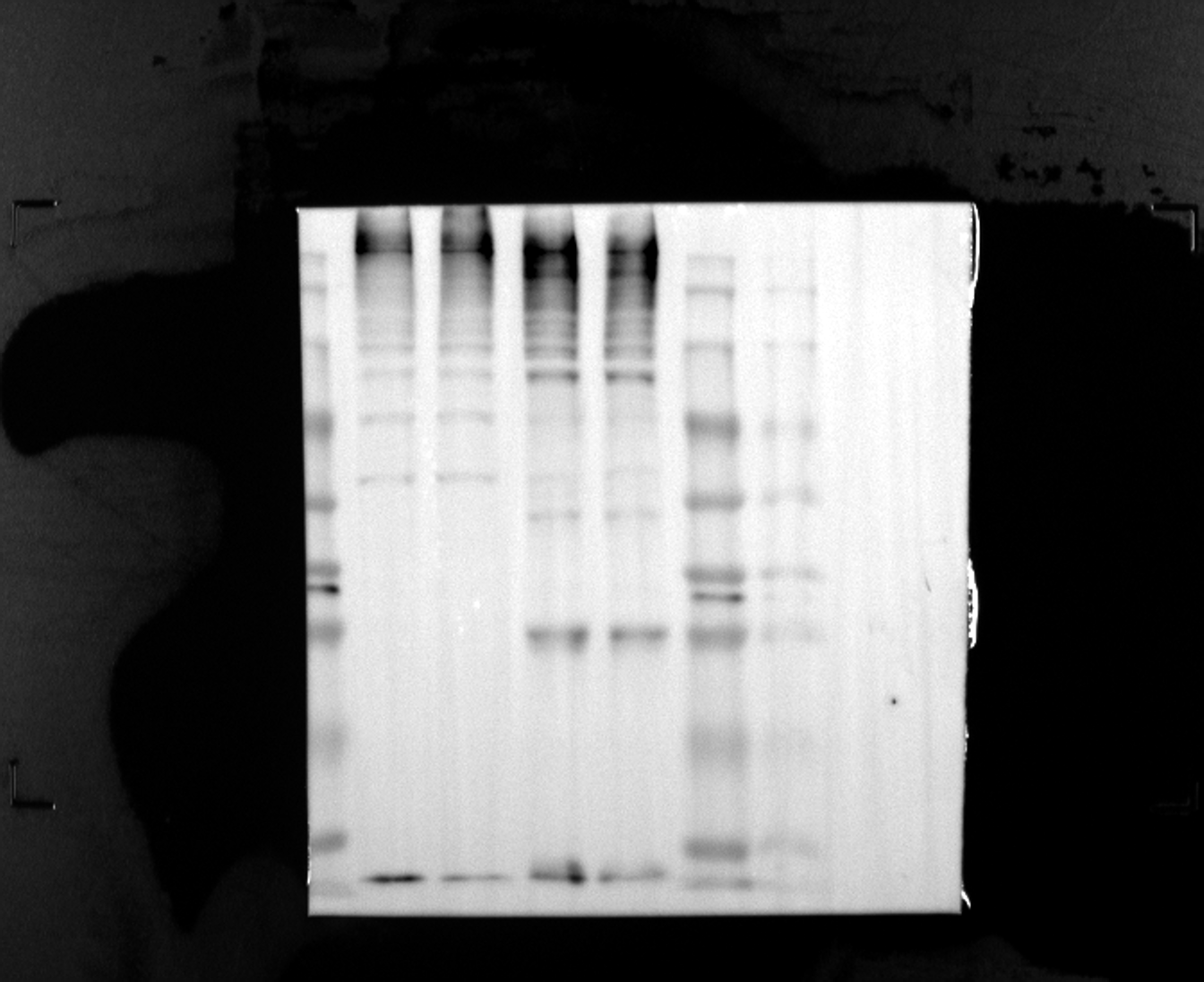

Supplement: Supplementary file 4 — Supplementary Material 4. [file 12964_2024_1770_MOESM4_ESM.zip › SENP3 TAM WB/WB-Figure4/B M0 M2 EndoIP/2023-02-18 ─┌╘┤IP shNC shSENP3 IRF4/INPUT SUMO23 0221/INPUT SUMO23 7S M 0221.Tif]

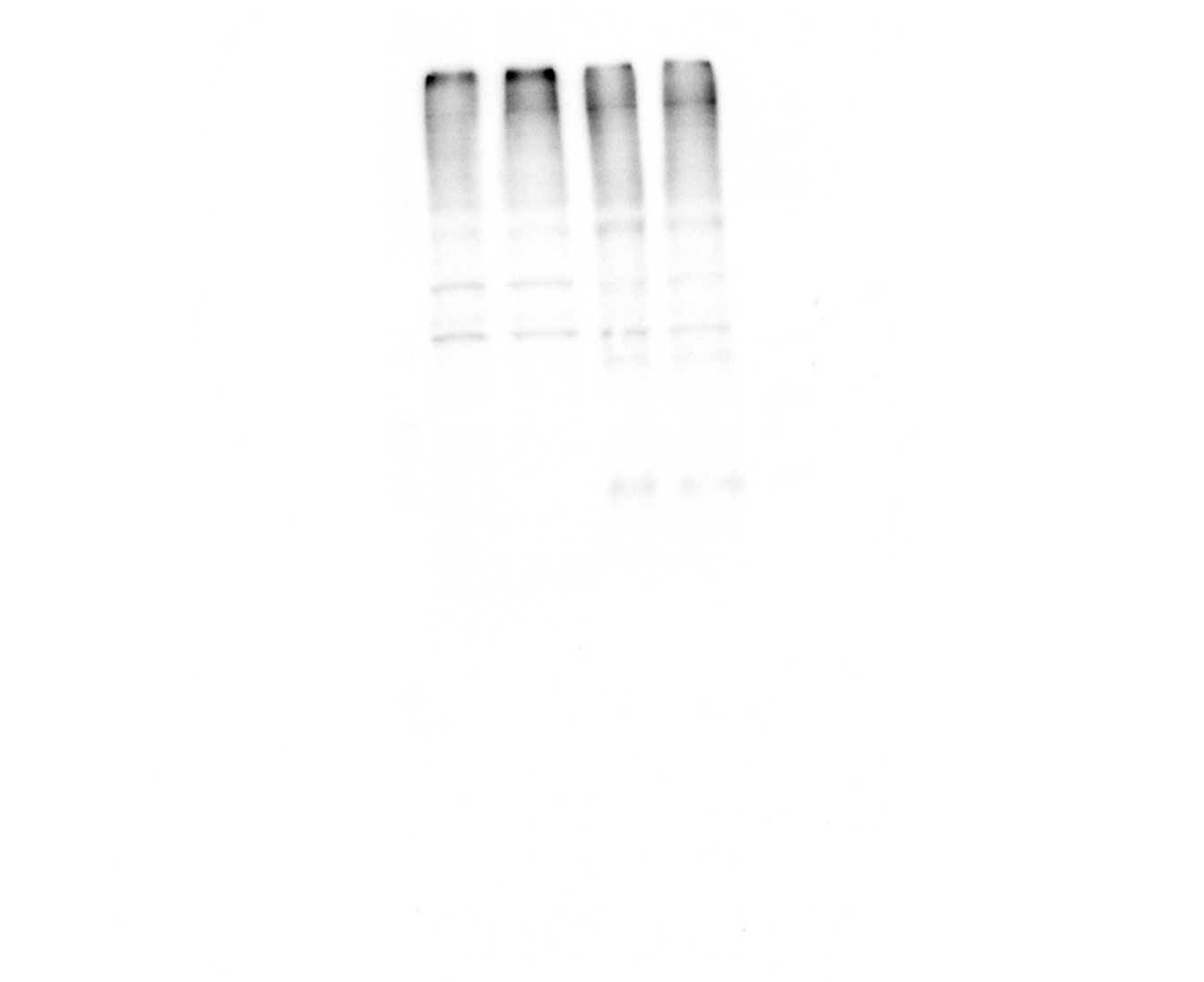

Supplement: Supplementary file 4 — Supplementary Material 4. [file 12964_2024_1770_MOESM4_ESM.zip › SENP3 TAM WB/WB-Figure4/B M0 M2 EndoIP/2023-02-18 ─┌╘┤IP shNC shSENP3 IRF4/INPUT SUMO23 0222/INPUT SUMO23 2S 0222.Tif]

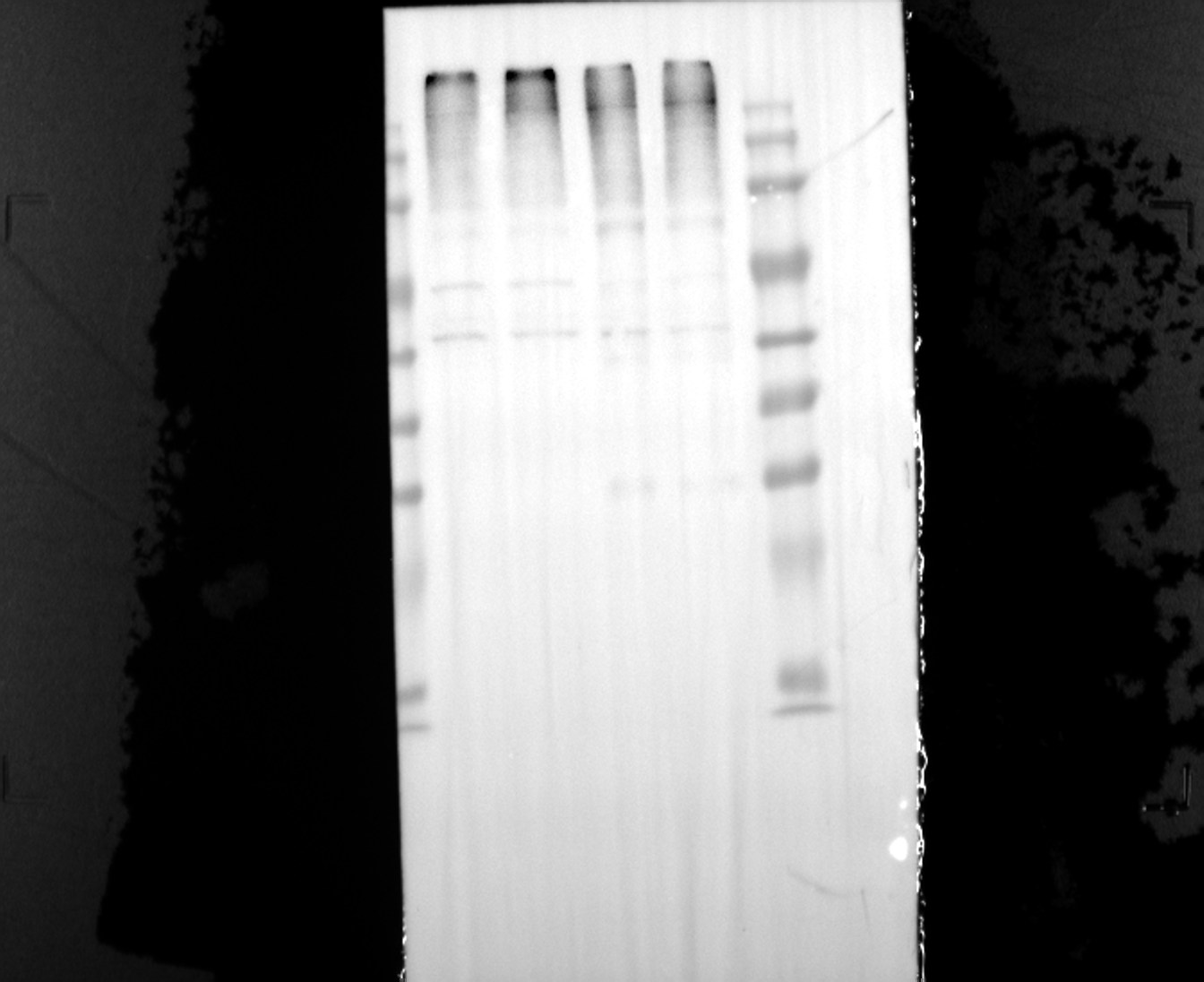

Supplement: Supplementary file 4 — Supplementary Material 4. [file 12964_2024_1770_MOESM4_ESM.zip › SENP3 TAM WB/WB-Figure4/B M0 M2 EndoIP/2023-02-18 ─┌╘┤IP shNC shSENP3 IRF4/INPUT SUMO23 0222/INPUT SUMO23 2S M 0222.Tif]

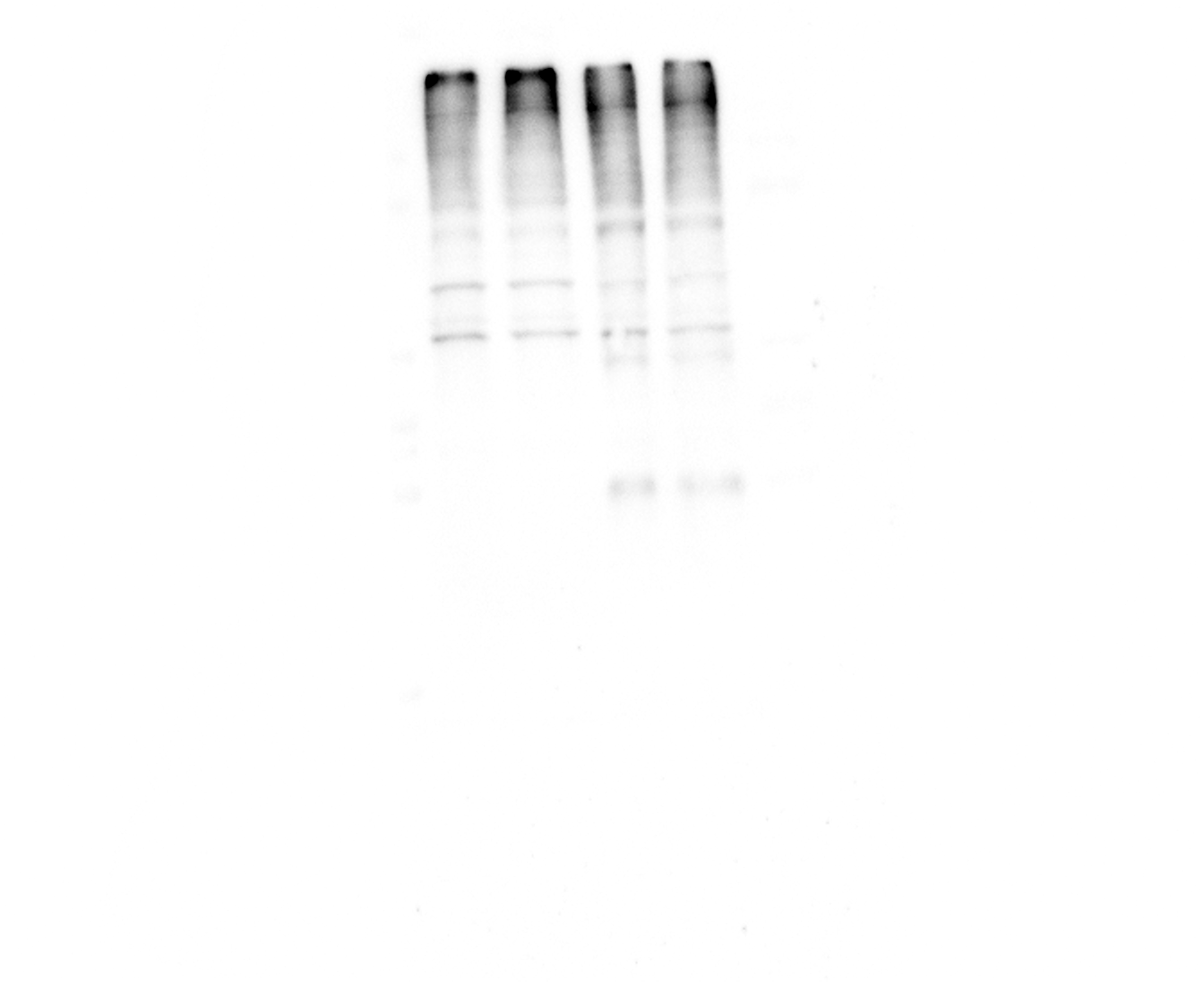

Supplement: Supplementary file 4 — Supplementary Material 4. [file 12964_2024_1770_MOESM4_ESM.zip › SENP3 TAM WB/WB-Figure4/B M0 M2 EndoIP/2023-02-18 ─┌╘┤IP shNC shSENP3 IRF4/INPUT SUMO23 0222/INPUT SUMO23 3S 0222.Tif]

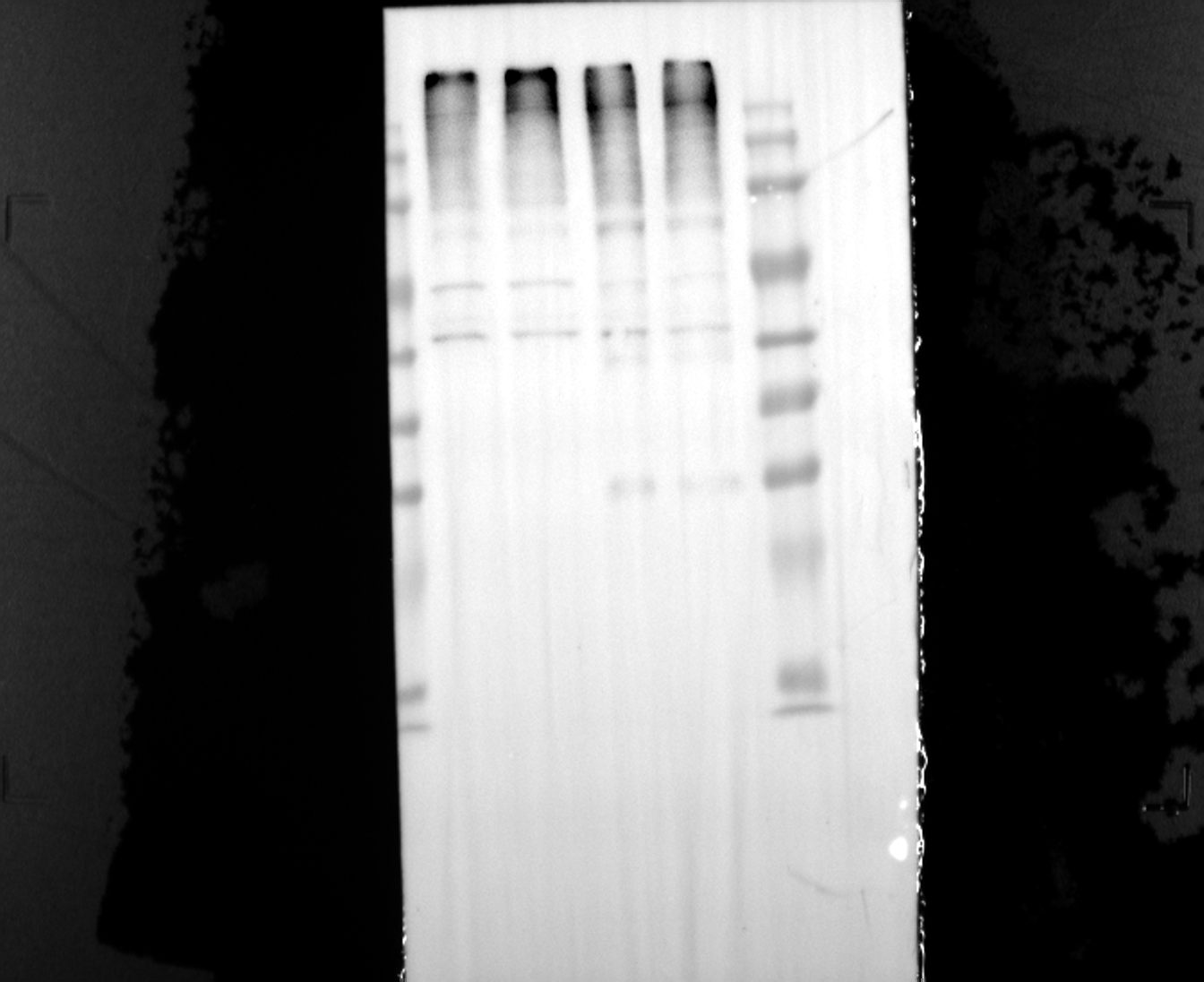

Supplement: Supplementary file 4 — Supplementary Material 4. [file 12964_2024_1770_MOESM4_ESM.zip › SENP3 TAM WB/WB-Figure4/B M0 M2 EndoIP/2023-02-18 ─┌╘┤IP shNC shSENP3 IRF4/INPUT SUMO23 0222/INPUT SUMO23 3S M 0222.Tif]

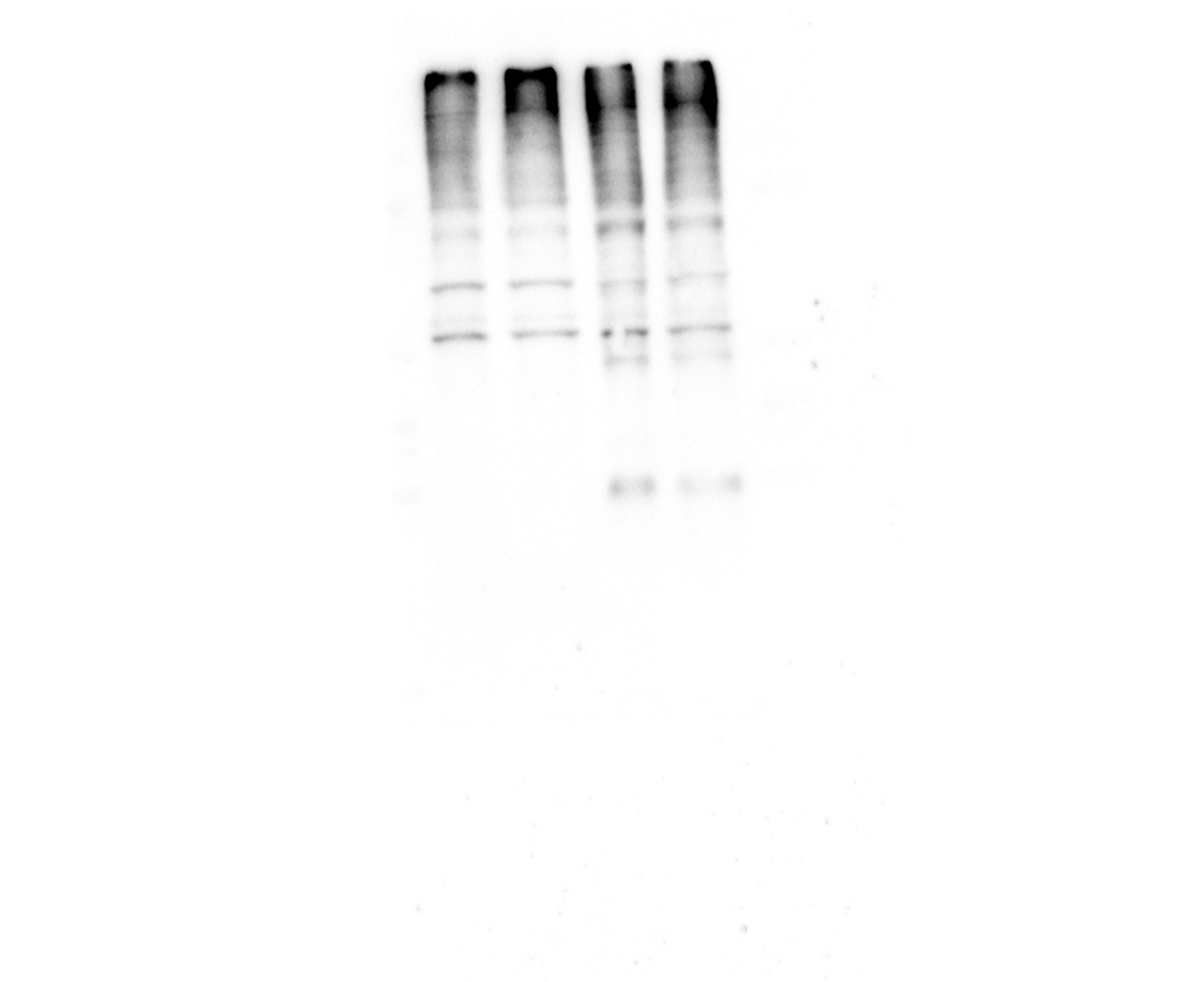

Supplement: Supplementary file 4 — Supplementary Material 4. [file 12964_2024_1770_MOESM4_ESM.zip › SENP3 TAM WB/WB-Figure4/B M0 M2 EndoIP/2023-02-18 ─┌╘┤IP shNC shSENP3 IRF4/INPUT SUMO23 0222/INPUT SUMO23 5S 0222.Tif]

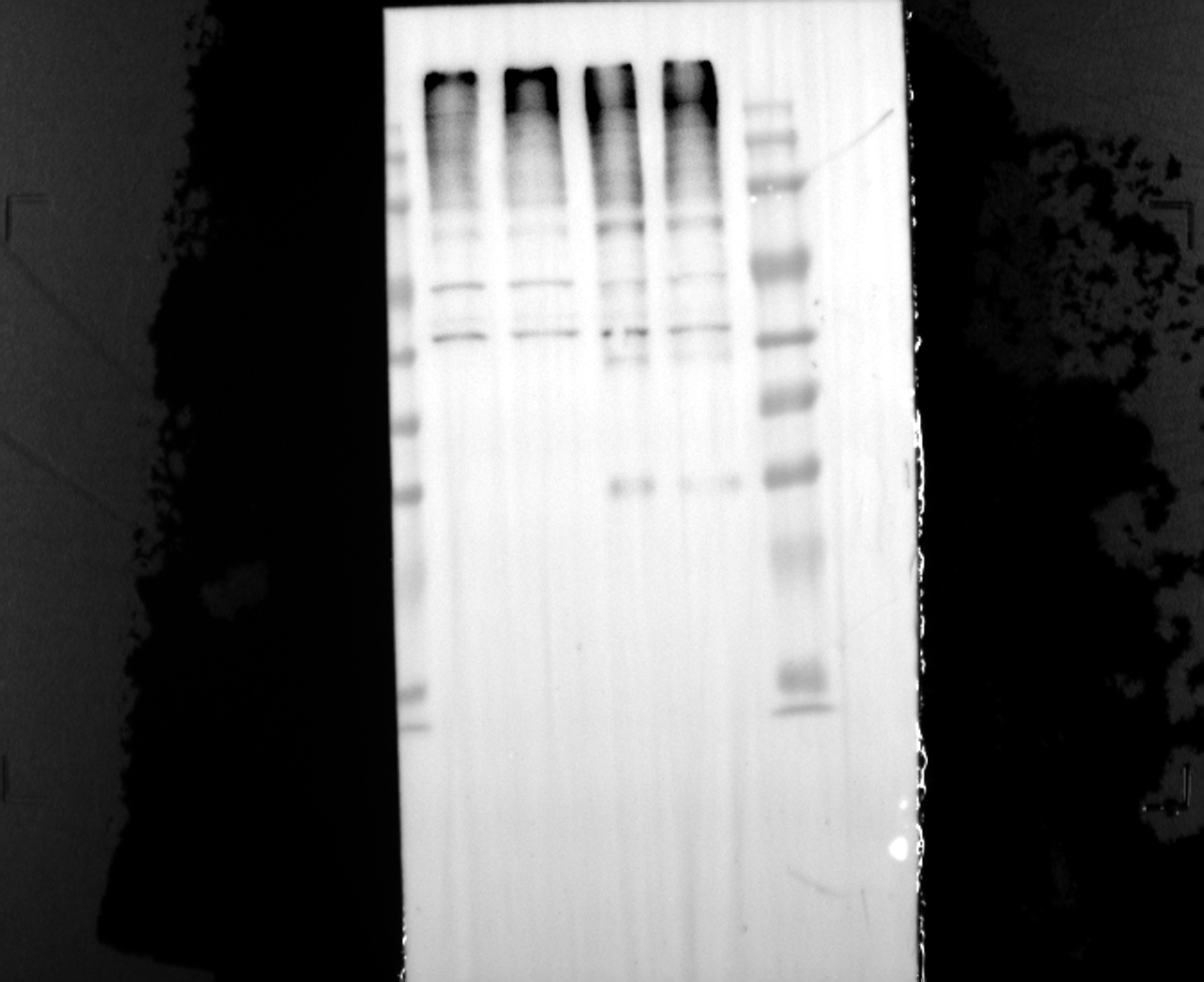

Supplement: Supplementary file 4 — Supplementary Material 4. [file 12964_2024_1770_MOESM4_ESM.zip › SENP3 TAM WB/WB-Figure4/B M0 M2 EndoIP/2023-02-18 ─┌╘┤IP shNC shSENP3 IRF4/INPUT SUMO23 0222/INPUT SUMO23 5S M 0222.Tif]

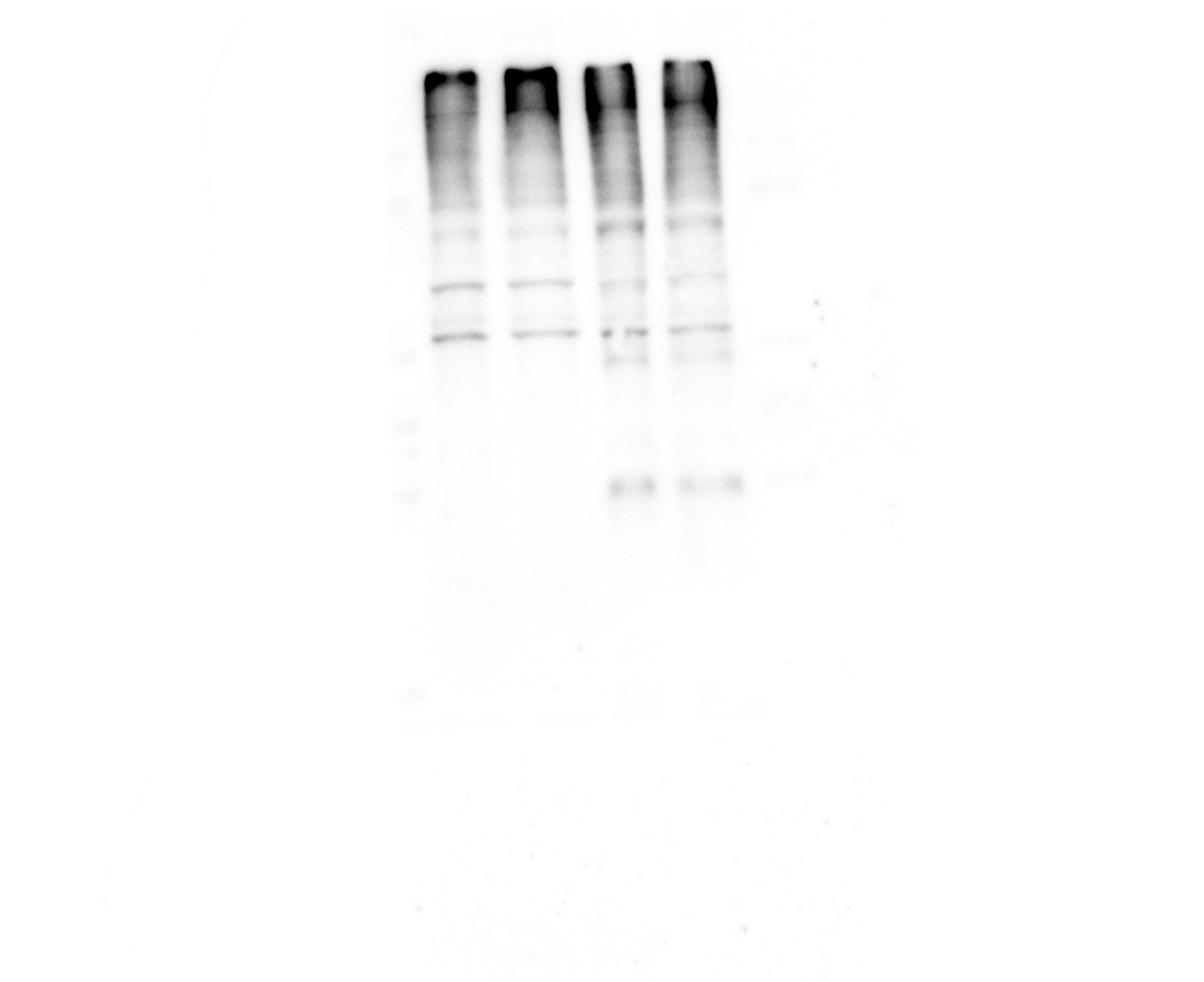

Supplement: Supplementary file 4 — Supplementary Material 4. [file 12964_2024_1770_MOESM4_ESM.zip › SENP3 TAM WB/WB-Figure4/B M0 M2 EndoIP/2023-02-18 ─┌╘┤IP shNC shSENP3 IRF4/INPUT SUMO23 0222/INPUT SUMO23 7S 0222.Tif]

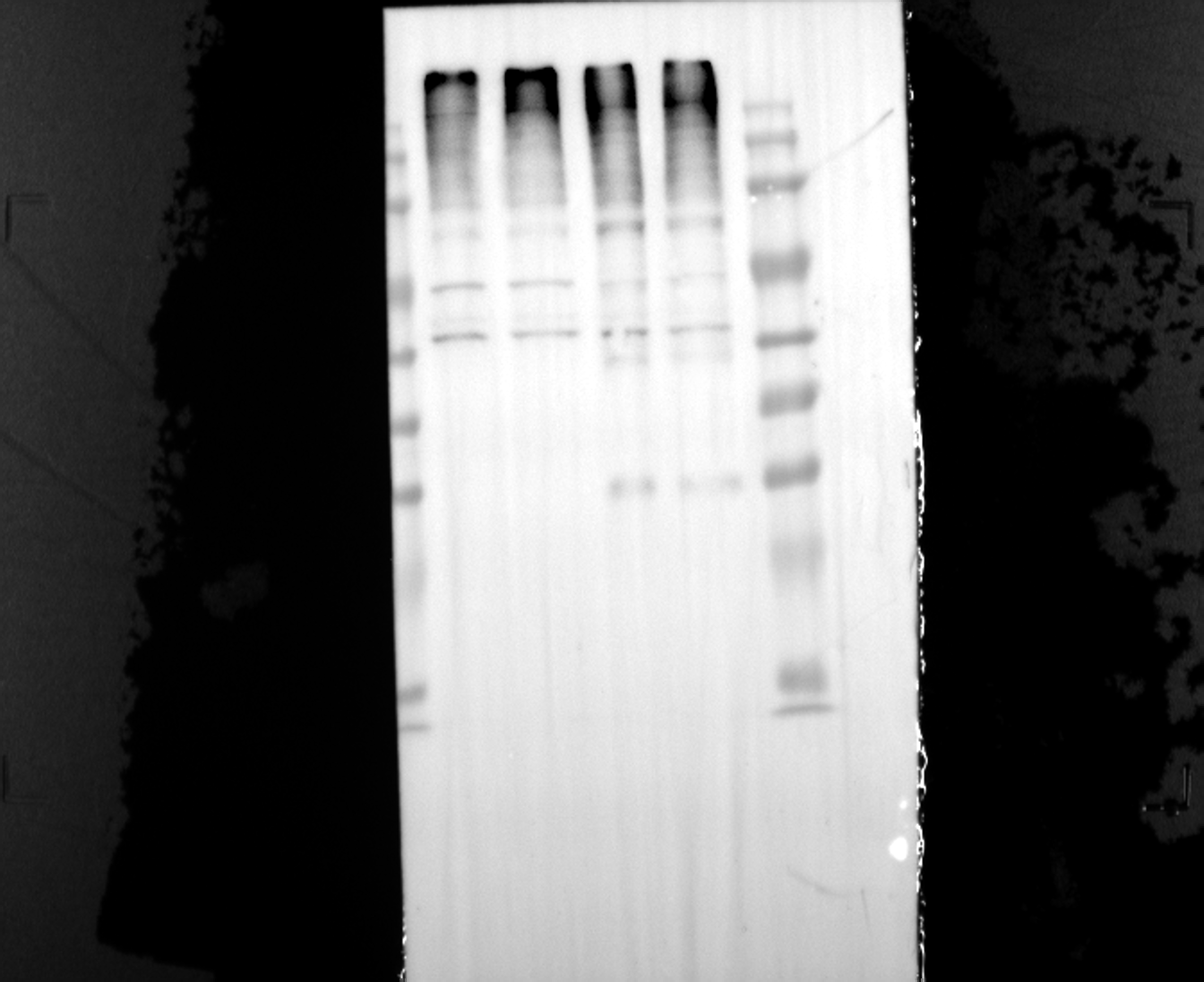

Supplement: Supplementary file 4 — Supplementary Material 4. [file 12964_2024_1770_MOESM4_ESM.zip › SENP3 TAM WB/WB-Figure4/B M0 M2 EndoIP/2023-02-18 ─┌╘┤IP shNC shSENP3 IRF4/INPUT SUMO23 0222/INPUT SUMO23 7S M 0222.Tif]

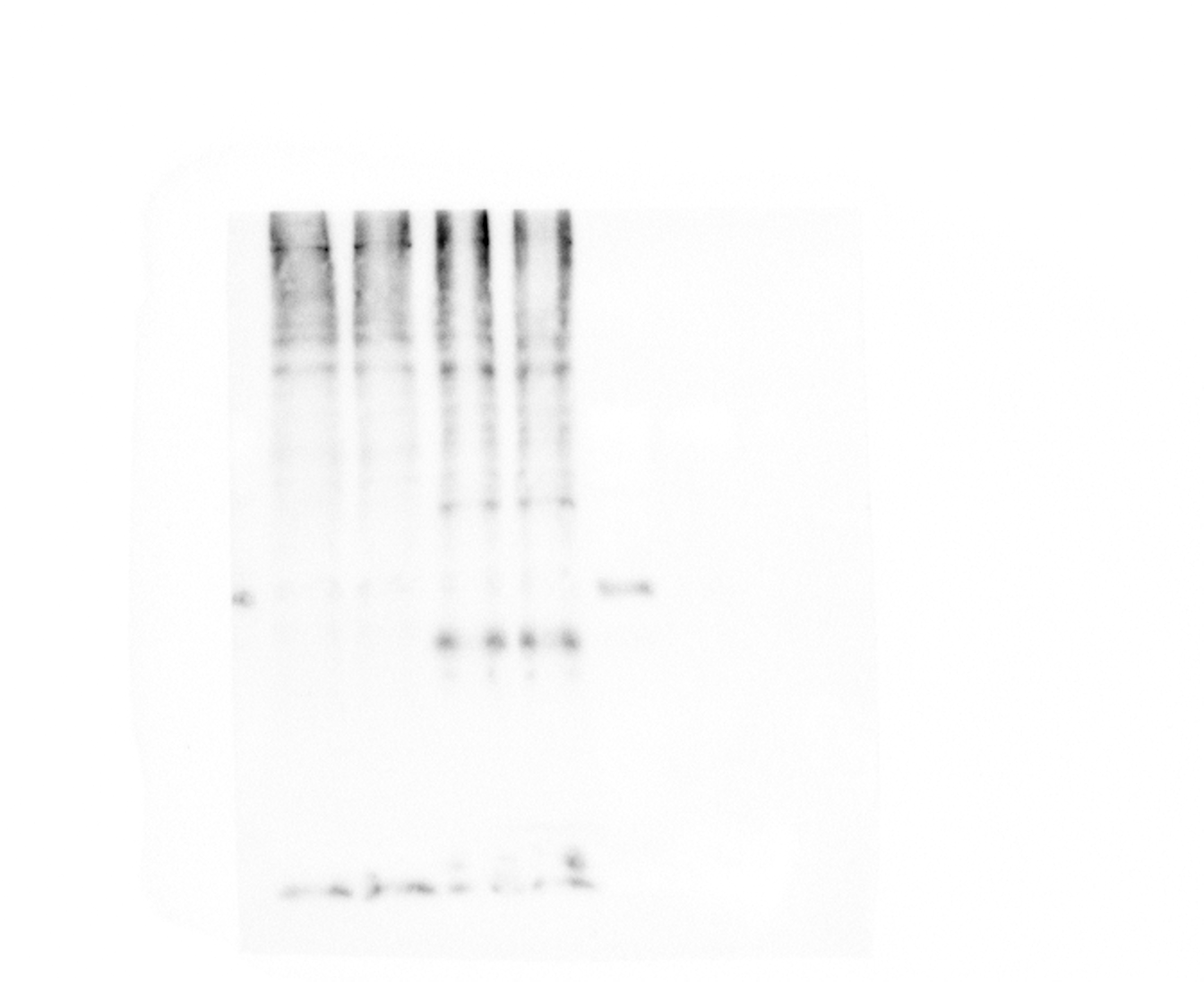

Supplement: Supplementary file 4 — Supplementary Material 4. [file 12964_2024_1770_MOESM4_ESM.zip › SENP3 TAM WB/WB-Figure4/B M0 M2 EndoIP/2023-02-18 ─┌╘┤IP shNC shSENP3 IRF4/INPUT SUMO23/INPUT SUMO23 3S 0218.Tif]

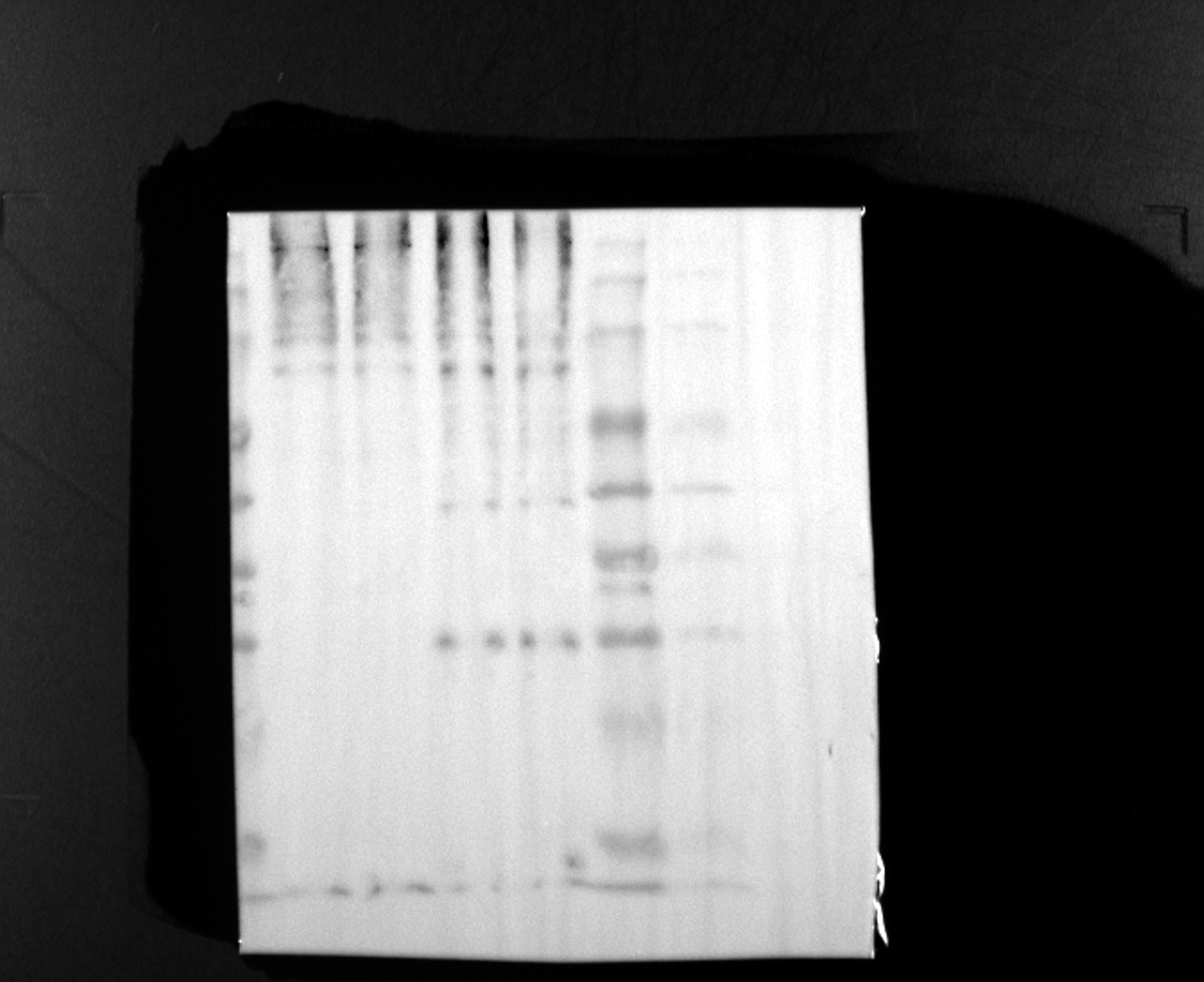

Supplement: Supplementary file 4 — Supplementary Material 4. [file 12964_2024_1770_MOESM4_ESM.zip › SENP3 TAM WB/WB-Figure4/B M0 M2 EndoIP/2023-02-18 ─┌╘┤IP shNC shSENP3 IRF4/INPUT SUMO23/INPUT SUMO23 3S M 0218.Tif]

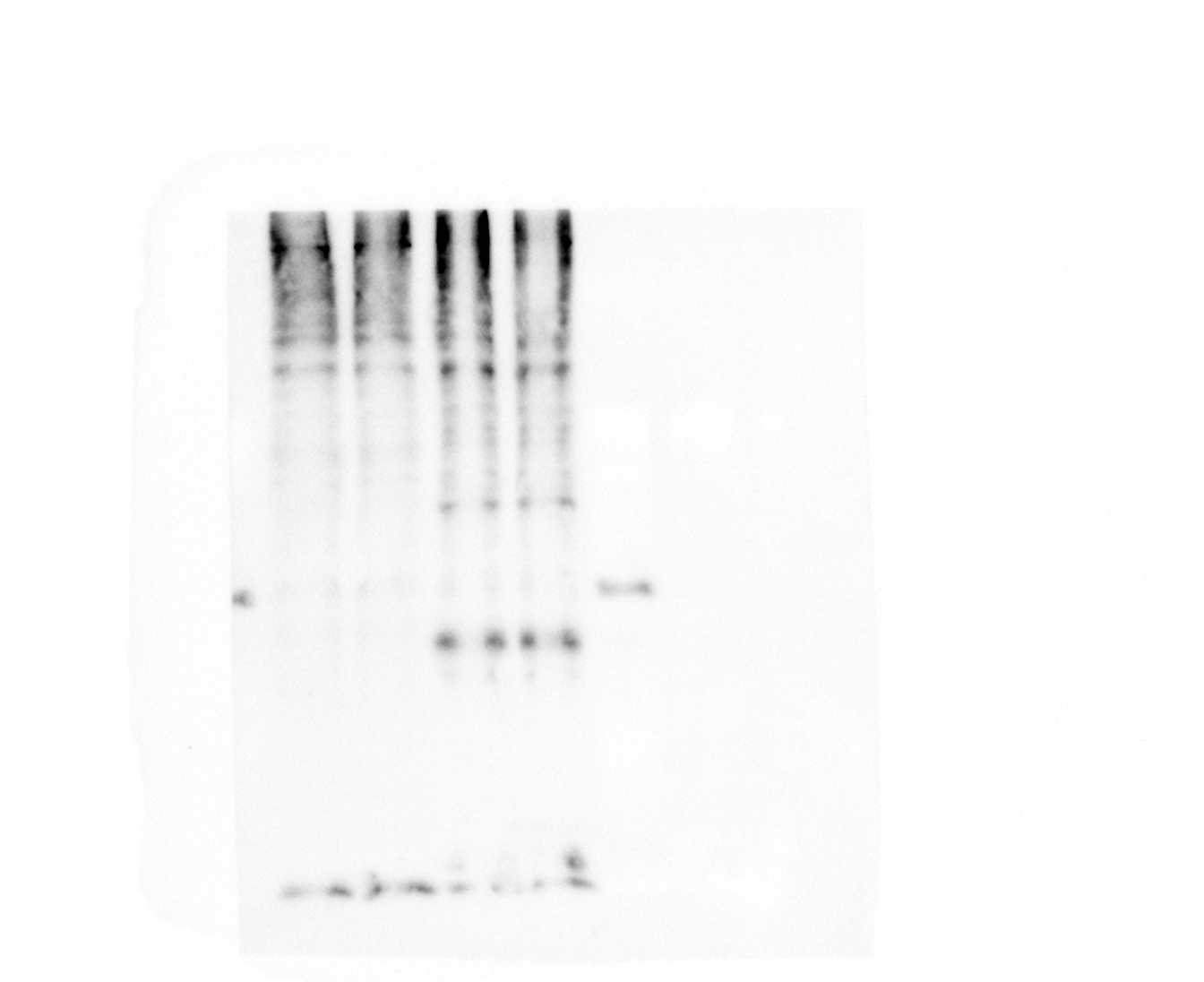

Supplement: Supplementary file 4 — Supplementary Material 4. [file 12964_2024_1770_MOESM4_ESM.zip › SENP3 TAM WB/WB-Figure4/B M0 M2 EndoIP/2023-02-18 ─┌╘┤IP shNC shSENP3 IRF4/INPUT SUMO23/INPUT SUMO23 5S 0218.Tif]

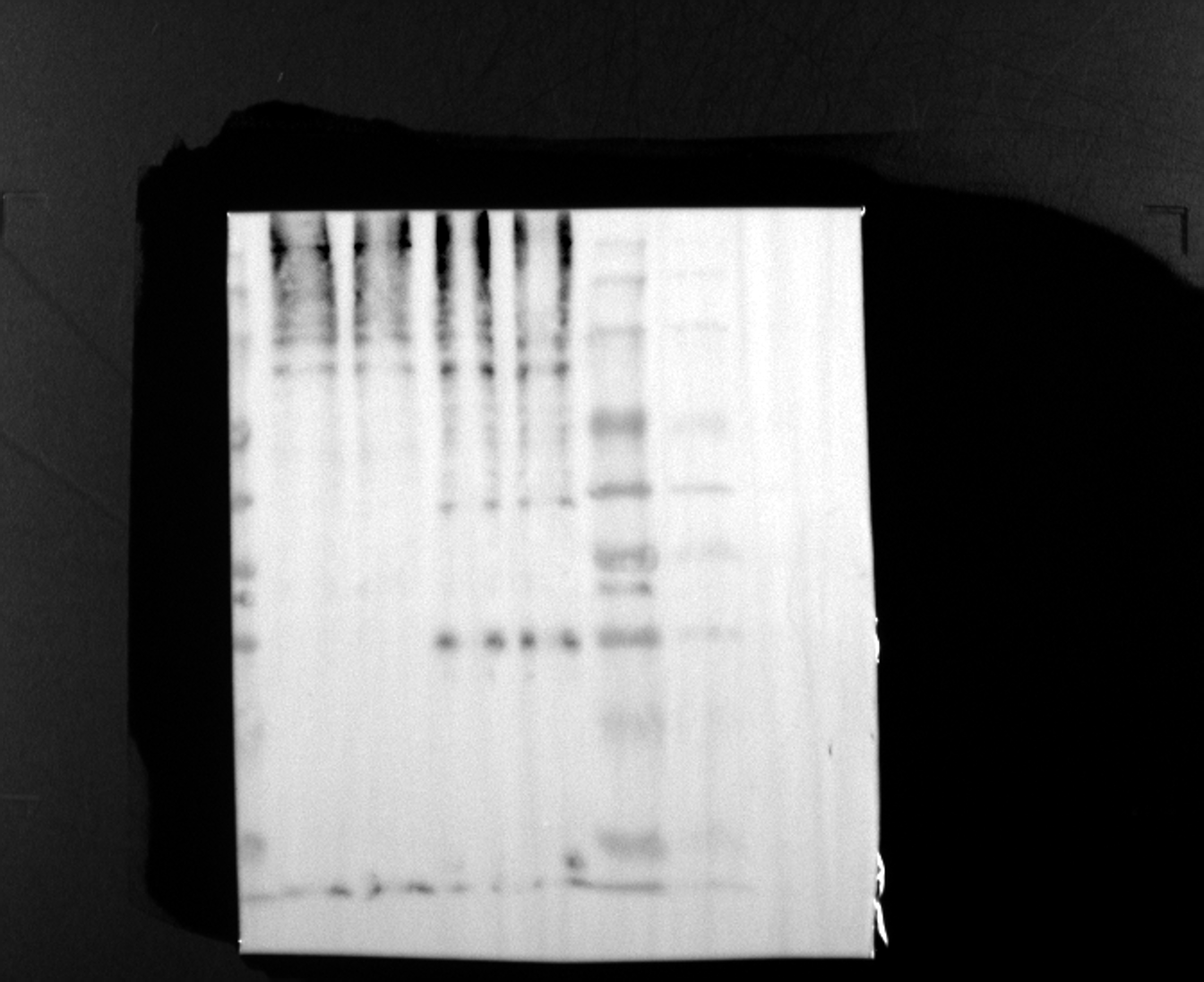

Supplement: Supplementary file 4 — Supplementary Material 4. [file 12964_2024_1770_MOESM4_ESM.zip › SENP3 TAM WB/WB-Figure4/B M0 M2 EndoIP/2023-02-18 ─┌╘┤IP shNC shSENP3 IRF4/INPUT SUMO23/INPUT SUMO23 5S M 0218.Tif]

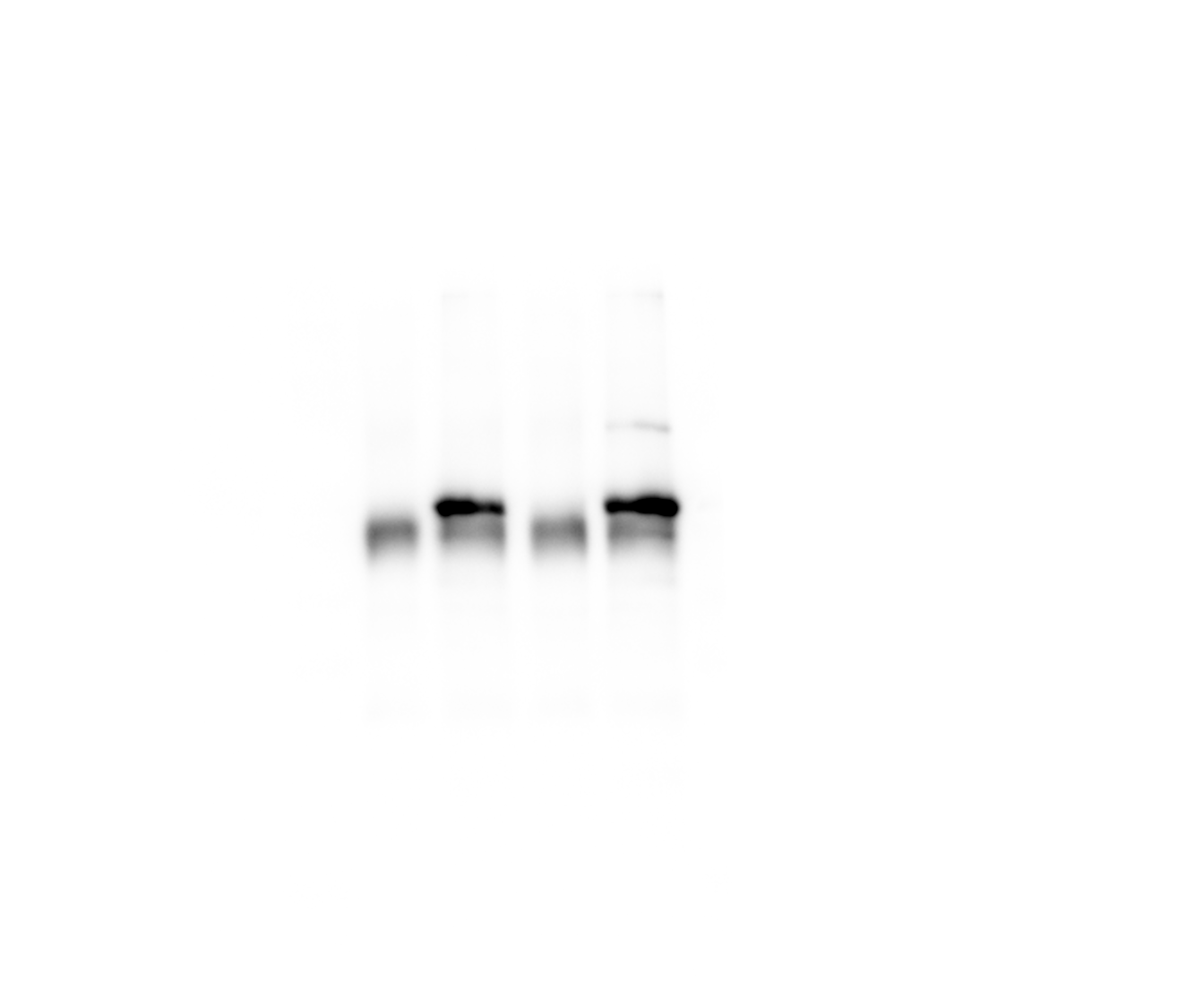

Supplement: Supplementary file 4 — Supplementary Material 4. [file 12964_2024_1770_MOESM4_ESM.zip › SENP3 TAM WB/WB-Figure4/B M0 M2 EndoIP/2023-02-18 ─┌╘┤IP shNC shSENP3 IRF4/IP IRF4 0221/IP IRF4 0.4S 0221.Tif]

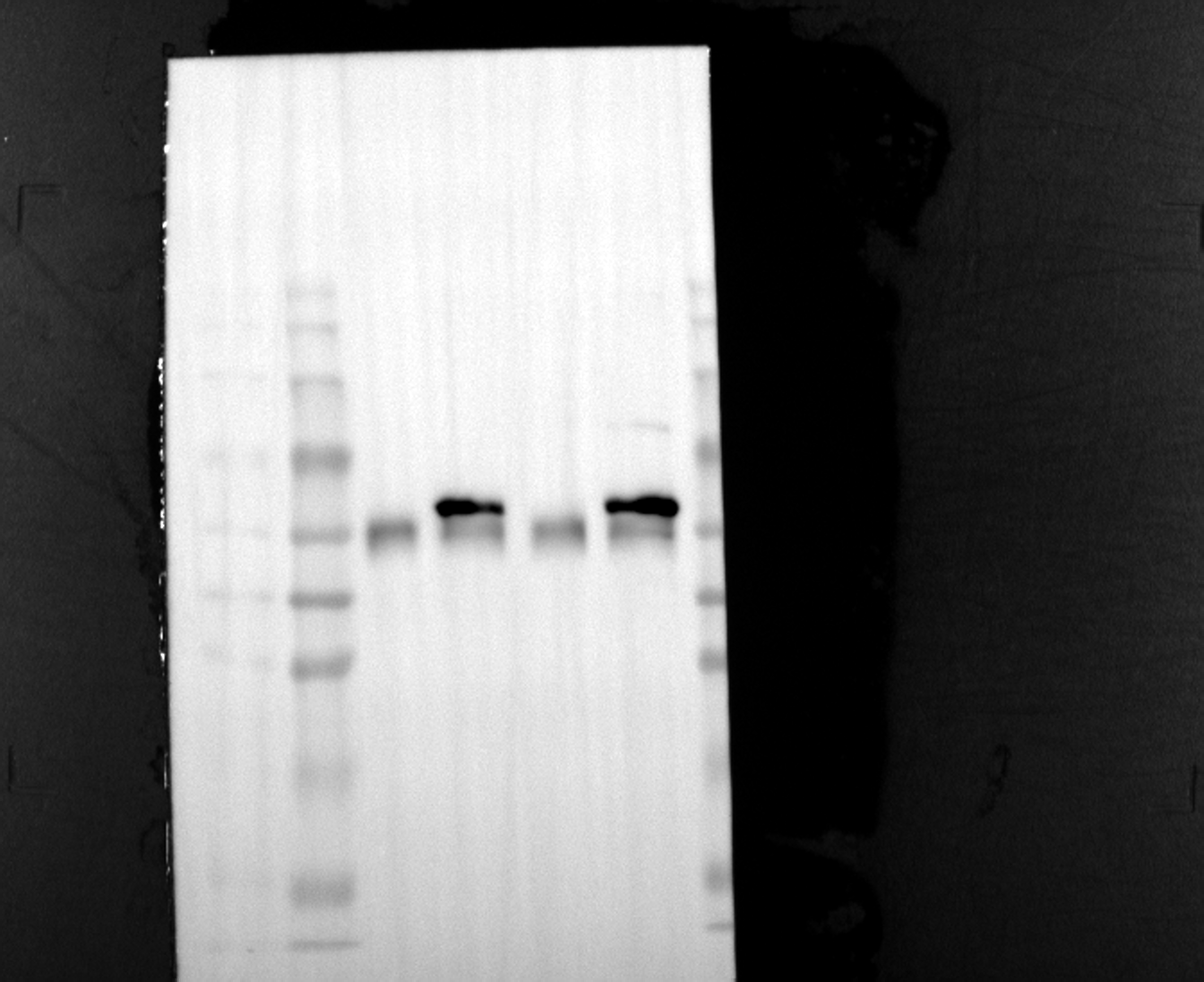

Supplement: Supplementary file 4 — Supplementary Material 4. [file 12964_2024_1770_MOESM4_ESM.zip › SENP3 TAM WB/WB-Figure4/B M0 M2 EndoIP/2023-02-18 ─┌╘┤IP shNC shSENP3 IRF4/IP IRF4 0221/IP IRF4 0.4S M 0221.Tif]

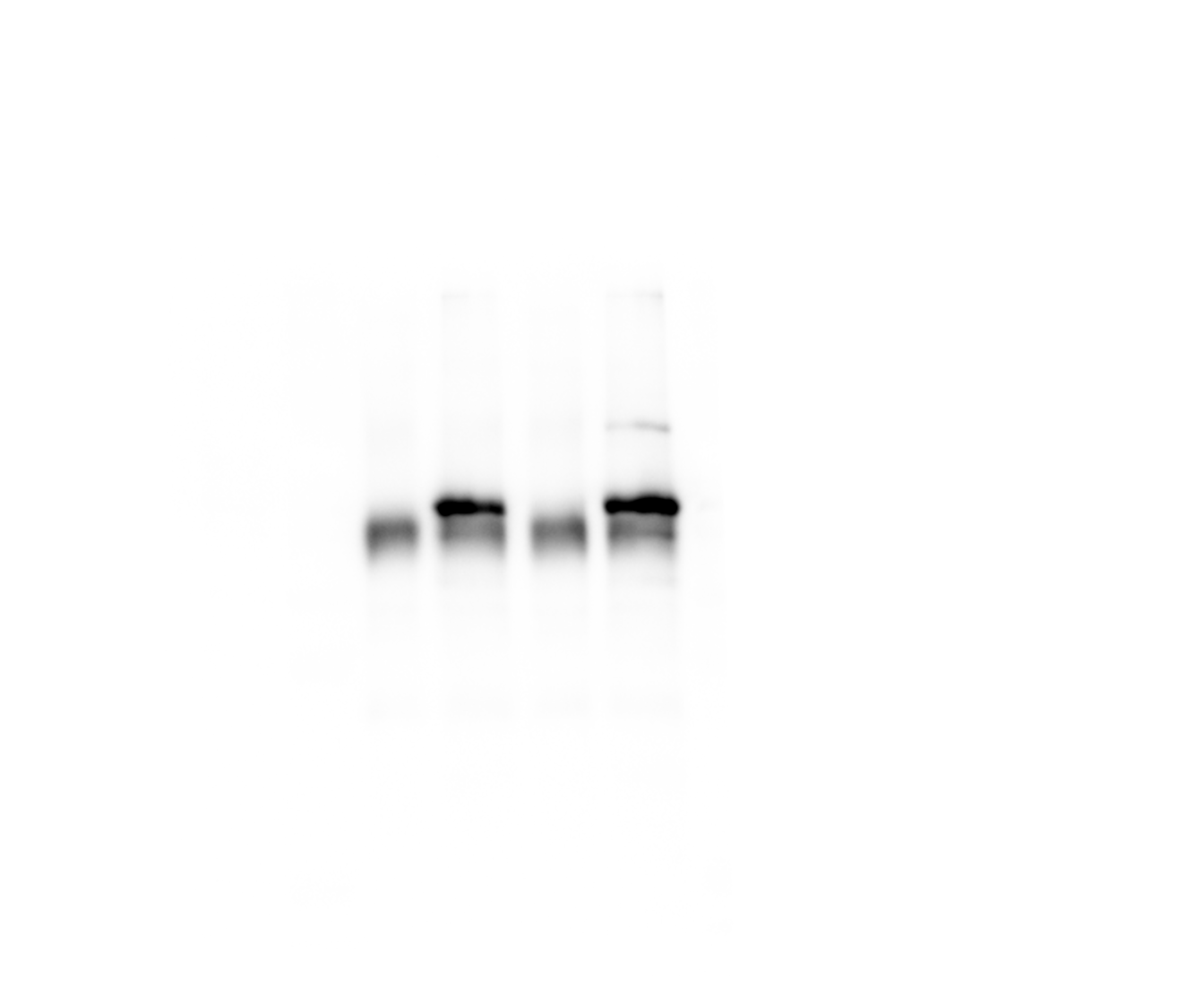

Supplement: Supplementary file 4 — Supplementary Material 4. [file 12964_2024_1770_MOESM4_ESM.zip › SENP3 TAM WB/WB-Figure4/B M0 M2 EndoIP/2023-02-18 ─┌╘┤IP shNC shSENP3 IRF4/IP IRF4 0221/IP IRF4 0.5S 0221.Tif]

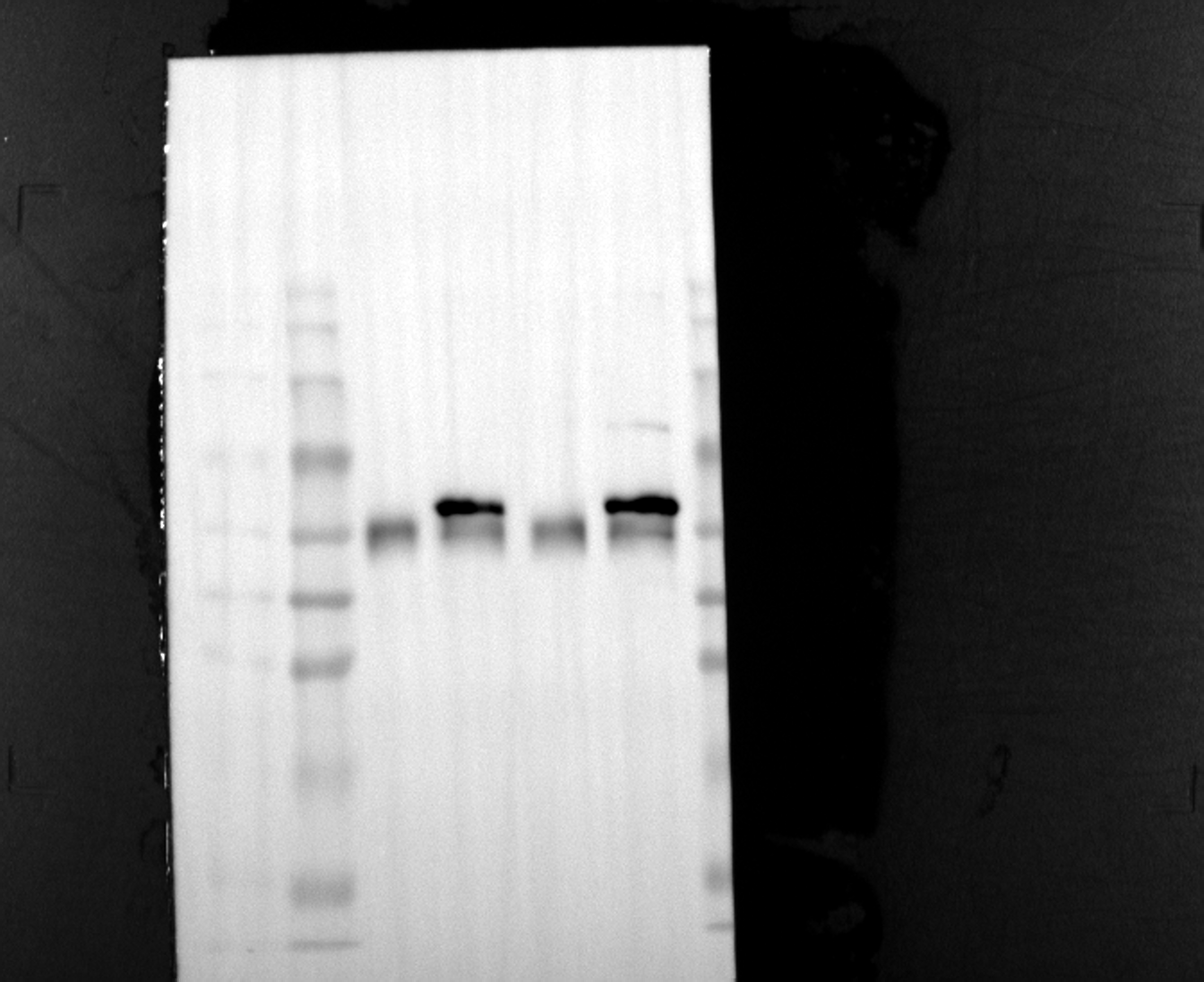

Supplement: Supplementary file 4 — Supplementary Material 4. [file 12964_2024_1770_MOESM4_ESM.zip › SENP3 TAM WB/WB-Figure4/B M0 M2 EndoIP/2023-02-18 ─┌╘┤IP shNC shSENP3 IRF4/IP IRF4 0221/IP IRF4 0.5S M 0221.Tif]

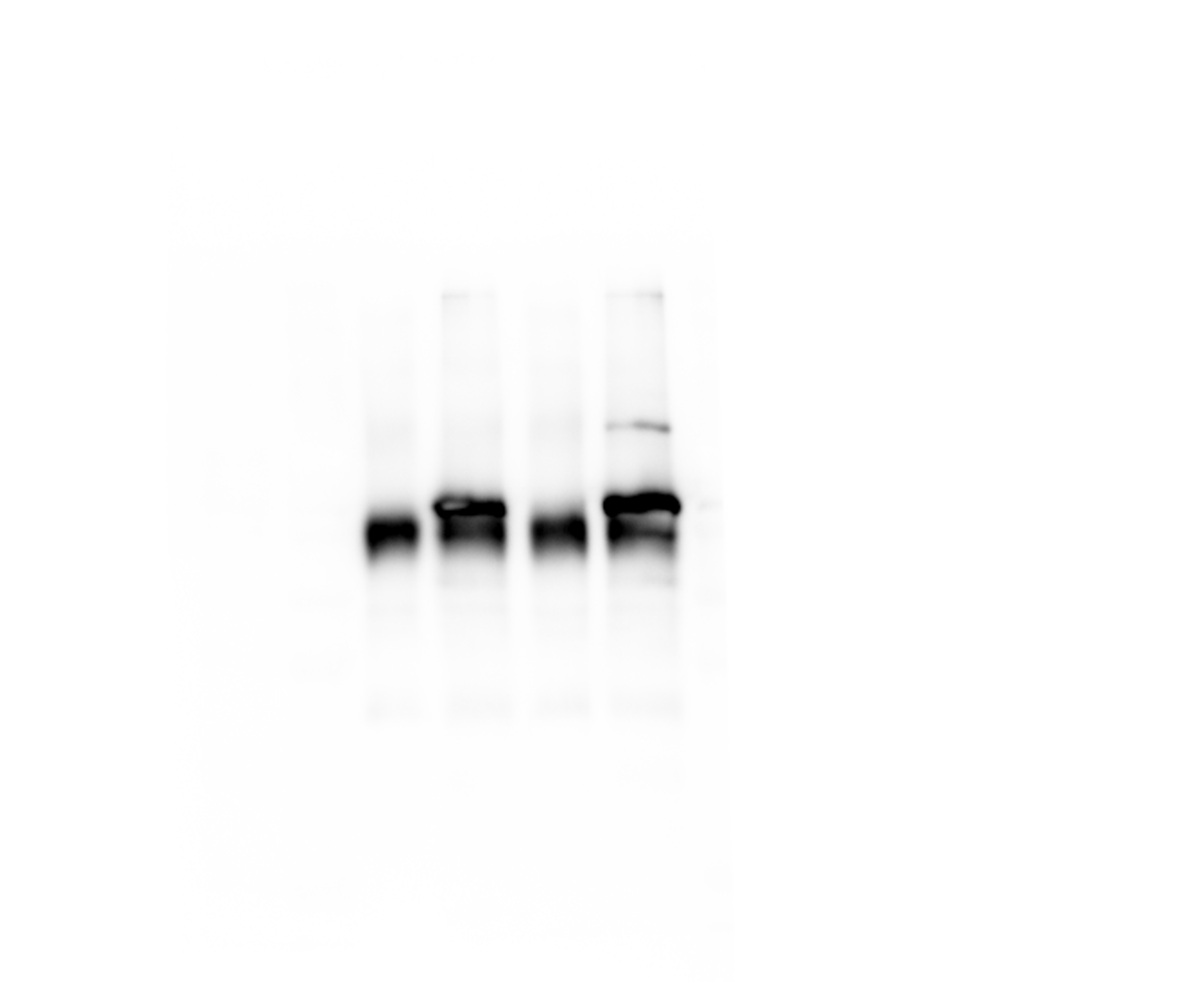

Supplement: Supplementary file 4 — Supplementary Material 4. [file 12964_2024_1770_MOESM4_ESM.zip › SENP3 TAM WB/WB-Figure4/B M0 M2 EndoIP/2023-02-18 ─┌╘┤IP shNC shSENP3 IRF4/IP IRF4 0221/IP IRF4 1.5S 0221.Tif]

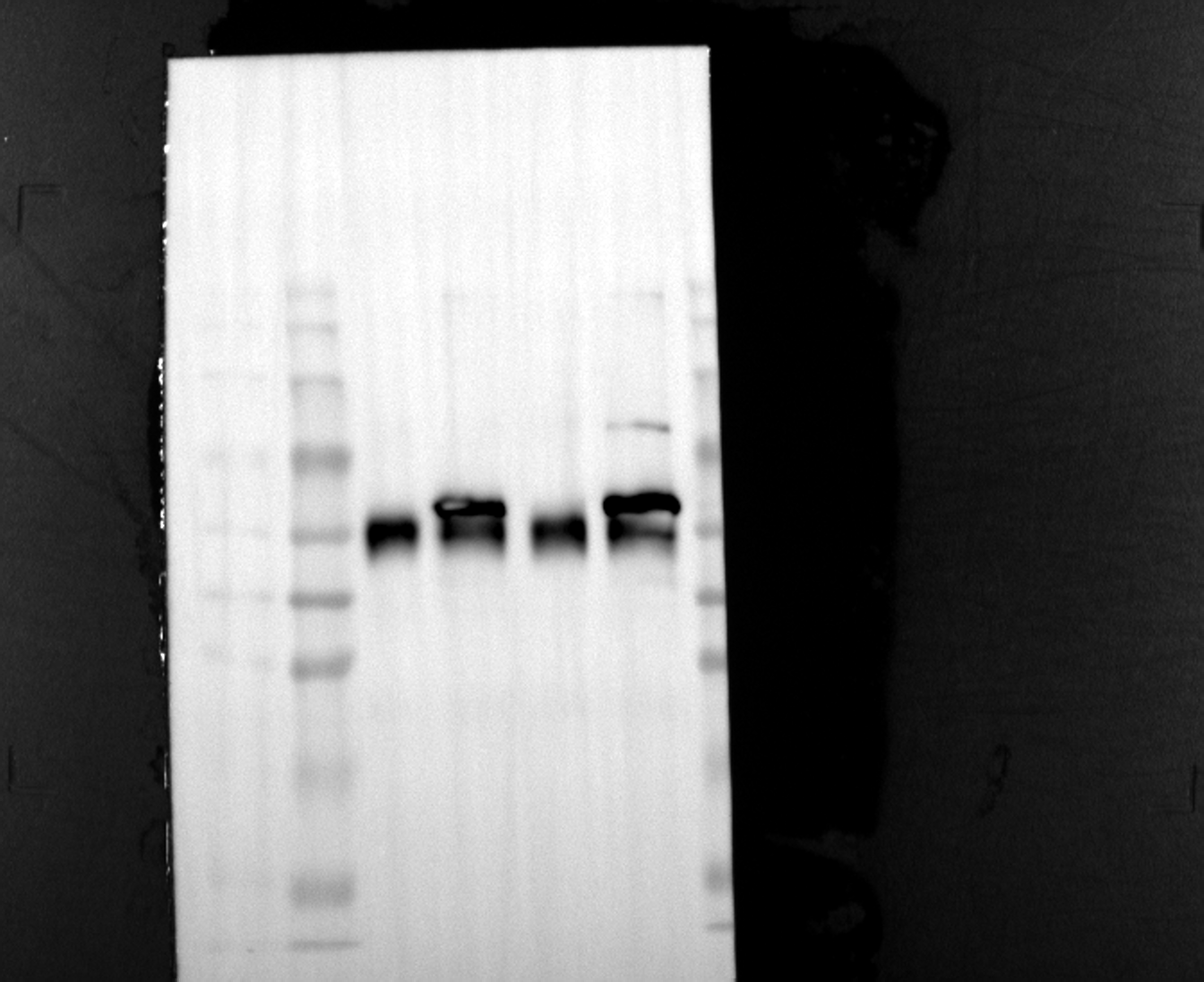

Supplement: Supplementary file 4 — Supplementary Material 4. [file 12964_2024_1770_MOESM4_ESM.zip › SENP3 TAM WB/WB-Figure4/B M0 M2 EndoIP/2023-02-18 ─┌╘┤IP shNC shSENP3 IRF4/IP IRF4 0221/IP IRF4 1.5S M 0221.Tif]

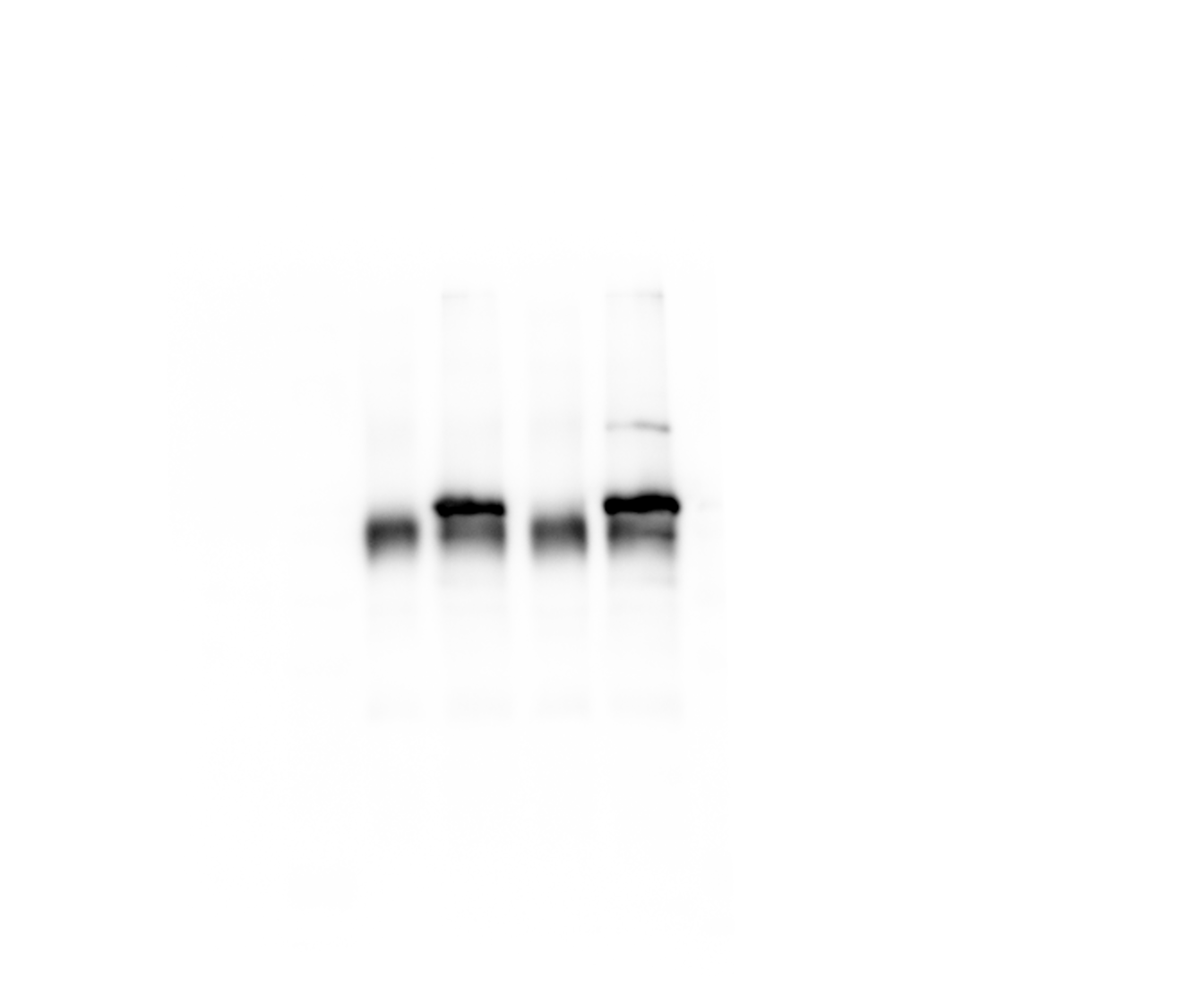

Supplement: Supplementary file 4 — Supplementary Material 4. [file 12964_2024_1770_MOESM4_ESM.zip › SENP3 TAM WB/WB-Figure4/B M0 M2 EndoIP/2023-02-18 ─┌╘┤IP shNC shSENP3 IRF4/IP IRF4 0221/IP IRF4 1S 0221.Tif]

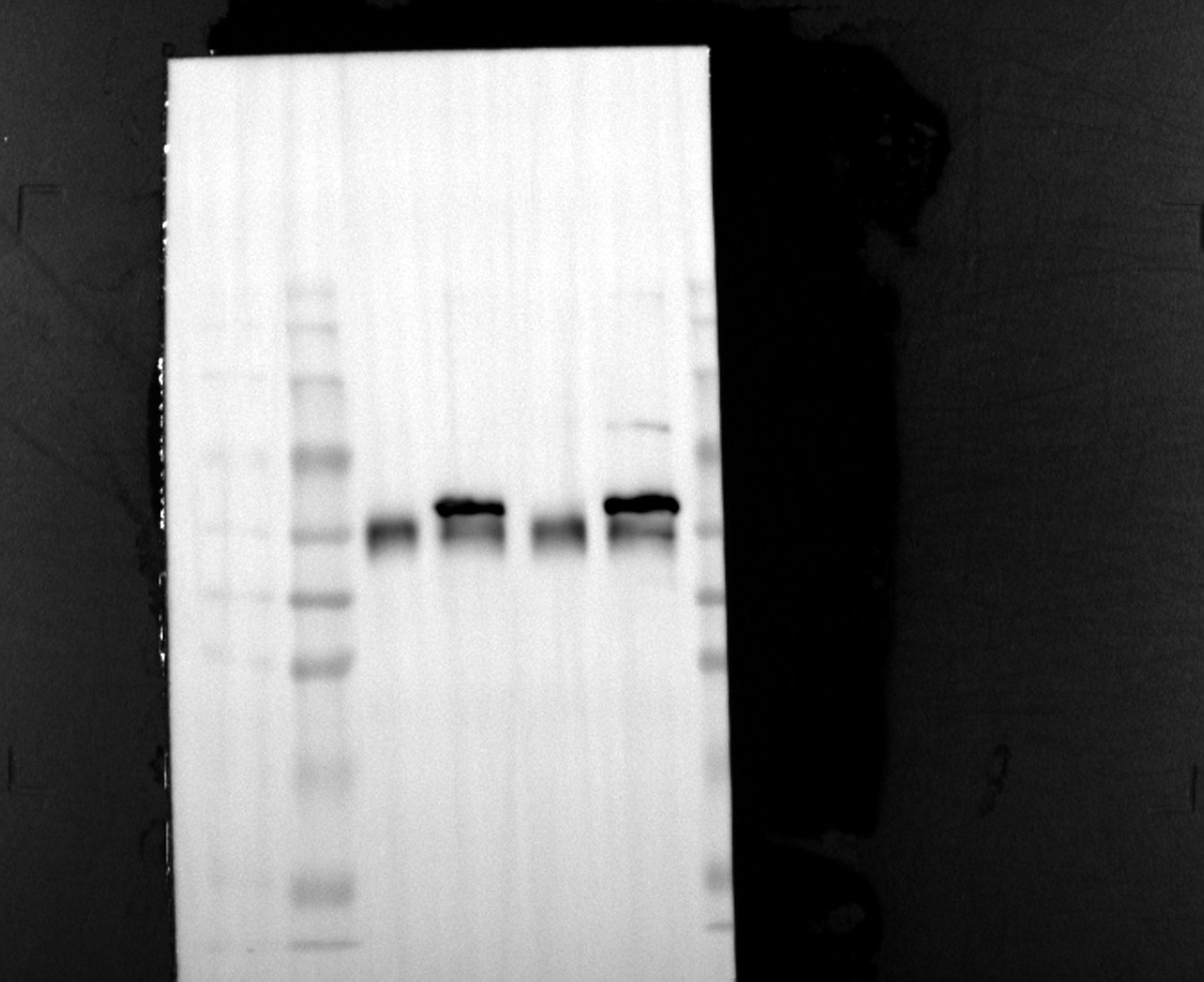

Supplement: Supplementary file 4 — Supplementary Material 4. [file 12964_2024_1770_MOESM4_ESM.zip › SENP3 TAM WB/WB-Figure4/B M0 M2 EndoIP/2023-02-18 ─┌╘┤IP shNC shSENP3 IRF4/IP IRF4 0221/IP IRF4 1S M 0221.Tif]

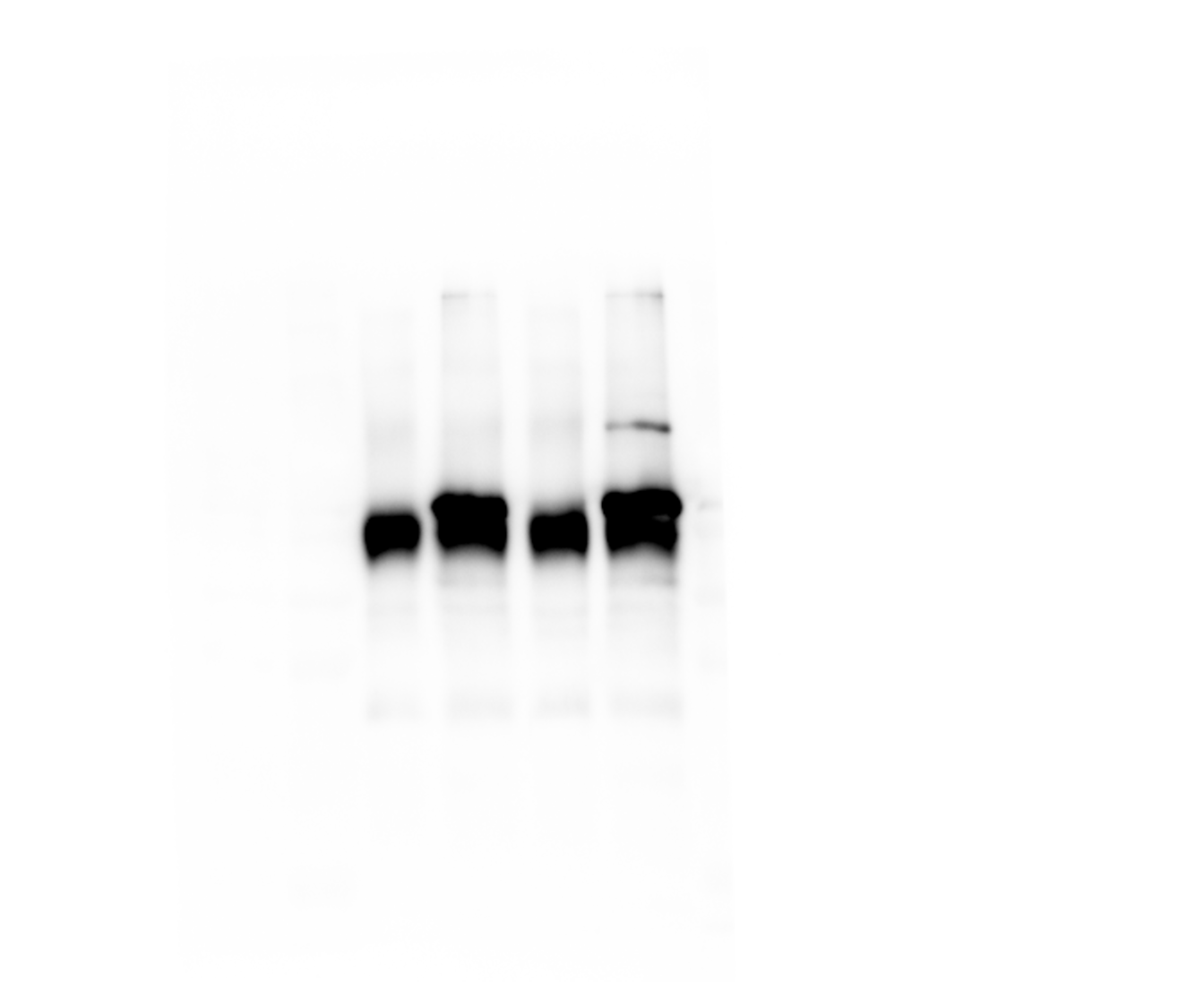

Supplement: Supplementary file 4 — Supplementary Material 4. [file 12964_2024_1770_MOESM4_ESM.zip › SENP3 TAM WB/WB-Figure4/B M0 M2 EndoIP/2023-02-18 ─┌╘┤IP shNC shSENP3 IRF4/IP IRF4 0221/IP IRF4 2.5S 0221.Tif]

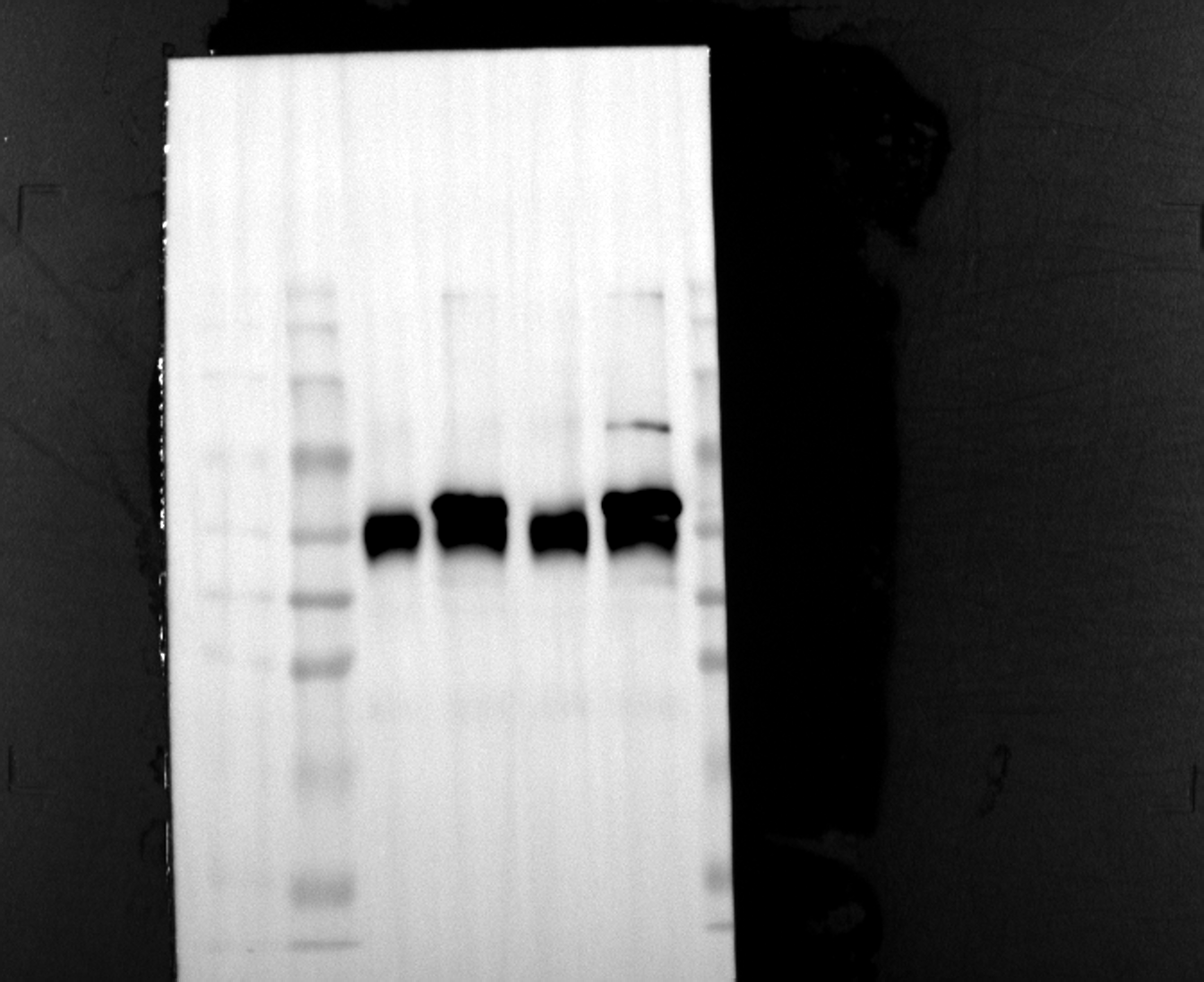

Supplement: Supplementary file 4 — Supplementary Material 4. [file 12964_2024_1770_MOESM4_ESM.zip › SENP3 TAM WB/WB-Figure4/B M0 M2 EndoIP/2023-02-18 ─┌╘┤IP shNC shSENP3 IRF4/IP IRF4 0221/IP IRF4 2.5S M 0221.Tif]

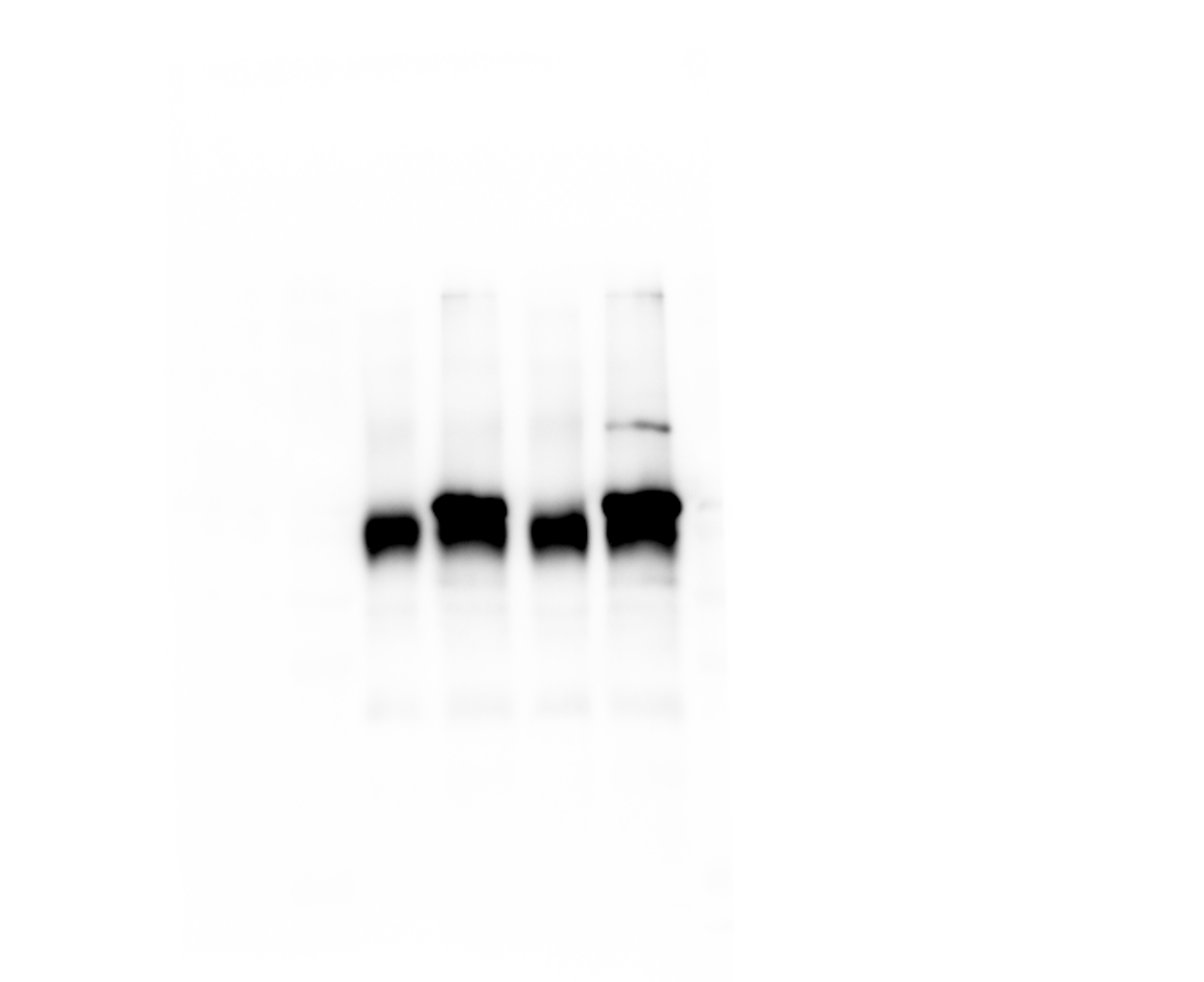

Supplement: Supplementary file 4 — Supplementary Material 4. [file 12964_2024_1770_MOESM4_ESM.zip › SENP3 TAM WB/WB-Figure4/B M0 M2 EndoIP/2023-02-18 ─┌╘┤IP shNC shSENP3 IRF4/IP IRF4 0221/IP IRF4 2S 0221.Tif]

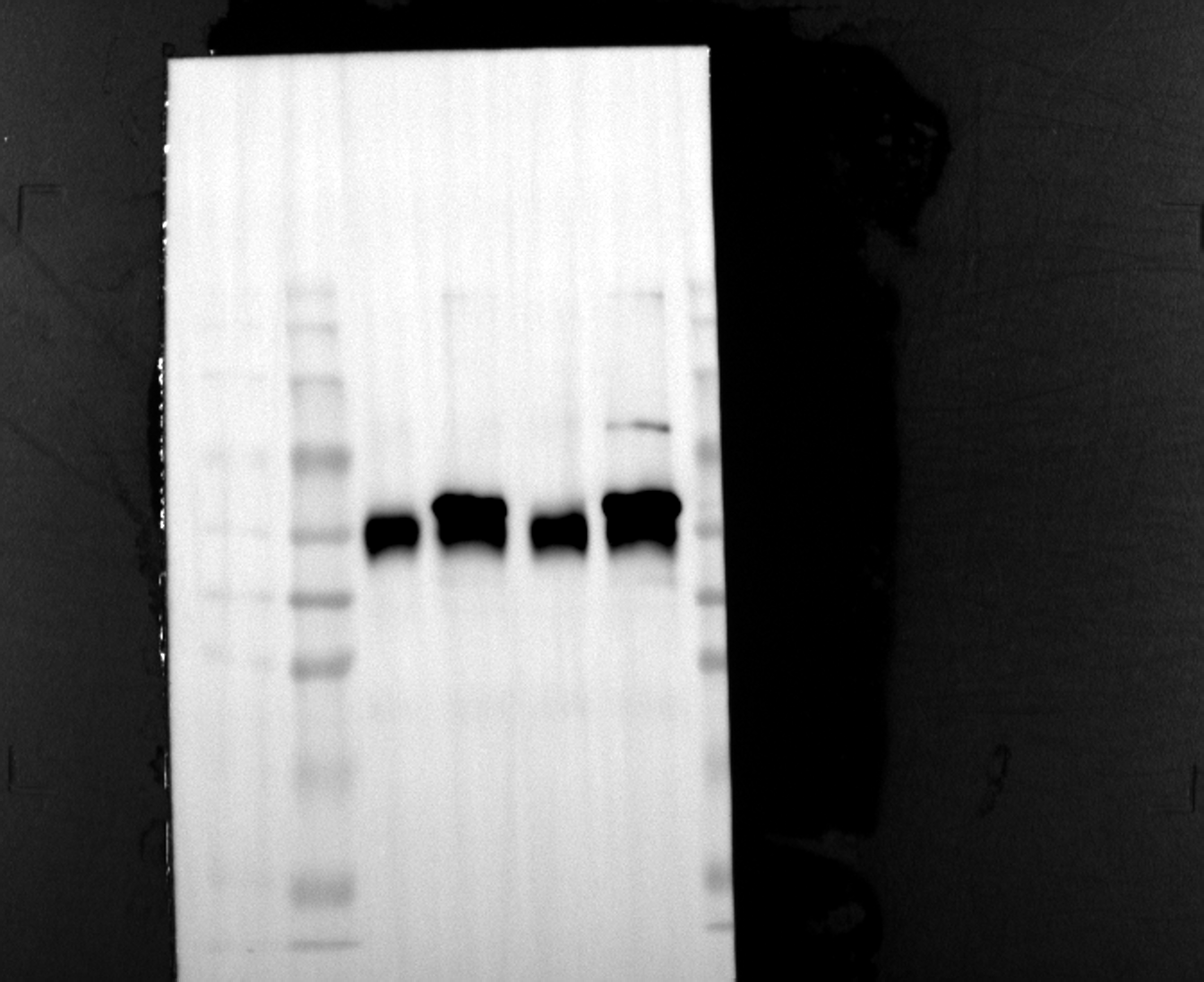

Supplement: Supplementary file 4 — Supplementary Material 4. [file 12964_2024_1770_MOESM4_ESM.zip › SENP3 TAM WB/WB-Figure4/B M0 M2 EndoIP/2023-02-18 ─┌╘┤IP shNC shSENP3 IRF4/IP IRF4 0221/IP IRF4 2S M 0221.Tif]

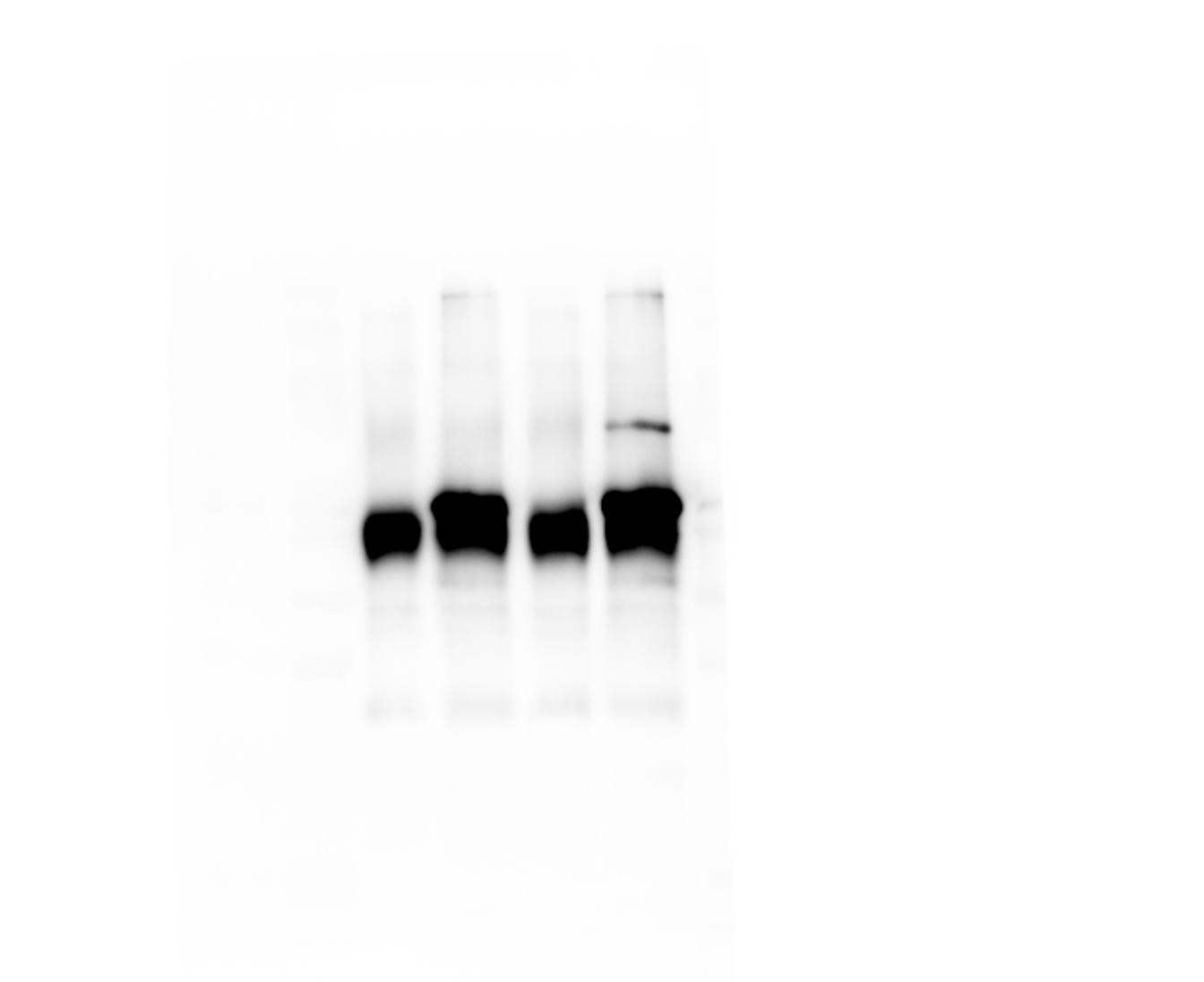

Supplement: Supplementary file 4 — Supplementary Material 4. [file 12964_2024_1770_MOESM4_ESM.zip › SENP3 TAM WB/WB-Figure4/B M0 M2 EndoIP/2023-02-18 ─┌╘┤IP shNC shSENP3 IRF4/IP IRF4 0221/IP IRF4 3S 0221.Tif]

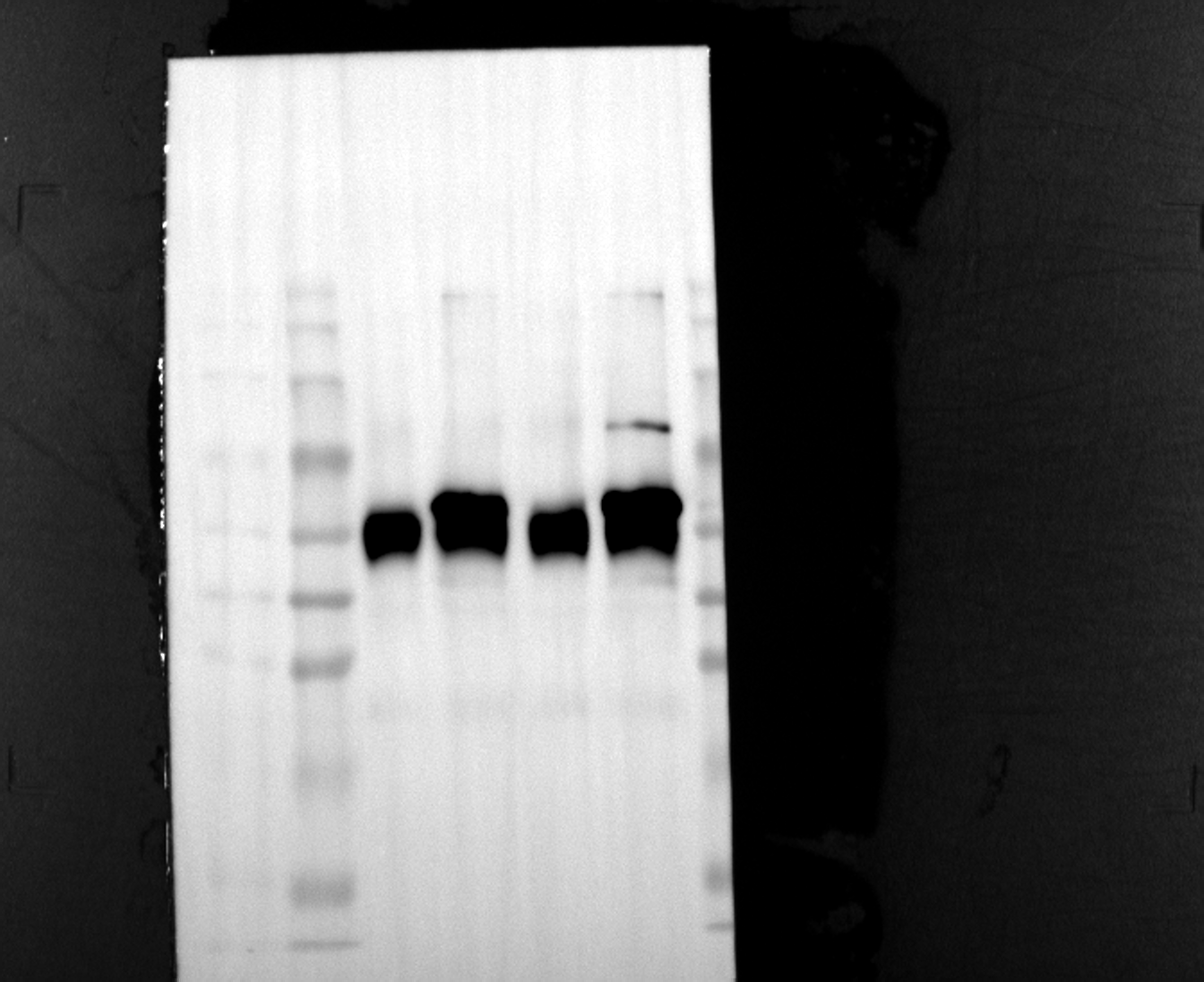

Supplement: Supplementary file 4 — Supplementary Material 4. [file 12964_2024_1770_MOESM4_ESM.zip › SENP3 TAM WB/WB-Figure4/B M0 M2 EndoIP/2023-02-18 ─┌╘┤IP shNC shSENP3 IRF4/IP IRF4 0221/IP IRF4 3S M 0221.Tif]

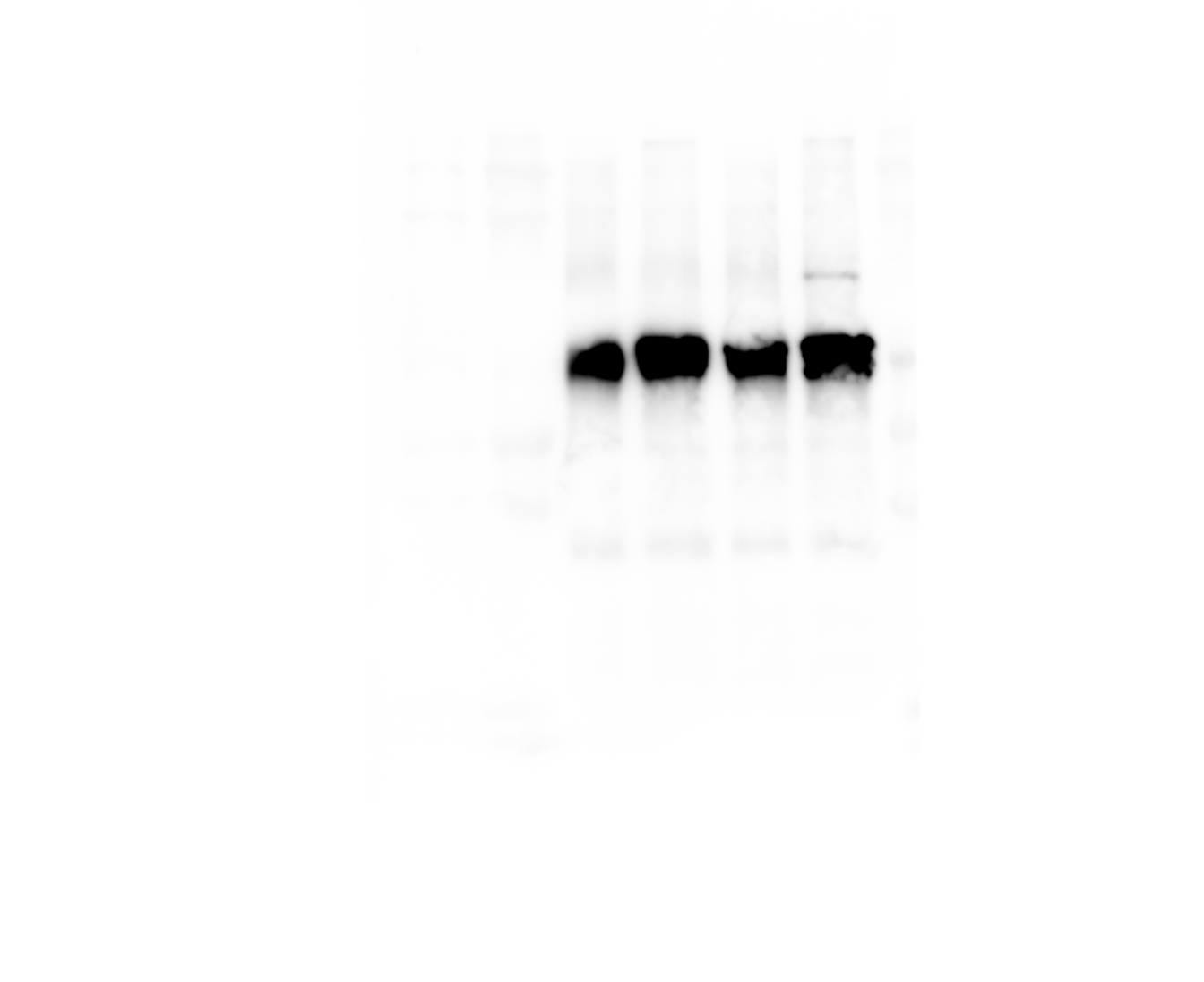

Supplement: Supplementary file 4 — Supplementary Material 4. [file 12964_2024_1770_MOESM4_ESM.zip › SENP3 TAM WB/WB-Figure4/B M0 M2 EndoIP/2023-02-18 ─┌╘┤IP shNC shSENP3 IRF4/IP IRF4 0222/IP IRF4 10S 0222.Tif]

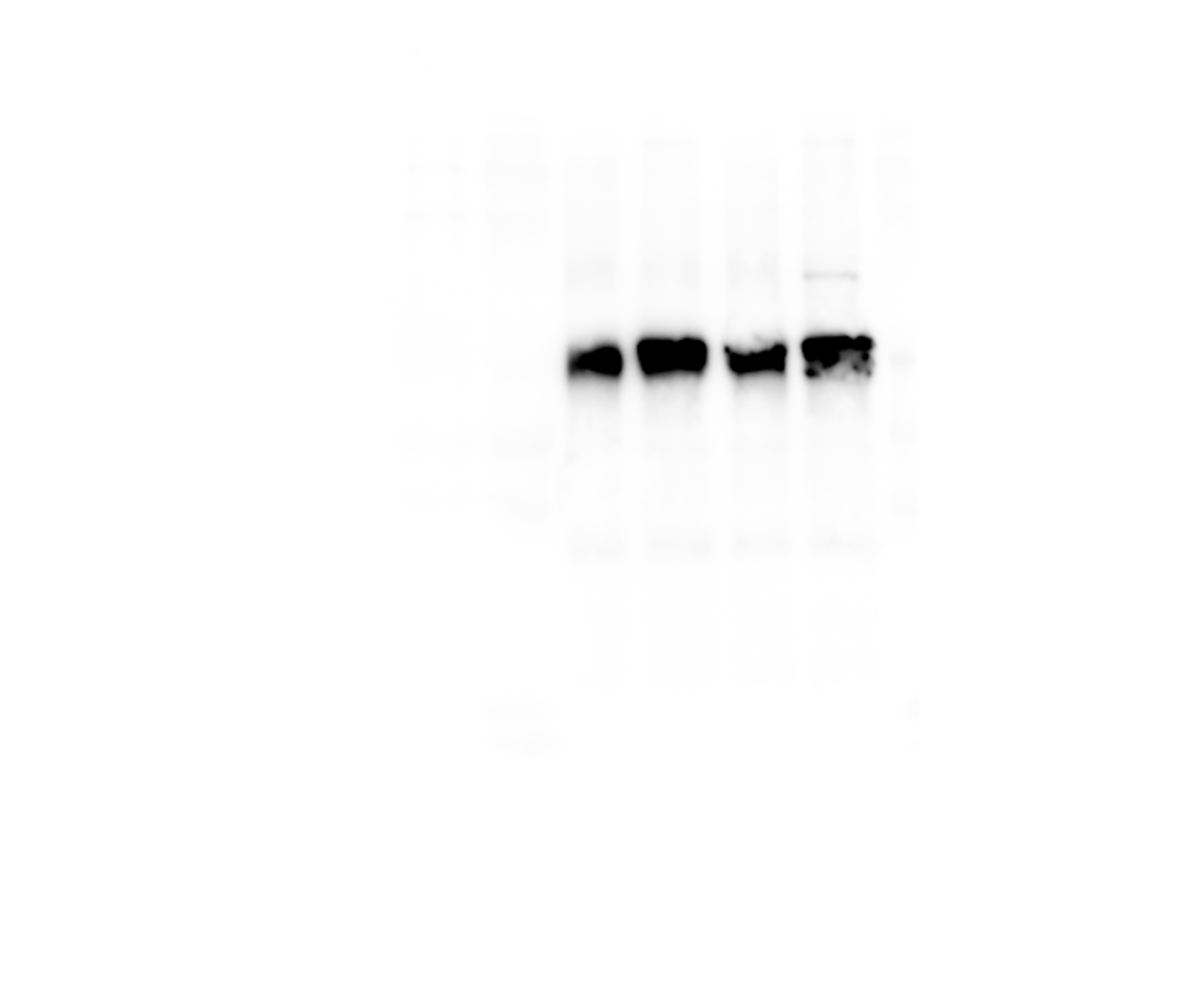

Supplement: Supplementary file 4 — Supplementary Material 4. [file 12964_2024_1770_MOESM4_ESM.zip › SENP3 TAM WB/WB-Figure4/B M0 M2 EndoIP/2023-02-18 ─┌╘┤IP shNC shSENP3 IRF4/IP IRF4 0222/IP IRF4 5S 0222.Tif]

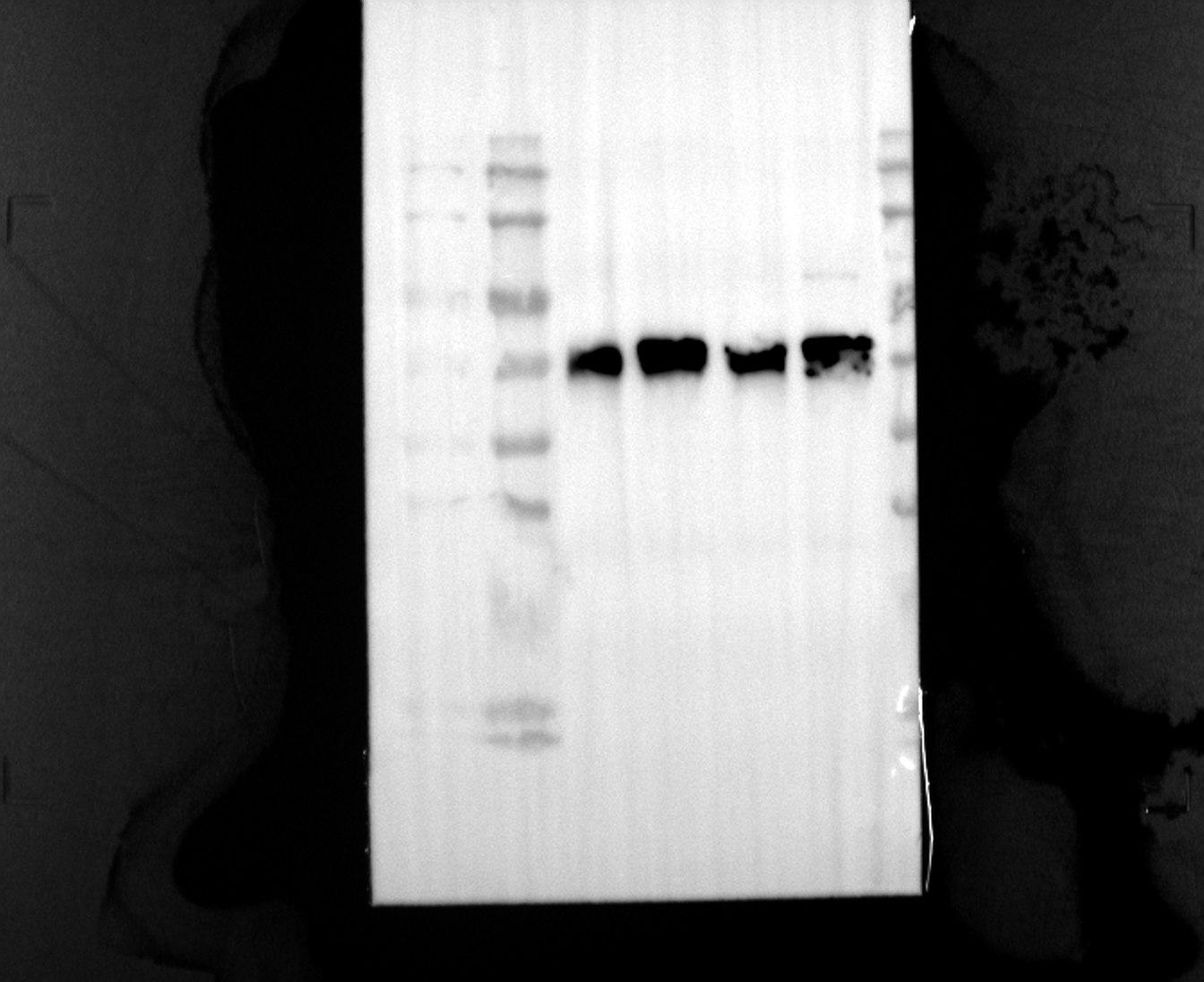

Supplement: Supplementary file 4 — Supplementary Material 4. [file 12964_2024_1770_MOESM4_ESM.zip › SENP3 TAM WB/WB-Figure4/B M0 M2 EndoIP/2023-02-18 ─┌╘┤IP shNC shSENP3 IRF4/IP IRF4 0222/IP IRF4 5S M 0222.Tif]

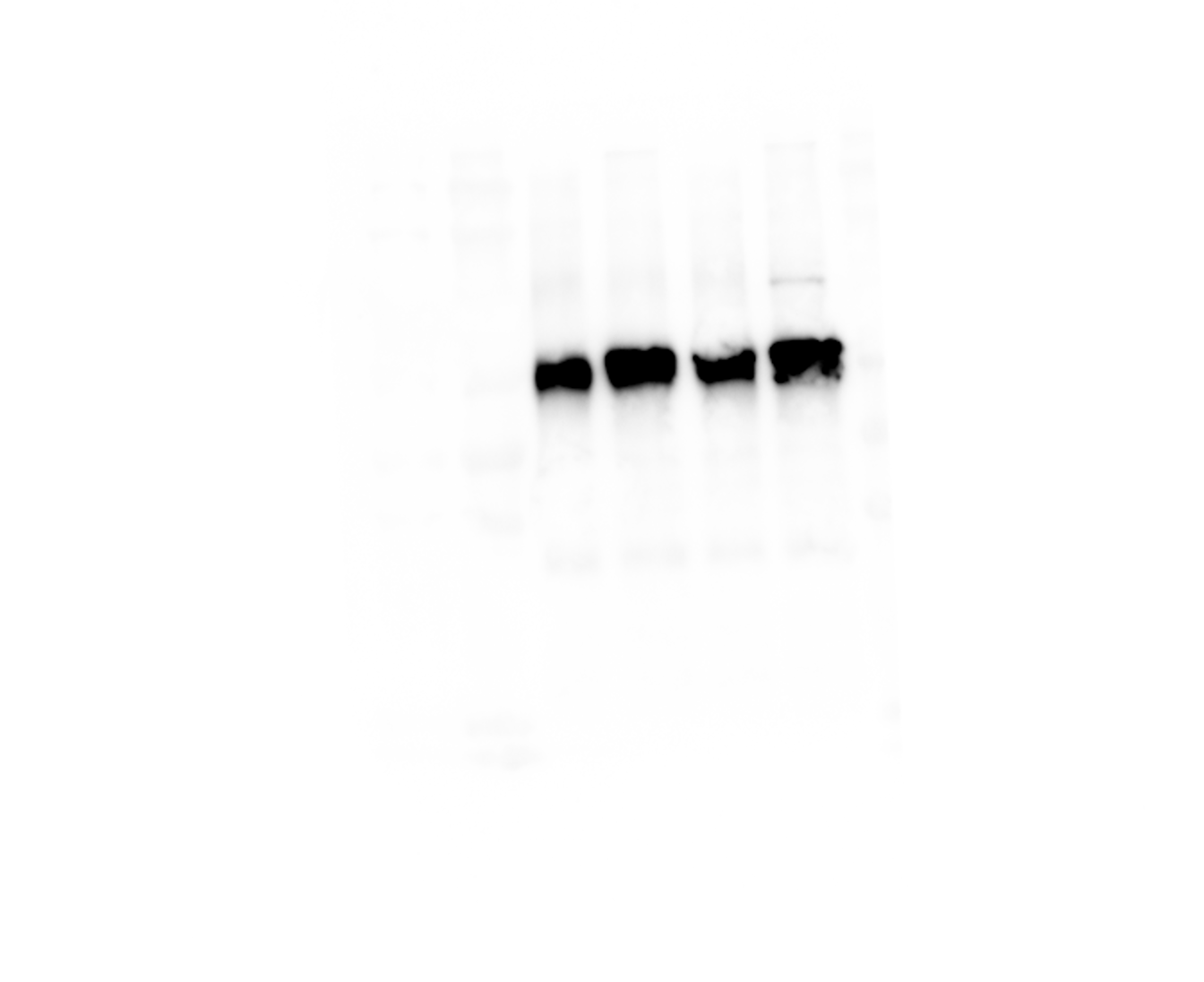

Supplement: Supplementary file 4 — Supplementary Material 4. [file 12964_2024_1770_MOESM4_ESM.zip › SENP3 TAM WB/WB-Figure4/B M0 M2 EndoIP/2023-02-18 ─┌╘┤IP shNC shSENP3 IRF4/IP IRF4 0222/IP IRF4 6S 0222.Tif]

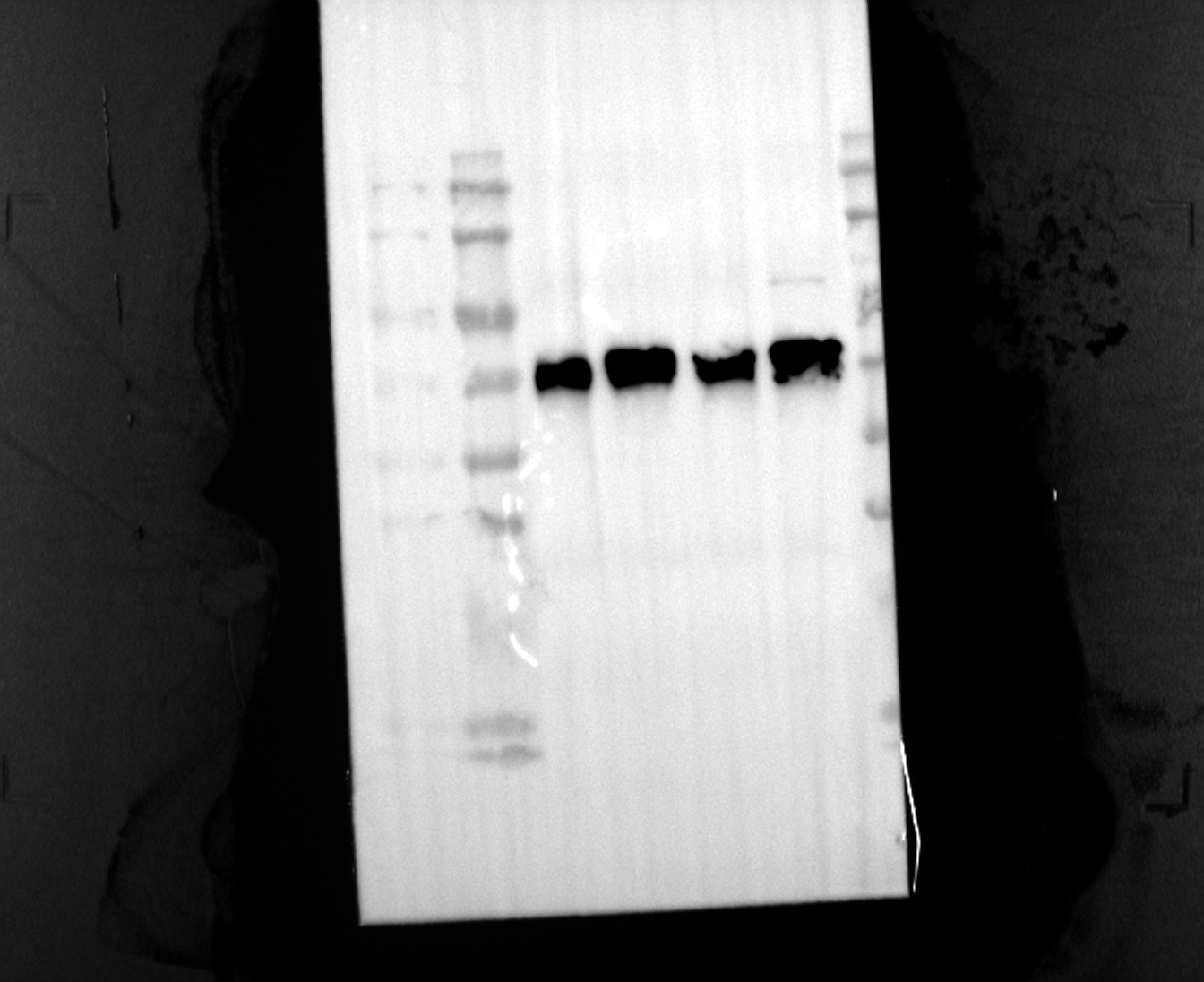

Supplement: Supplementary file 4 — Supplementary Material 4. [file 12964_2024_1770_MOESM4_ESM.zip › SENP3 TAM WB/WB-Figure4/B M0 M2 EndoIP/2023-02-18 ─┌╘┤IP shNC shSENP3 IRF4/IP IRF4 0222/IP IRF4 6S M 0222.Tif]

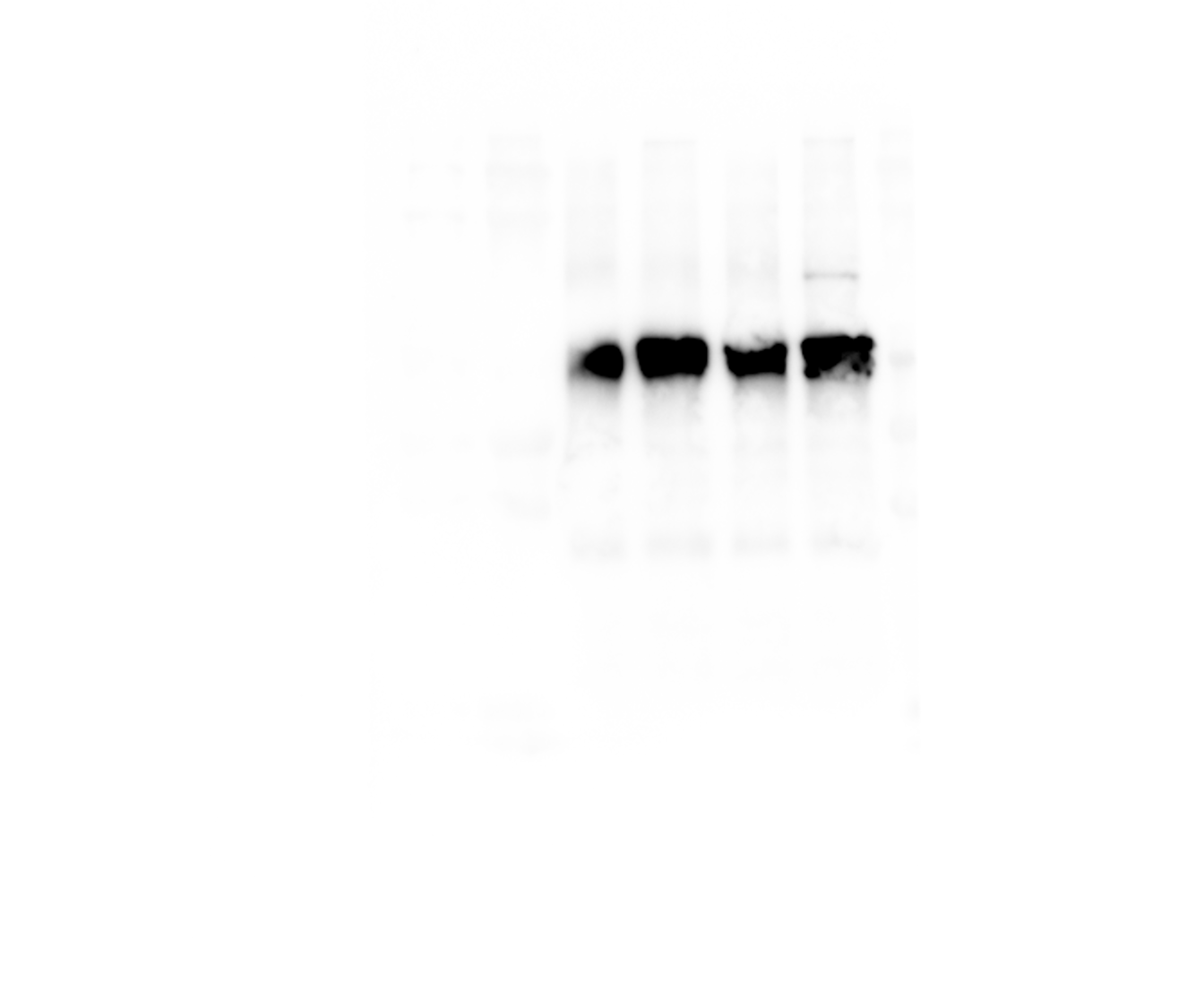

Supplement: Supplementary file 4 — Supplementary Material 4. [file 12964_2024_1770_MOESM4_ESM.zip › SENP3 TAM WB/WB-Figure4/B M0 M2 EndoIP/2023-02-18 ─┌╘┤IP shNC shSENP3 IRF4/IP IRF4 0222/IP IRF4 8S 0222.Tif]

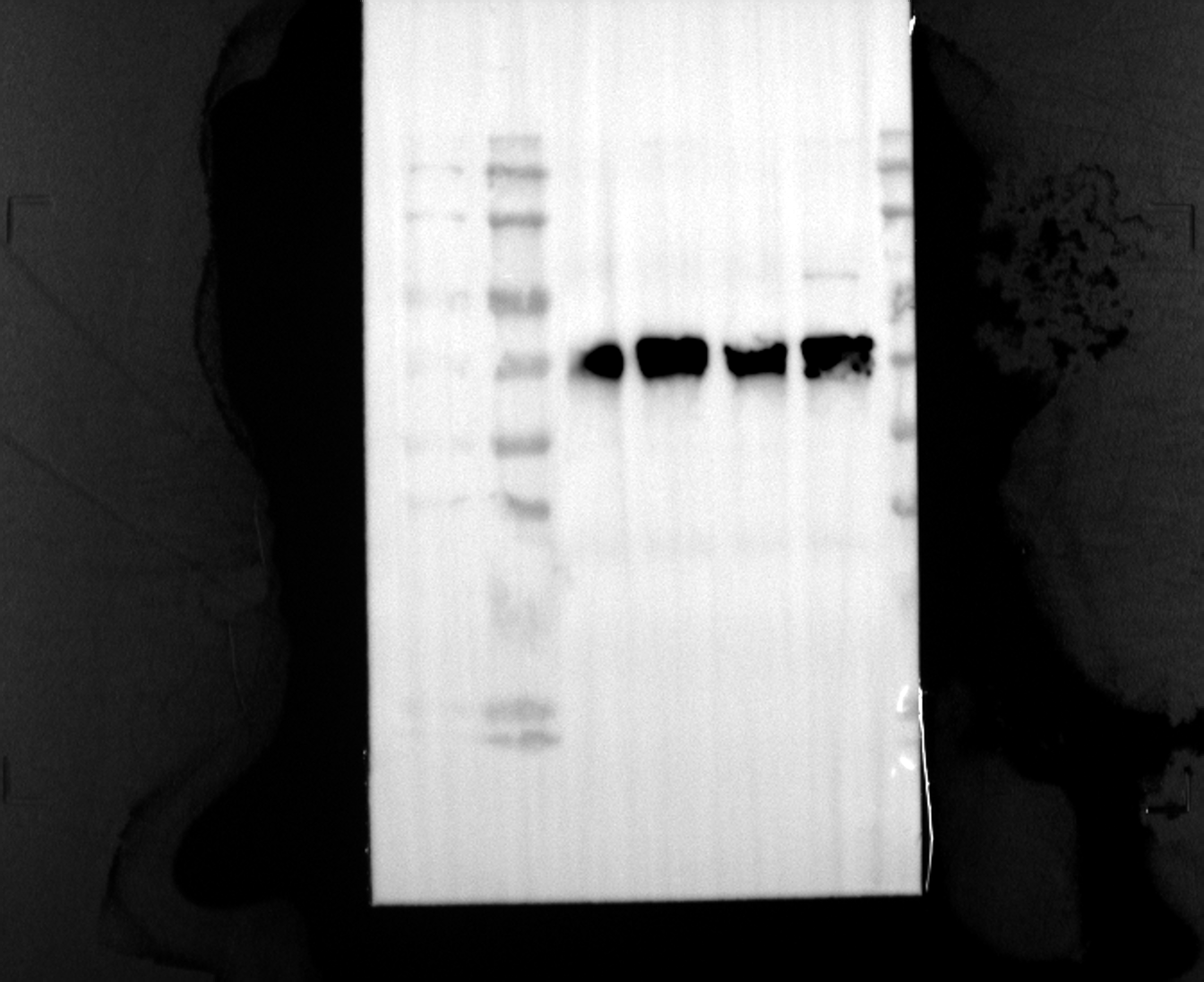

Supplement: Supplementary file 4 — Supplementary Material 4. [file 12964_2024_1770_MOESM4_ESM.zip › SENP3 TAM WB/WB-Figure4/B M0 M2 EndoIP/2023-02-18 ─┌╘┤IP shNC shSENP3 IRF4/IP IRF4 0222/IP IRF4 8S M 0222.Tif]

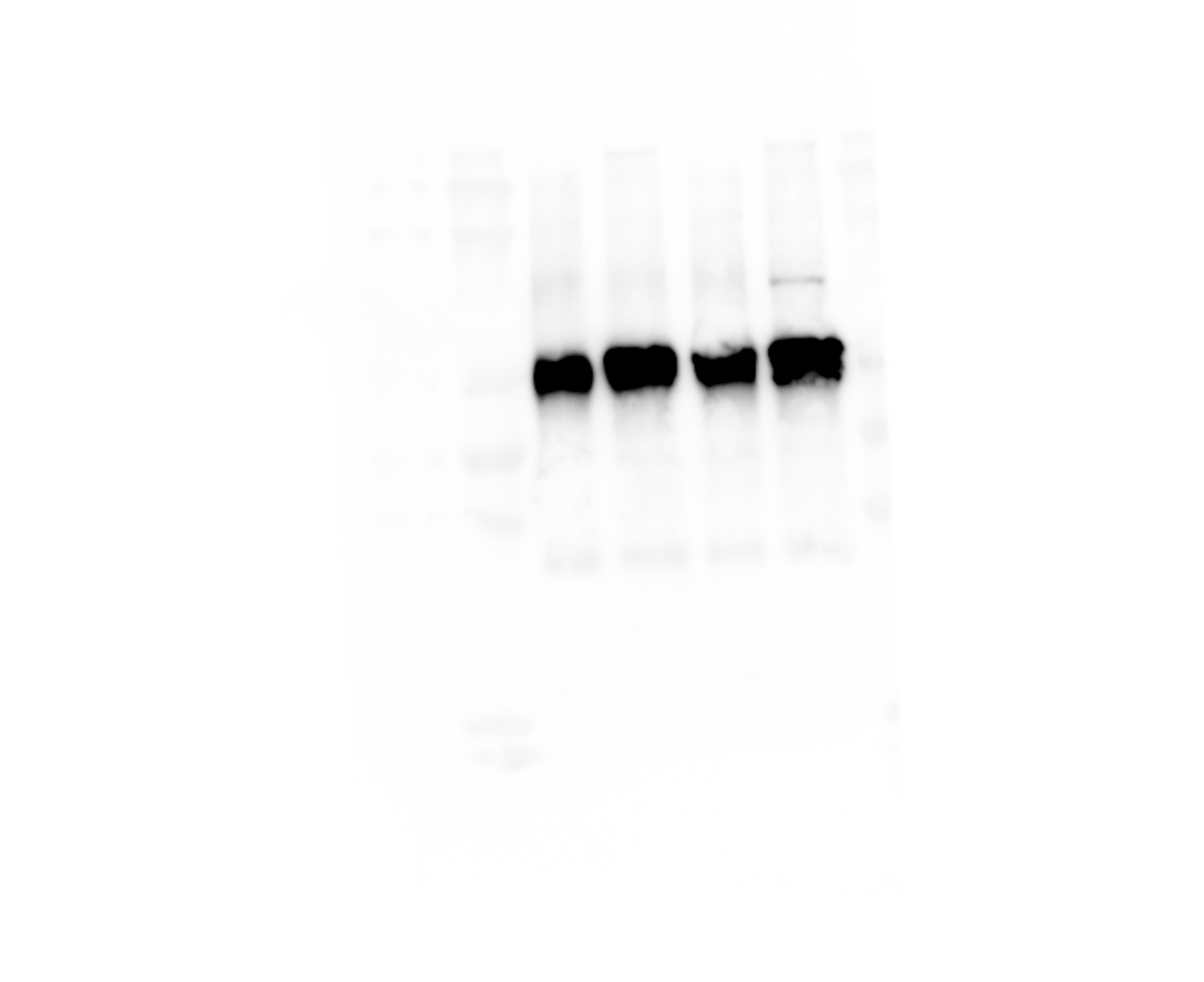

Supplement: Supplementary file 4 — Supplementary Material 4. [file 12964_2024_1770_MOESM4_ESM.zip › SENP3 TAM WB/WB-Figure4/B M0 M2 EndoIP/2023-02-18 ─┌╘┤IP shNC shSENP3 IRF4/IP IRF4 0222/IP IRF4 9S 0222.Tif]

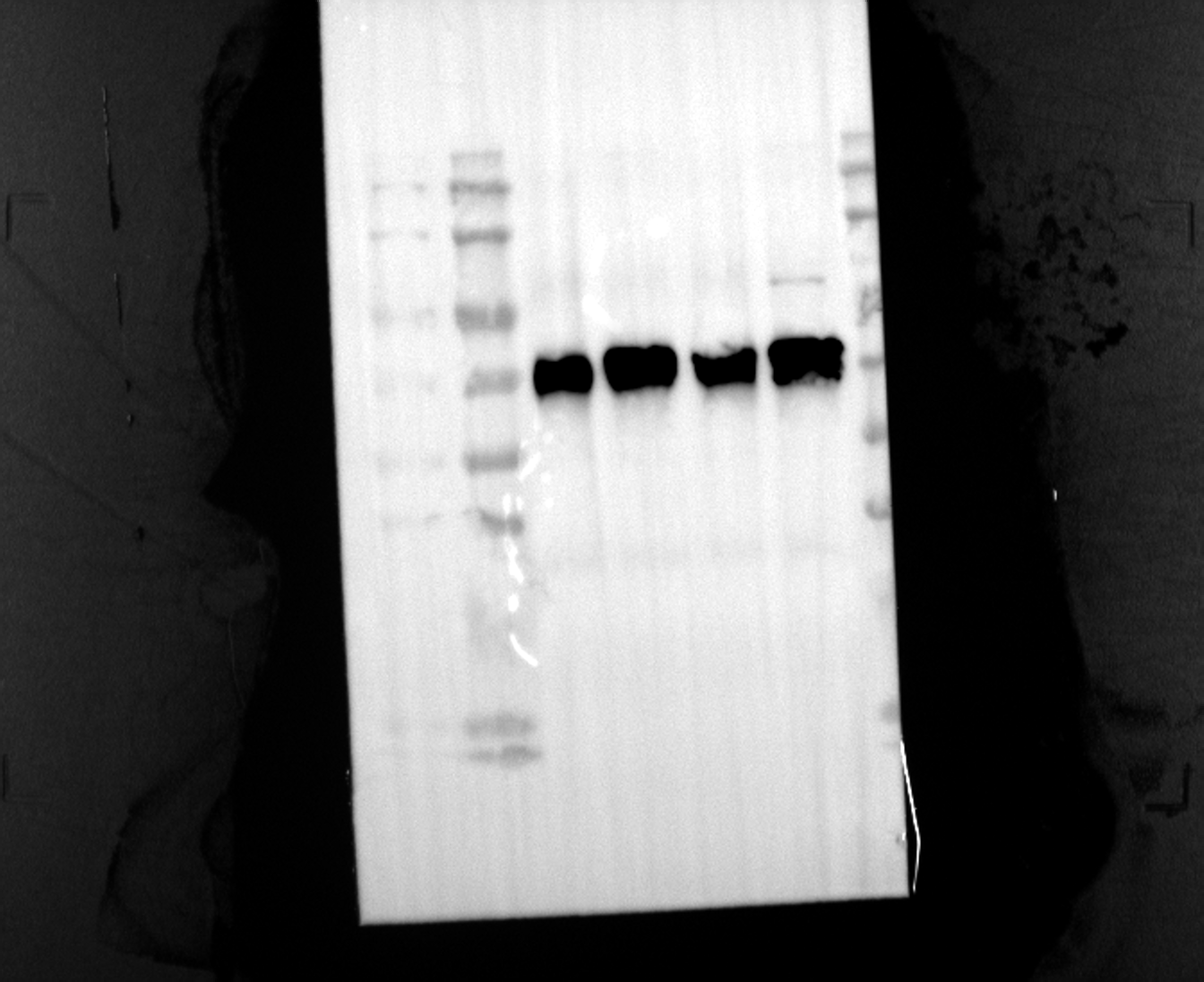

Supplement: Supplementary file 4 — Supplementary Material 4. [file 12964_2024_1770_MOESM4_ESM.zip › SENP3 TAM WB/WB-Figure4/B M0 M2 EndoIP/2023-02-18 ─┌╘┤IP shNC shSENP3 IRF4/IP IRF4 0222/IP IRF4 9S M 0222.Tif]

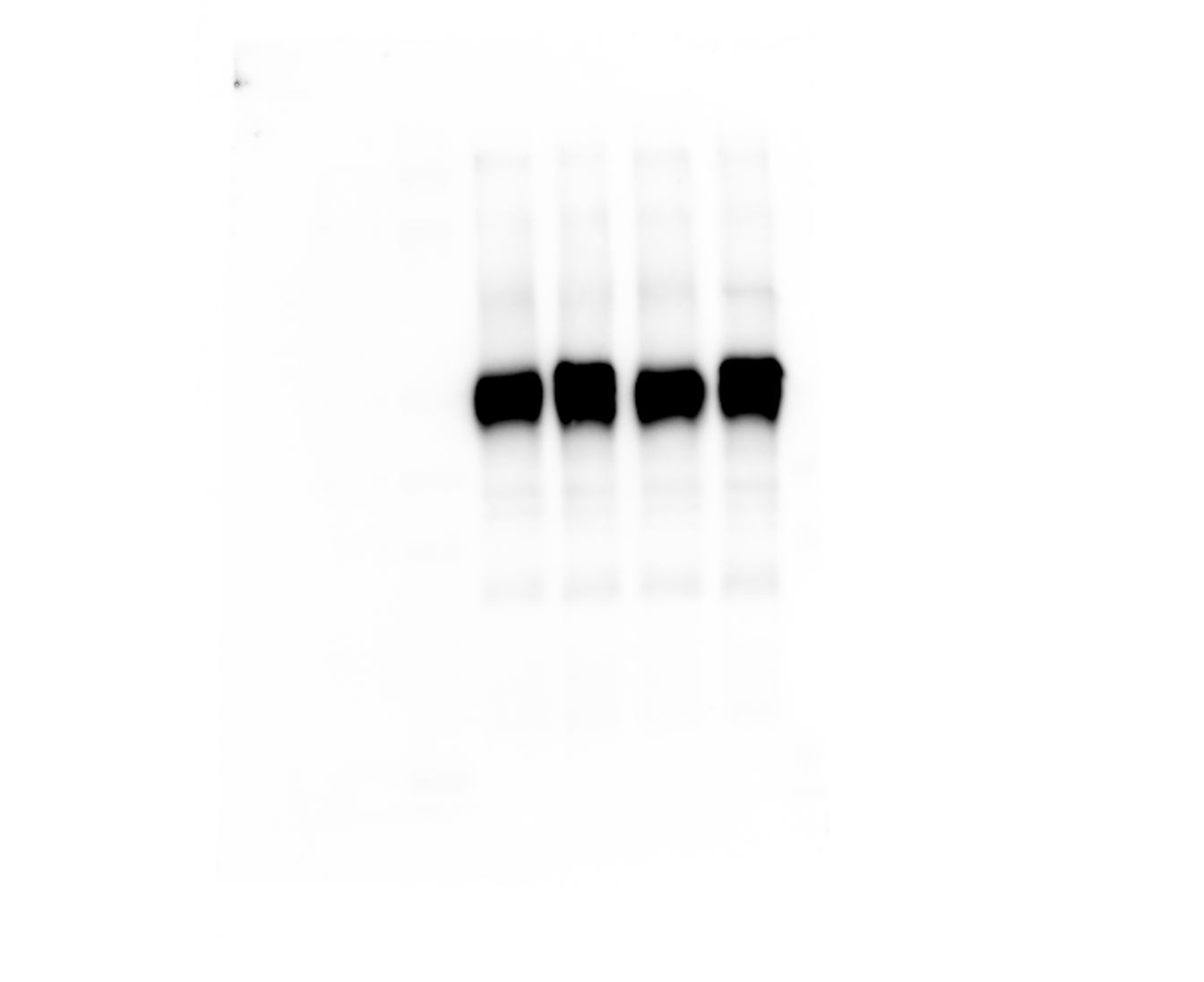

Supplement: Supplementary file 4 — Supplementary Material 4. [file 12964_2024_1770_MOESM4_ESM.zip › SENP3 TAM WB/WB-Figure4/B M0 M2 EndoIP/2023-02-18 ─┌╘┤IP shNC shSENP3 IRF4/IP IRF4 0224/IP IRF4 0224 30S.Tif]
