# Supplementary material for: Suppression of SENP3 enhances macrophage alternative activation by mediating IRF4 de-SUMOylation in ESCC progression
Source: Cell Commun Signal. 2024 Aug 9;22:395. doi: 10.1186/s12964-024-01770-z (PMC11312810; doi:10.1186/s12964-024-01770-z)
Supplement: Supplementary file 5 — Supplementary Material 5. [file 12964_2024_1770_MOESM5_ESM.docx]

**Suppressing SENP3 enhances Macrophage Alternative Activation by Mediating IRF4 de-SUMOylation in ESCC Progression**

Shaoyuan Zhang, Jianmin Gu, Wenhan Wang, Linyi Sun, Tian Jiang, Xinyu Yang, Jun Yin, Miao Lin, Dong Lin, Hao Wang & Lijie Tan

***Supplementary Materials***

**Antibody List**

| **Antibody** | **Company** | **Cat. No.** |
| --- | --- | --- |
| Anti-SENP3 Rabbit mAb | Cell Signaling Technology | #5591S |
| Anti-SUMO-2/3 Rabbit mAb | Cell Signaling Technology | #4971 |
| Anti-IRF-4 Rabbit mAb | Cell Signaling Technology | #62834 |
| Anti-FLAG antibody | Sigma-Aldrich | F1804 |
| Anti-HA antibody | Sigma-Aldrich | H6908 |
| Anti-SUMO2/3 antibody | Sigma-Aldrich | SAB3500488 |
| Myc-Tag antibody | ABclonal | AE010 |
| Anti-Ubiquitin Antibody | Abcam | ab134953 |
| Beta Actin Monoclonal antibody | Proteintech | 60004-1-Ig |
| SENP3 Antibody (For IHC) | Novus Biologicals | NBP2-20294 |
| Anti-Ki67 Antibody (For IHC) | Abcam | ab15580 |
| Anti-CD68 Antibody (For IHC) | Abcam | ab125212 |
| Anti-Mannose Receptor (CD206) Antibody (For IHC) | Abcam | ab64693 |
| Brilliant Violet 421™ anti-human CD206 (MMR) Antibody (For Flow) | BioLegend, Inc | 321126 |
| APC/Cyanine7 anti-human CD68 Antibody (For Flow) | BioLegend, Inc | 333822 |
| PE/Cyanine7 anti-mouse/human CD11b Antibody (For Flow) | BioLegend, Inc | 101216 |
| PE anti-human CD86 Antibody (For Flow) | BioLegend, Inc | 381010 |
| APC anti-human CD163 Antibody  (For Flow) | BioLegend, Inc | 326510 |
| BD Horizon™ BV421 Mouse Anti-Human CD45 (For Flow) | BD | 563879 |
| FITC anti-mouse F4/80 Recombinant Antibody | BioLegend, Inc | 157310 |

**Chemicals, peptides, and recombinant proteins**

| **Chemicals, peptides, and recombinant proteins** | **Company** | **Cat. No.** |
| --- | --- | --- |
| Phorbol 12-myristate 13-acetate (PMA) | Sigma-Aldrich | P1585 |
| Cycloheximide (CHX) | Sigma-Aldrich | 01810 |
| 4-Nitroquinoline N-oxide | Sigma-Aldrich | N8141 |
| Recombinant Protein (4-NQO) (Interleukin-4 Human) | Sangon Biotech | C610006 |
| Recombinant Human Interleukin-13 | Sangon Biotech | C620012 |
| Recombinant Murine Interleukin-4 | Sangon Biotech | C600050 |
| Anti-Flag Magnetic Beads | MCE | HY-K0207 |
| Protein A/G Magnetic Beads | MCE | HY-K0202 |
| CD14 MicroBeads, human | Miltenyi Biotec | 130-050-201 |

**RT-PCR Primer**

| **Gene** | **Primer** |  |
| --- | --- | --- |
| Human ARG1 | Forward | 5'-GTGGAAACTTGCATGGACAAC-3' |
|  | Reverse | 5'-AATCCTGGCACATCGGGAATC-3' |
| Human CD206 | Forward | 5'-GGGTTGCTATCACTCTCTATGC-3' |
|  | Reverse | 5'-TTTCTTGTCTGTTGCCGTAGTT-3' |
| Human SENP3 | Forward | 5'-GGATGCTGCTCTACTCAAAAAGC-3' |
|  | Reverse | 5'-GGGAGTCAAAACGACAACAGG-3' |
| Human CD163 | Forward | 5'-TTTGTCAACTTGAGTCCCTTCAC-3' |
|  | Reverse | 5'-TCCCGCTACACTTGTTTTCAC-3' |
| Human CCL22 | Forward | 5'-ATTACGTCCGTTACCGTCTGC-3' |
|  | Reverse | 5'-TCCCTGAAGGTTAGCAACACC-3' |
| Human TNF-α | Forward | 5'-GAGGCCAAGCCCTGGTATG-3' |
|  | Reverse | 5'-CGGGCCGATTGATCTCAGC-3' |
| Human IL-1β | Forward | 5'-TTCGACACATGGGATAACGAGG-3' |
|  | Reverse | 5'-TTTTTGCTGTGAGTCCCGGAG-3' |
| Human IL-10 | Forward | 5'-GACTTTAAGGGTTACCTGGGTTG-3' |
|  | Reverse | 5'-TCACATGCGCCTTGATGTCTG-3' |

**shRNA**

| **shRNA** | **Target sequence (bold)** & flanker sequence area position interference score | **Area** | **Location** |
| --- | --- | --- | --- |
| hSENP3[shRNA#1] | TTTTTTTCTT**TGAGAGAATACTTGTTGATTT**CTGATGTGCA | 3' UTR | 2164-2184 |

**Experimental models**

| **Antibody** | **Company** | **Cat. No.** |
| --- | --- | --- |
| LyZ2-Cre mice | The Jackson Laboratory | #004781 |
| Senp3 fl/fl mice | Model Animal Research Center of Nanjing University | T005127 |

**Software**

| **Software** | **Company** | **Resource** |
| --- | --- | --- |
| R 4.2.1/R Studio | R Foundation | https://www.r-project.org/ |
| TissueFAXS Viewer | TissueGnostics GmbH | https://tissuegnostics.com/products |
| TissueQuest | TissueGnostics GmbH | https://tissuegnostics.com/products |
| GraphPad 9.4.1 | GraphPad Software | https://www.graphpad.com/ |
| ImageJ | National Institutes of Health | https://imagej.net/ij/index.html |
| FlowJo 10.8.1 | Becton, Dickinson, and Company | https://www.flowjo.com/ |

**Cell lines**

| **Cell lines** | **Company** | **Cat. No.** |
| --- | --- | --- |
| THP-1 | ATCC | TIB-202 |
| HEK-293T | ATCC | CRL-3216 |
| KYSE150 | Cell Bank/Stem Cell Bank, Chinese Academy of Sciences | TCHu 236 |
| Eca109 | Cell Bank/Stem Cell Bank, Chinese Academy of Sciences | TCHu 69 |
| TE-1 | Cell Bank/Stem Cell Bank, Chinese Academy of Sciences | TCHu 89 |

**Supplementary Table 1 Baseline clinicopathological information of 270 patients in Tumor MicroArray Analysis**

|  |  |
| --- | --- |
|  | **n=270** |
| **Gender (%)** |  |
| Female | 59 (21.9) |
| Male | 211 (78.1) |
| **Age** | 64.2 (8.0) |
| **Pathological T Stage (%)** |  |
| T1a | 21 (7.8) |
| T1b | 65 (24.1) |
| T2 | 60 (22.2) |
| T3-4a | 124 (45.9) |
| **Pathological N Stage (%)** |  |
| N0 | 176 (65.2) |
| N1 | 64 (23.7) |
| N2 | 24 (8.9) |
| N3 | 6 (2.2) |
| **Lympho-vascular invasion (%)** |  |
| Yes | 62 (23.0) |
| No | 208 (77.0) |
| **Neural invasion (%)** |  |
| Yes | 82 (30.4) |
| No | 188 (69.6) |
| **Total Lymph Node Dissection** | 25.4 (9.8) |
| **Differentiation (%)** |  |
| High | 7 (2.6) |
| Moderate | 177 (65.6) |
| Poor/Undifferentiation | 86 (31.9) |
| **Primary Tumor SUVmax** | 11.6 (6.7) |
| **Surgical Approach (%)** |  |
| Ivor Lewis | 39 (14.4) |
| McKeown | 201 (74.4) |
| Sweet | 30 (11.1) |
| **Minimally Invasive (%)** |  |
| MIE | 201 (74.4) |
| Open | 69 (25.6) |

Data in the table are described as number (%) or mean (SD);

**Supplementary Table 2 Relationship between relative SENP3 expression in CD206+ cells and clinicopathology information**

| **Variable** | **Relatively Low SENP3 expression in CD206+ cells** | **Relatively High SENP3 expression in CD206+ cells** | **P-Value** |
| --- | --- | --- | --- |
|  | (n=135) | (n=135) |  |
| **Gender (%)** |  |  | 0.883 |
| Female | 30 (22.2) | 29 (21.5) |  |
| Male | 105 (77.8) | 106 (78.5) |  |
| **Age** | 65.0 [60.0, 72.0] | 64.0 [59.0, 69.0] | 0.101 |
| **Total Lymph Node Dissection** | 25.0 [18.0, 32.0] | 24.0 [19.0, 30.5] | 0.390 |
| **pT Stage (%)** |  |  | **0.001*** |
| T1a | 7 (5.2) | 14 (10.4) |  |
| T1b | 22 (16.3) | 43 (31.9) |  |
| T2 | 28 (20.7) | 32 (23.7) |  |
| T3 | 78 (57.8) | 46 (34.1) |  |
| **pN Stage (%)** |  |  | **0.038*** |
| N0 | 78 (57.8) | 98 (72.6) |  |
| N1 | 39 (28.9) | 25 (18.5) |  |
| N2+ | 18 (13.3) | 12 (8.9) |  |
| **Differentiation (%)** |  |  | 0.414 |
| High | 3 (2.2) | 4 (3.0) |  |
| Moderate | 84 (62.2) | 93 (68.9) |  |
| Poor/Undifferentiation | 48 (35.6) | 38 (28.1) |  |
| **SUVmax** | 13.0 [8.0, 17.4] | 9.4 [5.4, 13.6] | **<0.001*** |
| **Ki-67 Positive Rate** | 0.6 [0.4, 0.7] | 0.6 [0.4, 0.7] | 0.680 |

Data in the table are described as number (%) or mean [IQR];

**Supplementary Figure 1**


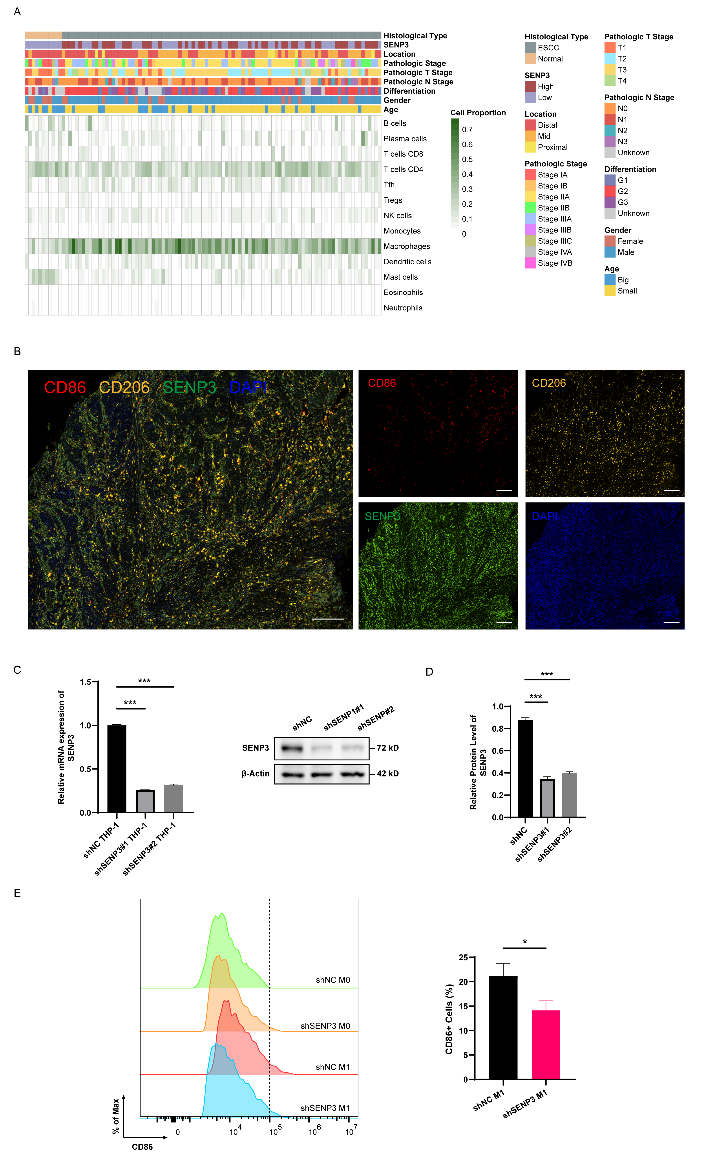


**FigureS1 Legend**

(A) Immune cell ratio analysis of ESCC patients and normal patients in TCGA-ESCA by CibersortX

(B) Representative immunofluorescence of SENP3, CD206, and CD86 in esophageal squamous cell carcinoma tissues

(C) The mRNA levels of SENP3 in THP-1 of shNC and shSENP3

(D) The Protein levels of SENP3 in THP-1 of shNC and shSENP3

(E) Flow cytometric analysis of shNC and shSENP3-induced differentiation into M1 macrophages

**Supplementary Figure 2**


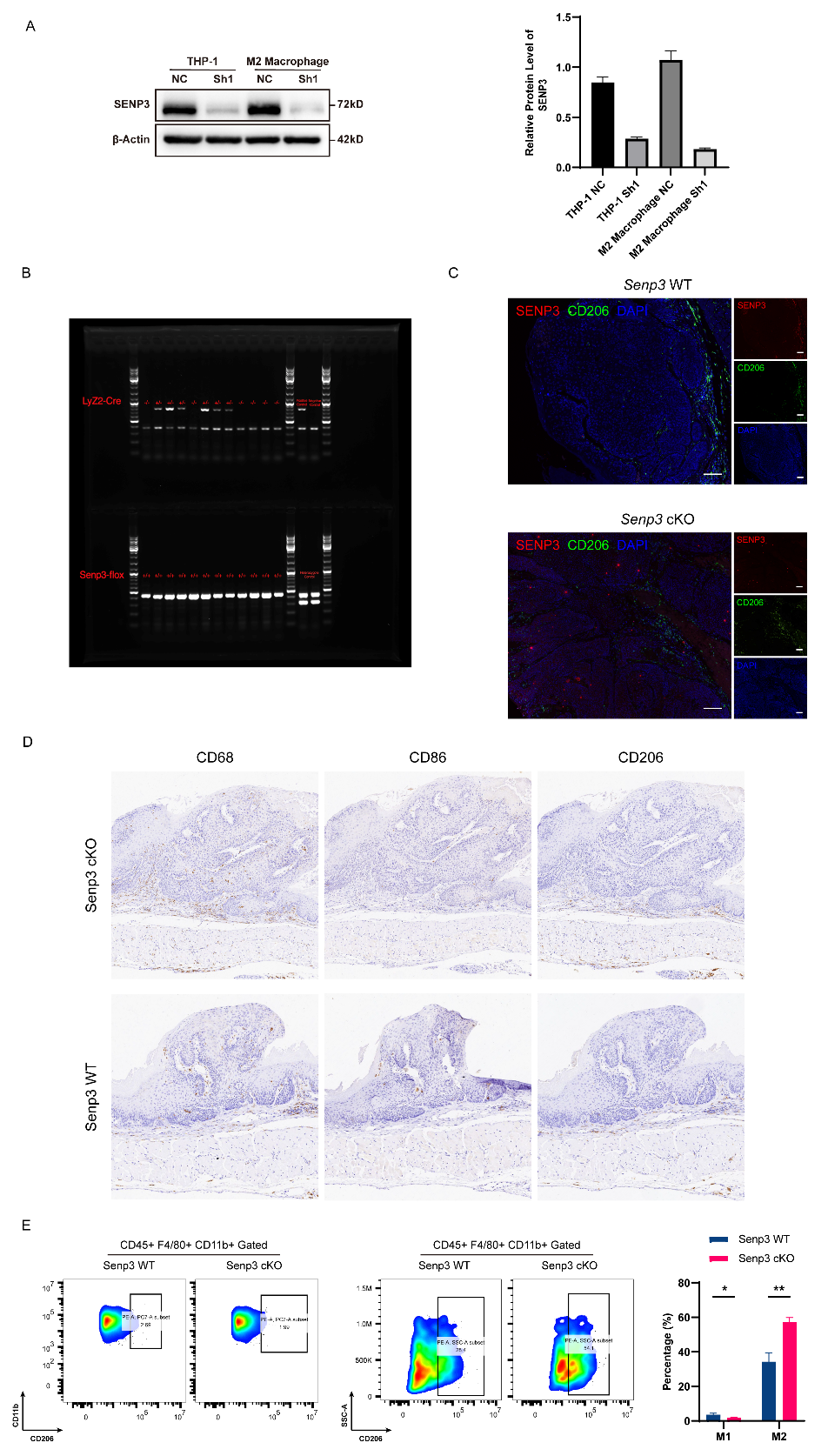


**FigureS2 Legend**

(A) The Protein levels of SENP3 in THP-1 and M2 Macrophages of shNC and shSENP3

(B) Genotype identification of Senp3 WT mice and Senp3 cKO mice

(C) Multiple immunofluorescence staining of Senp3 and Cd206 was performed after 4-NQO-induced ESCC in Senp3 WT and Senp3 cKO mice

(D) Immunohistochemical staining of CD68, CD86 and CD206 in esophageal squamous cell carcinoma tissues of Senp3 WT and Senp cKO mice

(E) Flow cytometry analysis of BMDMs induced into M1 and M2 macrophages in vitro in Senp3 WT and Senp cKO mice, respectively

**Supplementary Figure 3**


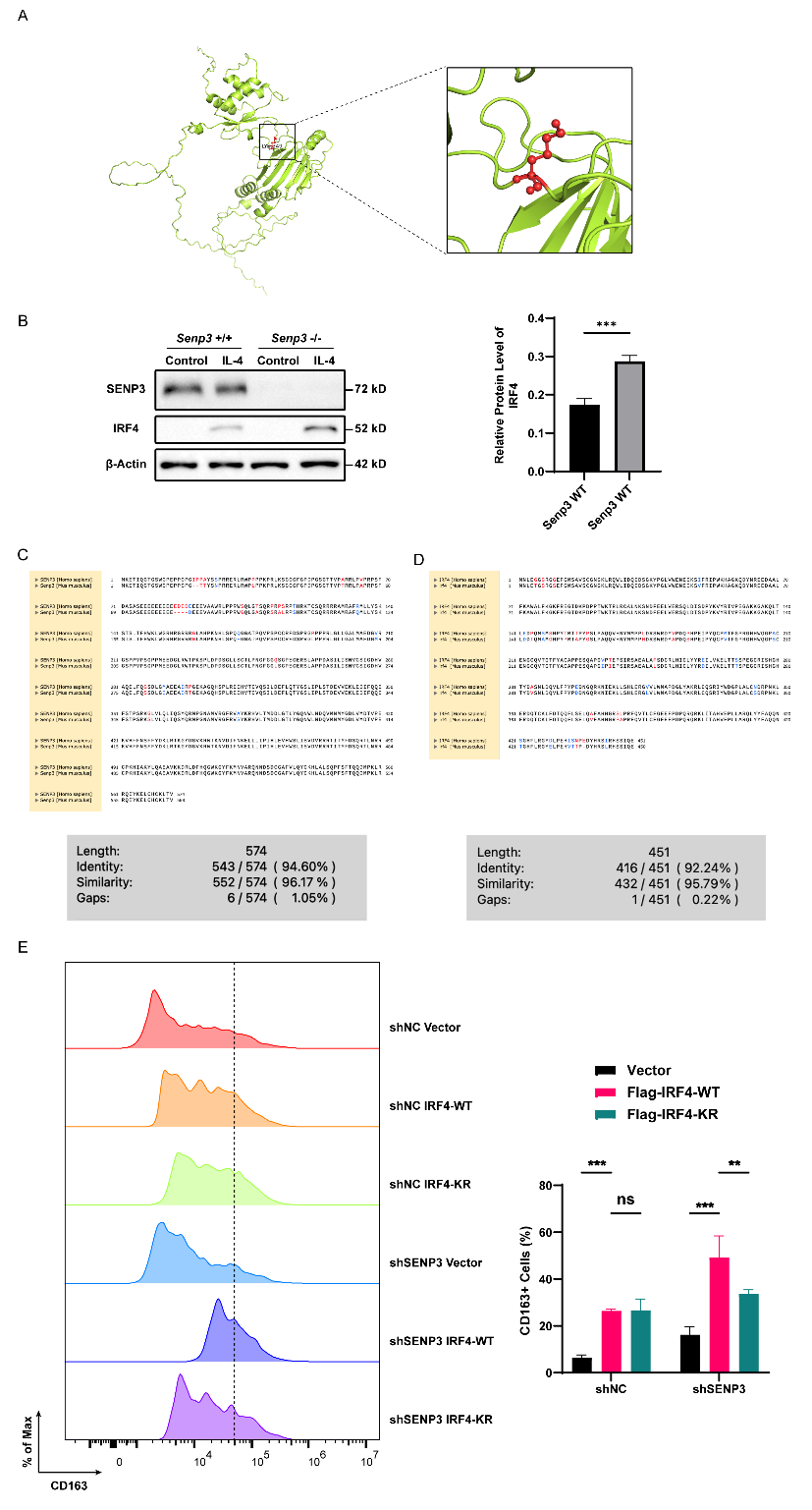


**FigureS3 Legend**

(A) Spatial structure and K349 site of interferon regulatory factor 4 (IRF4)

(B) Protein expression levels of IRF4 after BMDM stimulation in vitro in Senp3 WT and Senp3 cKO mice

(C) Homologous sequence analysis of human SENP3 and mouse Senp3

(D) Homologous sequence analysis of human IRF4 and mouse Irf4

(E) Flow cytometry analysis of the proportion of CD163+ cells in shNC and shSENP3 groups after transfection with Vector, Flag-IRF4-WT, and Flag-IRF4-KR.
